# Cross-Omics Analysis of Fenugreek Supplementation Reveals Beneficial Effects Are Caused by Gut Microbiome Changes Not Mammalian Host Physiology

**DOI:** 10.3390/ijms23073654

**Published:** 2022-03-26

**Authors:** Katarina A. Jones, Allison J. Richard, J. Michael Salbaum, Susan Newman, Richard Carmouche, Sara Webb, Annadora J. Bruce-Keller, Jacqueline M. Stephens, Shawn R. Campagna

**Affiliations:** 1 Department of Chemistry, University of Tennessee, Knoxville, TN 37916, USA; kjone166@vols.utk.edu; 2 Pennington Biomedical Research Center, Louisiana State University System, Baton Rouge, LA 70808, USA; allison.richard@pbrc.edu (A.J.R.); michael.salbaum@pbrc.edu (J.M.S.); susan.newman@pbrc.edu (S.N.); richard.carmouche@pbrc.edu (R.C.); sara.webb@pbrc.edu (S.W.); annadora.bruce-keller@pbrc.edu (A.J.B.-K.); jsteph1@lsu.edu (J.M.S.); 3 Biological and Small Molecule Mass Spectrometry Core, University of Tennessee, Knoxville, TN 37916, USA

**Keywords:** metabolomics, fenugreek, UHPLC-HRMS, C57BL/6J mice, metagenomics

## Abstract

Herbal remedies are increasing in popularity as treatments for metabolic conditions such as obesity and Type 2 Diabetes. One potential therapeutic option is fenugreek seeds (*Trigonella foenum-graecum*), which have been used for treating high cholesterol and Type 2 diabetes. A proposed mechanism for these benefits is through alterations in the microbiome, which impact mammalian host metabolic function. This study used untargeted metabolomics to investigate the fenugreek-induced alterations in the intestinal, liver, and serum profiles of mice fed either a 60% high-fat or low-fat control diet each with or without fenugreek supplementation (2% *w*/*w*) for 14 weeks. Metagenomic analyses of intestinal contents found significant alterations in the relative composition of the gut microbiome resulting from fenugreek supplementation. Specifically, Verrucomicrobia, a phylum containing beneficial bacteria which are correlated with health benefits, increased in relative abundance with fenugreek. Metabolomics partial least squares discriminant analysis revealed substantial fenugreek-induced changes in the large intestines. However, it was observed that while the magnitude of changes was less, significant modifications were present in the liver tissues resulting from fenugreek supplementation. Further analyses revealed metabolic processes affected by fenugreek and showed broad ranging impacts in multiple pathways, including carnitine biosynthesis, cholesterol and bile acid metabolism, and arginine biosynthesis. These pathways may play important roles in the beneficial effects of fenugreek.

## 1. Introduction

Obesity is a global epidemic which has been steadily increasing over the last few decades and now affects more than two million people worldwide [1]. Accompanying obesity are several comorbidities including, but not limited to, metabolic and endocrine disorders such as Type 2 diabetes, dyslipidemia, and cardiovascular disease, and it is frequently linked to mental health, specifically anxiety and depression [2,3,4,5,6]. These diseases are often related to an unhealthy diet, particularly, high fat or high caloric diets. Despite this being well known, it is often unattainable for subjects with obesity to have consistently healthy diets due to societal and physiological barriers. To combat this growing problem, ongoing research aims to develop more effective treatments. However, many pharmacological therapies are limited by high costs and detrimental side effects [7,8,9]. Herbal remedies and specific plant materials, which have traditionally been used for obesity and metabolic diseases, are an alternative approach to therapy development [10,11]. Many of these traditional remedies include fenugreek (*Trigonella foenum-graecum*), an annual herbaceous plant belonging to the *Fabaceae* (also known as the *Leguminoseae*) family [11,12,13].

Fenugreek is grown in several regions globally, including parts of Africa, Asia, and Europe and is used for both medicinal properties and for food purposes, as a seasoning [12,13,14]. It has been used for its anti-diabetic, anti-inflammatory, anti-cancer, anti-hyperlipidemic, and hepatoprotective properties [13,14,15]. The anti-diabetic effects of fenugreek have been suggested to result from the inhibition of glucose absorption in the intestines and/or insulinotropic activity [15]. Fenugreek seeds contain several bioactive compounds including diosgenin, trigonelline, galactomannan, and 4-hydroxyisoleucine, among others; however, the source of the beneficial effects of fenugreek is not well known [11,12,13]. Studies have shown that fenugreek supplementation is sufficient to alter intestinal microbiota and thus influence the host physiology [15,16,17]. Microbiome changes as a result of fenugreek diet supplementation are a possible explanation for the therapeutic effects of fenugreek. A previous study using metabolomic and microbiome analyses has shown that microbiota from the *Lachnospiraceae* and *Runinococcacea* families (in the Firmicutes phylum) increase coprostanol production in healthy humans and thus reduces blood cholesterol levels through increased fecal cholesterol excretion [18]. More recent microbiome data demonstrate that a fenugreek-supplemented high fat diet was able to alter the expression of several taxa belonging to these two families, suggesting that fenugreek may encourage fecal fat excretion [15]. This may be a possible explanation for the anti-hyperlipidemic properties of fenugreek.

Metabolic health is influenced by gut microbiota and is often mutually beneficial to both microbiota and the host [15,19,20]. The gut microbiome is a complex heterogeneous mixture not only of primarily bacteria but also of other types of microbes, with the greatest concentration found in the large intestines [19]. Intestinal microbiota have several important roles including aiding in digestion and synthesis of vitamins, as well as neutralizing carcinogens, toxins, and pathogenic bacteria [15,21]. In healthy gut microbiomes, there is high diversity between bacterial species (alpha diversity), but the majority of bacteria belong to three phyla, specifically Gram-positive Firmicutes (60%), Gram-negative Bacteroidetes and Gram-positive Actinobacteria (combined ~10%) [19]. However, high fat and caloric diets, such as Western style diets which are high in fat, sucrose, and animal protein significantly alter the composition of the microbiome [19,22,23]. These diets, including Western style diets, result in decreased microbial diversity and an increase in pathogenic and pro-inflammatory producing bacteria [15,19,24]. These changes not only alter the microbiome but also are sufficient to cause detrimental metabolic effects [15]. Fenugreek seeds are known to alter the microbiome, and a previous study found that fenugreek was able to partially restore a healthy microbiome to mice fed a high fat diet, thus providing metabolic resiliency and improving metabolic health in mice [15].

This study was designed to determine how the structure and function of the mammalian host is altered by high fat diets and fenugreek seeds and the expected microbiome changes associated with these diets. High resolution mass spectrometry-based untargeted metabolomics was used to study male C57BL/6J mice that were fed either a 60% high fat diet or a nutritionally matched low fat control diet. A subset of each experimental group received diets supplemented with ground fenugreek seeds (2% *w*/*w*) for 14 weeks and the effects of the diets on the metabolome and intestinal microbiome were determined. Metabolomics permits high throughput analysis of all water-soluble small molecules, which allows for a systems-level phenotype analysis of the gut metabolome. To provide insight into circulating nutrients resulting from fenugreek supplementation, liver and serum were also analyzed. These data reveal significant alterations to the both the metabolic and microbiome profiles resulting from fenugreek supplementation. 

## 2. Results

### 2.1. Significant Differences between Small Intestine, Large Intestine, Liver, and Serum

**Figure 1:**
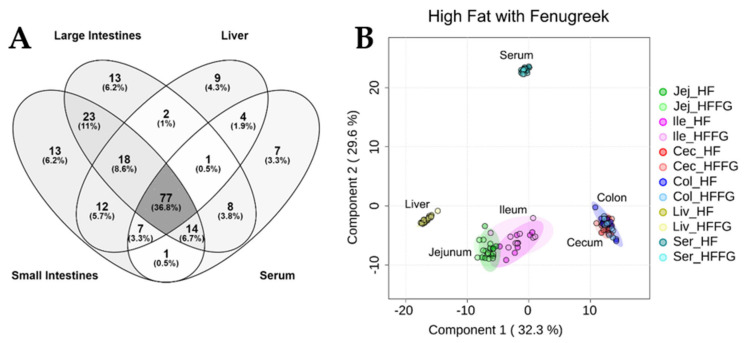
(**A**) Venn diagram depicting the overlap of identified metabolites between the small intestines, large intestines, liver, and serum. Of the identified metabolites, 36.8% were detected in all matrices; however, the least overlap was observed between large intestine, liver, and serum samples (0.5%) and between serum and small intestine samples (0.5%). (**B**) Partial least squares discriminant analysis (PLS-DA) comparing metabolomes associated with HF and HFFG diets for small intestines (jejunum and ileum), large intestines (cecum and colon), liver, and serum. Cross validation values can be found in Supplementary Table S1. Experimental replicates are shown with respective confidence intervals; HF and HFFG jejunum samples are dark and light green; HF and HFFG ileum samples are dark and light pink; HF and HFFG cecum samples are shown in dark and light red; HF and HFFG colon samples are shown in dark and light blue; HF and HFFG liver samples are dark and light yellow, and HF and HFFG serum samples are dark and light teal.

This study employed untargeted metabolomics to analyze the metabolic effects of fenugreek on the gut, liver, and serum of mice fed HF diet for 14 weeks. From these analyses, a total of 209 metabolites were identified by exact mass and retention time across the contents of four intestinal regions (jejunum, ileum, cecum, and colon), livers, and serum of mice fed either HF or CD diet, each with and without fenugreek for a total of 4 different diets (HF, HFFG, CD, and CDFG). Of these 209 metabolites, 77 (36.8%) were detected in the small intestine, large intestine, liver, and serum (Figure 1A). As anticipated, the largest influence on the metabolic profiles was due to the origin of the sample (jejunum, ileum, cecum, colon, liver, or serum), rather than the specific diet. PLS-DA analysis (Figure 1B), when including groups for different sample location and diet, shows four clear and distinct groupings—small intestine (jejunum and ileum), large intestine (cecum and colon), liver, or serum—but the difference resulting from fenugreek supplementation is indistinguishable. However, when focusing on only one sample location at a time, the influence of diet and fenugreek supplementation is apparent, which will be reported below.

### 2.2. Metabolomics Data Indicate Significant Differences as a Result of Fenugreek Supplementation

#### 2.2.1. Small Intestine

**Figure 2:**
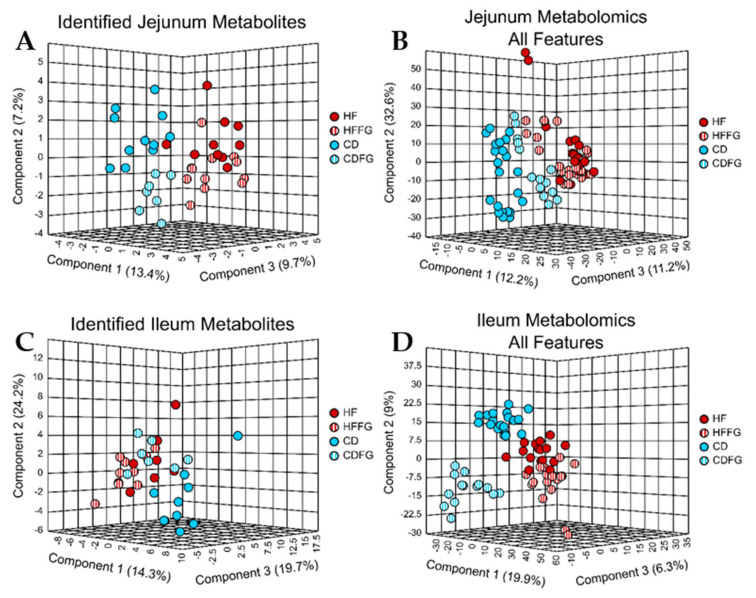
PLS-DA display minimal differences in metabolic profiles between diets and the respective FG supplemented diet in small intestines. Untargeted metabolomics was performed on the jejunum and ileum contents mice fed HF, HFFG, CD, or CDFFG diets for 14 weeks. PLS-DA were performed for (**A**) identified jejunum metabolites, (**B**) all jejunum spectral features, (**C**) identified ileum metabolites, and (**D**) all ileum spectral features. Cross validation values can be found in Supplementary Table S1. Experimental replicates are shown; HF samples are shown in red, HFFG samples are shown in red stripes, CD samples are shown in blue, and CDFG samples are shown in blue stripes.

From the metabolomics data, nearly 7000 unique spectral features were detected in the jejunum and ileum samples (6837 and 6823 for jejunum and ileum, respectively; Table 1). Of these features, 145 and 140 were identified as metabolites in the jejunum and ileum samples, respectively. While there were similar numbers of features and annotated metabolites, there were vast differences in the number of features which were determined to be significantly different (*p* < 0.05 and fold change > |1.5|) between HF- and CD-fed mice, and features were altered by both HF diet and fenugreek supplementation between the two small intestine regions. Specifically, in the jejunum contents, no annotated metabolites and only 18 unidentified features were significantly different when comparing HF vs. CD and also when comparing HF vs. HFFG (HF-altered and FG-altered; HF vs. HFFG; Table 1). Additionally, using visualization tools such as PLS-DA, minimal to no separation was observed between the groups with and without fenugreek, although there is slight separation between HF and CD diets in the identified metabolome (Figure 2A). However, there was more separation between the profiles associated with the diets and resulting from fenugreek supplementation of CD diet when comparing the unidentified and identified features (Figure 2B). In the ileum content samples, 321 spectral features were determined to be significantly different both when comparing HF to CD and when comparing HF to HFFG with 310 of these features having been decreased with fenugreek supplementation to HF diet (HF-altered and FG-altered; HF vs. HFFG; Table 1). While the PLS-DA for the annotated metabolites again only shows a little separation between the groups (Figure 2C), similar to the jejunum samples, including the unidentified features increases the distinction (Figure 2D) between all four diets.

#### 2.2.2. Large Intestine

**Figure 3:**
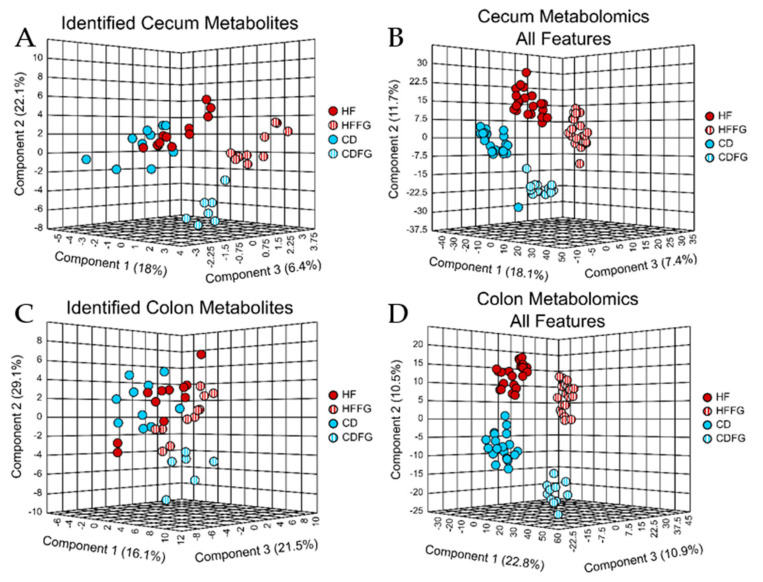
Diet and FG supplementation induce distinct differences in the large intestine metabolome as shown by PLS-DA. Untargeted metabolomics was performed on the cecum and colon contents of mice fed either HF, HFFG, CD, or CDFG diets after 14 weeks of diet exposure. PLS-DA were performed for (**A**) identified cecum metabolites, (**B**) all cecum spectral features, (**C**) identified colon metabolites, and (**D**) all colon spectral features. Cross validation values can be found in Supplementary Table S1. Experimental replicates are shown; HF samples are shown in red, HFFG samples are shown in red stripes, CD samples are shown in blue, and CDFG samples are shown in blue stripes.

**Figure 4:**
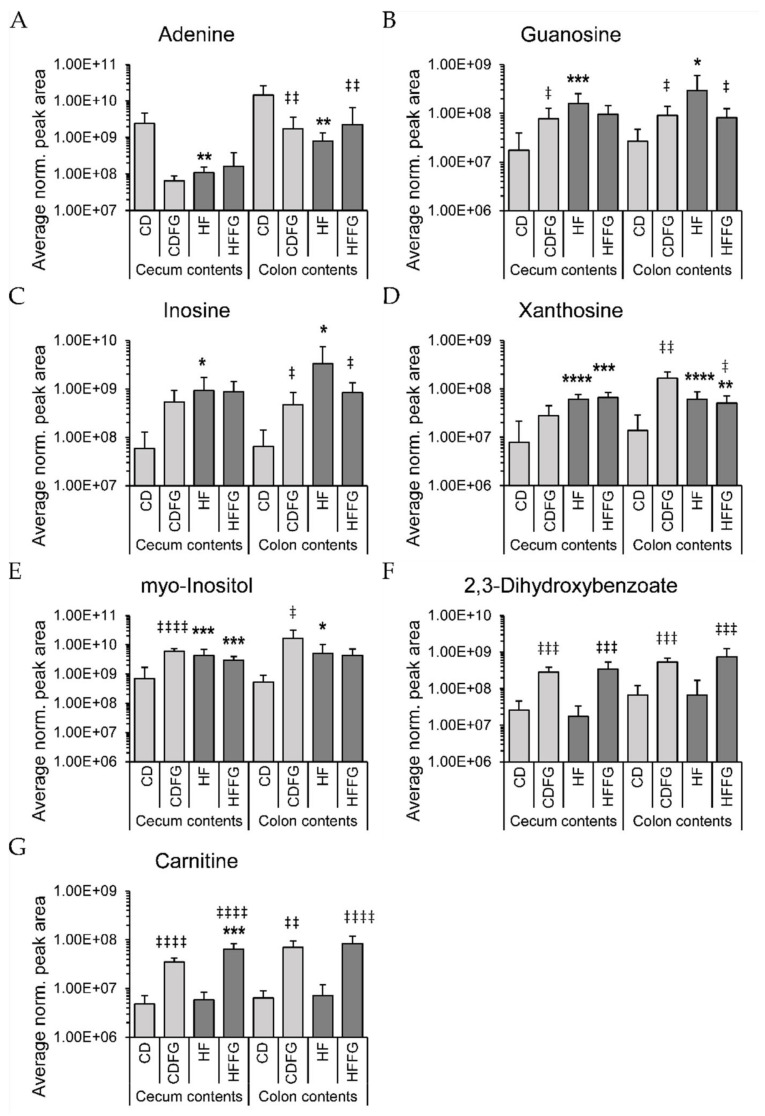
Normalized intensities of (**A**) adenine, (**B**) guanosine, (**C**) inosine, (**D**) xanthosine, (**E**) myo-inositol, (**F**) 2,3-dihydroxybenzoate, and (**G**) carnitine from both cecum and colon data. Data were normalized according to mass and the normalized peak area is represented on a log_10_ scale as mean ± standard deviation. Significance was determined using a student’s *t*-test. Significance is represented as * *p* < 0.05, ** *p* < 0.01, *** *p* < 0.001, and **** *p* < 0.0001 for comparisons against respective CD groups or ‡ *p* < 0.05, ‡‡ *p* < 0.01, ‡‡‡ *p* < 0.001, and ‡‡‡‡ *p* < 0.0001 for comparisons against either HF or CD, respectively.

Large intestine contents show dramatic differences with fenugreek supplementation in these metabolomics data. Specifically, of the 209 total metabolites annotated in these studies, 141 and 137 were detected in the cecum and colon contents, respectively (67% and 66%). Moreover, a total of 11,157 (cecum) and 8974 (colon) spectral features were detected in the large intestines contents of mice used in this study (Table 1). Among these features, 1209 (cecum) and 753 (colon) were determined to be significantly altered by HF relative to CD and also significantly increased or decreased with the addition of fenugreek to HF diet (Table 1). Using PLS-DA to visualize the small molecule profiles in the cecum and colon contents, it is apparent that the profiles of mice fed fenugreek are distinctly different from the profiles of mice not given fenugreek. This is in addition to alterations caused by either HF or CD diets for both identified and unidentified small molecules (Figure 3). Among the identified small molecules, many of these are annotated to be involved in central carbon pathways. PLS-DA plots for these annotated metabolites display no overlap between the HF and HFFG groups (Figure 3A,C). Using the variable importance in projection (VIP) score, it was determined that of the top 10 highest VIP scoring metabolites, five metabolites were conserved between cecum and colon and have a compelling impact on the separation of these groups for both regions (Figure S1). These metabolites are inosine, adenine, myo-inositol, xanthosine, and guanine. They are significantly altered by HF diet, particularly, inosine, myo-inositol, xanthosine, and guanine increase in HF- relative to CD-fed mice, but adenine decreases in HF- relative to CD-fed mice. While the abundance of these metabolites in CDFG may be altered to be more similar to HF than CD groups, these changes are not associated with a worsened metabolic state as is seen between HF and CD (Figure S6). No consistent trend of increasing or decreasing with fenugreek supplementation is evident for these metabolites (Figure 4A–E). However, a pairwise PLS-DA comparison of HF and HFFG groups, which are clearly separated, (Figure S2) found that while several metabolites contribute to the separation of these profiles, two specific metabolites, carnitine and 2,3-dihydroxybenzoate, heavily influenced the separation. As shown in Figure 4F,G, these metabolites consistently display high fold changes in CDFG and HFFG relative to CD and HF groups, respectively.

#### 2.2.3. Liver

**Figure 5:**
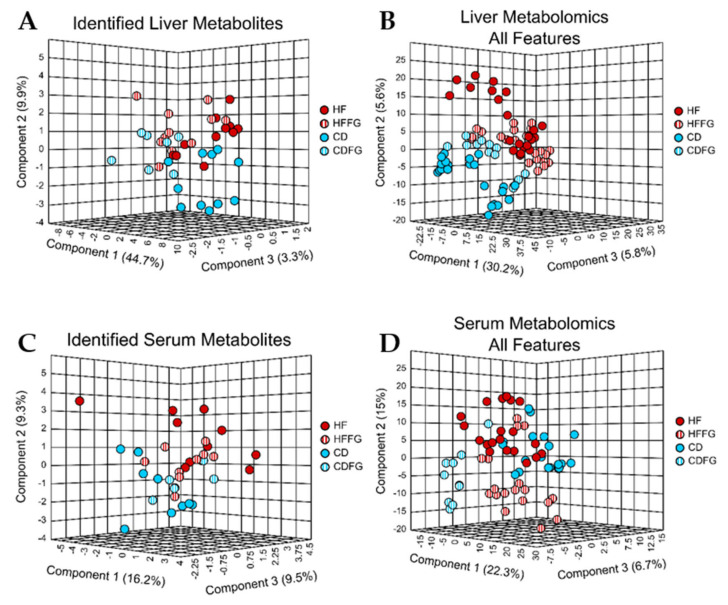
PLS-DA of liver and serum samples reveal subtle distinctions between the metabolic profiles associated with each diet, more notably in the unidentified features. Untargeted metabolomics was performed on the liver and serum samples from mice fed either HF, HFFG, CD, or CDFG diets. PLS-DA were performed for (**A**) identified liver metabolites, (**B**) all liver spectral features, (**C**) identified serum metabolites, and (**D**) all serum spectral features. Cross validation values can be found in Supplementary Table S1. Experimental replicates are shown; HF samples are shown in red, HFFG samples are shown in red stripes, CD samples are shown in blue, and CDFG samples are shown in blue stripes.

While not as dramatic as in the large intestines, metabolomics data reveal the liver metabolome is also altered by fenugreek. From the livers, a total of 3323 spectral features were detected, from which 130 features were identified as water-soluble metabolites when compared to an in-house standard library (Table 1). Of the unidentified spectral features, 88 were determined to be significantly altered in HF-fed mice relative to CD-fed mice and also significantly different with the addition of fenugreek to HF diet (Table 1). From the PLS-DA, which only includes the annotated metabolites (Figure 5A), and another that includes all features (Figure 5B), it is clear that the metabolite profiles in the livers of mice are moderately different when the diet is supplemented with fenugreek. From the multivariate analysis of the annotated metabolites within HF and HFFG groups (Figure S3), it was determined that the metabolite with the highest VIP score and thus the one most substantially driving this separation is the bile salt, glycodeoxycholate. This bile salt has a fold change (HFFG/HF) of 2.6 and *p*-value below 0.06.

#### 2.2.4. Serum

Metabolomics data display minimal differences in the serum metabolome resulting from fenugreek supplementation. A total of 1772 spectral features were detected in the serum samples, with 120 metabolites identified (Table 1). From the unidentified spectral features, only 21 features were determined to be significantly different between HF and CD and also significantly different between HF and HFFG (Table 1). Neither PLS-DA of identified (Figure 5C) or unidentified (Figure 5D) metabolites show obvious separation; however, a small degree of separation can be observed in both. In the PLS-DA of identified metabolites (Figure 5C), there is slight separation between groups as a result of the diet, with FG having little to no influence. Despite this, the PLS-DA of unidentified features (Figure 5D) indicates there is a separation, albeit small, resulting from the addition of fenugreek to the diets.

### 2.3. Metagenomic Analysis Reveal Notable Modifications in Gut Microbiome of FG Supplemented Mice

**Figure 6:**
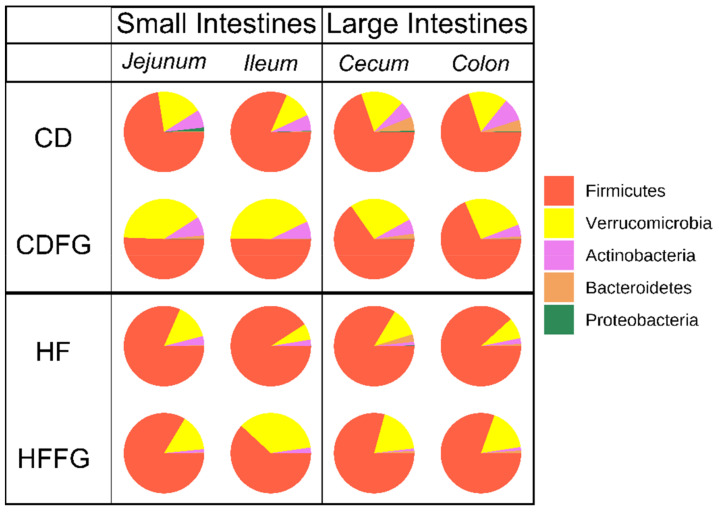
Relative abundance of operational taxonomic units (OTU) determined by 16S sequencing and grouped by phyla, showing an increase in Verrucomicrobia with the addition of FG to either CD or HF diets. The relative abundances are shown for each diet individually for each intestinal region analyzed.

Samples from the contents from jejunum, ileum, cecum, and colon intestinal regions were collected and the microbial composition was determined via 16S sequencing. Using both weighted and unweighted Unifrac phylogenetic analyses, it was revealed that, similar to the metabolomics results, the most statistically significant differences between HF- and HFFG-fed mice occurred in the large intestines (Table 2) [25]. Analysis of the abundance of operational taxonomic units (OTU) at the phylum level reveals that on average the abundance of the Actinobacteria, Bacteroidetes, and Firmicutes phyla decreases with FG supplementation, while Verrucomicrobia remains more constant. This results in a significant increase in the relative abundance or ratio of Verrucomicrobia to the sum of counts in each intestinal region with FG supplementation to CD or HF diets (Figure 6). Upon closer inspection, it was determined that only one OTU from the Verrucomicrobia phylum, found in the *Akkermansia* genus, was found in these samples. However, despite these substantial differences in the relative abundances of these phyla, the alpha diversity showed no statistical significance between dietary groups for any of the analyzed intestinal regions. Using DESeq2, of the core OTUs which were present in all dietary groups, OTUs which significantly (*p* < 0.05 adjusted with Benjamini–Hochberg correction) increased or decreased in abundance in the HF-fed mice relative to the CD-fed mice were determined [26]. Of these HFD-altered OTUs, the OTUs which were significantly altered in the opposite direction in HFFG-fed mice relative to HF-fed mice were considered to be corrected by FG. These results are summarized in Table 3. However, OTUs which were significantly altered by HF diet and corrected by FG were only found in the cecum and colon contents. Correlation analyses for these HF-altered and FG-corrected OTUs and the selected metabolites found to significantly contribute to separation between either HF and CD or HF and HFFG groups were performed (Tables S7–S10). From these correlation analyses, the OTUs and metabolites were clustered, and one OTU from the Firmicutes phylum and two from the Bacteroidetes frequently clustered with 2,3-dihydroxybenzoate and carnitine, which were found to be significantly higher in abundance with FG supplementation.

### 2.4. Fenugreek Supplementation Modulates HDL Balance and Total Cholesterol in HF Diet-Fed Mice

**Figure 7:**
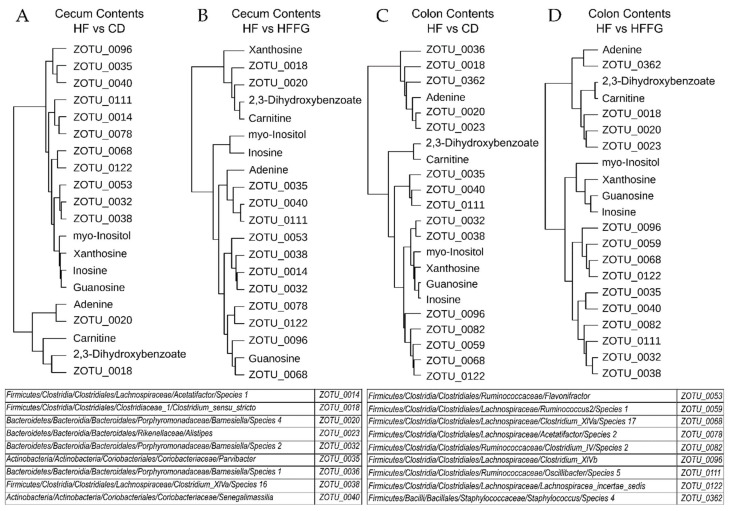
Dendrogram cluster analysis using Pearson distance measure for HF-altered and FG-corrected OTUs and metabolites significantly contributing to separation between groups in the large intestines. Dendrograms are show for (**A**) HF versus CD comparisons in the cecum contents, (**B**) HF versus HFFG comparisons in the cecum contents, (**C**) HF versus CD comparison in the colon contents, and (**D**) HF versus HFFG comparison in the colon contents.

This study expands on our previous research showing that fenugreek diet supplementation improved markers of metabolic health as well as countered dysbiotic effects in mice fed high-fat diet [14,15]. Since our previous study demonstrated an attenuated ability of fenugreek to counter HF-diet induced metabolic dysfunction when mice were supplemented for 16 weeks, we decreased the treatment time to 14 weeks for the current study. As shown in Figure 7, the expected increases in total cholesterol and HDL and LDL cholesterol in HF- versus CD-fed mice were observed. Fenugreek supplementation decreased total cholesterol levels in HF-fed mice, but this attenuation did not reach statistical significance (*p* = 0.0627). A previous study demonstrated small but significant decreases in absolute levels of LDL with FG supplementation to HF diet; however, this was not the case for the present study [14,15]. One possible explanation for the contrast is due to increased baseline LDL levels in the present study. When HDL was expressed as a percent of total cholesterol, HF diet decreased the percent HDL compared to CD diet in the absence of fenugreek but not in the presence of fenugreek (compare CDFG and HFFG). Concordant with previous results [14], fenugreek supplementation improved the HDL cholesterol composition in HF-fed mice without altering body weight or body fat (Table S2).

## 3. Discussion

Fenugreek has traditionally been used in herbal remedies to treat disorders such as hyperlipidemia and diabetes; however, the mechanisms by which these benefits arise are largely unknown. While several mechanisms have been proposed, a compelling theory is that at least some beneficial effects are a result of fenugreek altered gut microbiomes. Specifically, a previous study determined that while a HF diet has detrimental effects such as reducing alpha diversity and inducing gut dysbiosis, fenugreek was able to reverse some of the harmful effects [15]. This is important as gut dysbiosis has been seen to be enough to impair metabolic and neurologic function of the mammalian host [15,27,28,29,30,31,32]. In particular, studies have shown that fenugreek is an efficient treatment for hyperlipidemia, cholesterolemia, and hyperglycemia which maybe a result of the microbial alterations [15,33,34,35,36].

This study, however, focused on the changes in the metabolome resulting from fenugreek supplementation. As fenugreek and the microbiome both contribute to metabolic health, and the metabolome serves as a way to increase understanding of phenotypes, it follows that metabolomics is an important addition to fenugreek studies. Untargeted mass spectrometry-based metabolomics allows for the analysis of all detectable, water-soluble small molecules, rather than limiting the analysis to known central carbon metabolites, which are only a fraction of the total metabolites. This study demonstrates, using high throughput mass analysis, that fenugreek not only alters the microbiome but also significantly alters the intestinal and liver metabolomes of mice.

As anticipated, metabolomics data reveal distinctly different metabolic profiles in the small intestine, large intestines, liver, and serum which arise from the different microbial activity and physiological function of these tissues and biofluids. For the small and large intestine samples, while it is expected that the metabolome will be differentiated, it is not guaranteed that there will be clear and obvious distinctions in metabolomics since certain metabolites are common to many cells and organisms. However, substantial distinctions are present in this study, which is an indicator of high-quality data. The sum of the normalized abundance displays a higher abundance for the identified metabolites in the liver and small intestine regions than in the large intestinal regions or serum (Figure S4); however, there were thousands more unidentified spectral features in the cecum and colon as compared to other regions (Table 1). It is interesting that while the large intestines have a lower abundance of identified metabolites, there are significantly more features, which likely leads to greater diversity in the spectral features in this region.

### 3.1. High Fat Diet Influences Purine Metabolite Abundances

The alterations in the relative abundance of purine metabolites within the large intestine contents are intriguing as these compounds are the building blocks for nucleic acids and cofactors, which are important for energy metabolism. Additionally, a previous study, which investigated the impact of various diets on both male and female mice found that purine metabolites were impacted in liver tissues [23]. Purine metabolites are regulated in mammalian cells primarily through salvage pathways, but also de novo biosynthesis and purine degradation pathways [37]. However, as the metabolites leading into these pathways (ex. AICAR, IMP, and uric acid) are not significantly different between HF and CD diets in this study, alterations in mammalian purine metabolite regulation seem an unlikely explanation. Another possible reason for these alterations is different abundances of purine metabolites in the diets, but as the diets in this study were nutritionally matched, this also seems an unlikely explanation. While these metabolites are altered between HF and CD diets and an integral part of primary metabolism, we do not have evidence to suggest a causative relationship between these metabolite abundances and the negative effects of HF diet.

### 3.2. Fenugreek Induces Considerable Changes in the Large Intestines

Although fenugreek-induced alterations are less obvious in the small intestines, the large intestines display fenugreek-induced alterations which are undeniable. PLS-DA analyses of the metabolite profiles associated with the four diets for each intestinal region highlight the significant differences in the metabolome of the cecum and colon metabolites between mice fed either HF or CD diet and each supplemented with fenugreek. These two PLS-DA analyses (Figure 3) show very similar separation, and from the VIP scores (Figure S1), it was determined that of the top 10 metabolites contributing most to the separations, five metabolites are shared between cecum and colon. From these data, it can be concluded that fenugreek has a dramatic effect on the metabolome of the large intestines. In these same PLS-DA analyses of identified metabolites (Figure 3A,C), while HF and CD diet groups are separated, the greater separation is found as a result of FG supplementation. Additionally, while significant differences (fold change > |1.5|, *p* < 0.1) were apparent between HF- and CD-fed mice for all intestinal regions, only the large intestinal regions contained identified metabolites which were corrected by FG (Table S3). Metabolites labeled as corrected by FG were significantly increased in HF-fed mice relative to CD and HFFG-fed mice decreased relative to HF and vice versa. This reiterates that fenugreek has a significant influence on the metabolome of the large intestines. The metabolome is representative of and informs about the phenotype, and thus, the similarities between the metabolite profiles in the cecum and colon are suggestive of similar microbial activity and response to fenugreek in these regions.

The metabolomics data correspond with microbial data described previously, in which the fecal microbiome populations were sequenced after exposure to fenugreek [15]. In this former study, which was designed in a similar way to the present study, mice were given either an HF or CD diet each with and without fenugreek (2% *w*/*w*) for 16 weeks. This study found significant differences in the alpha diversity as well as microbiome profiles determined by principal coordinate analysis of the beta diversity [15]. However, this is in contrast to the present study in which no significant differences in the alpha diversity were determined for any of the analyzed intestinal regions. After fecal microbiome samples were sequenced from the former study, 410 OTUs were found to be common to all mice. Of these 410 OTUs, the HF diet significantly altered 147 OTUs, but 50 OTUs were restored by fenugreek supplementation. Further analyses of these OTUs found that many were from the Firmicutes phylum and were predictive of metabolic function [15]. In the present study, HF-altered OTUs were found in all four analyzed intestinal regions but only FG-corrected OTUs in the large intestinal regions (Table 3). This correlates with the metabolomics results which display differences between HF and CD groups in all regions but only display separations caused by fenugreek supplementation in the large intestinal regions. Consistent with previous results, most of these FG-corrected OTUs belong to the Firmicutes phylum (Table S4). However, from the correlational and cluster analysis, two of the three OTUs which were found to be more similar to 2,3-dihydroxybenzoate and carnitine belong to the Bacteroidetes phylum (Figure 7). More specifically, these two OTUs show a significant positive correlation with these metabolites, which increase in abundance in mice given a FG supplemented diet (Tables S7–S10). This is compelling as some studies have suggested that the abundance of Bacteroidetes decreases with obesity but increases with weight loss [15].

While metabolites cannot be traced to specific bacterial or intestinal origins, the differences observed in the metabolome often parallel changes in the microbiome. Additionally, as the microbiome is well known to alter the mammalian host function, and the most significant metabolome and microbiome differences were in the large intestine, it follows that fenugreek more significantly alters the metabolic function in the large intestines than the small intestines. However, the microbiome changes in this study are more modest in comparison to the previous study [15]. Since the same strain of mice and experimental conditions, other than experimental duration, remained constant between these two studies, a likely explanation of the milder physiological effects of fenugreek reported here is the modest microbiome changes. Nonetheless, it is significant to note that even mild microbiome changes were sufficient to induce dramatic metabolite changes.

### 3.3. Liver Metabolome Is Affected by Fenugreek

**Figure 8:**
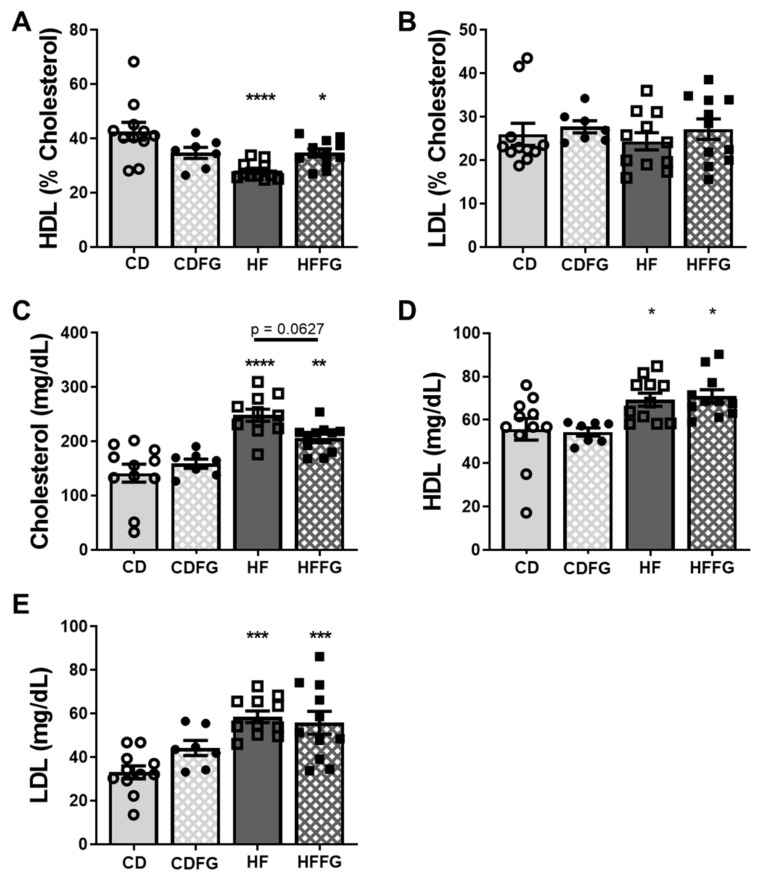
Fenugreek supplementation improves cholesterol levels and high-density lipoprotein (HDL) balance in HF diet-fed mice. Whole blood was collected from fasted mice following 14 weeks on CD, CDFG, HF, or HFFG diet, and serum was separated and analyzed for total cholesterol, HDL, and low-density lipoprotein (LDL). (**A**,**B**) HDL and LDL are represented as percent cholesterol; (**C**–**E**) show the absolute levels of total cholesterol, HDL, and LDL. Results are presented as mean ± SEM for each group—CD, HF and HFFG (*n* = 11); CDFG (*n* = 7). Statistical significance was determined using Tukey’s multiple comparison test following a one-way ANOVA. Significance is represented as * *p* < 0.05, ** *p* < 0.01, *** *p* < 0.001, and **** *p* < 0.0001 for comparisons against the CD group. No significance (*p* < 0.05) was determined between the dietary groups and the respective FG supplemented group.

While the metabolic profile changes in the livers of mice given diets supplemented with fenugreek are less dramatic than in the intestine, it is a significant observation. Despite well-regulated pathways found in the liver regardless of dietary changes, our analysis revealed distinct metabolic profiles indicating that fenugreek does alter the liver metabolome. These global metabolome changes are more likely due to relative abundance differences than different metabolites detected. In fact, the total relative abundance of all identified metabolites is lower in livers of the HFFG-fed mice than in those of the HF-fed mice (Figure S4). Of the metabolites with significant *p*-values, there are more that have decreased than increased with fenugreek (Table 1). There was a trend towards decreased total cholesterol with fenugreek supplementation of HF diet (Figure 8C). Furthermore, the high-density lipoprotein (HDL) percentage was elevated with fenugreek supplementation in HF diet (Figure 8A). These beneficial effects coincide with the observed liver metabolome changes, warranting further investigation.

When considering the effects of particular diets or supplements, it is important to assess the availability of nutrients in circulation. This study included the analysis of the metabolites and small molecules found in both the serum and the liver. It is beneficial to consider both, as the liver works to detoxify blood from the intestines before entering circulation. Nearly all detected metabolites from the liver display lower abundance in HFFG-fed mice as compared to HF-fed mice, while more serum metabolites display higher abundance with fenugreek (Figure S5). In particular, allantoate and 5-methyltetrahydrofolate are significantly lower abundance with fenugreek supplementation in the liver, but not in serum. Allantoate is involved with purine degradation which produces urea and is a plant metabolite which is involved in storage and transport of fixed nitrogen, while 5-methyltetrahydrofolate is involved in the synthesis of methionine and regulation of homocysteine [38,39,40]. A possible explanation for the decreased abundance in the liver is the increased utilization of these metabolites. However, glycodeoxycholate is a metabolite which was significantly more abundant in livers of fenugreek mice. Again, in the serum, glycodeoxycholate was not detected, but the bile salt taurodeoxycholate was detected and in a higher abundance in HF-fed compared to HFFG-fed mice (Figure S5). As these bile salts are synthesized from cholesterol, they could potentially be decreased due to the slight decrease of total cholesterol in fenugreek-supplemented mice. These results reported from liver and serum metabolomics provide insight into how the circulating metabolite levels may be altered by fenugreek.

### 3.4. Fenugreek Impacts Specific Pathways by Location

A more in depth look at the metabolomics data reported in this study reveals specific pathways and metabolites that may be more significantly contributing to the beneficial effects of fenugreek. For instance, carnitine biosynthesis and its relative abundance are significantly increased by fenugreek supplementation in the large intestines of both CD- and HF-fed mice. The differences in carnitine abundances are intriguing as this metabolite has an important role in lipid transport and fatty acid metabolism [41,42,43]. This metabolite is synthesized in the liver, kidneys, and brain, but the majority is absorbed from dietary sources such as red meat [41,43]. As carnitine is used for fatty acid oxidation and energy metabolism, the highest physiological concentrations of carnitine occur in muscular tissues, specifically cardiac and skeletal muscle [42,43]. However, like most metabolites, nearly all carnitine is absorbed through the small intestine and the remaining fraction is metabolized by bacteria in the large intestine, making it uncommon to detect carnitine in the large intestine [41]. Excess carnitine in the intestines can be problematic as gut microbiota degrade carnitine into trimethylamine (TMA) which is oxidized to trimethylamine *N*-oxide (TMAO) which promotes atherosclerosis [44]. However, due to technical limitations, TMA and TMAO were not detected in this study. Carnitine deficiency, however, can also be concerning as it has been associated with diabetes, cardiomyopathy, and obesity, as well as other conditions [43]. Further studies will be needed to focus more on this metabolite and how it specifically relates to the beneficial effects of fenugreek; however, the metagenomics results can provide additional insights to this metabolite.

While there were few global profile alterations in the metagenomics results, the increase of the Verrucomicrobia phylum in relative abundance is a significant finding. The single OTU within this phylum was found in the *Akkermansia* genus, in which there are only two identified species [45,46]. Specifically, *A. muciniphilia* was first reported in 2004 and has been the focus of several microbiome studies since that time [46,47,48]. These studies have found *A. muciniphilia* to be a commensal, mucin-degrading bacterium which has been negatively correlated with several health conditions. These conditions include obesity, diabetes, inflammation, and metabolic disorders [48,49]. *A. muciniphilia* has also been negatively correlated with serum TMAO levels, suggesting that this bacterium may be reduce or inhibit the conversion of carnitine into TMA in the intestines [50]. One study has found that oligofructose and polyphenols from black raspberries were sufficient to increase the population of *A. muciniphilia* in C57BL/6J mice [49]. From the present study, *Akkermansia* on average increased in relative abundance with the addition of FG to either diet. As FG is known to contain polyphenols, a possible explanation for this observation is that FG supplementation promoted the growth of this genus and decreased the conversion of carnitine into TMA. Future studies will be designed to investigate this hypothesis.

Besides carnitine metabolism, other pathways affected by fenugreek include cholesterol and bile acid metabolism, as well as arginine biosynthesis and the urea cycle. While these pathways have important roles in the liver, the magnitude of the impact fenugreek had on these pathways varies by sample location. Using MetaboAnalyst’s pathway analysis feature, arginine biosynthesis was determined to be the most significantly impacted pathway from these studies, but glycodeoxycholate, a bile salt, most significantly contributed to differences in the metabolic profiles of the livers of HF-fed and HFFG-fed mice [51]. In particular, this indicates that cholesterol metabolism and bile acids may be of more influence and experience more modifications in the liver, but arginine biosynthesis, while affected in all samples analyzed, shows the most differences in the cecum and colon contents. These pathways serve important functions and metabolic benefits; however, to our knowledge, there is no known connection between them. Bile acids and bile salts are primarily used to transport lipids through the intestines, and arginine assists in regulation of nitric oxide (NO) and nitrogen waste. L-Arginine has potential for use in treating several health conditions such as atherosclerosis and hypertension, cardiovascular disease, and Type 2 diabetes because of its role in the urea cycle and NO regulation [52,53]. This suggests that the beneficial effects of fenugreek may not be the result of a single altered pathway but rather a combination of pathways.

It is also significant to note the high numbers of features detected with only a fraction identified. Within this fraction, many metabolites were impacted by fenugreek; however, the profiles of the unidentified features are even more distinct than the identified metabolites. This shows how important these features are and how an HF diet and fenugreek alter the small molecules, despite the lack of knowledge of these features. When both the identified and unidentified features are considered, it is clear that the small molecule metabolome is shaped by fenugreek which implies alterations within the microbiome and thus the overall host function because of fenugreek supplementation.

## 4. Methods

### 4.1. Animals and Diets

Forty male C57BL/6J mice were purchased form Jackson Laboratories at 8 weeks of age (WOA) and fed chow diet until they were randomized at 9 WOA into four groups: (1) 60% high fat diet (HF; *n* = 11), (2) HF diet with fenugreek (HFFG; *n* = 11), (3) nutritionally matched low fat (10% kcal from fat) control diet (CD; *n* = 11), or (4) CD with fenugreek (CDFG; *n* = 7). As previously described [14,15], fenugreek-supplemented diets were prepared at Research Diets Inc. by adding 2% *w*/*w* ground fenugreek seed powder to the HF and control diets prior to pelleting. The fenugreek seeds used were fully characterized by the Rutgers University Botanical Dietary Supplements Research Center (http://www.botanical.pbrc.edu/institutions.html, accessed on 15 November 2021), the full characterization can be found in the Supplementary Materials (see Appendix A). These data include LC-MS characterization of the seeds as well as macronutrient characterization for all diets used herein. The mice were fed the indicated diets for 14 weeks prior to euthanasia and subjected to assessment of metabolic parameters including weight gain, adiposity, total cholesterol, high-density lipoprotein (HDL), and low-density lipoprotein (LDL). Body weight/adiposity data were collected regularly throughout the study. Serum, liver, and intestine sections were collected at euthanasia. The entire intestinal tract was isolated and removed carefully and separated into sections (duodenum, mid/distal jejunum, ilium, cecum, and colon) based on anatomical location. Specifically, the duodenum was identified as being 1–5 cm distal to the pylorus and proximal to the ligament of Treitz; the jejunum was identified as being 10–20 cm distal to the pylorus, and the ileum was identified as being 24–34 cm distal to the pylorus and immediately proximal to the cecum. Individual sections were irrigated intraluminally with 500 microliters of sterile PBS, and luminal contents were expressed and collected into sterile Eppendorf tubes and immediately frozen at −80 °C. Luminal intestinal contents from the jejunum, ileum, cecum, and colon were used for metabolomics analysis as well as 16S metagenomic sequencing. This study was conducted in strict accordance with the National Institutes of Health guidelines on the care and use of laboratory animals, and all experimental protocols were approved by the Institutional Animal Care and Use Committee at Pennington Biomedical Research Center. 

### 4.2. Metabolic Phenotyping

Body composition was measured using a Bruker minispec LF110 NMR analyzer (Bruker Optics, Billerica, MA, USA) as previously described [14]. All mice were fed indicated diets for 14 weeks and then euthanized by decapitation under deep isoflurane anesthesia following a 5 h fast. Fasting levels of HDL and LDL cholesterol as well as total cholesterol were measured using colorimetric assays (Wako Chemicals, Richmond, VA, USA) from serum samples collected at euthanasia.

### 4.3. Metagenomic Sequencing

Metagenomic 16S RNA sample preparation and sequencing were performed at Pennington Biomedical Research Center (PBRC) Genomics Core Facility. Using previously described methods [15], DNA was isolated from jejunum, ileum, cecum, and colon contents, and samples were sequenced on an Illumina MiSeq instrument using v3 sequencing chemistry (300 bp paired end reads). Sequence reads were processed using ‘mothur’ and followed by further identification and processing using ‘usearch’ [54,55]. Taxonomical classification of OTUs were based on the SILVA 16S rRNA sequence database v123.1 [56]. OTU relative abundances were analyzed on the phylum, class, order, family, and genus levels. Pairwise comparisons between dietary groups were carried out using the DESeq2 software package, and beta diversity was analyzed based on weighted and unweighted UniFrac distances [25,26].

### 4.4. Metabolite Extractions

Prior to extraction of water-soluble metabolites, serum samples and pulverized liver samples, jejunum contents, ileum contents, cecum contents, and colon contents were pre-weighed. The extraction procedure was adapted from Rabinowitz and Kimball [57]. All solvents used were HPLC grade. The pre-weighed samples were mixed with 1.3 mL of extraction solvent (4:4:2 acetonitrile: methanol: water with 0.1 M formic acid). The mixture was allowed to extract for 20 min at −20 °C. The mixture was centrifuged and the supernatant collected. To the remaining pellet, an additional 200 µL of extraction solvent was added and extracted for 20 min at −20 °C. This mixture was centrifuged, and supernatants from both extraction steps were combined and dried under nitrogen. The dried samples were resuspended in 300 µL of water prior to mass analysis.

### 4.5. UHPLC-HRMS

The metabolomics analysis was performed at the Biological and Small Molecule Mass Spectrometry Core at the University of Tennessee Knoxville (RRID: SCR_021368). The chromatographic and mass spectral analysis was performed according to an established method using ultra high-performance liquid chromatography coupled to high resolution mass spectrometry (UHPLC-HRMS) [58]. The resuspended metabolites were stored at 4 °C in an UltiMate 3000 RS autosampler (Dionex, Sunnyvale, CA, USA) until analysis. Samples were analyzed with duplicate injections. All solvents used were HPLC grade (Fisher Scientific, Hampton, NH, USA). Reversed phase separations were carried out using a Synergi 2.6 µm Hydro RP column (100 mm × 2.1 mm, 100 Å; Phenomenex, Torrance, CA, USA) and an UltiMate 3000 pump (Dionex). The chromatography utilized a 25 min gradient elution as described previously [58] with a water:methanol solvent system and a tributylamine ion pairing reagent. The separated metabolites were ionized via negative mode electrospray ionization prior to analysis on an Exactive Plus Orbitrap MS (Thermo Scientific, San Jose, CA, USA). The full scan analysis was performed as described previously [58].

### 4.6. Metabolomics Data Processing

Initial raw spectral files, which were generated by Thermo’s Xcalibur software, were converted to mzML format using the msConvert package from ProteoWizard [59]. After converting these files, Metabolomic Analysis and Visualization Engine (MAVEN) (Princeton University) was used for data processing [60,61]. This software was used for retention time correction and peak alignment. Metabolites were manually identified by comparing the exact mass (±5 ppm) and retention times to an in-house standard library of 279 metabolites. The unidentified spectral features were automatically selected and isotope and adduct peaks annotated using XCMS and CAMERA R packages [62,63,64]. Before further processing the adduct and isotope peaks were removed, and only features with a signal to blank ratio of 3 or greater in at least half of the biological replicates for at least one diet were used for further analyses and are reported here. Metabolomic data are available in MetaboLights data repository under the unique study ID MTBLS4376 (www.ebi.ac.uk/metabolights/MTBLS4376, accessed on 24 February 2022) [65].

### 4.7. Statistical Analysis

All spectral data were normalized according to mass and background subtracted. Heatmaps display log_2_ fold changes and *p*-values determined by a Student’s *t*-test and were prepared using R (v4.0.3) [66]. The normalized data were filtered via interquartile range (IQR), log transformed, and Pareto scaled via MetaboAnalyst 5.0 before partial least squares discriminant analysis (PLS-DA) was performed using the same software [51,67]. After PLS-DA was performed, the plots were cross validated using 10-fold cross validation via MetaboAnalyst 5.0, and Q^2^ and R^2^ scores were reported as qualitative assessment of the analysis (Table S1) [68,69]. Venn diagrams were prepared using an open source software, Venny 2.1 [70]. Bar graphs were prepared using Microsoft Excel and exported by Daniel’s XL Toolbox add-in for Excel [71]. For the unidentified spectral features, those with a fold change greater than 1.5 or less than 0.667 and *p*-value below 0.05 were used for Fiehn’s Seven Golden Rules analysis to determine potential molecular formulas [72]. Formulas were constricted to a mass accuracy of 5 ppm and to only the following elements: C, H, N, O, P, S, and Cl. Metabolic data are expressed as mean ± SEM, and GraphPad Prism v8.0 was used for statistical analyses. For comparisons between three or more groups, two-way ANOVA with Tukey’s multiple comparisons testing was performed.

## 5. Conclusions

The metabolic profile of the contents of four intestinal regions (jejunum, ileum, cecum, and colon), the liver tissues, and serum of mice were analyzed to determine the effects of fenugreek on the structure and function on the mammalian host. However, this study was limited in that only male mice were used, preventing analysis of sex-based differences in the impact of fenugreek. Future studies could benefit from including lipid analyses to evaluate the effect of fenugreek on lipid metabolism. The inclusion of lipidomics analyses would allow for direct measurements of cholesterol and circulating lipids. Nonetheless, the results from this study reveal insight into the effects of fenugreek on the gut metabolome. Specifically, the most metabolic alterations were found to occur in the large intestines, and small but significant metabolic alterations were found in the liver. Furthermore, discrete pathways were more significantly affected by fenugreek, including carnitine biosynthesis, bile acid and cholesterol metabolism, and arginine biosynthesis. In addition to the changes observed in the metabolome, fenugreek was able to significantly increase the relative abundance of beneficial commensal bacteria and thus, with time, impart beneficial effects on the host.

## Figures and Tables

**Table 1:** Summary of identified metabolites and unidentified mass spectral features from metabolomics data. Only features with *p*-values less than 0.05 and fold change greater than 1.5 or less than 0.667 were counted as significantly increased with fenugreek. Features with *p*-value less than 0.05 and fold change (HFFG/HF) less than 0.667 were counted as significantly decreased with fenugreek.

High Fat Diet Metabolomics							
		Jejunum	Ileum	Cecum	Colon	Liver	Serum
**Total identified metabolites**		**145**	**140**	**141**	**137**	**130**	**120**
HF-altered		11	2	41	30	2	7
*HF* vs. *CD*	*increased w/HF*	5	0	35	9	0	6
	*decreased w/HF*	6	2	6	21	2	1
HF-altered & FG-altered		6	4	1	6	3	0
*CD* vs. *CDFG*	*increased w/FG*	2	4	0	0	1	0
	*decreased w/FG*	4	0	0	0	2	0
*HF* vs. *HFFG*	*increased w/FG*	0	0	0	5	0	0
	*decreased w/FG*	0	0	1	1	0	0
**Total unique spectral features**		**6837**	**6823**	**11157**	**8974**	**3323**	**1772**
HF-altered		594	718	4294	2809	339	105
*HF* vs. *CD*	*increased w/HF*	114	508	3671	1977	59	94
	*decreased w/HF*	480	210	623	832	280	11
HF-altered & FG-altered		104	473	2917	1913	111	37
*CD* vs. *CDFG*	*increased w/FG*	18	0	1685	1243	0	16
	*decreased w/FG*	72	255	678	282	23	2
*HF* vs. *HFFG*	*increased w/FG*	12	11	217	238	51	8
	*decreased w/FG*	6	310	992	515	37	13

**Table 2:** Comparison of microbial communities in the intestinal regions of CD- or HF-fed mice each with and without fenugreek supplementation. Comparisons were made using both weighted and unweighted Unifrac phylogenetic analysis metrics. Significance is indicated as * *p* < 0.1, ** *p* < 0.05, *** *p* < 0.01.

		Weighted		Unweighted	
Intestinal Region	Comparison	Score	*p*-Value	Score	*p*-Value
Jejunum	CD vs. CDFG	0.632386	0.158	0.794898	0.261
	HF vs. CD	0.65695	0.121	0.849862	0.158
	HF vs. HFFG	0.70675	0.136	0.787471	0.134
Ileum	CD vs. CDFG	0.620738	0.454	0.742053	0.78
	HF vs. CD	0.654047	0.254	0.758081	0.574
	HF vs. HFFG	0.692661	0.584	0.83724	0.789
Cecum	CD vs. CDFG	0.654342	0.069 *	0.724363	0.222
	**HF vs. CD**	0.534961	0.004 ***	0.809126	0.031 **
	**HF vs. HFFG**	0.627892	0.008 ***	0.893359	0.003 ***
Colon	CD vs. CDFG	0.376439	0.54	0.673875	0.284
	**HF vs. CD**	0.589599	<0.0010 ***	0.908955	<0.0010 ***
	**HF vs. HFFG**	0.607401	<0.0010***	0.908432	0.005***

**Table 3:** Summary of OTUs from each analyzed intestinal region in this study. OTUs which were present in all dietary groups were labeled as core OTUs. HF-altered FG-corrected OTUs were determined by log_2_ fold changes as determined from pairwise comparisons using DESeq2. *p*-values were adjusted using a Benjamini–Hochberg correction, and a threshold of p_adj_ < 0.05 was used for each comparison. Specific OTUs, log_2_ fold changes, and adjusted *p*-values can be found in Supplementary Tables S4–S6.

	Jejunum	Ileum	Cecum	Colon
**Core OTUs**	**63**	**69**	**113**	**120**
**HF-altered**	**10**	**8**	**57**	**53**
*Significantly increased by HF*	2	7	50	40
*Significantly decreased by HF*	8	1	7	13
**HF-altered and FG corrected**	**0**	**0**	**13**	**15**
*Significantly increased by HF*	0	0	11	10
*Significantly decreased by HF*	0	0	2	5

**Figure A1:**
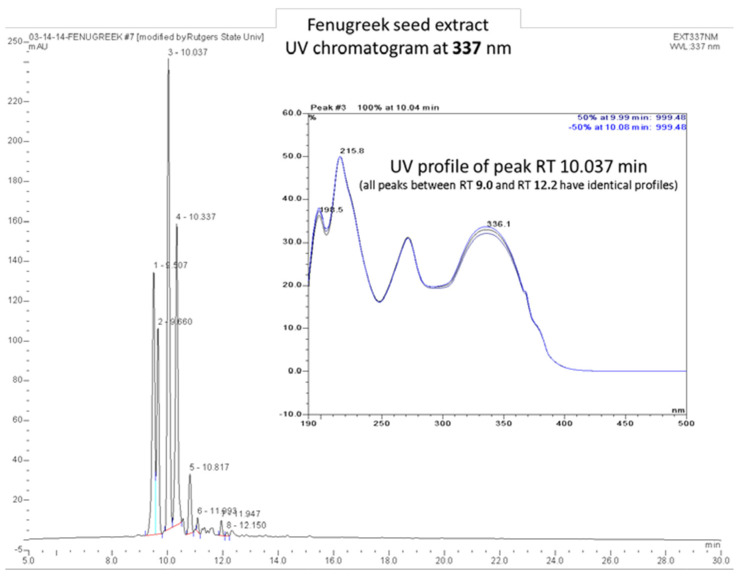
UV Chromatogram fingerprint of fenugreek seed extract at 337 nm. The fingerprint shown in Figure A1 is characterized by a series of peaks between RT 8 min and RT 12.5 min which appear to be polyphenols, mainly flavonoid di- and trisaccharides, based on their corresponding masses*, MS profiles, and UV profiles. The inserted UV spectrum is identical for all peaks suggesting similar phytochemical identity.

**Figure A2:**
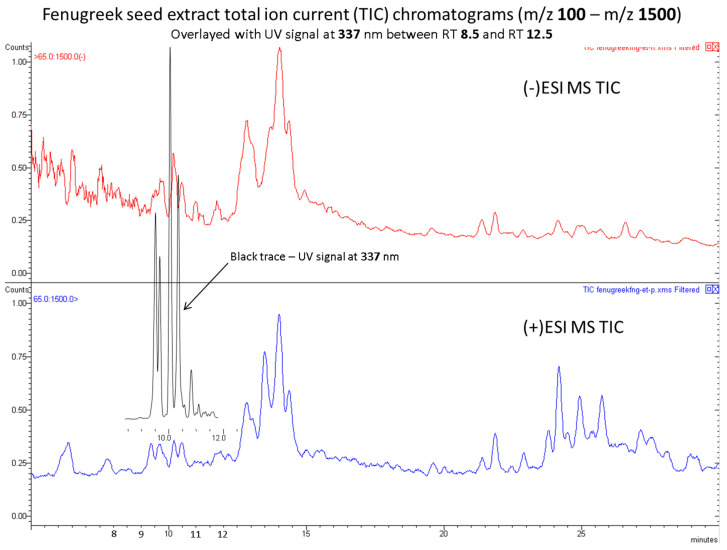
Total ion current LC/MS chromatograms (positive and negative ionization) of Ethanolic Fenugreek Seed Extract.The TIC chromatograms of the fenugreek extract are shown in Figure A2 and characterized by many peaks that were not visible in the UV chromatogram. The peaks between 12.5 min and 16 min appear to be di-through hepta-saccharides of furo-/spirostanol (trigonellosides, trigoneosides, etc.) derivatives based on their lack of absorbance signal at 254 nm, or higher, and their corresponding masses*. *Compounds’ masses and plant species cross-reference searches were made with www.reaxys.com database (Elsevier, RELX group). Samples in Figure A1 and Figure A2 were analyzed by a UPLC/MS system including the Dionex^®^ UltiMate 3000 RSLC ultra-high pressure liquid chromatography system and a Varian 1200L triple quadrupole mass detector with Varian’s MS Workstation, v. 6.41, SP2.

**Figure A3:**
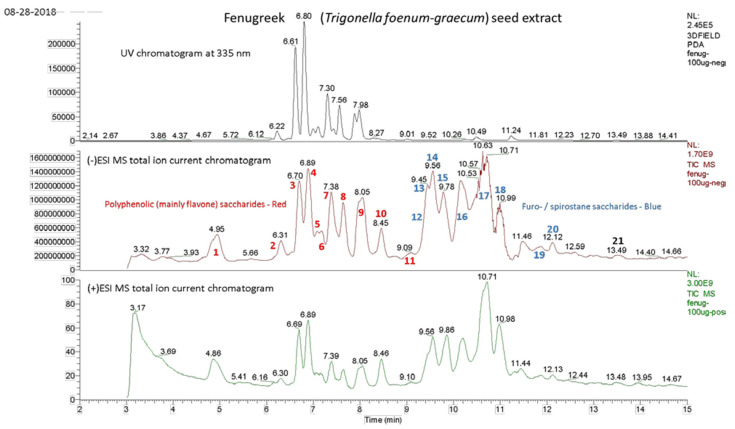
UV Chromatogram fingerprint of fenugreek seed extract at 335 nm (**top panel**) and total ion current LC/MS chromatograms (positive and negative ionization) of fenugreek seed extract (**bottom panels**) by HR-LC-MS.

## Data Availability

16S sequencing data is available in the supplementary files. Metabolome data is publicly available in MetaboLights under the study ID MTBLS4376 (www.ebi.ac.uk/metabolights/MTBLS4376).

## Supplementary Materials

The following supporting information can be downloaded at: https://www.mdpi.com/article/10.3390/ijms23073654/s1.

## Appendix A. NIH/NCCIH Product Integrity Form for Fenugreek Seed in Animal Studies

Prepared 28 August 2018 by David Ribnicky, Ph.D., Rutgers University, Botanical Dietary Supplements.

Research Center (Botanicals and Metabolic Resiliency), for NCCIH-funded studies conducted with fenugreek seeds at Pennington Biomedical Research Center, Louisiana State University, Baton Rouge.

### Appendix A.1. Identify the Source Plant for the Product Using the Scientific Taxonomic Nomenclature and Author Citation

*Trigonella foenum-graecum* L., origin India, certified organic seed sold for sprouting.

### Appendix A.2. Describe the Parts of the Plant from Which the Product Is Derived. If an Extract Is Used, Identify the Extraction Solvent

Whole Seeds.

### Appendix A.3. Name the Supplier of the Product. Include the Brand Name of the Product, If Applicable

Johnny's Selected Seeds, 955 Benton Avenue, Winslow, Maine.

### Appendix A.4. Present Data on the Characterization (e.g., Chemical Profile or Fingerprint) of the Final Product as Thoroughly as the State of the Science Allows

Whole seed is used as the raw material and no single analytical method can be used to chemically characterize an entire seed. However, an ethanolic (90%) extract was made from the seeds for the purposes of creating a reference fingerprint and which should contain many of the relevant phytochemicals (but no dietary fibers). Identification of specific compounds that are active in relevant assays can occur in conjunction with the studies of the whole seed once key metabolites relevant to biological signatures have been identified.

### Appendix A.5. Provide a Certificate of Analysis from the Supplier to Show Compliance to Their Specifications for Content and Any Other Supporting Information Relating to the Batches to Be Used in the Study. This Should Include Data on the Analysis of the Product for Contaminants/Impurities Such as Pesticides, Heavy Metals, and Residual Solvents

Whole seeds are used, so no solvents or other vehicle-based contaminants will be introduced.

Johnny's Selected Seeds is a non-GMO seed supplier. The fenugreek seed is certified organic by MOFGA Certification Services LLC under accepted USDA standards and procedures and therefore no pesticides are expected to be present. The seed lot was tested for E. coli O157H7 and Salmonella species, both of which were not detected using methods AOAC 2000.14 and AOAC 2011.03/LM respectively.

### Appendix A.6. Provide Data on Batch-To-Batch Reproducibility. These Data May Be Required Separately for the Botanical Alone, the Vehicle Alone, and the Final Product, as Appropriate

An ethanolic extract of fenugreek seeds was produced recently and evaluated in a similar manner as described in Figure A1 and Figure A2 using current high-resolution instrumentation as shown in Figure A3. Samples were separated and analyzed by a UPLC/MS system consisting of a Dionex^®^ UltiMate 3000 RSLC ultra-high pressure liquid chromatography system, and a Q Exactive Plus Orbitrap high-resolution high-mass-accuracy mass spectrometer (MS). Mass detection was full MS scan from 100 to 1500 *m*/*z*. The overall fingerprints shown in Figure A3 are similar to those shown in Figure A1 and Figure A2 suggesting that seed extracts of fenugreek are consistent and reproducible over time. The characterization of selected peaks in Figure A3 is more detailed than from Figure A2 due to the high mass accuracy of the instrument. The potential identification of the compounds comprising the numbered peaks is shown in Table A1. The molecular mass and chemical formulas used for mass determination are also shown. The number of compounds reported from *Trigonella sp.* that could have the listed mass are noted as an indicator of the number of isomers that may be possible. Further identification of specific active compounds can only be accomplished using a specific bioactivity guided fractionation, which can only be conducted once bioactivity is established by completion of the proposed studies.

**Table A1:** Putative identification of peaks corresponding to those labeled in Figure A3 designated by peak number, molecular mass, chemical formula and compound type as determined by HR-LC-MS.

Peak No.	Mass	Formula	Compound(s)	Reported from *Trigonella* (Reaxys.com)
1	Mix of 324, 444	C_21_H_32_O_10_, C_13_H_24_O_9_	Organic acid glycosides?	No; 32 structures
2	594	C_27_H_30_O_15_	Flavone (most likely Quercetin) disaccharide	Yes; 2 structures
3 and 4	564	C_26_H_28_O_14_	Flavone disaccharides?	No; >100 structures
5 and 6	448	C_21_H_20_O_11_	Flavone (Quercitrin, Luteolin, Vitexin) glycosides	Yes; 4 structures
7 and 8	534	C_25_H_26_O_13_	Possibly flavone disaccharides, Caffeoyl-syringoyl quinic acids, etc.	No; 40 structures
9	432	C_21_H_20_O_10_	Vitexin and/or Kaempferol-rhamnoside	Yes; 2 structures
10	548	C_26_H_28_O_13_	Wide variety of polyphenolic (di-)saccharides	No; 47 structures
11	504	C_24_H_30_O_11_	Megastigmane glycoside(s)?	No; 24 structures
12	904	C_44_H_72_O_19_	Furostane and/or spirostane trisaccharides	No; 5 structures
13 and 14	906	C_44_H_74_O_19_	Furostane and/or spirostane trisaccharides (Trigoneosides Ia, Ib)	Yes; 3 structures
15	920	C_45_H_76_O_19_	Furostane trisaccharides	Yes; 2 structures
16	738	C_39_H_64_O_13_	Spirostane disaccharides, saponins, etc.	No; 51 structures
17	888	C_44_H_72_O_18_	Furostane and/or spirostane trisaccharides, saponins, etc.	No; 23 structures
	890	C_44_H_74_O_18_	Furostane trisaccharides	Yes; 2 structures
	1048	C_51_H_84_O_22_	Furostane tetrasaccharides (Trigoneosides, or Trigonellosides)	Yes; 2 structures
18	902	C_45_H_74_O_18_	Furostane trisaccharides; Trigofoenoside A	Yes; 4 structures
19	1046	C_51_H_82_O_22_	Furo-/spirostane tetrasaccharides	No; 40 structures
	872	C_44_H_72_O_17_	Spirostane trisaccharides	No; 27 structures
20	888	C_45_H_76_O_17_	Cholestane trisaccharides	No; 9 structures
21	644	C_34_H_44_O_12_	Cyclopentane perhydro-phenanthrene derivatives	No; 18 structures

### Appendix A.7. Present a Plan to Monitor the Stability of Product Samples from All Batches Used during the Course of the Study

Seed lots used in mouse feeding studies will be stored under airtight, desiccated conditions at −20, and allowed to thaw slowly to 4° and then to RT before incorporation info feed, and a single seed lot will be used for the 4 year durations of the proposed studies. Based on past experience, time-based decomposition of seeds is not anticipated, but seed quality will be monitored every 6–12 months using ultra-high pressure liquid chromatography analyses on ethanolic extracts (by Dr Ribnicky as described above—see Appendix A.6). Once incorporated into feed, ethanolic extracts of food pellets will be likewise analyzed to ensure that specific seed components are retained following incorporation and sterilization. For these analyses, 6–8 of the highest represented peaks (see Table A1) will be selected for analysis as resolution of fenugreek constituents from the spectra obtained from food components could be difficult for smaller peaks. Each batch of feed will be individually tested, and variability of greater than 15% in fenugreek representation (based on peak analyses) would trigger further investigation and the likely procurement of a replacement lot of feed. Finally, seeds and samples of each lot of feed will be archived (airtight, desiccated conditions at −80) for future analyses once the biological signature of fenugreek in our animals is established.

### Appendix A.8. Describe the Method of Authentication of the Raw Material and Where and How an Authenticated Voucher Specimen of the Plant Material Is Reserved

Our seed source is a reputable commercial supplier with production and identification records. A voucher specimen of the seeds was deposited in the Chrysler Herbarium at Rutgers University. The identity of the seed, which is unique and easily identifiable, was confirmed by a Rutgers taxonomist.

### Appendix A.9. Describe the Manufacturing Process

No manufacturing is necessary for whole seeds. For incorporation into diets, whole seeds are ground into a powder with a fenugreek-dedicated laboratory blender and provided directly to Research Diets.

### Appendix A.10. If the Product Is Combined with a Diet, a Certificate of Analysis and Specifications for the Vehicle Will Also Be Necessary to Assure Purity, Consistency, Absence of Bioactive Components (e.g., Soy), and/or Reproducibility of the Vehicle. Furthermore, Analysis of the Formulated Diet May Be Required to Assess Consistency, Stability, etc. of the Product in this Matrix

The seed powder (see above) was incorporated into the diet by Research Diets at the rate (i.e., 2% *w*/*w*) specified by the investigator conducting the studies. The shelf life of the diets is 6 months as determined by Research Diets. Diet compositions from past studies using fenugreek supplementation into control and 60% high fat diets are included below (proposed studies will utilize control and Western-Style diets).

**Table A2:** D12492 and D16020408 Rodent Diets with 10 or 60 kcal% Fat and Same with Fenugreek Seeds.

	D12450J	D16020408	D12492	D16020408
Product #	10 kcal% Fat	10 kcal% Fat	60 kcal% Fat	60 kcal% Fat
		Fenugreek		Fenugreek
Ingredient		Seeds		Seeds
Casein	200	193.4	200	193.4
L-Cystine	3	3	3	3
Corn Starch	506.2	495.4		
Maltodextrin 10	125	125	125	114.2
Sucrose	68.8	68.8	68.8	68.8
Cellulose	50	48.6	50	48.6
Lard	20	18.7	245	243.7
Soybean Oil	25	25	25	25
Mineral Mix S10026	10	10	10	10
Dicalcium Phosphate	13	13	13	13
Calcium Carbonate	5.5	5.5	5.5	5.5
Potassium Citrate, 1 H_2_O	16.5	16.5	16.5	16.5
Vitamin Mix V10001	10	10	10	10
Choline Bitartrate	2	2	2	2
Fenugreek Seeds	0	21.1	0	21.1
Red Dye #40, FD&C	0	0.025	0	0.05
Blue Dye #1, FD&C	0.01	0.025	0.05	0
Yellow Dye #5, FD&C	0.04	0	0	0
Total	1055.05	1056.05	773.85	774.85
	D12492	D16020408	D12492	D16020408
gm				
Protein	179	179	179	179
Carbohydrate	710	710	203.8	203.8
Fat	47.4	47.4	272.4	272.4
Fiber	50	50	50	50
gm%				
Protein	17	16.9	23.1	23.1
Carbohydrate	0	0	26.3	26.3
Fat	4.5	4.5	35.2	35.2
Fiber	4.7	4.7	6.5	6.5
Fenugreek seeds	0	2	0	2.72
kcals				
Protein	716	716	716	716
Carbohydrate	2840	2840	815	815
Fat	427	427	2452	2452
Total	3983	3983	3983	3983
kcal%				
Protein	18	18	18	18
Carbohydrate	71	71	20	20
Fat	11	11	62	62
Total	100	100	100	100
kcal/gm	3.8	3.8	5.1	5.1

## References

[1] Caballero B. (2019). Humans against Obesity: Who Will Win?. Adv. Nutr..

[2] Rao S.V., Donahue M., Pi-Sunyer F.X., Fuster V. (2001). Obesity as a risk factor in coronary artery disease. Am. Heart J..

[3] Khaodhiar L., McCowen K.C., Blackburn G.L. (1999). Obesity and its comorbid conditions. Clin. Cornerstone.

[4] Gregor M.F., Hotamisligil G.S. (2011). Inflammatory Mechanisms in Obesity. Annu. Rev. Immunol..

[5] Simon G.E., Von Korff M., Saunders K., Miglioretti D.L., Crane P.K., van Belle G., Kessler R.C. (2006). Association Between Obesity and Psychiatric Disorders in the US Adult Population. Arch. Gen. Psychiatry.

[6] Ma J., Xiao L. (2010). Obesity and Depression in US Women: Results From the 2005–2006 National Health and Nutritional Examination Survey. Obesity.

[7] Nissen S.E., Wolski K. (2007). Effect of Rosiglitazone on the Risk of Myocardial Infarction and Death from Cardiovascular Causes. N. Engl. J. Med..

[8] Filippatos T.D., Panagiotopoulou T.V., Elisaf M.S. (2014). Adverse Effects of GLP-1 Receptor Agonists. Rev. Diabet. Stud..

[9] Piette J.D., Heisler M., Wagner T.H. (2004). Problems Paying Out-of-Pocket Medication Costs Among Older Adults with Diabetes. Diabetes Care.

[10] Payab M., Hasani-Ranjbar S., Shahbal N., Qorbani M., Aletaha A., Haghi-Aminjan H., Soltani A., Khatami F., Nikfar S., Hassani S. (2020). Effect of the herbal medicines in obesity and metabolic syndrome: A systematic review and meta-analysis of clinical trials. Phytother. Res..

[11] Fuller S., Stephens J.M. (2015). Diosgenin, 4-Hydroxyisoleucine, and Fiber from Fenugreek: Mechanisms of Actions and Potential Effects on Metabolic Syndrome. Adv. Nutr..

[12] Garg R.C., Gupta R.C. (2016). Chapter 44—Fenugreek: Multiple Health Benefits. Nutraceuticals.

[13] Nagulapalli Venkata K.C., Swaroop A., Bagchi D., Bishayee A. (2017). A small plant with big benefits: Fenugreek (Trigonella foenum-graecum Linn.) for disease prevention and health promotion. Mol. Nutr. Food Res..

[14] Knott E.J., Richard A.J., Mynatt R.L., Ribnicky D., Stephens J.M., Bruce-Keller A. (2017). Fenugreek supplementation during high-fat feeding improves specific markers of metabolic health. Sci. Rep..

[15] Bruce-Keller A.J., Richard A.J., Fernandez-Kim S.-O., Ribnicky D.M., Salbaum J.M., Newman S., Carmouche R., Stephens J.M. (2020). Fenugreek Counters the Effects of High Fat Diet on Gut Microbiota in Mice: Links to Metabolic Benefit. Sci. Rep..

[16] Zentek J., Gärtner S., Tedin L., Männer K., Mader A., Vahjen W. (2013). Fenugreek seed affects intestinal microbiota and immunological variables in piglets after weaning. Br. J. Nutr..

[17] Shtriker M.G., Hahn M., Taieb E., Nyska A., Moallem U., Tirosh O., Madar Z. (2018). Fenugreek galactomannan and citrus pectin improve several parameters associated with glucose metabolism and modulate gut microbiota in mice. Nutrition.

[18] Antharam V.C., McEwen D.C., Garrett T.J., Dossey A.T., Li E.C., Kozlov A.N., Mesbah Z., Wang G.P. (2016). An Integrated Metabolomic and Microbiome Analysis Identified Specific Gut Microbiota Associated with Fecal Cholesterol and Coprostanol in *Clostridium difficile* Infection. PLoS ONE.

[19] Cornejo-Pareja I., Muñoz-Garach A., Clemente-Postigo M., Tinahones F.J. (2019). Importance of gut microbiota in obesity. Eur. J. Clin. Nutr..

[20] Wikoff W.R., Anfora A.T., Liu J., Schultz P.G., Lesley S.A., Peters E.C., Siuzdak G. (2009). Metabolomics analysis reveals large effects of gut microflora on mammalian blood metabolites. Proc. Natl. Acad. Sci. USA.

[21] Sekirov I., Russell S.L., Antunes L.C.M., Finlay B.B. (2010). Gut Microbiota in Health and Disease. Physiol. Rev..

[22] Shen W., Gaskins H.R., McIntosh M.K. (2014). Influence of dietary fat on intestinal microbes, inflammation, barrier function and metabolic outcomes. J. Nutr. Biochem..

[23] Wells A., Barrington W.T., Dearth S., May A., Threadgill D.W., Campagna S.R., Voy B.H. (2018). Tissue Level Diet and Sex-by-Diet Interactions Reveal Unique Metabolite and Clustering Profiles Using Untargeted Liquid Chromatography–Mass Spectrometry on Adipose, Skeletal Muscle, and Liver Tissue in C57BL6/J Mice. J. Proteome Res..

[24] Pendyala S., Walker J.M., Holt P.R. (2012). A High-Fat Diet Is Associated with Endotoxemia That Originates from the Gut. Gastroenterology.

[25] Lozupone C., Hamady M., Knight R. (2006). UniFrac—An online tool for comparing microbial community diversity in a phylogenetic context. BMC Bioinform..

[26] Love M.I., Huber W., Anders S. (2014). Moderated estimation of fold change and dispersion for RNA-seq data with DESeq2. Genome Biol..

[27] Douglas-Escobar M., Elliott E., Neu J. (2013). Effect of Intestinal Microbial Ecology on the Developing Brain. JAMA Pediatr..

[28] Dinan T.G., Quigley E.M. (2011). Probiotics in the Treatment of Depression: Science or Science Fiction?. Aust. N. Z. J. Psychiatry.

[29] Bruce-Keller A.J., Salbaum J.M., Berthoud H.-R. (2018). Harnessing Gut Microbes for Mental Health: Getting from Here to There. Biol. Psychiatry.

[30] Tillisch K. (2014). The effects of gut microbiota on CNS function in humans. Gut Microbes.

[31] Neufeld K.M., Kang N., Bienenstock J., Foster J.A. (2011). Reduced anxiety-like behavior and central neurochemical change in germ-free mice. Neurogastroenterol. Motil..

[32] Heijtz R.D., Wang S., Anuar F., Qian Y., Björkholm B., Samuelsson A., Hibberd M.L., Forssberg H., Pettersson S. (2011). Normal gut microbiota modulates brain development and behavior. Proc. Natl. Acad. Sci. USA.

[33] Hannan J.M.A., Rokeya B., Faruque O., Nahar N., Mosihuzzaman M., Azad Khan A.K., Ali L. (2003). Effect of soluble dietary fibre fraction of Trigonella foenum graecum on glycemic, insulinemic, lipidemic and platelet aggregation status of Type 2 diabetic model rats. J. Ethnopharmacol..

[34] Narender T., Puri A., Shweta, Khaliq T., Saxena R., Bhatia G., Chandra R. (2006). 4-Hydroxyisoleucine an unusual amino acid as antidyslipidemic and antihyperglycemic agent. Bioorg. Med. Chem. Lett..

[35] Hamden K., Jaouadi B., Carreau S., Bejar S., Elfeki A. (2010). Inhibitory effect of fenugreek galactomannan on digestive enzymes related to diabetes, hyperlipidemia, and liver-kidney dysfunctions. Biotechnol. Bioprocess Eng..

[36] Jiang W., Gao L., Li P., Kan H., Qu J., Men L., Liu Z., Liu Z. (2017). Metabonomics study of the therapeutic mechanism of fenugreek galactomannan on diabetic hyperglycemia in rats, by ultra-performance liquid chromatography coupled with quadrupole time-of-flight mass spectrometry. J. Chromatogr. B.

[37] Pedley A.M., Benkovic S.J. (2017). A New View into the Regulation of Purine Metabolism: The Purinosome. Trends Biochem. Sci..

[38] Díaz-Leal J.L., Torralbo F., Antonio Quiles F., Pineda M., Alamillo J.M. (2014). Molecular and functional characterization of allantoate amidohydrolase from Phaseolus vulgaris. Physiol. Plant..

[39] Witte C.-P. (2011). Urea metabolism in plants. Plant Sci..

[40] Finkelstein J.D., Martin J.J., Harris B.J. (1988). Methionine metabolism in mammals. The methionine-sparing effect of cystine. J. Biol. Chem..

[41] Hoppel C. (2003). The role of carnitine in normal and altered fatty acid metabolism. Am. J. Kidney Dis..

[42] Bene J., Hadzsiev K., Melegh B. (2018). Role of carnitine and its derivatives in the development and management of type 2 diabetes. Nutr. Diabetes.

[43] Flanagan J.L., Simmons P.A., Vehige J., Willcox M.D.P., Garrett Q. (2010). Role of carnitine in disease. Nutr. Metab..

[44] Koeth R.A., Wang Z., Levison B.S., Buffa J.A., Org E., Sheehy B.T., Britt E.B., Fu X., Wu Y., Li L. (2013). Intestinal microbiota metabolism of l-carnitine, a nutrient in red meat, promotes atherosclerosis. Nat. Med..

[45] Ouwerkerk J.P., Aalvink S., Belzer C., de Vos W.M. (2016). Akkermansia glycaniphila sp. nov. an anaerobic mucin-degrading bacterium isolated from reticulated python faeces. Int. J. Syst. Evol. Microbiol..

[46] Derrien M., Vaughan E.E., Plugge C.M., de Vos W.M. (2004). *Akkermansia muciniphila* gen. nov. sp. nov. a human intestinal mucin-degrading bacterium. Int. J. Syst. Evol. Microbiol..

[47] Ouyang J., Lin J., Isnard S., Fombuena B., Peng X., Marette A., Routy B., Messaoudene M., Chen Y., Routy J.-P. (2020). The Bacterium *Akkermansia muciniphila*: A Sentinel for Gut Permeability and Its Relevance to HIV-Related Inflammation. Front. Immunol..

[48] Zhou K. (2017). Strategies to promote abundance of *Akkermansia muciniphila*, an emerging probiotics in the gut, evidence from dietary intervention studies. J. Funct. Foods.

[49] Tu P., Bian X., Chi L., Gao B., Ru H., Knobloch T.J., Weghorst C.M., Lu K. (2018). Characterization of the Functional Changes in Mouse Gut Microbiome Associated with Increased *Akkermansia muciniphila* Population Modulated by Dietary Black Raspberries. ACS Omega.

[50] Iglesias-Carres L., Hughes M.D., Steele C.N., Ponder M.A., Davy K.P., Neilson A.P. (2021). Use of dietary phytochemicals for inhibition of trimethylamine N-oxide formation. J. Nutr. Biochem..

[51] Chong J., Soufan O., Li C., Caraus I., Li S., Bourque G., Wishart D.S., Xia J. (2018). MetaboAnalyst 4.0: Towards more transparent and integrative metabolomics analysis. Nucleic Acids Res..

[52] Wu G., Bazer F.W., Davis T.A., Kim S.W., Li P., Marc Rhoads J., Carey Satterfield M., Smith S.B., Spencer T.E., Yin Y. (2009). Arginine metabolism and nutrition in growth, health and disease. Amino Acids.

[53] Allerton T.D., Proctor D.N., Stephens J.M., Dugas T.R., Spielmann G., Irving B.A. (2018). l-Citrulline Supplementation: Impact on Cardiometabolic Health. Nutrients.

[54] Kozich J.J., Westcott S.L., Baxter N.T., Highlander S.K., Schloss P.D. (2013). Development of a Dual-Index Sequencing Strategy and Curation Pipeline for Analyzing Amplicon Sequence Data on the MiSeq Illumina Sequencing Platform. Appl. Environ. Microbiol..

[55] Edgar R.C. (2013). UPARSE: Highly accurate OTU sequences from microbial amplicon reads. Nat. Methods.

[56] Quast C., Pruesse E., Yilmaz P., Gerken J., Schweer T., Yarza P., Peplies J., Glöckner F.O. (2012). The SILVA ribosomal RNA gene database project: Improved data processing and web-based tools. Nucleic Acids Res..

[57] Rabinowitz J.D., Kimball E. (2007). Acidic Acetonitrile for Cellular Metabolome Extraction from Escherichia coli. Anal. Chem..

[58] Bazurto J.V., Dearth S.P., Tague E.D., Campagna S.R., Downs D.M. (2017). Untargeted metabolomics confirms and extends the understanding of the impact of aminoimidazole carboxamide ribotide (AICAR) in the metabolic network of Salmonella enterica. Microb. Cell.

[59] Chambers M.C., Maclean B., Burke R., Amodei D., Ruderman D.L., Neumann S., Gatto L., Fischer B., Pratt B., Egertson J. (2012). A cross-platform toolkit for mass spectrometry and proteomics. Nat. Biotechnol..

[60] Clasquin M.F., Melamud E., Rabinowitz J.D. (2012). LC-MS Data Processing with MAVEN: A Metabolomic Analysis and Visualization Engine. Curr. Protoc. Bioinform..

[61] Melamud E., Vastag L., Rabinowitz J.D. (2010). Metabolomic analysis and visualization engine for LC-MS data. Anal. Chem..

[62] R Core Team (2018). R: A Language and Environment for Statistical Computing.

[63] Tautenhahn R., Patti G.J., Rinehart D., Siuzdak G. (2012). XCMS Online: A Web-Based Platform to Process Untargeted Metabolomic Data. Anal. Chem..

[64] Kuhl C., Tautenhahn R., Böttcher C., Larson T.R., Neumann S. (2012). CAMERA: An Integrated Strategy for Compound Spectra Extraction and Annotation of Liquid Chromatography/Mass Spectrometry Data Sets. Anal. Chem..

[65] Haug K., Cochrane K., Nainala V.C., Williams M., Chang J., Jayaseelan K.V., O’Donovan C. (2019). MetaboLights: A resource evolving in response to the needs of its scientific community. Nucleic Acids Res..

[66] R Core Team (2020). R: A Language and Environment for Statistical Computing.

[67] Xia J., Psychogios N., Young N., Wishart D.S. (2009). MetaboAnalyst: A web server for metabolomic data analysis and interpretation. Nucleic Acids Res..

[68] Worley B., Powers R. (2013). Multivariate Analysis in Metabolomics. Curr. Metab..

[69] Worley B., Powers R. (2016). PCA as a practical indicator of OPLS-DA model reliability. Curr. Metab..

[70] Oliveros J.C. Venny. An Interactive Tool for Comparing Lists with Venn’s Diagrams; 2007–2015. https://bioinfogp.cnb.csic.es/tools/venny/index.html.

[71] Kraus D. (2014). Consolidated data analysis and presentation using an open-source add-in for the Microsoft Excel^®^ spreadsheet software. Med. Writ..

[72] Kind T., Fiehn O. (2007). Seven Golden Rules for heuristic filtering of molecular formulas obtained by accurate mass spectrometry. BMC Bioinform..

