# Supplementary material for: Cross-Omics Analysis of Fenugreek Supplementation Reveals Beneficial Effects Are Caused by Gut Microbiome Changes Not Mammalian Host Physiology
Source: Int J Mol Sci. 2022 Mar 26;23(7):3654. doi: 10.3390/ijms23073654 (PMC8998956; doi:10.3390/ijms23073654)
Supplement: Supplementary file 1 [file ijms-23-03654-s001.zip › ijms-1541508-supplementary/ijms-1541508 Supplementary Materials_revised.pdf]

Supplemental Material for

Cross-omics Analysis of Fenugreek Supplementation Reveals Beneficial Effects are Caused by Gut Microbiome Changes Not Mammalian Host Physiology

Katarina A. Jones[1], Allison J. Richard[2], J. Michael Salbaum[2], Susan Newman[2], Richard Carmouche[2], Sara Webb[2], Annadora J. Bruce-Keller[2], Jacqueline M. Stephens[2], Shawn R. Campagna[1,3*]

[1]University of Tennessee, Department of Chemistry, Knoxville, TN, USA

[2]Pennington Biomedical Research Center, Louisiana State University System, Baton Rouge, LA, USA

[3]Biological and Small Molecule Mass Spectrometry Core, University of Tennessee, Knoxville, TN, USA

Address correspondence to Shawn Campagna, campagna@utk.edu

List of Figures

Figure S1. Top 10 metabolites with the highest VIP scores from PLS-DA of **(A)** cecum and **(B)** colon metabolomics data comparing CD, CDFG, HF and HFFG diets. VIP scores for components 1-3 are shown.

Figure S2. Pairwise PLS-DA of identified metabolites from (A) cecum and (B) colon contents of HF- and HFFG-fed mice and respective VIP score plots for (C) cecum and (D) colon. Experimental replicates are shown; HF samples are shown in red and HFFG samples are shown in green.

Figure S3. Pairwise (A) PLS-DA of identified metabolites from liver samples of HF- and HFFG-fed mice and respective (B) VIP score plot. PLS-DA were prepared using MetaboAnalyst 5.0 software. Experimental replicates are shown; HF samples are shown in red and HFFG samples are shown in green. (C) Normalized intensities of glycodeoxycholate detected in liver samples. Data was normalized according to mass and the normalized peak area is represented on a $\log_{10}$ scale as mean ± standard deviation. Significance was determined using a Student's t-test.

Figure S4. Sum of the average intensity for each metabolite identified from livers and the contents of the jejunum, ileum, cecum, and colon, and serum of mice fed HF or CD with and without fenugreek (FG; 2% w/w). The intensities were normalized by mass prior to being averaged and summed.

Figure S5. Heatmap analysis of metabolomics data. Fold changes are shown as HFFG vs HF and CDFG vs CD. Each column represents either HF or CD diets and each sample type, and are (left to right) jejunum contents, ileum contents, cecum contents, colon contents, liver, and serum, first for HF diet, followed by CD diet. The fold changes are displayed on a log 2 scale and p-values denoted by dots (* = p<0.1; ** = p<0.5; *** = p<0.01). All data was normalized by mass.

Figure S6. Insulin levels are significantly raised in HF fed mice relative to CD fed mice, but no significant changes are detected with FG supplementation. Data are represented as means ± SEM for each group – CD, HF and HFFG (n = 11); CDFG (n = 7). Statistical significance was determined using Tukey's multiple comparison test following a one-way ANOVA. Significance is represented as *p < 0.05, **p < 0.01, ***p < 0.001, and ****p < 0.0001 for comparisons against the LF group. No significance was determined with FG supplementation to either CD or HF diet.

List of Tables

Table S1. Cross validation values for PLS-DA plots using 10-fold cross validation method over 5 components. Q^2 values greater than 0.4 are considered acceptable for biological models, and low or negative values are indicative of an overfitting model.

Table S2. Fenugreek supplementation does not alter body weight, body composition or liver weight in mice fed a CD or HF diet. Group comparisons were made using a one-way ANOVA, and statistical significance was determined using Tukey's multiple comparisons test. Significance is donated by ***p < 0.001 and ****p < 0.0001 for comparisons against the CD group. No significance was determined for HF vs HFFG or CD vs CDFG comparisons, however for liver weight HF vs HFFG almost reached significance with p=0.0521.

Table S3. Metabolites which were altered by high fat diet and corrected by fenugreek. Significant differences were determined by fold change (FC >1.5 or <0.667) and p-values (p<0.1) as calculated by a Student's T-test.

Table S4. Individual OTUs altered by high fat diet but not corrected by fenugreek supplementation in small intestine contents. Taxonomy is based on SILVA 16S rRNA sequence database. Log base 2 fold changes and adjusted p-values (with Benjamini-Hochberg correction) were calculated for pairwise comparisons with DESeq. 2.

Table S5. Individual OTUs altered by high fat diet but not corrected by fenugreek supplementation in large intestine contents. Taxonomy is based on SILVA 16S rRNA sequence database. Log base 2 fold changes and adjusted p-values (with Benjamini-Hochberg correction) were calculated for pairwise comparisons with DESeq. 2.

Table S6. Individual OTUs altered by high fat diet and corrected by fenugreek supplementation. Taxonomy is based on SILVA 16S rRNA sequence database. Log base 2 fold changes and adjusted p-values (with Benjamini-Hochberg correction) were calculated for pairwise comparisons with DESeq. 2.

Table S7. Pearson correlation coefficients for HF-altered and FG-corrected OTUs and metabolites significantly contributing to separation between groups in cecum contents.

Table S8. P-values corresponding to Pearson correlation for HF-altered and FG-corrected OTUs and metabolites significantly contributing to separation between groups in cecum contents.

Table S9. Pearson correlation coefficients for HF-altered and FG-corrected OTUs and metabolites significantly contributing to separation between groups in colon contents.

Table S10. P-values corresponding to Pearson correlation for HF-altered and FG-corrected OTUs and metabolites significantly contributing to separation between groups in colon contents.

Table S11. Raw OTU counts from 16S sequencing of jejunum contents.

Table S12. Raw OTU counts from 16S sequencing of ileum contents.

Table S13. Raw OTU counts from 16S sequencing of cecum contents.

Table S14. Raw OTU counts from 16S sequencing of colon contents.

	Q^2 values	R^2 values
**All regions**	0.41125	0.45337
**Jejunum**		
Identified	-0.10162	0.47966
All features	0.55087	0.80319
**Ileum**		
Identified	-0.18882	0.45563
All features	0.77976	0.93015
**Cecum**		
Identified	0.80876	0.93406
All features	0.92423	0.97907
**Colon**		
Identified	0.78829	0.9114
All features	0.94455	0.96734
**Liver**		
Identified	-0.23075	0.55896
All features	0.71741	0.82862
**Serum**		
Identified	-0.40516	0.64936
All features	0.65483	0.82901

Table S1. Cross validation values for PLS-DA plots using 10-fold cross validation method over 5 components. Q^2 values greater than 0.4 are considered acceptable for biological models, and low or negative values are indicative of an overfitting model.

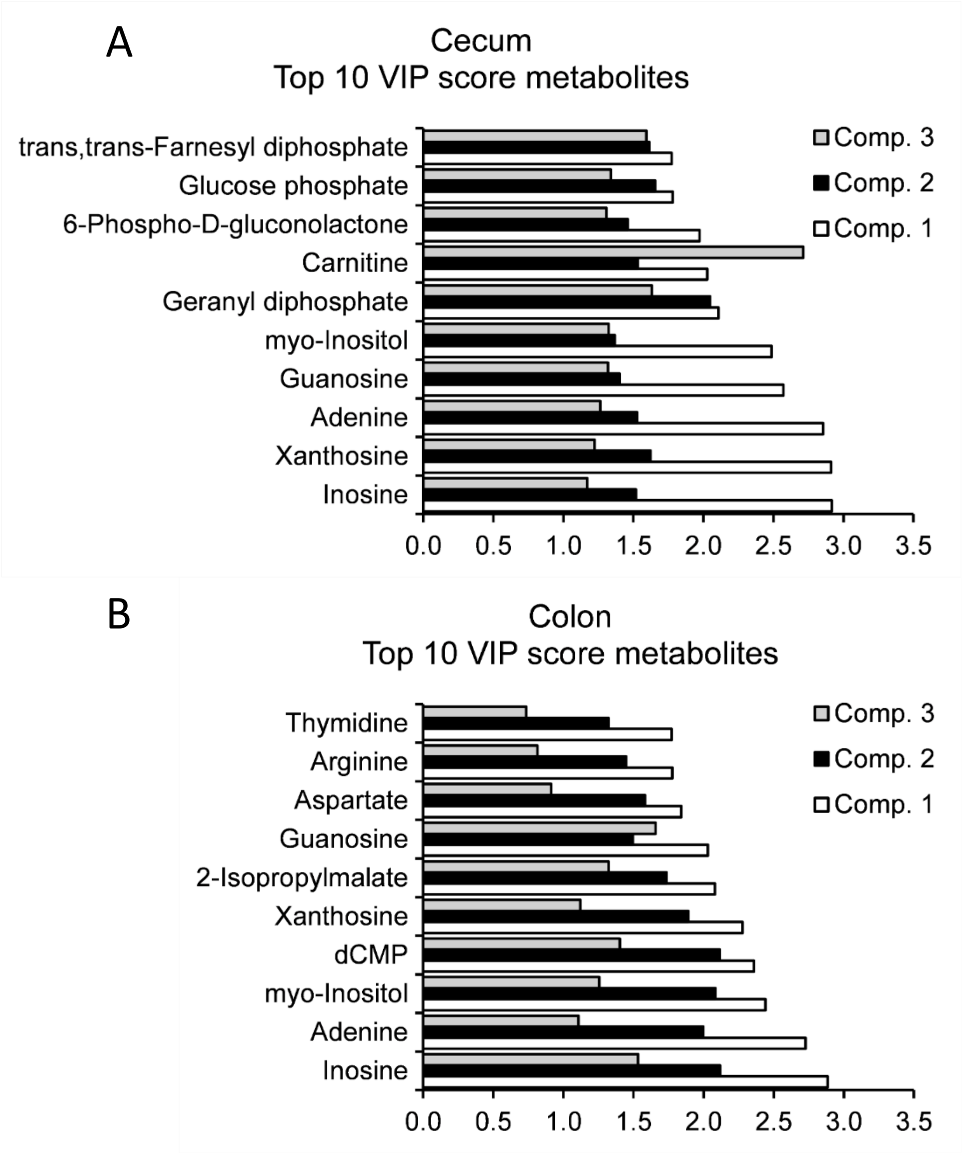

Figure S1. Top 10 metabolites with the highest VIP scores from PLS-DA of **(A)** cecum and **(B)** colon metabolomics data comparing CD, CDFG, HF and HFFG diets. VIP scores for components 1-3 are shown.

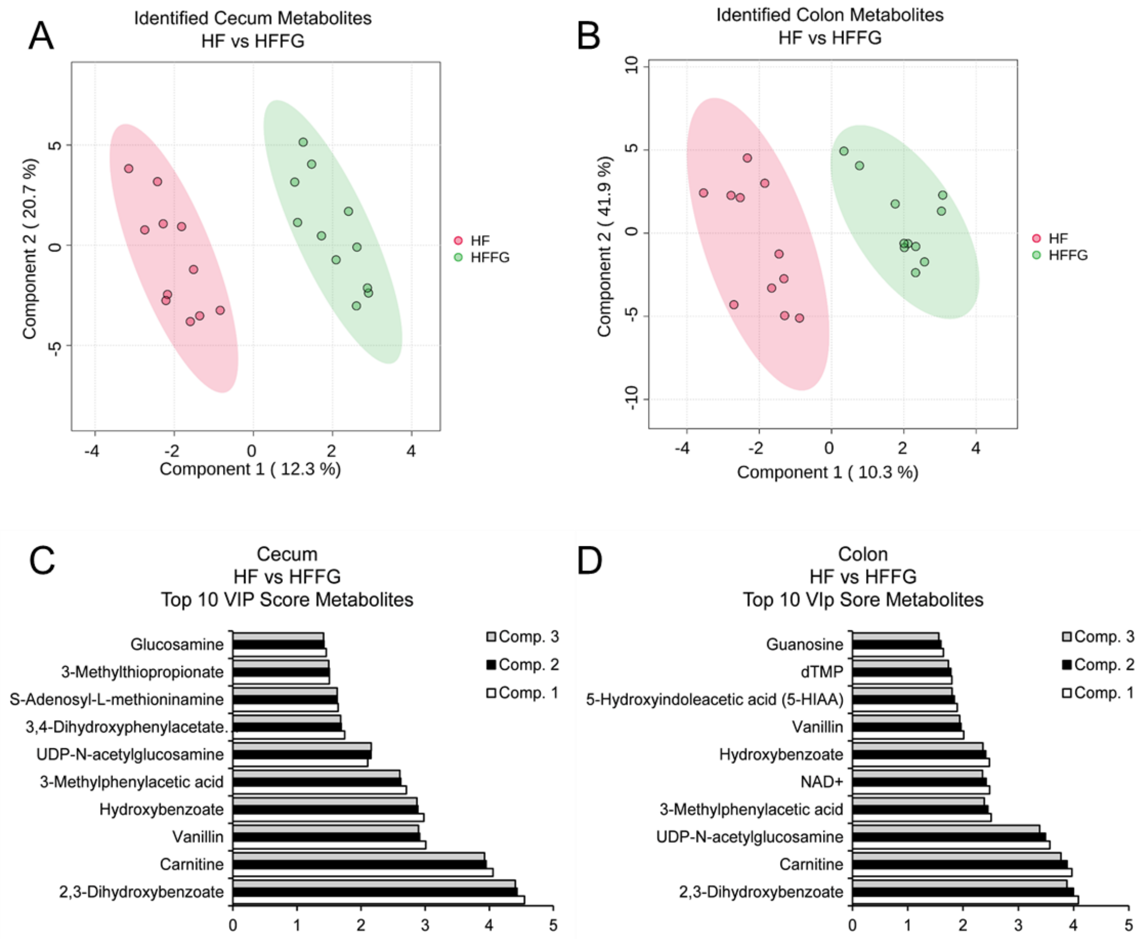

Figure S2. Pairwise PLS-DA of identified metabolites from (A) cecum and (B) colon contents of HF- and HFFG-fed mice and respective VIP score plots for (C) cecum and (D) colon. Experimental replicates are shown; HF samples are shown in red and HFFG samples are shown in green.

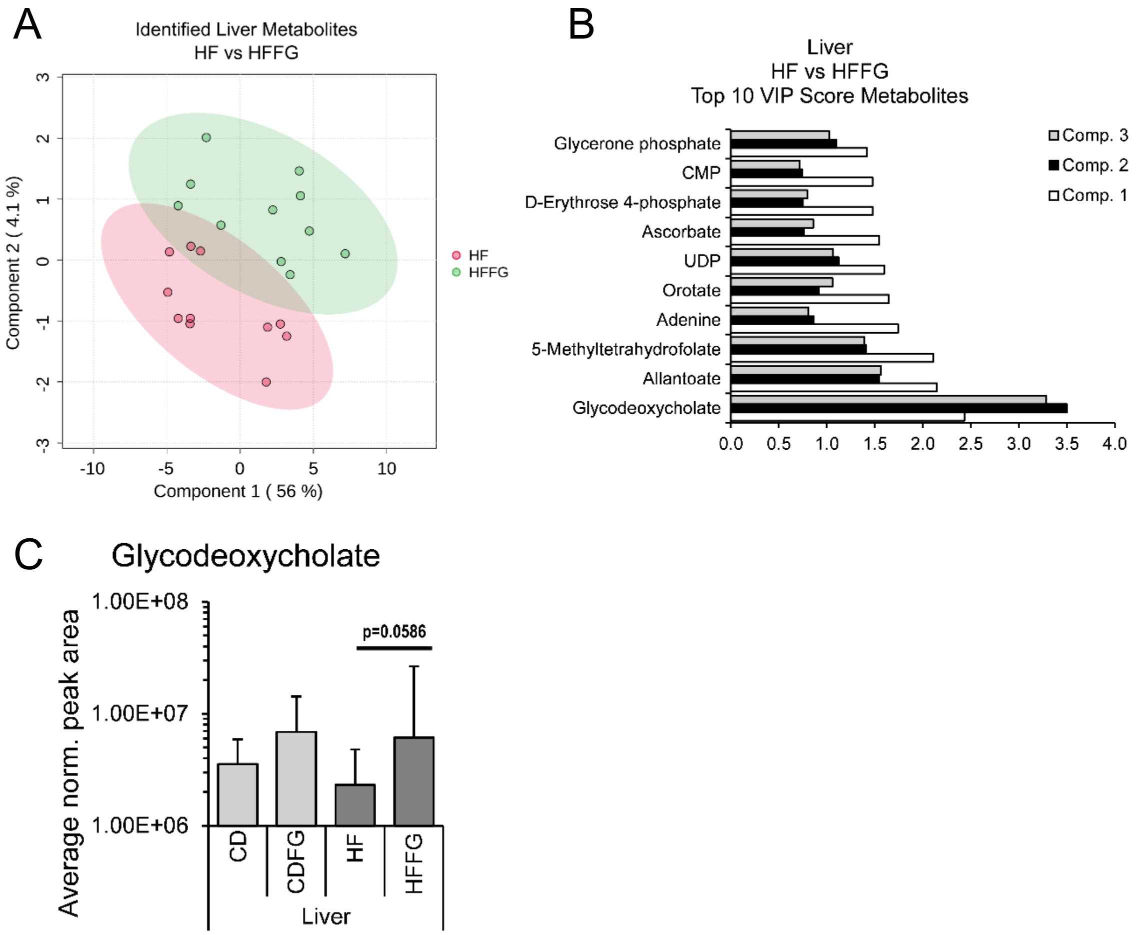

Figure S3. Pairwise (A) PLS-DA of identified metabolites from liver samples of HF- and HFFG-fed mice and respective (B) VIP score plot. PLS-DA were prepared using MetaboAnalyst 5.0 software. Experimental replicates are shown; HF samples are shown in red and HFFG samples are shown in green. (C) Normalized intensities of glycodeoxycholate detected in liver samples. Data was normalized according to mass and the normalized peak area is represented on a $\log_{10}$ scale as mean ± standard deviation. Significance was determined using a Student's t-test.

	CD (n=11)	CDFG (n=7)	HF (n=11)	HFFG (n=11)
Body Weight (g)	35.1 ± 0.9	33.0 ± 0.8	46.5 ± 1.2****	49.0 ± 0.6****
Body Fat (g)	4.7 ± 0.4	5.2 ± 0.6	13.9 ± 0.5****	14.5 ± 0.3****
Liver Weight (g)	1.21 ± 0.06	1.45 ± 0.06	1.89 ± 0.1***	2.31 ± 0.17****

Table S2. Fenugreek supplementation does not alter body weight, body composition or liver weight in mice fed a CD or HF diet. Group comparisons were made using a one-way ANOVA, and statistical significance was determined using Tukey's multiple comparisons test. Significance is donated by ***p < 0.001 and ****p < 0.0001 for comparisons against the CD group. No significance was determined for HF vs HFFG or CD vs CDFG comparisons, however for liver weight HF vs HFFG almost reached significance with p=0.0521.

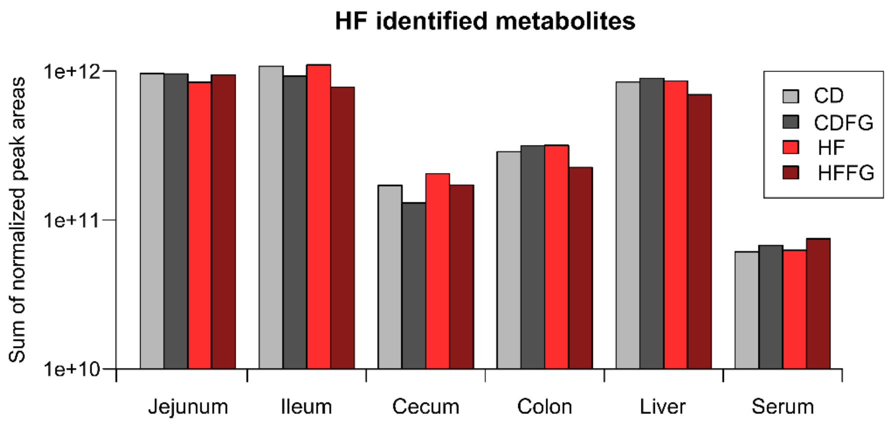

Figure S4. Sum of the average intensity for each metabolite identified from livers and the contents of the jejunum, ileum, cecum, and colon, and serum of mice fed HF or CD with and without fenugreek (FG; 2% w/w). The intensities were normalized by mass prior to being averaged and summed.

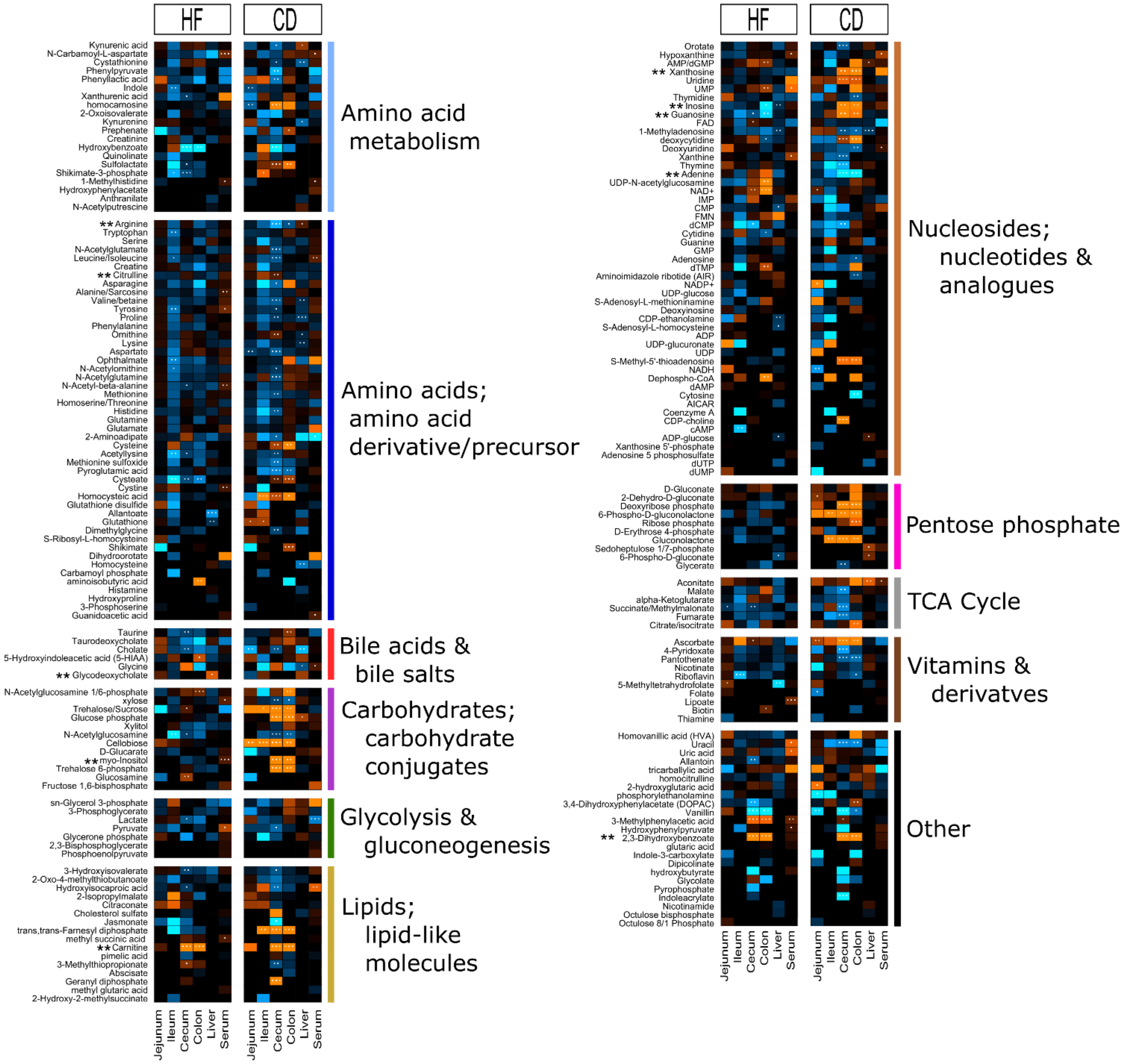

Figure S5. Heatmap analysis of metabolomics data. Fold changes are shown as HFFG vs HF and CDFG vs CD. Each column represents either HF or CD diets and each sample type, and are (left to right) jejunum contents, ileum contents, cecum contents, colon contents, liver, and serum, first for HF diet, followed by CD diet. The fold changes are displayed on a log 2 scale and p-values denoted by dots (* = p<0.1; ** = p<0.5; *** = p<0.01). All data was normalized by mass.

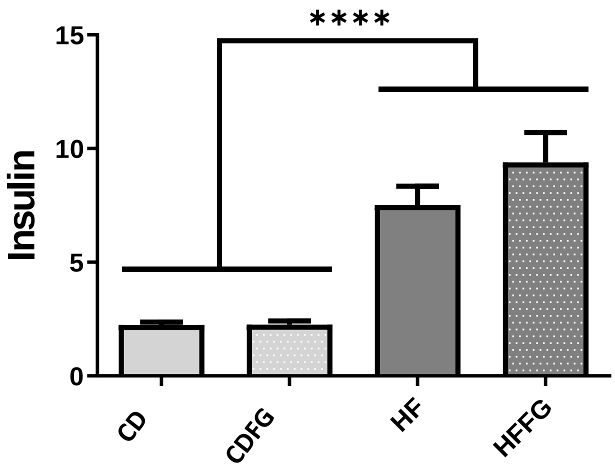

Figure S6. Insulin levels are significantly raised in HF fed mice relative to CD fed mice, but no significant changes are detected with FG supplementation. Data are represented as means ± SEM for each group – CD, HF and HFFG (n = 11); CDFG (n = 7). Statistical significance was determined using Tukey's multiple comparison test following a one-way ANOVA. Significance is represented as *$p < 0.05$, **$p < 0.01$, ***$p < 0.001$, and ****$p < 0.0001$ for comparisons against the LF group. No significance was determined with FG supplementation to either CD or HF diet.

Metabolites significantly altered by high fat diet and corrected by FG supplementation

Cecum contents					Colon contents				
Metabolite name	HF/CD		HFFG/HF		Metabolite name	HF/CD		HFFG/HF	
	fold change	p-value	fold change	p-value		fold change	p-value	fold change	p-value
Guanosine	9.202	4.65E-04	0.603	0.065	Inosine	52.163	0.021	0.249	0.064
Lactate	3.671	0.003	0.603	0.071	Guanosine	11.039	0.016	0.280	0.045
Allantoin	2.454	0.009	0.512	0.025	deoxycytidine	2.253	0.011	0.624	0.068
Acetyllysine	2.113	0.005	0.690	0.066	dTMP	0.123	0.005	2.288	0.048
N-Acetylglucosamine	1.837	0.002	0.682	0.059	UMP	0.195	0.004	2.000	0.041
N-Acetyl-beta-alanine	1.767	0.009	0.735	0.098	AMP/dGMP	0.230	0.013	1.843	0.040
Taurine	1.682	0.003	0.677	0.012	UDP-N-acetylglucosamine	0.252	0.081	5.207	0.019
Xanthurenic acid	1.605	0.069	0.687	0.076	NAD+	0.265	0.009	4.223	0.007
3-Methylthiopropionate	0.531	0.038	1.778	0.058	Biotin	0.439	0.001	1.517	0.085
Glucosamine	0.575	0.073	1.969	0.023	N-Acetylglucosamine 1/6-phosphate	0.470	0.049	1.592	0.008

Table S3. Metabolites which were altered by high fat diet and corrected by fenugreek. Significant differences were determined by fold change (FC >1.5 or <0.667) and p-values (p<0.1) as calculated by a Student's T-test.

Individual OTUs significantly altered by high fat diet but not corrected by FG supplementation in small intestinal contents

Jejunum contents			Ileum contents		
	HF/CD			HF/CD	
OTU	log_2 fold change	adjusted p-value	OTU	log_2 fold change	adjusted p-value
Firmicutes/Bacilli/Bacillales /Staphylococcaceae/Staphylococcus/Species 1	2.84	2.93E-02	Firmicutes/Clostridia/Clostridiales /Peptostreptococcaceae/Romboutsia/Species 1	2.77	3.08E-02
Firmicutes/Bacilli/Bacillales /Staphylococcaceae/Staphylococcus/Species 2	3.63	4.96E-03	Firmicutes/Bacilli/Lactobacillales /Streptococcaceae/Lactococcus	2.91	7.78E-03
Firmicutes/Erysipelotrichia/Erysipelotrichales /Erysipelotrichaceae/Allobaculum	-2.39	4.94E-02	Firmicutes/Clostridia/Clostridiales /Lachnospiraceae/Acetatifactor/Species 1	4.08	1.46E-03
Verrucomicrobia/Verrucomicrobiae/Verrucomicrobiales /Verrucomicrobiaceae/Akkermansia	-2.01	3.52E-02	Firmicutes/Clostridia/Clostridiales /Peptostreptococcaceae/Romboutsia/Species 2	3.70	3.92E-03
Firmicutes/Erysipelotrichia/Erysipelotrichales /Erysipelotrichaceae/Catenisphaera	-2.88	4.57E-03	Bacteroidetes/Bacteroidia/Bacteroidales /Porphyromonadaceae/Barnesiella/Species 2	5.46	1.92E-03
Actinobacteria/Actinobacteria/Bifidobacteriales /Bifidobacteriaceae/Bifidobacterium	-2.64	8.34E-03	Firmicutes/Clostridia/Clostridiales /Lachnospiraceae/Clostridium_XlVa/Species 2	3.39	2.70E-02
Proteobacteria/Betaproteobacteria/Burkholderiales /Sutterellaceae/Parasutterella	-5.95	2.16E-08	Firmicutes/Clostridia/Clostridiales /Lachnospiraceae/Acetatifactor/Species 2	3.95	3.92E-03
Actinobacteria/Actinobacteria/Coriobacteriales /Coriobacteriaceae/Parvibacter	-2.72	2.93E-02	Firmicutes/Bacilli/Bacillales /Staphylococcaceae/Staphylococcus/Species 3	-2.58	3.08E-02
Bacteroidetes/Bacteroidia/Bacteroidales /Porphyromonadaceae/Barnesiella/Species 1	-3.62	4.96E-03			
Firmicutes/Clostridia/Clostridiales /Lachnospiraceae/Clostridium_XlVa/Species 1	-2.82	3.42E-02			

Table S4. Individual OTUs altered by high fat diet but not corrected by fenugreek supplementation in small intestine contents. Taxonomy is based on SILVA 16S rRNA sequence database. Log base 2 fold changes and adjusted p-values (with Benjamini-Hochberg correction) were calculated for pairwise comparisons with DESeq. 2.

Individual OTUs significantly altered by high fat diet but not corrected by FG supplementation in large intestinal contents

Cecum contents OTU	Cecum HF/CD log$_2$ fold change	Cecum HF/CD adjusted p-value	Colon contents OTU	Colon HF/CD log$_2$ fold change	Colon HF/CD adjusted p-value
Firmicutes/Bacilli/Lactobacillales /Enterococcaceae/Enterococcus/Species 1	1.40	5.60E-03	Firmicutes/Bacilli/Lactobacillales /Enterococcaceae/Enterococcus/Species 1	1.09	2.10E-02
Firmicutes/Bacilli/Lactobacillales /Lactobacillaceae/Lactobacillus	2.92	7.80E-04	Firmicutes/Bacilli/Lactobacillales /Lactobacillaceae/Lactobacillus	2.86	2.11E-03
Firmicutes/Clostridia/Clostridiales /Peptostreptococcaceae/Romboutsia/Species 1	2.32	2.20E-03	Firmicutes/Clostridia/Clostridiales /Peptostreptococcaceae/Romboutsia/Species 1	2.64	3.34E-04
Firmicutes/Bacilli/Lactobacillales /Streptococcaceae/Lactococcus	1.29	4.46E-02	Firmicutes/Clostridia/Clostridiales /Lachnospiraceae/Clostridium_XlVa/Species 3	4.94	4.30E-06
Firmicutes/Clostridia/Clostridiales /Lachnospiraceae/Clostridium_XlVa/Species 3	4.75	3.81E-05	Firmicutes/Clostridia/Clostridiales /Lachnospiraceae/Acetatifactor/Species 1	2.23	1.34E-03
Actinobacteria/Actinobacteria/Coriobacteriales /Coriobacteriaceae/Enterorhabdus	1.51	7.36E-03	Firmicutes/Clostridia/Clostridiales /Lachnospiraceae/Clostridium_XlVa/Species 4	2.53	1.26E-02
Firmicutes/Clostridia/Clostridiales /Lachnospiraceae/Clostridium_XlVa/Species 4	2.74	4.25E-03	Firmicutes/Clostridia/Clostridiales /Peptostreptococcaceae/Romboutsia/Species 2	3.12	6.29E-05
Firmicutes/Clostridia/Clostridiales /Peptostreptococcaceae/Romboutsia/Species 2	2.83	5.03E-04	Firmicutes/Clostridia/Clostridiales /Lachnospiraceae/Clostridium_XlVa/Species 5	3.16	1.15E-04
Firmicutes/Clostridia/Clostridiales /Lachnospiraceae/Clostridium_XlVa/Species 5	3.73	2.29E-05	Firmicutes/Clostridia/Clostridiales /Ruminococcaceae/Pseudoflavonifractor	3.28	1.11E-03
Firmicutes/Clostridia/Clostridiales /Lachnospiraceae/Clostridium_XlVa/Species 6	2.41	5.03E-04	Firmicutes/Clostridia/Clostridiales /Lachnospiraceae/Clostridium_XlVa/Species 6	2.36	7.15E-05
Firmicutes/Clostridia/Clostridiales /Lachnospiraceae/Blautia/Species 1	2.34	5.03E-04	Firmicutes/Clostridia/Clostridiales /Lachnospiraceae/Blautia/Species 1	1.50	1.41E-02
Firmicutes/Clostridia/Clostridiales /Ruminococcaceae/Oscillibacter/Species 1	3.84	1.24E-06	Firmicutes/Clostridia/Clostridiales /Ruminococcaceae/Oscillibacter/Species 1	2.52	2.58E-04
Firmicutes/Clostridia/Clostridiales /Ruminococcaceae/Oscillibacter/Species 2	2.03	1.18E-02	Firmicutes/Clostridia/Clostridiales /Lachnospiraceae/Clostridium_XlVa/Species 13	1.02	4.46E-02
Firmicutes/Clostridia/Clostridiales /Lachnospiraceae/Ruminococcus/Species 1	1.87	3.30E-02	Firmicutes/Clostridia/Clostridiales /Lachnospiraceae/Acetatifactor/Species 2	1.93	2.07E-02
Firmicutes/Clostridia/Clostridiales /Lachnospiraceae/Blautia/Species 2	3.23	2.81E-03	Firmicutes/Clostridia/Clostridiales /Ruminococcaceae/Oscillibacter/Species 2	1.87	2.39E-03
Firmicutes/Clostridia/Clostridiales /Lachnospiraceae/Acetatifactor/Species 3	1.92	3.86E-02	Firmicutes/Clostridia/Clostridiales /Lachnospiraceae/Acetatifactor/Species 4	2.99	2.37E-04
Firmicutes/Clostridia/Clostridiales /Lachnospiraceae/Acetatifactor/Species 4	2.05	1.19E-02	Firmicutes/Clostridia/Clostridiales /Ruminococcaceae/Ruminococcus/Species 2	1.99	2.39E-03
Firmicutes/Clostridia/Clostridiales /Ruminococcaceae/Ruminococcus/Species 2	2.96	5.04E-04	Firmicutes/Clostridia/Clostridiales /Ruminococcaceae/Oscillibacter/Species 3	2.89	2.05E-03
Firmicutes/Clostridia/Clostridiales /Ruminococcaceae/Clostridium_IV/Species 1	2.74	1.51E-03	Firmicutes/Clostridia/Clostridiales /Ruminococcaceae/Clostridium_IV/Species 3	2.95	3.52E-04

Table S5. Individual OTUs altered by high fat diet but not corrected by fenugreek supplementation in large intestine contents. Taxonomy is based on SILVA 16S rRNA sequence database. Log base 2 fold changes and adjusted p-values (with Benjamini-Hochberg correction) were calculated for pairwise comparisons with DESeq. 2.

Individual OTUs significantly altered by high fat diet but not corrected by FG supplementation in large intestinal contents

Cecum contents			Colon contents		
	HF/CD			HF/CD	
OTU	log_2 fold change	adjusted p-value	OTU	log_2 fold change	adjusted p-value
Firmicutes/Clostridia/Clostridiales /Ruminococcaceae/Clostridium_III	1.49	1.29E-02	Firmicutes/Clostridia/Clostridiales /Lachnospiraceae/Blautia/Species 3	1.77	2.11E-03
Firmicutes/Clostridia/Clostridiales /Lachnospiraceae/Murimonas	2.92	2.20E-03	Firmicutes/Clostridia/Clostridiales /Lachnospiraceae/Clostridium_XlVa/Species 8	1.56	2.67E-02
Firmicutes/Clostridia/Clostridiales /Ruminococcaceae/Oscillibacter/Species 3	3.88	2.64E-05	Firmicutes/Clostridia/Clostridiales /Ruminococcaceae/Clostridium_IV/Species 4	2.10	9.68E-03
Firmicutes/Clostridia/Clostridiales /Ruminococcaceae/Clostridium_IV/Species 2	2.57	6.19E-04	Firmicutes/Clostridia/Clostridiales /Lachnospiraceae/Clostridium_XlVa/Species 9	1.87	4.80E-02
Firmicutes/Clostridia/Clostridiales /Ruminococcaceae/Clostridium_IV/Species 3	2.64	7.85E-04	Firmicutes/Clostridia/Clostridiales /Lachnospiraceae/Clostridium_XlVa/Species 10	1.98	8.20E-03
Firmicutes/Clostridia/Clostridiales /Lachnospiraceae/Blautia/Species 3	2.55	2.81E-03	Firmicutes/Clostridia/Clostridiales /Lachnospiraceae/Clostridium_XlVa/Species 12	2.95	6.20E-03
Firmicutes/Clostridia/Clostridiales /Lachnospiraceae/Clostridium_XlVa/Species 7	2.04	3.18E-03	Firmicutes/Clostridia/Clostridiales /Lachnospiraceae/Clostridium_XlVa/Species 14	1.81	4.46E-02
Firmicutes/Clostridia/Clostridiales /Ruminococcaceae/Saccharofermentans	1.81	4.72E-02	Firmicutes/Erysipelotrichia/Erysipelotrichales /Erysipelotrichaceae/Erysipelotrichaceae_incertae_sedis	3.50	1.15E-04
Firmicutes/Clostridia/Clostridiales /Lachnospiraceae/Clostridium_XlVa/Species 8	1.69	4.95E-02	Firmicutes/Clostridia/Clostridiales /Ruminococcaceae/Clostridium_IV/Species 5	2.53	2.55E-03
Firmicutes/Clostridia/Clostridiales /Lachnospiraceae/Roseburia	2.80	1.55E-03	Firmicutes/Clostridia/Clostridiales /Ruminococcaceae/Oscillibacter/Species 4	2.86	1.10E-03
Firmicutes/Clostridia/Clostridiales /Ruminococcaceae/Clostridium_IV/Species 4	2.18	7.36E-03	Firmicutes/Bacilli/Lactobacillales /Enterococcaceae/Enterococcus/Species 2	2.44	1.34E-02
Firmicutes/Clostridia/Clostridiales /Ruminococcaceae/Flavonifractor	4.05	5.04E-04	Actinobacteria/Actinobacteria/Bifidobacteriales /Bifidobacteriaceae/Bifidobacterium	-4.82	8.52E-12
Firmicutes/Clostridia/Clostridiales /Clostridiaceae_2/Alkaliphilus	3.52	7.36E-03	Bacteroidetes/Bacteroidia/Bacteroidales /Bacteroidaceae/Bacteroides	-3.82	3.42E-06
Firmicutes/Clostridia/Clostridiales /Lachnospiraceae/Clostridium_XlVa/Species 9	2.09	3.54E-03	Proteobacteria/Betaproteobacteria/Burkholderiales /Sutterellaceae/Parasutterella	-2.53	1.62E-05
Firmicutes/Clostridia/Clostridiales /Lachnospiraceae/Clostridium_XlVa/Species 10	3.34	2.81E-03	Firmicutes/Clostridia/Clostridiales /Lachnospiraceae/Clostridium_XlVa/Species 15	-2.86	2.67E-02
Firmicutes/Clostridia/Clostridiales /Lachnospiraceae/Clostridium_XlVa/Species 11	3.61	2.29E-05	Firmicutes/Clostridia/Clostridiales /Ruminococcaceae/Sporobacter	-2.52	2.58E-02
Firmicutes/Clostridia/Clostridiales /Lachnospiraceae/Clostridium_XlVa/Species 12	3.51	5.03E-04	Bacteroidetes/Bacteroidia/Bacteroidales /Porphyromonadaceae/Barnesiella/Species 3	-2.04	9.27E-03
Firmicutes/Erysipelotrichia/Erysipelotrichales /Erysipelotrichaceae/Erysipelotrichaceae_incertae_sedis	2.45	1.22E-02	Firmicutes/Clostridia/Clostridiales /Clostridiaceae_4/Geosporobacter	-1.88	3.28E-02
Firmicutes/Clostridia/Clostridiales /Ruminococcaceae/Clostridium_IV/Species 5	2.94	1.11E-03	Firmicutes/Erysipelotrichia/Erysipelotrichales /Erysipelotrichaceae/Clostridium_XVIII	-3.01	6.53E-04

Table S5 continued.

Individual OTUs significantly altered by high fat diet but not corrected by FG supplementation in large intestinal contents

Cecum contents			Colon contents		
OTU	HF/CD		OTU	HF/CD	
	log_2 fold change	adjusted p-value		log_2 fold change	adjusted p-value
Firmicutes/Bacilli/Lactobacillales /Enterococcaceae/Enterococcus/Species 2	2.34	2.92E-02			
Firmicutes/Erysipelotrichia/Erysipelotrichales /Erysipelotrichaceae/Allobaculum	-0.98	4.37E-02			
Actinobacteria/Actinobacteria/Bifidobacteriales /Bifidobacteriaceae/Bifidobacterium	-4.12	5.30E-09			
Bacteroidetes/Bacteroidia/Bacteroidales /Bacteroidaceae/Bacteroides	-1.89	3.26E-03			
Bacteroidetes/Bacteroidia/Bacteroidales /Rikenellaceae/Alistipes	-3.33	1.24E-06			
Bacteroidetes/Bacteroidia/Bacteroidales /Porphyromonadaceae/Barnesiella/Species 1	-1.59	2.69E-02			

Table S5 continued.

Individual OTUs significantly altered by high fat diet and corrected by FG supplementation

Cecum contents					Colon contents				
OTU	HF/CD		HFFG/HF		OTU	HF/CD		HFFG/HF	
	log_2 fold change	adjusted p-value	log_2 fold change	adjusted p-value		log_2 fold change	adjusted p-value	log_2 fold change	adjusted p-value
Firmicutes/Clostridia/Clostridiales /Lachnospiraceae/Acetatifactor	2.56	2.50E-03	-2.46	6.79E-06	Bacteroidetes/Bacteroidia/Bacteroidales /Porphyromonadaceae/Barnesiella	7.53	7.31E-33	-7.69	6.02E-24
Bacteroidetes/Bacteroidia/Bacteroidales /Porphyromonadaceae/Barnesiella	8.24	1.64E-30	-7.20	1.48E-21	Actinobacteria/Actinobacteria/Coriobacteriales /Coriobacteriaceae/Parvibacter	1.30	6.20E-03	-2.34	8.66E-03
Actinobacteria/Actinobacteria/Coriobacteriales /Coriobacteriaceae/Parvibacter	2.17	6.19E-04	-1.99	1.40E-03	Firmicutes/Clostridia/Clostridiales /Lachnospiraceae/Clostridium_XlVa	3.75	3.62E-12	-3.33	3.03E-09
Firmicutes/Clostridia/Clostridiales /Lachnospiraceae/Clostridium_XlVa	4.17	8.93E-10	-2.09	4.58E-07	Actinobacteria/Actinobacteria/Coriobacteriales /Coriobacteriaceae/Senegalimassilia	1.38	2.67E-02	-2.46	1.89E-07
Actinobacteria/Actinobacteria/Coriobacteriales /Coriobacteriaceae/Senegalimassilia	3.02	1.83E-04	-2.38	1.71E-05	Firmicutes/Clostridia/Clostridiales /Lachnospiraceae/Ruminococcus2	2.53	4.87E-03	-1.97	3.24E-03
Firmicutes/Clostridia/Clostridiales /Ruminococcaceae/Flavonifractor	2.79	1.11E-03	-1.36	1.43E-02	Firmicutes/Clostridia/Clostridiales /Lachnospiraceae/Clostridium_XlVa	3.44	2.11E-03	-3.91	5.63E-03
Firmicutes/Clostridia/Clostridiales /Lachnospiraceae/Clostridium_XlVa	4.07	1.98E-04	-3.15	6.30E-03	Firmicutes/Clostridia/Clostridiales /Ruminococcaceae/Clostridium_IV	2.77	7.06E-06	-1.69	7.85E-03
Firmicutes/Clostridia/Clostridiales /Lachnospiraceae/Acetatifactor	2.96	2.20E-03	-1.77	2.63E-02	Firmicutes/Clostridia/Clostridiales /Lachnospiraceae/Clostridium_XlVb	1.94	3.46E-02	-2.60	8.02E-03
Firmicutes/Clostridia/Clostridiales /Lachnospiraceae/Clostridium_XlVb	2.99	1.14E-03	-2.86	4.10E-03	Firmicutes/Clostridia/Clostridiales /Ruminococcaceae/Oscillibacter	1.94	2.61E-02	-3.14	1.03E-03
Firmicutes/Clostridia/Clostridiales /Ruminococcaceae/Oscillibacter	3.07	2.20E-03	-3.00	1.85E-05	Firmicutes/Clostridia/Clostridiales /Lachnospiraceae/Lachnospiracea_incertae_sedis	4.16	1.36E-03	-4.60	9.99E-04
Firmicutes/Clostridia/Clostridiales /Lachnospiraceae/Lachnospiracea_incertae_sedis	2.28	4.75E-02	-2.89	4.98E-03	Firmicutes/Clostridia/Clostridiales /Clostridiaceae_1/Clostridium_sensu_stricto	-2.72	2.97E-03	2.64	1.20E-04
Firmicutes/Clostridia/Clostridiales /Clostridiaceae_1/Clostridium_sensu_stricto	-2.10	3.20E-02	2.75	2.02E-05	Bacteroidetes/Bacteroidia/Bacteroidales /Porphyromonadaceae/Barnesiella	-5.16	1.51E-10	3.85	3.39E-07
Bacteroidetes/Bacteroidia/Bacteroidales /Porphyromonadaceae/Barnesiella	-3.25	1.31E-05	2.89	4.55E-06	Bacteroidetes/Bacteroidia/Bacteroidales /Rikenellaceae/Alistipes	-4.26	1.10E-11	1.78	1.72E-02
					Bacteroidetes/Bacteroidia/Bacteroidales /Porphyromonadaceae/Barnesiella	-3.40	1.69E-06	1.75	9.14E-03
					Firmicutes/Bacilli/Bacillales /Staphylococcaceae/Staphylococcus	-2.51	2.67E-02	3.70	1.90E-03

Table S6. Individual OTUs altered by high fat diet and corrected by fenugreek supplementation. Taxonomy is based on SILVA 16S rRNA sequence database. Log base 2 fold changes and adjusted p-values (with Benjamini-Hochberg correction) were calculated for pairwise comparisons with DESeq. 2.

Correlation (Pearson) of HF-altered, FG-corrected OTU's to high VIP metabolites in cecum

Individual OTUs altered by HF diet and corrected by FG	HF vs CD						
	Adenine	Guanosine	Inosine	Xanthosine	myo-Inositol	2,3-Dihydroxybenzoate	Carnitine
Firmicutes/Clostridia/Clostridiales /Lachnospiraceae/Acetatifactor/Species 1	-0.1716	0.3959	0.6203	0.6151	0.5045	-0.2256	0.1524
Firmicutes/Clostridia/Clostridiales /Clostridiaceae_1/Clostridium_sensu_stricto	-0.1321	0.0949	-0.0290	-0.1019	-0.2545	0.2220	-0.1324
Bacteroidetes/Bacteroidia/Bacteroidales /Porphyromonadaceae/Barnesiella/Species 4	0.5771	-0.6149	-0.6749	-0.5871	-0.5621	0.0536	-0.1205
Bacteroidetes/Bacteroidia/Bacteroidales /Porphyromonadaceae/Barnesiella/Species 2	-0.6727	0.7928	0.8956	0.8132	0.7904	-0.3582	0.1219
Actinobacteria/Actinobacteria/Coriobacteriales /Coriobacteriaceae/Parvibacter	-0.6027	0.4507	0.4775	0.4266	0.5153	-0.3255	-0.2091
Firmicutes/Clostridia/Clostridiales /Lachnospiraceae/Clostridium_XlVa/Species 16	-0.4995	0.6876	0.8021	0.8562	0.6555	-0.2910	0.2900
Actinobacteria/Actinobacteria/Coriobacteriales /Coriobacteriaceae/Senegalimassilia	-0.6708	0.6880	0.6442	0.7007	0.6160	-0.2664	0.1596
Firmicutes/Clostridia/Clostridiales /Ruminococcaceae/Flavonifractor	-0.4139	0.5620	0.7189	0.7213	0.6465	-0.4287	0.0348
Firmicutes/Clostridia/Clostridiales /Lachnospiraceae/Clostridium_XlVa/Species 17	-0.5463	0.6382	0.7188	0.5896	0.7014	-0.2779	0.0508
Firmicutes/Clostridia/Clostridiales /Lachnospiraceae/Acetatifactor/Species 2	-0.2334	0.4347	0.5852	0.5263	0.3493	-0.2391	0.2452
Firmicutes/Clostridia/Clostridiales /Lachnospiraceae/Clostridium_XlVb	-0.3557	0.4831	0.5257	0.2836	0.3050	0.1943	-0.0677
Firmicutes/Clostridia/Clostridiales /Ruminococcaceae/Oscillibacter/Species 5	-0.1848	0.2754	0.4236	0.5131	0.2754	-0.4339	-0.0527
Firmicutes/Clostridia/Clostridiales /Lachnospiraceae/Lachnospiracea_incertae_sedis	-0.4489	0.4570	0.5435	0.5187	0.7096	-0.0872	0.2526

	HF vs HFFG						
	Adenine	Guanosine	Inosine	Xanthosine	myo-Inositol	2,3-Dihydroxybenzoate	Carnitine
Firmicutes/Clostridia/Clostridiales /Lachnospiraceae/Acetatifactor/Species 1	-0.0055	0.3924	0.1100	-0.2486	0.3291	-0.5831	-0.7214
Firmicutes/Clostridia/Clostridiales /Clostridiaceae_1/Clostridium_sensu_stricto	0.1624	-0.2547	-0.0043	0.0605	-0.3804	0.7024	0.6444
Bacteroidetes/Bacteroidia/Bacteroidales /Porphyromonadaceae/Barnesiella/Species 4	-0.1508	-0.3338	-0.1700	0.1339	-0.1129	0.6369	0.7512
Bacteroidetes/Bacteroidia/Bacteroidales /Porphyromonadaceae/Barnesiella/Species 2	0.1402	0.5959	0.1920	-0.2834	0.2044	-0.7682	-0.8589
Actinobacteria/Actinobacteria/Coriobacteriales /Coriobacteriaceae/Parvibacter	0.3461	-0.2012	-0.4167	-0.2748	-0.1672	-0.4911	-0.6344
Firmicutes/Clostridia/Clostridiales /Lachnospiraceae/Clostridium_XlVa/Species 16	0.2114	0.2614	-0.0681	-0.1862	0.1253	-0.7330	-0.7943
Actinobacteria/Actinobacteria/Coriobacteriales /Coriobacteriaceae/Senegalimassilia	0.3813	0.1217	-0.3285	-0.2921	-0.1115	-0.6290	-0.6587
Firmicutes/Clostridia/Clostridiales /Ruminococcaceae/Flavonifractor	-0.1139	0.1173	-0.2162	-0.4618	-0.0964	-0.7752	-0.6617
Firmicutes/Clostridia/Clostridiales /Lachnospiraceae/Clostridium_XlVa/Species 17	0.0960	0.6820	0.3502	-0.3575	0.2592	-0.4932	-0.5495
Firmicutes/Clostridia/Clostridiales /Lachnospiraceae/Acetatifactor/Species 2	-0.3496	0.3886	0.5173	0.1669	0.4056	-0.3445	-0.3717
Firmicutes/Clostridia/Clostridiales /Lachnospiraceae/Clostridium_XlVb	0.0910	0.5927	0.4207	-0.3101	-0.0670	-0.3616	-0.5834
Firmicutes/Clostridia/Clostridiales /Ruminococcaceae/Oscillibacter/Species 5	0.4838	0.0512	-0.3413	-0.2578	-0.1614	-0.7295	-0.7221
Firmicutes/Clostridia/Clostridiales /Lachnospiraceae/Lachnospiracea_incertae_sedis	-0.0592	0.5014	0.2327	0.0124	0.7136	-0.5629	-0.5273

Table S7. Pearson correlation coefficients for HF-altered and FG-corrected OTUs and metabolites significantly contributing to separation between groups in cecum contents.

Correlation (Pearson) of HF-altered, FG-corrected OTU's to high VIP metabolites in cecum

Individual OTUs altered by HF diet and corrected by FG	HF vs CD						
	Adenine	Guanosine	Inosine	Xanthosine	myo-Inositol	2,3-Dihydroxybenzoate	Carnitine
Firmicutes/Clostridia/Clostridiales /Lachnospiraceae/Acetatifactor/Species 1	ns	ns	0.0027	0.0030	0.0197	ns	ns
Firmicutes/Clostridia/Clostridiales /Clostridiaceae_1/Clostridium_sensu_stricto	ns	ns	ns	ns	ns	ns	ns
Bacteroidetes/Bacteroidia/Bacteroidales /Porphyromonadaceae/Barnesiella/Species 4	0.0062	0.0030	7.9E-04	0.0051	0.0080	ns	ns
Bacteroidetes/Bacteroidia/Bacteroidales /Porphyromonadaceae/Barnesiella/Species 2	8.3E-04	1.8E-05	4.1E-08	7.4E-06	2.0E-05	ns	ns
Actinobacteria/Actinobacteria/Coriobacteriales /Coriobacteriaceae/Parvibacter	0.0038	0.0403	0.0286	ns	0.0168	ns	ns
Firmicutes/Clostridia/Clostridiales /Lachnospiraceae/Clostridium_XlVa/Species 16	0.0211	5.7E-04	1.2E-05	7.4E-07	0.0013	ns	ns
Actinobacteria/Actinobacteria/Coriobacteriales /Coriobacteriaceae/Senegalimassilia	8.7E-04	5.7E-04	0.0016	4.0E-04	0.0029	ns	ns
Firmicutes/Clostridia/Clostridiales /Ruminococcaceae/Flavonifractor	ns	0.0080	2.4E-04	2.2E-04	0.0015	ns	ns
Firmicutes/Clostridia/Clostridiales /Lachnospiraceae/Clostridium_XlVa/Species 17	0.0104	0.0018	2.4E-04	0.0049	4.0E-04	ns	ns
Firmicutes/Clostridia/Clostridiales /Lachnospiraceae/Acetatifactor/Species 2	ns	0.0489	0.0053	0.0143	ns	ns	ns
Firmicutes/Clostridia/Clostridiales /Lachnospiraceae/Clostridium_XlVb	ns	0.0265	0.0144	ns	ns	ns	ns
Firmicutes/Clostridia/Clostridiales /Ruminococcaceae/Oscillibacter/Species 5	ns	ns	ns	0.0174	ns	0.0494	ns
Firmicutes/Clostridia/Clostridiales /Lachnospiraceae/Lachnospiracea_incertae_sedis	0.0412	0.0373	0.0109	0.0160	3.2E-04	ns	ns

	HF vs HFFG						
	Adenine	Guanosine	Inosine	Xanthosine	myo-Inositol	2,3-Dihydroxybenzoate	Carnitine
Firmicutes/Clostridia/Clostridiales /Lachnospiraceae/Acetatifactor/Species 1	ns	ns	ns	ns	ns	0.0088	4.9E-04
Firmicutes/Clostridia/Clostridiales /Clostridiaceae_1/Clostridium_sensu_stricto	ns	ns	ns	ns	ns	8.0E-04	0.0029
Bacteroidetes/Bacteroidia/Bacteroidales /Porphyromonadaceae/Barnesiella/Species 4	ns	ns	ns	ns	ns	0.0034	2.1E-04
Bacteroidetes/Bacteroidia/Bacteroidales /Porphyromonadaceae/Barnesiella/Species 2	ns	0.0071	ns	ns	ns	1.2E-04	2.5E-06
Actinobacteria/Actinobacteria/Coriobacteriales /Coriobacteriaceae/Parvibacter	ns	ns	ns	ns	ns	0.0328	0.0035
Firmicutes/Clostridia/Clostridiales /Lachnospiraceae/Clostridium_XlVa/Species 16	ns	ns	ns	ns	ns	3.6E-04	4.9E-05
Actinobacteria/Actinobacteria/Coriobacteriales /Coriobacteriaceae/Senegalimassilia	ns	ns	ns	ns	ns	0.0039	0.0022
Firmicutes/Clostridia/Clostridiales /Ruminococcaceae/Flavonifractor	ns	ns	ns	0.0465	ns	9.7E-05	0.0020
Firmicutes/Clostridia/Clostridiales /Lachnospiraceae/Clostridium_XlVa/Species 17	ns	0.0013	ns	ns	ns	0.0319	0.0148
Firmicutes/Clostridia/Clostridiales /Lachnospiraceae/Acetatifactor/Species 2	ns	ns	0.0233	ns	ns	ns	ns
Firmicutes/Clostridia/Clostridiales /Lachnospiraceae/Clostridium_XlVb	ns	0.0075	ns	ns	ns	ns	0.0087
Firmicutes/Clostridia/Clostridiales /Ruminococcaceae/Oscillibacter/Species 5	0.0359	ns	ns	ns	ns	3.9E-04	4.8E-04
Firmicutes/Clostridia/Clostridiales /Lachnospiraceae/Lachnospiracea_incertae_sedis	ns	0.0287	ns	ns	6.0E-04	0.0121	0.0203

Table S8. P-values corresponding to pearson correlation for HF-altered and FG-corrected OTUs and metabolites significantly contributing to separation between groups in cecum contents.

Correlation (Pearson) of HF-altered, FG-corrected OTU's to high VIP metabolites in colon

Individual OTUs altered by HF diet and corrected by FG	HF vs CD						
	Adenine	Guanosine	Inosine	Xanthosine	myo-Inositol	2,3-Dihydroxybenzoate	Carnitine
Firmicutes/Clostridia/Clostridiales /Clostridiaceae_1/Clostridium_sensu_stricto	0.3829	-0.2577	-0.1737	-0.2833	-0.4427	0.0958	-0.1456
Bacteroidetes/Bacteroidia/Bacteroidales /Porphyromonadaceae/Barnesiella/Species 4	0.6176	-0.6965	-0.6861	-0.5507	-0.6891	0.0827	-0.0774
Bacteroidetes/Bacteroidia/Bacteroidales /Rikenellaceae/Alistipes	0.6538	-0.6237	-0.6096	-0.7351	-0.7533	0.1770	-0.0270
Bacteroidetes/Bacteroidia/Bacteroidales /Porphyromonadaceae/Barnesiella/Species 2	-0.8703	0.6864	0.6923	0.7133	0.7875	-0.1870	0.0312
Actinobacteria/Actinobacteria/Coriobacteriales /Coriobacteriaceae/Parvibacter	-0.4519	0.4894	0.4630	0.5651	0.4476	-0.3499	-0.2336
Bacteroidetes/Bacteroidia/Bacteroidales /Porphyromonadaceae/Barnesiella/Species 1	0.6064	-0.4719	-0.4323	-0.4585	-0.3714	0.3902	0.3165
Firmicutes/Clostridia/Clostridiales /Lachnospiraceae/Clostridium_XlVa/Species 16	-0.8137	0.6621	0.6635	0.6321	0.7513	-0.0057	0.0869
Actinobacteria/Actinobacteria/Coriobacteriales /Coriobacteriaceae/Senegalimassilia	-0.4762	0.2339	0.2514	0.3851	0.1794	-0.3827	-0.5150
Firmicutes/Clostridia/Clostridiales /Lachnospiraceae/Ruminococcus/Species 1	-0.4247	0.4348	0.4482	0.3213	0.5956	0.2853	0.3587
Firmicutes/Clostridia/Clostridiales /Lachnospiraceae/Clostridium_XlVa/Species 17	-0.5213	0.4866	0.4741	0.4757	0.6003	0.1988	0.4424
Firmicutes/Clostridia/Clostridiales /Ruminococcaceae/Clostridium_IV/Species 2	-0.5566	0.4989	0.4908	0.5289	0.6508	-0.0226	0.0548
Firmicutes/Clostridia/Clostridiales /Lachnospiraceae/Clostridium_XlVb	-0.4898	0.6541	0.6078	0.6197	0.4678	0.1711	0.3034
Firmicutes/Clostridia/Clostridiales /Ruminococcaceae/Oscillibacter/Species 5	-0.3084	0.3221	0.2556	0.2574	0.1544	-0.2089	-0.3358
Firmicutes/Clostridia/Clostridiales /Lachnospiraceae/Lachnospiracea_incertae_sedis	-0.5345	0.4643	0.4731	0.5510	0.7500	0.3480	0.5703
Firmicutes/Bacilli/Bacillales /Staphylococcaceae/Staphylococcus/Species 4	0.4021	-0.7345	-0.6991	-0.4153	-0.5947	-0.2177	-0.1996

Table S9. Pearson correlation coefficients for HF-altered and FG-corrected OTUs and metabolites significantly contributing to separation between groups in colon contents.

	HF vs HFFG						
	Adenine	Guanosine	Inosine	Xanthosine	myo-Inositol	2,3-Dihydroxybenzoate	Carnitine
Firmicutes/Clostridia/Clostridiales /Clostridiaceae_1/Clostridium_sensu_stricto	0.2901	-0.0966	0.0686	0.1825	-0.0773	0.5202	0.5834
Bacteroidetes/Bacteroidia/Bacteroidales /Porphyromonadaceae/Barnesiella/Species 4	0.0348	-0.4662	-0.3307	-0.3041	-0.0950	0.6094	0.6473
Bacteroidetes/Bacteroidia/Bacteroidales /Rikenellaceae/Alistipes	-0.1277	-0.1068	0.1276	-0.1836	-0.1947	0.3873	0.3837
Bacteroidetes/Bacteroidia/Bacteroidales /Porphyromonadaceae/Barnesiella/Species 2	-0.1410	0.4121	0.2049	0.2117	-0.0130	-0.8317	-0.8961
Actinobacteria/Actinobacteria/Coriobacteriales /Coriobacteriaceae/Parvibacter	0.2715	0.0410	0.0536	-0.0079	-0.3096	-0.6422	-0.6439
Bacteroidetes/Bacteroidia/Bacteroidales /Porphyromonadaceae/Barnesiella/Species 1	-0.1661	-0.1133	-0.0268	-0.1876	0.2432	0.5589	0.5259
Firmicutes/Clostridia/Clostridiales /Lachnospiraceae/Clostridium_XlVa/Species 16	-0.2641	0.3487	0.1310	0.0677	-0.1226	-0.7478	-0.8013
Actinobacteria/Actinobacteria/Coriobacteriales /Coriobacteriaceae/Senegalimassilia	0.3736	0.0413	-0.1339	0.2236	-0.2330	-0.6337	-0.6392
Firmicutes/Clostridia/Clostridiales /Lachnospiraceae/Ruminococcus/Species 1	-0.2294	0.2381	0.1278	-0.0284	0.2918	-0.1671	-0.2141
Firmicutes/Clostridia/Clostridiales /Lachnospiraceae/Clostridium_XlVa/Species 17	-0.1679	0.4255	0.2314	0.1678	0.2330	-0.3912	-0.4323
Firmicutes/Clostridia/Clostridiales /Ruminococcaceae/Clostridium_IV/Species 2	-0.3338	0.1167	-0.0639	-0.0803	-0.1349	-0.5415	-0.5797
Firmicutes/Clostridia/Clostridiales /Lachnospiraceae/Clostridium_XlVb	-0.3042	0.6190	0.4909	0.4803	0.0412	-0.4680	-0.4946
Firmicutes/Clostridia/Clostridiales /Ruminococcaceae/Oscillibacter/Species 5	-0.2114	0.1400	0.0673	0.1315	-0.5338	-0.7920	-0.7913
Firmicutes/Clostridia/Clostridiales /Lachnospiraceae/Lachnospiracea_incertae_sedis	-0.1139	0.3143	0.1409	-0.0012	0.3419	-0.4002	-0.4763
Firmicutes/Bacilli/Bacillales /Staphylococcaceae/Staphylococcus/Species 4	0.3604	-0.5155	-0.5644	-0.1470	0.0169	0.2622	0.2816

Table S9 continued.

Correlation (Pearson) of HF-altered, FG-corrected OTU's to high VIP metabolites in colon

Individual OTUs altered by HF diet and corrected by FG	HF vs CD						
	Adenine	Guanosine	Inosine	Xanthosine	myo-Inositol	2,3-Dihydroxybenzoate	Carnitine
Firmicutes/Clostridia/Clostridiales /Clostridiaceae_1/Clostridium_sensu_stricto	*ns*	*ns*	*ns*	*ns*	0.0445	*ns*	*ns*
Bacteroidetes/Bacteroidia/Bacteroidales /Porphyromonadaceae/Barnesiella/Species 4	0.0029	4.5E-04	5.9E-04	0.0097	5.5E-04	*ns*	*ns*
Bacteroidetes/Bacteroidia/Bacteroidales /Rikenellaceae/Alistipes	0.0013	0.0025	0.0033	1.5E-04	8.1E-05	*ns*	*ns*
Bacteroidetes/Bacteroidia/Bacteroidales /Porphyromonadaceae/Barnesiella/Species 2	2.9E-07	5.9E-04	5.1E-04	2.8E-04	2.3E-05	*ns*	*ns*
Actinobacteria/Actinobacteria/Coriobacteriales /Coriobacteriaceae/Parvibacter	0.0397	0.0243	0.0346	0.0076	0.0419	*ns*	*ns*
Bacteroidetes/Bacteroidia/Bacteroidales /Porphyromonadaceae/Barnesiella/Species 1	0.0036	0.0308	*ns*	0.0366	*ns*	*ns*	*ns*
Firmicutes/Clostridia/Clostridiales /Lachnospiraceae/Clostridium_XlVa/Species 16	7.2E-06	0.0011	0.0010	0.0021	8.7E-05	*ns*	*ns*
Actinobacteria/Actinobacteria/Coriobacteriales /Coriobacteriaceae/Senegalimassilia	0.0291	*ns*	*ns*	*ns*	*ns*	*ns*	0.0169
Firmicutes/Clostridia/Clostridiales /Lachnospiraceae/Ruminococcus/Species 1	*ns*	0.0489	0.0416	*ns*	0.0044	*ns*	*ns*
Firmicutes/Clostridia/Clostridiales /Lachnospiraceae/Clostridium_XlVa/Species 17	0.0154	0.0253	0.0299	0.0293	0.0040	*ns*	0.0446
Firmicutes/Clostridia/Clostridiales /Ruminococcaceae/Clostridium_IV/Species 2	0.0088	0.0213	0.0239	0.0137	0.0014	*ns*	*ns*
Firmicutes/Clostridia/Clostridiales /Lachnospiraceae/Clostridium_XlVb	0.0242	0.0013	0.0035	0.0027	0.0325	*ns*	*ns*
Firmicutes/Clostridia/Clostridiales /Ruminococcaceae/Oscillibacter/Species 5	*ns*	*ns*	*ns*	*ns*	*ns*	*ns*	*ns*
Firmicutes/Clostridia/Clostridiales /Lachnospiraceae/Lachnospiracea_incertae_sedis	0.0126	0.0340	0.0303	0.0096	9.0E-05	*ns*	0.0069
Firmicutes/Bacilli/Bacillales /Staphylococcaceae/Staphylococcus/Species 4	*ns*	1.5E-04	4.2E-04	*ns*	0.0045	*ns*	*ns*

Table S10. P-values corresponding to pearson correlation for HF-altered and FG-corrected OTUs and metabolites significantly contributing to separation between groups in colon contents.

	HF vs HFFG						
	Adenine	Guanosine	Inosine	Xanthosine	myo-Inositol	2,3-Dihydroxybenzoate	Carnitine
Firmicutes/Clostridia/Clostridiales /Clostridiaceae_1/Clostridium_sensu_stricto	*ns*	*ns*	*ns*	*ns*	*ns*	0.0131	0.0044
Bacteroidetes/Bacteroidia/Bacteroidales /Porphyromonadaceae/Barnesiella/Species 4	*ns*	0.0288	*ns*	*ns*	*ns*	0.0026	0.0011
Bacteroidetes/Bacteroidia/Bacteroidales /Rikenellaceae/Alistipes	*ns*	*ns*	*ns*	*ns*	*ns*	*ns*	*ns*
Bacteroidetes/Bacteroidia/Bacteroidales /Porphyromonadaceae/Barnesiella/Species 2	*ns*	*ns*	*ns*	*ns*	*ns*	1.6E-06	1.7E-08
Actinobacteria/Actinobacteria/Coriobacteriales /Coriobacteriaceae/Parvibacter	*ns*	*ns*	*ns*	*ns*	*ns*	0.0013	0.0012
Bacteroidetes/Bacteroidia/Bacteroidales /Porphyromonadaceae/Barnesiella/Species 1	*ns*	*ns*	*ns*	*ns*	*ns*	0.0069	0.0119
Firmicutes/Clostridia/Clostridiales /Lachnospiraceae/Clostridium_XlVa/Species 16	*ns*	*ns*	*ns*	*ns*	*ns*	6.3E-05	7.4E-06
Actinobacteria/Actinobacteria/Coriobacteriales /Coriobacteriaceae/Senegalimassilia	*ns*	*ns*	*ns*	*ns*	*ns*	0.0015	0.0014
Firmicutes/Clostridia/Clostridiales /Lachnospiraceae/Ruminococcus/Species 1	*ns*	*ns*	*ns*	*ns*	*ns*	*ns*	*ns*
Firmicutes/Clostridia/Clostridiales /Lachnospiraceae/Clostridium_XlVa/Species 17	*ns*	0.0483	*ns*	*ns*	*ns*	*ns*	0.0445
Firmicutes/Clostridia/Clostridiales /Ruminococcaceae/Clostridium_IV/Species 2	*ns*	*ns*	*ns*	*ns*	*ns*	0.0092	0.0047
Firmicutes/Clostridia/Clostridiales /Lachnospiraceae/Clostridium_XlVb	*ns*	0.0021	0.0203	0.0237	*ns*	0.0280	0.0193
Firmicutes/Clostridia/Clostridiales /Ruminococcaceae/Oscillibacter/Species 5	*ns*	*ns*	*ns*	*ns*	0.0105	1.1E-05	1.2E-05
Firmicutes/Clostridia/Clostridiales /Lachnospiraceae/Lachnospiracea_incertae_sedis	*ns*	*ns*	*ns*	*ns*	*ns*	*ns*	0.0250
Firmicutes/Bacilli/Bacillales /Staphylococcaceae/Staphylococcus/Species 4	*ns*	0.0141	0.0062	*ns*	*ns*	*ns*	*ns*

Table S10 continued.

Raw OTU count tables:

Table S11 (pages S-25 to S-69). Raw OTU count data from 16S sequencing of jejunum contents.

Kingdom	Baceria	-Bacteria	Bacteria	Bacteria	Bacteria	Bacteria	Bacteria	Bacteria	Bacteria	Bacteria
Phylum	Firmicutes	Verrucomicrobia	Firmicutes	Firmicutes	Firmicutes	Firmicutes	Actinobacteria	Firmicutes	Firmicutes	Firmicutes
Class	Erysipelotrichia	Verrucomicrobiae	Bacilli	Erysipelotrichia	Bacilli	Bacilli	Actinobacteria	Bacilli	Bacilli	Clostridia
Order	Erysipelotrichales	Verrucomicrobiales	Lactobacillales	Erysipelotrichales	Bacillales	Lactobacillales	Bifidobacteriales	Bacillales	Bacillales	Clostridiales
Family	Erysipelotrichaceae	Verrucomicrobiaceae	Enterococcaceae	Erysipelotrichaceae	Staphylococcaceae	Lactobacillaceae	Bifidobacteriaceae	Bacillaceae_	Staphylococcaceae	Peptostreptococcaceae
Genus	Allobaculum	Akkermansia	Enterococcus	Catenisphaera	Staphylococcus	Lactobacillus	Bifidobacterium	Bacillus	Staphylococcus	Romboutsia
#NAME	ZOTU_0001	ZOTU_0002	ZOTU_0003	ZOTU_0004	ZOTU_0005	ZOTU_0006	ZOTU_0007	ZOTU_0008	ZOTU_0009	ZOTU_0010
CD_49	1038	5595	3340	11259	386	810	1523	565	149	541
CD_50	28070	9224	1121	17377	163	338	2735	438	957	121
CD_52	72413	13421	276	20503	43	7656	7487	12	621	31
CD_65	12201	9941	808	6285	364	2213	8434	485	5	212
CD_67	1927	3427	250	4251	159	5569	347	283	14	109
CD_68	11886	15509	122	20005	40	332	1738	53	3	22
CD_78	3794	1421	277	1823	56	27	200	776	8	71
CD_79	1535	8535	503	888	69	28	137	1690	6	116
CD_80	27	2951	2783	255	260	93	23	6737	1	421
CDFG_53	3548	9830	49203	391	4029	656	3004	12	27	68
CDFG_54	3658	33267	13878	972	2964	8992	25057	96	175	364
CDFG_55	16845	51324	34310	4115	168	10452	1033	117	190	288
CDFG_56	4291	49021	32257	1829	1737	6752	967	7451	3010	1024
CDFG_70	2315	50085	921	1485	92	445	1009	932	98	1944
CDFG_71	4906	12281	457	10754	67	216	254	604	159	108
HF_41	144	362	64462	800	265	25	81	91	82	68
HF_43	1445	186	1346	487	51	8915	43	54	8682	628
HF_44	876	258	426	262	15	7911	15	36	5350	119
HF_57	2817	2376	1787	2419	809	203	1812	880	2	388
HF_58	1700	13626	2483	1959	1219	3262	781	1401	103	790
HF_59	937	7688	4298	902	354	12142	220	635	26	518
HF_60	5329	5961	7213	1517	286	778	377	100	22	132
HF_73	1617	6780	2768	1669	1290	583	402	208	1	603
HFFG_45	580	758	1086	704	25	1542	205	78	13758	534
HFFG_46	336	1109	1070	1526	17	6900	192	15	50386	57
HFFG_47	20	19	3322	14	36	14	3	2	45737	4
HFFG_48	1141	4588	6316	744	208	979	678	2278	19394	504
HFFG_61	897	3113	1661	769	84	317	506	122	26	588
HFFG_62	149	5996	117	732	11	1958	83	58	10	130
HFFG_75	865	1941	746	2076	131	3373	118	309	27	249
HFFG_76	1629	20993	3946	4955	243	5140	1129	1309	80	827

Kingdom	Bacteria	Bacteria	Bacteria	Bacteria	Bacteria	Bacteria	Bacteria	Bacteria	Bacteria	Bacteria
Phylum	Firmicutes	Bacteroidetes	Firmicutes	Firmicutes	Actinobacteria	Firmicutes	Firmicutes	Firmicutes	Firmicutes	Bacteroidetes
Class	Bacilli	Bacteroidia	Clostridia	Clostridia	Actinobacteria	Bacilli	Clostridia	Clostridia	Clostridia	Bacteroidia
Order	Lactobacillales	Bacteroidales	Clostridiales	Clostridiales	Coriobacteriales	Bacillales	Clostridiales	Clostridiales	Clostridiales	Bacteroidales
Family	Streptococcaceae	Bacteroidaceae	Lachnospiraceae	Lachnospiraceae	Coriobacteriaceae	Staphylococcaceae	Lachnospiraceae	Clostridiaceae_	Peptostreptococcaceae	Porphyromonadaceae
Genus	Lactococcus	Bacteroides	Clostridium_XlVa	Acetatifactor	Enterorhabdus	Staphylococcus	Clostridium_XlVa	Clostridium_sensu_stricto	Romboutsia	Barnesiella
#NAME	ZOTU_0011	ZOTU_0012	ZOTU_0013	ZOTU_0014	ZOTU_0015	ZOTU_0016	ZOTU_0017	ZOTU_0018	ZOTU_0019	ZOTU_0020
CD_49	1524	11	32	13	38	17	1	445	188	39
CD_50	925	7	15	19	723	17	0	194	51	80
CD_52	2455	3	0	19	535	1	13	2	8	34
CD_65	683	16	22	51	262	19	0	435	90	33
CD_67	77	2	11	39	20	4	0	196	47	36
CD_68	619	3	4	334	464	3	1	89	8	59
CD_78	450	1	9	0	25	1	0	78	14	12
CD_79	306	12	9	0	64	0	0	46	44	122
CD_80	56	0	34	0	6	0	0	114	186	1
CDFG_53	11	49	0	1	134	1	59	30	38	32
CDFG_54	83	155	0	0	2710	5	20	1039	121	13
CDFG_55	56	5299	1	0	1180	0	55	91	135	51
CDFG_56	500	54	82	23	105	30	32	733	377	92
CDFG_70	317	7	5	49	54	2	431	85	447	157
CDFG_71	289	2	3	10	77	1	16	40	36	119
HF_41	23	1	7	1	4	7	0	94	28	3
HF_43	32	2	18	19	6	3	1	73	280	0
HF_44	190	2	115	18	0	0	0	10	52	0
HF_57	1589	14	57	28	36	50	2	726	191	63
HF_58	3685	23	62	4507	3291	36	0	2037	291	151
HF_59	4172	3	28	1513	239	34	0	287	226	26
HF_60	5565	1	5	84	63	4	0	86	57	11
HF_73	185	7	14	105	22	42	2	528	262	36
HFFG_45	291	0	1	22	9	10	4	174	240	2
HFFG_46	1355	0	0	0	65	0	0	51	36	1
HFFG_47	0	0	0	0	0	1	0	2	1	0
HFFG_48	78	2	40	12	8	155	0	546	199	12
HFFG_61	161	2	46	1	16	2	4	71	239	10
HFFG_62	368	0	1	4	11	2	4	4	75	0
HFFG_75	575	2	23	22	17	1	0	319	115	16
HFFG_76	482	11	136	117	155	3	15	487	374	173

Kingdom	Bacteria	Bacteria	Bacteria	Bacteria	Bacteria	Bacteria	Bacteria	Bacteria	Bacteria	Bacteria	Bacteria
Phylum	Firmicutes	Firmicutes	Bacteroidetes	Proteobacteria	Firmicutes	Actinobacteria	Firmicutes	Bacteroidetes	Firmicutes	Firmicutes	Firmicutes
Class	Erysipelotrichia	Clostridia	Bacteroidia	Betaproteobacteria	Clostridia	Actinobacteria	Clostridia	Bacteroidia	Clostridia	Bacilli	Erysipelotrichia
Order	Erysipelotrichales	Clostridiales	Bacteroidales	Burkholderiales	Clostridiales	Coriobacteriales	Clostridiales	Bacteroidales	Clostridiales	Bacillales	Erysipelotrichales
Family	Erysipelotrichaceae	Ruminococcaceae	Rikenellaceae	Sutterellaceae	Lachnospiraceae	Coriobacteriaceae	Eubacteriaceae	Porphyromonadaceae	Ruminococcaceae	Planococcaceae	Erysipelotrichaceae
Genus	Allobaculum	Pseudoflavonifractor	Alistipes	Parasutterella	Clostridium_XlVa	Enterorhabdus	Eubacterium	Parabacteroides	Pseudoflavonifractor	Lysinibacillus	Turicibacter
#NAME	ZOTU_0021	ZOTU_0022	ZOTU_0023	ZOTU_0024	ZOTU_0025	ZOTU_0026	ZOTU_0027	ZOTU_0028	ZOTU_0029	ZOTU_0030	ZOTU_0031
CD_49	492	4	6	49	4	197	2	0	1	0	60
CD_50	265	4	3	290	6	103	2	0	0	0	27
CD_52	5	0	4	382	25	444	0	0	0	0	1
CD_65	927	7	10	1416	5	118	1	18	0	0	32
CD_67	657	1	4	198	4	90	0	0	0	0	12
CD_68	9	5	2	2518	1	601	3	152	0	0	1
CD_78	99	1	2	102	0	8	3	0	0	0	26
CD_79	8	1	2	674	12	15	12	0	0	0	25
CD_80	34	1	4	2	0	22	153	0	0	0	19
CDFG_53	19	0	0	32	0	106	0	0	0	2	4
CDFG_54	2	3	0	9	0	158	21	0	0	1	2
CDFG_55	7	4	23	263	1	544	1	0	0	0	0
CDFG_56	2527	2	16	70	9	99	76	8	0	7	99
CDFG_70	14	15	7	42	7	65	2	0	1	2	923
CDFG_71	15	8	5	91	6	372	1	0	0	0	62
HF_41	307	0	0	0	0	6	0	0	0	0	6
HF_43	26	0	0	1	0	3	0	0	1	1	4
HF_44	8	0	0	0	2	2	0	0	1	0	1
HF_57	1004	7	9	184	2	44	5	0	2	0	88
HF_58	5435	10	21	40	24	1432	4	0	1	3	118
HF_59	466	4	7	11	6	35	2	0	0	0	28
HF_60	38	0	0	11	5	25	0	0	0	0	5
HF_73	2081	2	18	16	5	62	2	1	0	0	88
HFFG_45	56	1	1	0	0	7	0	0	0	0	2
HFFG_46	1	3	0	0	1	24	1	0	0	0	3
HFFG_47	5	0	0	0	0	0	0	0	0	0	0
HFFG_48	2122	2	1	3	1	22	19	3	0	1	27
HFFG_61	87	1	1	2	271	5	1	2	0	2	3
HFFG_62	15	0	0	1	5	1	0	0	0	0	9
HFFG_75	2367	3	3	24	0	22	0	2	0	0	56
HFFG_76	7446	51	6	72	29	140	0	0	1	1	177

Kingdom	Bacteria	Bacteria	Bacteria	Bacteria	Bacteria	Bacteria	Bacteria	Bacteria	Bacteria	Bacteria	Bacteria
Phylum	Bacteroidetes	Firmicutes	Firmicutes	Actinobacteria	Bacteroidetes	Firmicutes	Firmicutes	Firmicutes	Actinobacteria	Firmicutes	Firmicutes
Class	Bacteroidia	Clostridia	Erysipelotrichia	Actinobacteria	Bacteroidia	Clostridia	Clostridia	Clostridia	Actinobacteria	Clostridia	Clostridia
Order	Bacteroidales	Clostridiales	Erysipelotrichales	Coriobacteriales	Bacteroidales	Clostridiales	Clostridiales	Clostridiales	Coriobacteriales	Clostridiales	Clostridiales
Family	Porphyromonadaceae	Lachnospiraceae	Erysipelotrichaceae	Coriobacteriaceae	Porphyromonadaceae	Clostridiaceae_	Lachnospiraceae	Lachnospiraceae	Coriobacteriaceae	Lachnospiraceae	Lachnospiraceae
Genus	Barnesiella	Clostridium_XlVa	Clostridium_XVIII	Parvibacter	Barnesiella	Alkaliphilus	Clostridium_XlVa	Clostridium_XlVa	Senegalimassilia	Clostridium_XlVa	Clostridium_XlVa
#NAME	ZOTU_0032	ZOTU_0033	ZOTU_0034	ZOTU_0035	ZOTU_0036	ZOTU_0037	ZOTU_0038	ZOTU_0039	ZOTU_0040	ZOTU_0041	ZOTU_0042
CD_49	0	1	13	20	10	0	0	44	8	2	30
CD_50	0	15	6	192	13	0	0	164	25	1	14
CD_52	0	11	1	261	80	0	1	18	41	3	21
CD_65	1	19	24	21	50	0	4	234	15	0	28
CD_67	0	0	15	3	0	0	0	120	5	0	2
CD_68	1	13	0	70	33	0	0	184	25	0	168
CD_78	0	0	2	2	3	0	0	3	2	0	6
CD_79	0	5	8	9	70	0	0	7	6	1	58
CD_80	0	0	60	14	1	0	2	0	1	1	5
CDFG_53	39	0	0	52	4	0	1	120	80	0	1
CDFG_54	96	0	0	303	62	0	79	832	332	17	2
CDFG_55	74	0	1	488	156	0	4	404	140	2	10
CDFG_56	201	4	31	33	71	0	33	534	69	15	11
CDFG_70	1	9	3	27	67	0	35	10	33	31	28
CDFG_71	0	10	3	73	43	0	1	138	66	22	99
HF_41	0	0	6	0	1	0	0	3	5	0	0
HF_43	0	1	0	2	0	0	0	124	1	1	0
HF_44	0	0	0	0	0	0	4	6	0	1	2
HF_57	0	3	50	5	8	0	1	21	9	1	3
HF_58	31	4	80	131	16	0	0	163	91	4	204
HF_59	8	1	28	17	2	0	0	26	37	1	232
HF_60	35	0	3	10	2	0	0	11	16	0	11
HF_73	18	4	53	14	7	0	14	62	16	3	33
HFFG_45	1	1	1	1	0	0	1	0	3	1	130
HFFG_46	0	0	0	8	1	0	0	1	10	3	28
HFFG_47	0	0	0	1	0	0	0	0	1	0	0
HFFG_48	3	0	20	2	2	0	3	8	12	9	43
HFFG_61	1	4	1	5	1	0	2	55	12	2	19
HFFG_62	0	0	0	0	1	0	0	35	2	1	1
HFFG_75	0	0	8	0	2	0	0	14	6	0	1
HFFG_76	3	5	29	41	46	0	5	248	33	5	52

Kingdom	Bacteria	Bacteria	Bacteria	Bacteria	Bacteria	Bacteria	Bacteria	Bacteria	Bacteria	Bacteria	Bacteria
Phylum	Firmicutes	Firmicutes	Actinobacteria	Firmicutes	Firmicutes	Firmicutes	Firmicutes	Bacteroidetes	Firmicutes	Firmicutes	Firmicutes
Class	Clostridia	Clostridia	Actinobacteria	Bacilli	Clostridia	Clostridia	Clostridia	Bacteroidia	Clostridia	Clostridia	Clostridia
Order	Clostridiales	Clostridiales	Coriobacteriales	Bacillales	Clostridiales	Clostridiales	Clostridiales	Bacteroidales	Clostridiales	Clostridiales	Clostridiales
Family	Lachnospiraceae	Lachnospiraceae	Coriobacteriaceae	Staphylococcaceae	Lachnospiraceae	Ruminococcaceae	Lachnospiraceae	Porphyromonadaceae	Clostridiaceae_	Clostridiaceae_	Ruminococcaceae
Genus	Clostridium_XlVa	Blautia	Asaccharobacter	Staphylococcus	Acetatifactor	Oscillibacter	Clostridium_XlVa	Parabacteroides	Alkaliphilus	Alkaliphilus	Flavonifractor
#NAME	ZOTU_0043	ZOTU_0044	ZOTU_0045	ZOTU_0046	ZOTU_0047	ZOTU_0048	ZOTU_0049	ZOTU_0050	ZOTU_0051	ZOTU_0052	ZOTU_0053
CD_49	0	1	4	0	28	1	2	4	5	0	0
CD_50	1	6	1	0	34	0	5	2	13	0	1
CD_52	0	1	0	0	74	0	2	0	4	0	0
CD_65	2	0	3	0	71	0	15	75	14	0	0
CD_67	0	0	0	2	13	0	1	34	1	0	0
CD_68	0	0	3	1	29	0	4	208	15	0	0
CD_78	0	0	1	0	5	0	3	1	1	0	0
CD_79	0	1	2	0	45	0	11	9	3	0	1
CD_80	0	3	3	0	0	0	0	1	0	0	0
CDFG_53	0	0	0	2	34	0	0	0	5	4	0
CDFG_54	20	5	0	0	125	0	2	0	5	1	0
CDFG_55	6	6	1	0	97	1	1	0	17	65	1
CDFG_56	16	2	33	0	142	0	2	14	7	9	2
CDFG_70	30	51	2	2	334	5	169	0	1	21	27
CDFG_71	142	5	2	1	195	3	14	0	1	9	3
HF_41	0	0	2	0	0	0	1	1	0	0	0
HF_43	0	0	0	0	54	0	0	0	0	0	0
HF_44	0	0	0	0	45	0	0	0	0	0	0
HF_57	0	1	8	0	13	1	3	17	1	0	0
HF_58	0	3	102	0	161	9	0	14	23	0	2
HF_59	0	0	0	0	165	0	1	2	1	0	0
HF_60	0	0	1	0	7	1	0	0	0	0	0
HF_73	0	12	38	2	14	0	4	5	22	0	1
HFFG_45	0	0	1	1	102	0	1	0	2	1	0
HFFG_46	0	2	0	1	23	0	0	0	1	3	0
HFFG_47	0	0	0	13	0	0	0	0	0	0	0
HFFG_48	0	0	6	6	11	0	4	4	2	1	0
HFFG_61	5	0	3	0	28	0	0	0	3	2	0
HFFG_62	0	0	0	1	13	0	0	0	0	0	1
HFFG_75	0	0	4	1	5	0	1	3	0	0	0
HFFG_76	0	6	151	0	116	6	5	7	9	59	5

Kingdom	Bacteria	Bacteria	Bacteria	Bacteria	Bacteria	Bacteria	Bacteria	Bacteria	Bacteria	Bacteria	Bacteria
Phylum	Firmicutes	Bacteroidetes	Firmicutes	Firmicutes	Firmicutes	Firmicutes	Firmicutes	Firmicutes	Firmicutes	Bacteroidetes	Firmicutes
Class	Clostridia	Bacteroidia	Clostridia	Clostridia	Clostridia	Clostridia	Clostridia	Clostridia	Clostridia	Bacteroidia	Clostridia
Order	Clostridiales	Bacteroidales	Clostridiales	Clostridiales	Clostridiales	Clostridiales	Clostridiales	Clostridiales	Clostridiales	Bacteroidales	Clostridiales
Family	Ruminococcaceae	Porphyromonadaceae	Lachnospiraceae	Lachnospiraceae	Ruminococcaceae	Lachnospiraceae	Clostridiaceae_	Lachnospiraceae	Lachnospiraceae	Porphyromonadaceae	Lachnospiraceae
Genus	Clostridium_IV	Barnesiella	Clostridium_XlVa	Acetatifactor	Oscillibacter	Ruminococcus	Alkaliphilus	Blautia	Clostridium_XlVa	Barnesiella	Acetatifactor
#NAME	ZOTU_0054	ZOTU_0055	ZOTU_0056	ZOTU_0057	ZOTU_0058	ZOTU_0059	ZOTU_0060	ZOTU_0061	ZOTU_0062	ZOTU_0063	ZOTU_0064
CD_49	3	0	1	0	0	0	0	0	0	1	0
CD_50	4	0	4	12	0	0	0	5	0	1	3
CD_52	0	4	0	20	0	7	0	306	14	5	11
CD_65	1	5	56	24	2	4	0	2	3	0	10
CD_67	0	3	0	4	0	0	0	1	0	1	0
CD_68	0	16	7	67	1	7	0	1	3	16	2
CD_78	2	0	0	1	0	0	0	0	0	0	1
CD_79	1	0	0	26	0	5	0	0	0	0	1
CD_80	0	0	0	0	0	0	0	0	0	0	0
CDFG_53	0	0	1	2	0	0	0	63	3	2	5
CDFG_54	0	3	2	63	1	1	0	72	22	5	43
CDFG_55	0	23	3	19	0	2	0	105	17	29	11
CDFG_56	2	22	1	41	2	0	0	81	49	23	98
CDFG_70	5	0	167	503	4	14	1	528	54	0	154
CDFG_71	0	0	2	55	1	1	0	87	23	2	15
HF_41	1	0	0	0	0	0	0	0	0	0	0
HF_43	0	0	0	8	0	0	0	1	0	0	0
HF_44	0	0	0	36	0	0	0	1	0	0	0
HF_57	4	1	3	6	1	0	0	0	0	0	0
HF_58	8	1	6	11	1	1	0	0	0	1	1
HF_59	2	0	1	2	0	0	0	0	0	0	0
HF_60	0	0	0	0	0	0	0	9	0	1	0
HF_73	4	0	2	22	0	0	0	0	1	1	6
HFFG_45	0	0	4	11	0	0	0	9	30	0	24
HFFG_46	0	0	0	20	0	0	0	2	5	1	3
HFFG_47	0	0	0	0	0	0	0	0	0	0	0
HFFG_48	4	0	2	3	0	0	0	1	1	0	49
HFFG_61	0	0	0	6	0	0	0	0	2	0	65
HFFG_62	0	0	0	1	0	0	0	0	5	0	43
HFFG_75	0	0	0	2	0	1	0	1	0	0	1
HFFG_76	2	46	1	17	2	17	12	19	13	41	19

Kingdom	Bacteria	Bacteria	Bacteria	Bacteria	Bacteria	Bacteria	Bacteria	Bacteria	Bacteria	Bacteria	Bacteria
Phylum	Firmicutes	Firmicutes	Actinobacteria	Firmicutes	Firmicutes	Firmicutes	Firmicutes	Firmicutes	Firmicutes	Firmicutes	Firmicutes
Class	Clostridia	Clostridia	Actinobacteria	Clostridia	Clostridia	Clostridia	Clostridia	Clostridia	Clostridia	Clostridia	Clostridia
Order	Clostridiales	Clostridiales	Coriobacteriales	Clostridiales	Clostridiales	Clostridiales	Clostridiales	Clostridiales	Clostridiales	Clostridiales	Clostridiales
Family	Ruminococcaceae	Lachnospiraceae	Coriobacteriaceae	Lachnospiraceae	Ruminococcaceae	Ruminococcaceae	Lachnospiraceae	Lachnospiraceae	Lachnospiraceae	Catabacteriaceae	Lachnospiraceae
Genus	Clostridium_III	Acetatifactor	Paraeggerthella	Clostridium_XlVa	Ruminococcus	Clostridium_IV	Clostridium_XlVa	Clostridium_XlVa	Acetatifactor	Catabacter	Clostridium_XlVa
#NAME	ZOTU_0065	ZOTU_0066	ZOTU_0067	ZOTU_0068	ZOTU_0069	ZOTU_0070	ZOTU_0071	ZOTU_0072	ZOTU_0073	ZOTU_0074	ZOTU_0075
CD_49	74	13	61	5	0	0	0	0	8	1	0
CD_50	41	37	12	6	5	0	0	0	12	0	1
CD_52	24	124	102	11	3	11	0	4	20	1	0
CD_65	9	22	7	0	3	8	0	0	13	1	0
CD_67	9	3	1	0	1	0	0	0	2	0	0
CD_68	172	55	21	0	1	4	0	0	10	0	0
CD_78	5	0	1	0	0	0	0	0	1	1	2
CD_79	1	8	1	0	4	0	0	2	10	0	0
CD_80	4	0	0	0	0	0	0	0	1	0	0
CDFG_53	20	10	10	0	0	9	22	0	7	0	0
CDFG_54	30	2	98	2	35	82	6	0	30	0	0
CDFG_55	66	20	63	0	4	11	17	0	34	0	0
CDFG_56	60	30	23	1	18	31	53	0	35	4	0
CDFG_70	13	37	26	2	73	129	545	2	122	0	4
CDFG_71	0	18	10	21	46	2	12	3	76	2	1
HF_41	2	0	0	0	0	0	0	0	0	0	0
HF_43	0	12	0	0	0	0	0	0	18	0	0
HF_44	7	17	0	0	0	0	0	0	9	0	0
HF_57	25	5	1	0	2	1	0	0	1	1	0
HF_58	166	286	59	60	6	5	0	4	49	3	1
HF_59	27	71	2	1	1	0	0	1	46	1	0
HF_60	6	22	4	3	0	0	0	4	2	0	0
HF_73	17	5	3	1	2	2	0	1	5	1	1
HFFG_45	21	621	9	0	12	1	56	0	22	0	0
HFFG_46	97	4	34	1	12	6	30	0	13	0	0
HFFG_47	0	0	0	0	0	0	0	0	0	0	0
HFFG_48	11	27	2	0	1	0	18	0	9	0	0
HFFG_61	17	337	2	0	2	1	0	0	5	0	0
HFFG_62	73	0	1	0	0	1	0	0	3	0	0
HFFG_75	6	2	2	0	0	1	0	0	1	0	0
HFFG_76	37	29	11	2	4	7	1	3	48	3	0

Kingdom	Bacteria	Bacteria	Bacteria	Bacteria	Bacteria	Bacteria	Bacteria	Bacteria	Bacteria	Bacteria	Bacteria
Phylum	Firmicutes	Firmicutes	Firmicutes	Firmicutes	Bacteroidetes	Firmicutes	Firmicutes	Firmicutes	Firmicutes	Firmicutes	Firmicutes
Class	Clostridia	Clostridia	Clostridia	Clostridia	Bacteroidia	Clostridia	Clostridia	Clostridia	Clostridia	Clostridia	Clostridia
Order	Clostridiales	Clostridiales	Clostridiales	Clostridiales	Bacteroidales	Clostridiales	Clostridiales	Clostridiales	Clostridiales	Clostridiales	Clostridiales
Family	Ruminococcaceae	Ruminococcaceae	Lachnospiraceae	Lachnospiraceae	Porphyromonadaceae	Ruminococcaceae	Ruminococcaceae	Ruminococcaceae	Clostridiaceae_	Ruminococcaceae	Lachnospiraceae
Genus	Clostridium_III	Sporobacter	Acetatifactor	Murimonas	Coprobacter	Oscillibacter	Clostridium_IV	Pseudoflavonifractor	Alkaliphilus	Clostridium_IV	Acetatifactor
#NAME	ZOTU_0076	ZOTU_0077	ZOTU_0078	ZOTU_0079	ZOTU_0080	ZOTU_0081	ZOTU_0082	ZOTU_0083	ZOTU_0084	ZOTU_0085	ZOTU_0086
CD_49	0	2	0	0	0	0	0	0	0	2	7
CD_50	1	0	0	0	0	0	0	1	2	2	12
CD_52	0	1	0	16	0	0	0	0	0	0	25
CD_65	2	20	15	10	20	1	1	4	2	0	12
CD_67	0	0	7	0	3	0	0	0	0	0	1
CD_68	1	5	348	3	27	0	0	0	0	0	15
CD_78	0	0	3	1	0	0	0	0	0	1	0
CD_79	0	1	5	0	0	0	0	0	0	0	4
CD_80	0	0	0	0	0	0	0	0	0	0	1
CDFG_53	0	0	0	10	0	0	0	0	0	0	1
CDFG_54	0	0	0	29	0	0	1	0	0	0	3
CDFG_55	2	1	0	12	0	0	0	0	0	0	4
CDFG_56	5	7	0	41	3	0	3	0	2	4	12
CDFG_70	0	20	14	43	0	0	0	3	3	0	6
CDFG_71	0	2	11	25	0	0	0	0	49	0	4
HF_41	0	0	0	0	0	0	0	0	0	1	0
HF_43	0	0	3	1	0	0	0	0	0	0	2
HF_44	0	0	8	0	0	0	0	0	0	0	5
HF_57	1	2	2	0	0	0	0	0	2	3	1
HF_58	3	1	228	26	0	3	3	4	4	4	144
HF_59	0	0	142	2	0	0	1	0	0	1	30
HF_60	0	0	7	1	0	0	0	0	0	0	3
HF_73	0	0	7	0	6	0	2	3	0	0	4
HFFG_45	0	0	0	2	0	0	1	0	0	0	301
HFFG_46	0	0	0	41	0	0	0	0	0	0	4
HFFG_47	0	0	0	0	0	0	0	0	0	0	0
HFFG_48	1	0	0	3	0	0	0	0	0	2	9
HFFG_61	0	0	8	0	0	0	0	0	0	0	184
HFFG_62	0	0	2	1	0	0	0	0	0	0	0
HFFG_75	0	0	2	0	0	0	0	1	1	0	1
HFFG_76	0	17	9	10	0	3	3	2	39	3	17

Kingdom	Bacteria	Bacteria	Bacteria	Bacteria	Bacteria	Bacteria	Bacteria	Bacteria	Bacteria	Bacteria	Bacteria
Phylum	Firmicutes	Firmicutes	Firmicutes	Firmicutes	Bacteroidetes	Firmicutes	Firmicutes	Firmicutes	Firmicutes	Firmicutes	Firmicutes
Class	Clostridia	Clostridia	Clostridia	Clostridia	Bacteroidia	Clostridia	Clostridia	Clostridia	Clostridia	Clostridia	Clostridia
Order	Clostridiales	Clostridiales	Clostridiales	Clostridiales	Bacteroidales	Clostridiales	Clostridiales	Clostridiales	Clostridiales	Clostridiales	Clostridiales
Family	Lachnospiraceae	Lachnospiraceae	Lachnospiraceae	Eubacteriaceae	Porphyromonadaceae	Ruminococcaceae	Lachnospiraceae	Ruminococcaceae	Lachnospiraceae	Lachnospiraceae	Clostridiaceae_
Genus	Acetatifactor	Blautia	Clostridium_XlVa	Eubacterium	Barnesiella	Saccharofermentans	Clostridium_XlVa	Clostridium_IV	Anaerostipes	Clostridium_XlVb	Alkaliphilus
#NAME	ZOTU_0087	ZOTU_0088	ZOTU_0089	ZOTU_0090	ZOTU_0091	ZOTU_0092	ZOTU_0093	ZOTU_0094	ZOTU_0095	ZOTU_0096	ZOTU_0097
CD_49	0	0	2	0	4	1	0	3	0	0	1
CD_50	0	1	0	0	6	0	4	3	1	2	0
CD_52	0	2	5	0	20	0	1	24	0	0	0
CD_65	0	0	23	1	10	1	4	5	0	0	1
CD_67	0	0	0	0	0	0	0	1	0	0	1
CD_68	0	0	44	0	7	0	0	4	0	0	0
CD_78	0	0	0	0	2	0	1	0	0	0	0
CD_79	0	0	1	0	12	0	1	1	0	0	0
CD_80	0	3	0	0	0	0	0	0	0	0	0
CDFG_53	0	0	0	0	1	0	0	0	0	0	0
CDFG_54	3	0	0	0	14	0	4	5	10	3	0
CDFG_55	1	1	0	0	39	0	1	3	7	1	0
CDFG_56	7	2	4	4	9	4	1	7	20	0	1
CDFG_70	33	11	0	1	12	0	29	3	26	2	0
CDFG_71	3	4	4	0	2	0	10	8	8	5	0
HF_41	0	0	0	0	0	0	0	0	0	0	0
HF_43	0	1	2	0	0	1	2	0	0	0	0
HF_44	0	0	0	0	0	0	0	0	0	1	0
HF_57	0	0	1	3	1	1	0	1	0	0	2
HF_58	1	0	3	2	0	2	24	0	0	8	5
HF_59	0	0	1	2	0	0	8	0	0	1	0
HF_60	0	1	1	0	6	0	0	0	0	0	0
HF_73	0	0	4	0	2	4	0	3	0	0	2
HFFG_45	0	0	0	0	1	0	0	3	3	0	0
HFFG_46	0	0	0	0	0	0	7	19	6	0	0
HFFG_47	0	0	0	0	0	0	0	0	0	0	0
HFFG_48	0	1	0	0	1	1	0	3	0	0	0
HFFG_61	0	0	0	0	0	0	0	1	5	0	0
HFFG_62	0	0	1	0	0	0	2	2	3	0	0
HFFG_75	0	0	0	0	0	0	0	0	0	0	0
HFFG_76	0	0	4	7	11	1	5	12	0	1	4

Kingdom	Bacteria	Bacteria	Bacteria	Bacteria	Bacteria	Bacteria	Bacteria	Bacteria	Bacteria	Bacteria	Bacteria
Phylum	Firmicutes	Firmicutes	Firmicutes	Firmicutes	Firmicutes	Firmicutes	Firmicutes	Firmicutes	Actinobacteria	Firmicutes	Firmicutes
Class	Clostridia	Clostridia	Clostridia	Clostridia	Clostridia	Clostridia	Clostridia	Clostridia	Actinobacteria	Clostridia	Clostridia
Order	Clostridiales	Clostridiales	Clostridiales	Clostridiales	Clostridiales	Clostridiales	Clostridiales	Clostridiales	Actinomycetales	Clostridiales	Clostridiales
Family	Ruminococcaceae	Lachnospiraceae	Ruminococcaceae	Lachnospiraceae	Lachnospiraceae	Lachnospiraceae	Lachnospiraceae	Lachnospiraceae	Mycobacteriaceae	Ruminococcaceae	Ruminococcaceae
Genus	Clostridium_IV	Clostridium_XlVa	Pseudoflavonifractor	Acetatifactor	Clostridium_XlVa	Roseburia	Marvinbryantia	Clostridium_XlVa	Mycobacterium	Clostridium_IV	Flavonifractor
#NAME	ZOTU_0098	ZOTU_0099	ZOTU_0100	ZOTU_0101	ZOTU_0102	ZOTU_0103	ZOTU_0104	ZOTU_0105	ZOTU_0106	ZOTU_0107	ZOTU_0108
CD_49	0	0	1	8	0	0	0	0	0	1	0
CD_50	0	0	0	12	2	1	0	0	0	1	0
CD_52	0	0	0	32	1	2	0	0	0	0	2
CD_65	0	24	1	3	0	1	0	0	0	1	0
CD_67	0	0	0	0	0	0	0	0	0	0	0
CD_68	0	0	2	20	0	1	0	0	0	0	0
CD_78	0	2	0	0	0	0	0	0	0	0	0
CD_79	0	1	0	1	1	1	0	0	0	0	0
CD_80	0	0	0	0	0	0	0	0	0	0	0
CDFG_53	0	12	0	2	0	0	0	0	0	0	0
CDFG_54	1	0	1	1	0	3	0	0	0	0	7
CDFG_55	0	8	3	4	0	0	0	0	1	0	0
CDFG_56	0	9	1	9	0	0	0	0	1	0	7
CDFG_70	0	1	2	4	2	27	1	0	0	0	15
CDFG_71	0	0	0	2	0	6	1	0	0	0	4
HF_41	0	0	0	0	1	0	0	0	0	0	0
HF_43	0	0	0	3	2	0	0	0	0	0	0
HF_44	0	0	0	4	0	0	0	0	0	0	0
HF_57	0	1	0	1	1	0	0	0	1	0	0
HF_58	0	0	0	154	1	1	0	0	0	1	0
HF_59	0	0	0	60	0	0	0	0	3	0	0
HF_60	0	0	0	4	0	0	0	0	0	0	0
HF_73	0	1	0	5	0	0	0	0	5	1	0
HFFG_45	0	0	0	337	0	0	0	0	2	0	0
HFFG_46	0	0	0	2	0	0	0	0	32	0	0
HFFG_47	0	0	0	0	0	0	0	0	0	0	0
HFFG_48	0	1	0	15	0	0	0	0	0	0	0
HFFG_61	0	2	0	138	0	0	0	0	0	0	0
HFFG_62	0	1	0	1	0	0	0	0	0	0	0
HFFG_75	0	0	0	3	0	0	0	0	0	0	0
HFFG_76	0	3	3	7	2	1	0	0	2	0	0

Kingdom	Bacteria	Bacteria	Bacteria	Bacteria	Bacteria	Bacteria	Bacteria	Bacteria	Bacteria	Bacteria	Bacteria
Phylum	Firmicutes	Firmicutes	Firmicutes	Firmicutes	Firmicutes	Firmicutes	Firmicutes	Firmicutes	Firmicutes	Firmicutes	Firmicutes
Class	Clostridia	Clostridia	Clostridia	Clostridia	Clostridia	Clostridia	Clostridia	Clostridia	Bacilli	Clostridia	Clostridia
Order	Clostridiales	Clostridiales	Clostridiales	Clostridiales	Clostridiales	Clostridiales	Clostridiales	Clostridiales	Bacillales	Clostridiales	Clostridiales
Family	Clostridiaceae_	Lachnospiraceae	Ruminococcaceae	Lachnospiraceae	Lachnospiraceae	Lachnospiraceae	Lachnospiraceae	Lachnospiraceae	Bacillaceae_	Lachnospiraceae	Clostridiales_Incertae_Sedis_XII
Genus	Alkaliphilus	Acetatifactor	Oscillibacter	Blautia	Clostridium_XlVa	Clostridium_XlVa	Clostridium_XlVa	Clostridium_XlVa	Bacillus	Clostridium_XlVa	Guggenheimella
#NAME	ZOTU_0109	ZOTU_0110	ZOTU_0111	ZOTU_0112	ZOTU_0113	ZOTU_0114	ZOTU_0115	ZOTU_0116	ZOTU_0117	ZOTU_0118	ZOTU_0119
CD_49	1	0	0	0	0	16	0	0	0	0	1
CD_50	0	0	0	0	2	1	1	0	0	0	0
CD_52	0	0	0	88	3	6	2	0	0	0	0
CD_65	0	0	1	0	2	1	0	1	0	0	0
CD_67	0	0	0	0	1	0	0	0	1	0	0
CD_68	0	0	0	0	2	2	0	0	0	0	0
CD_78	1	0	0	0	0	0	0	0	0	0	0
CD_79	0	0	0	0	0	0	1	0	0	0	0
CD_80	0	0	0	0	0	0	1	0	0	0	0
CDFG_53	0	0	0	17	1	0	0	5	0	0	0
CDFG_54	0	0	0	24	0	0	0	3	0	0	0
CDFG_55	0	0	0	40	1	3	1	7	0	0	0
CDFG_56	0	0	0	23	2	10	0	21	0	0	0
CDFG_70	2	0	0	169	27	2	0	221	0	0	0
CDFG_71	0	0	0	30	14	10	0	5	0	0	0
HF_41	0	0	0	0	0	0	0	0	0	0	0
HF_43	0	0	3	0	0	0	0	1	13	0	0
HF_44	0	0	0	1	0	0	0	0	0	0	0
HF_57	0	0	0	0	0	0	0	0	0	0	1
HF_58	4	0	0	0	1	0	12	0	0	0	0
HF_59	2	0	0	0	0	0	3	0	0	0	0
HF_60	0	0	0	4	0	0	0	2	0	0	0
HF_73	0	0	0	0	1	0	0	0	0	0	0
HFFG_45	2	0	0	5	0	7	0	17	0	0	0
HFFG_46	0	0	0	0	6	25	0	11	0	0	0
HFFG_47	0	0	0	0	0	0	0	0	0	0	0
HFFG_48	0	0	0	0	0	3	2	6	0	0	0
HFFG_61	0	0	0	3	0	0	0	0	0	0	0
HFFG_62	0	0	0	0	0	1	0	0	0	0	0
HFFG_75	0	0	0	0	0	0	2	1	0	0	0
HFFG_76	0	0	0	8	1	4	0	0	0	0	0

Kingdom	Bacteria	Bacteria	Bacteria	Bacteria	Bacteria	Bacteria	Bacteria	Bacteria	Bacteria	Bacteria
Phylum	Firmicutes	Firmicutes	Firmicutes	Firmicutes	Firmicutes	Firmicutes	Firmicutes	Firmicutes	Firmicutes	Firmicutes
Class	Clostridia	Clostridia	Clostridia	Clostridia	Clostridia	Bacilli	Clostridia	Clostridia	Clostridia	Clostridia
Order	Clostridiales	Clostridiales	Clostridiales	Clostridiales	Clostridiales	Bacillales	Clostridiales	Clostridiales	Clostridiales	Clostridiales
Family	Ruminococcaceae	Lachnospiraceae	Lachnospiraceae	Lachnospiraceae	Lachnospiraceae	Paenibacillaceae_	Clostridiaceae_	Lachnospiraceae	Lachnospiraceae	Lachnospiraceae
Genus	Clostridium_IV	Clostridium_XlVa	Lachnospiracea_incertae_sedis	Clostridium_XlVa	Clostridium_XlVa	Brevibacillus	Clostridium_sensu_stricto	Clostridium_XlVa	Clostridium_XlVa	Acetatifactor
#NAME	ZOTU_0120	ZOTU_0121	ZOTU_0122	ZOTU_0123	ZOTU_0124	ZOTU_0125	ZOTU_0126	ZOTU_0127	ZOTU_0128	ZOTU_0129
CD_49	0	2	4	0	1	0	1	0	0	0
CD_50	0	19	1	1	0	0	6	0	0	1
CD_52	0	18	3	0	5	0	0	0	0	7
CD_65	0	35	0	0	4	0	5	2	0	4
CD_67	0	5	0	0	0	1	0	0	0	0
CD_68	2	59	0	0	4	0	5	1	0	0
CD_78	0	3	0	0	0	0	0	0	0	0
CD_79	0	21	0	0	0	0	5	0	0	0
CD_80	0	0	0	0	0	0	0	0	0	0
CDFG_53	0	4	0	3	1	0	2	0	0	2
CDFG_54	1	17	1	3	0	0	2	0	0	7
CDFG_55	0	4	0	27	0	0	5	0	0	2
CDFG_56	0	7	1	3	0	0	3	1	0	23
CDFG_70	5	15	0	10	1	0	0	0	0	13
CDFG_71	2	17	1	2	2	0	0	2	0	0
HF_41	0	0	0	0	0	0	0	0	0	0
HF_43	0	3	0	0	0	0	1	0	0	0
HF_44	0	0	3	0	0	0	3	0	0	0
HF_57	0	6	0	1	0	1	2	1	0	0
HF_58	0	15	11	6	3	0	41	1	0	0
HF_59	0	2	19	0	1	0	4	0	0	0
HF_60	0	4	0	2	0	0	1	0	0	2
HF_73	0	4	0	0	2	0	3	0	0	0
HFFG_45	0	12	0	3	0	0	1	0	0	6
HFFG_46	0	54	0	5	4	0	19	1	0	3
HFFG_47	0	0	0	0	0	0	0	0	0	0
HFFG_48	0	0	0	0	0	0	1	0	0	10
HFFG_61	0	8	0	0	0	0	1	0	0	17
HFFG_62	0	4	0	0	0	0	0	0	0	12
HFFG_75	0	0	0	0	0	0	0	0	0	0
HFFG_76	0	35	0	1	1	0	2	1	0	7

Kingdom	Bacteria	Bacteria	Bacteria	Bacteria	Bacteria	Bacteria	Bacteria	Bacteria	Bacteria	Bacteria
Phylum	Firmicutes	Firmicutes	Firmicutes	Firmicutes	Firmicutes	Firmicutes	Firmicutes	Firmicutes	Firmicutes	Firmicutes
Class	Clostridia	Clostridia	Clostridia	Clostridia	Erysipelotrichia	Clostridia	Clostridia	Erysipelotrichia	Clostridia	Clostridia
Order	Clostridiales	Clostridiales	Clostridiales	Clostridiales	Erysipelotrichales	Clostridiales	Clostridiales	Erysipelotrichales	Clostridiales	Clostridiales
Family	Lachnospiraceae	Eubacteriaceae	Ruminococcaceae	Lachnospiraceae	Erysipelotrichaceae	Lachnospiraceae	Lachnospiraceae	Erysipelotrichaceae	Lachnospiraceae	Ruminococcaceae
Genus	Clostridium_XlVa	Eubacterium	Clostridium_IV	Acetatifactor	Coprobacillus	Clostridium_XlVa	Acetatifactor	Erysipelotrichaceae_incertae_sedis	Dorea	Clostridium_IV
#NAME	ZOTU_0130	ZOTU_0131	ZOTU_0132	ZOTU_0133	ZOTU_0134	ZOTU_0135	ZOTU_0136	ZOTU_0137	ZOTU_0138	ZOTU_0139
CD_49	0	0	0	0	0	0	0	0	0	0
CD_50	0	2	0	0	0	0	0	0	0	0
CD_52	1	0	0	0	0	0	0	1	0	8
CD_65	0	6	5	0	1	0	5	0	0	9
CD_67	0	0	0	0	1	0	0	0	0	0
CD_68	2	0	2	0	0	0	111	0	0	0
CD_78	0	0	1	0	0	0	0	0	0	0
CD_79	1	0	1	0	0	0	4	0	0	0
CD_80	0	0	0	0	3	0	0	1	0	0
CDFG_53	0	2	0	0	0	0	0	0	9	0
CDFG_54	2	0	1	2	0	0	0	0	20	12
CDFG_55	0	0	0	0	0	1	0	2	0	0
CDFG_56	0	0	4	0	0	0	0	1	20	5
CDFG_70	3	0	1	15	0	0	3	0	30	89
CDFG_71	5	0	1	1	0	1	2	0	22	0
HF_41	0	1	0	0	0	0	0	0	0	0
HF_43	0	0	0	0	0	0	0	0	0	0
HF_44	0	0	0	0	0	0	0	0	0	0
HF_57	0	0	1	0	2	2	1	0	0	0
HF_58	6	1	3	0	4	0	61	1	0	4
HF_59	0	0	1	0	2	0	39	0	0	0
HF_60	0	0	0	0	0	0	3	0	0	0
HF_73	0	1	0	1	4	0	1	4	0	1
HFFG_45	2	0	0	0	0	0	1	0	8	1
HFFG_46	4	0	0	0	0	0	0	0	51	5
HFFG_47	0	0	0	0	0	0	0	0	0	0
HFFG_48	0	0	0	0	0	0	0	3	0	0
HFFG_61	0	0	0	0	0	1	3	4	2	3
HFFG_62	0	0	0	0	0	0	0	0	1	2
HFFG_75	0	0	0	0	0	0	0	0	0	0
HFFG_76	4	4	9	0	4	3	3	1	18	5

Kingdom	Bacteria	Bacteria	Bacteria	Bacteria	Bacteria	Bacteria	Bacteria	Bacteria	Bacteria	Bacteria
Phylum	Firmicutes	Firmicutes	Firmicutes	Firmicutes	Firmicutes	Firmicutes	Firmicutes	Firmicutes	Firmicutes	Firmicutes
Class	Erysipelotrichia	Clostridia	Clostridia	Clostridia	Clostridia	Clostridia	Clostridia	Clostridia	Clostridia	Clostridia
Order	Erysipelotrichales	Clostridiales	Clostridiales	Clostridiales	Clostridiales	Clostridiales	Clostridiales	Clostridiales	Clostridiales	Clostridiales
Family	Erysipelotrichaceae	Lachnospiraceae	Clostridiaceae_	Lachnospiraceae	Ruminococcaceae	Clostridiaceae_	Ruminococcaceae	Ruminococcaceae	Lachnospiraceae	Lachnospiraceae
Genus	Erysipelotrichaceae_incertae_sedis	Eisenbergiella	Alkaliphilus	Clostridium_XlVa	Intestinimonas	Alkaliphilus	Ruminococcus	Clostridium_IV	Anaerosporobacter	Butyrivibrio
#NAME	ZOTU_0140	ZOTU_0141	ZOTU_0142	ZOTU_0143	ZOTU_0144	ZOTU_0145	ZOTU_0146	ZOTU_0147	ZOTU_0148	ZOTU_0149
CD_49	0	0	0	5	0	0	0	0	0	8
CD_50	0	2	0	0	0	0	0	0	0	0
CD_52	1	2	0	11	0	0	1	0	0	12
CD_65	0	0	0	1	1	0	0	0	0	0
CD_67	0	0	0	0	0	0	0	0	0	1
CD_68	0	6	0	4	0	0	0	1	0	2
CD_78	0	0	0	0	0	0	0	0	0	0
CD_79	0	2	0	6	0	0	1	0	0	0
CD_80	0	0	0	0	0	0	0	0	0	0
CDFG_53	0	0	0	2	0	1	0	0	0	2
CDFG_54	0	1	0	2	0	1	0	1	2	1
CDFG_55	0	3	0	0	2	24	1	0	2	0
CDFG_56	0	2	0	2	0	7	0	2	5	9
CDFG_70	2	14	0	11	7	10	8	44	3	2
CDFG_71	0	0	0	19	0	3	0	38	1	0
HF_41	0	0	0	0	0	0	0	0	0	0
HF_43	0	0	0	0	0	0	0	0	0	0
HF_44	0	0	0	1	0	0	0	0	0	0
HF_57	0	0	0	0	0	0	0	0	0	2
HF_58	0	0	0	7	0	0	0	0	1	0
HF_59	1	0	0	2	0	0	0	0	0	0
HF_60	0	0	0	0	0	0	0	0	0	0
HF_73	1	0	0	1	1	0	0	0	0	0
HFFG_45	0	5	0	1	0	0	0	0	0	25
HFFG_46	0	1	0	3	0	0	0	1	0	16
HFFG_47	0	0	0	0	0	0	0	0	0	0
HFFG_48	0	0	0	0	0	0	0	0	0	2
HFFG_61	1	1	0	0	0	0	0	0	0	24
HFFG_62	0	0	0	0	0	0	0	0	0	11
HFFG_75	0	0	0	0	0	0	0	0	1	0
HFFG_76	0	3	0	1	6	0	0	1	3	6

Kingdom	Bacteria	Bacteria	Bacteria	Bacteria	Bacteria	Bacteria	Bacteria	Bacteria	Bacteria	Bacteria	Bacteria
Phylum	Firmicutes	Firmicutes	Firmicutes	Firmicutes	Firmicutes	Actinobacteria	Firmicutes	Firmicutes	Bacteroidetes	Firmicutes	Firmicutes
Class	Clostridia	Clostridia	Clostridia	Clostridia	Clostridia	Actinobacteria	Clostridia	Clostridia	Bacteroidia	Clostridia	Clostridia
Order	Clostridiales	Clostridiales	Clostridiales	Clostridiales	Clostridiales	Coriobacteriales	Clostridiales	Clostridiales	Bacteroidales	Clostridiales	Clostridiales
Family	Clostridiaceae_	Clostridiaceae_	Eubacteriaceae	Lachnospiraceae	Ruminococcaceae	Coriobacteriaceae	Proteinivoraceae	Lachnospiraceae	Porphyromonadaceae	Lachnospiraceae	Lachnospiraceae
Genus	Alkaliphilus	Alkaliphilus	Eubacterium	Clostridium_XlVa	Flavonifractor	Adlercreutzia	Proteinivorax	Lachnospiracea_incertae_sedis	Barnesiella	Clostridium_XlVa	Clostridium_XlVa
#NAME	ZOTU_0150	ZOTU_0151	ZOTU_0152	ZOTU_0153	ZOTU_0154	ZOTU_0155	ZOTU_0156	ZOTU_0157	ZOTU_0158	ZOTU_0159	ZOTU_0160
CD_49	0	0	0	0	0	0	0	0	0	0	2
CD_50	0	0	0	0	1	2	1	0	0	0	0
CD_52	0	0	1	0	0	2	0	0	0	0	0
CD_65	0	0	0	0	4	1	0	0	1	0	0
CD_67	0	0	0	0	0	0	0	0	0	0	0
CD_68	0	0	0	0	0	2	0	0	0	0	0
CD_78	0	0	0	0	0	0	0	0	0	0	0
CD_79	0	0	0	0	0	0	0	1	0	0	0
CD_80	0	0	0	0	0	0	0	0	0	0	0
CDFG_53	0	0	0	0	0	3	0	0	0	0	0
CDFG_54	0	0	0	1	0	1	0	0	0	0	0
CDFG_55	0	0	2	0	0	12	0	0	0	0	0
CDFG_56	0	0	0	0	0	2	0	0	0	3	0
CDFG_70	0	0	1	0	0	0	0	7	0	0	5
CDFG_71	0	0	0	0	0	0	0	2	2	0	1
HF_41	0	0	0	0	0	0	0	0	0	0	0
HF_43	0	0	0	0	0	0	0	0	0	0	0
HF_44	0	0	0	0	0	0	0	0	0	0	0
HF_57	0	0	1	0	0	0	3	0	0	0	0
HF_58	0	0	0	0	2	0	1	0	0	0	0
HF_59	0	0	0	0	0	0	0	0	0	0	0
HF_60	0	0	0	0	0	1	0	0	0	0	0
HF_73	0	0	2	1	0	0	2	0	0	0	0
HFFG_45	0	0	0	0	0	0	1	0	0	0	0
HFFG_46	0	0	0	0	0	0	0	0	0	0	14
HFFG_47	0	0	0	0	0	0	0	0	0	0	0
HFFG_48	0	0	0	0	0	0	0	0	0	0	0
HFFG_61	0	0	0	0	0	0	0	0	0	0	0
HFFG_62	0	0	0	0	0	0	0	0	0	1	0
HFFG_75	0	0	0	0	0	0	0	0	0	0	0
HFFG_76	0	0	1	1	7	3	0	1	0	0	4

Kingdom	Bacteria	Bacteria	Bacteria	Bacteria	Bacteria	Bacteria	Bacteria	Bacteria	Bacteria	Bacteria
Phylum	Firmicutes	Firmicutes	Firmicutes	Firmicutes	Firmicutes	Firmicutes	Firmicutes	Firmicutes	Firmicutes	Firmicutes
Class	Clostridia	Clostridia	Clostridia	Clostridia	Clostridia	Clostridia	Clostridia	Clostridia	Clostridia	Clostridia
Order	Clostridiales	Clostridiales	Clostridiales	Clostridiales	Clostridiales	Clostridiales	Clostridiales	Clostridiales	Clostridiales	Clostridiales
Family	Lachnospiraceae	Lachnospiraceae	Lachnospiraceae	Lachnospiraceae	Clostridiaceae_	Lachnospiraceae	Lachnospiraceae	Ruminococcaceae	Lachnospiraceae	Lachnospiraceae
Genus	Clostridium_XlVa	Butyrivibrio	Clostridium_XlVa	Lachnospiracea_incertae_sedis	Geosporobacter	Clostridium_XlVa	Clostridium_XlVa	Anaerotruncus	Clostridium_XlVa	Clostridium_XlVa
#NAME	ZOTU_0161	ZOTU_0162	ZOTU_0163	ZOTU_0164	ZOTU_0165	ZOTU_0166	ZOTU_0167	ZOTU_0168	ZOTU_0169	ZOTU_0170
CD_49	0	2	0	0	0	0	0	0	0	1
CD_50	6	0	1	0	1	0	0	0	0	0
CD_52	16	6	0	0	0	2	0	0	0	0
CD_65	1	3	0	0	0	0	0	0	0	0
CD_67	1	1	0	0	0	0	0	0	0	0
CD_68	8	1	0	0	2	1	0	1	2	1
CD_78	0	0	0	0	0	0	0	0	0	0
CD_79	0	1	0	0	0	0	0	0	0	0
CD_80	0	1	0	0	0	0	0	0	0	0
CDFG_53	3	1	0	0	0	0	0	0	0	0
CDFG_54	3	0	1	0	0	2	0	0	0	0
CDFG_55	0	1	0	0	2	0	0	0	0	0
CDFG_56	0	6	0	0	0	0	0	0	0	5
CDFG_70	16	0	1	0	2	1	1	0	16	0
CDFG_71	3	0	1	0	0	2	0	0	4	1
HF_41	0	0	0	0	0	0	0	0	0	0
HF_43	0	0	0	0	0	3	0	0	0	0
HF_44	0	0	0	0	0	0	0	0	0	0
HF_57	0	0	0	0	2	0	0	0	0	0
HF_58	59	0	0	0	0	3	0	0	0	0
HF_59	1	0	0	0	0	1	0	0	0	0
HF_60	3	0	0	0	0	0	0	0	0	0
HF_73	0	0	0	0	1	0	0	0	0	1
HFFG_45	0	11	0	0	0	0	0	0	0	0
HFFG_46	0	8	0	0	0	0	0	0	0	0
HFFG_47	0	0	0	0	0	0	0	0	0	0
HFFG_48	0	0	0	0	0	0	0	0	0	0
HFFG_61	0	8	0	0	0	0	0	0	0	0
HFFG_62	0	6	0	0	0	0	0	2	0	1
HFFG_75	0	0	0	0	0	0	0	0	0	0
HFFG_76	1	5	3	0	2	0	1	3	0	0

Kingdom	Bacteria	Bacteria	Bacteria	Bacteria	Bacteria	Bacteria	Bacteria	Bacteria	Bacteria	Bacteria	Bacteria
Phylum	Tenericutes	Firmicutes	Firmicutes	Firmicutes	Firmicutes	Firmicutes	Firmicutes	Firmicutes	Firmicutes	Tenericutes	Firmicutes
Class	Mollicutes	Clostridia	Clostridia	Clostridia	Clostridia	Clostridia	Clostridia	Clostridia	Clostridia	Mollicutes	Clostridia
Order	Anaeroplasmatales	Clostridiales	Clostridiales	Clostridiales	Clostridiales	Clostridiales	Clostridiales	Clostridiales	Clostridiales	Entomoplasmatales	Clostridiales
Family	Anaeroplasmataceae	Clostridiaceae_	Ruminococcaceae	Lachnospiraceae	Lachnospiraceae	Ruminococcaceae	Lachnospiraceae	Lachnospiraceae	Ruminococcaceae	Spiroplasmataceae	Lachnospiraceae
Genus	Anaeroplasma	Alkaliphilus	Oscillibacter	Acetatifactor	Blautia	Clostridium_IV	Dorea	Clostridium_XlVa	Oscillibacter	Spiroplasma	Acetatifactor
#NAME	ZOTU_0171	ZOTU_0172	ZOTU_0173	ZOTU_0174	ZOTU_0175	ZOTU_0176	ZOTU_0177	ZOTU_0178	ZOTU_0179	ZOTU_0180	ZOTU_0181
CD_49	0	0	0	0	0	0	0	0	0	0	0
CD_50	0	0	0	1	0	0	0	2	0	0	0
CD_52	0	0	0	2	0	0	0	2	0	0	0
CD_65	0	0	0	0	0	0	0	12	1	1	3
CD_67	0	0	0	0	0	0	0	2	0	0	0
CD_68	0	0	0	6	0	0	0	1	1	0	2
CD_78	0	0	0	0	0	1	0	0	0	0	0
CD_79	0	0	1	1	0	0	0	0	0	0	1
CD_80	0	0	0	0	0	0	0	0	0	0	0
CDFG_53	0	0	0	1	0	0	5	0	1	0	0
CDFG_54	0	0	0	10	0	0	9	0	0	0	0
CDFG_55	0	0	0	1	0	0	1	1	0	0	0
CDFG_56	0	0	0	1	0	0	12	0	0	0	0
CDFG_70	0	0	0	37	0	4	10	1	0	0	1
CDFG_71	0	0	0	18	0	1	19	0	0	0	0
HF_41	0	0	0	0	0	0	0	0	0	0	0
HF_43	0	0	0	0	0	0	0	0	0	0	0
HF_44	0	0	0	0	0	0	0	0	0	0	0
HF_57	0	0	0	0	0	0	0	0	0	0	0
HF_58	0	0	1	0	0	0	0	0	0	3	0
HF_59	0	0	0	0	0	0	0	0	0	0	2
HF_60	0	0	0	0	0	0	0	0	0	0	0
HF_73	0	0	0	3	0	0	0	0	0	0	0
HFFG_45	0	0	0	1	0	0	4	0	0	0	0
HFFG_46	0	0	0	0	0	0	25	0	0	0	0
HFFG_47	0	0	0	0	0	0	0	0	0	0	0
HFFG_48	0	0	0	0	0	0	0	0	0	0	0
HFFG_61	0	0	0	0	0	0	2	0	0	1	0
HFFG_62	0	0	0	0	0	0	4	0	0	0	0
HFFG_75	0	0	0	0	0	0	0	0	0	0	0
HFFG_76	0	0	0	1	0	1	10	0	6	2	2

Kingdom	Bacteria	Bacteria	Bacteria	Bacteria	Bacteria	Bacteria	Bacteria	Bacteria	Bacteria	Bacteria	Bacteria
Phylum	Firmicutes	Firmicutes	Firmicutes	Firmicutes	Firmicutes	Firmicutes	Firmicutes	Firmicutes	Actinobacteria	Firmicutes	Firmicutes
Class	Clostridia	Clostridia	Clostridia	Clostridia	Clostridia	Clostridia	Clostridia	Clostridia	Actinobacteria	Clostridia	Clostridia
Order	Clostridiales	Clostridiales	Clostridiales	Clostridiales	Clostridiales	Clostridiales	Halanaerobiales	Clostridiales	Actinomycetales	Clostridiales	Clostridiales
Family	Lachnospiraceae	Ruminococcaceae	Lachnospiraceae	Lachnospiraceae	Lachnospiraceae	Lachnospiraceae	Halanaerobiaceae	Ruminococcaceae	Streptomycetaceae	Lachnospiraceae	Lachnospiraceae
Genus	Clostridium_XlVa	Butyricicoccus	Clostridium_XlVa	Clostridium_XlVa	Lachnospira	Clostridium_XlVa	Halanaerobium	Clostridium_IV	Streptomyces	Ruminococcus	Roseburia
#NAME	ZOTU_0182	ZOTU_0183	ZOTU_0184	ZOTU_0185	ZOTU_0186	ZOTU_0187	ZOTU_0188	ZOTU_0189	ZOTU_0190	ZOTU_0191	ZOTU_0192
CD_49	0	0	2	0	0	0	1	0	0	1	0
CD_50	1	0	1	4	0	1	1	0	0	2	0
CD_52	0	0	9	9	0	0	1	3	0	11	0
CD_65	0	0	1	0	0	0	0	1	0	14	2
CD_67	0	0	0	0	0	0	0	0	0	0	0
CD_68	0	0	1	6	0	0	0	0	0	7	4
CD_78	0	0	0	0	0	0	0	0	0	0	0
CD_79	0	1	0	0	0	0	0	0	0	0	0
CD_80	2	0	0	0	0	0	0	0	0	0	0
CDFG_53	0	0	0	0	0	0	0	0	0	0	0
CDFG_54	0	0	1	3	0	0	1	0	2	0	0
CDFG_55	0	0	0	0	0	0	1	0	0	0	0
CDFG_56	3	0	0	0	0	1	144	2	0	0	0
CDFG_70	0	2	27	18	0	2	0	10	28	15	2
CDFG_71	0	0	6	11	0	0	0	6	2	8	0
HF_41	0	0	0	0	0	0	0	0	0	0	0
HF_43	0	0	0	0	0	0	0	0	0	0	0
HF_44	0	0	0	0	0	0	0	0	1	0	0
HF_57	1	0	0	0	0	0	0	0	0	1	1
HF_58	1	0	32	28	0	3	0	0	6	3	0
HF_59	1	0	1	3	0	0	0	0	8	0	0
HF_60	0	0	0	0	0	0	0	0	1	0	0
HF_73	2	1	0	0	0	0	0	0	0	0	0
HFFG_45	0	0	0	0	0	2	0	0	0	0	0
HFFG_46	0	0	0	0	0	1	0	0	0	0	0
HFFG_47	0	0	0	0	0	0	0	0	0	0	0
HFFG_48	1	0	0	0	0	0	1	0	0	0	0
HFFG_61	0	0	0	0	0	0	2	0	10	1	0
HFFG_62	0	0	0	0	0	0	127	0	1	1	0
HFFG_75	0	0	0	0	0	0	0	0	0	0	0
HFFG_76	0	1	1	0	0	5	1	5	3	0	0

Kingdom	Bacteria	Bacteria	Bacteria	Bacteria	Bacteria	Bacteria	Bacteria	Bacteria	Bacteria	Bacteria	Bacteria
Phylum	Firmicutes	Firmicutes	Firmicutes	Firmicutes	Firmicutes	Firmicutes	Firmicutes	Firmicutes	Firmicutes	Firmicutes	Firmicutes
Class	Clostridia	Clostridia	Clostridia	Clostridia	Clostridia	Clostridia	Clostridia	Clostridia	Clostridia	Clostridia	Clostridia
Order	Clostridiales	Clostridiales	Clostridiales	Clostridiales	Clostridiales	Clostridiales	Clostridiales	Clostridiales	Clostridiales	Clostridiales	Clostridiales
Family	Lachnospiraceae	Ruminococcaceae	Ruminococcaceae	Ruminococcaceae	Lachnospiraceae	Lachnospiraceae	Ruminococcaceae	Lachnospiraceae	Lachnospiraceae	Lachnospiraceae	Lachnospiraceae
Genus	Clostridium_XlVa	Clostridium_III	Oscillibacter	Anaerotruncus	Clostridium_XlVa	Clostridium_XlVa	Intestinimonas	Clostridium_XlVa	Acetatifactor	Acetatifactor	Clostridium_XlVa
#NAME	ZOTU_0193	ZOTU_0194	ZOTU_0195	ZOTU_0196	ZOTU_0197	ZOTU_0198	ZOTU_0199	ZOTU_0200	ZOTU_0201	ZOTU_0202	ZOTU_0203
CD_49	2	0	0	0	0	0	0	0	4	2	0
CD_50	3	0	2	0	0	0	0	0	0	1	0
CD_52	7	0	0	0	0	1	0	0	0	0	0
CD_65	4	0	0	0	0	2	2	1	1	0	0
CD_67	1	0	0	0	0	0	0	0	0	0	0
CD_68	3	0	0	0	0	1	0	0	0	0	0
CD_78	0	0	0	0	0	0	0	0	0	0	0
CD_79	0	0	0	0	0	0	0	0	1	0	0
CD_80	0	0	0	0	0	0	0	0	0	0	0
CDFG_53	0	0	0	0	0	0	0	0	0	0	1
CDFG_54	1	3	0	0	0	0	0	1	0	0	0
CDFG_55	0	1	0	0	0	1	0	1	0	0	0
CDFG_56	0	0	0	0	0	1	0	0	1	0	0
CDFG_70	20	10	0	0	0	1	5	13	8	4	0
CDFG_71	1	0	0	0	0	0	2	8	5	3	0
HF_41	0	0	0	0	0	0	0	0	0	0	0
HF_43	0	1	0	0	0	0	0	0	0	0	0
HF_44	0	1	0	0	0	0	0	0	0	0	0
HF_57	0	0	0	0	0	0	0	0	4	1	0
HF_58	28	1	0	0	0	0	0	0	2	8	0
HF_59	0	0	0	0	0	0	0	0	0	1	0
HF_60	0	0	0	0	0	0	0	0	0	0	0
HF_73	3	0	0	0	0	0	0	0	1	0	0
HFFG_45	0	0	0	0	0	0	0	0	0	0	0
HFFG_46	0	0	0	0	0	0	1	0	0	0	0
HFFG_47	0	0	0	0	0	0	0	0	0	0	0
HFFG_48	0	0	0	0	0	0	0	0	1	0	0
HFFG_61	0	0	0	0	0	0	0	0	0	0	0
HFFG_62	0	0	0	0	0	0	0	0	0	0	0
HFFG_75	0	0	0	0	0	0	0	0	0	0	0
HFFG_76	0	1	0	1	0	0	3	0	0	0	0

Kingdom	Bacteria	Bacteria	Bacteria	Bacteria	Bacteria	Bacteria	Bacteria	Bacteria	Bacteria	Bacteria	Bacteria
Phylum	Firmicutes	Firmicutes	Firmicutes	Firmicutes	Firmicutes	Firmicutes	Firmicutes	Firmicutes	Firmicutes	Firmicutes	Firmicutes
Class	Clostridia	Clostridia	Clostridia	Clostridia	Clostridia	Clostridia	Clostridia	Clostridia	Clostridia	Clostridia	Clostridia
Order	Clostridiales	Clostridiales	Clostridiales	Clostridiales	Clostridiales	Clostridiales	Clostridiales	Clostridiales	Clostridiales	Clostridiales	Clostridiales
Family	Ruminococcaceae	Lachnospiraceae	Lachnospiraceae	Ruminococcaceae	Lachnospiraceae	Clostridiaceae_	Clostridiaceae_	Lachnospiraceae	Clostridiaceae_	Lachnospiraceae	Peptostreptococcaceae
Genus	Anaerotruncus	Clostridium_XlVa	Clostridium_XlVa	Hydrogenoanaerobacterium	Clostridium_XlVa	Alkaliphilus	Alkaliphilus	Clostridium_XlVa	Alkaliphilus	Acetatifactor	Filifactor
#NAME	ZOTU_0204	ZOTU_0205	ZOTU_0206	ZOTU_0207	ZOTU_0208	ZOTU_0209	ZOTU_0210	ZOTU_0211	ZOTU_0212	ZOTU_0213	ZOTU_0214
CD_49	0	0	0	0	1	0	0	0	0	0	0
CD_50	0	0	0	0	0	0	0	0	0	1	0
CD_52	0	0	1	0	0	0	0	0	0	1	0
CD_65	1	0	0	1	5	0	0	0	1	2	0
CD_67	0	0	0	1	0	0	2	0	0	0	0
CD_68	0	0	0	0	3	0	0	0	0	0	0
CD_78	0	0	0	0	0	0	0	0	0	0	0
CD_79	3	0	0	0	0	0	0	0	0	0	0
CD_80	0	0	0	0	0	0	0	0	0	0	0
CDFG_53	0	0	0	0	0	0	0	0	0	0	0
CDFG_54	0	0	0	0	1	0	0	0	0	2	0
CDFG_55	0	0	0	0	0	0	0	0	2	1	0
CDFG_56	0	0	0	0	0	0	1	0	0	1	0
CDFG_70	1	0	5	0	0	0	0	0	0	3	0
CDFG_71	0	0	3	0	0	1	0	0	0	8	0
HF_41	0	0	0	0	0	0	0	0	0	0	0
HF_43	4	0	0	0	0	0	0	0	0	0	0
HF_44	0	0	0	0	0	0	0	0	0	0	0
HF_57	0	0	0	0	0	0	5	0	0	0	0
HF_58	0	0	0	0	0	0	2	0	0	0	0
HF_59	0	0	0	0	0	0	1	0	0	0	0
HF_60	0	0	0	0	0	0	0	0	0	0	0
HF_73	0	0	0	0	0	0	0	0	0	0	0
HFFG_45	0	0	0	0	0	0	0	0	0	0	0
HFFG_46	0	0	0	0	0	0	0	0	0	0	0
HFFG_47	0	0	0	0	0	0	0	0	0	0	0
HFFG_48	0	0	0	0	0	0	0	0	0	0	0
HFFG_61	0	0	0	0	0	0	0	0	0	0	0
HFFG_62	0	0	0	0	0	0	0	0	0	0	0
HFFG_75	0	0	0	0	0	0	0	0	0	0	0
HFFG_76	1	0	0	0	0	0	0	0	0	0	0

Kingdom	Bacteria	Bacteria	Bacteria	Bacteria	Bacteria	Bacteria	Bacteria	Bacteria	Bacteria	Bacteria
Phylum	Firmicutes	Firmicutes	Firmicutes	Firmicutes	Firmicutes	Bacteroidetes	Firmicutes	Actinobacteria	Firmicutes	Firmicutes
Class	Clostridia	Clostridia	Clostridia	Clostridia	Clostridia	Bacteroidia	Clostridia	Actinobacteria	Clostridia	Clostridia
Order	Clostridiales	Clostridiales	Clostridiales	Clostridiales	Clostridiales	Bacteroidales	Clostridiales	Coriobacteriales	Clostridiales	Clostridiales
Family	Ruminococcaceae	Ruminococcaceae	Lachnospiraceae	Ruminococcaceae	Lachnospiraceae	Porphyromonadaceae	Ruminococcaceae	Coriobacteriaceae	Ruminococcaceae	Ruminococcaceae
Genus	Intestinimonas	Ruminococcus	Lachnospiracea_incertae_sedis	Sporobacter	Acetatifactor	Barnesiella	Sporobacter	Eggerthella	Anaerotruncus	Clostridium_IV
#NAME	ZOTU_0215	ZOTU_0216	ZOTU_0217	ZOTU_0218	ZOTU_0219	ZOTU_0220	ZOTU_0221	ZOTU_0222	ZOTU_0223	ZOTU_0224
CD_49	1	0	2	0	0	0	0	0	0	0
CD_50	0	0	0	0	9	0	0	11	0	0
CD_52	0	0	0	0	2	0	0	5	0	0
CD_65	0	0	8	0	8	0	0	1	0	1
CD_67	0	0	0	0	0	0	0	0	0	0
CD_68	0	3	24	0	4	0	0	5	0	0
CD_78	0	0	0	0	0	0	0	0	0	0
CD_79	0	0	0	0	3	0	0	0	0	0
CD_80	0	0	0	0	0	0	0	0	0	0
CDFG_53	0	0	0	0	0	0	0	0	0	0
CDFG_54	0	2	0	0	1	0	0	0	0	2
CDFG_55	0	0	0	0	0	0	0	0	0	1
CDFG_56	0	7	6	2	1	0	0	0	0	0
CDFG_70	0	10	8	0	30	0	0	0	0	0
CDFG_71	2	14	12	0	16	0	0	4	0	0
HF_41	0	0	0	0	0	0	0	0	0	0
HF_43	0	0	0	0	0	0	0	0	0	0
HF_44	0	0	0	0	0	0	0	0	0	1
HF_57	0	0	0	0	1	0	0	0	0	1
HF_58	0	0	7	0	1	0	0	0	1	1
HF_59	0	0	4	1	0	0	0	0	0	0
HF_60	0	0	0	0	0	0	0	0	0	0
HF_73	0	0	1	0	1	0	0	0	0	0
HFFG_45	0	1	0	0	3	0	0	0	0	0
HFFG_46	0	0	0	0	4	0	0	0	0	0
HFFG_47	0	0	0	0	0	0	0	0	0	0
HFFG_48	0	0	1	0	0	0	0	0	0	0
HFFG_61	0	0	3	0	0	0	0	3	0	0
HFFG_62	0	0	2	0	1	0	0	0	0	0
HFFG_75	0	0	0	0	0	0	0	0	0	0
HFFG_76	0	2	5	0	0	0	1	0	0	1

Kingdom	Bacteria	Bacteria	Bacteria	Bacteria	Bacteria	Bacteria	Bacteria	Bacteria	Bacteria	Bacteria	Bacteria
Phylum	Firmicutes	Firmicutes	Firmicutes	Firmicutes	Firmicutes	Bacteroidetes	Firmicutes	Firmicutes	Firmicutes	Firmicutes	Actinobacteria
Class	Clostridia	Clostridia	Clostridia	Clostridia	Clostridia	Bacteroidia	Clostridia	Clostridia	Clostridia	Clostridia	Actinobacteria
Order	Clostridiales	Clostridiales	Clostridiales	Clostridiales	Clostridiales	Bacteroidales	Clostridiales	Clostridiales	Clostridiales	Clostridiales	Actinomycetales
Family	Lachnospiraceae	Ruminococcaceae	Lachnospiraceae	Clostridiaceae_	Lachnospiraceae	Rikenellaceae	Ruminococcaceae	Lachnospiraceae	Lachnospiraceae	Clostridiaceae_	Streptomycetaceae
Genus	Clostridium_XlVa	Hydrogenoanaerobacterium	Acetatifactor	Alkaliphilus	Clostridium_XlVa	Alistipes	Acetanaerobacterium	Clostridium_XlVb	Clostridium_XlVa	Alkaliphilus	Streptomyces
#NAME	ZOTU_0225	ZOTU_0226	ZOTU_0227	ZOTU_0228	ZOTU_0229	ZOTU_0230	ZOTU_0231	ZOTU_0232	ZOTU_0233	ZOTU_0234	ZOTU_0235
CD_49	0	0	0	0	0	0	0	0	0	0	0
CD_50	0	1	0	0	0	0	0	5	0	0	0
CD_52	0	0	0	0	0	0	0	0	1	0	0
CD_65	0	0	0	0	0	0	0	8	2	0	0
CD_67	0	0	0	0	0	0	0	2	0	0	0
CD_68	0	0	0	0	0	0	0	2	4	0	0
CD_78	0	0	0	0	0	0	0	0	0	0	0
CD_79	0	0	0	0	0	0	0	0	0	0	0
CD_80	0	0	0	0	0	0	0	0	0	0	0
CDFG_53	0	0	0	0	0	0	0	0	0	0	0
CDFG_54	0	0	0	0	0	0	0	2	0	0	0
CDFG_55	0	0	0	0	0	0	0	0	2	0	0
CDFG_56	0	0	0	0	0	0	0	5	1	0	0
CDFG_70	0	0	0	0	0	0	0	14	9	0	31
CDFG_71	0	0	0	0	0	0	0	4	0	0	4
HF_41	0	0	0	0	0	0	0	0	0	0	0
HF_43	0	0	0	0	0	0	0	0	8	0	0
HF_44	0	0	0	0	0	0	0	0	1	0	1
HF_57	0	0	0	0	0	0	0	1	0	0	0
HF_58	0	0	0	0	0	0	0	14	19	0	0
HF_59	0	0	1	0	0	0	0	5	8	0	0
HF_60	0	0	0	0	0	0	0	0	0	0	0
HF_73	0	0	1	0	0	0	1	2	2	0	0
HFFG_45	0	0	0	0	0	0	0	4	2	0	0
HFFG_46	0	0	0	0	0	0	0	2	1	0	0
HFFG_47	0	0	0	0	0	0	0	0	0	0	0
HFFG_48	0	0	0	0	0	0	0	0	1	1	0
HFFG_61	0	0	0	0	0	0	0	4	1	0	9
HFFG_62	0	0	0	0	0	0	0	1	0	0	3
HFFG_75	0	0	0	0	0	0	0	0	0	0	0
HFFG_76	0	2	0	0	0	0	0	10	3	1	0

Kingdom	Bacteria	Bacteria	Bacteria	Bacteria	Bacteria	Bacteria	Bacteria	Bacteria	Bacteria
Phylum	Firmicutes	Firmicutes	Firmicutes	Firmicutes	Firmicutes	Firmicutes	Firmicutes	Actinobacteria	Firmicutes
Class	Clostridia	Clostridia	Clostridia	Clostridia	Clostridia	Clostridia	Clostridia	Actinobacteria	Clostridia
Order	Clostridiales	Clostridiales	Clostridiales	Clostridiales	Clostridiales	Clostridiales	Clostridiales	Coriobacteriales	Clostridiales
Family	Lachnospiraceae	Lachnospiraceae	Lachnospiraceae	Lachnospiraceae	Lachnospiraceae	Clostridiaceae_	Lachnospiraceae	Coriobacteriaceae	Lachnospiraceae
Genus	Clostridium_XlVa	Lachnospiracea_incertae_sedis	Clostridium_XlVa	Butyrivibrio	Clostridium_XlVa	Alkaliphilus	Clostridium_XlVa	Enterorhabdus	Lachnospiracea_incertae_sedis
#NAME	ZOTU_0236	ZOTU_0237	ZOTU_0238	ZOTU_0239	ZOTU_0240	ZOTU_0241	ZOTU_0242	ZOTU_0243	ZOTU_0244
CD_49	0	0	0	0	0	0	0	0	1
CD_50	0	0	0	2	0	0	0	0	1
CD_52	0	0	0	0	0	0	0	4	0
CD_65	1	0	2	0	0	0	0	2	0
CD_67	0	0	0	0	0	0	0	0	0
CD_68	0	0	0	1	0	0	0	0	0
CD_78	0	0	0	0	0	0	0	0	0
CD_79	0	0	0	1	0	0	0	0	1
CD_80	0	0	0	0	0	0	0	0	0
CDFG_53	0	0	0	0	0	0	0	1	0
CDFG_54	0	0	1	0	0	0	0	0	1
CDFG_55	1	0	0	0	0	0	0	2	0
CDFG_56	0	0	0	4	0	0	0	0	0
CDFG_70	0	0	4	1	21	0	2	0	14
CDFG_71	0	0	0	13	0	0	1	0	5
HF_41	0	0	0	0	0	0	0	0	0
HF_43	0	0	5	0	0	0	0	0	0
HF_44	0	0	0	0	0	0	0	0	0
HF_57	0	0	2	0	0	0	0	1	0
HF_58	0	0	9	2	0	0	0	0	0
HF_59	0	0	2	0	0	0	0	1	0
HF_60	0	0	0	0	0	0	0	0	0
HF_73	0	0	0	0	0	0	0	0	0
HFFG_45	0	0	0	0	0	0	0	0	0
HFFG_46	0	0	0	0	0	0	0	1	1
HFFG_47	0	0	0	0	0	0	0	0	0
HFFG_48	0	0	0	0	0	0	0	0	0
HFFG_61	0	0	3	0	0	0	0	0	0
HFFG_62	0	0	1	0	0	0	0	0	0
HFFG_75	0	0	0	0	0	0	0	0	0
HFFG_76	0	0	4	1	0	0	0	3	1

Kingdom	Bacteria	Bacteria	Bacteria	Bacteria	Bacteria	Bacteria	Bacteria	Bacteria	Bacteria	Bacteria
Phylum	Firmicutes	Firmicutes	Firmicutes	Firmicutes	Firmicutes	Firmicutes	Firmicutes	Firmicutes	Firmicutes	Firmicutes
Class	Clostridia	Clostridia	Erysipelotrichia	Clostridia	Clostridia	Clostridia	Clostridia	Clostridia	Bacilli	Clostridia
Order	Clostridiales	Clostridiales	Erysipelotrichales	Clostridiales	Clostridiales	Clostridiales	Clostridiales	Clostridiales	Bacillales	Clostridiales
Family	Lachnospiraceae	Ruminococcaceae	Erysipelotrichaceae	Lachnospiraceae	Lachnospiraceae	Lachnospiraceae	Peptostreptococcaceae	Lachnospiraceae	Staphylococcaceae	Lachnospiraceae
Genus	Lachnospiracea_incertae_sedis	Clostridium_IV	Catenisphaera	Clostridium_XlVa	Roseburia	Marvinbryantia	Filifactor	Acetatifactor	Staphylococcus	Clostridium_XlVa
#NAME	ZOTU_0245	ZOTU_0246	ZOTU_0247	ZOTU_0248	ZOTU_0249	ZOTU_0250	ZOTU_0251	ZOTU_0252	ZOTU_0253	ZOTU_0254
CD_49	0	1	0	0	0	0	0	0	9	0
CD_50	0	0	0	0	0	0	0	0	3	0
CD_52	0	0	0	0	0	0	0	0	0	0
CD_65	0	0	0	0	0	0	0	0	2	0
CD_67	0	0	0	0	0	0	0	0	0	0
CD_68	0	0	0	0	0	2	0	0	0	0
CD_78	0	0	0	1	0	0	0	0	0	0
CD_79	0	0	0	1	0	0	0	1	0	0
CD_80	0	0	0	0	0	0	0	0	9	0
CDFG_53	0	0	0	0	0	1	0	0	304	0
CDFG_54	0	0	0	3	0	1	0	1	96	0
CDFG_55	0	0	1	0	0	1	0	0	12	0
CDFG_56	0	1	0	11	0	1	0	0	71	2
CDFG_70	0	0	0	7	0	0	0	5	1	0
CDFG_71	0	0	0	7	0	1	0	4	3	0
HF_41	0	0	0	0	0	0	0	0	31	0
HF_43	0	0	0	0	0	0	0	0	6	0
HF_44	0	0	0	0	0	0	0	0	0	0
HF_57	0	0	0	0	0	0	0	0	15	0
HF_58	1	1	0	0	0	0	0	0	39	0
HF_59	1	0	1	0	0	0	0	0	3	0
HF_60	0	0	0	0	0	0	0	0	27	0
HF_73	1	1	0	0	0	0	0	0	55	0
HFFG_45	0	0	0	0	0	0	0	0	12	0
HFFG_46	0	0	0	0	0	0	0	0	24	0
HFFG_47	0	0	0	0	0	0	0	0	51	0
HFFG_48	0	0	0	1	0	0	0	0	114	0
HFFG_61	0	0	0	0	0	0	0	0	0	0
HFFG_62	0	0	0	0	0	0	0	0	0	0
HFFG_75	0	0	0	0	0	0	0	0	5	0
HFFG_76	0	1	0	0	0	0	0	0	4	0

Kingdom	Bacteria	Bacteria	Bacteria	Bacteria	Bacteria	Bacteria	Bacteria	Bacteria	Bacteria	Bacteria	Bacteria
Phylum	Firmicutes	Firmicutes	Firmicutes	Firmicutes	Firmicutes	Firmicutes	Tenericutes	Firmicutes	Tenericutes	Firmicutes	Firmicutes
Class	Clostridia	Clostridia	Clostridia	Clostridia	Clostridia	Clostridia	Mollicutes	Clostridia	Mollicutes	Clostridia	Clostridia
Order	Clostridiales	Clostridiales	Clostridiales	Clostridiales	Clostridiales	Clostridiales	Acholeplasmatales	Clostridiales	Acholeplasmatales	Clostridiales	Clostridiales
Family	Lachnospiraceae	Lachnospiraceae	Lachnospiraceae	Lachnospiraceae	Lachnospiraceae	Lachnospiraceae	Acholeplasmataceae	Lachnospiraceae	Acholeplasmataceae	Clostridiaceae_	Lachnospiraceae
Genus	Clostridium_XlVa	Butyrivibrio	Clostridium_XlVa	Clostridium_XlVb	Clostridium_XlVa	Clostridium_XlVa	Acholeplasma	Clostridium_XlVa	Acholeplasma	Geosporobacter	Clostridium_XlVa
#NAME	ZOTU_0255	ZOTU_0256	ZOTU_0257	ZOTU_0258	ZOTU_0259	ZOTU_0260	ZOTU_0261	ZOTU_0262	ZOTU_0263	ZOTU_0264	ZOTU_0265
CD_49	0	0	0	0	0	0	0	0	0	0	0
CD_50	0	0	0	0	0	0	0	0	0	0	2
CD_52	0	0	8	2	0	0	0	0	0	0	1
CD_65	0	0	0	1	0	0	0	0	0	0	1
CD_67	0	0	0	0	0	0	0	0	0	0	0
CD_68	1	0	0	0	0	0	0	0	0	0	0
CD_78	0	0	0	2	1	0	0	0	0	0	0
CD_79	0	0	2	0	3	0	1	0	0	0	0
CD_80	0	0	1	0	0	0	0	0	0	0	0
CDFG_53	0	0	0	0	0	0	0	0	0	0	0
CDFG_54	0	0	1	0	0	0	0	0	0	0	0
CDFG_55	0	0	0	0	0	0	0	0	0	0	0
CDFG_56	0	0	0	1	0	0	0	0	0	0	0
CDFG_70	0	0	1	6	1	0	0	0	0	0	0
CDFG_71	2	0	8	0	0	0	1	0	0	0	0
HF_41	0	0	0	0	0	0	0	0	0	0	0
HF_43	0	0	0	0	0	0	0	0	0	0	0
HF_44	0	0	0	0	0	0	0	0	0	0	0
HF_57	0	0	1	0	0	0	0	0	0	0	0
HF_58	0	0	16	0	0	0	0	0	0	1	0
HF_59	1	0	0	0	0	0	0	0	0	0	0
HF_60	0	0	0	0	0	0	0	0	0	0	0
HF_73	0	0	1	0	0	0	0	0	0	0	0
HFFG_45	0	0	0	0	0	0	0	3	0	0	1
HFFG_46	1	0	0	1	0	0	0	1	0	0	0
HFFG_47	0	0	0	0	0	0	0	0	0	0	0
HFFG_48	0	0	0	0	0	0	0	0	0	0	0
HFFG_61	0	0	0	0	0	0	0	0	0	0	0
HFFG_62	0	0	0	0	0	0	0	1	0	0	0
HFFG_75	0	0	0	0	0	0	0	0	0	0	0
HFFG_76	0	1	0	1	0	0	2	0	0	0	0

Kingdom	Bacteria	Bacteria	Bacteria	Bacteria	Bacteria	Bacteria	Bacteria	Bacteria	Bacteria	Bacteria	Bacteria
Phylum	Tenericutes	Firmicutes	Firmicutes	Firmicutes	Firmicutes	Firmicutes	Firmicutes	Firmicutes	Firmicutes	Firmicutes	Firmicutes
Class	Mollicutes	Clostridia	Clostridia	Clostridia	Clostridia	Clostridia	Clostridia	Clostridia	Clostridia	Erysipelotrichia	Clostridia
Order	Entomoplasmatales	Clostridiales	Clostridiales	Clostridiales	Clostridiales	Clostridiales	Clostridiales	Clostridiales	Clostridiales	Erysipelotrichales	Clostridiales
Family	Spiroplasmataceae	Ruminococcaceae	Lachnospiraceae	Lachnospiraceae	Lachnospiraceae	Lachnospiraceae	Ruminococcaceae	Lachnospiraceae	Lachnospiraceae	Erysipelotrichaceae	Clostridiaceae_
Genus	Spiroplasma	Butyricicoccus	Roseburia	Blautia	Clostridium_XlVa	Clostridium_XlVa	Clostridium_IV	Clostridium_XlVa	Clostridium_XlVa	Holdemania	Geosporobacter
#NAME	ZOTU_0266	ZOTU_0267	ZOTU_0268	ZOTU_0269	ZOTU_0270	ZOTU_0271	ZOTU_0272	ZOTU_0273	ZOTU_0274	ZOTU_0275	ZOTU_0276
CD_49	0	0	0	0	0	0	0	0	0	0	0
CD_50	0	0	0	0	0	0	0	0	0	0	0
CD_52	0	0	0	0	0	2	0	0	0	0	0
CD_65	0	0	0	0	1	1	0	0	0	0	0
CD_67	0	0	0	0	0	0	0	0	0	0	0
CD_68	0	0	0	0	0	1	0	0	0	0	0
CD_78	0	0	0	0	0	0	0	0	0	0	0
CD_79	0	0	0	0	0	0	0	0	0	0	0
CD_80	0	0	0	0	0	0	0	0	0	0	0
CDFG_53	0	0	0	0	0	0	0	0	0	0	0
CDFG_54	0	0	0	0	0	0	0	0	8	0	0
CDFG_55	0	0	0	0	0	0	0	0	1	1	0
CDFG_56	0	0	0	0	0	0	0	2	11	0	0
CDFG_70	0	0	0	0	0	2	0	5	0	0	0
CDFG_71	0	0	0	0	0	4	0	4	0	0	1
HF_41	0	0	0	0	0	0	0	0	0	0	0
HF_43	0	0	0	0	0	0	0	0	0	0	0
HF_44	0	0	0	0	0	0	0	0	0	0	0
HF_57	0	0	0	0	0	1	0	0	0	0	0
HF_58	0	0	0	0	0	13	0	0	0	0	0
HF_59	0	0	0	0	0	0	0	0	0	0	0
HF_60	0	0	0	0	0	0	0	0	0	0	0
HF_73	0	0	0	0	0	0	0	0	0	0	0
HFFG_45	0	0	0	0	0	0	0	0	1	0	0
HFFG_46	0	0	0	0	0	0	0	0	0	0	0
HFFG_47	0	0	0	0	0	0	0	0	0	0	0
HFFG_48	0	0	0	0	0	0	0	0	0	0	0
HFFG_61	0	0	0	0	0	0	0	0	0	0	0
HFFG_62	0	0	0	0	0	0	0	0	0	0	0
HFFG_75	0	0	0	0	0	0	0	0	0	0	0
HFFG_76	0	0	0	0	0	0	0	0	0	0	0

Kingdom	Bacteria	Bacteria	Bacteria	Bacteria	Bacteria	Bacteria	Bacteria	Bacteria	Bacteria
Phylum	Firmicutes	Cyanobacteria_Chloroplast	Firmicutes	Firmicutes	Firmicutes	Firmicutes	Firmicutes	Tenericutes	Firmicutes
Class	Clostridia	Chloroplast	Clostridia	Clostridia	Clostridia	Clostridia	Clostridia	Mollicutes	Clostridia
Order	Clostridiales	Chloroplast	Clostridiales	Clostridiales	Clostridiales	Clostridiales	Clostridiales	Entomoplasmatales	Clostridiales
Family	Ruminococcaceae	Streptophyta	Lachnospiraceae	Lachnospiraceae	Clostridiaceae_	Lachnospiraceae	Lachnospiraceae	Spiroplasmataceae	Clostridiaceae_
Genus	Oscillibacter	NA	Clostridium_XlVa	Lachnospiracea_incertae_sedis	Clostridium_sensu_stricto	Clostridium_XlVa	Lachnospiracea_incertae_sedis	Spiroplasma	Alkaliphilus
#NAME	ZOTU_0277	ZOTU_0278	ZOTU_0279	ZOTU_0280	ZOTU_0281	ZOTU_0282	ZOTU_0283	ZOTU_0284	ZOTU_0285
CD_49	0	0	0	0	0	0	0	0	0
CD_50	0	0	0	0	0	0	0	0	0
CD_52	0	0	0	0	0	0	0	0	0
CD_65	0	0	0	0	0	0	0	1	0
CD_67	0	0	0	0	0	0	0	0	0
CD_68	0	0	0	0	0	0	0	0	0
CD_78	0	0	0	0	0	0	0	0	0
CD_79	2	0	0	0	0	0	0	0	0
CD_80	0	0	0	0	0	0	0	0	0
CDFG_53	0	0	0	0	0	0	0	0	0
CDFG_54	0	0	0	0	0	0	0	0	0
CDFG_55	0	1	0	0	0	0	0	0	0
CDFG_56	0	0	0	0	0	0	0	0	0
CDFG_70	0	0	0	0	0	0	0	0	0
CDFG_71	0	0	0	0	0	0	0	0	0
HF_41	0	0	0	0	0	0	0	0	0
HF_43	0	0	0	0	0	0	0	0	0
HF_44	0	0	0	0	0	0	0	0	0
HF_57	0	0	0	0	0	0	0	2	0
HF_58	0	0	1	0	0	0	0	0	0
HF_59	0	0	0	0	0	0	0	0	0
HF_60	0	0	0	0	0	0	0	0	0
HF_73	0	0	0	0	0	0	0	0	0
HFFG_45	0	2	0	0	0	0	0	0	0
HFFG_46	0	5	0	0	0	0	0	0	0
HFFG_47	0	0	0	0	0	0	0	0	0
HFFG_48	0	0	0	0	0	0	0	0	0
HFFG_61	0	7	0	0	0	0	0	0	0
HFFG_62	0	3	0	0	0	0	0	0	0
HFFG_75	0	0	0	0	0	0	0	0	0
HFFG_76	0	1	0	0	2	0	0	0	0

Kingdom	Bacteria	Bacteria	Bacteria	Bacteria	Bacteria	Bacteria	Bacteria	Bacteria	Bacteria	Bacteria	Bacteria
Phylum	Firmicutes	Firmicutes	Firmicutes	Firmicutes	Firmicutes	Firmicutes	Firmicutes	Firmicutes	Firmicutes	Proteobacteria	Firmicutes
Class	Clostridia	Clostridia	Clostridia	Clostridia	Clostridia	Clostridia	Bacilli	Bacilli	Clostridia	Gammaproteobacteria	Clostridia
Order	Clostridiales	Clostridiales	Clostridiales	Clostridiales	Clostridiales	Halanaerobiales	Bacillales	Lactobacillales	Clostridiales	Pasteurellales	Clostridiales
Family	Lachnospiraceae	Lachnospiraceae	Ruminococcaceae	Lachnospiraceae	Lachnospiraceae	Halanaerobiaceae	Bacillaceae_	Streptococcaceae	Ruminococcaceae	Pasteurellaceae	Ruminococcaceae
Genus	Clostridium_XlVa	Clostridium_XlVa	Intestinimonas	Clostridium_XlVa	Clostridium_XlVa	Halanaerobium	Bacillus	Streptococcus	Clostridium_IV	Haemophilus	Sporobacter
#NAME	ZOTU_0286	ZOTU_0287	ZOTU_0288	ZOTU_0289	ZOTU_0290	ZOTU_0291	ZOTU_0292	ZOTU_0293	ZOTU_0294	ZOTU_0295	ZOTU_0296
CD_49	0	0	0	0	0	0	0	1	0	1	0
CD_50	0	0	0	0	0	0	0	0	0	0	0
CD_52	0	0	0	0	0	0	0	0	0	0	1
CD_65	0	0	0	0	1	0	0	0	0	0	0
CD_67	0	0	0	0	0	0	0	0	0	0	0
CD_68	0	0	0	0	0	0	0	5	0	3	0
CD_78	0	0	0	0	0	0	0	0	0	0	0
CD_79	1	0	0	0	0	0	0	0	0	0	2
CD_80	0	0	0	1	0	0	0	0	0	0	0
CDFG_53	0	0	0	0	0	0	0	0	0	0	0
CDFG_54	0	0	0	0	1	1	2	1	1	1	2
CDFG_55	0	0	0	0	0	0	0	0	0	0	0
CDFG_56	0	0	0	0	0	0	0	11	0	0	0
CDFG_70	4	0	1	0	0	0	0	0	0	0	2
CDFG_71	0	1	0	0	0	0	1	0	1	0	0
HF_41	0	0	0	0	0	0	0	1	0	0	2
HF_43	0	0	0	0	0	0	0	1	0	0	0
HF_44	0	0	0	0	0	0	0	0	0	0	0
HF_57	2	0	0	0	0	0	0	9	0	9	0
HF_58	0	0	0	0	0	0	1	2	0	6	0
HF_59	0	0	0	0	0	0	0	1	0	9	0
HF_60	0	0	0	0	0	0	0	1	0	2	0
HF_73	0	0	0	0	0	0	0	0	0	0	0
HFFG_45	0	0	0	0	0	0	0	0	0	0	0
HFFG_46	0	0	0	0	0	0	0	1	0	0	0
HFFG_47	0	0	0	0	0	0	0	0	0	0	0
HFFG_48	0	0	0	0	0	0	0	0	0	0	0
HFFG_61	0	0	0	0	1	0	0	2	0	2	0
HFFG_62	0	0	0	0	0	0	0	3	0	2	0
HFFG_75	0	0	0	0	0	0	0	0	0	0	0
HFFG_76	0	0	1	0	0	0	0	0	0	0	1

Kingdom	Bacteria	Bacteria	Bacteria	Bacteria	Bacteria	Bacteria	Bacteria	Bacteria	Bacteria	Bacteria	Bacteria
Phylum	Firmicutes	Actinobacteria	Firmicutes	Firmicutes	Firmicutes	Firmicutes	Firmicutes	Firmicutes	Firmicutes	Tenericutes	Firmicutes
Class	Clostridia	Actinobacteria	Clostridia	Clostridia	Clostridia	Clostridia	Bacilli	Bacilli	Clostridia	Mollicutes	Clostridia
Order	Clostridiales	Actinomycetales	Clostridiales	Clostridiales	Clostridiales	Clostridiales	Bacillales	Lactobacillales	Clostridiales	Acholeplasmatales	Clostridiales
Family	Lachnospiraceae	Streptomycetaceae	Lachnospiraceae	Lachnospiraceae	Lachnospiraceae	Lachnospiraceae	Bacillaceae_	Enterococcaceae	Lachnospiraceae	Acholeplasmataceae	Ruminococcaceae
Genus	Clostridium_XlVa	Streptomyces	Clostridium_XlVa	Blautia	Clostridium_XlVa	Acetatifactor	Bacillus	Enterococcus	Clostridium_XlVa	Acholeplasma	Anaerotruncus
#NAME	ZOTU_0297	ZOTU_0298	ZOTU_0299	ZOTU_0300	ZOTU_0301	ZOTU_0302	ZOTU_0303	ZOTU_0304	ZOTU_0305	ZOTU_0306	ZOTU_0307
CD_49	0	0	0	0	0	0	0	3	0	0	0
CD_50	0	0	0	0	0	0	0	0	0	0	0
CD_52	0	0	0	0	1	0	0	1	0	0	0
CD_65	2	0	0	0	0	0	1	2	0	0	0
CD_67	0	0	0	0	1	0	0	3	0	0	0
CD_68	0	0	0	0	0	0	1	0	0	0	0
CD_78	0	2	0	0	0	0	0	0	0	0	0
CD_79	0	0	0	0	1	0	0	0	0	0	0
CD_80	0	0	0	1	0	0	0	0	0	0	0
CDFG_53	0	0	0	0	0	0	0	2	0	0	0
CDFG_54	2	0	0	0	0	0	0	80	0	0	0
CDFG_55	0	0	0	0	0	0	0	113	0	0	0
CDFG_56	0	0	0	0	0	0	0	20	0	0	0
CDFG_70	0	0	0	0	2	1	0	0	0	0	0
CDFG_71	1	0	0	0	5	0	0	0	0	0	0
HF_41	0	0	0	0	0	0	0	0	0	0	0
HF_43	0	0	0	0	0	0	0	7	0	0	0
HF_44	0	0	0	0	0	0	0	0	0	0	0
HF_57	0	0	0	0	0	0	0	0	0	0	0
HF_58	0	2	0	0	7	0	0	23	0	0	0
HF_59	0	0	0	2	0	0	0	92	0	0	0
HF_60	0	0	0	0	0	0	0	13	0	0	0
HF_73	0	0	0	1	0	0	0	1	0	0	0
HFFG_45	0	0	0	0	0	0	0	2	0	0	0
HFFG_46	0	0	0	0	0	0	0	7	0	0	0
HFFG_47	0	0	0	0	0	0	0	0	0	0	0
HFFG_48	0	0	0	0	0	0	0	2	0	0	0
HFFG_61	0	0	0	0	0	0	0	5	0	0	0
HFFG_62	0	0	0	0	0	0	0	1	0	0	0
HFFG_75	0	0	0	0	0	0	0	12	0	0	0
HFFG_76	0	1	0	4	0	3	0	31	0	0	0

Kingdom	Bacteria	Bacteria	Bacteria	Bacteria	Bacteria	Bacteria	Bacteria	Bacteria	Bacteria	Bacteria
Phylum	Firmicutes	Firmicutes	Firmicutes	Firmicutes	Firmicutes	Proteobacteria	Firmicutes	Firmicutes	Firmicutes	Firmicutes
Class	Clostridia	Clostridia	Clostridia	Clostridia	Erysipelotrichia	Betaproteobacteria	Clostridia	Clostridia	Clostridia	Clostridia
Order	Clostridiales	Clostridiales	Clostridiales	Clostridiales	Erysipelotrichales	Neisseriales	Clostridiales	Clostridiales	Clostridiales	Clostridiales
Family	Lachnospiraceae	Ruminococcaceae	Ruminococcaceae	Lachnospiraceae	Erysipelotrichaceae	Neisseriaceae	Lachnospiraceae	Lachnospiraceae	Clostridiaceae_	Lachnospiraceae
Genus	Acetatifactor	Flavonifractor	Ethanoligenens	Butyrivibrio	Holdemania	Neisseria	Ruminococcus	Lachnospiracea_incertae_sedis	Alkaliphilus	Clostridium_XlVa
#NAME	ZOTU_0308	ZOTU_0309	ZOTU_0310	ZOTU_0311	ZOTU_0312	ZOTU_0313	ZOTU_0314	ZOTU_0315	ZOTU_0316	ZOTU_0317
CD_49	0	0	0	0	0	5	0	0	0	0
CD_50	0	0	0	0	0	1	0	0	0	0
CD_52	0	0	0	0	0	0	0	0	0	0
CD_65	2	0	0	1	0	0	2	0	0	0
CD_67	0	0	0	0	0	0	0	0	0	0
CD_68	2	0	0	0	0	0	0	1	0	0
CD_78	0	0	0	1	0	0	0	0	0	0
CD_79	0	0	0	2	0	0	1	1	0	0
CD_80	0	0	0	0	0	0	0	0	0	0
CDFG_53	0	0	0	0	0	0	0	0	0	0
CDFG_54	1	0	2	0	0	1	0	0	0	0
CDFG_55	0	1	0	1	0	0	3	0	0	0
CDFG_56	0	0	4	0	0	0	0	0	0	0
CDFG_70	2	0	1	10	0	0	3	18	0	0
CDFG_71	6	0	0	4	0	0	12	2	0	0
HF_41	0	0	0	0	0	0	0	0	0	0
HF_43	0	0	0	0	0	0	0	0	0	0
HF_44	0	0	0	0	0	1	0	0	0	0
HF_57	0	0	0	0	0	1	0	0	0	0
HF_58	0	0	0	0	0	1	0	0	0	0
HF_59	0	0	0	0	0	0	0	0	0	0
HF_60	0	0	0	0	0	0	0	0	0	0
HF_73	0	0	0	0	0	0	0	0	0	0
HFFG_45	0	0	0	0	0	0	0	0	0	0
HFFG_46	0	0	0	0	0	0	0	0	0	0
HFFG_47	0	0	0	0	0	0	0	0	0	0
HFFG_48	0	0	0	1	0	0	0	0	0	0
HFFG_61	0	0	0	0	0	2	0	0	0	0
HFFG_62	0	0	0	0	0	1	0	0	0	0
HFFG_75	0	0	0	0	0	0	0	0	0	0
HFFG_76	0	0	0	0	0	1	0	0	0	0

Kingdom	Bacteria	Bacteria	Bacteria	Bacteria	Bacteria	Bacteria	Bacteria	Bacteria	Bacteria	Bacteria
Phylum	Firmicutes	Firmicutes	Firmicutes	Firmicutes	Firmicutes	Firmicutes	Bacteroidetes	Firmicutes	Firmicutes	Firmicutes
Class	Clostridia	Clostridia	Clostridia	Clostridia	Clostridia	Clostridia	Bacteroidia	Clostridia	Clostridia	Clostridia
Order	Clostridiales	Clostridiales	Clostridiales	Clostridiales	Clostridiales	Clostridiales	Bacteroidales	Clostridiales	Clostridiales	Clostridiales
Family	Lachnospiraceae	Clostridiaceae_	Lachnospiraceae	Lachnospiraceae	Clostridiaceae_	Lachnospiraceae	Porphyromonadaceae	Lachnospiraceae	Gracilibacteraceae	Lachnospiraceae
Genus	Butyrivibrio	Clostridium_sensu_stricto	Butyrivibrio	Clostridium_XlVa	Clostridium_sensu_stricto	Clostridium_XlVa	Parabacteroides	Clostridium_XlVa	Lutispora	Clostridium_XlVa
#NAME	ZOTU_0318	ZOTU_0319	ZOTU_0320	ZOTU_0321	ZOTU_0322	ZOTU_0323	ZOTU_0324	ZOTU_0325	ZOTU_0326	ZOTU_0327
CD_49	0	0	0	0	0	0	0	0	0	0
CD_50	0	0	0	0	0	0	0	1	0	0
CD_52	0	0	0	0	0	0	0	0	1	0
CD_65	0	0	0	0	0	0	0	0	1	0
CD_67	0	0	0	0	0	0	0	0	0	0
CD_68	0	0	0	0	0	0	0	0	1	0
CD_78	0	0	0	0	0	0	0	0	0	0
CD_79	0	0	0	0	0	0	0	0	0	0
CD_80	0	0	0	0	0	0	0	0	0	0
CDFG_53	0	0	0	0	0	0	0	0	0	0
CDFG_54	0	0	0	0	0	0	0	0	2	0
CDFG_55	0	0	0	0	0	0	0	0	0	0
CDFG_56	0	0	0	0	0	0	0	0	0	0
CDFG_70	0	0	0	0	0	0	0	0	0	0
CDFG_71	0	0	0	0	0	0	0	0	1	0
HF_41	0	0	0	0	0	0	0	0	0	0
HF_43	0	0	0	0	0	0	0	0	0	0
HF_44	0	0	0	0	0	0	0	0	0	0
HF_57	0	0	0	0	0	0	0	0	0	0
HF_58	0	0	0	0	0	0	0	0	0	0
HF_59	0	0	0	0	0	0	0	0	0	0
HF_60	0	0	0	0	0	0	0	0	0	0
HF_73	0	0	0	0	0	0	0	0	0	0
HFFG_45	0	0	0	0	0	0	0	0	0	0
HFFG_46	0	0	0	0	0	0	0	0	1	0
HFFG_47	0	0	0	0	0	0	0	0	0	0
HFFG_48	0	0	0	0	0	0	0	0	0	0
HFFG_61	0	0	0	0	0	0	0	0	0	0
HFFG_62	0	0	0	0	0	0	0	0	0	0
HFFG_75	0	0	0	0	0	0	0	0	0	0
HFFG_76	0	0	0	0	0	0	0	0	1	0

Kingdom	Bacteria	Bacteria	Bacteria	Bacteria	Bacteria	Bacteria	Bacteria	Bacteria	Bacteria
Phylum	Firmicutes	Firmicutes	Firmicutes	Firmicutes	Firmicutes	Firmicutes	Bacteroidetes	Firmicutes	Firmicutes
Class	Clostridia	Clostridia	Clostridia	Clostridia	Clostridia	Clostridia	Bacteroidia	Clostridia	Clostridia
Order	Clostridiales	Clostridiales	Clostridiales	Clostridiales	Clostridiales	Clostridiales	Bacteroidales	Clostridiales	Clostridiales
Family	Lachnospiraceae	Peptostreptococcaceae	Ruminococcaceae	Clostridiales_Incertae_Sedis_XI	Lachnospiraceae	Ruminococcaceae	Porphyromonadaceae	Ruminococcaceae	Lachnospiraceae
Genus	Ruminococcus	Clostridium_XI	Clostridium_IV	Tissierella	Acetitomaculum	Oscillibacter	Barnesiella	Sporobacter	Anaerosporobacter
#NAME	ZOTU_0328	ZOTU_0329	ZOTU_0330	ZOTU_0331	ZOTU_0332	ZOTU_0333	ZOTU_0334	ZOTU_0335	ZOTU_0336
CD_49	0	1	0	0	0	0	0	0	0
CD_50	0	0	0	0	0	0	0	0	1
CD_52	0	0	0	0	2	0	0	0	0
CD_65	0	0	0	1	1	0	0	0	0
CD_67	0	0	0	0	0	0	0	0	0
CD_68	0	0	0	0	1	0	0	0	1
CD_78	0	0	0	0	0	0	0	0	0
CD_79	3	0	0	0	0	0	0	0	0
CD_80	0	0	0	0	0	0	0	0	0
CDFG_53	0	1	0	0	0	0	0	0	0
CDFG_54	0	2	0	0	0	0	5	0	1
CDFG_55	6	0	0	0	0	0	12	0	1
CDFG_56	0	1	0	0	0	0	0	0	0
CDFG_70	7	2	0	6	0	5	0	0	0
CDFG_71	6	0	0	1	0	1	0	0	0
HF_41	0	0	0	0	0	0	0	0	0
HF_43	0	0	0	0	0	0	0	0	0
HF_44	0	0	0	0	0	0	0	0	0
HF_57	0	0	0	0	0	0	0	0	0
HF_58	0	3	0	0	0	0	0	0	0
HF_59	0	2	0	0	0	0	0	0	0
HF_60	0	0	0	0	0	0	0	0	0
HF_73	0	0	0	0	0	0	0	0	0
HFFG_45	0	0	0	1	1	0	0	0	0
HFFG_46	0	0	0	3	0	0	0	0	0
HFFG_47	0	0	0	0	0	0	0	0	0
HFFG_48	0	1	0	0	5	0	0	0	0
HFFG_61	0	1	0	0	0	0	0	0	0
HFFG_62	0	2	0	0	0	0	0	0	0
HFFG_75	0	0	1	0	0	0	0	0	0
HFFG_76	0	0	0	0	13	0	0	1	1

Kingdom	Bacteria	Bacteria	Bacteria	Bacteria	Bacteria	Bacteria	Bacteria	Bacteria	Bacteria	Bacteria
Phylum	Bacteroidetes	Firmicutes	Firmicutes	Firmicutes	Firmicutes	Firmicutes	Firmicutes	Firmicutes	Firmicutes	Firmicutes
Class	Bacteroidia	Clostridia	Clostridia	Clostridia	Clostridia	Clostridia	Clostridia	Clostridia	Clostridia	Clostridia
Order	Bacteroidales	Clostridiales	Clostridiales	Clostridiales	Clostridiales	Clostridiales	Clostridiales	Clostridiales	Clostridiales	Clostridiales
Family	Porphyromonadaceae	Lachnospiraceae	Lachnospiraceae	Lachnospiraceae	Lachnospiraceae	Ruminococcaceae	Clostridiaceae_	Lachnospiraceae	Lachnospiraceae	Lachnospiraceae
Genus	Parabacteroides	Clostridium_XlVa	Clostridium_XlVa	Lachnospiracea_incertae_sedis	Clostridium_XlVa	Clostridium_IV	Clostridium_sensu_stricto	Blautia	Acetitomaculum	Lachnobacterium
#NAME	ZOTU_0337	ZOTU_0338	ZOTU_0339	ZOTU_0340	ZOTU_0341	ZOTU_0342	ZOTU_0343	ZOTU_0344	ZOTU_0345	ZOTU_0346
CD_49	0	0	0	0	0	0	0	0	0	0
CD_50	0	0	0	0	0	0	0	0	0	0
CD_52	0	0	0	0	0	0	0	0	0	0
CD_65	0	0	0	0	0	0	0	0	0	0
CD_67	0	0	0	0	0	0	0	0	0	0
CD_68	0	0	0	0	0	0	0	0	0	0
CD_78	0	0	0	0	0	0	0	0	0	0
CD_79	0	0	0	0	0	0	0	0	0	0
CD_80	0	0	0	0	0	0	0	0	0	0
CDFG_53	0	0	0	0	0	0	0	0	0	0
CDFG_54	0	0	0	0	0	0	0	0	0	0
CDFG_55	0	0	0	0	1	0	0	0	0	0
CDFG_56	0	0	0	0	0	0	0	0	0	0
CDFG_70	0	0	0	0	0	0	0	0	3	2
CDFG_71	0	0	0	0	0	0	0	0	1	0
HF_41	0	0	0	0	0	0	0	0	0	0
HF_43	1	0	0	0	0	0	0	0	0	0
HF_44	0	0	0	0	0	0	0	0	0	0
HF_57	0	0	0	0	0	0	0	0	0	0
HF_58	0	0	0	0	0	0	0	0	0	0
HF_59	0	0	0	0	0	0	0	0	0	0
HF_60	0	0	0	0	0	0	0	0	0	0
HF_73	0	0	0	0	0	0	0	0	0	0
HFFG_45	0	0	0	0	4	0	0	0	0	0
HFFG_46	0	0	0	0	0	0	0	0	0	0
HFFG_47	0	0	0	0	0	0	0	0	0	0
HFFG_48	0	0	0	0	0	0	0	0	0	0
HFFG_61	0	0	0	0	1	0	0	0	0	0
HFFG_62	0	0	0	0	0	0	0	0	0	0
HFFG_75	0	0	0	0	0	0	0	0	0	0
HFFG_76	31	0	0	0	0	0	0	0	0	0

Kingdom	Bacteria	Bacteria	Bacteria	Bacteria	Bacteria	Bacteria	Bacteria	Bacteria	Bacteria	Bacteria	Bacteria
Phylum	Firmicutes	Firmicutes	Firmicutes	Firmicutes	Firmicutes	Firmicutes	Firmicutes	Firmicutes	Firmicutes	Firmicutes	Firmicutes
Class	Bacilli	Clostridia	Clostridia	Clostridia	Clostridia	Clostridia	Clostridia	Clostridia	Clostridia	Clostridia	Erysipelotrichia
Order	Bacillales	Clostridiales	Clostridiales	Clostridiales	Clostridiales	Clostridiales	Clostridiales	Clostridiales	Clostridiales	Clostridiales	Erysipelotrichales
Family	Paenibacillaceae_	Lachnospiraceae	Lachnospiraceae	Lachnospiraceae	Lachnospiraceae	Ruminococcaceae	Lachnospiraceae	Lachnospiraceae	Lachnospiraceae	Ruminococcaceae	Erysipelotrichaceae
Genus	Paenibacillus	Ruminococcus	Marvinbryantia	Acetatifactor	Blautia	Intestinimonas	Acetatifactor	Acetatifactor	Shuttleworthia	Clostridium_IV	Catenisphaera
#NAME	ZOTU_0347	ZOTU_0348	ZOTU_0349	ZOTU_0350	ZOTU_0351	ZOTU_0352	ZOTU_0353	ZOTU_0354	ZOTU_0355	ZOTU_0356	ZOTU_0357
CD_49	0	0	0	0	0	0	0	0	0	0	0
CD_50	0	0	0	0	0	0	0	0	0	0	0
CD_52	0	0	0	0	0	0	0	0	0	0	0
CD_65	0	0	0	1	1	0	0	0	0	0	0
CD_67	0	0	0	0	0	0	0	0	0	0	0
CD_68	0	0	0	0	1	0	0	0	0	0	0
CD_78	0	0	0	0	0	0	0	0	0	0	0
CD_79	0	3	0	0	0	0	0	0	0	0	0
CD_80	0	0	0	0	0	0	0	0	0	0	0
CDFG_53	0	0	0	0	0	0	0	0	0	0	0
CDFG_54	1	0	0	0	0	0	0	0	0	0	0
CDFG_55	7	0	0	0	0	0	0	0	0	0	0
CDFG_56	14	0	4	0	0	0	0	0	0	0	0
CDFG_70	0	2	0	0	0	0	0	5	0	0	0
CDFG_71	0	10	0	0	0	0	0	0	1	0	0
HF_41	0	0	0	0	0	0	0	0	0	0	0
HF_43	0	0	0	0	0	0	0	0	0	0	0
HF_44	0	0	0	0	0	0	0	0	0	0	0
HF_57	0	0	0	0	0	0	0	0	0	0	0
HF_58	0	0	0	0	0	0	0	0	0	0	0
HF_59	0	0	0	0	0	0	0	0	0	0	0
HF_60	0	0	0	0	0	0	0	0	0	0	0
HF_73	0	0	0	0	0	0	0	0	0	0	0
HFFG_45	0	0	0	0	0	0	0	0	0	0	0
HFFG_46	0	0	0	0	0	0	0	0	0	0	0
HFFG_47	0	0	0	0	0	0	0	0	0	0	0
HFFG_48	0	0	0	0	0	0	0	0	0	0	0
HFFG_61	0	0	0	0	0	0	0	0	0	0	0
HFFG_62	0	0	0	0	0	0	0	0	0	0	0
HFFG_75	0	0	0	0	0	0	0	0	0	0	0
HFFG_76	0	0	0	0	0	0	0	0	0	0	0

Kingdom	Bacteria	Bacteria	Bacteria	Bacteria	Bacteria	Bacteria	Bacteria	Bacteria	Bacteria	Bacteria
Phylum	Firmicutes	Actinobacteria	Firmicutes	Proteobacteria	Firmicutes	Firmicutes	Firmicutes	Firmicutes	Firmicutes	Firmicutes
Class	Bacilli	Actinobacteria	Bacilli	Gammaproteobacteria	Bacilli	Clostridia	Clostridia	Clostridia	Clostridia	Clostridia
Order	Lactobacillales	Coriobacteriales	Lactobacillales	Pseudomonadales	Bacillales	Clostridiales	Clostridiales	Clostridiales	Clostridiales	Clostridiales
Family	Lactobacillaceae	Coriobacteriaceae	Lactobacillaceae	Pseudomonadaceae	Staphylococcaceae	Natranaerovirga	Lachnospiraceae	Lachnospiraceae	Lachnospiraceae	Lachnospiraceae
Genus	Lactobacillus	Enterorhabdus	Lactobacillus	Pseudomonas	Staphylococcus	NA	Clostridium_XlVa	Clostridium_XlVa	Clostridium_XlVa	Lachnospiracea_incertae_sedis
#NAME	ZOTU_0358	ZOTU_0359	ZOTU_0360	ZOTU_0361	ZOTU_0362	ZOTU_0363	ZOTU_0364	ZOTU_0365	ZOTU_0366	ZOTU_0367
CD_49	0	1	1	1	11	0	0	0	0	0
CD_50	0	9	0	0	5	0	0	0	0	0
CD_52	0	17	0	0	0	0	0	0	0	0
CD_65	0	14	2	0	11	0	0	0	0	0
CD_67	0	0	0	0	4	0	0	0	0	0
CD_68	0	4	0	0	0	0	0	0	0	0
CD_78	0	0	0	0	1	0	0	0	0	0
CD_79	0	0	0	0	12	0	0	0	0	0
CD_80	0	0	0	0	31	0	0	0	0	0
CDFG_53	0	1	0	0	0	0	0	0	0	0
CDFG_54	0	129	0	0	2	0	0	1	0	0
CDFG_55	0	2	0	1	0	0	0	0	0	0
CDFG_56	3	0	0	4	50	0	0	1	0	0
CDFG_70	0	0	0	0	1	0	0	0	0	0
CDFG_71	0	0	0	0	7	0	0	0	0	0
HF_41	0	0	0	0	0	0	0	0	0	0
HF_43	0	0	0	0	3	0	0	0	0	0
HF_44	0	0	0	1	0	0	0	0	0	0
HF_57	0	0	0	2	10	0	0	0	0	0
HF_58	0	5	0	1	60	0	0	0	0	0
HF_59	0	0	0	0	4	0	0	0	0	0
HF_60	0	0	0	1	6	0	0	0	0	0
HF_73	0	0	32	0	2	0	0	0	0	0
HFFG_45	0	0	0	0	11	0	0	0	0	0
HFFG_46	0	0	0	0	0	0	0	0	0	0
HFFG_47	0	0	0	0	1	0	0	0	0	0
HFFG_48	0	0	0	1	328	0	0	0	0	0
HFFG_61	2	0	0	1	0	0	0	0	0	0
HFFG_62	0	0	1	0	0	0	0	0	0	0
HFFG_75	0	0	0	0	8	0	0	0	0	0
HFFG_76	0	0	0	0	4	0	0	0	0	0

Kingdom	Bacteria	Bacteria	Bacteria	Bacteria	Bacteria	Bacteria	Bacteria	Bacteria	Bacteria	Bacteria	Bacteria
Phylum	Firmicutes	Firmicutes	Firmicutes	Actinobacteria	Firmicutes	Firmicutes	Firmicutes	Firmicutes	Firmicutes	Firmicutes	Firmicutes
Class	Clostridia	Clostridia	Clostridia	Actinobacteria	Bacilli	Bacilli	Bacilli	Bacilli	Clostridia	Clostridia	Clostridia
Order	Clostridiales	Clostridiales	Clostridiales	Actinomycetales	Lactobacillales	Bacillales	Lactobacillales	Bacillales	Clostridiales	Clostridiales	Clostridiales
Family	Clostridiaceae_	Clostridiaceae_	Peptostreptococcaceae	Dietziaceae	Enterococcaceae	Planococcaceae	Streptococcaceae	Bacillaceae_	Lachnospiraceae	Ruminococcaceae	Ruminococcaceae
Genus	Alkaliphilus	Clostridium_sensu_stricto	Clostridium_XI	Dietzia	Enterococcus	Viridibacillus	Streptococcus	Bacillus	Acetatifactor	Clostridium_IV	Oscillibacter
#NAME	ZOTU_0368	ZOTU_0369	ZOTU_0370	ZOTU_0371	ZOTU_0372	ZOTU_0373	ZOTU_0374	ZOTU_0375	ZOTU_0376	ZOTU_0377	ZOTU_0378
CD_49	0	0	0	0	0	23	0	4	0	0	0
CD_50	0	0	0	0	0	1	0	3	0	0	0
CD_52	0	0	1	0	0	0	0	0	0	0	0
CD_65	0	0	0	0	0	7	0	1	0	0	0
CD_67	0	0	0	1	0	1	0	2	0	0	0
CD_68	0	0	0	0	0	0	2	1	0	0	0
CD_78	0	0	0	0	0	6	0	6	0	0	0
CD_79	0	0	0	0	0	44	0	3	0	0	0
CD_80	0	0	0	0	0	248	0	1	0	0	0
CDFG_53	0	0	0	0	0	52	0	0	0	0	0
CDFG_54	0	0	3	0	3	26	0	0	0	0	0
CDFG_55	0	0	0	0	1	13	0	0	0	0	0
CDFG_56	0	0	1	0	2	355	0	4	0	0	0
CDFG_70	0	0	4	0	0	11	0	2	0	0	0
CDFG_71	0	0	0	0	1	10	0	5	0	0	0
HF_41	0	0	0	0	0	35	0	1	0	0	0
HF_43	0	0	0	0	0	1	0	0	0	0	0
HF_44	0	0	0	1	0	0	2	0	0	0	0
HF_57	0	0	0	1	1	17	3	2	0	0	0
HF_58	0	0	1	2	0	53	7	2	1	1	0
HF_59	0	0	0	0	2	14	2	0	1	2	0
HF_60	0	0	3	0	0	8	0	0	0	0	0
HF_73	0	0	0	0	0	16	0	1	0	0	0
HFFG_45	0	0	0	0	0	1	0	1	0	0	0
HFFG_46	0	0	0	0	0	2	1	1	1	0	0
HFFG_47	0	0	0	0	0	0	0	0	0	0	0
HFFG_48	0	0	2	0	1	224	0	0	0	0	0
HFFG_61	0	0	0	0	0	4	0	0	0	0	0
HFFG_62	0	0	0	1	0	0	1	0	0	0	0
HFFG_75	0	0	0	0	0	28	0	1	0	0	0
HFFG_76	0	0	0	1	1	46	1	4	1	0	0

Kingdom	Bacteria	Bacteria	Bacteria	Bacteria	Bacteria	Bacteria	Bacteria	Bacteria	Bacteria	Bacteria
Phylum	Firmicutes	Firmicutes	Firmicutes	Firmicutes	Firmicutes	Firmicutes	Firmicutes	Firmicutes	Firmicutes	Firmicutes
Class	Clostridia	Clostridia	Clostridia	Bacilli	Clostridia	Clostridia	Clostridia	Clostridia	Clostridia	Negativicutes
Order	Clostridiales	Clostridiales	Clostridiales	Bacillales	Clostridiales	Clostridiales	Clostridiales	Clostridiales	Clostridiales	Selenomonadales
Family	Lachnospiraceae	Lachnospiraceae	Clostridiaceae_	Planococcaceae	Lachnospiraceae	Clostridiaceae_	Lachnospiraceae	Ruminococcaceae	Clostridiaceae_	Veillonellaceae
Genus	Acetatifactor	Clostridium_XlVa	Clostridium_sensu_stricto	Viridibacillus	Clostridium_XlVa	Clostridium_sensu_stricto	Clostridium_XlVa	Clostridium_IV	Alkaliphilus	Veillonella
#NAME	ZOTU_0379	ZOTU_0380	ZOTU_0381	ZOTU_0382	ZOTU_0383	ZOTU_0384	ZOTU_0385	ZOTU_0386	ZOTU_0387	ZOTU_0388
CD_49	0	0	0	2	0	0	0	0	0	0
CD_50	0	0	0	0	0	0	0	0	0	0
CD_52	1	0	0	0	0	0	0	0	0	0
CD_65	1	0	0	6	0	0	0	0	0	0
CD_67	0	0	0	0	0	0	0	0	0	0
CD_68	0	0	0	0	0	0	0	0	0	0
CD_78	0	0	0	0	0	0	0	0	2	0
CD_79	0	0	0	4	0	0	0	0	0	0
CD_80	0	0	0	8	0	0	0	0	0	0
CDFG_53	0	0	0	21	0	0	0	0	0	0
CDFG_54	0	0	0	8	0	0	0	0	0	0
CDFG_55	0	0	0	0	0	0	0	0	0	0
CDFG_56	0	0	0	25	0	0	0	0	6	0
CDFG_70	0	0	0	0	0	0	0	0	0	0
CDFG_71	0	0	0	2	1	0	0	0	0	0
HF_41	0	0	0	3	0	0	0	0	0	0
HF_43	0	0	0	0	0	0	0	0	0	0
HF_44	0	0	0	0	0	0	0	0	0	0
HF_57	0	0	0	3	0	0	0	0	2	2
HF_58	0	0	0	24	0	0	0	0	2	4
HF_59	1	0	0	1	0	0	0	0	0	2
HF_60	0	0	0	2	0	0	0	0	0	1
HF_73	0	0	0	1	0	0	0	0	2	0
HFFG_45	0	0	0	0	0	0	0	0	0	0
HFFG_46	0	0	0	1	0	0	0	0	0	0
HFFG_47	0	0	0	1	0	0	0	0	0	0
HFFG_48	0	0	0	11	0	0	0	0	0	0
HFFG_61	0	0	0	0	0	0	0	0	0	1
HFFG_62	0	0	0	0	0	0	0	0	0	1
HFFG_75	0	0	0	2	0	0	0	0	0	0
HFFG_76	0	0	0	3	1	0	0	0	1	0

Kingdom	Bacteria	Bacteria	Bacteria	Bacteria	Bacteria	Bacteria	Bacteria	Bacteria	Bacteria
Phylum	Proteobacteria	Cyanobacteria_Chloroplast	Tenericutes	Cyanobacteria_Chloroplast	Firmicutes	Bacteroidetes	Firmicutes	Bacteroidetes	Proteobacteria
Class	Betaproteobacteria	Chloroplast	Mollicutes	Chloroplast	Clostridia	Bacteroidia	Negativicutes	Bacteroidia	Betaproteobacteria
Order	Neisseriales	Chloroplast	Acholeplasmatales	Chloroplast	Clostridiales	Bacteroidales	Selenomonadales	Bacteroidales	Burkholderiales
Family	Neisseriaceae	Streptophyta	Acholeplasmataceae	Streptophyta	Lachnospiraceae	Porphyromonadaceae	Veillonellaceae	Porphyromonadaceae	Burkholderiaceae
Genus	Neisseria	NA	Acholeplasma	NA	Coprococcus	Parabacteroides	Veillonella	Parabacteroides	Burkholderia
#NAME	ZOTU_0389	ZOTU_0390	ZOTU_0391	ZOTU_0392	ZOTU_0393	ZOTU_0394	ZOTU_0395	ZOTU_0396	ZOTU_0397
CD_49	0	0	0	0	0	0	0	0	0
CD_50	0	0	0	0	0	0	0	0	0
CD_52	0	0	0	0	0	0	0	0	0
CD_65	0	0	0	0	0	1	0	0	0
CD_67	0	0	0	0	0	0	0	0	0
CD_68	0	0	0	0	0	14	3	0	0
CD_78	0	0	0	0	0	0	0	0	0
CD_79	0	0	0	0	0	0	0	0	0
CD_80	0	0	0	0	0	0	0	0	0
CDFG_53	0	0	0	0	0	0	0	0	0
CDFG_54	0	0	0	0	0	0	0	0	1
CDFG_55	0	0	0	0	0	0	0	0	0
CDFG_56	0	2	1	0	0	0	0	0	2
CDFG_70	0	0	0	0	0	0	0	0	2
CDFG_71	0	0	0	0	0	0	0	0	2
HF_41	0	0	0	0	0	0	0	0	0
HF_43	0	0	0	0	0	0	0	0	0
HF_44	0	0	0	0	0	0	0	0	2
HF_57	0	0	0	0	0	0	2	0	0
HF_58	0	0	0	0	0	0	1	0	0
HF_59	1	0	3	0	0	0	0	0	3
HF_60	0	0	0	0	0	0	0	0	0
HF_73	0	0	0	0	0	0	0	0	1
HFFG_45	0	0	0	0	0	0	0	0	0
HFFG_46	0	0	0	2	0	0	0	0	0
HFFG_47	0	0	0	0	0	0	0	0	0
HFFG_48	0	0	0	0	0	0	0	0	0
HFFG_61	2	0	0	2	0	0	0	0	0
HFFG_62	0	0	0	0	0	0	0	0	2
HFFG_75	0	0	0	0	0	0	0	0	0
HFFG_76	0	0	0	0	3	0	0	17	0

Kingdom	Bacteria	Bacteria	Bacteria	Bacteria	Bacteria	Bacteria	Bacteria	Bacteria	Bacteria	Bacteria
Phylum	Actinobacteria	Firmicutes	Firmicutes	Firmicutes	Proteobacteria	Firmicutes	Firmicutes	Actinobacteria	Firmicutes	Firmicutes
Class	Actinobacteria	Clostridia	Clostridia	Erysipelotrichia	Betaproteobacteria	Negativicutes	Clostridia	Actinobacteria	Clostridia	Clostridia
Order	Actinomycetales	Clostridiales	Clostridiales	Erysipelotrichales	Burkholderiales	Selenomonadales	Clostridiales	Actinomycetales	Clostridiales	Clostridiales
Family	Corynebacteriaceae	Clostridiales_Incertae_Sedis_XIII	Lachnospiraceae	Erysipelotrichaceae	Burkholderiaceae	Acidaminococcaceae	Eubacteriaceae	Corynebacteriaceae	Lachnospiraceae	Ruminococcaceae
Genus	Corynebacterium	Anaerovorax	Acetatifactor	Clostridium_XVIII	Ralstonia	Phascolarctobacterium	Anaerofustis	Corynebacterium	Acetatifactor	Oscillibacter
#NAME	ZOTU_0398	ZOTU_0399	ZOTU_0400	ZOTU_0401	ZOTU_0402	ZOTU_0403	ZOTU_0404	ZOTU_0405	ZOTU_0406	ZOTU_0407
CD_49	0	0	0	2	0	0	0	0	0	0
CD_50	0	0	0	1	0	0	0	0	0	0
CD_52	0	0	0	0	0	0	0	0	0	0
CD_65	0	0	0	1	0	0	0	0	0	0
CD_67	1	0	0	0	0	0	0	0	0	0
CD_68	0	0	0	0	0	0	0	0	0	0
CD_78	0	0	0	0	0	0	0	0	0	0
CD_79	0	0	0	0	0	0	0	0	0	0
CD_80	0	0	0	1	0	0	0	0	0	0
CDFG_53	0	0	0	0	0	0	0	0	0	0
CDFG_54	0	0	0	0	0	0	0	0	0	0
CDFG_55	0	0	0	0	0	0	0	0	0	0
CDFG_56	0	0	0	0	0	0	0	0	0	0
CDFG_70	0	0	0	0	1	0	0	0	0	0
CDFG_71	0	0	0	0	0	0	0	0	0	0
HF_41	0	0	0	0	0	0	0	0	0	0
HF_43	2	0	0	0	0	0	0	0	0	0
HF_44	0	0	0	0	0	0	0	0	0	0
HF_57	1	0	0	0	0	0	0	1	0	0
HF_58	0	0	0	0	0	0	0	0	0	0
HF_59	0	0	0	0	0	0	0	0	0	0
HF_60	0	0	0	0	0	0	0	0	0	0
HF_73	0	0	0	0	0	0	0	0	0	0
HFFG_45	0	0	0	0	0	0	0	0	0	0
HFFG_46	0	0	0	0	0	0	0	0	0	0
HFFG_47	0	0	0	0	0	0	0	0	0	0
HFFG_48	0	0	0	0	1	0	0	0	0	0
HFFG_61	0	1	0	0	0	0	0	0	0	0
HFFG_62	0	0	0	0	0	0	0	0	0	0
HFFG_75	0	0	0	0	0	0	0	0	0	0
HFFG_76	0	0	0	1	2	0	0	11	0	0

Kingdom	Bacteria	Bacteria	Bacteria	Bacteria	Bacteria	Bacteria	Bacteria	Bacteria	Bacteria
Phylum	Firmicutes	Bacteroidetes	Firmicutes	Actinobacteria	Proteobacteria	Cyanobacteria_Chloroplast	Bacteroidetes	Tenericutes	Firmicutes
Class	Clostridia	Bacteroidia	Clostridia	Actinobacteria	Betaproteobacteria	Chloroplast	Bacteroidia	Mollicutes	Clostridia
Order	Clostridiales	Bacteroidales	Clostridiales	Coriobacteriales	Neisseriales	Chloroplast	Bacteroidales	Acholeplasmatales	Clostridiales
Family	Ruminococcaceae	Porphyromonadaceae	Lachnospiraceae	Coriobacteriaceae	Neisseriaceae	Streptophyta	Porphyromonadaceae	Acholeplasmataceae	Clostridiaceae_
Genus	Ruminococcus	Parabacteroides	Clostridium_XlVa	Atopobium	Neisseria	NA	Parabacteroides	Acholeplasma	Clostridium_sensu_stricto
#NAME	ZOTU_0408	ZOTU_0409	ZOTU_0410	ZOTU_0411	ZOTU_0412	ZOTU_0413	ZOTU_0414	ZOTU_0415	ZOTU_0416
CD_49	0	0	0	0	0	0	0	0	0
CD_50	0	0	0	0	0	0	0	0	0
CD_52	0	0	0	0	0	0	0	0	0
CD_65	0	0	0	0	0	0	0	0	0
CD_67	0	0	0	0	0	0	0	0	0
CD_68	0	0	0	0	2	0	2	0	0
CD_78	0	0	0	0	0	0	0	0	0
CD_79	0	0	0	0	0	0	0	0	0
CD_80	0	0	0	0	0	0	0	0	0
CDFG_53	0	0	0	1	0	0	0	0	0
CDFG_54	0	0	0	0	1	0	0	0	0
CDFG_55	0	0	0	2	0	0	0	0	0
CDFG_56	0	0	0	0	0	0	0	0	0
CDFG_70	0	0	0	0	0	0	0	0	0
CDFG_71	0	0	0	0	0	0	0	0	0
HF_41	0	0	0	0	0	0	0	0	0
HF_43	0	0	0	0	0	0	0	0	0
HF_44	0	0	0	0	0	0	0	0	0
HF_57	0	0	0	0	1	0	0	0	0
HF_58	0	0	0	0	0	0	0	0	0
HF_59	0	0	0	0	2	0	0	0	0
HF_60	0	0	0	0	1	0	0	0	0
HF_73	0	0	0	0	0	0	0	0	0
HFFG_45	0	0	0	0	0	1	0	0	0
HFFG_46	0	0	0	0	0	0	0	0	0
HFFG_47	0	0	0	0	0	0	0	0	0
HFFG_48	0	0	0	0	0	0	0	0	0
HFFG_61	0	0	0	0	0	0	0	0	0
HFFG_62	0	0	0	0	0	0	0	0	0
HFFG_75	0	0	0	0	0	0	0	0	0
HFFG_76	0	13	0	0	0	0	0	0	0

Kingdom	Bacteria	Bacteria	Bacteria	Bacteria	Bacteria	Bacteria	Bacteria	Bacteria	Bacteria	Bacteria
Phylum	Firmicutes	Firmicutes	Cyanobacteria_Chloroplast	Firmicutes	Actinobacteria	Firmicutes	Firmicutes	Actinobacteria	Proteobacteria	Firmicutes
Class	Clostridia	Clostridia	Chloroplast	Clostridia	Actinobacteria	Bacilli	Bacilli	Actinobacteria	Gammaproteobacteria	Bacilli
Order	Clostridiales	Clostridiales	Chloroplast	Clostridiales	Coriobacteriales	Bacillales	Bacillales	Actinomycetales	Pasteurellales	Lactobacillales
Family	Lachnospiraceae	Clostridiaceae_	Streptophyta	Lachnospiraceae	Coriobacteriaceae	Staphylococcaceae	Bacillaceae_	Corynebacteriaceae	Pasteurellaceae	Lactobacillaceae
Genus	Clostridium_XlVa	Clostridium_sensu_stricto	NA	Butyrivibrio	Enterorhabdus	Staphylococcus	Caldibacillus	Corynebacterium	Actinobacillus	Lactobacillus
#NAME	ZOTU_0417	ZOTU_0418	ZOTU_0419	ZOTU_0420	ZOTU_0421	ZOTU_0422	ZOTU_0423	ZOTU_0424	ZOTU_0425	ZOTU_0426
CD_49	0	0	0	0	0	1	0	0	0	0
CD_50	0	0	0	0	4	1	0	0	0	0
CD_52	0	0	0	0	7	0	0	0	0	0
CD_65	0	0	0	0	4	0	0	0	0	0
CD_67	0	0	0	0	1	2	0	0	0	0
CD_68	0	0	0	0	3	0	0	0	0	0
CD_78	0	0	0	0	0	0	0	0	0	0
CD_79	0	0	0	2	0	5	0	0	0	0
CD_80	0	0	0	0	0	0	0	0	0	0
CDFG_53	0	0	0	0	0	0	0	0	0	0
CDFG_54	0	0	1	0	31	1	0	1	0	0
CDFG_55	0	0	0	0	0	0	0	0	0	0
CDFG_56	0	0	0	0	0	1	0	0	0	0
CDFG_70	0	0	0	0	0	2	0	0	0	0
CDFG_71	0	0	0	2	0	2	0	0	0	0
HF_41	0	0	0	0	0	0	0	0	0	0
HF_43	0	0	0	0	0	1	0	0	0	0
HF_44	0	0	0	0	0	0	0	0	1	0
HF_57	0	0	0	0	0	1	0	0	3	0
HF_58	0	0	0	0	3	0	0	3	1	0
HF_59	0	0	0	0	0	0	0	0	2	0
HF_60	0	0	0	0	0	0	0	0	0	0
HF_73	0	0	0	0	0	1	0	0	0	12
HFFG_45	0	0	0	0	0	2	0	0	0	0
HFFG_46	0	0	0	0	0	5	0	0	0	0
HFFG_47	0	0	0	0	0	0	0	0	0	0
HFFG_48	0	0	1	0	0	2	0	0	0	0
HFFG_61	0	0	2	0	0	0	0	1	0	0
HFFG_62	0	0	1	0	0	0	1	0	1	0
HFFG_75	0	0	0	0	0	0	0	0	0	0
HFFG_76	0	0	0	0	1	1	0	0	0	0

Kingdom	Bacteria	Bacteria	Bacteria	Bacteria	Bacteria	Bacteria	Bacteria	Bacteria	Bacteria
Phylum	Firmicutes	Firmicutes	Firmicutes	Actinobacteria	Firmicutes	Proteobacteria	Firmicutes	Firmicutes	Firmicutes
Class	Bacilli	Bacilli	Clostridia	Actinobacteria	Bacilli	Alphaproteobacteria	Erysipelotrichia	Clostridia	Clostridia
Order	Bacillales	Bacillales	Clostridiales	Actinomycetales	Bacillales	Rhizobiales	Erysipelotrichales	Clostridiales	Clostridiales
Family	Bacillaceae_	Bacillales_Incertae_Sedis_XI	Clostridiaceae_	Actinomycetaceae	Staphylococcaceae	Bradyrhizobiaceae	Erysipelotrichaceae	Clostridiaceae_	Lachnospiraceae
Genus	Bacillus	Gemella	Clostridium_sensu_stricto	Actinomyces	Staphylococcus	Bradyrhizobium	Erysipelotrichaceae_incertae_sedis	Alkaliphilus	Clostridium_XlVa
#NAME	ZOTU_0427	ZOTU_0428	ZOTU_0429	ZOTU_0430	ZOTU_0431	ZOTU_0432	ZOTU_0433	ZOTU_0434	ZOTU_0435
CD_49	0	0	0	0	0	0	0	0	0
CD_50	0	0	0	0	0	0	0	0	0
CD_52	0	0	0	0	2	0	0	0	0
CD_65	1	0	0	0	1	0	0	0	0
CD_67	0	0	0	0	2	0	0	0	0
CD_68	0	0	0	0	0	0	0	0	0
CD_78	0	0	0	0	0	0	0	0	0
CD_79	0	0	0	0	0	0	0	0	0
CD_80	0	0	0	0	0	0	0	0	0
CDFG_53	0	0	0	0	0	0	0	0	0
CDFG_54	0	0	0	0	12	0	0	0	0
CDFG_55	0	0	0	0	0	1	0	0	0
CDFG_56	0	1	0	0	3	0	0	0	0
CDFG_70	0	0	0	0	0	1	0	0	0
CDFG_71	0	0	0	0	0	0	0	0	0
HF_41	0	0	0	0	0	0	0	0	0
HF_43	0	0	0	0	17	0	0	0	0
HF_44	0	0	0	0	8	0	0	0	0
HF_57	0	0	0	2	0	0	0	0	0
HF_58	0	1	0	2	2	0	0	0	0
HF_59	0	0	0	1	3	0	0	0	0
HF_60	0	3	0	1	0	1	0	0	0
HF_73	0	0	0	0	1	0	0	0	0
HFFG_45	0	0	0	0	6	1	0	0	0
HFFG_46	0	0	0	0	71	0	0	0	0
HFFG_47	0	0	0	0	1	0	0	0	0
HFFG_48	0	0	0	0	4	1	0	0	0
HFFG_61	2	0	0	3	0	0	0	0	0
HFFG_62	0	0	0	0	0	0	0	0	0
HFFG_75	0	0	0	0	1	0	0	0	0
HFFG_76	0	0	0	0	0	0	0	2	0

Kingdom	Bacteria	Bacteria	Bacteria	Bacteria	Bacteria	Bacteria	Bacteria	Bacteria	Bacteria	Bacteria
Phylum	Actinobacteria	Firmicutes	Firmicutes	Bacteroidetes	Firmicutes	Firmicutes	Firmicutes	Firmicutes	Firmicutes	Actinobacteria
Class	Actinobacteria	Clostridia	Clostridia	Bacteroidia	Clostridia	Clostridia	Bacilli	Clostridia	Bacilli	Actinobacteria
Order	Coriobacteriales	Clostridiales	Clostridiales	Bacteroidales	Clostridiales	Clostridiales	Lactobacillales	Clostridiales	Lactobacillales	Coriobacteriales
Family	Coriobacteriaceae	Lachnospiraceae	Lachnospiraceae	Porphyromonadaceae	Lachnospiraceae	Peptostreptococcaceae	Enterococcaceae	Clostridiales_Incertae_Sedis_XII	Aerococcaceae	Coriobacteriaceae
Genus	Enterorhabdus	Clostridium_XlVa	Clostridium_XlVa	Parabacteroides	Clostridium_XlVa	Romboutsia	Enterococcus	Guggenheimella	Abiotrophia	Senegalimassilia
#NAME	ZOTU_0436	ZOTU_0437	ZOTU_0438	ZOTU_0439	ZOTU_0440	ZOTU_0441	ZOTU_0442	ZOTU_0443	ZOTU_0444	ZOTU_0445
CD_49	0	0	0	0	0	0	1	0	0	0
CD_50	0	0	0	0	0	0	0	0	0	0
CD_52	0	0	0	0	0	0	0	0	0	2
CD_65	0	1	0	0	1	2	0	0	0	0
CD_67	0	1	0	0	0	0	0	0	0	0
CD_68	0	0	0	0	0	0	0	0	0	2
CD_78	0	0	0	0	0	0	0	0	0	0
CD_79	0	0	0	0	0	0	0	0	0	0
CD_80	0	0	0	0	0	0	1	0	0	0
CDFG_53	0	1	0	0	0	0	0	0	0	0
CDFG_54	0	0	0	0	2	0	0	0	0	5
CDFG_55	0	0	0	0	0	0	0	0	0	3
CDFG_56	0	0	0	0	0	0	12	0	0	0
CDFG_70	0	0	0	0	2	1	12	0	0	0
CDFG_71	0	0	0	0	0	0	4	0	0	0
HF_41	0	0	0	0	0	0	3	0	0	0
HF_43	0	0	0	0	0	0	1	0	0	0
HF_44	0	0	0	0	0	0	0	0	0	0
HF_57	0	0	0	0	0	1	0	0	0	0
HF_58	6	0	0	0	0	8	14	0	0	8
HF_59	0	0	0	0	0	3	4	0	0	0
HF_60	0	0	0	0	0	0	2	0	0	0
HF_73	0	0	0	0	0	1	7	0	0	0
HFFG_45	0	0	0	0	0	0	0	0	0	0
HFFG_46	0	0	0	0	0	0	0	0	0	0
HFFG_47	0	0	0	0	0	0	0	0	0	0
HFFG_48	0	0	0	0	0	0	6	0	0	0
HFFG_61	0	0	0	0	0	1	0	0	0	0
HFFG_62	0	0	0	0	0	0	0	0	0	0
HFFG_75	0	0	0	0	0	1	3	0	0	0
HFFG_76	1	1	0	0	0	4	24	0	0	0

Kingdom	Bacteria	Bacteria	Bacteria	Bacteria	Bacteria	Bacteria	Bacteria	Bacteria	Bacteria	Bacteria	Bacteria
Phylum	Firmicutes	Actinobacteria	Firmicutes	Actinobacteria	Proteobacteria	Firmicutes	Actinobacteria	Actinobacteria	Firmicutes	Firmicutes	Firmicutes
Class	Clostridia	Actinobacteria	Clostridia	Actinobacteria	Gammaproteobacteria	Clostridia	Actinobacteria	Actinobacteria	Clostridia	Bacilli	Bacilli
Order	Clostridiales	Actinomycetales	Clostridiales	Coriobacteriales	Legionellales	Clostridiales	Actinomycetales	Coriobacteriales	Clostridiales	Bacillales	Bacillales
Family	Lachnospiraceae	Micrococcaceae	Lachnospiraceae	Coriobacteriaceae	Legionellaceae	Lachnospiraceae	Micrococcaceae	Coriobacteriaceae	Peptostreptococcaceae	Paenibacillaceae_	Paenibacillaceae_
Genus	Blautia	Micrococcus	Clostridium_XlVa	Enterorhabdus	Legionella	Clostridium_XlVa	Rothia	Enterorhabdus	Clostridium_XI	Paenibacillus	Paenibacillus
#NAME	ZOTU_0446	ZOTU_0447	ZOTU_0448	ZOTU_0449	ZOTU_0450	ZOTU_0451	ZOTU_0452	ZOTU_0453	ZOTU_0454	ZOTU_0455	ZOTU_0456
CD_49	0	0	0	0	0	0	0	5	0	0	0
CD_50	0	0	0	0	0	0	0	2	0	0	0
CD_52	0	0	0	0	0	0	0	14	0	0	0
CD_65	0	0	0	0	0	0	0	7	0	0	0
CD_67	0	0	0	0	0	0	0	0	0	0	0
CD_68	0	0	0	0	0	0	1	8	0	0	0
CD_78	0	0	0	0	0	0	0	0	0	0	0
CD_79	0	0	0	0	0	0	0	0	0	0	0
CD_80	0	0	0	0	0	0	0	0	0	0	0
CDFG_53	0	0	0	0	0	0	0	1	0	0	0
CDFG_54	0	0	0	0	0	0	0	7	0	0	0
CDFG_55	0	0	0	0	0	0	0	1	0	1	0
CDFG_56	0	0	0	0	0	0	1	0	0	4	0
CDFG_70	0	0	0	0	0	0	0	0	0	0	0
CDFG_71	0	0	0	0	0	0	0	0	0	0	0
HF_41	0	0	0	0	0	0	0	0	0	0	0
HF_43	0	0	0	0	0	0	0	0	0	0	0
HF_44	0	0	0	0	0	0	0	0	0	0	0
HF_57	0	0	0	0	0	0	1	0	0	0	0
HF_58	0	0	0	0	0	0	1	0	0	0	0
HF_59	0	0	0	0	0	0	0	0	0	0	0
HF_60	0	0	0	0	0	0	1	0	0	0	0
HF_73	0	0	0	0	0	0	0	0	0	0	0
HFFG_45	0	0	0	0	0	0	0	0	0	1	0
HFFG_46	0	1	0	0	0	0	0	0	0	0	0
HFFG_47	0	0	0	0	0	0	0	0	0	0	0
HFFG_48	0	0	0	0	0	0	0	0	0	0	0
HFFG_61	0	0	0	0	0	0	1	0	0	0	0
HFFG_62	0	0	0	0	0	0	0	0	0	0	0
HFFG_75	0	0	0	0	0	0	0	0	0	0	0
HFFG_76	0	0	0	0	0	0	0	2	0	0	0

Kingdom	Bacteria	Bacteria	Bacteria	Bacteria
Phylum	Firmicutes	Firmicutes	Firmicutes	Firmicutes
Class	Bacilli	Clostridia	Bacilli	Erysipelotrichia
Order	Bacillales	Clostridiales	Bacillales	Erysipelotrichales
Family	Bacillaceae_	Clostridiaceae_	Paenibacillaceae_	Erysipelotrichaceae
Genus	Virgibacillus	Alkaliphilus	Paenibacillus	Kandleria
#NAME	ZOTU_0457	ZOTU_0458	ZOTU_0459	ZOTU_0460
CD_49	0	0	0	0
CD_50	0	0	0	0
CD_52	0	0	0	0
CD_65	0	0	4	0
CD_67	0	0	0	0
CD_68	0	0	1	0
CD_78	0	0	0	0
CD_79	0	0	0	0
CD_80	0	0	0	0
CDFG_53	0	0	0	0
CDFG_54	0	0	0	0
CDFG_55	0	0	0	0
CDFG_56	0	0	0	0
CDFG_70	0	0	0	0
CDFG_71	0	0	0	0
HF_41	0	0	0	0
HF_43	0	0	0	0
HF_44	0	0	0	0
HF_57	0	0	0	0
THF_58	0	0	0	0
HF_59	0	0	0	0
HF_60	0	0	0	0
HF_73	0	0	0	0
HFFG_45	9	0	0	0
HFFG_46	0	0	0	0
HFFG_47	0	0	0	0
HFFG_48	0	0	0	0
HFFG_61	0	0	0	0
HFFG_62	0	0	0	0
HFFG_75	0	0	0	0
HFFG_76	0	0	0	0

Table S12 (pages S-70 to S-124). Raw OTU count data from 16S sequencing of ileum contents.

Kingdom	Bacteria	Bacteria	Bacteria	Bacteria	Bacteria	Bacteria	Bacteria	Bacteria	Bacteria
Phylum	Firmicutes	Verrucomicrobia	Firmicutes	Firmicutes	Firmicutes	Firmicutes	Actinobacteria	Firmicutes	Firmicutes
Class	Erysipelotrichia	Verrucomicrobiae	Bacilli	Erysipelotrichia	Bacilli	Bacilli	Actinobacteria	Bacilli	Bacilli
Order	Erysipelotrichales	Verrucomicrobiales	Lactobacillales	Erysipelotrichales	Bacillales	Lactobacillales	Bifidobacteriales	Bacillales	Bacillales
Family	Erysipelotrichaceae	Verrucomicrobiaceae	Enterococcaceae	Erysipelotrichaceae	Staphylococcaceae	Lactobacillaceae	Bifidobacteriaceae	Bacillaceae_	Staphylococcaceae
Genus	Allobaculum	Akkermansia	Enterococcus	Catenisphaera	Staphylococcus	Lactobacillus	Bifidobacterium	Bacillus	Staphylococcus
#NAME	ZOTU_0001	ZOTU_0002	ZOTU_0003	ZOTU_0004	ZOTU_0005	ZOTU_0006	ZOTU_0007	ZOTU_0008	ZOTU_0009
CD_49	22013	9201	14274	29954	6092	6224	4792	87	0
CD_51	33500	12288	23972	1279	115767	384	2415	20	1
CD_52	55083	4047	3089	16295	25947	21037	10350	51	2
CD_65	4689	16202	733	10661	5132	1346	7013	165	0
CD_66	29053	31062	158	33931	486	9423	22649	423	0
CD_67	31789	9237	6009	22094	32312	1004	9731	290	0
CD_68	19571	8401	636	15269	757	84	3571	88510	0
CD_78	14864	29939	3669	2898	30297	3	428	14	0
CD_79	34	200	82	9	83342	1	3	10	0
CDFG_53	37617	51532	1619	1399	27330	10502	12841	25	0
CDFG_56	15000	66782	41859	459	7952	613	1662	328	0
CDFG_69	40197	30761	1246	11908	3822	269	9747	1698	0
CDFG_70	11998	97609	2155	1517	19737	753	11246	2096	0
CDFG_71	10952	18794	311	1159	37428	30	877	14	0
HF_41	9400	3523	5341	13438	2831	1797	968	199	5
HF_42	2617	1820	33970	1975	1316	219	210	194	7
HF_43	2469	2287	760	21164	1052	14398	120	71	2
HF_57	6274	2621	1047	12664	1290	382	2350	551	0
HF_58	7460	13793	1075	6532	4474	4950	1734	5251	0
HF_72	1452	5310	2807	11960	10962	4039	568	127	0
HF_73	5492	3388	20328	24811	110471	16897	1780	395	0
HF_74	8729	658	557	6961	2033	34955	713	827	0
HFFG_45	4040	18517	462	2545	1565	6045	405	47	36
HFFG_48	9449	21793	19048	2949	6042	1071	2184	87	1
HFFG_62	4425	60472	5440	6709	5732	3371	1191	1798	0
HFFG_77	20212	16144	14193	7374	30431	6059	960	10133	0

Kingdom	Bacteria	Bacteria	Bacteria	Bacteria	Bacteria	Bacteria	Bacteria	Bacteria
Phylum	Firmicutes	Firmicutes	Bacteroidetes	Firmicutes	Firmicutes	Actinobacteria	Firmicutes	Firmicutes
Class	Clostridia	Bacilli	Bacteroidia	Clostridia	Clostridia	Actinobacteria	Bacilli	Clostridia
Order	Clostridiales	Lactobacillales	Bacteroidales	Clostridiales	Clostridiales	Coriobacteriales	Bacillales	Clostridiales
Family	Peptostreptococcaceae	Streptococcaceae	Bacteroidaceae	Lachnospiraceae	Lachnospiraceae	Coriobacteriaceae	Staphylococcaceae	Lachnospiraceae
Genus	Romboutsia	Lactococcus	Bacteroides	Clostridium_XlVa	Acetatifactor	Enterorhabdus	Staphylococcus	Clostridium_XlVa
#NAME	ZOTU_0010	ZOTU_0011	ZOTU_0012	ZOTU_0013	ZOTU_0014	ZOTU_0015	ZOTU_0016	ZOTU_0017
CD_49	4318	260	10	0	32	91	3	4
CD_51	703	1449	4	0	3	1705	0	0
CD_52	18	181	1	0	2	315	227	2
CD_65	25	1359	7	0	28	83	0	0
CD_66	13	985	0	1	16	278	0	0
CD_67	16	193	0	0	4	278	1	0
CD_68	0	107	2	0	15	256	8	0
CD_78	6	43	0	0	0	290	1	0
CD_79	11	2	0	0	0	0	0	0
CDFG_53	382	213	1	0	2	391	2	15
CDFG_56	80	143	0	0	2	226	4	1
CDFG_69	499	1318	0	0	79	379	0	10
CDFG_70	1442	3564	33	0	42	767	0	542
CDFG_71	39	389	0	0	16	165	0	2
HF_41	1321	351	0	52	8	19	8	4
HF_42	41	199	0	80	13	33	6	2
HF_43	1981	2051	1	92	104	409	4	2
HF_57	51	158	0	0	76	11	2	0
HF_58	184	876	0	0	577	603	0	0
HF_72	663	5616	0	4	270	78	4	5
HF_73	608	1056	2	2	71	272	1	3
HF_74	2307	1114	1	2	4	106	0	0
HFFG_45	5478	7181	3	4	6	186	4	2
HFFG_48	763	351	0	1	11	137	85	4
HFFG_62	240	2953	0	6	29	444	7	48
HFFG_77	750	933	1	4	61	248	2	1

Kingdom	Bacteria	Bacteria	Bacteria	Bacteria	Bacteria	Bacteria	Bacteria	Bacteria
Phylum	Firmicutes	Firmicutes	Bacteroidetes	Firmicutes	Firmicutes	Bacteroidetes	Proteobacteria	Firmicutes
Class	Clostridia	Clostridia	Bacteroidia	Erysipelotrichia	Clostridia	Bacteroidia	Betaproteobacteria	Clostridia
Order	Clostridiales	Clostridiales	Bacteroidales	Erysipelotrichales	Clostridiales	Bacteroidales	Burkholderiales	Clostridiales
Family	Clostridiaceae_	Peptostreptococcaceae	Porphyromonadaceae	Erysipelotrichaceae	Ruminococcaceae	Rikenellaceae	Sutterellaceae	Lachnospiraceae
Genus	Clostridium_sensu_stricto	Romboutsia	Barnesiella	Allobaculum	Pseudoflavonifractor	Alistipes	Parasutterella	Clostridium_XlVa
#NAME	ZOTU_0018	ZOTU_0019	ZOTU_0020	ZOTU_0021	ZOTU_0022	ZOTU_0023	ZOTU_0024	ZOTU_0025
CD_49	229	912	76	0	1	5	1420	0
CD_51	318	143	564	0	0	0	61	0
CD_52	1	4	4	0	1	0	375	7
CD_65	20	8	6	1	3	0	328	2
CD_66	7	1	6	0	2	1	331	5
CD_67	9	4	40	0	2	14	26	0
CD_68	10	0	32	0	0	2	680	0
CD_78	1	1	3	2	0	0	3	0
CD_79	0	1	0	0	0	0	0	0
CDFG_53	333	158	14	0	2	1	0	1
CDFG_56	90	27	5	2	0	0	1	0
CDFG_69	44	153	68	0	6	0	22	1
CDFG_70	9	347	226	6	23	10	22	32
CDFG_71	5	12	9	0	4	0	3	1
HF_41	69	378	9	1	5	0	214	1
HF_42	4	10	4	2	0	0	11	4
HF_43	119	791	3	0	3	1	35	14
HF_57	2	17	0	1	0	0	33	0
HF_58	9	95	1	2	0	0	20	3
HF_72	29	226	3	60	0	0	9	7
HF_73	9	242	0	3	0	0	30	4
HF_74	4	938	4	0	0	0	142	0
HFFG_45	271	2417	6	0	10	0	9	7
HFFG_48	649	363	3	34	7	1	4	2
HFFG_62	47	83	4	4	5	0	40	36
HFFG_77	21	318	2	33	2	0	16	2

Kingdom	Bacteria	Bacteria	Bacteria	Bacteria	Bacteria	Bacteria	Bacteria	Bacteria
Phylum	Actinobacteria	Firmicutes	Bacteroidetes	Firmicutes	Firmicutes	Firmicutes	Bacteroidetes	Firmicutes
Class	Actinobacteria	Clostridia	Bacteroidia	Clostridia	Bacilli	Erysipelotrichia	Bacteroidia	Clostridia
Order	Coriobacteriales	Clostridiales	Bacteroidales	Clostridiales	Bacillales	Erysipelotrichales	Bacteroidales	Clostridiales
Family	Coriobacteriaceae	Eubacteriaceae	Porphyromonadaceae	Ruminococcaceae	Planococcaceae	Erysipelotrichaceae	Porphyromonadaceae	Lachnospiraceae
Genus	Enterorhabdus	Eubacterium	Parabacteroides	Pseudoflavonifractor	Lysinibacillus	Turicibacter	Barnesiella	Clostridium_XlVa
#NAME	ZOTU_0026	ZOTU_0027	ZOTU_0028	ZOTU_0029	ZOTU_0030	ZOTU_0031	ZOTU_0032	ZOTU_0033
CD_49	368	0	0	0	0	1	0	0
CD_51	129	0	0	0	0	1	0	3
CD_52	222	0	0	0	0	0	0	0
CD_65	86	0	17	0	0	1	0	10
CD_66	170	0	30	0	0	1	0	0
CD_67	704	0	0	0	0	0	0	0
CD_68	210	0	101	0	0	0	0	0
CD_78	23	0	0	0	0	0	0	0
CD_79	0	0	0	0	0	0	0	0
CDFG_53	284	0	0	0	0	0	40	2
CDFG_56	60	0	5	0	0	0	30	0
CDFG_69	826	0	1	0	2	131	0	4
CDFG_70	562	0	1	4	0	368	0	30
CDFG_71	168	0	1	1	0	12	0	7
HF_41	51	0	1	0	0	0	0	1
HF_42	4	0	0	2	0	2	3	0
HF_43	69	0	0	34	1	1	87	35
HF_57	11	0	1	0	0	2	0	0
HF_58	125	1	1	0	1	2	2	0
HF_72	121	0	4	0	0	4	33	1
HF_73	769	0	0	1	0	2	6	1
HF_74	378	0	0	0	0	2	141	15
HFFG_45	28	1	3	0	3	269	0	21
HFFG_48	40	1	21	0	4	7	32	2
HFFG_62	146	0	59	0	2	22	0	26
HFFG_77	41	0	1	0	0	70	0	2

Kingdom	Bacteria	Bacteria	Bacteria	Bacteria	Bacteria	Bacteria	Bacteria	Bacteria	Bacteria
Phylum	Firmicutes	Actinobacteria	Bacteroidetes	Firmicutes	Firmicutes	Firmicutes	Actinobacteria	Firmicutes	Firmicutes
Class	Erysipelotrichia	Actinobacteria	Bacteroidia	Clostridia	Clostridia	Clostridia	Actinobacteria	Clostridia	Clostridia
Order	Erysipelotrichales	Coriobacteriales	Bacteroidales	Clostridiales	Clostridiales	Clostridiales	Coriobacteriales	Clostridiales	Clostridiales
Family	Erysipelotrichaceae	Coriobacteriaceae	Porphyromonadaceae	Clostridiaceae_	Lachnospiraceae	Lachnospiraceae	Coriobacteriaceae	Lachnospiraceae	Lachnospiraceae
Genus	Clostridium_XVIII	Parvibacter	Barnesiella	Alkaliphilus	Clostridium_XlVa	Clostridium_XlVa	Senegalimassilia	Clostridium_XlVa	Clostridium_XlVa
#NAME	ZOTU_0034	ZOTU_0035	ZOTU_0036	ZOTU_0037	ZOTU_0038	ZOTU_0039	ZOTU_0040	ZOTU_0041	ZOTU_0042
CD_49	1	27	8	3	0	8	1	2	58
CD_51	0	140	30	2	0	0	215	0	9
CD_52	0	79	4	0	0	1	14	0	2
CD_65	3	4	25	1	3	198	4	0	68
CD_66	0	36	29	0	0	57	36	1	32
CD_67	0	27	5	0	0	5	49	0	2
CD_68	0	19	11	0	0	0	17	0	7
CD_78	0	20	0	0	0	1	59	0	0
CD_79	0	0	1	0	0	0	0	0	0
CDFG_53	0	347	15	0	0	34	448	1	2
CDFG_56	0	103	4	0	1	8	187	0	0
CDFG_69	3	507	77	0	2	26	342	5	1
CDFG_70	7	338	152	0	83	0	292	39	30
CDFG_71	0	61	17	0	3	3	148	5	10
HF_41	0	5	6	8	0	7	5	3	11
HF_42	1	3	2	2	1	105	3	0	0
HF_43	5	44	35	2	25	202	157	0	0
HF_57	1	2	0	2	0	26	2	0	4
HF_58	0	66	2	0	1	20	41	0	18
HF_72	0	21	6	0	74	206	77	1	71
HF_73	0	68	2	2	12	32	94	0	6
HF_74	2	29	0	0	0	14	8	0	2
HFFG_45	1	21	20	0	0	1	43	2	63
HFFG_48	0	51	10	2	0	5	134	2	7
HFFG_62	2	16	56	0	7	106	29	2	28
HFFG_77	0	25	5	0	0	139	53	3	11

Kingdom	Bacteria	Bacteria	Bacteria	Bacteria	Bacteria	Bacteria	Bacteria	Bacteria	Bacteria
Phylum	Firmicutes	Firmicutes	Actinobacteria	Firmicutes	Firmicutes	Firmicutes	Firmicutes	Bacteroidetes	Firmicutes
Class	Clostridia	Clostridia	Actinobacteria	Bacilli	Clostridia	Clostridia	Clostridia	Bacteroidia	Clostridia
Order	Clostridiales	Clostridiales	Coriobacteriales	Bacillales	Clostridiales	Clostridiales	Clostridiales	Bacteroidales	Clostridiales
Family	Lachnospiraceae	Lachnospiraceae	Coriobacteriaceae	Staphylococcaceae	Lachnospiraceae	Ruminococcaceae	Lachnospiraceae	Porphyromonadaceae	Clostridiaceae_
Genus	Clostridium_XlVa	Blautia	Asaccharobacter	Staphylococcus	Acetatifactor	Oscillibacter	Clostridium_XlVa	Parabacteroides	Alkaliphilus
#NAME	ZOTU_0043	ZOTU_0044	ZOTU_0045	ZOTU_0046	ZOTU_0047	ZOTU_0048	ZOTU_0049	ZOTU_0050	ZOTU_0051
CD_49	0	0	0	8	15	0	2	0	9
CD_51	0	3	0	1406	2	0	0	0	10
CD_52	0	0	0	13	2	0	1	1	9
CD_65	0	0	0	1	91	0	6	101	7
CD_66	0	0	6	2	55	0	2	24	1
CD_67	0	0	1	57	3	0	0	119	6
CD_68	0	0	1	3	0	0	0	76	7
CD_78	0	0	0	120	1	0	0	0	0
CD_79	0	0	0	74	0	0	0	0	1
CDFG_53	8	1	0	9	26	0	1	0	5
CDFG_56	2	1	2	176	2	0	0	0	5
CDFG_69	1	11	0	3	32	2	4	0	3
CDFG_70	167	127	2	274	675	3	246	0	4
CDFG_71	18	2	0	14	45	0	3	0	0
HF_41	0	0	0	11	21	0	2	1	3
HF_42	0	0	5	3	5	0	0	2	1
HF_43	1	2	3	2	300	0	2	0	3
HF_57	0	0	1	3	8	0	0	3	0
HF_58	0	0	20	19	6	0	0	2	6
HF_72	3	3	3	3793	113	0	3	0	0
HF_73	0	1	23	121	21	0	0	3	1
HF_74	0	0	2	9	4	0	0	1	0
HFFG_45	0	25	6	13	51	0	4	0	17
HFFG_48	3	3	6	988	4	0	1	0	6
HFFG_62	0	5	211	49	99	0	3	0	2
HFFG_77	3	2	31	456	12	0	0	1	0

Kingdom	Bacteria	Bacteria	Bacteria	Bacteria	Bacteria	Bacteria	Bacteria	Bacteria	Bacteria
Phylum	Firmicutes	Firmicutes	Firmicutes	Bacteroidetes	Firmicutes	Firmicutes	Firmicutes	Firmicutes	Firmicutes
Class	Clostridia	Clostridia	Clostridia	Bacteroidia	Clostridia	Clostridia	Clostridia	Clostridia	Clostridia
Order	Clostridiales	Clostridiales	Clostridiales	Bacteroidales	Clostridiales	Clostridiales	Clostridiales	Clostridiales	Clostridiales
Family	Clostridiaceae_	Ruminococcaceae	Ruminococcaceae	Porphyromonadaceae	Lachnospiraceae	Lachnospiraceae	Ruminococcaceae	Lachnospiraceae	Clostridiaceae_
Genus	Alkaliphilus	Flavonifractor	Clostridium_IV	Barnesiella	Clostridium_XlVa	Acetatifactor	Oscillibacter	Ruminococcus	Alkaliphilus
#NAME	ZOTU_0052	ZOTU_0053	ZOTU_0054	ZOTU_0055	ZOTU_0056	ZOTU_0057	ZOTU_0058	ZOTU_0059	ZOTU_0060
CD_49	1	0	0	32	0	2	0	2	0
CD_51	0	0	0	58	0	6	0	0	0
CD_52	1	0	0	4	0	0	0	0	0
CD_65	0	0	0	4	26	22	0	20	0
CD_66	0	0	1	6	31	28	0	1	0
CD_67	0	0	0	5	0	0	0	0	0
CD_68	0	0	0	7	0	6	0	0	0
CD_78	0	0	0	0	0	1	0	0	0
CD_79	0	0	0	0	0	0	0	0	0
CDFG_53	51	0	0	8	0	0	0	0	0
CDFG_56	1	0	0	3	0	3	0	0	0
CDFG_69	1	2	1	0	2	32	1	0	0
CDFG_70	36	77	8	0	349	680	8	40	0
CDFG_71	0	1	0	0	0	22	0	0	0
HF_41	0	0	0	3	2	3	0	0	0
HF_42	0	0	0	1	0	85	0	2	13
HF_43	0	1	0	14	0	112	0	15	0
HF_57	0	0	0	0	2	4	0	0	0
HF_58	0	0	0	3	1	7	0	5	0
HF_72	3	0	0	2	0	235	0	10	0
HF_73	0	0	0	1	3	52	0	4	0
HF_74	1	0	0	32	1	17	0	0	0
HFFG_45	3	1	0	0	17	3	0	0	0
HFFG_48	3	0	0	8	0	3	0	0	0
HFFG_62	6	2	3	3	1	40	0	3	0
HFFG_77	0	0	0	3	1	18	0	1	0

Kingdom	Bacteria	Bacteria	Bacteria	Bacteria	Bacteria	Bacteria	Bacteria	Bacteria	Bacteria
Phylum	Firmicutes	Firmicutes	Bacteroidetes	Firmicutes	Firmicutes	Firmicutes	Actinobacteria	Firmicutes	Firmicutes
Class	Clostridia	Clostridia	Bacteroidia	Clostridia	Clostridia	Clostridia	Actinobacteria	Clostridia	Clostridia
Order	Clostridiales	Clostridiales	Bacteroidales	Clostridiales	Clostridiales	Clostridiales	Coriobacteriales	Clostridiales	Clostridiales
Family	Lachnospiraceae	Lachnospiraceae	Porphyromonadaceae	Lachnospiraceae	Ruminococcaceae	Lachnospiraceae	Coriobacteriaceae	Lachnospiraceae	Ruminococcaceae
Genus	Blautia	Clostridium_XlVa	Barnesiella	Acetatifactor	Clostridium_III	Acetatifactor	Paraeggerthella	Clostridium_XlVa	Ruminococcus
#NAME	ZOTU_0061	ZOTU_0062	ZOTU_0063	ZOTU_0064	ZOTU_0065	ZOTU_0066	ZOTU_0067	ZOTU_0068	ZOTU_0069
CD_49	0	0	31	1	6	2	8	1	0
CD_51	0	0	67	1	41	0	81	8	3
CD_52	23	2	3	2	2	3	21	1	1
CD_65	0	2	3	3	18	26	12	0	9
CD_66	0	1	4	3	12	13	24	0	3
CD_67	0	0	8	1	1	0	8	0	1
CD_68	0	0	9	0	12	2	23	0	0
CD_78	0	0	0	0	0	0	3	0	0
CD_79	0	0	0	0	0	0	0	0	0
CDFG_53	12	3	6	1	42	4	144	4	49
CDFG_56	0	0	2	1	16	1	31	2	4
CDFG_69	1	8	0	4	1	13	63	0	11
CDFG_70	278	71	0	229	2	62	330	8	185
CDFG_71	10	3	0	17	0	7	12	6	18
HF_41	2	0	2	0	3	2	3	0	0
HF_42	0	0	1	0	0	1	1	1	0
HF_43	36	0	9	7	2	63	16	5	17
HF_57	0	0	0	0	3	5	0	0	1
HF_58	0	0	0	0	21	25	26	8	2
HF_72	2	35	0	30	17	30	81	5	3
HF_73	1	7	2	9	8	2	27	3	2
HF_74	0	2	28	0	30	1	10	3	10
HFFG_45	11	34	1	6	261	233	130	8	63
HFFG_48	0	3	5	56	32	21	31	0	1
HFFG_62	1	41	0	351	493	23	260	1	7
HFFG_77	21	10	0	17	4	3	13	0	2

Kingdom	Bacteria	Bacteria	Bacteria	Bacteria	Bacteria	Bacteria	Bacteria	Bacteria	Bacteria
Phylum	Firmicutes	Firmicutes	Firmicutes	Firmicutes	Firmicutes	Firmicutes	Firmicutes	Firmicutes	Firmicutes
Class	Clostridia	Clostridia	Clostridia	Clostridia	Clostridia	Clostridia	Clostridia	Clostridia	Clostridia
Order	Clostridiales	Clostridiales	Clostridiales	Clostridiales	Clostridiales	Clostridiales	Clostridiales	Clostridiales	Clostridiales
Family	Ruminococcaceae	Lachnospiraceae	Lachnospiraceae	Lachnospiraceae	Catabacteriaceae	Lachnospiraceae	Ruminococcaceae	Ruminococcaceae	Lachnospiraceae
Genus	Clostridium_IV	Clostridium_XlVa	Clostridium_XlVa	Acetatifactor	Catabacter	Clostridium_XlVa	Clostridium_III	Sporobacter	Acetatifactor
#NAME	ZOTU_0070	ZOTU_0071	ZOTU_0072	ZOTU_0073	ZOTU_0074	ZOTU_0075	ZOTU_0076	ZOTU_0077	ZOTU_0078
CD_49	0	0	0	3	1	0	0	0	0
CD_51	0	0	0	2	0	0	0	0	0
CD_52	2	0	0	2	0	0	0	0	0
CD_65	7	0	0	22	0	0	0	8	11
CD_66	2	0	0	19	0	0	0	2	35
CD_67	0	0	0	0	0	0	0	0	0
CD_68	0	0	0	0	0	0	0	0	16
CD_78	0	0	0	0	0	0	0	0	0
CD_79	0	0	0	0	0	0	0	0	0
CDFG_53	98	4	0	9	0	0	0	1	0
CDFG_56	6	0	0	0	0	0	0	0	0
CDFG_69	0	4	2	10	0	0	0	1	7
CDFG_70	117	346	3	222	3	1	2	162	27
CDFG_71	0	0	0	11	0	0	0	1	0
HF_41	0	0	0	7	0	0	0	0	2
HF_42	1	0	0	1	0	0	0	3	13
HF_43	8	0	0	121	0	0	24	11	42
HF_57	0	0	0	4	0	0	0	1	7
HF_58	0	0	1	3	0	0	0	0	25
HF_72	0	0	0	46	0	0	2	3	45
HF_73	0	0	0	7	0	0	0	6	14
HF_74	2	0	0	0	0	0	0	2	4
HFFG_45	1	24	0	14	0	0	0	0	0
HFFG_48	6	4	0	1	0	0	2	0	0
HFFG_62	7	0	0	27	0	0	0	3	3
HFFG_77	4	1	0	5	0	0	0	0	6

Kingdom	Bacteria	Bacteria	Bacteria	Bacteria	Bacteria	Bacteria	Bacteria	Bacteria	Bacteria
Phylum	Firmicutes	Bacteroidetes	Firmicutes	Firmicutes	Firmicutes	Firmicutes	Firmicutes	Firmicutes	Firmicutes
Class	Clostridia	Bacteroidia	Clostridia	Clostridia	Clostridia	Clostridia	Clostridia	Clostridia	Clostridia
Order	Clostridiales	Bacteroidales	Clostridiales	Clostridiales	Clostridiales	Clostridiales	Clostridiales	Clostridiales	Clostridiales
Family	Lachnospiraceae	Porphyromonadaceae	Ruminococcaceae	Ruminococcaceae	Ruminococcaceae	Clostridiaceae_	Ruminococcaceae	Lachnospiraceae	Lachnospiraceae
Genus	Murimonas	Coprobacter	Oscillibacter	Clostridium_IV	Pseudoflavonifractor	Alkaliphilus	Clostridium_IV	Acetatifactor	Acetatifactor
#NAME	ZOTU_0079	ZOTU_0080	ZOTU_0081	ZOTU_0082	ZOTU_0083	ZOTU_0084	ZOTU_0085	ZOTU_0086	ZOTU_0087
CD_49	1	0	2	0	0	0	0	2	0
CD_51	1	0	0	0	1	0	0	0	0
CD_52	7	0	0	0	0	0	0	0	0
CD_65	32	14	0	1	0	0	0	16	0
CD_66	8	16	0	0	3	0	0	8	0
CD_67	0	27	0	0	0	0	0	0	0
CD_68	0	13	0	0	0	0	0	1	0
CD_78	0	0	0	0	0	0	0	0	0
CD_79	0	0	0	0	0	0	0	0	0
CDFG_53	23	0	0	0	0	0	0	1	0
CDFG_56	3	0	0	0	0	0	0	0	0
CDFG_69	9	0	0	1	1	0	0	0	1
CDFG_70	73	0	2	1	5	4	0	2	70
CDFG_71	15	0	0	0	1	2	0	0	1
HF_41	1	1	0	0	0	0	0	1	3
HF_42	0	0	0	0	0	0	0	1	1
HF_43	28	0	1	0	0	0	0	19	0
HF_57	3	0	0	0	0	0	0	4	0
HF_58	2	0	0	0	0	0	0	18	1
HF_72	8	4	0	2	2	0	0	1	0
HF_73	9	2	0	0	5	1	0	6	0
HF_74	23	14	0	1	0	0	0	0	0
HFFG_45	3	4	0	0	0	26	0	103	0
HFFG_48	3	1	0	0	0	0	0	15	0
HFFG_62	20	7	0	7	1	1	0	2	0
HFFG_77	15	1	0	0	0	2	0	4	0

Kingdom	Bacteria	Bacteria	Bacteria	Bacteria	Bacteria	Bacteria	Bacteria	Bacteria	Bacteria
Phylum	Firmicutes	Firmicutes	Firmicutes	Bacteroidetes	Firmicutes	Firmicutes	Firmicutes	Firmicutes	Firmicutes
Class	Clostridia	Clostridia	Clostridia	Bacteroidia	Clostridia	Clostridia	Clostridia	Clostridia	Clostridia
Order	Clostridiales	Clostridiales	Clostridiales	Bacteroidales	Clostridiales	Clostridiales	Clostridiales	Clostridiales	Clostridiales
Family	Lachnospiraceae	Lachnospiraceae	Eubacteriaceae	Porphyromonadaceae	Ruminococcaceae	Lachnospiraceae	Ruminococcaceae	Lachnospiraceae	Lachnospiraceae
Genus	Blautia	Clostridium_XlVa	Eubacterium	Barnesiella	Saccharofermentans	Clostridium_XlVa	Clostridium_IV	Anaerostipes	Clostridium_XlVb
#NAME	ZOTU_0088	ZOTU_0089	ZOTU_0090	ZOTU_0091	ZOTU_0092	ZOTU_0093	ZOTU_0094	ZOTU_0095	ZOTU_0096
CD_49	0	2	0	11	0	0	0	0	1
CD_51	1	0	0	20	0	1	0	0	2
CD_52	0	0	0	1	0	0	0	0	0
CD_65	0	11	0	10	0	1	13	0	0
CD_66	0	7	0	11	0	1	4	0	0
CD_67	0	0	0	0	0	0	1	0	0
CD_68	0	0	0	1	0	1	0	0	0
CD_78	0	0	0	0	0	0	0	0	0
CD_79	0	0	0	0	0	0	0	0	0
CDFG_53	0	0	0	3	1	1	2	6	0
CDFG_56	0	0	0	0	0	0	1	3	0
CDFG_69	7	0	0	2	0	1	10	9	7
CDFG_70	25	1	0	22	1	31	16	203	10
CDFG_71	0	0	0	1	0	3	2	0	0
HF_41	0	0	0	3	0	3	0	0	0
HF_42	0	0	5	0	0	0	0	0	1
HF_43	2	34	35	2	0	6	37	0	4
HF_57	0	4	0	0	0	1	3	0	1
HF_58	0	2	0	0	0	2	1	0	0
HF_72	1	3	2	0	0	6	10	0	0
HF_73	0	1	0	6	0	0	11	0	0
HF_74	0	1	0	0	0	3	9	0	0
HFFG_45	8	1	0	5	0	11	4	7	1
HFFG_48	0	1	0	0	0	0	1	0	2
HFFG_62	0	13	0	1	0	5	7	16	0
HFFG_77	0	0	0	0	0	5	0	0	0

Kingdom	Bacteria	Bacteria	Bacteria	Bacteria	Bacteria	Bacteria	Bacteria	Bacteria	Bacteria
Phylum	Firmicutes	Firmicutes	Firmicutes	Firmicutes	Firmicutes	Firmicutes	Firmicutes	Firmicutes	Firmicutes
Class	Clostridia	Clostridia	Clostridia	Clostridia	Clostridia	Clostridia	Clostridia	Clostridia	Clostridia
Order	Clostridiales	Clostridiales	Clostridiales	Clostridiales	Clostridiales	Clostridiales	Clostridiales	Clostridiales	Clostridiales
Family	Clostridiaceae_	Ruminococcaceae	Lachnospiraceae	Ruminococcaceae	Lachnospiraceae	Lachnospiraceae	Lachnospiraceae	Lachnospiraceae	Lachnospiraceae
Genus	Alkaliphilus	Clostridium_IV	Clostridium_XlVa	Pseudoflavonifractor	Acetatifactor	Clostridium_XlVa	Roseburia	Marvinbryantia	Clostridium_XlVa
#NAME	ZOTU_0097	ZOTU_0098	ZOTU_0099	ZOTU_0100	ZOTU_0101	ZOTU_0102	ZOTU_0103	ZOTU_0104	ZOTU_0105
CD_49	0	0	0	0	1	0	0	0	0
CD_51	0	0	0	0	0	0	0	0	0
CD_52	0	0	0	0	0	0	0	0	0
CD_65	0	0	22	0	6	1	1	0	0
CD_66	0	0	14	0	3	1	1	0	0
CD_67	0	0	0	0	0	0	0	0	0
CD_68	0	0	0	0	0	0	0	0	0
CD_78	0	0	0	0	0	0	0	0	0
CD_79	0	0	0	0	0	0	0	0	0
CDFG_53	0	1	0	0	0	0	0	0	0
CDFG_56	0	1	0	1	0	0	0	0	0
CDFG_69	0	0	0	0	0	0	0	0	0
CDFG_70	0	0	1	4	2	0	35	1	0
CDFG_71	0	0	0	0	2	1	4	0	0
HF_41	0	0	0	0	1	0	0	0	0
HF_42	0	0	0	0	0	0	0	0	0
HF_43	0	0	0	0	16	1	2	0	0
HF_57	0	0	2	0	4	0	0	0	0
HF_58	0	0	0	0	23	0	0	0	0
HF_72	0	0	1	0	6	0	0	0	0
HF_73	0	0	0	0	0	0	0	0	0
HF_74	0	0	1	0	1	0	0	0	0
HFFG_45	0	0	0	0	139	0	0	0	0
HFFG_48	0	0	0	0	14	0	0	0	0
HFFG_62	0	0	5	2	3	0	2	0	0
HFFG_77	0	0	1	0	2	0	0	0	0

Kingdom	Bacteria	Bacteria	Bacteria	Bacteria	Bacteria	Bacteria	Bacteria	Bacteria	Bacteria
Phylum	Actinobacteria	Firmicutes	Firmicutes	Firmicutes	Firmicutes	Firmicutes	Firmicutes	Firmicutes	Firmicutes
Class	Actinobacteria	Clostridia	Clostridia	Clostridia	Clostridia	Clostridia	Clostridia	Clostridia	Clostridia
Order	Actinomycetales	Clostridiales	Clostridiales	Clostridiales	Clostridiales	Clostridiales	Clostridiales	Clostridiales	Clostridiales
Family	Mycobacteriaceae	Ruminococcaceae	Ruminococcaceae	Clostridiaceae_	Lachnospiraceae	Ruminococcaceae	Lachnospiraceae	Lachnospiraceae	Lachnospiraceae
Genus	Mycobacterium	Clostridium_IV	Flavonifractor	Alkaliphilus	Acetatifactor	Oscillibacter	Blautia	Clostridium_XlVa	Clostridium_XlVa
#NAME	ZOTU_0106	ZOTU_0107	ZOTU_0108	ZOTU_0109	ZOTU_0110	ZOTU_0111	ZOTU_0112	ZOTU_0113	ZOTU_0114
CD_49	0	0	0	0	1	0	0	0	2
CD_51	0	0	0	0	0	0	0	0	6
CD_52	0	0	0	0	0	0	10	0	1
CD_65	0	0	0	0	0	2	0	1	0
CD_66	0	0	0	0	0	0	0	0	4
CD_67	0	0	0	0	0	0	0	0	0
CD_68	0	0	0	0	0	0	0	0	0
CD_78	0	0	0	0	0	0	0	0	0
CD_79	0	0	0	0	0	0	0	0	0
CDFG_53	0	0	0	0	0	0	5	5	0
CDFG_56	1	0	0	0	0	0	0	2	0
CDFG_69	0	0	4	0	0	0	0	1	4
CDFG_70	0	0	11	0	0	0	94	57	0
CDFG_71	0	0	0	0	0	0	5	3	5
HF_41	0	0	1	0	0	0	0	0	1
HF_42	1	1	0	0	0	2	0	0	1
HF_43	6	0	0	0	0	4	0	1	10
HF_57	0	0	0	0	0	0	0	0	0
HF_58	0	0	0	1	0	0	0	1	0
HF_72	0	0	0	0	0	0	0	0	2
HF_73	0	0	0	0	1	0	0	0	0
HF_74	0	0	0	0	0	0	1	0	0
HFFG_45	21	0	0	127	0	0	2	11	177
HFFG_48	5	0	0	0	0	0	0	1	3
HFFG_62	0	0	0	0	0	0	0	7	50
HFFG_77	0	0	0	0	0	0	2	5	9

Kingdom	Bacteria	Bacteria	Bacteria	Bacteria	Bacteria	Bacteria	Bacteria	Bacteria
Phylum	Firmicutes	Firmicutes	Firmicutes	Firmicutes	Firmicutes	Firmicutes	Firmicutes	Firmicutes
Class	Clostridia	Clostridia	Bacilli	Clostridia	Clostridia	Clostridia	Clostridia	Clostridia
Order	Clostridiales	Clostridiales	Bacillales	Clostridiales	Clostridiales	Clostridiales	Clostridiales	Clostridiales
Family	Lachnospiraceae	Lachnospiraceae	Bacillaceae_	Lachnospiraceae	Clostridiales_Incertae_Sedis_XII	Ruminococcaceae	Lachnospiraceae	Lachnospiraceae
Genus	Clostridium_XlVa	Clostridium_XlVa	Bacillus	Clostridium_XlVa	Guggenheimella	Clostridium_IV	Clostridium_XlVa	Lachnospiracea_incertae_sedis
#NAME	ZOTU_0115	ZOTU_0116	ZOTU_0117	ZOTU_0118	ZOTU_0119	ZOTU_0120	ZOTU_0121	ZOTU_0122
CD_49	0	0	18	0	0	0	0	0
CD_51	0	0	8	0	0	0	1	2
CD_52	0	0	6	0	0	0	1	0
CD_65	1	0	2	0	0	3	62	0
CD_66	0	0	5	0	0	0	57	0
CD_67	0	0	1204	0	0	0	4	0
CD_68	0	0	0	0	0	0	8	0
CD_78	0	0	0	0	0	0	0	0
CD_79	0	0	0	0	0	0	0	0
CDFG_53	0	2	0	0	0	0	11	0
CDFG_56	0	0	1	0	0	0	1	0
CDFG_69	0	7	4	0	0	0	17	0
CDFG_70	1	126	1	0	0	22	36	0
CDFG_71	0	0	0	0	0	0	7	0
HF_41	0	0	9	0	0	0	0	0
HF_42	0	0	3	0	0	1	1	0
HF_43	0	0	0	0	0	5	51	15
HF_57	0	0	11	0	0	0	10	0
HF_58	0	0	66	0	0	0	2	1
HF_72	0	0	0	0	0	0	39	25
HF_73	0	0	1	0	0	0	12	2
HF_74	1	0	2	0	0	0	15	9
HFFG_45	2	15	0	0	0	7	93	0
HFFG_48	0	2	1	0	0	1	5	0
HFFG_62	0	0	0	0	0	0	68	0
HFFG_77	0	0	11	0	0	0	31	0

Kingdom	Bacteria	Bacteria	Bacteria	Bacteria	Bacteria	Bacteria	Bacteria	Bacteria	Bacteria
Phylum	Firmicutes	Firmicutes	Firmicutes	Firmicutes	Firmicutes	Firmicutes	Firmicutes	Firmicutes	Firmicutes
Class	Clostridia	Clostridia	Bacilli	Clostridia	Clostridia	Clostridia	Clostridia	Clostridia	Clostridia
Order	Clostridiales	Clostridiales	Bacillales	Clostridiales	Clostridiales	Clostridiales	Clostridiales	Clostridiales	Clostridiales
Family	Lachnospiraceae	Lachnospiraceae	Paenibacillaceae_	Clostridiaceae_	Lachnospiraceae	Lachnospiraceae	Lachnospiraceae	Lachnospiraceae	Eubacteriaceae
Genus	Clostridium_XlVa	Clostridium_XlVa	Brevibacillus	Clostridium_sensu_stricto	Clostridium_XlVa	Clostridium_XlVa	Acetatifactor	Clostridium_XlVa	Eubacterium
#NAME	ZOTU_0123	ZOTU_0124	ZOTU_0125	ZOTU_0126	ZOTU_0127	ZOTU_0128	ZOTU_0129	ZOTU_0130	ZOTU_0131
CD_49	0	2	0	0	2	0	0	0	0
CD_51	0	7	0	18	1	0	1	0	0
CD_52	0	2	0	0	0	0	0	0	0
CD_65	0	3	0	11	1	0	3	0	4
CD_66	0	0	1	2	0	0	0	0	0
CD_67	0	0	1271	0	0	0	0	0	0
CD_68	0	0	7	1	0	0	0	0	0
CD_78	0	0	0	0	0	0	0	0	0
CD_79	0	0	0	0	0	0	0	0	0
CDFG_53	6	3	0	9	1	0	0	2	0
CDFG_56	0	0	0	0	0	0	1	0	0
CDFG_69	0	0	3	7	0	0	0	3	0
CDFG_70	11	6	0	1	3	0	31	15	2
CDFG_71	0	2	0	0	1	0	2	0	0
HF_41	0	1	1	0	4	0	0	1	1
HF_42	0	0	2	4	0	0	0	0	0
HF_43	1	1	1	0	11	0	0	3	0
HF_57	0	1	6	0	0	0	0	0	1
HF_58	0	0	50	12	2	0	0	0	0
HF_72	12	0	0	1	3	0	9	11	0
HF_73	0	0	2	3	1	0	0	0	0
HF_74	2	0	2	0	0	0	1	9	0
HFFG_45	26	7	0	38	5	0	4	34	0
HFFG_48	0	1	0	1	2	0	23	0	0
HFFG_62	0	0	0	2	1	0	80	16	0
HFFG_77	3	0	5	0	0	0	2	2	0

Kingdom	Bacteria	Bacteria	Bacteria	Bacteria	Bacteria	Bacteria	Bacteria	Bacteria
Phylum	Firmicutes	Firmicutes	Firmicutes	Firmicutes	Firmicutes	Firmicutes	Firmicutes	Firmicutes
Class	Clostridia	Clostridia	Erysipelotrichia	Clostridia	Clostridia	Erysipelotrichia	Clostridia	Clostridia
Order	Clostridiales	Clostridiales	Erysipelotrichales	Clostridiales	Clostridiales	Erysipelotrichales	Clostridiales	Clostridiales
Family	Ruminococcaceae	Lachnospiraceae	Erysipelotrichaceae	Lachnospiraceae	Lachnospiraceae	Erysipelotrichaceae	Lachnospiraceae	Ruminococcaceae
Genus	Clostridium_IV	Acetatifactor	Coprobacillus	Clostridium_XlVa	Acetatifactor	Erysipelotrichaceae_incertae_sedis	Dorea	Clostridium_IV
#NAME	ZOTU_0132	ZOTU_0133	ZOTU_0134	ZOTU_0135	ZOTU_0136	ZOTU_0137	ZOTU_0138	ZOTU_0139
CD_49	0	0	0	0	1	0	0	0
CD_51	0	0	0	0	0	2	0	0
CD_52	0	0	0	0	0	0	0	1
CD_65	1	0	0	0	4	0	0	2
CD_66	0	1	0	0	11	0	1	0
CD_67	0	0	0	0	0	0	0	0
CD_68	0	0	0	0	0	0	0	0
CD_78	0	0	0	0	0	0	0	0
CD_79	0	0	0	0	0	0	0	0
CDFG_53	1	1	0	0	0	1	15	0
CDFG_56	0	0	0	0	0	0	1	1
CDFG_69	0	0	1	0	1	0	11	0
CDFG_70	3	22	2	3	11	1	71	66
CDFG_71	0	0	0	0	1	0	5	0
HF_41	0	0	0	0	0	0	0	0
HF_42	0	0	0	0	3	0	0	0
HF_43	3	0	0	3	20	0	0	4
HF_57	0	0	0	0	2	0	0	0
HF_58	0	0	0	0	9	0	0	1
HF_72	0	0	0	0	17	0	1	1
HF_73	0	0	0	0	6	0	0	1
HF_74	0	0	0	0	1	0	0	0
HFFG_45	0	0	0	0	0	5	19	0
HFFG_48	0	0	0	0	0	0	2	1
HFFG_62	3	0	0	2	0	2	28	5
HFFG_77	0	0	0	0	1	0	27	5

Kingdom	Bacteria	Bacteria	Bacteria	Bacteria	Bacteria	Bacteria	Bacteria	Bacteria
Phylum	Firmicutes	Firmicutes	Firmicutes	Firmicutes	Firmicutes	Firmicutes	Firmicutes	Firmicutes
Class	Erysipelotrichia	Clostridia	Clostridia	Clostridia	Clostridia	Clostridia	Clostridia	Clostridia
Order	Erysipelotrichales	Clostridiales	Clostridiales	Clostridiales	Clostridiales	Clostridiales	Clostridiales	Clostridiales
Family	Erysipelotrichaceae	Lachnospiraceae	Clostridiaceae_	Lachnospiraceae	Ruminococcaceae	Clostridiaceae_	Ruminococcaceae	Ruminococcaceae
Genus	Erysipelotrichaceae_incertae_sedis	Eisenbergiella	Alkaliphilus	Clostridium_XlVa	Intestinimonas	Alkaliphilus	Ruminococcus	Clostridium_IV
#NAME	ZOTU_0140	ZOTU_0141	ZOTU_0142	ZOTU_0143	ZOTU_0144	ZOTU_0145	ZOTU_0146	ZOTU_0147
CD_49	0	1	0	2	0	0	0	0
CD_51	0	0	0	7	0	0	0	0
CD_52	0	0	0	0	0	0	0	0
CD_65	0	0	0	7	1	0	0	0
CD_66	0	1	0	5	0	0	0	0
CD_67	0	0	0	0	0	0	0	0
CD_68	0	0	0	1	0	0	0	0
CD_78	0	0	0	0	0	0	0	0
CD_79	0	0	0	0	0	0	0	0
CDFG_53	0	0	0	5	0	21	0	0
CDFG_56	0	0	0	1	0	1	1	0
CDFG_69	0	0	0	1	0	0	0	0
CDFG_70	0	6	0	35	9	8	5	81
CDFG_71	0	0	0	1	0	0	0	10
HF_41	0	0	0	1	0	0	1	1
HF_42	0	0	0	0	0	0	0	1
HF_43	0	0	0	14	3	0	2	0
HF_57	0	0	0	0	0	0	0	0
HF_58	0	0	0	2	0	0	0	0
HF_72	0	0	0	3	0	0	0	1
HF_73	0	1	0	3	0	1	0	0
HF_74	0	0	0	5	0	0	0	0
HFFG_45	0	3	0	36	1	0	0	0
HFFG_48	1	0	0	2	0	4	0	0
HFFG_62	1	1	0	4	0	1	0	0
HFFG_77	0	0	0	5	0	0	0	1

Kingdom	Bacteria	Bacteria	Bacteria	Bacteria	Bacteria	Bacteria	Bacteria	Bacteria	Bacteria
Phylum	Firmicutes	Firmicutes	Firmicutes	Firmicutes	Firmicutes	Firmicutes	Firmicutes	Actinobacteria	Firmicutes
Class	Clostridia	Clostridia	Clostridia	Clostridia	Clostridia	Clostridia	Clostridia	Actinobacteria	Clostridia
Order	Clostridiales	Clostridiales	Clostridiales	Clostridiales	Clostridiales	Clostridiales	Clostridiales	Coriobacteriales	Clostridiales
Family	Lachnospiraceae	Lachnospiraceae	Clostridiaceae_	Clostridiaceae_	Eubacteriaceae	Lachnospiraceae	Ruminococcaceae	Coriobacteriaceae	Proteinivoraceae
Genus	Anaerosporobacter	Butyrivibrio	Alkaliphilus	Alkaliphilus	Eubacterium	Clostridium_XlVa	Flavonifractor	Adlercreutzia	Proteinivorax
#NAME	ZOTU_0148	ZOTU_0149	ZOTU_0150	ZOTU_0151	ZOTU_0152	ZOTU_0153	ZOTU_0154	ZOTU_0155	ZOTU_0156
CD_49	0	3	0	0	0	0	0	2	0
CD_51	0	0	0	0	0	0	0	1	0
CD_52	0	4	0	0	0	0	0	10	0
CD_65	0	0	0	0	0	0	0	0	0
CD_66	0	2	0	0	0	0	0	0	0
CD_67	0	1	0	0	0	0	0	9	0
CD_68	1	0	0	0	0	1	2	1	0
CD_78	0	0	0	0	0	0	0	0	0
CD_79	0	0	0	0	0	0	0	0	0
CDFG_53	0	1	0	0	0	0	0	1	0
CDFG_56	0	0	0	0	0	0	0	7	0
CDFG_69	0	0	0	0	0	0	0	0	0
CDFG_70	10	4	0	1	0	2	1	0	1
CDFG_71	1	0	0	1	0	2	0	0	0
HF_41	0	0	0	0	0	0	0	0	0
HF_42	1	0	0	0	0	0	0	0	0
HF_43	7	0	0	0	5	9	5	0	1
HF_57	0	0	0	0	0	0	0	0	0
HF_58	1	0	0	0	0	0	0	0	0
HF_72	0	1	0	0	0	0	0	0	0
HF_73	0	0	0	0	0	0	0	1	0
HF_74	0	0	0	0	0	1	0	2	0
HFFG_45	0	43	0	0	0	0	0	0	0
HFFG_48	0	12	0	0	0	0	0	4	0
HFFG_62	0	74	0	0	0	1	0	22	0
HFFG_77	0	2	0	0	0	1	0	1	0

Kingdom	Bacteria	Bacteria	Bacteria	Bacteria	Bacteria	Bacteria	Bacteria
Phylum	Firmicutes	Bacteroidetes	Firmicutes	Firmicutes	Firmicutes	Firmicutes	Firmicutes
Class	Clostridia	Bacteroidia	Clostridia	Clostridia	Clostridia	Clostridia	Clostridia
Order	Clostridiales	Bacteroidales	Clostridiales	Clostridiales	Clostridiales	Clostridiales	Clostridiales
Family	Lachnospiraceae	Porphyromonadaceae	Lachnospiraceae	Lachnospiraceae	Lachnospiraceae	Lachnospiraceae	Lachnospiraceae
Genus	Lachnospiracea_incertae_sedis	Barnesiella	Clostridium_XlVa	Clostridium_XlVa	Clostridium_XlVa	Butyrivibrio	Clostridium_XlVa
#NAME	ZOTU_0157	ZOTU_0158	ZOTU_0159	ZOTU_0160	ZOTU_0161	ZOTU_0162	ZOTU_0163
CD_49	0	0	0	0	0	6	0
CD_51	0	0	0	0	0	0	0
CD_52	1	0	0	0	1	3	0
CD_65	1	0	0	0	6	2	0
CD_66	0	0	0	0	4	0	0
CD_67	0	0	0	0	0	3	0
CD_68	0	0	0	0	4	0	0
CD_78	0	0	0	0	0	0	0
CD_79	0	0	0	0	0	0	0
CDFG_53	0	0	0	1	1	1	0
CDFG_56	0	0	0	0	0	0	0
CDFG_69	0	0	0	0	1	0	0
CDFG_70	6	0	1	20	29	2	20
CDFG_71	0	1	0	0	2	0	0
HF_41	0	0	0	1	0	0	0
HF_42	0	0	0	2	0	0	0
HF_43	0	0	0	2	0	0	0
HF_57	0	0	0	0	0	0	0
HF_58	0	0	0	0	12	0	0
HF_72	0	0	0	0	15	0	0
HF_73	0	0	0	0	4	1	0
HF_74	1	0	0	0	3	0	0
HFFG_45	0	0	0	72	0	23	15
HFFG_48	0	0	0	5	3	4	0
HFFG_62	0	0	3	0	0	52	3
HFFG_77	0	0	0	5	0	1	0

Kingdom	Bacteria	Bacteria	Bacteria	Bacteria	Bacteria	Bacteria	Bacteria	Bacteria
Phylum	Firmicutes	Firmicutes	Firmicutes	Firmicutes	Firmicutes	Firmicutes	Firmicutes	Tenericutes
Class	Clostridia	Clostridia	Clostridia	Clostridia	Clostridia	Clostridia	Clostridia	Mollicutes
Order	Clostridiales	Clostridiales	Clostridiales	Clostridiales	Clostridiales	Clostridiales	Clostridiales	Anaeroplasmatales
Family	Lachnospiraceae	Clostridiaceae_	Lachnospiraceae	Lachnospiraceae	Ruminococcaceae	Lachnospiraceae	Lachnospiraceae	Anaeroplasmataceae
Genus	Lachnospiracea_incertae_sedis	Geosporobacter	Clostridium_XlVa	Clostridium_XlVa	Anaerotruncus	Clostridium_XlVa	Clostridium_XlVa	Anaeroplasma
#NAME	ZOTU_0164	ZOTU_0165	ZOTU_0166	ZOTU_0167	ZOTU_0168	ZOTU_0169	ZOTU_0170	ZOTU_0171
CD_49	0	0	0	0	0	0	3	0
CD_51	0	0	0	0	0	0	1	0
CD_52	0	0	1	0	0	1	0	0
CD_65	0	0	0	0	0	0	0	0
CD_66	0	0	0	0	0	0	0	1
CD_67	0	0	1	0	0	0	0	0
CD_68	0	1	1	0	0	0	0	1
CD_78	0	0	0	0	0	0	0	0
CD_79	0	0	0	0	0	0	0	0
CDFG_53	0	0	0	0	0	0	0	0
CDFG_56	0	1	0	0	0	0	0	0
CDFG_69	0	0	0	0	0	1	0	0
CDFG_70	0	0	5	0	0	20	0	0
CDFG_71	0	0	0	0	0	0	0	0
HF_41	0	0	0	0	0	0	1	0
HF_42	0	0	1	0	0	0	0	0
HF_43	0	0	6	0	1	0	1	0
HF_57	0	0	0	0	0	0	0	0
HF_58	0	0	0	0	0	0	0	0
HF_72	0	0	0	0	0	1	0	0
HF_73	0	0	0	0	0	0	0	0
HF_74	0	0	0	0	0	0	0	0
HFFG_45	0	0	0	0	0	0	0	0
HFFG_48	0	0	0	0	0	0	0	0
HFFG_62	0	0	0	5	0	0	2	0
HFFG_77	0	0	0	0	0	0	0	0

Kingdom	Bacteria	Bacteria	Bacteria	Bacteria	Bacteria	Bacteria	Bacteria	Bacteria	Bacteria
Phylum	Firmicutes	Firmicutes	Firmicutes	Firmicutes	Firmicutes	Firmicutes	Firmicutes	Firmicutes	Tenericutes
Class	Clostridia	Clostridia	Clostridia	Clostridia	Clostridia	Clostridia	Clostridia	Clostridia	Mollicutes
Order	Clostridiales	Clostridiales	Clostridiales	Clostridiales	Clostridiales	Clostridiales	Clostridiales	Clostridiales	Entomoplasmatales
Family	Clostridiaceae_	Ruminococcaceae	Lachnospiraceae	Lachnospiraceae	Ruminococcaceae	Lachnospiraceae	Lachnospiraceae	Ruminococcaceae	Spiroplasmataceae
Genus	Alkaliphilus	Oscillibacter	Acetatifactor	Blautia	Clostridium_IV	Dorea	Clostridium_XlVa	Oscillibacter	Spiroplasma
#NAME	ZOTU_0172	ZOTU_0173	ZOTU_0174	ZOTU_0175	ZOTU_0176	ZOTU_0177	ZOTU_0178	ZOTU_0179	ZOTU_0180
CD_49	2	0	0	0	0	0	0	0	0
CD_51	0	0	1	0	0	0	0	0	0
CD_52	0	0	0	0	0	0	0	0	0
CD_65	0	0	0	0	0	0	2	0	0
CD_66	0	0	2	0	0	0	6	0	1
CD_67	0	0	0	0	0	0	0	0	1
CD_68	0	0	2	0	0	0	0	0	0
CD_78	0	0	0	0	0	0	0	0	0
CD_79	0	0	0	0	0	0	0	0	0
CDFG_53	0	0	3	0	0	10	0	0	0
CDFG_56	0	0	1	0	0	0	0	0	0
CDFG_69	0	0	0	0	0	3	0	0	0
CDFG_70	0	0	60	0	2	39	17	0	4
CDFG_71	0	0	3	0	0	4	0	0	0
HF_41	0	0	0	0	0	0	0	0	0
HF_42	0	0	1	0	0	0	0	0	0
HF_43	62	0	6	0	0	1	0	0	2
HF_57	0	0	0	0	0	0	0	0	0
HF_58	0	0	1	0	0	0	0	0	0
HF_72	0	0	0	0	0	0	0	0	0
HF_73	0	0	1	0	0	0	2	0	0
HF_74	0	0	0	0	0	0	0	0	0
HFFG_45	0	0	7	0	0	3	0	0	0
HFFG_48	0	0	0	0	0	1	0	0	0
HFFG_62	0	0	3	0	0	16	1	0	0
HFFG_77	0	0	0	0	0	15	0	0	0

Kingdom	Bacteria	Bacteria	Bacteria	Bacteria	Bacteria	Bacteria	Bacteria	Bacteria	Bacteria
Phylum	Firmicutes	Firmicutes	Firmicutes	Firmicutes	Firmicutes	Firmicutes	Firmicutes	Firmicutes	Firmicutes
Class	Clostridia	Clostridia	Clostridia	Clostridia	Clostridia	Clostridia	Clostridia	Clostridia	Clostridia
Order	Clostridiales	Clostridiales	Clostridiales	Clostridiales	Clostridiales	Clostridiales	Clostridiales	Halanaerobiales	Clostridiales
Family	Lachnospiraceae	Lachnospiraceae	Ruminococcaceae	Lachnospiraceae	Lachnospiraceae	Lachnospiraceae	Lachnospiraceae	Halanaerobiaceae	Ruminococcaceae
Genus	Acetatifactor	Clostridium_XlVa	Butyricicoccus	Clostridium_XlVa	Clostridium_XlVa	Lachnospira	Clostridium_XlVa	Halanaerobium	Clostridium_IV
#NAME	ZOTU_0181	ZOTU_0182	ZOTU_0183	ZOTU_0184	ZOTU_0185	ZOTU_0186	ZOTU_0187	ZOTU_0188	ZOTU_0189
CD_49	0	0	0	1	1	0	0	0	0
CD_51	0	0	0	0	0	0	2	0	0
CD_52	0	0	0	0	2	0	0	0	0
CD_65	2	0	0	3	0	0	0	0	1
CD_66	3	0	0	3	6	0	0	0	0
CD_67	0	0	0	0	0	0	0	0	0
CD_68	0	0	0	1	0	0	0	0	0
CD_78	0	0	0	0	0	0	0	0	0
CD_79	0	0	0	0	0	0	0	0	0
CDFG_53	0	0	0	0	0	0	0	0	0
CDFG_56	0	0	0	1	0	0	0	0	0
CDFG_69	0	0	0	4	0	1	0	0	0
CDFG_70	4	0	5	41	41	0	4	20	26
CDFG_71	0	0	0	1	0	0	0	0	0
HF_41	0	0	0	0	0	0	0	0	0
HF_42	0	0	0	0	0	0	0	0	0
HF_43	0	0	0	0	0	0	0	0	1
HF_57	0	0	0	0	0	0	0	0	0
HF_58	0	0	0	6	3	0	0	1	0
HF_72	0	0	0	14	4	0	0	5	2
HF_73	0	0	0	6	4	0	0	0	0
HF_74	0	0	0	2	3	0	0	0	0
HFFG_45	0	0	0	0	0	0	8	0	0
HFFG_48	0	0	0	2	0	0	0	0	0
HFFG_62	0	0	1	0	0	0	0	0	1
HFFG_77	0	0	2	1	0	0	2	8	0

Kingdom	Bacteria	Bacteria	Bacteria	Bacteria	Bacteria	Bacteria	Bacteria	Bacteria	Bacteria
Phylum	Actinobacteria	Firmicutes	Firmicutes	Firmicutes	Firmicutes	Firmicutes	Firmicutes	Firmicutes	Firmicutes
Class	Actinobacteria	Clostridia	Clostridia	Clostridia	Clostridia	Clostridia	Clostridia	Clostridia	Clostridia
Order	Actinomycetales	Clostridiales	Clostridiales	Clostridiales	Clostridiales	Clostridiales	Clostridiales	Clostridiales	Clostridiales
Family	Streptomycetaceae	Lachnospiraceae	Lachnospiraceae	Lachnospiraceae	Ruminococcaceae	Ruminococcaceae	Ruminococcaceae	Lachnospiraceae	Lachnospiraceae
Genus	Streptomyces	Ruminococcus	Roseburia	Clostridium_XlVa	Clostridium_III	Oscillibacter	Anaerotruncus	Clostridium_XlVa	Clostridium_XlVa
#NAME	ZOTU_0190	ZOTU_0191	ZOTU_0192	ZOTU_0193	ZOTU_0194	ZOTU_0195	ZOTU_0196	ZOTU_0197	ZOTU_0198
CD_49	5	2	0	0	0	0	0	0	0
CD_51	0	0	0	0	0	0	0	0	0
CD_52	0	1	0	0	0	0	0	0	0
CD_65	0	32	3	1	0	0	0	0	0
CD_66	0	2	1	2	0	0	0	0	0
CD_67	0	0	0	0	0	0	0	0	0
CD_68	0	0	0	0	0	0	0	0	0
CD_78	0	0	0	0	0	0	0	0	0
CD_79	0	0	0	0	0	0	0	0	0
CDFG_53	0	0	0	0	2	0	0	0	0
CDFG_56	0	0	0	0	2	0	0	0	0
CDFG_69	13	1	2	0	0	0	0	0	2
CDFG_70	36	5	0	35	16	0	0	0	5
CDFG_71	2	2	0	2	0	0	0	0	0
HF_41	0	1	0	1	1	0	0	0	0
HF_42	0	0	0	0	0	0	0	0	0
HF_43	1	35	3	0	7	0	1	0	0
HF_57	2	1	0	0	0	0	0	0	0
HF_58	3	0	0	5	0	0	0	0	0
HF_72	9	13	0	9	0	0	0	0	0
HF_73	12	8	0	1	1	0	0	0	0
HF_74	7	7	0	2	0	0	0	0	0
HFFG_45	0	0	2	0	0	0	0	0	0
HFFG_48	0	1	0	1	0	0	0	0	0
HFFG_62	42	2	0	0	1	0	0	0	1
HFFG_77	2	1	0	0	4	0	0	0	0

Kingdom	Bacteria	Bacteria	Bacteria	Bacteria	Bacteria	Bacteria	Bacteria	Bacteria	Bacteria
Phylum	Firmicutes	Firmicutes	Firmicutes	Firmicutes	Firmicutes	Firmicutes	Firmicutes	Firmicutes	Firmicutes
Class	Clostridia	Clostridia	Clostridia	Clostridia	Clostridia	Clostridia	Clostridia	Clostridia	Clostridia
Order	Clostridiales	Clostridiales	Clostridiales	Clostridiales	Clostridiales	Clostridiales	Clostridiales	Clostridiales	Clostridiales
Family	Ruminococcaceae	Lachnospiraceae	Lachnospiraceae	Lachnospiraceae	Lachnospiraceae	Ruminococcaceae	Lachnospiraceae	Lachnospiraceae	Ruminococcaceae
Genus	Intestinimonas	Clostridium_XlVa	Acetatifactor	Acetatifactor	Clostridium_XlVa	Anaerotruncus	Clostridium_XlVa	Clostridium_XlVa	Hydrogenoanaerobacterium
#NAME	ZOTU_0199	ZOTU_0200	ZOTU_0201	ZOTU_0202	ZOTU_0203	ZOTU_0204	ZOTU_0205	ZOTU_0206	ZOTU_0207
CD_49	0	0	0	0	1	0	0	0	0
CD_51	0	0	0	0	9	0	0	0	0
CD_52	0	0	0	0	0	0	0	1	0
CD_65	1	0	0	0	0	0	0	1	0
CD_66	0	0	0	0	0	0	0	0	0
CD_67	0	0	0	0	0	0	0	0	0
CD_68	0	0	0	0	0	0	0	0	0
CD_78	0	0	0	0	0	0	0	0	0
CD_79	0	0	0	0	0	0	0	0	0
CDFG_53	0	0	0	0	0	1	0	0	0
CDFG_56	0	1	0	0	0	0	0	0	0
CDFG_69	0	0	0	0	0	0	0	0	0
CDFG_70	14	31	7	11	0	2	0	13	0
CDFG_71	0	0	3	0	0	0	0	2	0
HF_41	0	0	0	0	4	0	0	0	0
HF_42	0	0	0	0	0	0	0	0	0
HF_43	0	0	2	0	1	0	0	1	0
HF_57	0	0	0	0	0	0	0	0	0
HF_58	0	0	0	0	0	0	0	0	0
HF_72	0	0	0	0	0	0	0	0	0
HF_73	0	0	1	2	0	0	0	0	0
HF_74	0	0	1	0	0	0	0	0	0
HFFG_45	1	0	0	0	1	0	0	0	0
HFFG_48	0	0	0	0	0	0	0	0	0
HFFG_62	2	1	0	0	0	2	0	0	0
HFFG_77	0	0	0	0	0	0	0	0	0

Kingdom	Bacteria	Bacteria	Bacteria	Bacteria	Bacteria	Bacteria	Bacteria	Bacteria	Bacteria
Phylum	Firmicutes	Firmicutes	Firmicutes	Firmicutes	Firmicutes	Firmicutes	Firmicutes	Firmicutes	Firmicutes
Class	Clostridia	Clostridia	Clostridia	Clostridia	Clostridia	Clostridia	Clostridia	Clostridia	Clostridia
Order	Clostridiales	Clostridiales	Clostridiales	Clostridiales	Clostridiales	Clostridiales	Clostridiales	Clostridiales	Clostridiales
Family	Lachnospiraceae	Clostridiaceae_	Clostridiaceae_	Lachnospiraceae	Clostridiaceae_	Lachnospiraceae	Peptostreptococcaceae	Ruminococcaceae	Ruminococcaceae
Genus	Clostridium_XlVa	Alkaliphilus	Alkaliphilus	Clostridium_XlVa	Alkaliphilus	Acetatifactor	Filifactor	Intestinimonas	Ruminococcus
#NAME	ZOTU_0208	ZOTU_0209	ZOTU_0210	ZOTU_0211	ZOTU_0212	ZOTU_0213	ZOTU_0214	ZOTU_0215	ZOTU_0216
CD_49	0	0	0	0	0	0	0	0	0
CD_51	0	0	0	0	0	1	0	0	2
CD_52	2	0	0	0	0	0	0	0	0
CD_65	1	0	0	0	0	0	0	0	2
CD_66	1	0	0	0	0	0	0	0	0
CD_67	0	0	0	0	1	0	0	0	0
CD_68	0	0	0	0	0	0	0	0	0
CD_78	0	0	0	0	0	0	0	0	0
CD_79	0	0	0	0	0	0	0	0	0
CDFG_53	0	0	0	0	0	0	0	0	10
CDFG_56	0	0	0	0	0	0	0	0	0
CDFG_69	1	0	0	0	0	0	0	0	1
CDFG_70	1	0	0	0	0	9	0	0	12
CDFG_71	0	0	0	0	0	0	0	0	2
HF_41	0	0	0	0	0	0	0	0	0
HF_42	0	0	0	0	0	0	0	0	0
HF_43	0	0	0	0	0	1	0	0	0
HF_57	0	0	0	0	0	0	0	0	0
HF_58	0	0	0	0	0	0	0	0	0
HF_72	0	0	0	0	0	0	0	0	0
HF_73	0	0	0	0	0	0	0	0	0
HF_74	0	0	0	0	0	0	0	0	0
HFFG_45	0	0	0	0	0	0	0	0	4
HFFG_48	0	0	0	0	0	0	0	0	0
HFFG_62	0	0	0	0	0	0	0	0	0
HFFG_77	0	0	0	0	0	0	0	0	1

Kingdom	Bacteria	Bacteria	Bacteria	Bacteria	Bacteria	Bacteria	Bacteria	Bacteria
Phylum	Firmicutes	Firmicutes	Firmicutes	Bacteroidetes	Firmicutes	Actinobacteria	Firmicutes	Firmicutes
Class	Clostridia	Clostridia	Clostridia	Bacteroidia	Clostridia	Actinobacteria	Clostridia	Clostridia
Order	Clostridiales	Clostridiales	Clostridiales	Bacteroidales	Clostridiales	Coriobacteriales	Clostridiales	Clostridiales
Family	Lachnospiraceae	Ruminococcaceae	Lachnospiraceae	Porphyromonadaceae	Ruminococcaceae	Coriobacteriaceae	Ruminococcaceae	Ruminococcaceae
Genus	Lachnospiracea_incertae_sedis	Sporobacter	Acetatifactor	Barnesiella	Sporobacter	Eggerthella	Anaerotruncus	Clostridium_IV
#NAME	ZOTU_0217	ZOTU_0218	ZOTU_0219	ZOTU_0220	ZOTU_0221	ZOTU_0222	ZOTU_0223	ZOTU_0224
CD_49	0	0	0	0	0	0	0	0
CD_51	0	0	1	0	0	7	0	0
CD_52	0	0	0	0	0	1	0	0
CD_65	10	0	3	0	0	0	0	0
CD_66	3	0	1	0	0	1	0	0
CD_67	0	0	0	0	0	0	0	0
CD_68	1	0	0	0	0	2	0	0
CD_78	0	0	0	0	0	0	0	0
CD_79	0	0	0	0	0	0	0	0
CDFG_53	0	0	0	0	0	0	0	0
CDFG_56	0	0	0	0	0	0	0	0
CDFG_69	3	0	1	0	0	32	0	0
CDFG_70	21	0	34	0	0	3	0	1
CDFG_71	3	0	1	0	0	16	0	0
HF_41	0	0	0	0	0	0	0	0
HF_42	0	0	1	0	0	0	0	0
HF_43	0	0	5	0	0	0	0	0
HF_57	0	0	0	0	0	0	0	0
HF_58	0	0	1	0	0	0	0	0
HF_72	0	0	0	0	0	3	0	0
HF_73	2	0	0	0	0	0	0	0
HF_74	0	0	0	0	0	0	0	0
HFFG_45	0	0	6	0	0	0	0	0
HFFG_48	0	0	0	0	0	0	0	0
HFFG_62	2	0	4	0	0	8	0	0
HFFG_77	0	0	1	0	0	5	0	0

Kingdom	Bacteria	Bacteria	Bacteria	Bacteria	Bacteria	Bacteria	Bacteria	Bacteria	Bacteria
Phylum	Firmicutes	Firmicutes	Firmicutes	Firmicutes	Firmicutes	Bacteroidetes	Firmicutes	Firmicutes	Firmicutes
Class	Clostridia	Clostridia	Clostridia	Clostridia	Clostridia	Bacteroidia	Clostridia	Clostridia	Clostridia
Order	Clostridiales	Clostridiales	Clostridiales	Clostridiales	Clostridiales	Bacteroidales	Clostridiales	Clostridiales	Clostridiales
Family	Lachnospiraceae	Ruminococcaceae	Lachnospiraceae	Clostridiaceae_	Lachnospiraceae	Rikenellaceae	Ruminococcaceae	Lachnospiraceae	Lachnospiraceae
Genus	Clostridium_XlVa	Hydrogenoanaerobacterium	Acetatifactor	Alkaliphilus	Clostridium_XlVa	Alistipes	Acetanaerobacterium	Clostridium_XlVb	Clostridium_XlVa
#NAME	ZOTU_0225	ZOTU_0226	ZOTU_0227	ZOTU_0228	ZOTU_0229	ZOTU_0230	ZOTU_0231	ZOTU_0232	ZOTU_0233
CD_49	0	0	0	0	0	0	0	0	0
CD_51	0	1	0	0	0	0	0	0	0
CD_52	0	0	0	0	0	0	0	1	0
CD_65	0	0	0	0	0	0	0	2	6
CD_66	0	0	0	0	0	0	0	0	0
CD_67	0	0	0	0	0	0	0	0	0
CD_68	0	0	0	0	0	0	0	0	0
CD_78	0	0	0	0	0	0	0	0	0
CD_79	0	0	0	0	0	0	0	0	0
CDFG_53	0	0	0	0	0	0	0	1	0
CDFG_56	0	0	0	0	0	0	0	0	0
CDFG_69	0	0	0	0	0	0	0	1	0
CDFG_70	0	0	0	0	0	0	0	15	7
CDFG_71	0	0	0	0	0	0	0	1	0
HF_41	0	0	0	0	0	0	0	0	0
HF_42	0	0	0	0	0	0	0	0	0
HF_43	0	0	0	0	0	0	0	0	11
HF_57	0	0	0	0	0	0	0	0	0
HF_58	0	0	0	0	0	0	0	3	2
HF_72	0	0	0	0	0	0	0	9	4
HF_73	0	0	0	0	0	0	0	2	5
HF_74	0	0	0	0	0	0	0	0	0
HFFG_45	0	0	0	0	0	0	0	3	0
HFFG_48	0	0	0	0	0	0	0	0	2
HFFG_62	0	0	0	0	0	0	0	28	1
HFFG_77	0	0	0	0	0	0	0	0	0

Kingdom	Bacteria	Bacteria	Bacteria	Bacteria	Bacteria	Bacteria	Bacteria	Bacteria	Bacteria
Phylum	Firmicutes	Actinobacteria	Firmicutes	Firmicutes	Firmicutes	Firmicutes	Firmicutes	Firmicutes	Firmicutes
Class	Clostridia	Actinobacteria	Clostridia	Clostridia	Clostridia	Clostridia	Clostridia	Clostridia	Clostridia
Order	Clostridiales	Actinomycetales	Clostridiales	Clostridiales	Clostridiales	Clostridiales	Clostridiales	Clostridiales	Clostridiales
Family	Clostridiaceae_	Streptomycetaceae	Lachnospiraceae	Lachnospiraceae	Lachnospiraceae	Lachnospiraceae	Lachnospiraceae	Clostridiaceae_	Lachnospiraceae
Genus	Alkaliphilus	Streptomyces	Clostridium_XlVa	Lachnospiracea_incertae_sedis	Clostridium_XlVa	Butyrivibrio	Clostridium_XlVa	Alkaliphilus	Clostridium_XlVa
#NAME	ZOTU_0234	ZOTU_0235	ZOTU_0236	ZOTU_0237	ZOTU_0238	ZOTU_0239	ZOTU_0240	ZOTU_0241	ZOTU_0242
CD_49	0	0	0	0	0	0	0	0	0
CD_51	0	0	0	0	0	0	0	0	0
CD_52	0	0	0	0	0	0	0	0	0
CD_65	0	0	0	0	2	1	0	0	0
CD_66	0	0	0	0	0	0	0	0	0
CD_67	0	0	0	0	0	0	0	0	0
CD_68	0	0	0	0	0	0	0	0	0
CD_78	0	0	0	0	0	1	0	0	0
CD_79	0	0	0	0	0	0	0	0	0
CDFG_53	0	0	0	0	0	0	0	0	0
CDFG_56	0	0	0	0	0	0	0	0	0
CDFG_69	0	19	0	0	0	2	0	0	0
CDFG_70	0	40	0	0	6	8	54	0	5
CDFG_71	0	0	0	0	0	0	0	0	0
HF_41	0	0	0	0	0	0	0	0	0
HF_42	0	0	0	0	0	0	0	0	0
HF_43	0	0	0	0	10	0	0	0	0
HF_57	0	0	0	0	1	0	0	0	0
HF_58	0	0	0	0	0	0	0	0	0
HF_72	0	4	0	0	2	0	0	0	1
HF_73	0	7	0	0	1	0	0	0	0
HF_74	0	15	0	0	1	0	0	0	0
HFFG_45	0	0	0	0	0	0	0	0	0
HFFG_48	0	0	0	0	0	0	0	0	0
HFFG_62	0	13	0	0	1	0	0	0	0
HFFG_77	0	0	0	0	0	0	0	0	0

Kingdom	Bacteria	Bacteria	Bacteria	Bacteria	Bacteria	Bacteria	Bacteria
Phylum	Actinobacteria	Firmicutes	Firmicutes	Firmicutes	Firmicutes	Firmicutes	Firmicutes
Class	Actinobacteria	Clostridia	Clostridia	Clostridia	Erysipelotrichia	Clostridia	Clostridia
Order	Coriobacteriales	Clostridiales	Clostridiales	Clostridiales	Erysipelotrichales	Clostridiales	Clostridiales
Family	Coriobacteriaceae	Lachnospiraceae	Lachnospiraceae	Ruminococcaceae	Erysipelotrichaceae	Lachnospiraceae	Lachnospiraceae
Genus	Enterorhabdus	Lachnospiracea_incertae_sedis	Lachnospiracea_incertae_sedis	Clostridium_IV	Catenisphaera	Clostridium_XlVa	Roseburia
#NAME	ZOTU_0243	ZOTU_0244	ZOTU_0245	ZOTU_0246	ZOTU_0247	ZOTU_0248	ZOTU_0249
CD_49	0	0	0	0	0	0	0
CD_51	0	0	0	0	0	0	0
CD_52	0	0	0	0	0	0	0
CD_65	0	1	0	0	0	0	0
CD_66	0	0	0	0	0	0	0
CD_67	7	0	0	0	0	0	0
CD_68	0	0	0	0	0	0	0
CD_78	0	0	0	0	0	0	0
CD_79	0	0	0	0	0	0	0
CDFG_53	0	1	0	0	0	0	0
CDFG_56	0	0	0	0	0	0	0
CDFG_69	0	2	0	0	0	2	0
CDFG_70	0	26	1	0	0	26	0
CDFG_71	0	3	0	0	0	0	0
HF_41	0	0	0	0	0	0	0
HF_42	0	0	0	0	0	0	0
HF_43	0	1	1	0	0	0	0
HF_57	0	0	0	0	0	0	0
HF_58	0	0	0	0	0	0	0
HF_72	0	0	0	0	0	0	0
HF_73	0	0	0	0	0	0	0
HF_74	0	0	0	0	0	0	0
HFFG_45	1	1	0	0	0	1	0
HFFG_48	0	0	0	0	1	0	0
HFFG_62	0	0	0	0	0	0	0
HFFG_77	0	0	0	0	0	0	0

Kingdom	Bacteria	Bacteria	Bacteria	Bacteria	Bacteria	Bacteria	Bacteria	Bacteria	Bacteria
Phylum	Firmicutes	Firmicutes	Firmicutes	Firmicutes	Firmicutes	Firmicutes	Firmicutes	Firmicutes	Firmicutes
Class	Clostridia	Clostridia	Clostridia	Bacilli	Clostridia	Clostridia	Clostridia	Clostridia	Clostridia
Order	Clostridiales	Clostridiales	Clostridiales	Bacillales	Clostridiales	Clostridiales	Clostridiales	Clostridiales	Clostridiales
Family	Lachnospiraceae	Peptostreptococcaceae	Lachnospiraceae	Staphylococcaceae	Lachnospiraceae	Lachnospiraceae	Lachnospiraceae	Lachnospiraceae	Lachnospiraceae
Genus	Marvinbryantia	Filifactor	Acetatifactor	Staphylococcus	Clostridium_XlVa	Clostridium_XlVa	Butyrivibrio	Clostridium_XlVa	Clostridium_XlVb
#NAME	ZOTU_0250	ZOTU_0251	ZOTU_0252	ZOTU_0253	ZOTU_0254	ZOTU_0255	ZOTU_0256	ZOTU_0257	ZOTU_0258
CD_49	0	0	0	190	0	0	0	0	0
CD_51	0	0	0	1499	0	0	0	1	0
CD_52	0	0	0	218	0	0	0	0	0
CD_65	6	0	0	68	0	0	0	1	0
CD_66	0	0	0	4	0	0	0	0	0
CD_67	0	0	0	969	0	0	0	0	0
CD_68	0	0	0	4	0	0	0	0	0
CD_78	0	0	0	1304	0	0	0	0	0
CD_79	0	0	0	46	0	0	0	0	0
CDFG_53	4	0	0	167	0	0	0	2	1
CDFG_56	0	0	0	1516	0	0	0	0	0
CDFG_69	2	0	1	120	0	0	0	3	0
CDFG_70	2	0	3	144	0	0	0	7	6
CDFG_71	1	0	0	89	0	0	0	3	0
HF_41	0	0	0	61	0	0	0	0	1
HF_42	1	0	0	82	0	1	0	0	0
HF_43	1	0	0	2	0	0	2	0	3
HF_57	0	0	0	8	0	0	0	0	0
HF_58	0	0	0	90	0	0	0	4	0
HF_72	0	0	0	147	0	0	0	0	4
HF_73	0	0	0	2319	0	0	0	0	0
HF_74	5	0	0	8	0	0	0	1	0
HFFG_45	2	0	0	2	0	1	0	0	0
HFFG_48	0	0	0	194	0	0	1	0	0
HFFG_62	9	0	0	183	0	0	0	0	2
HFFG_77	0	0	0	795	0	0	0	3	0

Kingdom	Bacteria	Bacteria	Bacteria	Bacteria	Bacteria	Bacteria	Bacteria	Bacteria	Bacteria
Phylum	Firmicutes	Firmicutes	Tenericutes	Firmicutes	Tenericutes	Firmicutes	Firmicutes	Tenericutes	Firmicutes
Class	Clostridia	Clostridia	Mollicutes	Clostridia	Mollicutes	Clostridia	Clostridia	Mollicutes	Clostridia
Order	Clostridiales	Clostridiales	Acholeplasmatales	Clostridiales	Acholeplasmatales	Clostridiales	Clostridiales	Entomoplasmatales	Clostridiales
Family	Lachnospiraceae	Lachnospiraceae	Acholeplasmataceae	Lachnospiraceae	Acholeplasmataceae	Clostridiaceae_	Lachnospiraceae	Spiroplasmataceae	Ruminococcaceae
Genus	Clostridium_XlVa	Clostridium_XlVa	Acholeplasma	Clostridium_XlVa	Acholeplasma	Geosporobacter	Clostridium_XlVa	Spiroplasma	Butyricicoccus
#NAME	ZOTU_0259	ZOTU_0260	ZOTU_0261	ZOTU_0262	ZOTU_0263	ZOTU_0264	ZOTU_0265	ZOTU_0266	ZOTU_0267
CD_49	0	0	0	0	0	0	0	0	0
CD_51	0	0	0	0	0	0	0	0	0
CD_52	0	0	0	0	0	0	0	0	0
CD_65	0	0	0	0	0	0	0	0	0
CD_66	0	0	0	0	0	0	0	0	0
CD_67	0	0	0	0	0	0	0	0	0
CD_68	0	0	0	0	0	0	0	0	0
CD_78	0	0	0	0	0	0	0	0	0
CD_79	0	0	0	0	0	0	0	0	0
CDFG_53	0	0	0	0	0	0	0	0	0
CDFG_56	0	0	0	0	0	0	0	0	0
CDFG_69	0	0	1	0	0	0	0	0	0
CDFG_70	3	0	1	0	0	0	0	0	1
CDFG_71	0	0	0	0	0	0	0	0	0
HF_41	0	0	0	0	0	0	0	0	0
HF_42	0	0	0	0	0	0	0	0	0
HF_43	9	0	0	0	0	0	0	0	0
HF_57	0	0	0	0	0	0	0	0	0
HF_58	0	0	0	0	0	0	0	0	0
HF_72	0	0	0	0	0	0	5	0	0
HF_73	0	0	0	0	0	0	0	0	0
HF_74	0	0	0	0	0	0	0	0	0
HFFG_45	0	0	0	0	0	0	0	0	0
HFFG_48	0	1	0	0	0	0	0	0	0
HFFG_62	1	0	0	23	0	0	0	0	1
HFFG_77	0	0	0	0	0	0	0	0	0

Kingdom	Bacteria	Bacteria	Bacteria	Bacteria	Bacteria	Bacteria	Bacteria	Bacteria	Bacteria
Phylum	Firmicutes	Firmicutes	Firmicutes	Firmicutes	Firmicutes	Firmicutes	Firmicutes	Firmicutes	Firmicutes
Class	Clostridia	Clostridia	Clostridia	Clostridia	Clostridia	Clostridia	Clostridia	Erysipelotrichia	Clostridia
Order	Clostridiales	Clostridiales	Clostridiales	Clostridiales	Clostridiales	Clostridiales	Clostridiales	Erysipelotrichales	Clostridiales
Family	Lachnospiraceae	Lachnospiraceae	Lachnospiraceae	Lachnospiraceae	Ruminococcaceae	Lachnospiraceae	Lachnospiraceae	Erysipelotrichaceae	Clostridiaceae_
Genus	Roseburia	Blautia	Clostridium_XlVa	Clostridium_XlVa	Clostridium_IV	Clostridium_XlVa	Clostridium_XlVa	Holdemania	Geosporobacter
#NAME	ZOTU_0268	ZOTU_0269	ZOTU_0270	ZOTU_0271	ZOTU_0272	ZOTU_0273	ZOTU_0274	ZOTU_0275	ZOTU_0276
CD_49	0	0	0	0	0	0	0	0	0
CD_51	0	0	0	3	0	0	0	0	0
CD_52	0	0	0	0	0	0	0	0	0
CD_65	0	0	0	1	0	0	0	0	0
CD_66	0	0	0	0	0	0	0	0	0
CD_67	0	0	0	0	0	0	0	0	0
CD_68	0	0	0	0	1	0	0	0	0
CD_78	0	0	0	0	0	0	0	0	0
CD_79	0	0	0	0	0	0	0	0	0
CDFG_53	0	0	0	1	0	0	0	0	0
CDFG_56	0	0	0	0	0	0	0	0	0
CDFG_69	0	0	0	2	0	0	0	0	0
CDFG_70	0	0	1	4	0	9	0	0	0
CDFG_71	0	0	0	3	0	0	0	1	0
HF_41	0	0	0	0	0	0	0	0	0
HF_42	0	0	0	0	0	0	0	0	0
HF_43	0	0	0	1	0	0	0	0	0
HF_57	0	0	0	1	0	0	0	0	0
HF_58	0	0	0	2	0	0	0	0	0
HF_72	0	0	2	0	0	0	0	0	0
HF_73	0	0	0	0	0	0	0	0	0
HF_74	0	0	0	0	0	0	0	0	0
HFFG_45	0	0	0	3	0	0	0	0	0
HFFG_48	0	0	0	0	0	0	0	0	0
HFFG_62	0	0	0	1	0	0	0	0	0
HFFG_77	0	0	0	2	0	0	0	0	0

Kingdom	Bacteria	Bacteria	Bacteria	Bacteria	Bacteria	Bacteria	Bacteria
Phylum	Firmicutes	Cyanobacteria_Chloroplast	Firmicutes	Firmicutes	Firmicutes	Firmicutes	Firmicutes
Class	Clostridia	Chloroplast	Clostridia	Clostridia	Clostridia	Clostridia	Clostridia
Order	Clostridiales	Chloroplast	Clostridiales	Clostridiales	Clostridiales	Clostridiales	Clostridiales
Family	Ruminococcaceae	Streptophyta	Lachnospiraceae	Lachnospiraceae	Clostridiaceae_	Lachnospiraceae	Lachnospiraceae
Genus	Oscillibacter	NA	Clostridium_XlVa	Lachnospiracea_incertae_sedis	Clostridium_sensu_stricto	Clostridium_XlVa	Lachnospiracea_incertae_sedis
#NAME	ZOTU_0277	ZOTU_0278	ZOTU_0279	ZOTU_0280	ZOTU_0281	ZOTU_0282	ZOTU_0283
CD_49	0	0	0	0	1	0	0
CD_51	0	0	0	0	0	0	0
CD_52	1	0	0	0	0	0	0
CD_65	1	0	0	0	0	0	0
CD_66	0	0	0	0	0	0	0
CD_67	0	0	0	0	0	0	0
CD_68	0	0	0	0	0	0	0
CD_78	0	0	0	0	0	0	0
CD_79	0	0	0	0	0	0	0
CDFG_53	0	4	0	0	0	0	1
CDFG_56	0	1	0	0	0	0	0
CDFG_69	0	4	0	0	0	0	0
CDFG_70	1	0	0	0	0	0	0
CDFG_71	0	0	0	0	0	0	0
HF_41	0	0	0	0	0	0	0
HF_42	0	0	0	0	0	0	0
HF_43	0	0	0	0	0	0	0
HF_57	0	0	0	0	0	0	0
HF_58	0	0	0	0	0	0	0
HF_72	0	0	0	0	0	0	0
HF_73	0	0	1	0	0	0	0
HF_74	0	0	0	0	0	0	0
HFFG_45	0	32	0	0	0	0	0
HFFG_48	0	1	0	0	0	0	0
HFFG_62	0	40	0	0	0	0	0
HFFG_77	0	0	0	0	0	0	0

Kingdom	Bacteria	Bacteria	Bacteria	Bacteria	Bacteria	Bacteria	Bacteria	Bacteria	Bacteria
Phylum	Tenericutes	Firmicutes	Firmicutes	Firmicutes	Firmicutes	Firmicutes	Firmicutes	Firmicutes	Firmicutes
Class	Mollicutes	Clostridia	Clostridia	Clostridia	Clostridia	Clostridia	Clostridia	Clostridia	Bacilli
Order	Entomoplasmatales	Clostridiales	Clostridiales	Clostridiales	Clostridiales	Clostridiales	Clostridiales	Halanaerobiales	Bacillales
Family	Spiroplasmataceae	Clostridiaceae_	Lachnospiraceae	Lachnospiraceae	Ruminococcaceae	Lachnospiraceae	Lachnospiraceae	Halanaerobiaceae	Bacillaceae_
Genus	Spiroplasma	Alkaliphilus	Clostridium_XlVa	Clostridium_XlVa	Intestinimonas	Clostridium_XlVa	Clostridium_XlVa	Halanaerobium	Bacillus
#NAME	ZOTU_0284	ZOTU_0285	ZOTU_0286	ZOTU_0287	ZOTU_0288	ZOTU_0289	ZOTU_0290	ZOTU_0291	ZOTU_0292
CD_49	0	0	0	0	0	0	0	0	2
CD_51	0	0	0	0	0	0	0	0	3
CD_52	0	0	0	0	0	0	0	1	3
CD_65	0	0	0	0	0	0	0	0	0
CD_66	0	0	0	1	0	0	1	0	0
CD_67	0	0	0	0	0	0	0	0	268
CD_68	0	0	0	0	0	0	0	0	0
CD_78	0	0	0	0	0	0	0	0	0
CD_79	0	0	0	0	0	0	0	0	0
CDFG_53	0	0	0	0	0	0	0	69	1
CDFG_56	0	0	0	0	0	0	0	0	0
CDFG_69	0	0	0	0	0	0	0	0	0
CDFG_70	0	6	2	1	3	0	0	0	2
CDFG_71	0	0	0	0	0	0	0	0	0
HF_41	0	0	0	0	0	0	0	0	0
HF_42	0	0	0	0	0	0	0	0	0
HF_43	0	0	1	0	0	1	0	2	0
HF_57	0	0	0	0	0	0	0	0	0
HF_58	0	0	0	0	0	0	0	0	5
HF_72	0	0	0	1	0	0	0	0	0
HF_73	0	0	0	0	0	0	0	0	5
HF_74	0	0	0	1	0	0	0	0	0
HFFG_45	0	0	0	5	0	0	0	1	0
HFFG_48	0	0	0	0	0	0	0	0	0
HFFG_62	0	0	0	1	0	0	1	2	1
HFFG_77	0	0	0	0	0	0	0	1	1

Kingdom	Bacteria	Bacteria	Bacteria	Bacteria	Bacteria	Bacteria	Bacteria	Bacteria	Bacteria
Phylum	Firmicutes	Firmicutes	Proteobacteria	Firmicutes	Firmicutes	Actinobacteria	Firmicutes	Firmicutes	Firmicutes
Class	Bacilli	Clostridia	Gammaproteobacteria	Clostridia	Clostridia	Actinobacteria	Clostridia	Clostridia	Clostridia
Order	Lactobacillales	Clostridiales	Pasteurellales	Clostridiales	Clostridiales	Actinomycetales	Clostridiales	Clostridiales	Clostridiales
Family	Streptococcaceae	Ruminococcaceae	Pasteurellaceae	Ruminococcaceae	Lachnospiraceae	Streptomycetaceae	Lachnospiraceae	Lachnospiraceae	Lachnospiraceae
Genus	Streptococcus	Clostridium_IV	Haemophilus	Sporobacter	Clostridium_XlVa	Streptomyces	Clostridium_XlVa	Blautia	Clostridium_XlVa
#NAME	ZOTU_0293	ZOTU_0294	ZOTU_0295	ZOTU_0296	ZOTU_0297	ZOTU_0298	ZOTU_0299	ZOTU_0300	ZOTU_0301
CD_49	0	0	0	0	0	2	0	0	0
CD_51	0	0	0	0	0	0	0	0	1
CD_52	0	0	0	1	0	0	0	0	0
CD_65	2	0	0	0	3	0	0	0	5
CD_66	0	0	0	0	0	0	0	0	1
CD_67	0	0	0	0	0	0	0	0	0
CD_68	0	0	0	0	0	0	0	0	0
CD_78	0	0	0	0	0	0	0	0	0
CD_79	0	0	0	0	0	0	0	0	0
CDFG_53	0	1	0	0	0	1	0	0	0
CDFG_56	0	0	0	0	0	0	0	0	0
CDFG_69	0	0	1	0	0	0	0	0	1
CDFG_70	3	1	0	6	1	0	0	0	4
CDFG_71	0	0	0	0	1	0	0	0	0
HF_41	1	0	0	0	0	0	0	0	0
HF_42	1	0	0	0	0	0	0	1	0
HF_43	1	1	0	0	0	0	0	0	0
HF_57	0	0	0	0	0	1	0	0	0
HF_58	0	0	2	0	0	0	0	0	0
HF_72	0	0	0	0	0	0	0	0	1
HF_73	1	0	0	0	0	0	0	0	0
HF_74	0	0	0	0	0	0	0	0	1
HFFG_45	0	0	1	0	0	0	0	0	1
HFFG_48	1	0	0	0	0	0	0	0	0
HFFG_62	6	0	9	1	0	9	0	0	1
HFFG_77	0	0	1	0	0	1	0	0	1

Kingdom	Bacteria	Bacteria	Bacteria	Bacteria	Bacteria	Bacteria	Bacteria	Bacteria	Bacteria
Phylum	Firmicutes	Firmicutes	Firmicutes	Firmicutes	Tenericutes	Firmicutes	Firmicutes	Firmicutes	Firmicutes
Class	Clostridia	Bacilli	Bacilli	Clostridia	Mollicutes	Clostridia	Clostridia	Clostridia	Clostridia
Order	Clostridiales	Bacillales	Lactobacillales	Clostridiales	Acholeplasmatales	Clostridiales	Clostridiales	Clostridiales	Clostridiales
Family	Lachnospiraceae	Bacillaceae_	Enterococcaceae	Lachnospiraceae	Acholeplasmataceae	Ruminococcaceae	Lachnospiraceae	Ruminococcaceae	Ruminococcaceae
Genus	Acetatifactor	Bacillus	Enterococcus	Clostridium_XlVa	Acholeplasma	Anaerotruncus	Acetatifactor	Flavonifractor	Ethanoligenens
#NAME	ZOTU_0302	ZOTU_0303	ZOTU_0304	ZOTU_0305	ZOTU_0306	ZOTU_0307	ZOTU_0308	ZOTU_0309	ZOTU_0310
CD_49	0	0	54	0	0	0	0	0	0
CD_51	0	0	3	0	0	0	0	0	0
CD_52	0	0	41	0	0	0	0	0	0
CD_65	0	0	4	0	0	0	2	0	1
CD_66	0	0	4	0	0	0	0	0	0
CD_67	0	0	2	0	0	0	0	0	0
CD_68	0	0	0	0	0	0	0	0	0
CD_78	0	0	0	0	0	0	0	0	0
CD_79	0	0	0	0	0	0	0	0	0
CDFG_53	0	0	21	0	0	0	0	0	0
CDFG_56	0	0	30	0	0	0	0	0	0
CDFG_69	0	0	0	0	0	0	2	0	0
CDFG_70	3	0	1	0	0	0	1	0	7
CDFG_71	0	0	0	0	0	0	1	0	0
HF_41	0	0	9	0	0	0	0	0	0
HF_42	0	0	3	0	0	0	0	0	0
HF_43	0	0	21	0	0	0	0	0	1
HF_57	0	0	2	0	0	0	0	0	0
HF_58	0	0	12	0	0	0	0	0	0
HF_72	0	0	2	0	0	0	0	0	0
HF_73	0	0	100	0	0	0	0	0	0
HF_74	0	0	43	0	0	0	0	0	0
HFFG_45	0	0	1	0	0	0	0	0	0
HFFG_48	0	0	2	0	0	0	0	0	0
HFFG_62	0	1	13	0	0	0	0	0	0
HFFG_77	0	0	30	0	0	0	0	0	0

Kingdom	Bacteria	Bacteria	Bacteria	Bacteria	Bacteria	Bacteria	Bacteria	Bacteria
Phylum	Firmicutes	Firmicutes	Proteobacteria	Firmicutes	Firmicutes	Firmicutes	Firmicutes	Firmicutes
Class	Clostridia	Erysipelotrichia	Betaproteobacteria	Clostridia	Clostridia	Clostridia	Clostridia	Clostridia
Order	Clostridiales	Erysipelotrichales	Neisseriales	Clostridiales	Clostridiales	Clostridiales	Clostridiales	Clostridiales
Family	Lachnospiraceae	Erysipelotrichaceae	Neisseriaceae	Lachnospiraceae	Lachnospiraceae	Clostridiaceae_	Lachnospiraceae	Lachnospiraceae
Genus	Butyrivibrio	Holdemania	Neisseria	Ruminococcus	Lachnospiracea_incertae_sedis	Alkaliphilus	Clostridium_XlVa	Butyrivibrio
#NAME	ZOTU_0311	ZOTU_0312	ZOTU_0313	ZOTU_0314	ZOTU_0315	ZOTU_0316	ZOTU_0317	ZOTU_0318
CD_49	0	0	0	0	0	0	0	0
CD_51	0	0	2	0	0	0	0	0
CD_52	0	0	0	0	0	0	0	0
CD_65	1	0	0	0	3	0	0	0
CD_66	0	0	0	0	0	0	0	0
CD_67	0	0	0	0	0	0	0	0
CD_68	0	0	0	0	0	0	0	0
CD_78	0	0	0	1	0	0	0	0
CD_79	0	0	0	0	0	0	0	0
CDFG_53	0	0	0	0	0	0	0	0
CDFG_56	0	0	1	0	0	0	0	0
CDFG_69	0	0	0	0	0	0	0	0
CDFG_70	7	0	0	17	33	0	0	0
CDFG_71	0	0	0	3	0	0	0	0
HF_41	0	0	3	0	0	0	0	0
HF_42	0	0	2	0	0	0	0	0
HF_43	3	0	0	0	0	0	0	0
HF_57	0	0	0	0	0	0	0	0
HF_58	1	0	3	0	0	0	0	0
HF_72	0	0	0	0	0	0	0	0
HF_73	0	1	0	0	0	0	0	0
HF_74	0	0	1	0	0	0	0	0
HFFG_45	0	0	0	0	0	0	0	0
HFFG_48	0	0	2	0	0	0	0	0
HFFG_62	0	0	0	0	0	0	0	0
HFFG_77	3	0	0	0	0	0	0	0

Kingdom	Bacteria	Bacteria	Bacteria	Bacteria	Bacteria	Bacteria	Bacteria	Bacteria
Phylum	Firmicutes	Firmicutes	Firmicutes	Firmicutes	Firmicutes	Bacteroidetes	Firmicutes	Firmicutes
Class	Clostridia	Clostridia	Clostridia	Clostridia	Clostridia	Bacteroidia	Clostridia	Clostridia
Order	Clostridiales	Clostridiales	Clostridiales	Clostridiales	Clostridiales	Bacteroidales	Clostridiales	Clostridiales
Family	Clostridiaceae_	Lachnospiraceae	Lachnospiraceae	Clostridiaceae_	Lachnospiraceae	Porphyromonadaceae	Lachnospiraceae	Gracilibacteraceae
Genus	Clostridium_sensu_stricto	Butyrivibrio	Clostridium_XlVa	Clostridium_sensu_stricto	Clostridium_XlVa	Parabacteroides	Clostridium_XlVa	Lutispora
#NAME	ZOTU_0319	ZOTU_0320	ZOTU_0321	ZOTU_0322	ZOTU_0323	ZOTU_0324	ZOTU_0325	ZOTU_0326
CD_49	0	0	0	0	0	0	0	0
CD_51	0	0	0	0	0	0	0	0
CD_52	0	0	0	0	0	0	0	0
CD_65	0	0	0	0	0	0	0	1
CD_66	0	0	0	0	0	0	0	0
CD_67	0	0	0	0	0	0	0	0
CD_68	0	0	0	0	0	0	0	0
CD_78	0	0	0	0	0	0	0	0
CD_79	0	0	0	0	0	0	0	0
CDFG_53	0	0	0	0	0	0	0	0
CDFG_56	0	0	0	0	0	0	0	0
CDFG_69	0	0	0	0	0	0	0	1
CDFG_70	0	0	0	0	0	0	0	4
CDFG_71	0	0	0	0	0	0	0	0
HF_41	0	0	0	0	0	0	0	0
HF_42	0	0	0	0	0	0	0	0
HF_43	0	0	0	0	0	0	2	0
HF_57	0	0	0	0	0	0	0	0
HF_58	0	0	0	0	0	0	0	0
HF_72	0	0	0	0	0	0	0	0
HF_73	0	0	0	0	0	0	0	0
HF_74	0	0	0	0	0	0	0	1
HFFG_45	0	0	0	0	0	0	0	13
HFFG_48	0	0	0	0	0	0	0	0
HFFG_62	0	0	0	0	0	0	0	1
HFFG_77	0	0	0	0	0	0	0	1

Kingdom	Bacteria	Bacteria	Bacteria	Bacteria	Bacteria	Bacteria	Bacteria	Bacteria
Phylum	Firmicutes	Firmicutes	Firmicutes	Firmicutes	Firmicutes	Firmicutes	Firmicutes	Bacteroidetes
Class	Clostridia	Clostridia	Clostridia	Clostridia	Clostridia	Clostridia	Clostridia	Bacteroidia
Order	Clostridiales	Clostridiales	Clostridiales	Clostridiales	Clostridiales	Clostridiales	Clostridiales	Bacteroidales
Family	Lachnospiraceae	Lachnospiraceae	Peptostreptococcaceae	Ruminococcaceae	Clostridiales_Incertae_Sedis_XI	Lachnospiraceae	Ruminococcaceae	Porphyromonadaceae
Genus	Clostridium_XlVa	Ruminococcus	Clostridium_XI	Clostridium_IV	Tissierella	Acetitomaculum	Oscillibacter	Barnesiella
#NAME	ZOTU_0327	ZOTU_0328	ZOTU_0329	ZOTU_0330	ZOTU_0331	ZOTU_0332	ZOTU_0333	ZOTU_0334
CD_49	0	0	0	0	0	0	0	0
CD_51	0	0	0	0	0	0	0	0
CD_52	0	0	0	0	0	0	0	0
CD_65	0	0	0	0	1	1	0	0
CD_66	0	0	0	0	0	2	0	0
CD_67	0	0	0	0	0	0	0	0
CD_68	0	0	0	0	0	0	0	0
CD_78	0	0	0	0	0	0	0	0
CD_79	0	0	0	0	0	0	0	0
CDFG_53	0	0	0	0	0	0	0	0
CDFG_56	0	0	0	0	0	0	0	2
CDFG_69	0	0	0	0	0	0	1	0
CDFG_70	1	22	1	0	5	0	6	0
CDFG_71	0	1	0	0	1	0	0	0
HF_41	0	0	0	0	0	0	0	0
HF_42	0	0	0	0	0	0	0	0
HF_43	0	0	0	0	0	0	0	2
HF_57	0	0	2	0	0	0	0	0
HF_58	0	0	0	0	0	0	0	0
HF_72	0	0	0	0	0	3	0	0
HF_73	0	0	3	0	0	0	0	0
HF_74	0	0	0	0	0	0	0	0
HFFG_45	0	0	0	0	0	0	0	0
HFFG_48	0	0	3	0	0	0	0	0
HFFG_62	0	0	4	0	0	0	0	0
HFFG_77	0	0	0	0	0	3	0	0

Kingdom	Bacteria	Bacteria	Bacteria	Bacteria	Bacteria	Bacteria	Bacteria	Bacteria
Phylum	Firmicutes	Firmicutes	Bacteroidetes	Firmicutes	Firmicutes	Firmicutes	Firmicutes	Firmicutes
Class	Clostridia	Clostridia	Bacteroidia	Clostridia	Clostridia	Clostridia	Clostridia	Clostridia
Order	Clostridiales	Clostridiales	Bacteroidales	Clostridiales	Clostridiales	Clostridiales	Clostridiales	Clostridiales
Family	Ruminococcaceae	Lachnospiraceae	Porphyromonadaceae	Lachnospiraceae	Lachnospiraceae	Lachnospiraceae	Lachnospiraceae	Ruminococcaceae
Genus	Sporobacter	Anaerosporobacter	Parabacteroides	Clostridium_XlVa	Clostridium_XlVa	Lachnospiracea_incertae_sedis	Clostridium_XlVa	Clostridium_IV
#NAME	ZOTU_0335	ZOTU_0336	ZOTU_0337	ZOTU_0338	ZOTU_0339	ZOTU_0340	ZOTU_0341	ZOTU_0342
CD_49	0	0	0	0	0	0	0	0
CD_51	0	0	0	0	0	0	0	0
CD_52	0	0	0	0	0	0	0	0
CD_65	0	1	0	0	0	0	0	0
CD_66	0	0	0	0	0	0	0	0
CD_67	0	0	0	0	0	0	0	0
CD_68	0	0	0	0	0	0	0	0
CD_78	0	0	0	0	0	0	0	0
CD_79	0	0	0	0	0	0	0	0
CDFG_53	0	0	0	0	0	0	0	0
CDFG_56	0	0	0	0	0	0	0	0
CDFG_69	0	0	0	0	0	0	0	0
CDFG_70	0	5	0	0	1	0	0	0
CDFG_71	0	0	0	0	0	0	0	0
HF_41	0	0	0	0	0	0	0	0
HF_42	0	0	0	0	0	0	0	0
HF_43	0	0	0	0	0	0	0	0
HF_57	0	0	0	0	0	0	0	0
HF_58	0	0	0	0	0	0	0	0
HF_72	0	0	0	0	0	0	0	0
HF_73	0	0	0	0	0	0	0	0
HF_74	0	0	0	0	0	0	0	0
HFFG_45	0	0	0	0	0	0	3	0
HFFG_48	0	0	0	0	0	0	0	0
HFFG_62	0	1	0	0	0	0	0	0
HFFG_77	0	0	0	0	0	0	0	0

Kingdom	Bacteria	Bacteria	Bacteria	Bacteria	Bacteria	Bacteria	Bacteria	Bacteria	Bacteria
Phylum	Firmicutes	Firmicutes	Firmicutes	Firmicutes	Firmicutes	Firmicutes	Firmicutes	Firmicutes	Firmicutes
Class	Clostridia	Clostridia	Clostridia	Clostridia	Bacilli	Clostridia	Clostridia	Clostridia	Clostridia
Order	Clostridiales	Clostridiales	Clostridiales	Clostridiales	Bacillales	Clostridiales	Clostridiales	Clostridiales	Clostridiales
Family	Clostridiaceae_	Lachnospiraceae	Lachnospiraceae	Lachnospiraceae	Paenibacillaceae_	Lachnospiraceae	Lachnospiraceae	Lachnospiraceae	Lachnospiraceae
Genus	Clostridium_sensu_stricto	Blautia	Acetitomaculum	Lachnobacterium	Paenibacillus	Ruminococcus	Marvinbryantia	Acetatifactor	Blautia
#NAME	ZOTU_0343	ZOTU_0344	ZOTU_0345	ZOTU_0346	ZOTU_0347	ZOTU_0348	ZOTU_0349	ZOTU_0350	ZOTU_0351
CD_49	0	0	0	0	0	0	0	0	0
CD_51	0	0	0	0	0	0	0	0	0
CD_52	0	0	0	0	0	0	0	0	0
CD_65	0	0	0	0	0	0	0	0	0
CD_66	0	0	0	0	0	0	0	0	0
CD_67	0	0	0	0	0	0	0	0	0
CD_68	0	0	0	0	0	0	0	0	0
CD_78	0	0	0	0	0	0	0	0	0
CD_79	0	0	0	0	0	0	0	0	0
CDFG_53	0	0	0	0	1	0	3	0	0
CDFG_56	0	0	0	0	2	1	0	0	0
CDFG_69	0	0	0	2	0	0	0	0	0
CDFG_70	0	0	0	9	0	17	0	0	2
CDFG_71	0	0	0	0	0	0	0	0	0
HF_41	0	0	0	0	0	0	0	0	0
HF_42	0	0	0	0	0	0	0	0	0
HF_43	0	0	0	0	0	0	3	0	0
HF_57	0	0	0	0	0	0	0	0	0
HF_58	0	0	0	0	0	0	0	0	0
HF_72	0	0	0	2	0	0	0	0	0
HF_73	0	0	0	0	0	0	0	0	0
HF_74	0	0	0	0	0	0	0	0	0
HFFG_45	0	0	0	0	0	0	1	0	0
HFFG_48	0	0	0	0	4	0	0	0	0
HFFG_62	0	0	0	0	0	0	1	0	0
HFFG_77	0	0	0	0	0	0	0	0	0

Kingdom	Bacteria	Bacteria	Bacteria	Bacteria	Bacteria	Bacteria	Bacteria	Bacteria	Bacteria
Phylum	Firmicutes	Firmicutes	Firmicutes	Firmicutes	Firmicutes	Firmicutes	Firmicutes	Actinobacteria	Firmicutes
Class	Clostridia	Clostridia	Clostridia	Clostridia	Clostridia	Erysipelotrichia	Bacilli	Actinobacteria	Bacilli
Order	Clostridiales	Clostridiales	Clostridiales	Clostridiales	Clostridiales	Erysipelotrichales	Lactobacillales	Coriobacteriales	Lactobacillales
Family	Ruminococcaceae	Lachnospiraceae	Lachnospiraceae	Lachnospiraceae	Ruminococcaceae	Erysipelotrichaceae	Lactobacillaceae	Coriobacteriaceae	Lactobacillaceae
Genus	Intestinimonas	Acetatifactor	Acetatifactor	Shuttleworthia	Clostridium_IV	Catenisphaera	Lactobacillus	Enterorhabdus	Lactobacillus
#NAME	ZOTU_0352	ZOTU_0353	ZOTU_0354	ZOTU_0355	ZOTU_0356	ZOTU_0357	ZOTU_0358	ZOTU_0359	ZOTU_0360
CD_49	0	0	0	0	0	0	0	1	0
CD_51	0	0	0	0	0	0	0	10	0
CD_52	0	0	0	0	0	0	0	10	0
CD_65	0	0	0	0	0	0	0	8	0
CD_66	0	0	0	0	0	0	0	27	0
CD_67	0	0	0	0	0	0	0	9	0
CD_68	0	0	0	0	0	0	0	8	0
CD_78	0	0	0	0	0	0	0	1	0
CD_79	0	0	0	0	0	0	0	0	0
CDFG_53	0	0	0	0	0	0	1	23	0
CDFG_56	0	0	0	0	0	0	0	0	0
CDFG_69	0	0	1	1	0	0	2	12	0
CDFG_70	0	2	4	0	0	0	0	27	0
CDFG_71	0	0	0	0	0	0	0	2	0
HF_41	0	0	0	0	0	0	0	0	0
HF_42	0	0	0	0	0	0	0	0	0
HF_43	0	1	0	0	0	0	0	0	0
HF_57	0	0	0	0	0	0	0	0	0
HF_58	0	0	0	0	0	0	0	7	0
HF_72	0	0	0	0	0	0	0	0	0
HF_73	0	0	0	0	0	0	0	0	0
HF_74	0	0	0	0	0	0	0	2	0
HFFG_45	0	0	0	2	0	0	4	0	0
HFFG_48	0	0	0	0	0	0	0	0	0
HFFG_62	0	0	0	0	0	1	9	1	0
HFFG_77	0	0	0	0	0	0	0	0	0

Kingdom	Bacteria	Bacteria	Bacteria	Bacteria	Bacteria	Bacteria	Bacteria	Bacteria
Phylum	Proteobacteria	Firmicutes	Firmicutes	Firmicutes	Firmicutes	Firmicutes	Firmicutes	Firmicutes
Class	Gammaproteobacteria	Bacilli	Clostridia	Clostridia	Clostridia	Clostridia	Clostridia	Clostridia
Order	Pseudomonadales	Bacillales	Clostridiales	Clostridiales	Clostridiales	Clostridiales	Clostridiales	Clostridiales
Family	Pseudomonadaceae	Staphylococcaceae	Natranaerovirga	Lachnospiraceae	Lachnospiraceae	Lachnospiraceae	Lachnospiraceae	Clostridiaceae_
Genus	Pseudomonas	Staphylococcus	NA	Clostridium_XlVa	Clostridium_XlVa	Clostridium_XlVa	Lachnospiracea_incertae_sedis	Alkaliphilus
#NAME	ZOTU_0361	ZOTU_0362	ZOTU_0363	ZOTU_0364	ZOTU_0365	ZOTU_0366	ZOTU_0367	ZOTU_0368
CD_49	1	33	0	0	0	0	0	0
CD_51	0	56	0	0	0	0	0	0
CD_52	0	112	0	0	0	0	0	0
CD_65	0	42	0	0	0	0	0	0
CD_66	0	25	0	0	0	0	0	0
CD_67	0	255	0	0	0	0	0	0
CD_68	0	228	0	0	0	0	0	0
CD_78	0	12	0	0	0	0	0	0
CD_79	0	0	0	0	0	0	0	0
CDFG_53	1	39	0	0	0	0	0	0
CDFG_56	0	36	0	0	0	0	0	0
CDFG_69	0	306	0	0	1	0	0	0
CDFG_70	0	178	0	0	0	0	0	0
CDFG_71	0	23	0	0	0	0	0	0
HF_41	0	13	0	0	0	0	0	0
HF_42	1	1	0	0	0	0	0	0
HF_43	1	2	0	0	0	0	0	0
HF_57	0	20	0	0	0	0	0	0
HF_58	1	565	0	0	0	0	0	0
HF_72	3	25	0	0	0	0	0	0
HF_73	0	89	0	0	0	0	0	0
HF_74	0	47	0	0	0	0	0	0
HFFG_45	1	2	0	0	0	0	0	0
HFFG_48	2	6	0	0	0	0	0	0
HFFG_62	3	120	0	0	0	0	0	0
HFFG_77	2	1131	0	0	0	0	0	0

Kingdom	Bacteria	Bacteria	Bacteria	Bacteria	Bacteria	Bacteria	Bacteria	Bacteria	Bacteria
Phylum	Firmicutes	Firmicutes	Actinobacteria	Firmicutes	Firmicutes	Firmicutes	Firmicutes	Firmicutes	Firmicutes
Class	Clostridia	Clostridia	Actinobacteria	Bacilli	Bacilli	Bacilli	Bacilli	Clostridia	Clostridia
Order	Clostridiales	Clostridiales	Actinomycetales	Lactobacillales	Bacillales	Lactobacillales	Bacillales	Clostridiales	Clostridiales
Family	Clostridiaceae_	Peptostreptococcaceae	Dietziaceae	Enterococcaceae	Planococcaceae	Streptococcaceae	Bacillaceae_	Lachnospiraceae	Ruminococcaceae
Genus	Clostridium_sensu_stricto	Clostridium_XI	Dietzia	Enterococcus	Viridibacillus	Streptococcus	Bacillus	Acetatifactor	Clostridium_IV
#NAME	ZOTU_0369	ZOTU_0370	ZOTU_0371	ZOTU_0372	ZOTU_0373	ZOTU_0374	ZOTU_0375	ZOTU_0376	ZOTU_0377
CD_49	0	0	0	0	27	0	12	0	0
CD_51	0	0	0	0	491	0	6	0	0
CD_52	0	1	0	0	30	0	7	0	0
CD_65	0	0	0	0	26	0	9	0	0
CD_66	0	0	0	0	12	0	6	0	0
CD_67	0	0	0	0	113	0	54	0	0
CD_68	0	0	0	0	251	0	401	0	0
CD_78	0	0	0	0	276	0	2	0	0
CD_79	0	0	0	0	15	0	0	0	0
CDFG_53	0	0	0	1	30	0	4	0	0
CDFG_56	0	0	0	0	518	0	2	0	0
CDFG_69	0	0	0	0	208	1	15	0	0
CDFG_70	0	1	0	0	116	0	2	0	0
CDFG_71	0	0	0	0	15	0	1	0	0
HF_41	0	0	0	0	15	0	7	0	0
HF_42	0	2	0	0	31	0	0	0	0
HF_43	0	0	0	0	0	0	2	0	0
HF_57	0	0	0	0	24	0	1	0	0
HF_58	0	0	1	0	341	1	12	0	0
HF_72	0	1	0	0	33	0	10	0	0
HF_73	0	0	0	0	558	0	65	0	0
HF_74	0	0	0	0	40	0	4	0	0
HFFG_45	0	5	0	0	0	1	0	1	0
HFFG_48	0	0	0	2	48	0	0	0	0
HFFG_62	0	0	0	0	119	4	2	0	0
HFFG_77	0	0	0	0	904	0	26	0	0

Kingdom	Bacteria	Bacteria	Bacteria	Bacteria	Bacteria	Bacteria	Bacteria	Bacteria
Phylum	Firmicutes	Firmicutes	Firmicutes	Firmicutes	Firmicutes	Firmicutes	Firmicutes	Firmicutes
Class	Clostridia	Clostridia	Clostridia	Clostridia	Bacilli	Clostridia	Clostridia	Clostridia
Order	Clostridiales	Clostridiales	Clostridiales	Clostridiales	Bacillales	Clostridiales	Clostridiales	Clostridiales
Family	Ruminococcaceae	Lachnospiraceae	Lachnospiraceae	Clostridiaceae_	Planococcaceae	Lachnospiraceae	Clostridiaceae_	Lachnospiraceae
Genus	Oscillibacter	Acetatifactor	Clostridium_XlVa	Clostridium_sensu_stricto	Viridibacillus	Clostridium_XlVa	Clostridium_sensu_stricto	Clostridium_XlVa
#NAME	ZOTU_0378	ZOTU_0379	ZOTU_0380	ZOTU_0381	ZOTU_0382	ZOTU_0383	ZOTU_0384	ZOTU_0385
CD_49	0	0	0	0	14	0	0	0
CD_51	0	0	0	0	27	0	0	0
CD_52	0	0	0	0	9	0	0	0
CD_65	0	0	0	0	16	1	0	0
CD_66	0	0	0	0	16	0	0	0
CD_67	0	0	0	0	37	0	0	0
CD_68	0	0	0	0	64	0	0	0
CD_78	0	0	0	0	44	0	0	0
CD_79	0	0	0	0	4	0	0	0
CDFG_53	0	0	0	0	14	0	0	0
CDFG_56	0	0	0	0	153	0	0	0
CDFG_69	0	0	0	0	145	0	0	0
CDFG_70	2	0	0	0	100	1	0	0
CDFG_71	0	0	0	0	8	0	0	0
HF_41	0	0	0	0	4	0	0	0
HF_42	0	0	0	0	2	0	0	0
HF_43	0	0	0	0	0	0	0	0
HF_57	0	0	0	0	13	0	0	0
HF_58	0	0	0	0	262	0	0	0
HF_72	0	0	0	0	16	0	0	0
HF_73	0	0	0	0	127	0	0	0
HF_74	0	0	0	0	20	0	0	0
HFFG_45	0	0	0	0	0	0	0	0
HFFG_48	0	0	0	0	8	0	0	0
HFFG_62	0	0	0	0	69	0	0	0
HFFG_77	0	0	0	0	710	0	0	0

Kingdom	Bacteria	Bacteria	Bacteria	Bacteria	Bacteria	Bacteria	Bacteria	Bacteria
Phylum	Firmicutes	Firmicutes	Firmicutes	Proteobacteria	Cyanobacteria_Chloroplast	Tenericutes	Cyanobacteria_Chloroplast	Firmicutes
Class	Clostridia	Clostridia	Negativicutes	Betaproteobacteria	Chloroplast	Mollicutes	Chloroplast	Clostridia
Order	Clostridiales	Clostridiales	Selenomonadales	Neisseriales	Chloroplast	Acholeplasmatales	Chloroplast	Clostridiales
Family	Ruminococcaceae	Clostridiaceae_	Veillonellaceae	Neisseriaceae	Streptophyta	Acholeplasmataceae	Streptophyta	Lachnospiraceae
Genus	Clostridium_IV	Alkaliphilus	Veillonella	Neisseria	NA	Acholeplasma	NA	Coprococcus
#NAME	ZOTU_0386	ZOTU_0387	ZOTU_0388	ZOTU_0389	ZOTU_0390	ZOTU_0391	ZOTU_0392	ZOTU_0393
CD_49	0	0	0	0	0	0	0	0
CD_51	0	0	0	0	0	0	0	0
CD_52	0	0	0	0	0	0	0	0
CD_65	0	0	0	0	0	0	0	0
CD_66	0	0	0	0	0	0	0	0
CD_67	0	0	0	0	0	0	0	0
CD_68	0	0	0	0	0	0	0	0
CD_78	0	0	0	0	0	0	0	0
CD_79	0	0	0	0	0	0	0	0
CDFG_53	0	0	0	0	0	0	0	0
CDFG_56	0	0	0	0	0	0	0	0
CDFG_69	0	0	0	0	0	0	0	0
CDFG_70	0	0	0	0	1	0	0	0
CDFG_71	0	0	0	0	0	0	0	0
HF_41	0	0	0	0	0	0	0	0
HF_42	0	0	0	0	0	0	0	0
HF_43	0	0	0	0	0	0	0	0
HF_57	0	0	0	2	0	0	0	0
HF_58	0	0	0	0	0	0	0	0
HF_72	0	0	0	0	0	0	0	1
HF_73	0	0	0	0	0	0	0	0
HF_74	0	0	0	0	0	0	0	1
HFFG_45	0	0	0	0	9	0	9	0
HFFG_48	0	0	0	0	2	0	0	0
HFFG_62	0	0	7	0	0	0	3	0
HFFG_77	0	0	0	9	0	0	0	0

Kingdom	Bacteria	Bacteria	Bacteria	Bacteria	Bacteria	Bacteria	Bacteria
Phylum	Bacteroidetes	Firmicutes	Bacteroidetes	Proteobacteria	Actinobacteria	Firmicutes	Firmicutes
Class	Bacteroidia	Negativicutes	Bacteroidia	Betaproteobacteria	Actinobacteria	Clostridia	Clostridia
Order	Bacteroidales	Selenomonadales	Bacteroidales	Burkholderiales	Actinomycetales	Clostridiales	Clostridiales
Family	Porphyromonadaceae	Veillonellaceae	Porphyromonadaceae	Burkholderiaceae	Corynebacteriaceae	Clostridiales_Incertae_Sedis_XIII	Lachnospiraceae
Genus	Parabacteroides	Veillonella	Parabacteroides	Burkholderia	Corynebacterium	Anaerovorax	Acetatifactor
#NAME	ZOTU_0394	ZOTU_0395	ZOTU_0396	ZOTU_0397	ZOTU_0398	ZOTU_0399	ZOTU_0400
CD_49	0	0	0	1	0	0	0
CD_51	0	0	0	0	0	0	0
CD_52	0	0	0	0	0	0	0
CD_65	1	0	0	0	0	0	0
CD_66	0	0	0	0	0	0	0
CD_67	6	0	0	0	0	0	0
CD_68	5	0	0	0	0	0	0
CD_78	0	0	0	0	0	0	0
CD_79	0	0	0	0	0	0	0
CDFG_53	0	0	0	0	0	0	0
CDFG_56	0	0	0	0	1	0	0
CDFG_69	0	0	0	0	0	0	0
CDFG_70	0	0	0	1	0	0	0
CDFG_71	0	0	0	0	0	0	0
HF_41	0	0	0	0	0	0	0
HF_42	0	1	0	1	0	0	0
HF_43	0	1	0	1	0	0	0
HF_57	0	0	0	0	1	0	0
HF_58	0	0	0	0	0	0	0
HF_72	0	1	0	6	0	0	0
HF_73	0	0	0	1	0	0	0
HF_74	0	0	0	0	0	0	0
HFFG_45	0	0	0	1	0	0	0
HFFG_48	0	2	0	0	1	0	0
HFFG_62	0	0	0	1	0	0	0
HFFG_77	0	2	0	1	0	0	0

Kingdom	Bacteria	Bacteria	Bacteria	Bacteria	Bacteria	Bacteria	Bacteria	Bacteria
Phylum	Firmicutes	Proteobacteria	Firmicutes	Firmicutes	Actinobacteria	Firmicutes	Firmicutes	Firmicutes
Class	Erysipelotrichia	Betaproteobacteria	Negativicutes	Clostridia	Actinobacteria	Clostridia	Clostridia	Clostridia
Order	Erysipelotrichales	Burkholderiales	Selenomonadales	Clostridiales	Actinomycetales	Clostridiales	Clostridiales	Clostridiales
Family	Erysipelotrichaceae	Burkholderiaceae	Acidaminococcaceae	Eubacteriaceae	Corynebacteriaceae	Lachnospiraceae	Ruminococcaceae	Ruminococcaceae
Genus	Clostridium_XVIII	Ralstonia	Phascolarctobacterium	Anaerofustis	Corynebacterium	Acetatifactor	Oscillibacter	Ruminococcus
#NAME	ZOTU_0401	ZOTU_0402	ZOTU_0403	ZOTU_0404	ZOTU_0405	ZOTU_0406	ZOTU_0407	ZOTU_0408
CD_49	0	0	0	0	0	0	0	0
CD_51	0	0	0	0	0	0	0	0
CD_52	0	0	0	0	0	0	0	0
CD_65	0	0	0	0	0	0	0	0
CD_66	0	0	0	0	0	0	0	0
CD_67	0	0	0	0	0	0	0	0
CD_68	0	0	0	0	0	0	0	0
CD_78	0	0	0	0	0	0	0	0
CD_79	0	0	0	0	0	0	0	0
CDFG_53	0	0	0	0	0	0	0	0
CDFG_56	0	1	0	0	0	0	0	0
CDFG_69	0	0	0	1	0	0	1	0
CDFG_70	0	4	1	13	0	0	0	0
CDFG_71	0	0	0	0	0	0	0	0
HF_41	0	1	0	0	0	0	0	0
HF_42	0	0	0	0	0	0	0	0
HF_43	0	0	0	0	0	0	0	0
HF_57	0	0	0	0	0	0	0	0
HF_58	0	1	0	0	0	0	0	0
HF_72	0	0	0	0	0	0	0	0
HF_73	0	0	0	0	1	0	0	0
HF_74	0	0	0	0	0	0	0	0
HFFG_45	0	0	0	0	0	0	0	0
HFFG_48	0	0	3	0	0	0	0	0
HFFG_62	0	0	0	0	0	0	0	0
HFFG_77	0	0	0	0	0	0	0	0

Kingdom	Bacteria	Bacteria	Bacteria	Bacteria	Bacteria	Bacteria	Bacteria
Phylum	Bacteroidetes	Firmicutes	Actinobacteria	Proteobacteria	Cyanobacteria_Chloroplast	Bacteroidetes	Tenericutes
Class	Bacteroidia	Clostridia	Actinobacteria	Betaproteobacteria	Chloroplast	Bacteroidia	Mollicutes
Order	Bacteroidales	Clostridiales	Coriobacteriales	Neisseriales	Chloroplast	Bacteroidales	Acholeplasmatales
Family	Porphyromonadaceae	Lachnospiraceae	Coriobacteriaceae	Neisseriaceae	Streptophyta	Porphyromonadaceae	Acholeplasmataceae
Genus	Parabacteroides	Clostridium_XlVa	Atopobium	Neisseria	NA	Parabacteroides	Acholeplasma
#NAME	ZOTU_0409	ZOTU_0410	ZOTU_0411	ZOTU_0412	ZOTU_0413	ZOTU_0414	ZOTU_0415
CD_49	0	0	0	0	0	0	0
CD_51	0	0	0	0	0	0	0
CD_52	0	0	0	0	0	0	0
CD_65	0	0	0	0	0	0	0
CD_66	0	0	0	0	0	0	0
CD_67	0	0	0	0	0	2	0
CD_68	0	0	0	0	0	1	0
CD_78	0	0	0	0	0	0	0
CD_79	0	0	0	0	0	0	0
CDFG_53	0	0	0	0	0	0	0
CDFG_56	0	0	0	0	0	0	1
CDFG_69	0	0	0	0	0	0	0
CDFG_70	0	0	0	0	0	0	0
CDFG_71	0	0	0	0	0	0	0
HF_41	0	0	0	0	0	0	0
HF_42	0	0	0	0	0	0	0
HF_43	0	0	0	0	0	0	0
HF_57	0	0	0	0	0	0	0
HF_58	0	0	0	0	0	0	0
HF_72	0	0	0	0	0	0	0
HF_73	0	0	0	0	0	0	0
HF_74	0	0	0	0	0	0	0
HFFG_45	0	0	0	0	4	0	0
HFFG_48	0	0	0	0	0	0	0
HFFG_62	0	0	1	0	3	0	0
HFFG_77	0	0	0	1	0	0	0

Kingdom	Bacteria	Bacteria	Bacteria	Bacteria	Bacteria	Bacteria	Bacteria	Bacteria
Phylum	Firmicutes	Firmicutes	Firmicutes	Cyanobacteria_Chloroplast	Firmicutes	Actinobacteria	Firmicutes	Firmicutes
Class	Clostridia	Clostridia	Clostridia	Chloroplast	Clostridia	Actinobacteria	Bacilli	Bacilli
Order	Clostridiales	Clostridiales	Clostridiales	Chloroplast	Clostridiales	Coriobacteriales	Bacillales	Bacillales
Family	Clostridiaceae_	Lachnospiraceae	Clostridiaceae_	Streptophyta	Lachnospiraceae	Coriobacteriaceae	Staphylococcaceae	Bacillaceae_
Genus	Clostridium_sensu_stricto	Clostridium_XlVa	Clostridium_sensu_stricto	NA	Butyrivibrio	Enterorhabdus	Staphylococcus	Caldibacillus
#NAME	ZOTU_0416	ZOTU_0417	ZOTU_0418	ZOTU_0419	ZOTU_0420	ZOTU_0421	ZOTU_0422	ZOTU_0423
CD_49	0	0	0	0	0	2	10	0
CD_51	0	0	0	0	0	2	11	0
CD_52	0	0	0	0	0	2	16	0
CD_65	0	0	0	0	0	4	5	0
CD_66	0	0	0	0	0	10	5	0
CD_67	0	0	0	0	0	13	76	0
CD_68	0	0	0	0	0	4	157	0
CD_78	0	0	0	0	0	0	6	0
CD_79	0	0	0	0	0	0	0	0
CDFG_53	0	0	0	0	0	2	3	0
CDFG_56	0	0	0	0	0	0	0	0
CDFG_69	0	0	0	0	1	8	2	0
CDFG_70	0	0	0	0	1	11	5	0
CDFG_71	0	0	0	0	0	0	4	0
HF_41	0	0	0	0	0	0	1	0
HF_42	0	0	0	0	0	0	0	0
HF_43	0	0	0	0	0	0	0	0
HF_57	0	0	0	0	0	0	1	0
HF_58	0	0	0	0	0	5	8	0
HF_72	0	0	0	0	0	0	15	0
HF_73	0	0	0	0	0	0	129	0
HF_74	0	0	0	0	0	0	2	0
HFFG_45	0	0	0	2	0	0	0	0
HFFG_48	0	0	0	0	0	0	3	0
HFFG_62	0	0	0	2	0	1	4	9
HFFG_77	0	0	0	0	0	0	7	0

Kingdom	Bacteria	Bacteria	Bacteria	Bacteria	Bacteria	Bacteria	Bacteria	Bacteria
Phylum	Actinobacteria	Proteobacteria	Firmicutes	Firmicutes	Firmicutes	Firmicutes	Actinobacteria	Firmicutes
Class	Actinobacteria	Gammaproteobacteria	Bacilli	Bacilli	Bacilli	Clostridia	Actinobacteria	Bacilli
Order	Actinomycetales	Pasteurellales	Lactobacillales	Bacillales	Bacillales	Clostridiales	Actinomycetales	Bacillales
Family	Corynebacteriaceae	Pasteurellaceae	Lactobacillaceae	Bacillaceae_	Bacillales_Incertae_Sedis_XI	Clostridiaceae_	Actinomycetaceae	Staphylococcaceae
Genus	Corynebacterium	Actinobacillus	Lactobacillus	Bacillus	Gemella	Clostridium_sensu_stricto	Actinomyces	Staphylococcus
#NAME	ZOTU_0424	ZOTU_0425	ZOTU_0426	ZOTU_0427	ZOTU_0428	ZOTU_0429	ZOTU_0430	ZOTU_0431
CD_49	0	0	0	0	0	0	0	16
CD_51	0	0	0	0	0	0	0	5
CD_52	0	0	0	0	0	0	0	127
CD_65	0	0	0	0	0	0	0	4
CD_66	0	0	0	0	0	0	0	5
CD_67	0	0	0	0	0	0	0	17
CD_68	0	0	0	0	0	0	0	0
CD_78	0	0	0	0	0	0	0	3
CD_79	0	0	0	0	0	0	0	0
CDFG_53	0	0	0	0	0	0	0	91
CDFG_56	0	0	0	0	0	0	0	2
CDFG_69	0	0	0	0	0	0	0	2
CDFG_70	0	0	0	0	0	0	0	4
CDFG_71	0	0	0	3	0	0	0	2
HF_41	0	0	0	0	0	0	1	4
HF_42	0	0	0	0	0	0	0	0
HF_43	0	0	0	0	2	0	0	8
HF_57	0	0	0	0	0	0	0	0
HF_58	0	0	0	1	0	0	0	23
HF_72	0	0	0	1	0	0	0	13
HF_73	0	0	0	1	0	0	0	184
HF_74	0	0	0	0	0	0	0	67
HFFG_45	1	0	0	0	0	0	3	0
HFFG_48	1	0	0	0	1	0	0	1
HFFG_62	0	1	0	1	0	0	0	10
HFFG_77	0	0	0	0	0	0	1	20

Kingdom	Bacteria	Bacteria	Bacteria	Bacteria	Bacteria	Bacteria	Bacteria	Bacteria
Phylum	Proteobacteria	Firmicutes	Firmicutes	Firmicutes	Actinobacteria	Firmicutes	Firmicutes	Bacteroidetes
Class	Alphaproteobacteria	Erysipelotrichia	Clostridia	Clostridia	Actinobacteria	Clostridia	Clostridia	Bacteroidia
Order	Rhizobiales	Erysipelotrichales	Clostridiales	Clostridiales	Coriobacteriales	Clostridiales	Clostridiales	Bacteroidales
Family	Bradyrhizobiaceae	Erysipelotrichaceae	Clostridiaceae_	Lachnospiraceae	Coriobacteriaceae	Lachnospiraceae	Lachnospiraceae	Porphyromonadaceae
Genus	Bradyrhizobium	Erysipelotrichaceae_incertae_sedis	Alkaliphilus	Clostridium_XlVa	Enterorhabdus	Clostridium_XlVa	Clostridium_XlVa	Parabacteroides
#NAME	ZOTU_0432	ZOTU_0433	ZOTU_0434	ZOTU_0435	ZOTU_0436	ZOTU_0437	ZOTU_0438	ZOTU_0439
CD_49	0	0	0	0	0	0	0	0
CD_51	0	0	0	0	0	0	0	0
CD_52	0	0	0	0	0	0	0	0
CD_65	0	0	0	0	0	0	0	0
CD_66	0	0	0	0	0	0	0	0
CD_67	0	0	0	0	0	0	0	0
CD_68	0	0	0	0	0	0	0	0
CD_78	0	0	0	0	0	0	0	0
CD_79	0	0	0	0	0	0	0	0
CDFG_53	0	0	0	0	0	0	0	0
CDFG_56	0	0	0	0	0	0	0	0
CDFG_69	0	0	0	0	0	0	0	0
CDFG_70	0	0	0	0	0	0	0	0
CDFG_71	0	0	0	0	0	0	0	0
HF_41	0	0	0	0	0	0	0	0
HF_42	0	0	0	0	0	0	0	0
HF_43	0	0	0	0	0	0	0	0
HF_57	0	0	0	0	0	0	0	0
HF_58	0	0	0	0	4	0	0	0
HF_72	0	0	0	0	0	1	0	0
HF_73	0	0	0	0	0	0	0	0
HF_74	0	0	0	0	0	0	0	0
HFFG_45	0	0	0	0	0	0	0	1
HFFG_48	0	0	0	0	0	0	0	0
HFFG_62	1	0	0	0	1	0	0	0
HFFG_77	0	0	0	0	0	4	0	0

Kingdom	Bacteria	Bacteria	Bacteria	Bacteria	Bacteria	Bacteria	Bacteria	Bacteria
Phylum	Firmicutes	Firmicutes	Firmicutes	Firmicutes	Firmicutes	Actinobacteria	Firmicutes	Actinobacteria
Class	Clostridia	Clostridia	Bacilli	Clostridia	Bacilli	Actinobacteria	Clostridia	Actinobacteria
Order	Clostridiales	Clostridiales	Lactobacillales	Clostridiales	Lactobacillales	Coriobacteriales	Clostridiales	Actinomycetales
Family	Lachnospiraceae	Peptostreptococcaceae	Enterococcaceae	Clostridiales_Incertae_Sedis_XII	Aerococcaceae	Coriobacteriaceae	Lachnospiraceae	Micrococcaceae
Genus	Clostridium_XlVa	Romboutsia	Enterococcus	Guggenheimella	Abiotrophia	Senegalimassilia	Blautia	Micrococcus
#NAME	ZOTU_0440	ZOTU_0441	ZOTU_0442	ZOTU_0443	ZOTU_0444	ZOTU_0445	ZOTU_0446	ZOTU_0447
CD_49	0	2	0	0	0	0	0	0
CD_51	0	0	0	0	0	5	0	0
CD_52	0	0	0	0	0	0	0	0
CD_65	2	0	0	0	0	0	0	0
CD_66	1	0	0	0	0	1	0	0
CD_67	0	0	4	0	0	1	0	0
CD_68	0	0	6	0	0	0	0	0
CD_78	0	0	1	0	0	2	0	0
CD_79	0	0	0	0	0	0	0	0
CDFG_53	0	3	0	0	0	10	0	0
CDFG_56	0	0	1	0	0	9	0	0
CDFG_69	0	0	2	0	0	17	0	0
CDFG_70	2	1	4	0	0	1	0	0
CDFG_71	0	0	0	0	0	9	0	0
HF_41	0	0	0	0	0	0	0	0
HF_42	0	0	1	0	0	0	0	0
HF_43	3	0	0	0	0	0	0	0
HF_57	0	0	0	0	0	0	0	0
HF_58	0	0	0	0	0	2	0	0
HF_72	0	0	0	0	0	0	0	0
HF_73	0	0	0	0	0	2	0	0
HF_74	0	0	0	0	0	0	0	0
HFFG_45	0	1	0	0	0	0	0	1
HFFG_48	0	1	1	0	0	1	0	0
HFFG_62	1	0	2	0	0	0	0	5
HFFG_77	0	0	7	0	0	0	0	0

Kingdom	Bacteria	Bacteria	Bacteria	Bacteria	Bacteria	Bacteria	Bacteria	Bacteria
Phylum	Firmicutes	Actinobacteria	Proteobacteria	Firmicutes	Actinobacteria	Actinobacteria	Firmicutes	Firmicutes
Class	Clostridia	Actinobacteria	Gammaproteobacteria	Clostridia	Actinobacteria	Actinobacteria	Clostridia	Bacilli
Order	Clostridiales	Coriobacteriales	Legionellales	Clostridiales	Actinomycetales	Coriobacteriales	Clostridiales	Bacillales
Family	Lachnospiraceae	Coriobacteriaceae	Legionellaceae	Lachnospiraceae	Micrococcaceae	Coriobacteriaceae	Peptostreptococcaceae	Paenibacillaceae_
Genus	Clostridium_XlVa	Enterorhabdus	Legionella	Clostridium_XlVa	Rothia	Enterorhabdus	Clostridium_XI	Paenibacillus
#NAME	ZOTU_0448	ZOTU_0449	ZOTU_0450	ZOTU_0451	ZOTU_0452	ZOTU_0453	ZOTU_0454	ZOTU_0455
CD_49	0	0	0	0	0	4	0	0
CD_51	0	0	0	0	0	2	0	0
CD_52	0	0	0	0	0	5	0	0
CD_65	0	0	0	0	0	7	0	0
CD_66	0	0	0	0	0	22	0	0
CD_67	0	0	0	0	0	33	0	0
CD_68	0	0	0	0	0	7	0	0
CD_78	0	0	0	0	0	0	0	0
CD_79	0	0	0	0	0	0	0	0
CDFG_53	0	0	0	0	0	11	0	1
CDFG_56	0	0	0	0	0	0	0	3
CDFG_69	0	0	0	0	0	28	0	0
CDFG_70	0	0	0	0	1	17	0	1
CDFG_71	0	0	0	0	0	1	0	0
HF_41	0	0	0	0	0	0	0	0
HF_42	0	0	0	0	0	0	0	0
HF_43	0	0	0	0	0	0	0	0
HF_57	0	0	0	0	0	0	0	0
HF_58	0	0	0	0	1	2	0	0
HF_72	0	0	0	0	0	0	0	0
HF_73	0	0	0	0	0	1	0	0
HF_74	0	0	0	0	0	1	0	0
HFFG_45	0	0	0	0	0	0	0	0
HFFG_48	0	0	0	0	1	0	0	1
HFFG_62	0	0	0	0	1	0	0	0
HFFG_77	0	0	0	0	0	0	0	0

Kingdom	Bacteria	Bacteria	Bacteria	Bacteria	Bacteria
Phylum	Firmicutes	Firmicutes	Firmicutes	Firmicutes	Firmicutes
Class	Bacilli	Bacilli	Clostridia	Bacilli	Erysipelotrichia
Order	Bacillales	Bacillales	Clostridiales	Bacillales	Erysipelotrichales
Family	Paenibacillaceae_	Bacillaceae_	Clostridiaceae_	Paenibacillaceae_	Erysipelotrichaceae
Genus	Paenibacillus	Virgibacillus	Alkaliphilus	Paenibacillus	Kandleria
#NAME	ZOTU_0456	ZOTU_0457	ZOTU_0458	ZOTU_0459	ZOTU_0460
CD_49	0	0	0	0	0
CD_51	0	0	0	0	0
CD_52	0	0	0	0	0
CD_65	0	0	0	0	0
CD_66	0	0	0	0	0
CD_67	0	0	0	0	0
CD_68	0	0	0	0	0
CD_78	0	0	0	0	0
CD_79	0	0	0	0	0
CDFG_53	0	0	0	0	0
CDFG_56	0	0	0	0	0
CDFG_69	0	0	0	0	0
CDFG_70	0	0	0	0	0
CDFG_71	0	0	0	0	0
HF_41	0	0	0	0	0
HF_42	0	0	0	0	0
HF_43	0	0	0	0	0
HF_57	0	0	0	0	0
HF_58	0	0	0	0	0
HF_72	0	0	0	0	0
HF_73	0	0	0	0	0
HF_74	0	0	0	0	0
HFFG_45	0	0	0	0	0
HFFG_48	0	0	0	0	0
HFFG_62	0	0	0	0	0
HFFG_77	0	0	0	0	0

Table S13 (pages S-125 to S-234). Raw OTU count data from 16S sequencing of cecum contents.

Kingdom	Bacteria	Bacteria	Bacteria	Bacteria	Bacteria	Bacteria	Bacteria	Bacteria	Bacteria
Phylum	Firmicutes	Verrucomicrobia	Firmicutes	Firmicutes	Firmicutes	Firmicutes	Actinobacteria	Firmicutes	Firmicutes
Class	Erysipelotrichia	Verrucomicrobiae	Bacilli	Erysipelotrichia	Bacilli	Bacilli	Actinobacteria	Bacilli	Bacilli
Order	Erysipelotrichales	Verrucomicrobiales	Lactobacillales	Erysipelotrichales	Bacillales	Lactobacillales	Bifidobacteriales	Bacillales	Bacillales
Family	Erysipelotrichaceae	Verrucomicrobiaceae	Enterococcaceae	Erysipelotrichaceae	Staphylococcaceae	Lactobacillaceae	Bifidobacteriaceae	Bacillaceae_	Staphylococcaceae
Genus	Allobaculum	Akkermansia	Enterococcus	Catenisphaera	Staphylococcus	Lactobacillus	Bifidobacterium	Bacillus	Staphylococcus
#NAME	ZOTU_0001	ZOTU_0002	ZOTU_0003	ZOTU_0004	ZOTU_0005	ZOTU_0006	ZOTU_0007	ZOTU_0008	ZOTU_0009
CD_49	8254	4492	2030	6707	25	823	1695	3	1
CD_50	18976	6669	3090	5539	8	90	2950	2	1
CD_51	18695	16457	10434	542	687	226	2214	5	0
CD_52	18900	3999	4087	4489	3	239	2286	2	1
CD_65	3519	1671	1174	2575	3	138	778	8	0
CD_66	2548	727	787	1683	6	75	597	5	0
CD_67	3610	1169	196	2450	98	131	710	4	0
CD_68	4346	1434	2427	3390	10	94	782	1	0
CD_78	12080	6881	3126	2928	43	1	1468	11	1
CD_79	1875	1567	466	301	1	4	105	1575	0
CD_80	3778	1211	615	492	1	2	360	6	0
CDFG_54	4472	5530	2136	384	7	15	683	3	1
CDFG_55	12682	9314	3563	733	32	75	2162	1402	16
CDFG_56	14547	14605	6588	1620	183	146	1873	1388	23
CDFG_70	2744	1996	1918	23	6	10	315	4	0
CDFG_71	4741	888	1275	821	3	7	885	4	0
HF_41	2355	738	2295	2892	17	338	52	7	0
HF_42	269	2030	1500	102	241	103	5	1	0
HF_43	7835	20	2311	6203	106	1206	3	9	5
HF_44	8411	490	6213	11152	15	4686	13	499	11
HF_57	2906	2039	3110	1775	113	304	133	5	0
HF_58	3460	10474	12902	4067	152	507	37	89	0
HF_59	1902	6115	3416	1372	0	242	9	34444	0
HF_60	1594	5279	5153	679	3	168	3	83	0
HF_73	4513	236	2567	4510	93	2871	167	41	0
HF_74	2736	95	2800	2764	9	1408	42	1	0
HF_75	1936	3356	71	450	1	995	5	6	0

Kingdom	Bacteria	Bacteria	Bacteria	Bacteria	Bacteria	Bacteria	Bacteria	Bacteria	Bacteria
Phylum	Firmicutes	Verrucomicrobia	Firmicutes	Firmicutes	Firmicutes	Firmicutes	Actinobacteria	Firmicutes	Firmicutes
Class	Erysipelotrichia	Verrucomicrobiae	Bacilli	Erysipelotrichia	Bacilli	Bacilli	Actinobacteria	Bacilli	Bacilli
Order	Erysipelotrichales	Verrucomicrobiales	Lactobacillales	Erysipelotrichales	Bacillales	Lactobacillales	Bifidobacteriales	Bacillales	Bacillales
Family	Erysipelotrichaceae	Verrucomicrobiaceae	Enterococcaceae	Erysipelotrichaceae	Staphylococcaceae	Lactobacillaceae	Bifidobacteriaceae	Bacillaceae_	Staphylococcaceae
Genus	Allobaculum	Akkermansia	Enterococcus	Catenisphaera	Staphylococcus	Lactobacillus	Bifidobacterium	Bacillus	Staphylococcus
#NAME	ZOTU_0001	ZOTU_0002	ZOTU_0003	ZOTU_0004	ZOTU_0005	ZOTU_0006	ZOTU_0007	ZOTU_0008	ZOTU_0009
HFFG_45	4330	5330	5267	3000	3	966	136	119	114
HFFG_46	1223	1186	92	339	0	344	7	929	2417
HFFG_47	997	1391	434	1072	2	99	47	14	2093
HFFG_61	2316	352	1610	968	13	453	20	1	0
HFFG_62	12447	4561	8448	5554	30	1562	68	6	1
HFFG_63	2170	1712	789	684	11	685	45	3	2
HFFG_64	1370	1483	1404	1401	3	611	46	5	0
HFFG_76	7223	13400	9148	2163	36	2022	5	2	0

Kingdom	Bacteria	Bacteria	Bacteria	Bacteria	Bacteria	Bacteria	Bacteria	Bacteria
Phylum	Firmicutes	Firmicutes	Bacteroidetes	Firmicutes	Firmicutes	Actinobacteria	Firmicutes	Firmicutes
Class	Clostridia	Bacilli	Bacteroidia	Clostridia	Clostridia	Actinobacteria	Bacilli	Clostridia
Order	Clostridiales	Lactobacillales	Bacteroidales	Clostridiales	Clostridiales	Coriobacteriales	Bacillales	Clostridiales
Family	Peptostreptococcaceae	Streptococcaceae	Bacteroidaceae	Lachnospiraceae	Lachnospiraceae	Coriobacteriaceae	Staphylococcaceae	Lachnospiraceae
Genus	Romboutsia	Lactococcus	Bacteroides	Clostridium_XlVa	Acetatifactor	Enterorhabdus	Staphylococcus	Clostridium_XlVa
#NAME	ZOTU_0010	ZOTU_0011	ZOTU_0012	ZOTU_0013	ZOTU_0014	ZOTU_0015	ZOTU_0016	ZOTU_0017
CD_49	119	14	992	17	606	146	0	56
CD_50	15	35	302	10	414	457	0	0
CD_51	1360	88	1411	4	993	1368	0	121
CD_52	5	27	775	5	77	443	0	18
CD_65	6	7	45	172	113	33	0	0
CD_66	2	10	73	2	84	22	0	0
CD_67	13	2	210	0	97	12	0	0
CD_68	9	14	118	2	1117	19	0	1
CD_78	92	31	544	0	0	218	0	4
CD_79	58	12	78	0	0	19	0	0
CD_80	74	16	7	0	1	40	0	1
CDFG_54	288	11	33	10	3	122	0	256
CDFG_55	501	34	27	5	8	175	1	1028
CDFG_56	794	35	938	3	138	258	0	1858
CDFG_70	171	23	4	1	10	51	0	82
CDFG_71	49	8	4	1	23	25	0	79
HF_41	184	9	16	690	262	88	0	15
HF_42	5	8	5	670	79	207	0	3
HF_43	108	21	225	580	235	388	0	34
HF_44	317	25	10	909	287	447	1	53
HF_57	258	247	156	208	1363	183	0	25
HF_58	787	109	273	106	5293	434	0	188
HF_59	454	36	51	65	5058	119	0	282
HF_60	1021	57	30	86	2240	203	0	43
HF_73	126	7	21	48	400	73	0	0
HF_74	212	10	22	387	735	73	0	4
HF_75	358	22	15	160	33	95	0	681

Kingdom	Bacteria	Bacteria	Bacteria	Bacteria	Bacteria	Bacteria	Bacteria	Bacteria
Phylum	Firmicutes	Firmicutes	Bacteroidetes	Firmicutes	Firmicutes	Actinobacteria	Firmicutes	Firmicutes
Class	Clostridia	Bacilli	Bacteroidia	Clostridia	Clostridia	Actinobacteria	Bacilli	Clostridia
Order	Clostridiales	Lactobacillales	Bacteroidales	Clostridiales	Clostridiales	Coriobacteriales	Bacillales	Clostridiales
Family	Peptostreptococcaceae	Streptococcaceae	Bacteroidaceae	Lachnospiraceae	Lachnospiraceae	Coriobacteriaceae	Staphylococcaceae	Lachnospiraceae
Genus	Romboutsia	Lactococcus	Bacteroides	Clostridium_XlVa	Acetatifactor	Enterorhabdus	Staphylococcus	Clostridium_XlVa
#NAME	ZOTU_0010	ZOTU_0011	ZOTU_0012	ZOTU_0013	ZOTU_0014	ZOTU_0015	ZOTU_0016	ZOTU_0017
HFFG_45	794	34	7	39	559	258	0	568
HFFG_46	121	8	3	4	6	58	12	312
HFFG_47	68	7	6	189	12	51	0	287
HFFG_61	43	1	0	46	27	16	0	123
HFFG_62	812	42	4	284	879	158	0	4219
HFFG_63	162	11	6	127	82	69	0	652
HFFG_64	361	11	7	2	92	25	0	643
HFFG_76	1100	45	86	1965	138	273	0	6652

Kingdom	Bacteria	Bacteria	Bacteria	Bacteria	Bacteria	Bacteria	Bacteria	Bacteria
Phylum	Firmicutes	Firmicutes	Bacteroidetes	Firmicutes	Firmicutes	Bacteroidetes	Proteobacteria	Firmicutes
Class	Clostridia	Clostridia	Bacteroidia	Erysipelotrichia	Clostridia	Bacteroidia	Betaproteobacteria	Clostridia
Order	Clostridiales	Clostridiales	Bacteroidales	Erysipelotrichales	Clostridiales	Bacteroidales	Burkholderiales	Clostridiales
Family	Clostridiaceae_	Peptostreptococcaceae	Porphyromonadaceae	Erysipelotrichaceae	Ruminococcaceae	Rikenellaceae	Sutterellaceae	Lachnospiraceae
Genus	Clostridium_sensu_stricto	Romboutsia	Barnesiella	Allobaculum	Pseudoflavonifractor	Alistipes	Parasutterella	Clostridium_XlVa
#NAME	ZOTU_0018	ZOTU_0019	ZOTU_0020	ZOTU_0021	ZOTU_0022	ZOTU_0023	ZOTU_0024	ZOTU_0025
CD_49	52	40	779	0	177	404	442	71
CD_50	13	4	580	0	377	145	150	28
CD_51	5834	362	2575	0	835	174	285	61
CD_52	9	1	182	0	170	358	166	86
CD_65	3	1	77	0	130	61	20	8
CD_66	0	1	14	0	38	42	55	28
CD_67	3	9	89	0	14	139	32	0
CD_68	20	2	28	0	165	59	134	0
CD_78	212	31	227	0	6	230	73	0
CD_79	50	12	29	0	5	27	20	2
CD_80	93	18	4	0	1	29	1	0
CDFG_54	297	145	7	0	165	46	5	3
CDFG_55	1019	230	114	0	366	211	16	66
CDFG_56	1095	425	21	3	265	148	17	153
CDFG_70	1	43	84	0	18	126	2	12
CDFG_71	7	13	28	0	19	68	2	5
HF_41	13	98	18	0	23	6	7	30
HF_42	5	0	1	0	13	4	2	60
HF_43	3	51	1	0	38	0	5	32
HF_44	40	165	4	0	110	2	2	162
HF_57	6	102	36	1	133	37	112	284
HF_58	21	348	17	5	1166	28	281	495
HF_59	9	194	7	0	376	19	92	281
HF_60	61	432	1	0	376	3	91	336
HF_73	2	64	13	2	49	9	25	175
HF_74	1	117	15	0	92	5	48	169
HF_75	80	139	102	1	261	9	5	211

Kingdom	Bacteria	Bacteria	Bacteria	Bacteria	Bacteria	Bacteria	Bacteria	Bacteria
Phylum	Firmicutes	Firmicutes	Bacteroidetes	Firmicutes	Firmicutes	Bacteroidetes	Proteobacteria	Firmicutes
Class	Clostridia	Clostridia	Bacteroidia	Erysipelotrichia	Clostridia	Bacteroidia	Betaproteobacteria	Clostridia
Order	Clostridiales	Clostridiales	Bacteroidales	Erysipelotrichales	Clostridiales	Bacteroidales	Burkholderiales	Clostridiales
Family	Clostridiaceae_	Peptostreptococcaceae	Porphyromonadaceae	Erysipelotrichaceae	Ruminococcaceae	Rikenellaceae	Sutterellaceae	Lachnospiraceae
Genus	Clostridium_sensu_stricto	Romboutsia	Barnesiella	Allobaculum	Pseudoflavonifractor	Alistipes	Parasutterella	Clostridium_XlVa
#NAME	ZOTU_0018	ZOTU_0019	ZOTU_0020	ZOTU_0021	ZOTU_0022	ZOTU_0023	ZOTU_0024	ZOTU_0025
HFFG_45	638	348	164	0	314	6	9	6
HFFG_46	26	46	21	0	60	7	1	73
HFFG_47	31	26	38	0	40	6	1	28
HFFG_61	6	22	17	1	67	0	0	201
HFFG_62	54	347	106	0	600	24	9	1354
HFFG_63	14	79	65	0	171	6	3	72
HFFG_64	25	156	13	0	57	22	13	94
HFFG_76	189	488	265	2	959	17	19	438

Kingdom	Bacteria	Bacteria	Bacteria	Bacteria	Bacteria	Bacteria	Bacteria	Bacteria
Phylum	Actinobacteria	Firmicutes	Bacteroidetes	Firmicutes	Firmicutes	Firmicutes	Bacteroidetes	Firmicutes
Class	Actinobacteria	Clostridia	Bacteroidia	Clostridia	Bacilli	Erysipelotrichia	Bacteroidia	Clostridia
Order	Coriobacteriales	Clostridiales	Bacteroidales	Clostridiales	Bacillales	Erysipelotrichales	Bacteroidales	Clostridiales
Family	Coriobacteriaceae	Eubacteriaceae	Porphyromonadaceae	Ruminococcaceae	Planococcaceae	Erysipelotrichaceae	Porphyromonadaceae	Lachnospiraceae
Genus	Enterorhabdus	Eubacterium	Parabacteroides	Pseudoflavonifractor	Lysinibacillus	Turicibacter	Barnesiella	Clostridium_XlVa
#NAME	ZOTU_0026	ZOTU_0027	ZOTU_0028	ZOTU_0029	ZOTU_0030	ZOTU_0031	ZOTU_0032	ZOTU_0033
CD_49	121	0	0	9	0	0	1	47
CD_50	18	0	0	4	0	0	1	443
CD_51	50	0	0	32	0	0	2	368
CD_52	54	0	0	5	0	0	2	364
CD_65	9	0	1	6	0	0	4	124
CD_66	5	0	21	2	0	0	0	5
CD_67	11	0	0	0	0	0	0	8
CD_68	20	0	46	4	0	0	1	12
CD_78	14	1	0	0	0	0	0	8
CD_79	1	0	0	1	0	1	0	30
CD_80	4	1	0	1	0	0	0	22
CDFG_54	9	20	1	7	0	0	92	25
CDFG_55	34	0	1	37	0	1	154	76
CDFG_56	47	4	1	28	0	3	101	50
CDFG_70	7	1	0	5	0	266	0	13
CDFG_71	10	1	0	1	0	28	1	61
HF_41	14	1	0	301	0	0	40	136
HF_42	3	0	0	688	0	0	36	205
HF_43	24	0	0	801	0	0	41	861
HF_44	28	0	0	920	0	1	291	603
HF_57	61	0	0	11	0	1	248	15
HF_58	175	0	0	31	0	0	2189	4
HF_59	29	0	0	14	0	0	1114	6
HF_60	22	0	0	12	0	0	525	3
HF_73	73	0	0	9	0	1	156	70
HF_74	93	0	0	14	0	0	497	86
HF_75	2	0	0	9	0	77	1	3

Kingdom	Bacteria	Bacteria	Bacteria	Bacteria	Bacteria	Bacteria	Bacteria	Bacteria
Phylum	Actinobacteria	Firmicutes	Bacteroidetes	Firmicutes	Firmicutes	Firmicutes	Bacteroidetes	Firmicutes
Class	Actinobacteria	Clostridia	Bacteroidia	Clostridia	Bacilli	Erysipelotrichia	Bacteroidia	Clostridia
Order	Coriobacteriales	Clostridiales	Bacteroidales	Clostridiales	Bacillales	Erysipelotrichales	Bacteroidales	Clostridiales
Family	Coriobacteriaceae	Eubacteriaceae	Porphyromonadaceae	Ruminococcaceae	Planococcaceae	Erysipelotrichaceae	Porphyromonadaceae	Lachnospiraceae
Genus	Enterorhabdus	Eubacterium	Parabacteroides	Pseudoflavonifractor	Lysinibacillus	Turicibacter	Barnesiella	Clostridium_XlVa
#NAME	ZOTU_0026	ZOTU_0027	ZOTU_0028	ZOTU_0029	ZOTU_0030	ZOTU_0031	ZOTU_0032	ZOTU_0033
HFFG_45	16	4	1	21	0	5	0	29
HFFG_46	5	34	0	8	0	15	3	4
HFFG_47	3	1	0	4	0	3	0	2
HFFG_61	4	0	8	4	0	1	0	8
HFFG_62	20	0	54	45	0	30	1	173
HFFG_63	11	0	23	15	0	8	0	13
HFFG_64	4	0	16	5	0	37	3	10
HFFG_76	19	0	0	72	0	60	0	16

Kingdom	Bacteria	Bacteria	Bacteria	Bacteria	Bacteria	Bacteria	Bacteria	Bacteria	Bacteria
Phylum	Firmicutes	Actinobacteria	Bacteroidetes	Firmicutes	Firmicutes	Firmicutes	Actinobacteria	Firmicutes	Firmicutes
Class	Erysipelotrichia	Actinobacteria	Bacteroidia	Clostridia	Clostridia	Clostridia	Actinobacteria	Clostridia	Clostridia
Order	Erysipelotrichales	Coriobacteriales	Bacteroidales	Clostridiales	Clostridiales	Clostridiales	Coriobacteriales	Clostridiales	Clostridiales
Family	Erysipelotrichaceae	Coriobacteriaceae	Porphyromonadaceae	Clostridiaceae_	Lachnospiraceae	Lachnospiraceae	Coriobacteriaceae	Lachnospiraceae	Lachnospiraceae
Genus	Clostridium_XVIII	Parvibacter	Barnesiella	Alkaliphilus	Clostridium_XlVa	Clostridium_XlVa	Senegalimassilia	Clostridium_XlVa	Clostridium_XlVa
#NAME	ZOTU_0034	ZOTU_0035	ZOTU_0036	ZOTU_0037	ZOTU_0038	ZOTU_0039	ZOTU_0040	ZOTU_0041	ZOTU_0042
CD_49	60	11	456	0	10	1	3	62	123
CD_50	26	84	127	0	19	5	2	89	54
CD_51	85	56	430	5	96	0	130	37	304
CD_52	25	43	149	0	5	0	10	11	1
CD_65	21	0	25	0	7	1	2	15	113
CD_66	9	2	29	0	5	1	0	8	21
CD_67	4	2	45	0	1	3	0	15	16
CD_68	15	0	24	0	15	0	0	43	69
CD_78	130	20	38	0	1	0	7	1	5
CD_79	41	0	6	0	1	1	4	0	10
CD_80	22	4	1	1	0	0	0	1	1
CDFG_54	30	56	3	0	40	1	8	147	2
CDFG_55	54	96	45	0	37	2	18	285	14
CDFG_56	101	132	19	0	36	18	24	191	3
CDFG_70	48	60	4	0	7	0	14	14	0
CDFG_71	17	19	4	0	3	1	22	27	5
HF_41	14	20	7	0	15	0	5	13	2
HF_42	3	13	0	0	39	6	21	19	0
HF_43	18	77	4	0	44	7	35	37	0
HF_44	31	107	3	0	84	0	101	65	0
HF_57	105	22	32	0	122	4	8	83	254
HF_58	326	59	123	0	229	11	25	331	1016
HF_59	98	20	106	0	241	8	9	182	406
HF_60	105	29	7	0	88	1	7	77	108
HF_73	11	17	8	0	109	0	30	122	299
HF_74	20	9	13	0	115	2	21	37	242
HF_75	114	0	27	0	10	3	7	102	54

Kingdom	Bacteria	Bacteria	Bacteria	Bacteria	Bacteria	Bacteria	Bacteria	Bacteria	Bacteria
Phylum	Firmicutes	Actinobacteria	Bacteroidetes	Firmicutes	Firmicutes	Firmicutes	Actinobacteria	Firmicutes	Firmicutes
Class	Erysipelotrichia	Actinobacteria	Bacteroidia	Clostridia	Clostridia	Clostridia	Actinobacteria	Clostridia	Clostridia
Order	Erysipelotrichales	Coriobacteriales	Bacteroidales	Clostridiales	Clostridiales	Clostridiales	Coriobacteriales	Clostridiales	Clostridiales
Family	Erysipelotrichaceae	Coriobacteriaceae	Porphyromonadaceae	Clostridiaceae_	Lachnospiraceae	Lachnospiraceae	Coriobacteriaceae	Lachnospiraceae	Lachnospiraceae
Genus	Clostridium_XVIII	Parvibacter	Barnesiella	Alkaliphilus	Clostridium_XlVa	Clostridium_XlVa	Senegalimassilia	Clostridium_XlVa	Clostridium_XlVa
#NAME	ZOTU_0034	ZOTU_0035	ZOTU_0036	ZOTU_0037	ZOTU_0038	ZOTU_0039	ZOTU_0040	ZOTU_0041	ZOTU_0042
HFFG_45	60	22	26	0	28	0	18	27	120
HFFG_46	8	1	8	0	2	0	0	23	47
HFFG_47	12	2	1	0	7	0	3	11	34
HFFG_61	5	3	9	0	4	0	0	20	34
HFFG_62	165	23	43	0	87	3	6	328	358
HFFG_63	41	10	12	0	27	0	0	94	73
HFFG_64	14	6	19	0	5	0	0	43	9
HFFG_76	235	0	93	0	47	4	9	282	295

Kingdom	Bacteria	Bacteria	Bacteria	Bacteria	Bacteria	Bacteria	Bacteria	Bacteria	Bacteria
Phylum	Firmicutes	Firmicutes	Actinobacteria	Firmicutes	Firmicutes	Firmicutes	Firmicutes	Bacteroidetes	Firmicutes
Class	Clostridia	Clostridia	Actinobacteria	Bacilli	Clostridia	Clostridia	Clostridia	Bacteroidia	Clostridia
Order	Clostridiales	Clostridiales	Coriobacteriales	Bacillales	Clostridiales	Clostridiales	Clostridiales	Bacteroidales	Clostridiales
Family	Lachnospiraceae	Lachnospiraceae	Coriobacteriaceae	Staphylococcaceae	Lachnospiraceae	Ruminococcaceae	Lachnospiraceae	Porphyromonadaceae	Clostridiaceae_
Genus	Clostridium_XlVa	Blautia	Asaccharobacter	Staphylococcus	Acetatifactor	Oscillibacter	Clostridium_XlVa	Parabacteroides	Alkaliphilus
#NAME	ZOTU_0043	ZOTU_0044	ZOTU_0045	ZOTU_0046	ZOTU_0047	ZOTU_0048	ZOTU_0049	ZOTU_0050	ZOTU_0051
CD_49	3	14	0	0	164	59	89	2	59
CD_50	5	11	0	0	67	11	60	0	43
CD_51	13	46	0	28	40	72	94	0	32
CD_52	1	22	0	0	19	8	34	0	26
CD_65	3	1	0	0	38	3	41	24	8
CD_66	0	1	0	0	9	7	10	43	1
CD_67	1	0	0	0	25	4	17	37	4
CD_68	0	3	1	0	56	13	54	70	0
CD_78	0	4	0	0	0	1	5	63	29
CD_79	0	0	0	0	3	0	9	3	2
CD_80	0	2	0	0	0	0	0	2	6
CDFG_54	101	72	0	0	26	19	83	0	26
CDFG_55	783	62	0	0	87	55	195	0	75
CDFG_56	103	123	0	0	90	15	33	0	14
CDFG_70	312	54	0	0	1	0	45	0	4
CDFG_71	180	25	0	0	9	6	10	0	6
HF_41	2	2	0	0	18	9	23	1	5
HF_42	2	11	0	0	2	43	49	0	10
HF_43	1	14	0	0	49	12	33	0	4
HF_44	12	27	0	0	14	68	42	0	81
HF_57	0	9	0	0	213	42	35	3	37
HF_58	3	65	6	0	220	399	68	2	112
HF_59	0	21	0	0	75	159	45	2	14
HF_60	9	23	1	0	6	160	45	0	10
HF_73	1	11	3	6	13	50	34	2	4
HF_74	2	9	1	0	62	167	38	2	1
HF_75	1	34	33	0	35	53	16	1	18

Kingdom	Bacteria	Bacteria	Bacteria	Bacteria	Bacteria	Bacteria	Bacteria	Bacteria	Bacteria
Phylum	Firmicutes	Firmicutes	Actinobacteria	Firmicutes	Firmicutes	Firmicutes	Firmicutes	Bacteroidetes	Firmicutes
Class	Clostridia	Clostridia	Actinobacteria	Bacilli	Clostridia	Clostridia	Clostridia	Bacteroidia	Clostridia
Order	Clostridiales	Clostridiales	Coriobacteriales	Bacillales	Clostridiales	Clostridiales	Clostridiales	Bacteroidales	Clostridiales
Family	Lachnospiraceae	Lachnospiraceae	Coriobacteriaceae	Staphylococcaceae	Lachnospiraceae	Ruminococcaceae	Lachnospiraceae	Porphyromonadaceae	Clostridiaceae_
Genus	Clostridium_XlVa	Blautia	Asaccharobacter	Staphylococcus	Acetatifactor	Oscillibacter	Clostridium_XlVa	Parabacteroides	Alkaliphilus
#NAME	ZOTU_0043	ZOTU_0044	ZOTU_0045	ZOTU_0046	ZOTU_0047	ZOTU_0048	ZOTU_0049	ZOTU_0050	ZOTU_0051
HFFG_45	237	46	4	0	48	72	124	2	37
HFFG_46	0	6	0	0	23	14	5	0	6
HFFG_47	0	4	0	0	7	5	9	0	19
HFFG_61	50	5	1	0	7	4	5	0	5
HFFG_62	2	31	5	0	252	119	26	0	6
HFFG_63	1	15	0	0	20	41	14	0	0
HFFG_64	2	7	3	0	79	10	1	0	6
HFFG_76	0	56	134	0	250	244	26	0	19

Kingdom	Bacteria	Bacteria	Bacteria	Bacteria	Bacteria	Bacteria	Bacteria	Bacteria	Bacteria
Phylum	Firmicutes	Firmicutes	Firmicutes	Bacteroidetes	Firmicutes	Firmicutes	Firmicutes	Firmicutes	Firmicutes
Class	Clostridia	Clostridia	Clostridia	Bacteroidia	Clostridia	Clostridia	Clostridia	Clostridia	Clostridia
Order	Clostridiales	Clostridiales	Clostridiales	Bacteroidales	Clostridiales	Clostridiales	Clostridiales	Clostridiales	Clostridiales
Family	Clostridiaceae_	Ruminococcaceae	Ruminococcaceae	Porphyromonadaceae	Lachnospiraceae	Lachnospiraceae	Ruminococcaceae	Lachnospiraceae	Clostridiaceae_
Genus	Alkaliphilus	Flavonifractor	Clostridium_IV	Barnesiella	Clostridium_XlVa	Acetatifactor	Oscillibacter	Ruminococcus	Alkaliphilus
#NAME	ZOTU_0052	ZOTU_0053	ZOTU_0054	ZOTU_0055	ZOTU_0056	ZOTU_0057	ZOTU_0058	ZOTU_0059	ZOTU_0060
CD_49	12	15	16	316	2	12	13	68	1
CD_50	1	6	29	161	27	5	1	1	0
CD_51	0	158	129	389	40	13	12	4	0
CD_52	0	18	17	224	0	0	5	7	0
CD_65	0	3	11	18	171	19	5	7	0
CD_66	0	1	8	37	158	2	11	9	0
CD_67	0	0	3	12	1	7	0	13	0
CD_68	0	6	8	52	339	20	8	43	0
CD_78	0	0	9	0	1	2	1	0	0
CD_79	0	0	6	0	0	1	0	0	0
CD_80	0	0	0	0	1	0	0	0	0
CDFG_54	0	4	52	13	0	1	13	1	0
CDFG_55	158	66	133	93	20	13	41	13	0
CDFG_56	139	16	102	38	1	11	19	15	0
CDFG_70	30	5	36	0	9	0	15	1	0
CDFG_71	11	6	25	0	0	0	6	0	0
HF_41	9	9	4	2	3	1	0	8	23
HF_42	0	9	14	1	2	2	0	2	180
HF_43	0	5	0	22	0	11	4	14	0
HF_44	0	24	30	37	3	18	7	12	2
HF_57	0	17	10	33	3	23	38	38	0
HF_58	0	78	31	407	2	21	211	229	0
HF_59	5	22	12	108	0	10	65	234	0
HF_60	2	47	39	36	0	8	62	31	0
HF_73	8	15	4	51	11	13	23	48	0
HF_74	57	99	4	71	4	62	35	44	0
HF_75	104	5	13	0	2	2	25	15	0

Kingdom	Bacteria	Bacteria	Bacteria	Bacteria	Bacteria	Bacteria	Bacteria	Bacteria	Bacteria
Phylum	Firmicutes	Firmicutes	Firmicutes	Bacteroidetes	Firmicutes	Firmicutes	Firmicutes	Firmicutes	Firmicutes
Class	Clostridia	Clostridia	Clostridia	Bacteroidia	Clostridia	Clostridia	Clostridia	Clostridia	Clostridia
Order	Clostridiales	Clostridiales	Clostridiales	Bacteroidales	Clostridiales	Clostridiales	Clostridiales	Clostridiales	Clostridiales
Family	Clostridiaceae_	Ruminococcaceae	Ruminococcaceae	Porphyromonadaceae	Lachnospiraceae	Lachnospiraceae	Ruminococcaceae	Lachnospiraceae	Clostridiaceae_
Genus	Alkaliphilus	Flavonifractor	Clostridium_IV	Barnesiella	Clostridium_XlVa	Acetatifactor	Oscillibacter	Ruminococcus	Alkaliphilus
#NAME	ZOTU_0052	ZOTU_0053	ZOTU_0054	ZOTU_0055	ZOTU_0056	ZOTU_0057	ZOTU_0058	ZOTU_0059	ZOTU_0060
HFFG_45	3	19	60	68	305	6	33	14	0
HFFG_46	45	1	6	7	0	6	4	8	0
HFFG_47	24	2	14	10	1	0	7	3	0
HFFG_61	5	6	2	0	0	1	19	18	0
HFFG_62	195	19	25	0	6	55	68	64	0
HFFG_63	8	1	18	0	1	25	39	11	0
HFFG_64	42	3	3	0	0	5	6	13	0
HFFG_76	254	43	43	0	5	31	86	57	0

Kingdom	Bacteria	Bacteria	Bacteria	Bacteria	Bacteria	Bacteria	Bacteria	Bacteria	Bacteria
Phylum	Firmicutes	Firmicutes	Bacteroidetes	Firmicutes	Firmicutes	Firmicutes	Actinobacteria	Firmicutes	Firmicutes
Class	Clostridia	Clostridia	Bacteroidia	Clostridia	Clostridia	Clostridia	Actinobacteria	Clostridia	Clostridia
Order	Clostridiales	Clostridiales	Bacteroidales	Clostridiales	Clostridiales	Clostridiales	Coriobacteriales	Clostridiales	Clostridiales
Family	Lachnospiraceae	Lachnospiraceae	Porphyromonadaceae	Lachnospiraceae	Ruminococcaceae	Lachnospiraceae	Coriobacteriaceae	Lachnospiraceae	Ruminococcaceae
Genus	Blautia	Clostridium_XlVa	Barnesiella	Acetatifactor	Clostridium_III	Acetatifactor	Paraeggerthella	Clostridium_XlVa	Ruminococcus
#NAME	ZOTU_0061	ZOTU_0062	ZOTU_0063	ZOTU_0064	ZOTU_0065	ZOTU_0066	ZOTU_0067	ZOTU_0068	ZOTU_0069
CD_49	0	4	269	6	27	24	4	9	7
CD_50	0	0	114	0	16	1	2	8	1
CD_51	1	0	348	4	37	8	48	41	13
CD_52	22	18	191	7	10	8	8	8	1
CD_65	0	0	17	2	4	5	2	0	0
CD_66	0	10	49	1	3	2	0	0	2
CD_67	0	1	18	3	1	0	1	0	1
CD_68	1	22	59	3	4	18	0	0	0
CD_78	0	0	0	0	4	0	1	0	0
CD_79	0	0	0	0	2	0	0	0	0
CD_80	0	0	0	1	2	1	0	0	0
CDFG_54	3	10	25	8	1	0	5	9	12
CDFG_55	24	35	73	24	49	23	16	6	52
CDFG_56	49	19	22	9	44	1	8	26	87
CDFG_70	11	24	0	3	0	1	4	4	8
CDFG_71	3	8	0	3	0	0	1	9	6
HF_41	2	0	12	2	4	3	1	1	3
HF_42	5	0	1	3	2	1	0	4	0
HF_43	4	0	10	8	0	17	2	0	9
HF_44	8	1	37	12	23	14	5	0	8
HF_57	4	1	28	0	3	42	0	30	6
HF_58	15	0	218	7	16	253	2	182	51
HF_59	24	0	74	8	8	63	1	78	22
HF_60	3	0	36	3	6	3	2	46	12
HF_73	2	12	34	8	6	4	1	35	5
HF_74	18	63	59	28	15	16	2	29	26
HF_75	6	19	0	17	3	6	4	17	4

Kingdom	Bacteria	Bacteria	Bacteria	Bacteria	Bacteria	Bacteria	Bacteria	Bacteria	Bacteria
Phylum	Firmicutes	Firmicutes	Bacteroidetes	Firmicutes	Firmicutes	Firmicutes	Actinobacteria	Firmicutes	Firmicutes
Class	Clostridia	Clostridia	Bacteroidia	Clostridia	Clostridia	Clostridia	Actinobacteria	Clostridia	Clostridia
Order	Clostridiales	Clostridiales	Bacteroidales	Clostridiales	Clostridiales	Clostridiales	Coriobacteriales	Clostridiales	Clostridiales
Family	Lachnospiraceae	Lachnospiraceae	Porphyromonadaceae	Lachnospiraceae	Ruminococcaceae	Lachnospiraceae	Coriobacteriaceae	Lachnospiraceae	Ruminococcaceae
Genus	Blautia	Clostridium_XlVa	Barnesiella	Acetatifactor	Clostridium_III	Acetatifactor	Paraeggerthella	Clostridium_XlVa	Ruminococcus
#NAME	ZOTU_0061	ZOTU_0062	ZOTU_0063	ZOTU_0064	ZOTU_0065	ZOTU_0066	ZOTU_0067	ZOTU_0068	ZOTU_0069
HFFG_45	10	26	51	24	12	21	5	1	31
HFFG_46	3	10	6	22	2	0	2	1	11
HFFG_47	1	7	5	2	4	5	0	0	0
HFFG_61	1	16	0	12	1	7	0	0	2
HFFG_62	3	122	0	126	10	30	7	0	14
HFFG_63	0	30	0	19	2	9	6	0	2
HFFG_64	0	5	0	15	2	8	1	0	1
HFFG_76	38	30	0	84	14	14	8	0	13

Kingdom	Bacteria	Bacteria	Bacteria	Bacteria	Bacteria	Bacteria	Bacteria	Bacteria	Bacteria
Phylum	Firmicutes	Firmicutes	Firmicutes	Firmicutes	Firmicutes	Firmicutes	Firmicutes	Firmicutes	Firmicutes
Class	Clostridia	Clostridia	Clostridia	Clostridia	Clostridia	Clostridia	Clostridia	Clostridia	Clostridia
Order	Clostridiales	Clostridiales	Clostridiales	Clostridiales	Clostridiales	Clostridiales	Clostridiales	Clostridiales	Clostridiales
Family	Ruminococcaceae	Lachnospiraceae	Lachnospiraceae	Lachnospiraceae	Catabacteriaceae	Lachnospiraceae	Ruminococcaceae	Ruminococcaceae	Lachnospiraceae
Genus	Clostridium_IV	Clostridium_XlVa	Clostridium_XlVa	Acetatifactor	Catabacter	Clostridium_XlVa	Clostridium_III	Sporobacter	Acetatifactor
#NAME	ZOTU_0070	ZOTU_0071	ZOTU_0072	ZOTU_0073	ZOTU_0074	ZOTU_0075	ZOTU_0076	ZOTU_0077	ZOTU_0078
CD_49	8	0	11	73	25	0	47	42	0
CD_50	4	0	6	29	23	0	26	1	0
CD_51	0	0	10	16	66	0	111	334	0
CD_52	1	0	15	18	13	0	14	15	0
CD_65	2	0	1	20	7	0	4	2	1
CD_66	1	0	0	2	5	4	7	3	2
CD_67	1	0	0	12	9	0	5	4	3
CD_68	2	0	0	18	15	1	11	1	18
CD_78	1	0	0	1	5	0	3	5	0
CD_79	0	0	0	3	1	0	1	1	0
CD_80	0	0	0	0	1	0	0	0	0
CDFG_54	11	0	0	4	13	0	7	0	0
CDFG_55	64	278	0	32	22	1	24	3	0
CDFG_56	38	35	2	31	32	0	28	5	0
CDFG_70	2	154	4	0	3	0	10	1	0
CDFG_71	0	11	0	3	1	0	5	0	1
HF_41	2	0	1	8	0	5	7	1	0
HF_42	1	0	0	2	8	0	11	4	1
HF_43	4	0	0	28	7	0	18	0	5
HF_44	4	1	2	3	9	0	35	1	3
HF_57	15	0	48	112	12	1	16	0	44
HF_58	34	0	95	108	30	0	74	7	95
HF_59	47	0	45	39	15	0	23	4	28
HF_60	2	0	61	4	39	0	49	1	10
HF_73	4	0	0	10	4	0	4	5	1
HF_74	30	12	2	19	16	0	17	1	13
HF_75	32	0	49	9	19	0	30	2	4

Kingdom	Bacteria	Bacteria	Bacteria	Bacteria	Bacteria	Bacteria	Bacteria	Bacteria	Bacteria
Phylum	Firmicutes	Firmicutes	Firmicutes	Firmicutes	Firmicutes	Firmicutes	Firmicutes	Firmicutes	Firmicutes
Class	Clostridia	Clostridia	Clostridia	Clostridia	Clostridia	Clostridia	Clostridia	Clostridia	Clostridia
Order	Clostridiales	Clostridiales	Clostridiales	Clostridiales	Clostridiales	Clostridiales	Clostridiales	Clostridiales	Clostridiales
Family	Ruminococcaceae	Lachnospiraceae	Lachnospiraceae	Lachnospiraceae	Catabacteriaceae	Lachnospiraceae	Ruminococcaceae	Ruminococcaceae	Lachnospiraceae
Genus	Clostridium_IV	Clostridium_XlVa	Clostridium_XlVa	Acetatifactor	Catabacter	Clostridium_XlVa	Clostridium_III	Sporobacter	Acetatifactor
#NAME	ZOTU_0070	ZOTU_0071	ZOTU_0072	ZOTU_0073	ZOTU_0074	ZOTU_0075	ZOTU_0076	ZOTU_0077	ZOTU_0078
HFFG_45	2	421	1	14	29	0	42	3	0
HFFG_46	7	14	0	7	2	0	1	0	0
HFFG_47	0	53	0	7	3	0	5	0	0
HFFG_61	2	0	2	1	2	0	0	0	1
HFFG_62	50	1	37	113	23	0	24	4	4
HFFG_63	10	0	1	6	11	0	3	2	2
HFFG_64	5	1	0	20	3	0	0	1	2
HFFG_76	52	0	69	108	42	1	37	12	33

Kingdom	Bacteria	Bacteria	Bacteria	Bacteria	Bacteria	Bacteria	Bacteria	Bacteria	Bacteria
Phylum	Firmicutes	Bacteroidetes	Firmicutes	Firmicutes	Firmicutes	Firmicutes	Firmicutes	Firmicutes	Firmicutes
Class	Clostridia	Bacteroidia	Clostridia	Clostridia	Clostridia	Clostridia	Clostridia	Clostridia	Clostridia
Order	Clostridiales	Bacteroidales	Clostridiales	Clostridiales	Clostridiales	Clostridiales	Clostridiales	Clostridiales	Clostridiales
Family	Lachnospiraceae	Porphyromonadaceae	Ruminococcaceae	Ruminococcaceae	Ruminococcaceae	Clostridiaceae_	Ruminococcaceae	Lachnospiraceae	Lachnospiraceae
Genus	Murimonas	Coprobacter	Oscillibacter	Clostridium_IV	Pseudoflavonifractor	Alkaliphilus	Clostridium_IV	Acetatifactor	Acetatifactor
#NAME	ZOTU_0079	ZOTU_0080	ZOTU_0081	ZOTU_0082	ZOTU_0083	ZOTU_0084	ZOTU_0085	ZOTU_0086	ZOTU_0087
CD_49	6	0	25	20	4	0	5	12	3
CD_50	0	0	0	16	15	0	16	4	0
CD_51	14	0	22	47	83	2	7	3	0
CD_52	1	0	4	10	4	0	0	1	0
CD_65	0	14	0	3	26	0	1	6	0
CD_66	0	37	1	3	17	0	0	2	0
CD_67	0	14	0	3	6	0	0	0	0
CD_68	1	42	3	21	54	0	6	15	0
CD_78	0	0	0	0	3	0	1	0	0
CD_79	0	0	0	0	0	0	0	0	0
CD_80	0	0	1	0	0	0	0	0	0
CDFG_54	11	0	7	4	7	0	5	0	5
CDFG_55	7	0	1	5	29	0	2	5	8
CDFG_56	9	7	3	7	6	1	3	3	1
CDFG_70	12	0	0	0	3	0	0	0	2
CDFG_71	7	1	1	0	1	54	0	0	0
HF_41	0	4	2	6	4	0	3	2	6
HF_42	0	0	11	7	5	0	1	0	2
HF_43	1	0	30	10	3	0	15	3	0
HF_44	10	1	15	8	36	0	8	1	0
HF_57	9	0	1	82	3	0	22	33	0
HF_58	52	1	188	96	51	0	42	145	1
HF_59	22	1	76	84	8	0	26	44	0
HF_60	9	0	69	40	15	0	9	9	0
HF_73	15	7	23	12	60	2	7	8	0
HF_74	6	23	34	52	211	9	17	1	0
HF_75	8	2	29	22	24	50	16	5	0

Kingdom	Bacteria	Bacteria	Bacteria	Bacteria	Bacteria	Bacteria	Bacteria	Bacteria	Bacteria
Phylum	Firmicutes	Bacteroidetes	Firmicutes	Firmicutes	Firmicutes	Firmicutes	Firmicutes	Firmicutes	Firmicutes
Class	Clostridia	Bacteroidia	Clostridia	Clostridia	Clostridia	Clostridia	Clostridia	Clostridia	Clostridia
Order	Clostridiales	Bacteroidales	Clostridiales	Clostridiales	Clostridiales	Clostridiales	Clostridiales	Clostridiales	Clostridiales
Family	Lachnospiraceae	Porphyromonadaceae	Ruminococcaceae	Ruminococcaceae	Ruminococcaceae	Clostridiaceae_	Ruminococcaceae	Lachnospiraceae	Lachnospiraceae
Genus	Murimonas	Coprobacter	Oscillibacter	Clostridium_IV	Pseudoflavonifractor	Alkaliphilus	Clostridium_IV	Acetatifactor	Acetatifactor
#NAME	ZOTU_0079	ZOTU_0080	ZOTU_0081	ZOTU_0082	ZOTU_0083	ZOTU_0084	ZOTU_0085	ZOTU_0086	ZOTU_0087
HFFG_45	36	7	31	11	17	54	20	15	1
HFFG_46	2	2	10	3	0	0	8	1	0
HFFG_47	4	5	6	4	4	14	6	2	0
HFFG_61	7	3	4	11	20	0	2	7	0
HFFG_62	33	21	0	107	48	0	36	27	1
HFFG_63	12	30	14	17	9	0	7	7	0
HFFG_64	7	3	4	6	2	0	4	7	0
HFFG_76	90	0	134	94	94	113	101	24	0

Kingdom	Bacteria	Bacteria	Bacteria	Bacteria	Bacteria	Bacteria	Bacteria	Bacteria	Bacteria
Phylum	Firmicutes	Firmicutes	Firmicutes	Bacteroidetes	Firmicutes	Firmicutes	Firmicutes	Firmicutes	Firmicutes
Class	Clostridia	Clostridia	Clostridia	Bacteroidia	Clostridia	Clostridia	Clostridia	Clostridia	Clostridia
Order	Clostridiales	Clostridiales	Clostridiales	Bacteroidales	Clostridiales	Clostridiales	Clostridiales	Clostridiales	Clostridiales
Family	Lachnospiraceae	Lachnospiraceae	Eubacteriaceae	Porphyromonadaceae	Ruminococcaceae	Lachnospiraceae	Ruminococcaceae	Lachnospiraceae	Lachnospiraceae
Genus	Blautia	Clostridium_XlVa	Eubacterium	Barnesiella	Saccharofermentans	Clostridium_XlVa	Clostridium_IV	Anaerostipes	Clostridium_XlVb
#NAME	ZOTU_0088	ZOTU_0089	ZOTU_0090	ZOTU_0091	ZOTU_0092	ZOTU_0093	ZOTU_0094	ZOTU_0095	ZOTU_0096
CD_49	2	17	0	119	5	0	26	0	9
CD_50	2	22	0	24	12	0	12	0	10
CD_51	9	51	0	107	37	3	2	0	31
CD_52	4	7	0	40	2	0	13	0	8
CD_65	0	17	0	1	2	0	6	0	2
CD_66	1	1	0	12	2	0	0	0	2
CD_67	0	3	0	1	0	2	0	0	0
CD_68	0	18	0	6	4	0	2	0	0
CD_78	2	1	0	9	1	0	6	0	5
CD_79	0	1	0	0	0	0	0	0	4
CD_80	0	0	0	0	1	0	2	0	1
CDFG_54	12	1	0	0	1	0	1	12	7
CDFG_55	7	1	0	11	12	1	33	10	2
CDFG_56	23	6	0	4	6	0	9	7	1
CDFG_70	20	1	0	1	2	2	7	0	1
CDFG_71	9	0	0	0	0	0	4	1	2
HF_41	0	6	6	1	3	0	5	0	2
HF_42	5	0	338	0	12	0	1	0	21
HF_43	7	31	3	0	1	1	9	0	1
HF_44	4	61	83	0	17	0	28	0	16
HF_57	4	36	0	8	1	9	1	0	14
HF_58	14	45	0	38	4	62	0	0	97
HF_59	5	49	0	28	3	28	0	0	31
HF_60	2	36	0	5	21	7	0	0	120
HF_73	2	44	1	3	1	1	19	0	3
HF_74	3	45	0	5	3	2	25	0	0
HF_75	10	3	0	12	8	0	0	0	5

Kingdom	Bacteria	Bacteria	Bacteria	Bacteria	Bacteria	Bacteria	Bacteria	Bacteria	Bacteria
Phylum	Firmicutes	Firmicutes	Firmicutes	Bacteroidetes	Firmicutes	Firmicutes	Firmicutes	Firmicutes	Firmicutes
Class	Clostridia	Clostridia	Clostridia	Bacteroidia	Clostridia	Clostridia	Clostridia	Clostridia	Clostridia
Order	Clostridiales	Clostridiales	Clostridiales	Bacteroidales	Clostridiales	Clostridiales	Clostridiales	Clostridiales	Clostridiales
Family	Lachnospiraceae	Lachnospiraceae	Eubacteriaceae	Porphyromonadaceae	Ruminococcaceae	Lachnospiraceae	Ruminococcaceae	Lachnospiraceae	Lachnospiraceae
Genus	Blautia	Clostridium_XlVa	Eubacterium	Barnesiella	Saccharofermentans	Clostridium_XlVa	Clostridium_IV	Anaerostipes	Clostridium_XlVb
#NAME	ZOTU_0088	ZOTU_0089	ZOTU_0090	ZOTU_0091	ZOTU_0092	ZOTU_0093	ZOTU_0094	ZOTU_0095	ZOTU_0096
HFFG_45	10	38	0	9	18	3	5	4	4
HFFG_46	1	4	0	1	2	1	1	7	0
HFFG_47	1	23	1	1	4	0	0	0	0
HFFG_61	1	5	0	5	1	1	5	5	0
HFFG_62	9	39	0	4	25	1	18	33	4
HFFG_63	1	13	0	2	2	0	3	2	0
HFFG_64	2	0	0	1	5	1	2	12	3
HFFG_76	8	36	0	37	15	0	5	0	6

Kingdom	Bacteria	Bacteria	Bacteria	Bacteria	Bacteria	Bacteria	Bacteria	Bacteria	Bacteria
Phylum	Firmicutes	Firmicutes	Firmicutes	Firmicutes	Firmicutes	Firmicutes	Firmicutes	Firmicutes	Firmicutes
Class	Clostridia	Clostridia	Clostridia	Clostridia	Clostridia	Clostridia	Clostridia	Clostridia	Clostridia
Order	Clostridiales	Clostridiales	Clostridiales	Clostridiales	Clostridiales	Clostridiales	Clostridiales	Clostridiales	Clostridiales
Family	Clostridiaceae_	Ruminococcaceae	Lachnospiraceae	Ruminococcaceae	Lachnospiraceae	Lachnospiraceae	Lachnospiraceae	Lachnospiraceae	Lachnospiraceae
Genus	Alkaliphilus	Clostridium_IV	Clostridium_XlVa	Pseudoflavonifractor	Acetatifactor	Clostridium_XlVa	Roseburia	Marvinbryantia	Clostridium_XlVa
#NAME	ZOTU_0097	ZOTU_0098	ZOTU_0099	ZOTU_0100	ZOTU_0101	ZOTU_0102	ZOTU_0103	ZOTU_0104	ZOTU_0105
CD_49	0	0	1	4	12	19	5	0	0
CD_50	0	0	0	5	0	0	5	5	0
CD_51	0	0	1	45	3	33	9	0	0
CD_52	0	0	0	4	2	11	3	1	0
CD_65	0	0	86	6	3	1	1	0	0
CD_66	0	0	37	4	1	6	0	1	0
CD_67	0	0	0	0	0	4	0	0	0
CD_68	0	0	59	15	6	5	2	0	0
CD_78	0	0	22	2	0	0	0	1	0
CD_79	0	0	3	1	0	0	0	0	0
CD_80	0	0	0	0	0	0	0	0	0
CDFG_54	0	29	2	5	0	9	1	4	0
CDFG_55	0	191	53	7	2	16	4	5	0
CDFG_56	0	27	181	2	1	23	7	4	0
CDFG_70	0	0	0	0	0	0	3	1	0
CDFG_71	0	14	1	0	0	1	3	2	0
HF_41	0	0	3	2	1	0	0	10	0
HF_42	1	0	6	0	1	2	1	0	0
HF_43	0	0	0	1	1	1	8	7	0
HF_44	0	0	0	2	2	5	12	12	0
HF_57	0	0	0	3	14	35	14	1	0
HF_58	0	0	0	14	109	79	33	0	0
HF_59	0	0	2	4	40	55	22	0	0
HF_60	0	0	1	6	1	39	0	0	0
HF_73	0	0	6	6	4	7	4	0	0
HF_74	0	0	8	6	1	11	13	0	0
HF_75	0	0	3	9	1	12	6	0	0

Kingdom	Bacteria	Bacteria	Bacteria	Bacteria	Bacteria	Bacteria	Bacteria	Bacteria	Bacteria
Phylum	Firmicutes	Firmicutes	Firmicutes	Firmicutes	Firmicutes	Firmicutes	Firmicutes	Firmicutes	Firmicutes
Class	Clostridia	Clostridia	Clostridia	Clostridia	Clostridia	Clostridia	Clostridia	Clostridia	Clostridia
Order	Clostridiales	Clostridiales	Clostridiales	Clostridiales	Clostridiales	Clostridiales	Clostridiales	Clostridiales	Clostridiales
Family	Clostridiaceae_	Ruminococcaceae	Lachnospiraceae	Ruminococcaceae	Lachnospiraceae	Lachnospiraceae	Lachnospiraceae	Lachnospiraceae	Lachnospiraceae
Genus	Alkaliphilus	Clostridium_IV	Clostridium_XlVa	Pseudoflavonifractor	Acetatifactor	Clostridium_XlVa	Roseburia	Marvinbryantia	Clostridium_XlVa
#NAME	ZOTU_0097	ZOTU_0098	ZOTU_0099	ZOTU_0100	ZOTU_0101	ZOTU_0102	ZOTU_0103	ZOTU_0104	ZOTU_0105
HFFG_45	0	0	0	12	7	27	37	0	1
HFFG_46	0	0	1	1	0	3	4	0	0
HFFG_47	0	0	0	1	2	6	7	0	0
HFFG_61	0	0	12	2	6	7	6	0	0
HFFG_62	0	0	71	30	2	44	45	0	0
HFFG_63	0	0	2	8	0	12	12	0	0
HFFG_64	0	0	0	2	1	0	0	0	0
HFFG_76	0	0	13	79	3	27	32	0	0

Kingdom	Bacteria	Bacteria	Bacteria	Bacteria	Bacteria	Bacteria	Bacteria	Bacteria	Bacteria
Phylum	Actinobacteria	Firmicutes	Firmicutes	Firmicutes	Firmicutes	Firmicutes	Firmicutes	Firmicutes	Firmicutes
Class	Actinobacteria	Clostridia	Clostridia	Clostridia	Clostridia	Clostridia	Clostridia	Clostridia	Clostridia
Order	Actinomycetales	Clostridiales	Clostridiales	Clostridiales	Clostridiales	Clostridiales	Clostridiales	Clostridiales	Clostridiales
Family	Mycobacteriaceae	Ruminococcaceae	Ruminococcaceae	Clostridiaceae_	Lachnospiraceae	Ruminococcaceae	Lachnospiraceae	Lachnospiraceae	Lachnospiraceae
Genus	Mycobacterium	Clostridium_IV	Flavonifractor	Alkaliphilus	Acetatifactor	Oscillibacter	Blautia	Clostridium_XlVa	Clostridium_XlVa
#NAME	ZOTU_0106	ZOTU_0107	ZOTU_0108	ZOTU_0109	ZOTU_0110	ZOTU_0111	ZOTU_0112	ZOTU_0113	ZOTU_0114
CD_49	0	3	1	0	0	9	0	2	7
CD_50	0	0	2	0	0	12	0	6	0
CD_51	0	11	2	0	0	16	0	48	10
CD_52	0	5	14	1	0	3	7	1	1
CD_65	0	2	0	0	0	10	0	1	0
CD_66	0	0	0	0	0	9	0	3	0
CD_67	0	1	0	0	0	0	0	1	6
CD_68	0	21	0	0	0	8	0	1	2
CD_78	0	0	0	0	0	0	0	0	0
CD_79	0	0	0	0	0	0	0	0	0
CD_80	0	0	0	0	0	0	0	0	0
CDFG_54	0	0	1	0	0	1	2	2	1
CDFG_55	0	1	5	0	1	21	3	14	5
CDFG_56	0	1	3	0	0	2	6	18	1
CDFG_70	0	1	1	0	0	1	7	17	0
CDFG_71	0	1	0	0	0	2	1	4	1
HF_41	0	2	31	3	0	4	0	0	0
HF_42	0	1	64	0	0	38	0	0	0
HF_43	0	2	4	0	0	17	0	2	3
HF_44	0	1	25	1	8	103	0	1	1
HF_57	0	35	0	10	0	6	0	0	0
HF_58	0	70	1	182	0	15	10	24	0
HF_59	0	61	1	1	0	5	10	12	0
HF_60	0	64	0	0	0	6	1	19	0
HF_73	0	11	1	1	0	15	1	5	0
HF_74	0	6	8	0	0	30	4	2	0
HF_75	0	11	1	66	0	1	7	11	3

Kingdom	Bacteria	Bacteria	Bacteria	Bacteria	Bacteria	Bacteria	Bacteria	Bacteria	Bacteria
Phylum	Actinobacteria	Firmicutes	Firmicutes	Firmicutes	Firmicutes	Firmicutes	Firmicutes	Firmicutes	Firmicutes
Class	Actinobacteria	Clostridia	Clostridia	Clostridia	Clostridia	Clostridia	Clostridia	Clostridia	Clostridia
Order	Actinomycetales	Clostridiales	Clostridiales	Clostridiales	Clostridiales	Clostridiales	Clostridiales	Clostridiales	Clostridiales
Family	Mycobacteriaceae	Ruminococcaceae	Ruminococcaceae	Clostridiaceae_	Lachnospiraceae	Ruminococcaceae	Lachnospiraceae	Lachnospiraceae	Lachnospiraceae
Genus	Mycobacterium	Clostridium_IV	Flavonifractor	Alkaliphilus	Acetatifactor	Oscillibacter	Blautia	Clostridium_XlVa	Clostridium_XlVa
#NAME	ZOTU_0106	ZOTU_0107	ZOTU_0108	ZOTU_0109	ZOTU_0110	ZOTU_0111	ZOTU_0112	ZOTU_0113	ZOTU_0114
HFFG_45	2	5	9	159	0	3	2	17	2
HFFG_46	0	5	0	11	0	0	1	0	3
HFFG_47	1	0	0	47	0	3	0	1	0
HFFG_61	0	0	15	0	0	0	0	1	2
HFFG_62	0	11	16	0	1	3	0	23	28
HFFG_63	0	5	14	0	0	2	0	9	10
HFFG_64	0	2	1	0	0	0	1	4	6
HFFG_76	0	70	1	0	0	5	15	29	30

Kingdom	Bacteria	Bacteria	Bacteria	Bacteria	Bacteria	Bacteria	Bacteria	Bacteria
Phylum	Firmicutes	Firmicutes	Firmicutes	Firmicutes	Firmicutes	Firmicutes	Firmicutes	Firmicutes
Class	Clostridia	Clostridia	Bacilli	Clostridia	Clostridia	Clostridia	Clostridia	Clostridia
Order	Clostridiales	Clostridiales	Bacillales	Clostridiales	Clostridiales	Clostridiales	Clostridiales	Clostridiales
Family	Lachnospiraceae	Lachnospiraceae	Bacillaceae_	Lachnospiraceae	Clostridiales_Incertae_Sedis_XII	Ruminococcaceae	Lachnospiraceae	Lachnospiraceae
Genus	Clostridium_XlVa	Clostridium_XlVa	Bacillus	Clostridium_XlVa	Guggenheimella	Clostridium_IV	Clostridium_XlVa	Lachnospiracea_incertae_sedis
#NAME	ZOTU_0115	ZOTU_0116	ZOTU_0117	ZOTU_0118	ZOTU_0119	ZOTU_0120	ZOTU_0121	ZOTU_0122
CD_49	8	0	0	0	0	0	0	12
CD_50	9	0	0	0	0	0	0	1
CD_51	6	0	0	0	0	2	3	14
CD_52	5	0	0	0	0	0	0	1
CD_65	3	0	0	0	0	1	5	0
CD_66	1	0	0	0	0	0	0	0
CD_67	2	0	0	0	0	0	2	0
CD_68	1	0	0	0	0	0	3	0
CD_78	0	0	0	0	0	0	1	0
CD_79	1	0	0	0	0	0	1	0
CD_80	0	0	0	0	0	0	0	0
CDFG_54	3	2	0	0	0	0	0	3
CDFG_55	5	112	0	0	0	2	0	11
CDFG_56	0	13	0	0	0	0	0	11
CDFG_70	0	66	0	0	0	0	0	0
CDFG_71	0	4	0	0	0	0	0	0
HF_41	2	0	0	0	0	2	0	0
HF_42	3	0	0	0	4	1	2	0
HF_43	5	0	0	0	0	1	0	2
HF_44	18	0	0	0	0	1	0	0
HF_57	31	0	0	0	1	0	0	9
HF_58	31	0	0	0	0	0	4	17
HF_59	4	0	0	0	0	0	0	11
HF_60	3	0	0	0	0	0	0	2
HF_73	5	0	0	0	0	0	2	1
HF_74	7	5	0	0	0	7	8	19
HF_75	2	0	0	0	0	0	4	0

Kingdom	Bacteria	Bacteria	Bacteria	Bacteria	Bacteria	Bacteria	Bacteria	Bacteria
Phylum	Firmicutes	Firmicutes	Firmicutes	Firmicutes	Firmicutes	Firmicutes	Firmicutes	Firmicutes
Class	Clostridia	Clostridia	Bacilli	Clostridia	Clostridia	Clostridia	Clostridia	Clostridia
Order	Clostridiales	Clostridiales	Bacillales	Clostridiales	Clostridiales	Clostridiales	Clostridiales	Clostridiales
Family	Lachnospiraceae	Lachnospiraceae	Bacillaceae_	Lachnospiraceae	Clostridiales_Incertae_Sedis_XII	Ruminococcaceae	Lachnospiraceae	Lachnospiraceae
Genus	Clostridium_XlVa	Clostridium_XlVa	Bacillus	Clostridium_XlVa	Guggenheimella	Clostridium_IV	Clostridium_XlVa	Lachnospiracea_incertae_sedis
#NAME	ZOTU_0115	ZOTU_0116	ZOTU_0117	ZOTU_0118	ZOTU_0119	ZOTU_0120	ZOTU_0121	ZOTU_0122
HFFG_45	42	197	0	0	0	2	4	0
HFFG_46	2	5	0	0	0	0	0	0
HFFG_47	6	26	0	0	0	0	1	0
HFFG_61	1	0	0	0	0	0	3	0
HFFG_62	2	0	0	0	0	4	31	0
HFFG_63	0	0	0	0	0	0	9	0
HFFG_64	0	0	0	0	0	0	0	0
HFFG_76	12	0	0	0	0	2	25	3

Kingdom	Bacteria	Bacteria	Bacteria	Bacteria	Bacteria	Bacteria	Bacteria	Bacteria	Bacteria
Phylum	Firmicutes	Firmicutes	Firmicutes	Firmicutes	Firmicutes	Firmicutes	Firmicutes	Firmicutes	Firmicutes
Class	Clostridia	Clostridia	Bacilli	Clostridia	Clostridia	Clostridia	Clostridia	Clostridia	Clostridia
Order	Clostridiales	Clostridiales	Bacillales	Clostridiales	Clostridiales	Clostridiales	Clostridiales	Clostridiales	Clostridiales
Family	Lachnospiraceae	Lachnospiraceae	Paenibacillaceae_	Clostridiaceae_	Lachnospiraceae	Lachnospiraceae	Lachnospiraceae	Lachnospiraceae	Eubacteriaceae
Genus	Clostridium_XlVa	Clostridium_XlVa	Brevibacillus	Clostridium_sensu_stricto	Clostridium_XlVa	Clostridium_XlVa	Acetatifactor	Clostridium_XlVa	Eubacterium
#NAME	ZOTU_0123	ZOTU_0124	ZOTU_0125	ZOTU_0126	ZOTU_0127	ZOTU_0128	ZOTU_0129	ZOTU_0130	ZOTU_0131
CD_49	0	17	0	23	2	0	1	0	7
CD_50	1	2	0	20	0	0	1	0	6
CD_51	6	40	0	45	10	0	0	1	12
CD_52	1	2	0	0	1	0	0	0	4
CD_65	0	0	0	11	0	0	1	1	2
CD_66	0	2	0	3	1	0	0	0	2
CD_67	0	0	0	0	1	0	1	0	5
CD_68	0	2	0	9	3	0	3	1	1
CD_78	0	0	0	4	2	0	0	0	2
CD_79	0	0	0	3	0	0	0	0	0
CD_80	0	0	0	1	0	0	0	0	1
CDFG_54	7	2	0	8	1	0	0	2	0
CDFG_55	170	1	0	12	0	0	5	6	0
CDFG_56	68	12	0	17	2	0	3	0	0
CDFG_70	2	1	0	0	0	0	0	0	2
CDFG_71	2	0	0	2	0	0	0	2	2
HF_41	0	0	0	0	0	0	0	0	2
HF_42	0	2	0	4	3	0	0	2	1
HF_43	0	0	0	0	1	0	2	6	2
HF_44	0	0	0	20	2	0	4	1	8
HF_57	5	3	0	36	29	0	1	2	1
HF_58	21	10	0	76	198	0	2	11	13
HF_59	24	3	0	32	153	0	1	4	7
HF_60	6	0	0	17	70	0	0	1	26
HF_73	39	0	0	10	6	0	0	11	4
HF_74	9	0	0	0	8	0	8	6	2
HF_75	0	0	0	4	4	0	4	3	2

Kingdom	Bacteria	Bacteria	Bacteria	Bacteria	Bacteria	Bacteria	Bacteria	Bacteria	Bacteria
Phylum	Firmicutes	Firmicutes	Firmicutes	Firmicutes	Firmicutes	Firmicutes	Firmicutes	Firmicutes	Firmicutes
Class	Clostridia	Clostridia	Bacilli	Clostridia	Clostridia	Clostridia	Clostridia	Clostridia	Clostridia
Order	Clostridiales	Clostridiales	Bacillales	Clostridiales	Clostridiales	Clostridiales	Clostridiales	Clostridiales	Clostridiales
Family	Lachnospiraceae	Lachnospiraceae	Paenibacillaceae_	Clostridiaceae_	Lachnospiraceae	Lachnospiraceae	Lachnospiraceae	Lachnospiraceae	Eubacteriaceae
Genus	Clostridium_XlVa	Clostridium_XlVa	Brevibacillus	Clostridium_sensu_stricto	Clostridium_XlVa	Clostridium_XlVa	Acetatifactor	Clostridium_XlVa	Eubacterium
#NAME	ZOTU_0123	ZOTU_0124	ZOTU_0125	ZOTU_0126	ZOTU_0127	ZOTU_0128	ZOTU_0129	ZOTU_0130	ZOTU_0131
HFFG_45	11	7	0	47	20	0	7	0	6
HFFG_46	6	0	0	7	9	0	6	1	0
HFFG_47	11	0	0	3	5	0	0	0	0
HFFG_61	1	0	0	1	3	0	3	0	2
HFFG_62	4	0	0	6	27	0	29	24	1
HFFG_63	1	0	0	2	4	0	3	6	0
HFFG_64	1	1	0	0	0	0	2	1	0
HFFG_76	16	0	0	0	25	0	12	7	2

Kingdom	Bacteria	Bacteria	Bacteria	Bacteria	Bacteria	Bacteria	Bacteria	Bacteria
Phylum	Firmicutes	Firmicutes	Firmicutes	Firmicutes	Firmicutes	Firmicutes	Firmicutes	Firmicutes
Class	Clostridia	Clostridia	Erysipelotrichia	Clostridia	Clostridia	Erysipelotrichia	Clostridia	Clostridia
Order	Clostridiales	Clostridiales	Erysipelotrichales	Clostridiales	Clostridiales	Erysipelotrichales	Clostridiales	Clostridiales
Family	Ruminococcaceae	Lachnospiraceae	Erysipelotrichaceae	Lachnospiraceae	Lachnospiraceae	Erysipelotrichaceae	Lachnospiraceae	Ruminococcaceae
Genus	Clostridium_IV	Acetatifactor	Coprobacillus	Clostridium_XlVa	Acetatifactor	Erysipelotrichaceae_incertae_sedis	Dorea	Clostridium_IV
#NAME	ZOTU_0132	ZOTU_0133	ZOTU_0134	ZOTU_0135	ZOTU_0136	ZOTU_0137	ZOTU_0138	ZOTU_0139
CD_49	3	1	0	12	0	1	0	3
CD_50	5	0	1	5	0	2	0	0
CD_51	16	1	11	21	0	10	0	1
CD_52	2	0	0	3	0	2	0	4
CD_65	2	0	0	2	0	0	0	3
CD_66	1	0	0	1	1	0	0	1
CD_67	2	0	0	0	0	0	0	1
CD_68	4	0	0	6	5	1	0	3
CD_78	14	0	13	2	0	1	0	0
CD_79	1	0	2	3	0	0	0	0
CD_80	3	0	3	0	0	1	0	0
CDFG_54	2	0	2	2	0	0	0	4
CDFG_55	1	2	0	9	0	4	1	0
CDFG_56	1	1	2	15	1	14	9	2
CDFG_70	0	1	0	2	0	1	1	4
CDFG_71	0	0	0	0	0	0	1	0
HF_41	1	3	0	1	0	4	0	0
HF_42	0	4	0	1	0	6	0	1
HF_43	5	0	0	1	1	2	0	11
HF_44	0	1	0	3	1	4	0	1
HF_57	1	0	0	8	6	2	0	10
HF_58	30	0	6	31	18	0	0	47
HF_59	18	0	6	20	6	6	0	45
HF_60	0	0	4	16	3	5	0	9
HF_73	0	0	0	4	1	0	0	4
HF_74	10	0	0	1	3	1	0	24
HF_75	8	0	2	2	0	5	6	20

Kingdom	Bacteria	Bacteria	Bacteria	Bacteria	Bacteria	Bacteria	Bacteria	Bacteria
Phylum	Firmicutes	Firmicutes	Firmicutes	Firmicutes	Firmicutes	Firmicutes	Firmicutes	Firmicutes
Class	Clostridia	Clostridia	Erysipelotrichia	Clostridia	Clostridia	Erysipelotrichia	Clostridia	Clostridia
Order	Clostridiales	Clostridiales	Erysipelotrichales	Clostridiales	Clostridiales	Erysipelotrichales	Clostridiales	Clostridiales
Family	Ruminococcaceae	Lachnospiraceae	Erysipelotrichaceae	Lachnospiraceae	Lachnospiraceae	Erysipelotrichaceae	Lachnospiraceae	Ruminococcaceae
Genus	Clostridium_IV	Acetatifactor	Coprobacillus	Clostridium_XlVa	Acetatifactor	Erysipelotrichaceae_incertae_sedis	Dorea	Clostridium_IV
#NAME	ZOTU_0132	ZOTU_0133	ZOTU_0134	ZOTU_0135	ZOTU_0136	ZOTU_0137	ZOTU_0138	ZOTU_0139
HFFG_45	3	0	15	23	0	3	2	0
HFFG_46	1	0	0	2	0	0	0	3
HFFG_47	0	0	1	3	0	0	0	0
HFFG_61	2	0	0	1	0	0	2	1
HFFG_62	12	0	0	12	2	5	16	43
HFFG_63	3	0	3	12	0	4	5	14
HFFG_64	3	0	2	1	2	2	4	2
HFFG_76	34	0	10	12	7	3	33	42

Kingdom	Bacteria	Bacteria	Bacteria	Bacteria	Bacteria	Bacteria	Bacteria	Bacteria
Phylum	Firmicutes	Firmicutes	Firmicutes	Firmicutes	Firmicutes	Firmicutes	Firmicutes	Firmicutes
Class	Erysipelotrichia	Clostridia	Clostridia	Clostridia	Clostridia	Clostridia	Clostridia	Clostridia
Order	Erysipelotrichales	Clostridiales	Clostridiales	Clostridiales	Clostridiales	Clostridiales	Clostridiales	Clostridiales
Family	Erysipelotrichaceae	Lachnospiraceae	Clostridiaceae_	Lachnospiraceae	Ruminococcaceae	Clostridiaceae_	Ruminococcaceae	Ruminococcaceae
Genus	Erysipelotrichaceae_incertae_sedis	Eisenbergiella	Alkaliphilus	Clostridium_XlVa	Intestinimonas	Alkaliphilus	Ruminococcus	Clostridium_IV
#NAME	ZOTU_0140	ZOTU_0141	ZOTU_0142	ZOTU_0143	ZOTU_0144	ZOTU_0145	ZOTU_0146	ZOTU_0147
CD_49	0	6	0	15	6	0	0	0
CD_50	0	1	0	4	4	0	1	0
CD_51	0	14	0	36	24	0	0	4
CD_52	5	1	0	4	5	0	1	0
CD_65	0	7	0	1	2	0	0	0
CD_66	0	2	0	4	1	0	0	0
CD_67	0	1	0	2	0	0	0	0
CD_68	0	25	0	0	6	0	0	0
CD_78	0	0	0	0	0	0	0	3
CD_79	0	0	0	0	2	0	0	0
CD_80	0	0	0	0	0	0	0	0
CDFG_54	0	0	0	0	0	2	3	5
CDFG_55	18	4	1	8	4	11	19	14
CDFG_56	0	4	0	4	1	12	4	3
CDFG_70	1	1	0	4	2	4	0	43
CDFG_71	0	0	0	3	1	2	3	2
HF_41	0	1	0	0	0	0	0	0
HF_42	0	0	0	0	0	0	2	0
HF_43	0	2	0	0	3	0	0	0
HF_44	0	15	0	0	1	0	0	0
HF_57	1	4	0	6	29	0	2	0
HF_58	40	15	0	8	61	0	5	3
HF_59	17	35	0	11	28	0	5	5
HF_60	11	4	0	3	15	0	3	0
HF_73	9	1	0	2	1	0	1	0
HF_74	0	4	0	0	10	0	2	2
HF_75	0	5	0	1	1	0	5	4

Kingdom	Bacteria	Bacteria	Bacteria	Bacteria	Bacteria	Bacteria	Bacteria	Bacteria
Phylum	Firmicutes	Firmicutes	Firmicutes	Firmicutes	Firmicutes	Firmicutes	Firmicutes	Firmicutes
Class	Erysipelotrichia	Clostridia	Clostridia	Clostridia	Clostridia	Clostridia	Clostridia	Clostridia
Order	Erysipelotrichales	Clostridiales	Clostridiales	Clostridiales	Clostridiales	Clostridiales	Clostridiales	Clostridiales
Family	Erysipelotrichaceae	Lachnospiraceae	Clostridiaceae_	Lachnospiraceae	Ruminococcaceae	Clostridiaceae_	Ruminococcaceae	Ruminococcaceae
Genus	Erysipelotrichaceae_incertae_sedis	Eisenbergiella	Alkaliphilus	Clostridium_XlVa	Intestinimonas	Alkaliphilus	Ruminococcus	Clostridium_IV
#NAME	ZOTU_0140	ZOTU_0141	ZOTU_0142	ZOTU_0143	ZOTU_0144	ZOTU_0145	ZOTU_0146	ZOTU_0147
HFFG_45	25	37	0	5	11	0	3	1
HFFG_46	0	7	0	0	0	0	2	10
HFFG_47	3	0	0	2	0	0	0	1
HFFG_61	3	2	0	0	0	0	1	0
HFFG_62	29	47	0	8	3	0	5	0
HFFG_63	0	0	0	2	2	0	7	0
HFFG_64	2	5	0	0	0	0	0	0
HFFG_76	14	12	0	10	22	0	9	9

Kingdom	Bacteria	Bacteria	Bacteria	Bacteria	Bacteria	Bacteria	Bacteria	Bacteria	Bacteria
Phylum	Firmicutes	Firmicutes	Firmicutes	Firmicutes	Firmicutes	Firmicutes	Firmicutes	Actinobacteria	Firmicutes
Class	Clostridia	Clostridia	Clostridia	Clostridia	Clostridia	Clostridia	Clostridia	Actinobacteria	Clostridia
Order	Clostridiales	Clostridiales	Clostridiales	Clostridiales	Clostridiales	Clostridiales	Clostridiales	Coriobacteriales	Clostridiales
Family	Lachnospiraceae	Lachnospiraceae	Clostridiaceae_	Clostridiaceae_	Eubacteriaceae	Lachnospiraceae	Ruminococcaceae	Coriobacteriaceae	Proteinivoraceae
Genus	Anaerosporobacter	Butyrivibrio	Alkaliphilus	Alkaliphilus	Eubacterium	Clostridium_XlVa	Flavonifractor	Adlercreutzia	Proteinivorax
#NAME	ZOTU_0148	ZOTU_0149	ZOTU_0150	ZOTU_0151	ZOTU_0152	ZOTU_0153	ZOTU_0154	ZOTU_0155	ZOTU_0156
CD_49	0	0	0	1	13	3	0	0	0
CD_50	2	0	0	1	4	2	11	0	4
CD_51	8	2	0	1	10	9	0	0	4
CD_52	1	0	0	0	14	2	1	7	1
CD_65	0	0	0	1	3	3	0	0	1
CD_66	1	0	0	0	2	2	2	0	0
CD_67	3	0	0	1	2	1	0	0	0
CD_68	1	0	0	0	0	3	14	0	0
CD_78	1	0	0	1	2	1	0	0	0
CD_79	0	0	0	0	1	0	0	0	0
CD_80	0	0	0	0	0	0	0	0	0
CDFG_54	3	0	0	0	1	0	0	2	0
CDFG_55	18	1	0	2	4	0	0	0	5
CDFG_56	14	2	0	2	4	1	4	0	2
CDFG_70	1	0	0	2	8	1	0	0	0
CDFG_71	3	0	0	0	2	0	1	0	0
HF_41	2	0	0	0	0	0	0	0	2
HF_42	1	0	0	0	1	0	0	0	0
HF_43	3	0	0	0	0	1	0	0	0
HF_44	2	0	0	0	3	0	0	0	9
HF_57	5	0	0	0	1	7	0	0	3
HF_58	10	0	0	0	4	10	0	0	14
HF_59	2	0	0	0	5	6	0	0	7
HF_60	2	0	0	0	23	0	0	0	9
HF_73	1	0	0	0	1	8	0	0	2
HF_74	0	0	0	0	1	12	8	1	1
HF_75	0	1	0	2	2	2	0	0	0

Kingdom	Bacteria	Bacteria	Bacteria	Bacteria	Bacteria	Bacteria	Bacteria	Bacteria	Bacteria
Phylum	Firmicutes	Firmicutes	Firmicutes	Firmicutes	Firmicutes	Firmicutes	Firmicutes	Actinobacteria	Firmicutes
Class	Clostridia	Clostridia	Clostridia	Clostridia	Clostridia	Clostridia	Clostridia	Actinobacteria	Clostridia
Order	Clostridiales	Clostridiales	Clostridiales	Clostridiales	Clostridiales	Clostridiales	Clostridiales	Coriobacteriales	Clostridiales
Family	Lachnospiraceae	Lachnospiraceae	Clostridiaceae_	Clostridiaceae_	Eubacteriaceae	Lachnospiraceae	Ruminococcaceae	Coriobacteriaceae	Proteinivoraceae
Genus	Anaerosporobacter	Butyrivibrio	Alkaliphilus	Alkaliphilus	Eubacterium	Clostridium_XlVa	Flavonifractor	Adlercreutzia	Proteinivorax
#NAME	ZOTU_0148	ZOTU_0149	ZOTU_0150	ZOTU_0151	ZOTU_0152	ZOTU_0153	ZOTU_0154	ZOTU_0155	ZOTU_0156
HFFG_45	6	1	0	0	13	4	23	0	5
HFFG_46	0	0	0	0	0	5	0	0	1
HFFG_47	1	0	0	0	1	0	0	0	0
HFFG_61	3	0	0	1	0	0	0	0	0
HFFG_62	8	3	0	12	4	37	0	3	5
HFFG_63	0	0	0	0	0	5	0	1	0
HFFG_64	0	0	0	7	0	0	0	1	0
HFFG_76	3	2	0	5	5	6	0	0	1

Kingdom	Bacteria	Bacteria	Bacteria	Bacteria	Bacteria	Bacteria	Bacteria
Phylum	Firmicutes	Bacteroidetes	Firmicutes	Firmicutes	Firmicutes	Firmicutes	Firmicutes
Class	Clostridia	Bacteroidia	Clostridia	Clostridia	Clostridia	Clostridia	Clostridia
Order	Clostridiales	Bacteroidales	Clostridiales	Clostridiales	Clostridiales	Clostridiales	Clostridiales
Family	Lachnospiraceae	Porphyromonadaceae	Lachnospiraceae	Lachnospiraceae	Lachnospiraceae	Lachnospiraceae	Lachnospiraceae
Genus	Lachnospiracea_incertae_sedis	Barnesiella	Clostridium_XlVa	Clostridium_XlVa	Clostridium_XlVa	Butyrivibrio	Clostridium_XlVa
#NAME	ZOTU_0157	ZOTU_0158	ZOTU_0159	ZOTU_0160	ZOTU_0161	ZOTU_0162	ZOTU_0163
CD_49	3	0	0	0	2	0	2
CD_50	8	0	0	0	0	0	3
CD_51	11	0	0	1	0	0	3
CD_52	3	0	0	0	0	0	1
CD_65	1	0	0	0	0	0	0
CD_66	0	0	0	0	0	0	3
CD_67	1	1	0	0	0	0	0
CD_68	2	0	0	0	5	0	2
CD_78	5	0	0	0	0	0	0
CD_79	1	0	0	0	0	0	0
CD_80	0	0	0	0	0	0	0
CDFG_54	4	0	0	0	0	0	0
CDFG_55	17	0	0	0	1	0	1
CDFG_56	12	0	2	0	1	1	4
CDFG_70	4	0	0	0	0	0	0
CDFG_71	4	0	0	0	0	0	0
HF_41	0	2	0	0	1	0	0
HF_42	0	0	0	0	0	0	0
HF_43	0	0	0	0	0	0	0
HF_44	2	0	0	0	0	0	1
HF_57	1	0	0	1	19	0	0
HF_58	0	0	0	0	53	0	4
HF_59	0	1	0	0	67	0	1
HF_60	2	0	0	0	4	0	2
HF_73	4	0	0	0	4	0	1
HF_74	2	0	0	0	13	0	0
HF_75	0	0	2	3	0	2	15

Kingdom	Bacteria	Bacteria	Bacteria	Bacteria	Bacteria	Bacteria	Bacteria
Phylum	Firmicutes	Bacteroidetes	Firmicutes	Firmicutes	Firmicutes	Firmicutes	Firmicutes
Class	Clostridia	Bacteroidia	Clostridia	Clostridia	Clostridia	Clostridia	Clostridia
Order	Clostridiales	Bacteroidales	Clostridiales	Clostridiales	Clostridiales	Clostridiales	Clostridiales
Family	Lachnospiraceae	Porphyromonadaceae	Lachnospiraceae	Lachnospiraceae	Lachnospiraceae	Lachnospiraceae	Lachnospiraceae
Genus	Lachnospiracea_incertae_sedis	Barnesiella	Clostridium_XlVa	Clostridium_XlVa	Clostridium_XlVa	Butyrivibrio	Clostridium_XlVa
#NAME	ZOTU_0157	ZOTU_0158	ZOTU_0159	ZOTU_0160	ZOTU_0161	ZOTU_0162	ZOTU_0163
HFFG_45	0	0	2	1	0	1	23
HFFG_46	0	0	1	1	0	1	1
HFFG_47	1	0	1	1	0	0	5
HFFG_61	0	0	0	0	0	0	0
HFFG_62	2	0	14	0	0	2	15
HFFG_63	0	0	2	0	0	0	5
HFFG_64	0	0	2	0	0	2	2
HFFG_76	1	0	23	9	0	1	20

Kingdom	Bacteria	Bacteria	Bacteria	Bacteria	Bacteria	Bacteria	Bacteria	Bacteria
Phylum	Firmicutes	Firmicutes	Firmicutes	Firmicutes	Firmicutes	Firmicutes	Firmicutes	Tenericutes
Class	Clostridia	Clostridia	Clostridia	Clostridia	Clostridia	Clostridia	Clostridia	Mollicutes
Order	Clostridiales	Clostridiales	Clostridiales	Clostridiales	Clostridiales	Clostridiales	Clostridiales	Anaeroplasmatales
Family	Lachnospiraceae	Clostridiaceae_	Lachnospiraceae	Lachnospiraceae	Ruminococcaceae	Lachnospiraceae	Lachnospiraceae	Anaeroplasmataceae
Genus	Lachnospiracea_incertae_sedis	Geosporobacter	Clostridium_XlVa	Clostridium_XlVa	Anaerotruncus	Clostridium_XlVa	Clostridium_XlVa	Anaeroplasma
#NAME	ZOTU_0164	ZOTU_0165	ZOTU_0166	ZOTU_0167	ZOTU_0168	ZOTU_0169	ZOTU_0170	ZOTU_0171
CD_49	0	2	1	6	13	1	1	0
CD_50	0	2	2	0	1	4	0	0
CD_51	0	2	8	4	26	1	10	0
CD_52	0	8	1	0	4	1	0	0
CD_65	0	1	1	1	0	2	1	1
CD_66	0	1	1	0	2	0	0	1
CD_67	0	1	2	0	1	0	0	5
CD_68	0	0	1	1	1	3	10	0
CD_78	0	6	1	0	0	0	0	0
CD_79	0	7	0	0	0	0	0	0
CD_80	0	3	0	0	0	0	0	0
CDFG_54	0	0	1	8	0	0	0	0
CDFG_55	0	2	8	10	1	9	1	0
CDFG_56	0	3	9	6	2	25	0	0
CDFG_70	0	0	0	0	0	5	0	0
CDFG_71	0	0	0	0	0	1	0	0
HF_41	0	1	0	0	0	0	0	0
HF_42	0	0	1	1	0	0	0	0
HF_43	0	0	2	0	2	0	0	0
HF_44	0	0	3	1	3	0	0	0
HF_57	0	0	0	1	1	0	7	0
HF_58	0	1	4	4	11	0	20	1
HF_59	0	2	1	0	13	0	25	0
HF_60	0	3	4	1	5	0	10	0
HF_73	0	1	0	2	2	1	3	0
HF_74	0	0	5	6	9	16	3	0
HF_75	0	6	3	7	3	0	3	0

Kingdom	Bacteria	Bacteria	Bacteria	Bacteria	Bacteria	Bacteria	Bacteria	Bacteria
Phylum	Firmicutes	Firmicutes	Firmicutes	Firmicutes	Firmicutes	Firmicutes	Firmicutes	Tenericutes
Class	Clostridia	Clostridia	Clostridia	Clostridia	Clostridia	Clostridia	Clostridia	Mollicutes
Order	Clostridiales	Clostridiales	Clostridiales	Clostridiales	Clostridiales	Clostridiales	Clostridiales	Anaeroplasmatales
Family	Lachnospiraceae	Clostridiaceae_	Lachnospiraceae	Lachnospiraceae	Ruminococcaceae	Lachnospiraceae	Lachnospiraceae	Anaeroplasmataceae
Genus	Lachnospiracea_incertae_sedis	Geosporobacter	Clostridium_XlVa	Clostridium_XlVa	Anaerotruncus	Clostridium_XlVa	Clostridium_XlVa	Anaeroplasma
#NAME	ZOTU_0164	ZOTU_0165	ZOTU_0166	ZOTU_0167	ZOTU_0168	ZOTU_0169	ZOTU_0170	ZOTU_0171
HFFG_45	0	0	3	11	4	1	7	0
HFFG_46	0	1	0	12	0	0	2	0
HFFG_47	0	0	0	7	0	0	3	0
HFFG_61	0	0	0	1	1	1	1	0
HFFG_62	0	3	3	30	7	4	4	0
HFFG_63	0	1	1	15	2	0	3	0
HFFG_64	0	1	0	7	0	2	0	0
HFFG_76	0	4	2	35	35	0	6	0

Kingdom	Bacteria	Bacteria	Bacteria	Bacteria	Bacteria	Bacteria	Bacteria	Bacteria	Bacteria
Phylum	Firmicutes	Firmicutes	Firmicutes	Firmicutes	Firmicutes	Firmicutes	Firmicutes	Firmicutes	Tenericutes
Class	Clostridia	Clostridia	Clostridia	Clostridia	Clostridia	Clostridia	Clostridia	Clostridia	Mollicutes
Order	Clostridiales	Clostridiales	Clostridiales	Clostridiales	Clostridiales	Clostridiales	Clostridiales	Clostridiales	Entomoplasmatales
Family	Clostridiaceae_	Ruminococcaceae	Lachnospiraceae	Lachnospiraceae	Ruminococcaceae	Lachnospiraceae	Lachnospiraceae	Ruminococcaceae	Spiroplasmataceae
Genus	Alkaliphilus	Oscillibacter	Acetatifactor	Blautia	Clostridium_IV	Dorea	Clostridium_XlVa	Oscillibacter	Spiroplasma
#NAME	ZOTU_0172	ZOTU_0173	ZOTU_0174	ZOTU_0175	ZOTU_0176	ZOTU_0177	ZOTU_0178	ZOTU_0179	ZOTU_0180
CD_49	2	0	1	0	1	0	3	8	0
CD_50	0	0	2	0	2	0	3	1	2
CD_51	0	1	13	0	4	0	14	19	13
CD_52	0	1	2	0	1	0	0	0	0
CD_65	0	0	1	0	2	0	5	1	0
CD_66	0	0	1	0	1	0	1	2	0
CD_67	0	0	0	0	0	0	1	0	0
CD_68	0	0	2	0	4	0	7	1	0
CD_78	0	0	0	0	0	0	0	0	1
CD_79	0	0	0	0	0	0	0	0	0
CD_80	0	0	0	0	0	0	0	0	1
CDFG_54	0	2	0	0	10	3	5	0	0
CDFG_55	0	3	0	0	5	0	3	1	0
CDFG_56	0	1	2	0	11	2	11	0	0
CDFG_70	0	1	2	0	2	1	0	0	5
CDFG_71	0	0	0	0	0	0	1	0	2
HF_41	0	1	0	0	0	0	0	0	0
HF_42	1	1	0	0	1	0	1	0	2
HF_43	3	0	1	0	1	0	1	0	1
HF_44	1	6	0	0	3	0	1	0	1
HF_57	0	0	1	0	0	0	0	8	0
HF_58	0	2	2	0	10	1	4	52	10
HF_59	0	3	1	0	5	0	1	9	6
HF_60	0	3	2	0	1	0	1	11	6
HF_73	0	1	7	0	2	0	1	0	1
HF_74	0	1	2	0	2	0	1	2	4
HF_75	0	0	0	0	3	5	0	0	1

Kingdom	Bacteria	Bacteria	Bacteria	Bacteria	Bacteria	Bacteria	Bacteria	Bacteria	Bacteria
Phylum	Firmicutes	Firmicutes	Firmicutes	Firmicutes	Firmicutes	Firmicutes	Firmicutes	Firmicutes	Tenericutes
Class	Clostridia	Clostridia	Clostridia	Clostridia	Clostridia	Clostridia	Clostridia	Clostridia	Mollicutes
Order	Clostridiales	Clostridiales	Clostridiales	Clostridiales	Clostridiales	Clostridiales	Clostridiales	Clostridiales	Entomoplasmatales
Family	Clostridiaceae_	Ruminococcaceae	Lachnospiraceae	Lachnospiraceae	Ruminococcaceae	Lachnospiraceae	Lachnospiraceae	Ruminococcaceae	Spiroplasmataceae
Genus	Alkaliphilus	Oscillibacter	Acetatifactor	Blautia	Clostridium_IV	Dorea	Clostridium_XlVa	Oscillibacter	Spiroplasma
#NAME	ZOTU_0172	ZOTU_0173	ZOTU_0174	ZOTU_0175	ZOTU_0176	ZOTU_0177	ZOTU_0178	ZOTU_0179	ZOTU_0180
HFFG_45	0	6	4	0	12	0	6	8	0
HFFG_46	0	0	0	0	1	0	0	1	0
HFFG_47	0	0	0	0	4	0	0	0	0
HFFG_61	0	0	0	0	0	0	0	1	1
HFFG_62	0	1	2	0	5	14	2	1	1
HFFG_63	0	0	0	0	3	0	0	0	0
HFFG_64	0	0	0	0	1	1	0	0	0
HFFG_76	0	2	6	0	25	20	2	11	5

Kingdom	Bacteria	Bacteria	Bacteria	Bacteria	Bacteria	Bacteria	Bacteria	Bacteria	Bacteria
Phylum	Firmicutes	Firmicutes	Firmicutes	Firmicutes	Firmicutes	Firmicutes	Firmicutes	Firmicutes	Firmicutes
Class	Clostridia	Clostridia	Clostridia	Clostridia	Clostridia	Clostridia	Clostridia	Clostridia	Clostridia
Order	Clostridiales	Clostridiales	Clostridiales	Clostridiales	Clostridiales	Clostridiales	Clostridiales	Halanaerobiales	Clostridiales
Family	Lachnospiraceae	Lachnospiraceae	Ruminococcaceae	Lachnospiraceae	Lachnospiraceae	Lachnospiraceae	Lachnospiraceae	Halanaerobiaceae	Ruminococcaceae
Genus	Acetatifactor	Clostridium_XlVa	Butyricicoccus	Clostridium_XlVa	Clostridium_XlVa	Lachnospira	Clostridium_XlVa	Halanaerobium	Clostridium_IV
#NAME	ZOTU_0181	ZOTU_0182	ZOTU_0183	ZOTU_0184	ZOTU_0185	ZOTU_0186	ZOTU_0187	ZOTU_0188	ZOTU_0189
CD_49	7	0	1	2	1	0	1	0	0
CD_50	3	0	0	0	0	0	6	0	0
CD_51	4	2	10	0	0	0	7	0	0
CD_52	1	0	0	0	0	0	0	0	1
CD_65	1	0	1	1	0	0	3	0	0
CD_66	1	1	1	0	0	0	0	0	0
CD_67	2	14	0	0	0	0	0	0	0
CD_68	8	6	0	1	2	0	0	0	0
CD_78	0	0	0	0	0	0	0	0	0
CD_79	0	0	0	0	0	0	0	0	0
CD_80	0	0	0	0	0	0	0	0	0
CDFG_54	0	0	0	0	0	1	0	0	0
CDFG_55	2	0	0	0	0	6	2	0	0
CDFG_56	0	4	2	0	1	3	3	0	1
CDFG_70	0	0	0	0	0	0	0	0	0
CDFG_71	0	0	0	0	0	0	0	0	1
HF_41	0	0	0	0	0	0	0	0	2
HF_42	0	0	0	0	0	0	0	0	0
HF_43	1	2	0	0	0	0	0	0	3
HF_44	0	0	0	0	0	0	3	0	0
HF_57	2	0	1	20	16	0	0	0	0
HF_58	13	3	6	34	36	0	5	0	0
HF_59	7	2	3	32	43	0	2	0	0
HF_60	2	0	7	2	5	0	0	0	0
HF_73	1	0	0	0	1	0	3	0	2
HF_74	6	1	0	7	9	0	1	0	14
HF_75	1	0	7	0	0	1	2	0	0

Kingdom	Bacteria	Bacteria	Bacteria	Bacteria	Bacteria	Bacteria	Bacteria	Bacteria	Bacteria
Phylum	Firmicutes	Firmicutes	Firmicutes	Firmicutes	Firmicutes	Firmicutes	Firmicutes	Firmicutes	Firmicutes
Class	Clostridia	Clostridia	Clostridia	Clostridia	Clostridia	Clostridia	Clostridia	Clostridia	Clostridia
Order	Clostridiales	Clostridiales	Clostridiales	Clostridiales	Clostridiales	Clostridiales	Clostridiales	Halanaerobiales	Clostridiales
Family	Lachnospiraceae	Lachnospiraceae	Ruminococcaceae	Lachnospiraceae	Lachnospiraceae	Lachnospiraceae	Lachnospiraceae	Halanaerobiaceae	Ruminococcaceae
Genus	Acetatifactor	Clostridium_XlVa	Butyricicoccus	Clostridium_XlVa	Clostridium_XlVa	Lachnospira	Clostridium_XlVa	Halanaerobium	Clostridium_IV
#NAME	ZOTU_0181	ZOTU_0182	ZOTU_0183	ZOTU_0184	ZOTU_0185	ZOTU_0186	ZOTU_0187	ZOTU_0188	ZOTU_0189
HFFG_45	2	0	5	0	0	7	1	0	0
HFFG_46	1	0	2	0	0	0	0	0	1
HFFG_47	0	0	0	0	0	0	0	0	0
HFFG_61	0	0	2	0	0	0	0	0	2
HFFG_62	12	0	10	0	0	13	6	0	5
HFFG_63	1	1	6	0	0	2	0	0	0
HFFG_64	1	0	5	0	0	4	0	0	0
HFFG_76	6	0	15	0	0	22	7	0	10

Kingdom	Bacteria	Bacteria	Bacteria	Bacteria	Bacteria	Bacteria	Bacteria	Bacteria	Bacteria
Phylum	Actinobacteria	Firmicutes	Firmicutes	Firmicutes	Firmicutes	Firmicutes	Firmicutes	Firmicutes	Firmicutes
Class	Actinobacteria	Clostridia	Clostridia	Clostridia	Clostridia	Clostridia	Clostridia	Clostridia	Clostridia
Order	Actinomycetales	Clostridiales	Clostridiales	Clostridiales	Clostridiales	Clostridiales	Clostridiales	Clostridiales	Clostridiales
Family	Streptomycetaceae	Lachnospiraceae	Lachnospiraceae	Lachnospiraceae	Ruminococcaceae	Ruminococcaceae	Ruminococcaceae	Lachnospiraceae	Lachnospiraceae
Genus	Streptomyces	Ruminococcus	Roseburia	Clostridium_XlVa	Clostridium_III	Oscillibacter	Anaerotruncus	Clostridium_XlVa	Clostridium_XlVa
#NAME	ZOTU_0190	ZOTU_0191	ZOTU_0192	ZOTU_0193	ZOTU_0194	ZOTU_0195	ZOTU_0196	ZOTU_0197	ZOTU_0198
CD_49	3	2	0	6	0	1	7	0	0
CD_50	0	1	1	0	0	2	1	0	0
CD_51	0	0	1	0	0	6	17	0	6
CD_52	0	0	0	0	0	0	3	0	1
CD_65	0	0	0	0	0	0	2	0	0
CD_66	0	0	0	0	0	0	1	0	0
CD_67	0	0	1	0	0	1	1	0	0
CD_68	0	0	2	0	0	0	4	0	1
CD_78	0	0	0	0	0	0	0	0	0
CD_79	0	0	1	0	0	2	0	0	0
CD_80	0	0	0	0	0	0	0	0	0
CDFG_54	0	0	0	0	0	1	1	0	0
CDFG_55	0	0	0	0	7	5	0	0	3
CDFG_56	1	0	0	0	0	2	0	0	0
CDFG_70	1	0	0	0	0	0	0	0	1
CDFG_71	0	0	0	0	0	0	0	0	0
HF_41	0	0	1	0	0	0	1	0	2
HF_42	0	0	0	0	0	0	0	0	4
HF_43	0	0	0	0	4	1	1	0	1
HF_44	0	0	1	0	0	7	2	0	2
HF_57	19	3	7	8	0	0	2	0	0
HF_58	37	95	4	36	0	2	10	0	2
HF_59	17	6	3	55	0	7	3	0	2
HF_60	1	2	1	8	0	2	2	0	0
HF_73	2	0	2	1	0	5	0	0	2
HF_74	2	12	0	6	0	6	1	0	0
HF_75	0	1	2	0	4	3	0	0	2

Kingdom	Bacteria	Bacteria	Bacteria	Bacteria	Bacteria	Bacteria	Bacteria	Bacteria	Bacteria
Phylum	Actinobacteria	Firmicutes	Firmicutes	Firmicutes	Firmicutes	Firmicutes	Firmicutes	Firmicutes	Firmicutes
Class	Actinobacteria	Clostridia	Clostridia	Clostridia	Clostridia	Clostridia	Clostridia	Clostridia	Clostridia
Order	Actinomycetales	Clostridiales	Clostridiales	Clostridiales	Clostridiales	Clostridiales	Clostridiales	Clostridiales	Clostridiales
Family	Streptomycetaceae	Lachnospiraceae	Lachnospiraceae	Lachnospiraceae	Ruminococcaceae	Ruminococcaceae	Ruminococcaceae	Lachnospiraceae	Lachnospiraceae
Genus	Streptomyces	Ruminococcus	Roseburia	Clostridium_XlVa	Clostridium_III	Oscillibacter	Anaerotruncus	Clostridium_XlVa	Clostridium_XlVa
#NAME	ZOTU_0190	ZOTU_0191	ZOTU_0192	ZOTU_0193	ZOTU_0194	ZOTU_0195	ZOTU_0196	ZOTU_0197	ZOTU_0198
HFFG_45	0	0	4	0	0	18	1	1	1
HFFG_46	0	0	1	0	0	0	1	0	0
HFFG_47	0	0	1	0	0	2	1	3	1
HFFG_61	0	0	0	0	0	1	1	0	0
HFFG_62	7	0	0	0	0	6	8	0	0
HFFG_63	3	1	1	0	0	3	0	0	0
HFFG_64	0	0	1	0	0	0	0	0	0
HFFG_76	6	3	5	0	12	12	14	0	7

Kingdom	Bacteria	Bacteria	Bacteria	Bacteria	Bacteria	Bacteria	Bacteria	Bacteria	Bacteria
Phylum	Firmicutes	Firmicutes	Firmicutes	Firmicutes	Firmicutes	Firmicutes	Firmicutes	Firmicutes	Firmicutes
Class	Clostridia	Clostridia	Clostridia	Clostridia	Clostridia	Clostridia	Clostridia	Clostridia	Clostridia
Order	Clostridiales	Clostridiales	Clostridiales	Clostridiales	Clostridiales	Clostridiales	Clostridiales	Clostridiales	Clostridiales
Family	Ruminococcaceae	Lachnospiraceae	Lachnospiraceae	Lachnospiraceae	Lachnospiraceae	Ruminococcaceae	Lachnospiraceae	Lachnospiraceae	Ruminococcaceae
Genus	Intestinimonas	Clostridium_XlVa	Acetatifactor	Acetatifactor	Clostridium_XlVa	Anaerotruncus	Clostridium_XlVa	Clostridium_XlVa	Hydrogenoanaerobacterium
#NAME	ZOTU_0199	ZOTU_0200	ZOTU_0201	ZOTU_0202	ZOTU_0203	ZOTU_0204	ZOTU_0205	ZOTU_0206	ZOTU_0207
CD_49	0	1	0	0	0	6	0	1	2
CD_50	0	3	0	0	0	4	0	1	0
CD_51	13	4	1	0	7	23	0	4	0
CD_52	1	1	0	0	0	4	0	3	0
CD_65	1	0	0	0	0	2	0	1	3
CD_66	0	0	0	0	0	3	0	0	0
CD_67	1	1	0	0	0	2	0	0	1
CD_68	1	0	0	0	0	3	0	3	1
CD_78	0	0	0	0	0	10	0	0	0
CD_79	0	0	0	0	0	0	0	0	0
CD_80	0	0	0	0	0	1	0	0	0
CDFG_54	0	6	0	0	0	0	0	0	0
CDFG_55	7	4	0	0	0	1	0	2	0
CDFG_56	2	14	1	0	0	2	0	0	0
CDFG_70	0	4	0	0	0	0	0	0	0
CDFG_71	1	0	0	0	0	0	0	0	0
HF_41	0	0	0	1	5	0	0	0	0
HF_42	2	3	0	0	3	0	0	4	2
HF_43	0	0	0	0	0	0	0	0	1
HF_44	0	0	0	0	0	4	0	0	0
HF_57	1	0	0	0	0	0	0	0	5
HF_58	0	0	1	1	0	1	0	0	17
HF_59	0	0	1	0	0	3	0	0	12
HF_60	2	0	0	0	0	1	0	0	4
HF_73	0	0	0	0	0	0	0	1	2
HF_74	1	0	0	2	0	1	0	1	1
HF_75	4	1	0	0	4	0	0	0	4

Kingdom	Bacteria	Bacteria	Bacteria	Bacteria	Bacteria	Bacteria	Bacteria	Bacteria	Bacteria
Phylum	Firmicutes	Firmicutes	Firmicutes	Firmicutes	Firmicutes	Firmicutes	Firmicutes	Firmicutes	Firmicutes
Class	Clostridia	Clostridia	Clostridia	Clostridia	Clostridia	Clostridia	Clostridia	Clostridia	Clostridia
Order	Clostridiales	Clostridiales	Clostridiales	Clostridiales	Clostridiales	Clostridiales	Clostridiales	Clostridiales	Clostridiales
Family	Ruminococcaceae	Lachnospiraceae	Lachnospiraceae	Lachnospiraceae	Lachnospiraceae	Ruminococcaceae	Lachnospiraceae	Lachnospiraceae	Ruminococcaceae
Genus	Intestinimonas	Clostridium_XlVa	Acetatifactor	Acetatifactor	Clostridium_XlVa	Anaerotruncus	Clostridium_XlVa	Clostridium_XlVa	Hydrogenoanaerobacterium
#NAME	ZOTU_0199	ZOTU_0200	ZOTU_0201	ZOTU_0202	ZOTU_0203	ZOTU_0204	ZOTU_0205	ZOTU_0206	ZOTU_0207
HFFG_45	8	2	0	0	1	2	1	0	1
HFFG_46	1	0	0	0	0	0	0	0	1
HFFG_47	1	0	0	0	1	0	0	0	0
HFFG_61	0	0	0	0	0	0	0	0	2
HFFG_62	5	0	0	0	0	0	0	0	3
HFFG_63	0	0	0	0	0	0	0	0	2
HFFG_64	0	0	0	0	0	0	0	0	0
HFFG_76	7	0	0	0	1	2	0	0	13

Kingdom	Bacteria	Bacteria	Bacteria	Bacteria	Bacteria	Bacteria	Bacteria	Bacteria	Bacteria
Phylum	Firmicutes	Firmicutes	Firmicutes	Firmicutes	Firmicutes	Firmicutes	Firmicutes	Firmicutes	Firmicutes
Class	Clostridia	Clostridia	Clostridia	Clostridia	Clostridia	Clostridia	Clostridia	Clostridia	Clostridia
Order	Clostridiales	Clostridiales	Clostridiales	Clostridiales	Clostridiales	Clostridiales	Clostridiales	Clostridiales	Clostridiales
Family	Lachnospiraceae	Clostridiaceae_	Clostridiaceae_	Lachnospiraceae	Clostridiaceae_	Lachnospiraceae	Peptostreptococcaceae	Ruminococcaceae	Ruminococcaceae
Genus	Clostridium_XlVa	Alkaliphilus	Alkaliphilus	Clostridium_XlVa	Alkaliphilus	Acetatifactor	Filifactor	Intestinimonas	Ruminococcus
#NAME	ZOTU_0208	ZOTU_0209	ZOTU_0210	ZOTU_0211	ZOTU_0212	ZOTU_0213	ZOTU_0214	ZOTU_0215	ZOTU_0216
CD_49	0	0	0	0	0	1	0	2	3
CD_50	2	1	0	0	2	1	0	4	0
CD_51	1	3	0	1	0	0	0	14	3
CD_52	0	3	0	0	13	0	0	0	0
CD_65	1	2	0	0	0	0	0	0	0
CD_66	0	0	0	0	3	0	0	1	0
CD_67	0	1	0	0	4	0	0	0	1
CD_68	7	2	0	0	0	2	0	0	0
CD_78	0	4	0	0	0	0	0	2	1
CD_79	0	0	0	0	0	0	0	1	0
CD_80	0	0	0	0	2	0	0	0	0
CDFG_54	0	0	0	2	0	4	0	3	1
CDFG_55	2	1	0	4	1	12	0	4	1
CDFG_56	3	0	0	9	0	13	0	6	4
CDFG_70	1	0	1	0	0	1	0	2	1
CDFG_71	0	0	1	0	0	0	0	3	0
HF_41	0	0	0	0	1	0	0	1	0
HF_42	1	0	0	0	0	0	0	0	0
HF_43	2	0	0	0	0	1	0	4	0
HF_44	5	0	5	0	0	2	0	7	0
HF_57	1	0	0	0	0	0	0	0	0
HF_58	3	0	0	0	0	0	0	0	0
HF_59	0	0	0	0	0	0	0	0	0
HF_60	0	0	0	0	0	0	0	1	1
HF_73	0	0	0	1	0	2	0	1	0
HF_74	1	0	0	0	0	2	0	1	4
HF_75	0	0	0	0	0	1	0	0	6

Kingdom	Bacteria	Bacteria	Bacteria	Bacteria	Bacteria	Bacteria	Bacteria	Bacteria	Bacteria
Phylum	Firmicutes	Firmicutes	Firmicutes	Firmicutes	Firmicutes	Firmicutes	Firmicutes	Firmicutes	Firmicutes
Class	Clostridia	Clostridia	Clostridia	Clostridia	Clostridia	Clostridia	Clostridia	Clostridia	Clostridia
Order	Clostridiales	Clostridiales	Clostridiales	Clostridiales	Clostridiales	Clostridiales	Clostridiales	Clostridiales	Clostridiales
Family	Lachnospiraceae	Clostridiaceae_	Clostridiaceae_	Lachnospiraceae	Clostridiaceae_	Lachnospiraceae	Peptostreptococcaceae	Ruminococcaceae	Ruminococcaceae
Genus	Clostridium_XlVa	Alkaliphilus	Alkaliphilus	Clostridium_XlVa	Alkaliphilus	Acetatifactor	Filifactor	Intestinimonas	Ruminococcus
#NAME	ZOTU_0208	ZOTU_0209	ZOTU_0210	ZOTU_0211	ZOTU_0212	ZOTU_0213	ZOTU_0214	ZOTU_0215	ZOTU_0216
HFFG_45	0	0	0	0	0	2	0	7	6
HFFG_46	0	0	0	0	0	1	0	0	1
HFFG_47	0	0	0	0	0	0	0	0	0
HFFG_61	0	0	0	0	0	0	0	1	0
HFFG_62	1	0	0	1	0	2	0	2	1
HFFG_63	0	0	0	0	0	0	0	0	0
HFFG_64	0	0	0	2	0	0	0	0	0
HFFG_76	0	0	0	5	0	2	0	5	53

Kingdom	Bacteria	Bacteria	Bacteria	Bacteria	Bacteria	Bacteria	Bacteria	Bacteria
Phylum	Firmicutes	Firmicutes	Firmicutes	Bacteroidetes	Firmicutes	Actinobacteria	Firmicutes	Firmicutes
Class	Clostridia	Clostridia	Clostridia	Bacteroidia	Clostridia	Actinobacteria	Clostridia	Clostridia
Order	Clostridiales	Clostridiales	Clostridiales	Bacteroidales	Clostridiales	Coriobacteriales	Clostridiales	Clostridiales
Family	Lachnospiraceae	Ruminococcaceae	Lachnospiraceae	Porphyromonadaceae	Ruminococcaceae	Coriobacteriaceae	Ruminococcaceae	Ruminococcaceae
Genus	Lachnospiracea_incertae_sedis	Sporobacter	Acetatifactor	Barnesiella	Sporobacter	Eggerthella	Anaerotruncus	Clostridium_IV
#NAME	ZOTU_0217	ZOTU_0218	ZOTU_0219	ZOTU_0220	ZOTU_0221	ZOTU_0222	ZOTU_0223	ZOTU_0224
CD_49	0	0	0	0	1	0	2	0
CD_50	0	0	7	0	0	1	0	0
CD_51	0	0	6	0	0	1	2	0
CD_52	0	0	0	0	0	0	0	0
CD_65	3	0	0	0	0	0	0	1
CD_66	0	0	0	0	0	0	0	0
CD_67	0	0	0	0	0	0	0	0
CD_68	8	0	0	0	2	0	0	0
CD_78	0	0	0	0	0	0	0	0
CD_79	0	0	0	0	0	0	0	0
CD_80	0	0	0	0	0	0	0	0
CDFG_54	0	0	0	0	0	0	5	0
CDFG_55	0	0	0	0	0	0	4	1
CDFG_56	0	0	0	0	0	0	4	1
CDFG_70	0	0	0	0	0	0	0	0
CDFG_71	1	0	1	0	0	0	0	0
HF_41	0	1	0	0	0	0	0	0
HF_42	0	1	0	0	0	0	0	0
HF_43	0	0	0	0	0	0	0	0
HF_44	0	0	0	0	0	0	1	0
HF_57	0	0	0	0	4	0	3	2
HF_58	0	0	1	0	13	0	8	12
HF_59	0	0	0	0	2	0	4	10
HF_60	0	1	1	0	2	0	6	3
HF_73	0	0	1	0	0	0	2	0
HF_74	0	0	0	0	0	0	2	1
HF_75	0	1	2	0	2	1	2	1

Kingdom	Bacteria	Bacteria	Bacteria	Bacteria	Bacteria	Bacteria	Bacteria	Bacteria
Phylum	Firmicutes	Firmicutes	Firmicutes	Bacteroidetes	Firmicutes	Actinobacteria	Firmicutes	Firmicutes
Class	Clostridia	Clostridia	Clostridia	Bacteroidia	Clostridia	Actinobacteria	Clostridia	Clostridia
Order	Clostridiales	Clostridiales	Clostridiales	Bacteroidales	Clostridiales	Coriobacteriales	Clostridiales	Clostridiales
Family	Lachnospiraceae	Ruminococcaceae	Lachnospiraceae	Porphyromonadaceae	Ruminococcaceae	Coriobacteriaceae	Ruminococcaceae	Ruminococcaceae
Genus	Lachnospiracea_incertae_sedis	Sporobacter	Acetatifactor	Barnesiella	Sporobacter	Eggerthella	Anaerotruncus	Clostridium_IV
#NAME	ZOTU_0217	ZOTU_0218	ZOTU_0219	ZOTU_0220	ZOTU_0221	ZOTU_0222	ZOTU_0223	ZOTU_0224
HFFG_45	0	0	1	0	1	0	5	0
HFFG_46	0	0	1	0	0	0	1	1
HFFG_47	0	0	1	0	0	0	0	2
HFFG_61	2	0	0	0	0	0	0	1
HFFG_62	29	0	1	0	3	3	14	8
HFFG_63	1	0	0	0	2	2	1	4
HFFG_64	1	0	0	0	1	0	0	1
HFFG_76	0	0	4	0	15	2	8	6

Kingdom	Bacteria	Bacteria	Bacteria	Bacteria	Bacteria	Bacteria	Bacteria	Bacteria	Bacteria
Phylum	Firmicutes	Firmicutes	Firmicutes	Firmicutes	Firmicutes	Bacteroidetes	Firmicutes	Firmicutes	Firmicutes
Class	Clostridia	Clostridia	Clostridia	Clostridia	Clostridia	Bacteroidia	Clostridia	Clostridia	Clostridia
Order	Clostridiales	Clostridiales	Clostridiales	Clostridiales	Clostridiales	Bacteroidales	Clostridiales	Clostridiales	Clostridiales
Family	Lachnospiraceae	Ruminococcaceae	Lachnospiraceae	Clostridiaceae_	Lachnospiraceae	Rikenellaceae	Ruminococcaceae	Lachnospiraceae	Lachnospiraceae
Genus	Clostridium_XlVa	Hydrogenoanaerobacterium	Acetatifactor	Alkaliphilus	Clostridium_XlVa	Alistipes	Acetanaerobacterium	Clostridium_XlVb	Clostridium_XlVa
#NAME	ZOTU_0225	ZOTU_0226	ZOTU_0227	ZOTU_0228	ZOTU_0229	ZOTU_0230	ZOTU_0231	ZOTU_0232	ZOTU_0233
CD_49	0	10	2	0	0	0	1	0	0
CD_50	0	13	1	0	0	0	0	0	0
CD_51	0	8	2	0	0	0	2	0	0
CD_52	0	2	0	0	0	0	0	0	0
CD_65	0	0	1	0	0	0	0	0	0
CD_66	0	0	0	0	0	0	0	0	0
CD_67	0	0	0	0	0	0	0	0	0
CD_68	0	1	3	0	0	0	1	0	0
CD_78	0	0	0	0	0	0	0	0	0
CD_79	0	0	0	0	0	0	0	0	0
CD_80	0	0	0	0	0	0	0	0	0
CDFG_54	0	0	0	0	0	0	3	0	0
CDFG_55	0	2	0	0	0	0	11	1	0
CDFG_56	0	0	1	0	0	0	6	0	0
CDFG_70	0	0	0	0	0	0	0	0	0
CDFG_71	0	0	0	0	0	0	0	0	0
HF_41	0	1	0	0	3	4	0	0	0
HF_42	0	1	1	0	2	4	0	0	0
HF_43	0	0	0	0	0	4	1	0	0
HF_44	0	0	0	0	1	6	0	0	0
HF_57	0	3	5	1	1	0	0	0	0
HF_58	0	2	16	0	0	0	2	1	2
HF_59	0	0	9	0	0	0	0	0	0
HF_60	0	2	13	0	2	0	1	3	0
HF_73	0	0	2	0	0	4	0	0	0
HF_74	0	3	1	0	1	6	0	0	0
HF_75	0	1	1	0	0	0	4	1	0

Kingdom	Bacteria	Bacteria	Bacteria	Bacteria	Bacteria	Bacteria	Bacteria	Bacteria	Bacteria
Phylum	Firmicutes	Firmicutes	Firmicutes	Firmicutes	Firmicutes	Bacteroidetes	Firmicutes	Firmicutes	Firmicutes
Class	Clostridia	Clostridia	Clostridia	Clostridia	Clostridia	Bacteroidia	Clostridia	Clostridia	Clostridia
Order	Clostridiales	Clostridiales	Clostridiales	Clostridiales	Clostridiales	Bacteroidales	Clostridiales	Clostridiales	Clostridiales
Family	Lachnospiraceae	Ruminococcaceae	Lachnospiraceae	Clostridiaceae_	Lachnospiraceae	Rikenellaceae	Ruminococcaceae	Lachnospiraceae	Lachnospiraceae
Genus	Clostridium_XlVa	Hydrogenoanaerobacterium	Acetatifactor	Alkaliphilus	Clostridium_XlVa	Alistipes	Acetanaerobacterium	Clostridium_XlVb	Clostridium_XlVa
#NAME	ZOTU_0225	ZOTU_0226	ZOTU_0227	ZOTU_0228	ZOTU_0229	ZOTU_0230	ZOTU_0231	ZOTU_0232	ZOTU_0233
HFFG_45	0	3	1	0	0	0	0	2	0
HFFG_46	0	2	0	0	0	0	0	0	0
HFFG_47	0	0	0	0	1	0	0	0	0
HFFG_61	0	1	0	0	0	0	1	1	0
HFFG_62	0	12	3	0	0	0	1	2	0
HFFG_63	0	1	1	0	0	0	2	0	0
HFFG_64	0	0	0	0	0	0	2	0	2
HFFG_76	0	10	1	0	1	0	4	0	0

Kingdom	Bacteria	Bacteria	Bacteria	Bacteria	Bacteria	Bacteria	Bacteria	Bacteria	Bacteria
Phylum	Firmicutes	Actinobacteria	Firmicutes	Firmicutes	Firmicutes	Firmicutes	Firmicutes	Firmicutes	Firmicutes
Class	Clostridia	Actinobacteria	Clostridia	Clostridia	Clostridia	Clostridia	Clostridia	Clostridia	Clostridia
Order	Clostridiales	Actinomycetales	Clostridiales	Clostridiales	Clostridiales	Clostridiales	Clostridiales	Clostridiales	Clostridiales
Family	Clostridiaceae_	Streptomycetaceae	Lachnospiraceae	Lachnospiraceae	Lachnospiraceae	Lachnospiraceae	Lachnospiraceae	Clostridiaceae_	Lachnospiraceae
Genus	Alkaliphilus	Streptomyces	Clostridium_XlVa	Lachnospiracea_incertae_sedis	Clostridium_XlVa	Butyrivibrio	Clostridium_XlVa	Alkaliphilus	Clostridium_XlVa
#NAME	ZOTU_0234	ZOTU_0235	ZOTU_0236	ZOTU_0237	ZOTU_0238	ZOTU_0239	ZOTU_0240	ZOTU_0241	ZOTU_0242
CD_49	0	0	0	0	0	0	0	0	2
CD_50	0	0	0	0	0	0	0	0	0
CD_51	0	0	4	0	0	2	2	0	1
CD_52	0	0	0	0	0	0	0	0	2
CD_65	0	0	0	0	0	0	0	0	0
CD_66	0	0	0	0	0	0	0	0	1
CD_67	0	0	0	0	0	1	0	0	0
CD_68	0	0	0	0	0	0	0	0	0
CD_78	0	0	0	0	0	1	0	0	0
CD_79	0	0	0	0	0	0	0	0	0
CD_80	0	0	0	0	0	0	0	0	0
CDFG_54	0	0	1	0	0	0	0	0	0
CDFG_55	0	0	1	3	0	0	0	0	0
CDFG_56	0	0	1	8	0	1	0	0	1
CDFG_70	0	1	0	1	0	0	0	0	0
CDFG_71	0	0	0	0	0	0	0	0	0
HF_41	1	0	0	0	0	0	0	0	0
HF_42	0	0	0	0	0	0	0	0	3
HF_43	0	0	0	2	0	0	0	0	0
HF_44	0	0	0	0	0	0	0	2	2
HF_57	0	0	0	0	1	0	0	0	0
HF_58	0	0	0	10	1	9	0	0	0
HF_59	0	0	0	6	1	3	0	0	0
HF_60	0	0	0	0	0	3	0	0	0
HF_73	0	1	1	3	0	0	0	0	0
HF_74	0	7	0	6	0	0	0	0	1
HF_75	0	0	0	5	1	0	0	0	1

Kingdom	Bacteria	Bacteria	Bacteria	Bacteria	Bacteria	Bacteria	Bacteria	Bacteria	Bacteria
Phylum	Firmicutes	Actinobacteria	Firmicutes	Firmicutes	Firmicutes	Firmicutes	Firmicutes	Firmicutes	Firmicutes
Class	Clostridia	Actinobacteria	Clostridia	Clostridia	Clostridia	Clostridia	Clostridia	Clostridia	Clostridia
Order	Clostridiales	Actinomycetales	Clostridiales	Clostridiales	Clostridiales	Clostridiales	Clostridiales	Clostridiales	Clostridiales
Family	Clostridiaceae_	Streptomycetaceae	Lachnospiraceae	Lachnospiraceae	Lachnospiraceae	Lachnospiraceae	Lachnospiraceae	Clostridiaceae_	Lachnospiraceae
Genus	Alkaliphilus	Streptomyces	Clostridium_XlVa	Lachnospiracea_incertae_sedis	Clostridium_XlVa	Butyrivibrio	Clostridium_XlVa	Alkaliphilus	Clostridium_XlVa
#NAME	ZOTU_0234	ZOTU_0235	ZOTU_0236	ZOTU_0237	ZOTU_0238	ZOTU_0239	ZOTU_0240	ZOTU_0241	ZOTU_0242
HFFG_45	0	0	3	6	1	0	6	0	2
HFFG_46	0	0	0	0	0	0	3	0	0
HFFG_47	0	0	0	0	0	0	0	0	0
HFFG_61	0	0	1	1	1	1	0	0	0
HFFG_62	0	9	0	9	0	0	0	0	1
HFFG_63	0	0	0	2	1	0	0	0	0
HFFG_64	0	0	0	2	2	0	0	0	0
HFFG_76	0	0	3	14	0	7	1	0	3

Kingdom	Bacteria	Bacteria	Bacteria	Bacteria	Bacteria	Bacteria	Bacteria
Phylum	Actinobacteria	Firmicutes	Firmicutes	Firmicutes	Firmicutes	Firmicutes	Firmicutes
Class	Actinobacteria	Clostridia	Clostridia	Clostridia	Erysipelotrichia	Clostridia	Clostridia
Order	Coriobacteriales	Clostridiales	Clostridiales	Clostridiales	Erysipelotrichales	Clostridiales	Clostridiales
Family	Coriobacteriaceae	Lachnospiraceae	Lachnospiraceae	Ruminococcaceae	Erysipelotrichaceae	Lachnospiraceae	Lachnospiraceae
Genus	Enterorhabdus	Lachnospiracea_incertae_sedis	Lachnospiracea_incertae_sedis	Clostridium_IV	Catenisphaera	Clostridium_XlVa	Roseburia
#NAME	ZOTU_0243	ZOTU_0244	ZOTU_0245	ZOTU_0246	ZOTU_0247	ZOTU_0248	ZOTU_0249
CD_49	1	0	3	1	0	0	0
CD_50	0	0	2	2	0	0	0
CD_51	0	1	7	1	0	0	0
CD_52	0	0	0	0	1	1	0
CD_65	0	0	0	1	0	0	0
CD_66	1	0	0	0	1	0	0
CD_67	0	0	0	0	0	0	0
CD_68	0	0	0	0	0	0	0
CD_78	0	0	0	0	0	0	0
CD_79	0	0	0	0	0	0	0
CD_80	0	0	0	0	0	0	0
CDFG_54	1	1	0	0	2	0	0
CDFG_55	0	3	0	5	6	2	0
CDFG_56	2	2	0	0	0	1	0
CDFG_70	0	0	0	1	0	1	0
CDFG_71	0	0	0	0	0	0	0
HF_41	0	0	0	0	0	0	0
HF_42	0	0	0	0	0	0	0
HF_43	1	0	0	0	0	0	0
HF_44	1	0	1	1	0	0	0
HF_57	1	0	1	1	0	0	0
HF_58	1	0	3	8	0	0	3
HF_59	0	0	6	2	0	0	0
HF_60	0	0	7	1	0	0	0
HF_73	1	0	2	0	0	0	0
HF_74	0	0	0	0	0	1	0
HF_75	0	0	0	0	0	0	0

Kingdom	Bacteria	Bacteria	Bacteria	Bacteria	Bacteria	Bacteria	Bacteria
Phylum	Actinobacteria	Firmicutes	Firmicutes	Firmicutes	Firmicutes	Firmicutes	Firmicutes
Class	Actinobacteria	Clostridia	Clostridia	Clostridia	Erysipelotrichia	Clostridia	Clostridia
Order	Coriobacteriales	Clostridiales	Clostridiales	Clostridiales	Erysipelotrichales	Clostridiales	Clostridiales
Family	Coriobacteriaceae	Lachnospiraceae	Lachnospiraceae	Ruminococcaceae	Erysipelotrichaceae	Lachnospiraceae	Lachnospiraceae
Genus	Enterorhabdus	Lachnospiracea_incertae_sedis	Lachnospiracea_incertae_sedis	Clostridium_IV	Catenisphaera	Clostridium_XlVa	Roseburia
#NAME	ZOTU_0243	ZOTU_0244	ZOTU_0245	ZOTU_0246	ZOTU_0247	ZOTU_0248	ZOTU_0249
HFFG_45	0	2	1	4	0	1	0
HFFG_46	0	0	0	1	0	0	0
HFFG_47	0	0	0	1	0	0	0
HFFG_61	0	0	0	0	0	0	0
HFFG_62	1	1	1	0	1	0	0
HFFG_63	0	0	0	0	0	0	0
HFFG_64	0	0	0	0	0	0	0
HFFG_76	1	1	1	4	0	2	0

Kingdom	Bacteria	Bacteria	Bacteria	Bacteria	Bacteria	Bacteria	Bacteria	Bacteria	Bacteria
Phylum	Firmicutes	Firmicutes	Firmicutes	Firmicutes	Firmicutes	Firmicutes	Firmicutes	Firmicutes	Firmicutes
Class	Clostridia	Clostridia	Clostridia	Bacilli	Clostridia	Clostridia	Clostridia	Clostridia	Clostridia
Order	Clostridiales	Clostridiales	Clostridiales	Bacillales	Clostridiales	Clostridiales	Clostridiales	Clostridiales	Clostridiales
Family	Lachnospiraceae	Peptostreptococcaceae	Lachnospiraceae	Staphylococcaceae	Lachnospiraceae	Lachnospiraceae	Lachnospiraceae	Lachnospiraceae	Lachnospiraceae
Genus	Marvinbryantia	Filifactor	Acetatifactor	Staphylococcus	Clostridium_XlVa	Clostridium_XlVa	Butyrivibrio	Clostridium_XlVa	Clostridium_XlVb
#NAME	ZOTU_0250	ZOTU_0251	ZOTU_0252	ZOTU_0253	ZOTU_0254	ZOTU_0255	ZOTU_0256	ZOTU_0257	ZOTU_0258
CD_49	0	5	0	2	0	0	0	0	0
CD_50	1	0	0	0	1	0	2	0	0
CD_51	0	1	5	53	3	0	2	0	17
CD_52	2	0	0	0	0	0	0	1	0
CD_65	0	0	0	0	0	0	1	0	0
CD_66	0	0	0	0	0	0	0	0	0
CD_67	0	0	0	1	1	0	0	0	0
CD_68	0	0	1	1	0	0	0	0	1
CD_78	0	0	2	1	0	0	0	0	1
CD_79	0	0	0	0	0	0	0	0	0
CD_80	0	0	0	0	0	0	0	0	0
CDFG_54	0	0	1	0	6	1	0	0	0
CDFG_55	0	0	0	2	3	0	1	0	0
CDFG_56	0	0	1	8	8	0	0	0	0
CDFG_70	1	0	2	1	0	0	0	0	1
CDFG_71	0	0	0	0	2	0	0	0	0
HF_41	0	0	4	1	0	0	0	0	0
HF_42	0	0	0	14	1	0	0	0	0
HF_43	0	0	4	0	0	0	1	0	0
HF_44	0	0	8	0	0	0	2	0	0
HF_57	0	0	0	5	0	0	4	0	0
HF_58	0	0	0	17	0	0	11	2	0
HF_59	0	0	0	0	0	0	1	0	0
HF_60	0	0	0	0	0	0	0	0	1
HF_73	0	0	0	2	1	0	2	0	0
HF_74	1	0	0	0	3	0	5	0	0
HF_75	0	0	0	0	0	0	0	0	0

Kingdom	Bacteria	Bacteria	Bacteria	Bacteria	Bacteria	Bacteria	Bacteria	Bacteria	Bacteria
Phylum	Firmicutes	Firmicutes	Firmicutes	Firmicutes	Firmicutes	Firmicutes	Firmicutes	Firmicutes	Firmicutes
Class	Clostridia	Clostridia	Clostridia	Bacilli	Clostridia	Clostridia	Clostridia	Clostridia	Clostridia
Order	Clostridiales	Clostridiales	Clostridiales	Bacillales	Clostridiales	Clostridiales	Clostridiales	Clostridiales	Clostridiales
Family	Lachnospiraceae	Peptostreptococcaceae	Lachnospiraceae	Staphylococcaceae	Lachnospiraceae	Lachnospiraceae	Lachnospiraceae	Lachnospiraceae	Lachnospiraceae
Genus	Marvinbryantia	Filifactor	Acetatifactor	Staphylococcus	Clostridium_XlVa	Clostridium_XlVa	Butyrivibrio	Clostridium_XlVa	Clostridium_XlVb
#NAME	ZOTU_0250	ZOTU_0251	ZOTU_0252	ZOTU_0253	ZOTU_0254	ZOTU_0255	ZOTU_0256	ZOTU_0257	ZOTU_0258
HFFG_45	7	0	3	0	5	0	0	0	0
HFFG_46	0	0	0	1	1	0	0	0	0
HFFG_47	0	0	0	1	0	0	0	0	1
HFFG_61	1	0	0	1	3	0	0	0	0
HFFG_62	3	0	0	5	5	0	0	0	0
HFFG_63	1	0	0	1	0	0	0	0	0
HFFG_64	2	0	0	0	1	0	0	0	0
HFFG_76	1	0	1	6	1	0	0	0	2

Kingdom	Bacteria	Bacteria	Bacteria	Bacteria	Bacteria	Bacteria	Bacteria	Bacteria	Bacteria
Phylum	Firmicutes	Firmicutes	Tenericutes	Firmicutes	Tenericutes	Firmicutes	Firmicutes	Tenericutes	Firmicutes
Class	Clostridia	Clostridia	Mollicutes	Clostridia	Mollicutes	Clostridia	Clostridia	Mollicutes	Clostridia
Order	Clostridiales	Clostridiales	Acholeplasmatales	Clostridiales	Acholeplasmatales	Clostridiales	Clostridiales	Entomoplasmatales	Clostridiales
Family	Lachnospiraceae	Lachnospiraceae	Acholeplasmataceae	Lachnospiraceae	Acholeplasmataceae	Clostridiaceae_	Lachnospiraceae	Spiroplasmataceae	Ruminococcaceae
Genus	Clostridium_XlVa	Clostridium_XlVa	Acholeplasma	Clostridium_XlVa	Acholeplasma	Geosporobacter	Clostridium_XlVa	Spiroplasma	Butyricicoccus
#NAME	ZOTU_0259	ZOTU_0260	ZOTU_0261	ZOTU_0262	ZOTU_0263	ZOTU_0264	ZOTU_0265	ZOTU_0266	ZOTU_0267
CD_49	0	0	2	0	0	0	2	0	0
CD_50	0	0	0	0	0	0	1	0	0
CD_51	0	0	0	0	16	0	2	0	0
CD_52	0	0	1	0	0	0	0	0	0
CD_65	0	0	0	0	0	0	0	0	0
CD_66	0	0	0	0	0	0	0	0	0
CD_67	0	0	0	0	0	0	0	0	0
CD_68	0	0	0	0	0	0	2	0	0
CD_78	0	0	0	0	4	0	0	0	0
CD_79	0	0	1	0	0	0	0	0	0
CD_80	0	0	0	0	1	0	0	0	0
CDFG_54	0	0	0	0	1	0	0	0	0
CDFG_55	1	0	0	0	0	0	0	0	0
CDFG_56	0	0	0	0	0	0	0	0	1
CDFG_70	0	0	1	0	2	0	0	0	0
CDFG_71	0	0	0	0	1	0	0	0	0
HF_41	0	0	0	0	0	0	1	0	0
HF_42	4	0	0	0	0	0	0	2	0
HF_43	2	0	0	0	0	0	0	0	0
HF_44	12	0	0	0	0	0	0	0	0
HF_57	0	0	0	0	0	0	1	0	0
HF_58	0	0	0	0	0	0	1	0	1
HF_59	0	0	0	0	0	0	8	0	3
HF_60	0	0	0	0	0	0	3	0	2
HF_73	0	0	0	0	0	0	2	0	0
HF_74	1	0	0	8	0	0	4	0	0
HF_75	0	0	0	2	0	0	0	0	0

Kingdom	Bacteria	Bacteria	Bacteria	Bacteria	Bacteria	Bacteria	Bacteria	Bacteria	Bacteria
Phylum	Firmicutes	Firmicutes	Tenericutes	Firmicutes	Tenericutes	Firmicutes	Firmicutes	Tenericutes	Firmicutes
Class	Clostridia	Clostridia	Mollicutes	Clostridia	Mollicutes	Clostridia	Clostridia	Mollicutes	Clostridia
Order	Clostridiales	Clostridiales	Acholeplasmatales	Clostridiales	Acholeplasmatales	Clostridiales	Clostridiales	Entomoplasmatales	Clostridiales
Family	Lachnospiraceae	Lachnospiraceae	Acholeplasmataceae	Lachnospiraceae	Acholeplasmataceae	Clostridiaceae_	Lachnospiraceae	Spiroplasmataceae	Ruminococcaceae
Genus	Clostridium_XlVa	Clostridium_XlVa	Acholeplasma	Clostridium_XlVa	Acholeplasma	Geosporobacter	Clostridium_XlVa	Spiroplasma	Butyricicoccus
#NAME	ZOTU_0259	ZOTU_0260	ZOTU_0261	ZOTU_0262	ZOTU_0263	ZOTU_0264	ZOTU_0265	ZOTU_0266	ZOTU_0267
HFFG_45	2	0	0	25	0	0	1	0	2
HFFG_46	0	0	0	1	0	0	0	0	0
HFFG_47	0	0	0	0	0	0	0	0	0
HFFG_61	0	0	0	0	0	0	0	0	0
HFFG_62	0	0	0	3	0	0	0	0	8
HFFG_63	0	0	0	2	0	0	0	0	1
HFFG_64	0	0	0	0	0	0	0	0	2
HFFG_76	1	0	1	10	0	0	0	0	6

Kingdom	Bacteria	Bacteria	Bacteria	Bacteria	Bacteria	Bacteria	Bacteria	Bacteria	Bacteria
Phylum	Firmicutes	Firmicutes	Firmicutes	Firmicutes	Firmicutes	Firmicutes	Firmicutes	Firmicutes	Firmicutes
Class	Clostridia	Clostridia	Clostridia	Clostridia	Clostridia	Clostridia	Clostridia	Erysipelotrichia	Clostridia
Order	Clostridiales	Clostridiales	Clostridiales	Clostridiales	Clostridiales	Clostridiales	Clostridiales	Erysipelotrichales	Clostridiales
Family	Lachnospiraceae	Lachnospiraceae	Lachnospiraceae	Lachnospiraceae	Ruminococcaceae	Lachnospiraceae	Lachnospiraceae	Erysipelotrichaceae	Clostridiaceae_
Genus	Roseburia	Blautia	Clostridium_XlVa	Clostridium_XlVa	Clostridium_IV	Clostridium_XlVa	Clostridium_XlVa	Holdemania	Geosporobacter
#NAME	ZOTU_0268	ZOTU_0269	ZOTU_0270	ZOTU_0271	ZOTU_0272	ZOTU_0273	ZOTU_0274	ZOTU_0275	ZOTU_0276
CD_49	0	0	3	0	1	1	0	0	1
CD_50	0	0	1	0	0	0	1	0	0
CD_51	0	0	2	0	2	3	0	0	0
CD_52	0	0	0	0	2	1	0	0	0
CD_65	0	0	1	0	0	0	0	0	0
CD_66	0	0	0	0	0	0	0	0	0
CD_67	0	0	0	0	0	0	0	0	0
CD_68	0	0	0	1	0	0	1	0	0
CD_78	0	0	0	0	0	0	0	0	0
CD_79	0	0	0	0	0	0	0	0	0
CD_80	0	0	0	0	0	0	0	0	0
CDFG_54	0	0	0	0	0	1	1	2	0
CDFG_55	1	0	1	0	1	1	2	4	0
CDFG_56	0	0	0	1	1	1	0	4	0
CDFG_70	0	0	0	0	1	3	0	0	0
CDFG_71	0	0	0	0	0	1	0	2	1
HF_41	0	0	0	0	0	0	0	0	0
HF_42	1	0	0	0	0	2	0	0	0
HF_43	0	0	0	0	0	0	0	0	0
HF_44	0	0	0	0	0	0	0	0	0
HF_57	0	0	4	0	0	0	0	0	0
HF_58	0	0	4	5	0	0	0	0	0
HF_59	4	0	1	1	0	0	0	0	0
HF_60	0	0	3	2	3	0	1	0	0
HF_73	0	0	1	0	0	0	1	0	0
HF_74	0	0	0	0	0	0	0	0	0
HF_75	0	0	0	0	0	0	0	0	0

Kingdom	Bacteria	Bacteria	Bacteria	Bacteria	Bacteria	Bacteria	Bacteria	Bacteria	Bacteria
Phylum	Firmicutes	Firmicutes	Firmicutes	Firmicutes	Firmicutes	Firmicutes	Firmicutes	Firmicutes	Firmicutes
Class	Clostridia	Clostridia	Clostridia	Clostridia	Clostridia	Clostridia	Clostridia	Erysipelotrichia	Clostridia
Order	Clostridiales	Clostridiales	Clostridiales	Clostridiales	Clostridiales	Clostridiales	Clostridiales	Erysipelotrichales	Clostridiales
Family	Lachnospiraceae	Lachnospiraceae	Lachnospiraceae	Lachnospiraceae	Ruminococcaceae	Lachnospiraceae	Lachnospiraceae	Erysipelotrichaceae	Clostridiaceae_
Genus	Roseburia	Blautia	Clostridium_XlVa	Clostridium_XlVa	Clostridium_IV	Clostridium_XlVa	Clostridium_XlVa	Holdemania	Geosporobacter
#NAME	ZOTU_0268	ZOTU_0269	ZOTU_0270	ZOTU_0271	ZOTU_0272	ZOTU_0273	ZOTU_0274	ZOTU_0275	ZOTU_0276
HFFG_45	1	0	1	0	1	0	0	1	0
HFFG_46	0	0	0	0	0	0	0	0	0
HFFG_47	0	0	0	0	1	0	0	0	0
HFFG_61	0	0	0	0	0	0	0	0	0
HFFG_62	5	0	3	0	0	0	0	0	0
HFFG_63	0	0	0	0	0	0	0	0	1
HFFG_64	0	0	0	0	1	0	0	0	0
HFFG_76	3	0	2	0	0	0	0	0	0

Kingdom	Bacteria	Bacteria	Bacteria	Bacteria	Bacteria	Bacteria	Bacteria
Phylum	Firmicutes	Cyanobacteria_Chloroplast	Firmicutes	Firmicutes	Firmicutes	Firmicutes	Firmicutes
Class	Clostridia	Chloroplast	Clostridia	Clostridia	Clostridia	Clostridia	Clostridia
Order	Clostridiales	Chloroplast	Clostridiales	Clostridiales	Clostridiales	Clostridiales	Clostridiales
Family	Ruminococcaceae	Streptophyta	Lachnospiraceae	Lachnospiraceae	Clostridiaceae_	Lachnospiraceae	Lachnospiraceae
Genus	Oscillibacter	NA	Clostridium_XlVa	Lachnospiracea_incertae_sedis	Clostridium_sensu_stricto	Clostridium_XlVa	Lachnospiracea_incertae_sedis
#NAME	ZOTU_0277	ZOTU_0278	ZOTU_0279	ZOTU_0280	ZOTU_0281	ZOTU_0282	ZOTU_0283
CD_49	2	0	0	0	0	0	0
CD_50	1	0	0	0	0	0	0
CD_51	7	0	0	0	0	0	0
CD_52	1	0	0	0	0	0	0
CD_65	0	0	0	0	0	0	0
CD_66	0	0	0	0	0	0	0
CD_67	0	0	0	0	0	0	0
CD_68	0	0	0	0	0	0	0
CD_78	0	0	0	0	0	0	0
CD_79	0	0	0	0	0	0	0
CD_80	0	0	0	0	0	0	0
CDFG_54	0	1	0	0	0	0	0
CDFG_55	1	0	0	0	0	0	0
CDFG_56	0	0	0	0	0	0	0
CDFG_70	0	0	0	0	0	0	0
CDFG_71	0	0	0	0	0	0	0
HF_41	0	0	0	0	0	0	0
HF_42	0	0	0	0	0	1	0
HF_43	0	0	0	0	0	1	0
HF_44	0	0	0	0	0	2	0
HF_57	0	0	0	0	0	2	0
HF_58	1	0	0	0	0	2	0
HF_59	4	0	0	0	0	1	0
HF_60	1	0	0	0	0	2	0
HF_73	1	0	0	0	0	1	0
HF_74	0	0	0	0	0	1	0
HF_75	0	0	0	0	0	1	0

Kingdom	Bacteria	Bacteria	Bacteria	Bacteria	Bacteria	Bacteria	Bacteria
Phylum	Firmicutes	Cyanobacteria_Chloroplast	Firmicutes	Firmicutes	Firmicutes	Firmicutes	Firmicutes
Class	Clostridia	Chloroplast	Clostridia	Clostridia	Clostridia	Clostridia	Clostridia
Order	Clostridiales	Chloroplast	Clostridiales	Clostridiales	Clostridiales	Clostridiales	Clostridiales
Family	Ruminococcaceae	Streptophyta	Lachnospiraceae	Lachnospiraceae	Clostridiaceae_	Lachnospiraceae	Lachnospiraceae
Genus	Oscillibacter	NA	Clostridium_XlVa	Lachnospiracea_incertae_sedis	Clostridium_sensu_stricto	Clostridium_XlVa	Lachnospiracea_incertae_sedis
#NAME	ZOTU_0277	ZOTU_0278	ZOTU_0279	ZOTU_0280	ZOTU_0281	ZOTU_0282	ZOTU_0283
HFFG_45	0	0	0	0	0	0	0
HFFG_46	0	0	0	0	0	0	0
HFFG_47	0	0	0	0	0	0	0
HFFG_61	0	0	0	0	0	0	0
HFFG_62	0	0	0	0	0	0	0
HFFG_63	0	0	0	0	0	0	1
HFFG_64	0	0	0	0	1	0	0
HFFG_76	2	0	0	0	0	0	0

Kingdom	Bacteria	Bacteria	Bacteria	Bacteria	Bacteria	Bacteria	Bacteria	Bacteria	Bacteria
Phylum	Tenericutes	Firmicutes	Firmicutes	Firmicutes	Firmicutes	Firmicutes	Firmicutes	Firmicutes	Firmicutes
Class	Mollicutes	Clostridia	Clostridia	Clostridia	Clostridia	Clostridia	Clostridia	Clostridia	Bacilli
Order	Entomoplasmatales	Clostridiales	Clostridiales	Clostridiales	Clostridiales	Clostridiales	Clostridiales	Halanaerobiales	Bacillales
Family	Spiroplasmataceae	Clostridiaceae_	Lachnospiraceae	Lachnospiraceae	Ruminococcaceae	Lachnospiraceae	Lachnospiraceae	Halanaerobiaceae	Bacillaceae_
Genus	Spiroplasma	Alkaliphilus	Clostridium_XlVa	Clostridium_XlVa	Intestinimonas	Clostridium_XlVa	Clostridium_XlVa	Halanaerobium	Bacillus
#NAME	ZOTU_0284	ZOTU_0285	ZOTU_0286	ZOTU_0287	ZOTU_0288	ZOTU_0289	ZOTU_0290	ZOTU_0291	ZOTU_0292
CD_49	0	0	1	0	0	0	0	0	0
CD_50	0	0	1	0	0	0	1	0	0
CD_51	1	0	3	0	2	0	0	0	0
CD_52	0	0	0	0	0	0	0	0	0
CD_65	0	0	3	0	1	0	0	0	0
CD_66	0	0	1	0	0	0	0	0	0
CD_67	0	0	0	0	0	0	0	0	0
CD_68	0	0	2	0	0	0	1	0	0
CD_78	0	0	0	0	0	0	0	0	0
CD_79	0	0	2	0	0	0	0	0	0
CD_80	1	0	0	0	0	0	0	0	0
CDFG_54	0	0	0	0	0	0	1	0	0
CDFG_55	0	0	0	3	3	1	0	0	0
CDFG_56	0	0	2	1	1	0	1	0	0
CDFG_70	0	10	0	0	0	0	0	0	0
CDFG_71	1	0	0	0	1	0	0	0	0
HF_41	0	0	0	0	0	1	0	0	0
HF_42	0	0	0	0	0	10	2	0	0
HF_43	0	0	0	0	0	0	0	0	0
HF_44	1	0	0	1	0	2	6	0	0
HF_57	0	0	1	0	0	0	1	0	0
HF_58	0	0	0	0	0	0	0	0	0
HF_59	0	0	0	0	0	0	0	0	0
HF_60	0	0	1	0	0	0	1	0	0
HF_73	0	0	2	0	0	0	0	0	0
HF_74	0	0	1	0	0	0	0	0	0
HF_75	0	0	0	0	0	0	1	0	0

Kingdom	Bacteria	Bacteria	Bacteria	Bacteria	Bacteria	Bacteria	Bacteria	Bacteria	Bacteria
Phylum	Tenericutes	Firmicutes	Firmicutes	Firmicutes	Firmicutes	Firmicutes	Firmicutes	Firmicutes	Firmicutes
Class	Mollicutes	Clostridia	Clostridia	Clostridia	Clostridia	Clostridia	Clostridia	Clostridia	Bacilli
Order	Entomoplasmatales	Clostridiales	Clostridiales	Clostridiales	Clostridiales	Clostridiales	Clostridiales	Halanaerobiales	Bacillales
Family	Spiroplasmataceae	Clostridiaceae_	Lachnospiraceae	Lachnospiraceae	Ruminococcaceae	Lachnospiraceae	Lachnospiraceae	Halanaerobiaceae	Bacillaceae_
Genus	Spiroplasma	Alkaliphilus	Clostridium_XlVa	Clostridium_XlVa	Intestinimonas	Clostridium_XlVa	Clostridium_XlVa	Halanaerobium	Bacillus
#NAME	ZOTU_0284	ZOTU_0285	ZOTU_0286	ZOTU_0287	ZOTU_0288	ZOTU_0289	ZOTU_0290	ZOTU_0291	ZOTU_0292
HFFG_45	0	0	2	0	1	0	0	0	0
HFFG_46	0	0	1	0	1	0	0	0	0
HFFG_47	0	0	1	0	0	0	0	0	0
HFFG_61	0	0	0	0	0	0	0	0	0
HFFG_62	0	0	0	0	0	0	1	0	0
HFFG_63	0	0	0	0	0	0	0	0	0
HFFG_64	0	0	0	0	0	0	0	0	0
HFFG_76	2	0	1	1	3	0	0	0	0

Kingdom	Bacteria	Bacteria	Bacteria	Bacteria	Bacteria	Bacteria	Bacteria	Bacteria	Bacteria
Phylum	Firmicutes	Firmicutes	Proteobacteria	Firmicutes	Firmicutes	Actinobacteria	Firmicutes	Firmicutes	Firmicutes
Class	Bacilli	Clostridia	Gammaproteobacteria	Clostridia	Clostridia	Actinobacteria	Clostridia	Clostridia	Clostridia
Order	Lactobacillales	Clostridiales	Pasteurellales	Clostridiales	Clostridiales	Actinomycetales	Clostridiales	Clostridiales	Clostridiales
Family	Streptococcaceae	Ruminococcaceae	Pasteurellaceae	Ruminococcaceae	Lachnospiraceae	Streptomycetaceae	Lachnospiraceae	Lachnospiraceae	Lachnospiraceae
Genus	Streptococcus	Clostridium_IV	Haemophilus	Sporobacter	Clostridium_XlVa	Streptomyces	Clostridium_XlVa	Blautia	Clostridium_XlVa
#NAME	ZOTU_0293	ZOTU_0294	ZOTU_0295	ZOTU_0296	ZOTU_0297	ZOTU_0298	ZOTU_0299	ZOTU_0300	ZOTU_0301
CD_49	0	0	0	0	0	2	0	0	0
CD_50	0	0	0	0	0	0	0	0	0
CD_51	0	0	0	0	0	0	0	2	2
CD_52	0	0	0	0	0	0	0	0	0
CD_65	0	1	0	0	0	0	0	1	0
CD_66	0	0	0	0	0	0	0	0	0
CD_67	0	0	0	0	0	0	0	0	0
CD_68	0	0	0	0	0	0	0	1	0
CD_78	0	0	0	0	0	0	0	0	0
CD_79	0	0	0	0	0	0	0	0	0
CD_80	0	0	0	0	0	0	0	0	0
CDFG_54	0	0	0	2	1	1	0	0	0
CDFG_55	0	0	0	0	0	0	0	0	0
CDFG_56	0	0	0	0	0	0	0	0	0
CDFG_70	0	0	0	0	0	0	0	0	0
CDFG_71	0	0	0	0	0	0	0	0	0
HF_41	0	0	0	0	0	0	0	2	0
HF_42	0	0	0	0	0	0	0	0	0
HF_43	0	0	0	0	0	0	0	0	0
HF_44	0	1	0	0	0	0	0	0	0
HF_57	0	2	2	4	2	8	0	0	0
HF_58	3	1	0	7	1	14	0	0	3
HF_59	0	0	0	1	6	10	0	5	1
HF_60	0	0	0	5	2	4	0	0	1
HF_73	0	0	0	0	0	0	0	2	0
HF_74	0	0	0	0	0	0	0	0	0
HF_75	0	0	0	0	1	0	0	0	0

Kingdom	Bacteria	Bacteria	Bacteria	Bacteria	Bacteria	Bacteria	Bacteria	Bacteria	Bacteria
Phylum	Firmicutes	Firmicutes	Proteobacteria	Firmicutes	Firmicutes	Actinobacteria	Firmicutes	Firmicutes	Firmicutes
Class	Bacilli	Clostridia	Gammaproteobacteria	Clostridia	Clostridia	Actinobacteria	Clostridia	Clostridia	Clostridia
Order	Lactobacillales	Clostridiales	Pasteurellales	Clostridiales	Clostridiales	Actinomycetales	Clostridiales	Clostridiales	Clostridiales
Family	Streptococcaceae	Ruminococcaceae	Pasteurellaceae	Ruminococcaceae	Lachnospiraceae	Streptomycetaceae	Lachnospiraceae	Lachnospiraceae	Lachnospiraceae
Genus	Streptococcus	Clostridium_IV	Haemophilus	Sporobacter	Clostridium_XlVa	Streptomyces	Clostridium_XlVa	Blautia	Clostridium_XlVa
#NAME	ZOTU_0293	ZOTU_0294	ZOTU_0295	ZOTU_0296	ZOTU_0297	ZOTU_0298	ZOTU_0299	ZOTU_0300	ZOTU_0301
HFFG_45	0	0	0	1	2	0	0	0	0
HFFG_46	0	0	0	0	0	0	0	0	0
HFFG_47	0	0	0	0	0	0	0	0	0
HFFG_61	0	0	0	0	1	0	0	0	0
HFFG_62	0	2	0	0	0	0	0	0	0
HFFG_63	0	1	0	0	1	2	0	0	0
HFFG_64	0	0	0	0	0	0	0	0	0
HFFG_76	0	1	0	0	2	1	0	0	0

Kingdom	Bacteria	Bacteria	Bacteria	Bacteria	Bacteria	Bacteria	Bacteria	Bacteria	Bacteria
Phylum	Firmicutes	Firmicutes	Firmicutes	Firmicutes	Tenericutes	Firmicutes	Firmicutes	Firmicutes	Firmicutes
Class	Clostridia	Bacilli	Bacilli	Clostridia	Mollicutes	Clostridia	Clostridia	Clostridia	Clostridia
Order	Clostridiales	Bacillales	Lactobacillales	Clostridiales	Acholeplasmatales	Clostridiales	Clostridiales	Clostridiales	Clostridiales
Family	Lachnospiraceae	Bacillaceae_	Enterococcaceae	Lachnospiraceae	Acholeplasmataceae	Ruminococcaceae	Lachnospiraceae	Ruminococcaceae	Ruminococcaceae
Genus	Acetatifactor	Bacillus	Enterococcus	Clostridium_XlVa	Acholeplasma	Anaerotruncus	Acetatifactor	Flavonifractor	Ethanoligenens
#NAME	ZOTU_0302	ZOTU_0303	ZOTU_0304	ZOTU_0305	ZOTU_0306	ZOTU_0307	ZOTU_0308	ZOTU_0309	ZOTU_0310
CD_49	0	0	8	2	0	2	0	0	1
CD_50	0	0	2	0	0	0	0	0	0
CD_51	0	0	0	1	0	1	0	2	0
CD_52	0	0	4	1	0	0	0	0	0
CD_65	0	0	1	0	0	0	0	0	0
CD_66	0	0	0	0	0	0	0	1	0
CD_67	0	0	0	0	0	0	0	0	0
CD_68	0	0	2	0	0	2	1	0	0
CD_78	0	0	0	0	0	0	0	0	0
CD_79	0	0	0	0	0	0	0	0	1
CD_80	0	0	0	0	0	0	0	0	0
CDFG_54	0	0	3	0	0	0	0	0	0
CDFG_55	0	0	0	2	0	0	1	5	0
CDFG_56	0	0	1	0	0	0	0	0	0
CDFG_70	0	0	0	0	0	0	0	0	0
CDFG_71	0	0	0	1	0	0	0	0	0
HF_41	1	0	0	0	0	0	0	0	0
HF_42	0	0	1	0	4	0	0	0	0
HF_43	2	0	4	0	0	0	0	0	0
HF_44	0	1	31	0	0	0	0	0	0
HF_57	0	0	2	0	0	1	0	0	0
HF_58	0	0	7	1	0	3	0	0	0
HF_59	0	0	0	0	0	2	2	0	0
HF_60	0	1	0	0	0	1	0	0	0
HF_73	0	0	28	0	0	0	0	0	0
HF_74	0	0	21	0	0	0	0	0	0
HF_75	0	0	0	0	0	1	0	0	1

Kingdom	Bacteria	Bacteria	Bacteria	Bacteria	Bacteria	Bacteria	Bacteria	Bacteria	Bacteria
Phylum	Firmicutes	Firmicutes	Firmicutes	Firmicutes	Tenericutes	Firmicutes	Firmicutes	Firmicutes	Firmicutes
Class	Clostridia	Bacilli	Bacilli	Clostridia	Mollicutes	Clostridia	Clostridia	Clostridia	Clostridia
Order	Clostridiales	Bacillales	Lactobacillales	Clostridiales	Acholeplasmatales	Clostridiales	Clostridiales	Clostridiales	Clostridiales
Family	Lachnospiraceae	Bacillaceae_	Enterococcaceae	Lachnospiraceae	Acholeplasmataceae	Ruminococcaceae	Lachnospiraceae	Ruminococcaceae	Ruminococcaceae
Genus	Acetatifactor	Bacillus	Enterococcus	Clostridium_XlVa	Acholeplasma	Anaerotruncus	Acetatifactor	Flavonifractor	Ethanoligenens
#NAME	ZOTU_0302	ZOTU_0303	ZOTU_0304	ZOTU_0305	ZOTU_0306	ZOTU_0307	ZOTU_0308	ZOTU_0309	ZOTU_0310
HFFG_45	1	0	7	1	0	1	0	0	0
HFFG_46	0	0	0	0	0	0	0	0	1
HFFG_47	0	0	2	0	0	0	0	0	0
HFFG_61	0	64	6	0	0	0	0	0	0
HFFG_62	0	2	23	0	0	0	0	0	0
HFFG_63	0	0	6	0	0	0	0	0	0
HFFG_64	0	0	3	0	0	0	0	0	0
HFFG_76	0	0	18	0	0	2	1	1	2

Kingdom	Bacteria	Bacteria	Bacteria	Bacteria	Bacteria	Bacteria	Bacteria	Bacteria
Phylum	Firmicutes	Firmicutes	Proteobacteria	Firmicutes	Firmicutes	Firmicutes	Firmicutes	Firmicutes
Class	Clostridia	Erysipelotrichia	Betaproteobacteria	Clostridia	Clostridia	Clostridia	Clostridia	Clostridia
Order	Clostridiales	Erysipelotrichales	Neisseriales	Clostridiales	Clostridiales	Clostridiales	Clostridiales	Clostridiales
Family	Lachnospiraceae	Erysipelotrichaceae	Neisseriaceae	Lachnospiraceae	Lachnospiraceae	Clostridiaceae_	Lachnospiraceae	Lachnospiraceae
Genus	Butyrivibrio	Holdemania	Neisseria	Ruminococcus	Lachnospiracea_incertae_sedis	Alkaliphilus	Clostridium_XlVa	Butyrivibrio
#NAME	ZOTU_0311	ZOTU_0312	ZOTU_0313	ZOTU_0314	ZOTU_0315	ZOTU_0316	ZOTU_0317	ZOTU_0318
CD_49	0	0	0	0	0	0	0	0
CD_50	0	0	0	0	0	0	0	0
CD_51	0	0	0	0	0	0	0	0
CD_52	0	0	0	0	0	0	0	0
CD_65	0	0	0	0	0	0	0	0
CD_66	0	0	0	0	0	0	0	0
CD_67	0	0	0	0	0	0	0	0
CD_68	0	0	0	0	0	0	0	0
CD_78	0	0	0	0	0	0	0	0
CD_79	0	0	0	0	0	0	0	0
CD_80	0	0	0	0	0	0	0	0
CDFG_54	0	0	0	0	0	0	0	0
CDFG_55	1	0	0	0	0	0	0	0
CDFG_56	0	0	0	4	0	0	0	0
CDFG_70	0	0	0	1	0	0	0	0
CDFG_71	0	0	0	0	0	0	0	0
HF_41	0	0	0	0	0	0	0	0
HF_42	1	1	0	0	0	0	0	0
HF_43	0	0	0	0	0	0	0	0
HF_44	0	0	0	0	0	0	0	0
HF_57	0	0	0	0	0	0	0	0
HF_58	0	0	0	0	0	0	0	0
HF_59	0	3	0	0	0	0	0	0
HF_60	0	4	0	0	0	0	0	0
HF_73	0	0	0	0	0	0	1	0
HF_74	0	0	0	0	0	0	0	0
HF_75	0	0	0	0	0	0	0	0

Kingdom	Bacteria	Bacteria	Bacteria	Bacteria	Bacteria	Bacteria	Bacteria	Bacteria
Phylum	Firmicutes	Firmicutes	Proteobacteria	Firmicutes	Firmicutes	Firmicutes	Firmicutes	Firmicutes
Class	Clostridia	Erysipelotrichia	Betaproteobacteria	Clostridia	Clostridia	Clostridia	Clostridia	Clostridia
Order	Clostridiales	Erysipelotrichales	Neisseriales	Clostridiales	Clostridiales	Clostridiales	Clostridiales	Clostridiales
Family	Lachnospiraceae	Erysipelotrichaceae	Neisseriaceae	Lachnospiraceae	Lachnospiraceae	Clostridiaceae_	Lachnospiraceae	Lachnospiraceae
Genus	Butyrivibrio	Holdemania	Neisseria	Ruminococcus	Lachnospiracea_incertae_sedis	Alkaliphilus	Clostridium_XlVa	Butyrivibrio
#NAME	ZOTU_0311	ZOTU_0312	ZOTU_0313	ZOTU_0314	ZOTU_0315	ZOTU_0316	ZOTU_0317	ZOTU_0318
HFFG_45	1	0	0	0	0	0	0	0
HFFG_46	0	0	0	0	0	0	0	0
HFFG_47	0	0	0	0	0	0	0	0
HFFG_61	0	0	0	0	0	0	0	0
HFFG_62	0	0	0	0	0	0	0	0
HFFG_63	0	0	0	0	0	0	0	0
HFFG_64	0	0	0	0	0	0	0	0
HFFG_76	0	0	0	0	0	0	0	0

Kingdom	Bacteria	Bacteria	Bacteria	Bacteria	Bacteria	Bacteria	Bacteria	Bacteria
Phylum	Firmicutes	Firmicutes	Firmicutes	Firmicutes	Firmicutes	Bacteroidetes	Firmicutes	Firmicutes
Class	Clostridia	Clostridia	Clostridia	Clostridia	Clostridia	Bacteroidia	Clostridia	Clostridia
Order	Clostridiales	Clostridiales	Clostridiales	Clostridiales	Clostridiales	Bacteroidales	Clostridiales	Clostridiales
Family	Clostridiaceae_	Lachnospiraceae	Lachnospiraceae	Clostridiaceae_	Lachnospiraceae	Porphyromonadaceae	Lachnospiraceae	Gracilibacteraceae
Genus	Clostridium_sensu_stricto	Butyrivibrio	Clostridium_XlVa	Clostridium_sensu_stricto	Clostridium_XlVa	Parabacteroides	Clostridium_XlVa	Lutispora
#NAME	ZOTU_0319	ZOTU_0320	ZOTU_0321	ZOTU_0322	ZOTU_0323	ZOTU_0324	ZOTU_0325	ZOTU_0326
CD_49	0	0	0	0	0	0	1	0
CD_50	0	0	0	0	0	0	0	0
CD_51	0	0	1	0	0	0	4	0
CD_52	0	0	0	0	0	0	0	0
CD_65	0	1	0	0	0	0	0	0
CD_66	0	0	0	0	0	0	0	0
CD_67	0	0	0	0	0	0	0	0
CD_68	0	0	0	0	0	0	0	0
CD_78	0	0	0	0	0	0	0	0
CD_79	0	0	0	0	0	0	0	0
CD_80	0	0	0	0	0	0	0	0
CDFG_54	0	0	0	0	0	0	0	0
CDFG_55	0	0	0	0	0	0	0	0
CDFG_56	0	0	0	0	0	0	1	0
CDFG_70	0	0	1	0	0	0	0	0
CDFG_71	0	0	0	0	0	0	0	0
HF_41	0	0	0	0	0	0	1	0
HF_42	0	0	0	0	0	0	0	0
HF_43	0	0	0	0	0	0	0	0
HF_44	0	0	0	0	0	0	1	0
HF_57	0	2	0	0	0	0	1	0
HF_58	0	3	0	0	0	0	0	0
HF_59	0	3	0	0	0	0	4	0
HF_60	0	0	0	0	0	0	0	0
HF_73	0	0	0	0	0	0	0	0
HF_74	0	0	0	0	0	0	2	0
HF_75	0	2	0	0	0	0	0	0

Kingdom	Bacteria	Bacteria	Bacteria	Bacteria	Bacteria	Bacteria	Bacteria	Bacteria
Phylum	Firmicutes	Firmicutes	Firmicutes	Firmicutes	Firmicutes	Bacteroidetes	Firmicutes	Firmicutes
Class	Clostridia	Clostridia	Clostridia	Clostridia	Clostridia	Bacteroidia	Clostridia	Clostridia
Order	Clostridiales	Clostridiales	Clostridiales	Clostridiales	Clostridiales	Bacteroidales	Clostridiales	Clostridiales
Family	Clostridiaceae_	Lachnospiraceae	Lachnospiraceae	Clostridiaceae_	Lachnospiraceae	Porphyromonadaceae	Lachnospiraceae	Gracilibacteraceae
Genus	Clostridium_sensu_stricto	Butyrivibrio	Clostridium_XlVa	Clostridium_sensu_stricto	Clostridium_XlVa	Parabacteroides	Clostridium_XlVa	Lutispora
#NAME	ZOTU_0319	ZOTU_0320	ZOTU_0321	ZOTU_0322	ZOTU_0323	ZOTU_0324	ZOTU_0325	ZOTU_0326
HFFG_45	0	0	0	0	0	2	0	0
HFFG_46	0	0	0	0	0	0	0	0
HFFG_47	0	0	0	0	0	2	0	0
HFFG_61	0	0	0	0	0	0	0	0
HFFG_62	0	0	0	0	0	0	1	0
HFFG_63	0	0	0	0	0	0	2	0
HFFG_64	0	0	0	1	0	0	0	0
HFFG_76	0	0	1	0	0	0	1	0

Kingdom	Bacteria	Bacteria	Bacteria	Bacteria	Bacteria	Bacteria	Bacteria	Bacteria
Phylum	Firmicutes	Firmicutes	Firmicutes	Firmicutes	Firmicutes	Firmicutes	Firmicutes	Bacteroidetes
Class	Clostridia	Clostridia	Clostridia	Clostridia	Clostridia	Clostridia	Clostridia	Bacteroidia
Order	Clostridiales	Clostridiales	Clostridiales	Clostridiales	Clostridiales	Clostridiales	Clostridiales	Bacteroidales
Family	Lachnospiraceae	Lachnospiraceae	Peptostreptococcaceae	Ruminococcaceae	Clostridiales_Incertae_Sedis_XI	Lachnospiraceae	Ruminococcaceae	Porphyromonadaceae
Genus	Clostridium_XlVa	Ruminococcus	Clostridium_XI	Clostridium_IV	Tissierella	Acetitomaculum	Oscillibacter	Barnesiella
#NAME	ZOTU_0327	ZOTU_0328	ZOTU_0329	ZOTU_0330	ZOTU_0331	ZOTU_0332	ZOTU_0333	ZOTU_0334
CD_49	0	0	0	1	0	0	0	0
CD_50	0	0	0	0	0	0	0	0
CD_51	0	0	0	1	0	0	1	0
CD_52	0	0	0	0	0	0	0	0
CD_65	0	0	0	0	0	0	0	0
CD_66	0	0	0	1	0	0	0	0
CD_67	0	0	0	0	0	0	0	0
CD_68	0	0	0	0	0	0	0	0
CD_78	0	0	0	0	0	0	0	0
CD_79	0	0	0	0	0	0	0	0
CD_80	0	0	0	0	0	0	0	0
CDFG_54	0	1	0	0	0	0	0	1
CDFG_55	0	0	0	1	0	0	0	1
CDFG_56	0	0	0	0	0	0	0	1
CDFG_70	0	0	0	0	0	0	0	0
CDFG_71	0	0	0	0	0	0	0	0
HF_41	0	0	0	0	0	0	0	0
HF_42	0	0	0	0	0	0	0	0
HF_43	0	0	0	1	0	0	0	0
HF_44	2	0	0	0	0	0	1	0
HF_57	0	0	0	0	0	0	0	0
HF_58	0	0	0	1	0	0	4	0
HF_59	0	0	0	3	0	0	0	0
HF_60	0	0	0	0	0	0	3	0
HF_73	0	0	0	0	0	0	0	0
HF_74	0	0	0	1	0	0	0	0
HF_75	0	0	0	1	0	0	1	0

Kingdom	Bacteria	Bacteria	Bacteria	Bacteria	Bacteria	Bacteria	Bacteria	Bacteria
Phylum	Firmicutes	Firmicutes	Firmicutes	Firmicutes	Firmicutes	Firmicutes	Firmicutes	Bacteroidetes
Class	Clostridia	Clostridia	Clostridia	Clostridia	Clostridia	Clostridia	Clostridia	Bacteroidia
Order	Clostridiales	Clostridiales	Clostridiales	Clostridiales	Clostridiales	Clostridiales	Clostridiales	Bacteroidales
Family	Lachnospiraceae	Lachnospiraceae	Peptostreptococcaceae	Ruminococcaceae	Clostridiales_Incertae_Sedis_XI	Lachnospiraceae	Ruminococcaceae	Porphyromonadaceae
Genus	Clostridium_XlVa	Ruminococcus	Clostridium_XI	Clostridium_IV	Tissierella	Acetitomaculum	Oscillibacter	Barnesiella
#NAME	ZOTU_0327	ZOTU_0328	ZOTU_0329	ZOTU_0330	ZOTU_0331	ZOTU_0332	ZOTU_0333	ZOTU_0334
HFFG_45	0	0	0	1	0	0	0	0
HFFG_46	0	0	0	0	0	0	0	0
HFFG_47	0	0	0	0	0	0	0	0
HFFG_61	0	0	0	0	0	0	1	0
HFFG_62	0	0	0	1	4	1	1	0
HFFG_63	0	0	0	0	0	0	0	0
HFFG_64	0	0	0	0	0	0	0	0
HFFG_76	0	0	0	0	0	3	0	0

Kingdom	Bacteria	Bacteria	Bacteria	Bacteria	Bacteria	Bacteria	Bacteria	Bacteria
Phylum	Firmicutes	Firmicutes	Bacteroidetes	Firmicutes	Firmicutes	Firmicutes	Firmicutes	Firmicutes
Class	Clostridia	Clostridia	Bacteroidia	Clostridia	Clostridia	Clostridia	Clostridia	Clostridia
Order	Clostridiales	Clostridiales	Bacteroidales	Clostridiales	Clostridiales	Clostridiales	Clostridiales	Clostridiales
Family	Ruminococcaceae	Lachnospiraceae	Porphyromonadaceae	Lachnospiraceae	Lachnospiraceae	Lachnospiraceae	Lachnospiraceae	Ruminococcaceae
Genus	Sporobacter	Anaerosporobacter	Parabacteroides	Clostridium_XlVa	Clostridium_XlVa	Lachnospiracea_incertae_sedis	Clostridium_XlVa	Clostridium_IV
#NAME	ZOTU_0335	ZOTU_0336	ZOTU_0337	ZOTU_0338	ZOTU_0339	ZOTU_0340	ZOTU_0341	ZOTU_0342
CD_49	0	0	0	0	0	0	0	0
CD_50	0	0	0	1	0	0	0	0
CD_51	0	4	0	2	3	0	0	0
CD_52	0	0	0	0	0	0	0	1
CD_65	0	0	0	0	0	0	0	0
CD_66	0	0	0	0	0	0	0	0
CD_67	0	0	0	0	0	0	0	0
CD_68	0	0	0	1	0	0	0	0
CD_78	0	0	0	0	0	0	0	0
CD_79	0	0	0	0	0	0	0	0
CD_80	0	0	0	0	0	0	0	0
CDFG_54	0	0	0	1	1	0	0	0
CDFG_55	0	0	0	3	1	0	0	3
CDFG_56	0	0	0	0	0	0	0	1
CDFG_70	0	0	0	0	1	0	0	0
CDFG_71	0	0	0	0	0	0	0	0
HF_41	0	0	0	1	0	0	0	0
HF_42	0	0	0	0	1	0	0	0
HF_43	0	0	0	0	0	0	0	0
HF_44	0	0	0	0	0	0	1	0
HF_57	0	1	0	0	0	0	1	0
HF_58	0	3	0	0	0	0	0	0
HF_59	0	2	0	2	0	0	0	0
HF_60	0	1	0	0	0	0	0	0
HF_73	0	0	0	0	0	0	0	0
HF_74	0	1	0	0	0	0	0	0
HF_75	0	0	0	0	1	0	0	0

Kingdom	Bacteria	Bacteria	Bacteria	Bacteria	Bacteria	Bacteria	Bacteria	Bacteria
Phylum	Firmicutes	Firmicutes	Bacteroidetes	Firmicutes	Firmicutes	Firmicutes	Firmicutes	Firmicutes
Class	Clostridia	Clostridia	Bacteroidia	Clostridia	Clostridia	Clostridia	Clostridia	Clostridia
Order	Clostridiales	Clostridiales	Bacteroidales	Clostridiales	Clostridiales	Clostridiales	Clostridiales	Clostridiales
Family	Ruminococcaceae	Lachnospiraceae	Porphyromonadaceae	Lachnospiraceae	Lachnospiraceae	Lachnospiraceae	Lachnospiraceae	Ruminococcaceae
Genus	Sporobacter	Anaerosporobacter	Parabacteroides	Clostridium_XlVa	Clostridium_XlVa	Lachnospiracea_incertae_sedis	Clostridium_XlVa	Clostridium_IV
#NAME	ZOTU_0335	ZOTU_0336	ZOTU_0337	ZOTU_0338	ZOTU_0339	ZOTU_0340	ZOTU_0341	ZOTU_0342
HFFG_45	0	0	0	2	1	0	7	0
HFFG_46	0	0	0	0	0	0	0	0
HFFG_47	0	0	0	0	0	0	0	0
HFFG_61	0	0	0	0	0	0	0	0
HFFG_62	0	0	0	0	0	0	0	0
HFFG_63	0	0	0	0	0	0	0	0
HFFG_64	0	0	0	0	0	0	0	0
HFFG_76	0	1	0	0	0	0	0	0

Kingdom	Bacteria	Bacteria	Bacteria	Bacteria	Bacteria	Bacteria	Bacteria	Bacteria	Bacteria
Phylum	Firmicutes	Firmicutes	Firmicutes	Firmicutes	Firmicutes	Firmicutes	Firmicutes	Firmicutes	Firmicutes
Class	Clostridia	Clostridia	Clostridia	Clostridia	Bacilli	Clostridia	Clostridia	Clostridia	Clostridia
Order	Clostridiales	Clostridiales	Clostridiales	Clostridiales	Bacillales	Clostridiales	Clostridiales	Clostridiales	Clostridiales
Family	Clostridiaceae_	Lachnospiraceae	Lachnospiraceae	Lachnospiraceae	Paenibacillaceae_	Lachnospiraceae	Lachnospiraceae	Lachnospiraceae	Lachnospiraceae
Genus	Clostridium_sensu_stricto	Blautia	Acetitomaculum	Lachnobacterium	Paenibacillus	Ruminococcus	Marvinbryantia	Acetatifactor	Blautia
#NAME	ZOTU_0343	ZOTU_0344	ZOTU_0345	ZOTU_0346	ZOTU_0347	ZOTU_0348	ZOTU_0349	ZOTU_0350	ZOTU_0351
CD_49	0	0	0	0	0	0	1	0	0
CD_50	0	0	0	0	0	0	0	1	0
CD_51	0	0	0	0	0	0	0	0	0
CD_52	0	0	0	0	0	0	0	0	0
CD_65	0	0	0	0	0	0	0	0	0
CD_66	0	0	0	0	0	0	0	0	1
CD_67	0	0	0	0	0	0	0	0	0
CD_68	0	0	0	0	0	0	0	0	0
CD_78	0	0	0	0	0	0	0	0	0
CD_79	0	0	0	0	0	0	0	0	1
CD_80	0	0	0	0	0	0	0	0	0
CDFG_54	0	0	0	0	0	0	0	0	0
CDFG_55	0	0	0	0	0	0	0	0	0
CDFG_56	0	0	0	0	0	1	0	1	1
CDFG_70	0	0	0	0	0	0	0	0	0
CDFG_71	0	0	0	0	0	0	0	0	0
HF_41	0	0	0	0	0	0	0	0	0
HF_42	0	0	0	0	0	0	0	0	0
HF_43	0	0	0	0	0	0	0	0	0
HF_44	0	0	0	0	0	0	0	0	0
HF_57	0	0	0	0	0	0	0	1	0
HF_58	0	0	0	0	0	0	0	0	0
HF_59	0	0	0	0	0	0	0	1	0
HF_60	0	0	0	0	0	0	0	0	0
HF_73	0	0	0	0	0	0	0	0	0
HF_74	0	0	1	2	0	0	0	0	0
HF_75	0	0	1	0	0	0	0	1	0

Kingdom	Bacteria	Bacteria	Bacteria	Bacteria	Bacteria	Bacteria	Bacteria	Bacteria	Bacteria
Phylum	Firmicutes	Firmicutes	Firmicutes	Firmicutes	Firmicutes	Firmicutes	Firmicutes	Firmicutes	Firmicutes
Class	Clostridia	Clostridia	Clostridia	Clostridia	Bacilli	Clostridia	Clostridia	Clostridia	Clostridia
Order	Clostridiales	Clostridiales	Clostridiales	Clostridiales	Bacillales	Clostridiales	Clostridiales	Clostridiales	Clostridiales
Family	Clostridiaceae_	Lachnospiraceae	Lachnospiraceae	Lachnospiraceae	Paenibacillaceae_	Lachnospiraceae	Lachnospiraceae	Lachnospiraceae	Lachnospiraceae
Genus	Clostridium_sensu_stricto	Blautia	Acetitomaculum	Lachnobacterium	Paenibacillus	Ruminococcus	Marvinbryantia	Acetatifactor	Blautia
#NAME	ZOTU_0343	ZOTU_0344	ZOTU_0345	ZOTU_0346	ZOTU_0347	ZOTU_0348	ZOTU_0349	ZOTU_0350	ZOTU_0351
HFFG_45	0	0	0	0	0	0	2	0	0
HFFG_46	0	0	0	0	0	0	0	0	0
HFFG_47	0	0	1	0	0	0	0	0	0
HFFG_61	0	0	0	0	0	0	1	0	0
HFFG_62	0	0	1	0	0	0	2	1	1
HFFG_63	0	0	0	0	0	0	0	0	0
HFFG_64	0	0	0	0	0	0	0	0	0
HFFG_76	0	0	2	0	0	0	0	0	1

Kingdom	Bacteria	Bacteria	Bacteria	Bacteria	Bacteria	Bacteria	Bacteria	Bacteria	Bacteria
Phylum	Firmicutes	Firmicutes	Firmicutes	Firmicutes	Firmicutes	Firmicutes	Firmicutes	Actinobacteria	Firmicutes
Class	Clostridia	Clostridia	Clostridia	Clostridia	Clostridia	Erysipelotrichia	Bacilli	Actinobacteria	Bacilli
Order	Clostridiales	Clostridiales	Clostridiales	Clostridiales	Clostridiales	Erysipelotrichales	Lactobacillales	Coriobacteriales	Lactobacillales
Family	Ruminococcaceae	Lachnospiraceae	Lachnospiraceae	Lachnospiraceae	Ruminococcaceae	Erysipelotrichaceae	Lactobacillaceae	Coriobacteriaceae	Lactobacillaceae
Genus	Intestinimonas	Acetatifactor	Acetatifactor	Shuttleworthia	Clostridium_IV	Catenisphaera	Lactobacillus	Enterorhabdus	Lactobacillus
#NAME	ZOTU_0352	ZOTU_0353	ZOTU_0354	ZOTU_0355	ZOTU_0356	ZOTU_0357	ZOTU_0358	ZOTU_0359	ZOTU_0360
CD_49	0	0	0	0	0	0	0	5	0
CD_50	0	0	0	0	0	0	0	10	0
CD_51	4	0	0	0	0	0	0	17	0
CD_52	0	0	0	0	0	0	0	15	0
CD_65	0	0	0	0	0	0	0	0	0
CD_66	0	0	0	0	0	0	0	0	0
CD_67	0	0	0	0	0	0	0	1	0
CD_68	0	0	0	0	0	0	0	0	0
CD_78	0	0	0	0	0	0	0	7	0
CD_79	0	0	0	0	0	0	0	0	0
CD_80	0	0	0	0	0	0	0	0	0
CDFG_54	0	0	0	0	0	0	0	0	0
CDFG_55	0	0	0	0	1	0	0	7	0
CDFG_56	0	0	0	0	0	0	0	4	0
CDFG_70	0	0	0	0	0	0	0	0	0
CDFG_71	0	0	0	0	0	0	0	1	0
HF_41	0	0	0	0	0	0	0	0	0
HF_42	0	0	0	0	0	0	0	0	0
HF_43	0	1	0	0	0	0	0	0	0
HF_44	0	0	0	0	0	0	0	0	0
HF_57	0	0	0	1	0	0	0	0	0
HF_58	0	0	0	1	0	0	0	0	0
HF_59	0	0	0	0	2	0	0	0	0
HF_60	0	0	0	0	0	0	0	0	0
HF_73	0	0	0	0	0	2	0	0	0
HF_74	0	0	0	0	1	3	0	0	0
HF_75	0	0	0	0	0	0	0	0	0

Kingdom	Bacteria	Bacteria	Bacteria	Bacteria	Bacteria	Bacteria	Bacteria	Bacteria	Bacteria
Phylum	Firmicutes	Firmicutes	Firmicutes	Firmicutes	Firmicutes	Firmicutes	Firmicutes	Actinobacteria	Firmicutes
Class	Clostridia	Clostridia	Clostridia	Clostridia	Clostridia	Erysipelotrichia	Bacilli	Actinobacteria	Bacilli
Order	Clostridiales	Clostridiales	Clostridiales	Clostridiales	Clostridiales	Erysipelotrichales	Lactobacillales	Coriobacteriales	Lactobacillales
Family	Ruminococcaceae	Lachnospiraceae	Lachnospiraceae	Lachnospiraceae	Ruminococcaceae	Erysipelotrichaceae	Lactobacillaceae	Coriobacteriaceae	Lactobacillaceae
Genus	Intestinimonas	Acetatifactor	Acetatifactor	Shuttleworthia	Clostridium_IV	Catenisphaera	Lactobacillus	Enterorhabdus	Lactobacillus
#NAME	ZOTU_0352	ZOTU_0353	ZOTU_0354	ZOTU_0355	ZOTU_0356	ZOTU_0357	ZOTU_0358	ZOTU_0359	ZOTU_0360
HFFG_45	0	2	0	0	3	0	0	1	0
HFFG_46	0	0	0	0	0	0	0	0	0
HFFG_47	0	0	0	0	0	1	0	0	0
HFFG_61	0	0	0	0	0	0	0	0	0
HFFG_62	1	0	0	1	0	5	0	0	0
HFFG_63	0	0	0	0	0	1	0	1	0
HFFG_64	0	0	0	0	0	0	0	0	0
HFFG_76	0	0	2	11	0	0	0	0	0

Kingdom	Bacteria	Bacteria	Bacteria	Bacteria	Bacteria	Bacteria	Bacteria	Bacteria
Phylum	Proteobacteria	Firmicutes	Firmicutes	Firmicutes	Firmicutes	Firmicutes	Firmicutes	Firmicutes
Class	Gammaproteobacteria	Bacilli	Clostridia	Clostridia	Clostridia	Clostridia	Clostridia	Clostridia
Order	Pseudomonadales	Bacillales	Clostridiales	Clostridiales	Clostridiales	Clostridiales	Clostridiales	Clostridiales
Family	Pseudomonadaceae	Staphylococcaceae	Natranaerovirga	Lachnospiraceae	Lachnospiraceae	Lachnospiraceae	Lachnospiraceae	Clostridiaceae_
Genus	Pseudomonas	Staphylococcus	NA	Clostridium_XlVa	Clostridium_XlVa	Clostridium_XlVa	Lachnospiracea_incertae_sedis	Alkaliphilus
#NAME	ZOTU_0361	ZOTU_0362	ZOTU_0363	ZOTU_0364	ZOTU_0365	ZOTU_0366	ZOTU_0367	ZOTU_0368
CD_49	0	0	0	0	0	0	0	0
CD_50	0	0	0	0	0	0	0	0
CD_51	0	1	0	0	0	0	0	0
CD_52	0	0	0	0	0	0	0	0
CD_65	0	0	0	0	0	0	0	0
CD_66	0	0	0	0	0	0	0	0
CD_67	0	0	0	0	0	0	0	0
CD_68	0	0	0	0	0	0	0	0
CD_78	0	3	0	0	0	0	0	0
CD_79	0	1	0	0	0	0	0	0
CD_80	0	0	0	0	0	0	0	0
CDFG_54	0	0	0	0	0	0	0	0
CDFG_55	0	2	0	0	0	0	0	0
CDFG_56	0	6	0	0	0	0	0	0
CDFG_70	0	0	0	0	0	0	0	0
CDFG_71	0	0	0	0	0	0	0	0
HF_41	0	0	0	0	2	0	0	0
HF_42	0	0	0	0	0	0	0	0
HF_43	0	0	0	0	0	0	0	0
HF_44	0	0	0	0	1	0	0	0
HF_57	0	1	0	0	0	0	0	0
HF_58	0	0	0	0	0	0	0	0
HF_59	0	0	0	0	0	0	0	0
HF_60	0	0	0	0	0	0	0	0
HF_73	0	2	0	0	0	0	0	0
HF_74	0	0	0	0	1	0	0	0
HF_75	0	0	0	0	0	0	0	0

Kingdom	Bacteria	Bacteria	Bacteria	Bacteria	Bacteria	Bacteria	Bacteria	Bacteria
Phylum	Proteobacteria	Firmicutes	Firmicutes	Firmicutes	Firmicutes	Firmicutes	Firmicutes	Firmicutes
Class	Gammaproteobacteria	Bacilli	Clostridia	Clostridia	Clostridia	Clostridia	Clostridia	Clostridia
Order	Pseudomonadales	Bacillales	Clostridiales	Clostridiales	Clostridiales	Clostridiales	Clostridiales	Clostridiales
Family	Pseudomonadaceae	Staphylococcaceae	Natranaerovirga	Lachnospiraceae	Lachnospiraceae	Lachnospiraceae	Lachnospiraceae	Clostridiaceae_
Genus	Pseudomonas	Staphylococcus	NA	Clostridium_XlVa	Clostridium_XlVa	Clostridium_XlVa	Lachnospiracea_incertae_sedis	Alkaliphilus
#NAME	ZOTU_0361	ZOTU_0362	ZOTU_0363	ZOTU_0364	ZOTU_0365	ZOTU_0366	ZOTU_0367	ZOTU_0368
HFFG_45	0	0	0	0	0	0	0	0
HFFG_46	0	29	0	0	1	0	0	0
HFFG_47	0	1	0	0	0	0	0	0
HFFG_61	0	0	0	0	0	0	0	0
HFFG_62	0	0	0	0	0	0	2	0
HFFG_63	0	0	0	0	0	0	0	0
HFFG_64	0	0	0	0	0	0	0	0
HFFG_76	0	0	0	0	0	0	0	0

Kingdom	Bacteria	Bacteria	Bacteria	Bacteria	Bacteria	Bacteria	Bacteria	Bacteria	Bacteria
Phylum	Firmicutes	Firmicutes	Actinobacteria	Firmicutes	Firmicutes	Firmicutes	Firmicutes	Firmicutes	Firmicutes
Class	Clostridia	Clostridia	Actinobacteria	Bacilli	Bacilli	Bacilli	Bacilli	Clostridia	Clostridia
Order	Clostridiales	Clostridiales	Actinomycetales	Lactobacillales	Bacillales	Lactobacillales	Bacillales	Clostridiales	Clostridiales
Family	Clostridiaceae_	Peptostreptococcaceae	Dietziaceae	Enterococcaceae	Planococcaceae	Streptococcaceae	Bacillaceae_	Lachnospiraceae	Ruminococcaceae
Genus	Clostridium_sensu_stricto	Clostridium_XI	Dietzia	Enterococcus	Viridibacillus	Streptococcus	Bacillus	Acetatifactor	Clostridium_IV
#NAME	ZOTU_0369	ZOTU_0370	ZOTU_0371	ZOTU_0372	ZOTU_0373	ZOTU_0374	ZOTU_0375	ZOTU_0376	ZOTU_0377
CD_49	0	0	0	0	0	0	0	0	0
CD_50	0	0	0	0	0	0	0	0	0
CD_51	0	0	0	34	8	0	0	0	4
CD_52	1	0	0	0	0	0	0	0	0
CD_65	0	0	0	0	0	0	0	0	0
CD_66	0	0	0	0	1	0	0	0	0
CD_67	0	0	0	0	0	0	0	0	0
CD_68	0	0	0	0	0	0	0	0	0
CD_78	0	0	0	2	3	0	0	0	0
CD_79	0	0	0	0	39	0	6	0	0
CD_80	0	0	0	0	0	0	0	0	0
CDFG_54	0	0	0	7	0	0	0	0	0
CDFG_55	0	0	0	6	59	0	12	0	0
CDFG_56	0	0	0	5	71	0	7	0	0
CDFG_70	0	0	0	0	1	0	0	0	0
CDFG_71	0	0	0	0	1	0	0	0	0
HF_41	0	0	0	0	0	0	0	0	0
HF_42	0	0	0	0	2	0	1	0	0
HF_43	0	0	0	0	0	0	0	0	0
HF_44	0	0	0	0	7	0	3	0	0
HF_57	0	0	0	0	0	0	0	3	0
HF_58	0	0	0	0	10	1	2	4	0
HF_59	0	0	0	0	383	0	20	2	1
HF_60	0	0	0	0	6	0	0	0	0
HF_73	0	0	0	0	3	0	0	0	0
HF_74	0	0	0	0	2	0	0	0	0
HF_75	0	0	0	0	0	0	0	0	0

Kingdom	Bacteria	Bacteria	Bacteria	Bacteria	Bacteria	Bacteria	Bacteria	Bacteria	Bacteria
Phylum	Firmicutes	Firmicutes	Actinobacteria	Firmicutes	Firmicutes	Firmicutes	Firmicutes	Firmicutes	Firmicutes
Class	Clostridia	Clostridia	Actinobacteria	Bacilli	Bacilli	Bacilli	Bacilli	Clostridia	Clostridia
Order	Clostridiales	Clostridiales	Actinomycetales	Lactobacillales	Bacillales	Lactobacillales	Bacillales	Clostridiales	Clostridiales
Family	Clostridiaceae_	Peptostreptococcaceae	Dietziaceae	Enterococcaceae	Planococcaceae	Streptococcaceae	Bacillaceae_	Lachnospiraceae	Ruminococcaceae
Genus	Clostridium_sensu_stricto	Clostridium_XI	Dietzia	Enterococcus	Viridibacillus	Streptococcus	Bacillus	Acetatifactor	Clostridium_IV
#NAME	ZOTU_0369	ZOTU_0370	ZOTU_0371	ZOTU_0372	ZOTU_0373	ZOTU_0374	ZOTU_0375	ZOTU_0376	ZOTU_0377
HFFG_45	0	0	0	3	5	0	0	0	0
HFFG_46	0	0	0	0	19	0	0	0	0
HFFG_47	0	0	0	0	1	0	0	0	0
HFFG_61	0	0	0	0	0	0	0	0	0
HFFG_62	0	0	0	1	1	0	0	0	0
HFFG_63	0	0	0	0	0	0	0	0	0
HFFG_64	0	0	0	0	0	0	0	0	0
HFFG_76	0	0	0	0	0	0	0	1	0

Kingdom	Bacteria	Bacteria	Bacteria	Bacteria	Bacteria	Bacteria	Bacteria	Bacteria
Phylum	Firmicutes	Firmicutes	Firmicutes	Firmicutes	Firmicutes	Firmicutes	Firmicutes	Firmicutes
Class	Clostridia	Clostridia	Clostridia	Clostridia	Bacilli	Clostridia	Clostridia	Clostridia
Order	Clostridiales	Clostridiales	Clostridiales	Clostridiales	Bacillales	Clostridiales	Clostridiales	Clostridiales
Family	Ruminococcaceae	Lachnospiraceae	Lachnospiraceae	Clostridiaceae_	Planococcaceae	Lachnospiraceae	Clostridiaceae_	Lachnospiraceae
Genus	Oscillibacter	Acetatifactor	Clostridium_XlVa	Clostridium_sensu_stricto	Viridibacillus	Clostridium_XlVa	Clostridium_sensu_stricto	Clostridium_XlVa
#NAME	ZOTU_0378	ZOTU_0379	ZOTU_0380	ZOTU_0381	ZOTU_0382	ZOTU_0383	ZOTU_0384	ZOTU_0385
CD_49	0	1	0	0	0	0	0	0
CD_50	0	0	0	0	0	0	0	10
CD_51	1	0	0	0	2	0	0	1
CD_52	0	0	0	0	0	0	0	0
CD_65	0	1	0	0	0	0	0	0
CD_66	0	0	0	0	0	0	0	0
CD_67	0	0	0	0	0	0	0	0
CD_68	0	0	0	0	0	0	0	0
CD_78	0	0	0	0	0	0	0	0
CD_79	0	0	0	0	0	0	0	0
CD_80	0	0	0	0	0	0	0	0
CDFG_54	0	0	0	0	1	0	0	0
CDFG_55	0	0	0	0	0	0	0	0
CDFG_56	0	0	0	0	5	0	0	0
CDFG_70	0	0	0	0	0	0	0	0
CDFG_71	0	0	0	0	0	0	0	0
HF_41	0	0	1	1	0	0	0	0
HF_42	1	0	0	3	0	0	0	0
HF_43	1	0	0	0	0	0	0	0
HF_44	0	0	0	0	0	1	0	0
HF_57	0	2	0	0	0	0	0	0
HF_58	0	2	0	0	2	0	0	0
HF_59	0	3	0	0	1	0	0	0
HF_60	0	0	0	0	0	0	0	0
HF_73	0	0	0	0	0	0	0	0
HF_74	0	0	0	0	0	0	0	1
HF_75	0	2	0	0	0	1	0	0

Kingdom	Bacteria	Bacteria	Bacteria	Bacteria	Bacteria	Bacteria	Bacteria	Bacteria
Phylum	Firmicutes	Firmicutes	Firmicutes	Firmicutes	Firmicutes	Firmicutes	Firmicutes	Firmicutes
Class	Clostridia	Clostridia	Clostridia	Clostridia	Bacilli	Clostridia	Clostridia	Clostridia
Order	Clostridiales	Clostridiales	Clostridiales	Clostridiales	Bacillales	Clostridiales	Clostridiales	Clostridiales
Family	Ruminococcaceae	Lachnospiraceae	Lachnospiraceae	Clostridiaceae_	Planococcaceae	Lachnospiraceae	Clostridiaceae_	Lachnospiraceae
Genus	Oscillibacter	Acetatifactor	Clostridium_XlVa	Clostridium_sensu_stricto	Viridibacillus	Clostridium_XlVa	Clostridium_sensu_stricto	Clostridium_XlVa
#NAME	ZOTU_0378	ZOTU_0379	ZOTU_0380	ZOTU_0381	ZOTU_0382	ZOTU_0383	ZOTU_0384	ZOTU_0385
HFFG_45	0	0	0	0	1	1	0	4
HFFG_46	0	0	0	0	0	0	0	0
HFFG_47	0	0	0	0	0	0	0	0
HFFG_61	0	0	0	0	0	0	0	0
HFFG_62	0	0	0	0	0	0	0	0
HFFG_63	0	0	0	0	0	0	0	0
HFFG_64	0	0	0	0	0	0	0	0
HFFG_76	1	0	0	0	0	1	0	0

Kingdom	Bacteria	Bacteria	Bacteria	Bacteria	Bacteria	Bacteria	Bacteria	Bacteria
Phylum	Firmicutes	Firmicutes	Firmicutes	Proteobacteria	Cyanobacteria_Chloroplast	Tenericutes	Cyanobacteria_Chloroplast	Firmicutes
Class	Clostridia	Clostridia	Negativicutes	Betaproteobacteria	Chloroplast	Mollicutes	Chloroplast	Clostridia
Order	Clostridiales	Clostridiales	Selenomonadales	Neisseriales	Chloroplast	Acholeplasmatales	Chloroplast	Clostridiales
Family	Ruminococcaceae	Clostridiaceae_	Veillonellaceae	Neisseriaceae	Streptophyta	Acholeplasmataceae	Streptophyta	Lachnospiraceae
Genus	Clostridium_IV	Alkaliphilus	Veillonella	Neisseria	NA	Acholeplasma	NA	Coprococcus
#NAME	ZOTU_0386	ZOTU_0387	ZOTU_0388	ZOTU_0389	ZOTU_0390	ZOTU_0391	ZOTU_0392	ZOTU_0393
CD_49	0	0	0	0	0	0	0	0
CD_50	0	0	0	0	0	0	0	0
CD_51	3	0	0	0	0	0	0	0
CD_52	0	0	0	0	0	0	0	0
CD_65	0	0	0	0	0	0	0	0
CD_66	0	0	0	0	0	0	0	0
CD_67	0	0	0	0	0	0	0	0
CD_68	0	0	0	0	0	0	0	0
CD_78	0	0	0	0	0	0	0	0
CD_79	0	0	0	0	0	0	0	0
CD_80	0	0	0	0	0	0	0	0
CDFG_54	0	0	0	0	0	0	0	0
CDFG_55	0	0	0	0	0	0	0	0
CDFG_56	0	0	0	0	0	0	0	0
CDFG_70	0	0	0	0	0	0	0	0
CDFG_71	0	0	0	0	0	0	0	0
HF_41	0	0	0	0	0	0	0	0
HF_42	0	0	0	0	0	0	0	0
HF_43	0	0	0	0	0	0	0	0
HF_44	0	0	0	0	0	0	0	0
HF_57	0	0	0	0	0	0	0	0
HF_58	0	0	0	1	0	0	0	0
HF_59	0	0	0	0	0	0	0	0
HF_60	0	0	0	0	0	2	0	0
HF_73	0	0	0	0	0	0	0	2
HF_74	0	0	0	0	0	0	0	1
HF_75	0	0	0	0	0	0	0	0

Kingdom	Bacteria	Bacteria	Bacteria	Bacteria	Bacteria	Bacteria	Bacteria	Bacteria
Phylum	Firmicutes	Firmicutes	Firmicutes	Proteobacteria	Cyanobacteria_Chloroplast	Tenericutes	Cyanobacteria_Chloroplast	Firmicutes
Class	Clostridia	Clostridia	Negativicutes	Betaproteobacteria	Chloroplast	Mollicutes	Chloroplast	Clostridia
Order	Clostridiales	Clostridiales	Selenomonadales	Neisseriales	Chloroplast	Acholeplasmatales	Chloroplast	Clostridiales
Family	Ruminococcaceae	Clostridiaceae_	Veillonellaceae	Neisseriaceae	Streptophyta	Acholeplasmataceae	Streptophyta	Lachnospiraceae
Genus	Clostridium_IV	Alkaliphilus	Veillonella	Neisseria	NA	Acholeplasma	NA	Coprococcus
#NAME	ZOTU_0386	ZOTU_0387	ZOTU_0388	ZOTU_0389	ZOTU_0390	ZOTU_0391	ZOTU_0392	ZOTU_0393
HFFG_45	0	0	0	0	0	0	0	0
HFFG_46	0	0	0	0	0	0	0	0
HFFG_47	0	0	0	0	0	0	0	0
HFFG_61	0	0	0	0	0	0	0	0
HFFG_62	0	0	1	0	0	0	0	0
HFFG_63	0	0	0	0	0	0	0	0
HFFG_64	0	0	0	0	0	0	0	0
HFFG_76	0	0	0	0	0	0	0	1

Kingdom	Bacteria	Bacteria	Bacteria	Bacteria	Bacteria	Bacteria	Bacteria
Phylum	Bacteroidetes	Firmicutes	Bacteroidetes	Proteobacteria	Actinobacteria	Firmicutes	Firmicutes
Class	Bacteroidia	Negativicutes	Bacteroidia	Betaproteobacteria	Actinobacteria	Clostridia	Clostridia
Order	Bacteroidales	Selenomonadales	Bacteroidales	Burkholderiales	Actinomycetales	Clostridiales	Clostridiales
Family	Porphyromonadaceae	Veillonellaceae	Porphyromonadaceae	Burkholderiaceae	Corynebacteriaceae	Clostridiales_Incertae_Sedis_XIII	Lachnospiraceae
Genus	Parabacteroides	Veillonella	Parabacteroides	Burkholderia	Corynebacterium	Anaerovorax	Acetatifactor
#NAME	ZOTU_0394	ZOTU_0395	ZOTU_0396	ZOTU_0397	ZOTU_0398	ZOTU_0399	ZOTU_0400
CD_49	0	0	0	0	0	0	0
CD_50	0	0	0	0	0	0	0
CD_51	0	0	0	0	0	3	0
CD_52	0	0	0	0	0	0	0
CD_65	2	0	0	0	0	0	0
CD_66	1	0	0	0	0	0	0
CD_67	1	0	0	0	0	0	0
CD_68	2	0	0	0	0	0	0
CD_78	2	0	0	0	0	0	0
CD_79	0	0	0	0	0	0	0
CD_80	0	0	0	0	0	0	0
CDFG_54	0	0	0	0	0	0	0
CDFG_55	0	0	0	0	0	0	0
CDFG_56	0	0	0	0	0	0	0
CDFG_70	0	0	0	0	0	0	0
CDFG_71	0	0	0	0	0	0	0
HF_41	0	0	0	0	0	0	0
HF_42	0	0	0	0	0	0	0
HF_43	0	0	0	0	0	0	0
HF_44	0	0	0	0	0	1	0
HF_57	0	0	0	0	0	0	0
HF_58	0	0	0	0	0	0	0
HF_59	0	0	0	0	0	0	0
HF_60	0	0	0	0	0	0	0
HF_73	0	0	0	0	0	0	0
HF_74	0	0	0	0	0	0	0
HF_75	0	0	0	0	1	0	0

Kingdom	Bacteria	Bacteria	Bacteria	Bacteria	Bacteria	Bacteria	Bacteria
Phylum	Bacteroidetes	Firmicutes	Bacteroidetes	Proteobacteria	Actinobacteria	Firmicutes	Firmicutes
Class	Bacteroidia	Negativicutes	Bacteroidia	Betaproteobacteria	Actinobacteria	Clostridia	Clostridia
Order	Bacteroidales	Selenomonadales	Bacteroidales	Burkholderiales	Actinomycetales	Clostridiales	Clostridiales
Family	Porphyromonadaceae	Veillonellaceae	Porphyromonadaceae	Burkholderiaceae	Corynebacteriaceae	Clostridiales_Incertae_Sedis_XIII	Lachnospiraceae
Genus	Parabacteroides	Veillonella	Parabacteroides	Burkholderia	Corynebacterium	Anaerovorax	Acetatifactor
#NAME	ZOTU_0394	ZOTU_0395	ZOTU_0396	ZOTU_0397	ZOTU_0398	ZOTU_0399	ZOTU_0400
HFFG_45	0	0	0	0	0	3	0
HFFG_46	0	0	0	0	0	0	0
HFFG_47	0	0	0	0	0	0	0
HFFG_61	0	0	0	0	0	0	0
HFFG_62	0	0	0	0	0	0	0
HFFG_63	0	0	0	0	0	0	0
HFFG_64	0	0	0	0	0	0	0
HFFG_76	0	0	0	0	0	0	0

Kingdom	Bacteria	Bacteria	Bacteria	Bacteria	Bacteria	Bacteria	Bacteria	Bacteria
Phylum	Firmicutes	Proteobacteria	Firmicutes	Firmicutes	Actinobacteria	Firmicutes	Firmicutes	Firmicutes
Class	Erysipelotrichia	Betaproteobacteria	Negativicutes	Clostridia	Actinobacteria	Clostridia	Clostridia	Clostridia
Order	Erysipelotrichales	Burkholderiales	Selenomonadales	Clostridiales	Actinomycetales	Clostridiales	Clostridiales	Clostridiales
Family	Erysipelotrichaceae	Burkholderiaceae	Acidaminococcaceae	Eubacteriaceae	Corynebacteriaceae	Lachnospiraceae	Ruminococcaceae	Ruminococcaceae
Genus	Clostridium_XVIII	Ralstonia	Phascolarctobacterium	Anaerofustis	Corynebacterium	Acetatifactor	Oscillibacter	Ruminococcus
#NAME	ZOTU_0401	ZOTU_0402	ZOTU_0403	ZOTU_0404	ZOTU_0405	ZOTU_0406	ZOTU_0407	ZOTU_0408
CD_49	2	0	0	0	0	0	0	0
CD_50	2	0	0	0	0	0	0	0
CD_51	2	0	0	0	0	0	1	0
CD_52	0	0	0	0	0	0	0	0
CD_65	0	0	0	0	0	0	0	0
CD_66	0	0	0	0	0	0	0	0
CD_67	0	0	0	0	0	0	0	0
CD_68	0	0	0	0	0	0	0	0
CD_78	8	0	0	0	0	0	0	0
CD_79	3	0	0	0	0	0	0	0
CD_80	0	0	0	0	0	0	0	0
CDFG_54	0	0	0	0	0	0	0	0
CDFG_55	1	0	0	0	0	0	0	0
CDFG_56	1	0	0	0	0	0	0	0
CDFG_70	0	0	0	0	0	0	0	0
CDFG_71	0	0	0	0	0	0	0	0
HF_41	0	0	0	0	0	0	0	0
HF_42	0	0	0	0	0	0	0	0
HF_43	0	0	0	0	0	0	0	0
HF_44	0	0	0	0	0	0	0	0
HF_57	2	0	0	0	0	0	0	0
HF_58	4	0	0	0	0	0	0	0
HF_59	2	0	0	0	0	0	0	0
HF_60	0	0	0	0	0	0	0	0
HF_73	1	0	0	0	0	1	0	0
HF_74	3	0	0	0	0	1	1	0
HF_75	6	0	0	0	0	0	0	0

Kingdom	Bacteria	Bacteria	Bacteria	Bacteria	Bacteria	Bacteria	Bacteria	Bacteria
Phylum	Firmicutes	Proteobacteria	Firmicutes	Firmicutes	Actinobacteria	Firmicutes	Firmicutes	Firmicutes
Class	Erysipelotrichia	Betaproteobacteria	Negativicutes	Clostridia	Actinobacteria	Clostridia	Clostridia	Clostridia
Order	Erysipelotrichales	Burkholderiales	Selenomonadales	Clostridiales	Actinomycetales	Clostridiales	Clostridiales	Clostridiales
Family	Erysipelotrichaceae	Burkholderiaceae	Acidaminococcaceae	Eubacteriaceae	Corynebacteriaceae	Lachnospiraceae	Ruminococcaceae	Ruminococcaceae
Genus	Clostridium_XVIII	Ralstonia	Phascolarctobacterium	Anaerofustis	Corynebacterium	Acetatifactor	Oscillibacter	Ruminococcus
#NAME	ZOTU_0401	ZOTU_0402	ZOTU_0403	ZOTU_0404	ZOTU_0405	ZOTU_0406	ZOTU_0407	ZOTU_0408
HFFG_45	0	0	0	0	0	0	0	0
HFFG_46	0	0	0	0	0	0	0	0
HFFG_47	0	0	0	0	0	0	0	0
HFFG_61	0	0	0	0	0	0	0	0
HFFG_62	2	0	0	0	0	1	0	0
HFFG_63	3	0	0	0	0	0	0	0
HFFG_64	0	0	0	0	0	0	0	0
HFFG_76	3	0	0	0	0	1	1	0

Kingdom	Bacteria	Bacteria	Bacteria	Bacteria	Bacteria	Bacteria	Bacteria
Phylum	Bacteroidetes	Firmicutes	Actinobacteria	Proteobacteria	Cyanobacteria_Chloroplast	Bacteroidetes	Tenericutes
Class	Bacteroidia	Clostridia	Actinobacteria	Betaproteobacteria	Chloroplast	Bacteroidia	Mollicutes
Order	Bacteroidales	Clostridiales	Coriobacteriales	Neisseriales	Chloroplast	Bacteroidales	Acholeplasmatales
Family	Porphyromonadaceae	Lachnospiraceae	Coriobacteriaceae	Neisseriaceae	Streptophyta	Porphyromonadaceae	Acholeplasmataceae
Genus	Parabacteroides	Clostridium_XlVa	Atopobium	Neisseria	NA	Parabacteroides	Acholeplasma
#NAME	ZOTU_0409	ZOTU_0410	ZOTU_0411	ZOTU_0412	ZOTU_0413	ZOTU_0414	ZOTU_0415
CD_49	0	0	0	0	0	0	0
CD_50	0	0	0	0	0	0	0
CD_51	0	0	0	0	0	0	0
CD_52	0	0	0	0	0	0	0
CD_65	0	0	0	0	0	0	0
CD_66	0	0	0	0	0	0	0
CD_67	0	0	0	0	0	0	0
CD_68	0	0	0	0	0	0	0
CD_78	0	0	0	0	0	0	0
CD_79	0	0	0	0	0	0	0
CD_80	0	0	0	0	0	0	0
CDFG_54	0	0	0	0	0	0	0
CDFG_55	0	0	0	0	0	0	0
CDFG_56	0	0	0	0	1	0	0
CDFG_70	0	0	0	0	0	0	0
CDFG_71	0	0	0	0	0	0	0
HF_41	0	0	0	0	0	0	0
HF_42	0	0	0	0	0	0	1
HF_43	0	0	0	0	0	0	0
HF_44	0	0	0	0	0	0	0
HF_57	0	0	0	0	0	0	0
HF_58	0	0	0	0	0	1	0
HF_59	0	0	0	0	0	0	0
HF_60	0	0	0	0	0	0	0
HF_73	0	0	0	0	0	0	0
HF_74	0	0	0	0	0	0	0
HF_75	0	0	0	0	0	0	0

Kingdom	Bacteria	Bacteria	Bacteria	Bacteria	Bacteria	Bacteria	Bacteria
Phylum	Bacteroidetes	Firmicutes	Actinobacteria	Proteobacteria	Cyanobacteria_Chloroplast	Bacteroidetes	Tenericutes
Class	Bacteroidia	Clostridia	Actinobacteria	Betaproteobacteria	Chloroplast	Bacteroidia	Mollicutes
Order	Bacteroidales	Clostridiales	Coriobacteriales	Neisseriales	Chloroplast	Bacteroidales	Acholeplasmatales
Family	Porphyromonadaceae	Lachnospiraceae	Coriobacteriaceae	Neisseriaceae	Streptophyta	Porphyromonadaceae	Acholeplasmataceae
Genus	Parabacteroides	Clostridium_XlVa	Atopobium	Neisseria	NA	Parabacteroides	Acholeplasma
#NAME	ZOTU_0409	ZOTU_0410	ZOTU_0411	ZOTU_0412	ZOTU_0413	ZOTU_0414	ZOTU_0415
HFFG_45	0	0	0	0	0	0	0
HFFG_46	0	0	0	0	0	0	0
HFFG_47	0	0	0	0	0	0	0
HFFG_61	0	0	0	0	0	0	0
HFFG_62	0	0	0	0	0	0	0
HFFG_63	0	0	0	0	0	0	0
HFFG_64	0	0	0	0	0	0	0
HFFG_76	0	0	0	0	0	0	0

Kingdom	Bacteria	Bacteria	Bacteria	Bacteria	Bacteria	Bacteria	Bacteria	Bacteria
Phylum	Firmicutes	Firmicutes	Firmicutes	Cyanobacteria_Chloroplast	Firmicutes	Actinobacteria	Firmicutes	Firmicutes
Class	Clostridia	Clostridia	Clostridia	Chloroplast	Clostridia	Actinobacteria	Bacilli	Bacilli
Order	Clostridiales	Clostridiales	Clostridiales	Chloroplast	Clostridiales	Coriobacteriales	Bacillales	Bacillales
Family	Clostridiaceae_	Lachnospiraceae	Clostridiaceae_	Streptophyta	Lachnospiraceae	Coriobacteriaceae	Staphylococcaceae	Bacillaceae_
Genus	Clostridium_sensu_stricto	Clostridium_XlVa	Clostridium_sensu_stricto	NA	Butyrivibrio	Enterorhabdus	Staphylococcus	Caldibacillus
#NAME	ZOTU_0416	ZOTU_0417	ZOTU_0418	ZOTU_0419	ZOTU_0420	ZOTU_0421	ZOTU_0422	ZOTU_0423
CD_49	0	0	0	0	0	3	0	0
CD_50	0	0	0	0	0	3	0	0
CD_51	0	0	0	0	0	1	0	0
CD_52	0	0	0	0	0	9	0	0
CD_65	0	0	0	0	0	0	0	0
CD_66	0	0	0	0	0	1	1	0
CD_67	0	0	0	0	0	0	2	0
CD_68	0	0	0	0	0	0	0	0
CD_78	0	0	0	0	0	3	0	0
CD_79	0	0	0	0	0	0	0	0
CD_80	0	0	0	0	0	0	0	0
CDFG_54	0	0	0	0	0	1	0	0
CDFG_55	0	0	0	0	0	1	0	0
CDFG_56	0	0	0	0	0	2	0	0
CDFG_70	0	0	0	0	0	0	0	0
CDFG_71	0	0	0	0	0	0	0	0
HF_41	0	0	0	0	0	0	0	0
HF_42	0	0	0	0	0	0	0	0
HF_43	0	0	0	0	0	0	0	0
HF_44	0	0	0	0	0	0	1	0
HF_57	0	0	0	0	0	0	0	0
HF_58	0	0	0	0	0	0	0	0
HF_59	0	0	0	0	0	0	9	0
HF_60	0	0	0	0	0	0	0	0
HF_73	0	0	0	0	0	0	0	0
HF_74	0	0	0	0	0	0	0	0
HF_75	0	0	0	0	0	0	0	0

Kingdom	Bacteria	Bacteria	Bacteria	Bacteria	Bacteria	Bacteria	Bacteria	Bacteria
Phylum	Firmicutes	Firmicutes	Firmicutes	Cyanobacteria_Chloroplast	Firmicutes	Actinobacteria	Firmicutes	Firmicutes
Class	Clostridia	Clostridia	Clostridia	Chloroplast	Clostridia	Actinobacteria	Bacilli	Bacilli
Order	Clostridiales	Clostridiales	Clostridiales	Chloroplast	Clostridiales	Coriobacteriales	Bacillales	Bacillales
Family	Clostridiaceae_	Lachnospiraceae	Clostridiaceae_	Streptophyta	Lachnospiraceae	Coriobacteriaceae	Staphylococcaceae	Bacillaceae_
Genus	Clostridium_sensu_stricto	Clostridium_XlVa	Clostridium_sensu_stricto	NA	Butyrivibrio	Enterorhabdus	Staphylococcus	Caldibacillus
#NAME	ZOTU_0416	ZOTU_0417	ZOTU_0418	ZOTU_0419	ZOTU_0420	ZOTU_0421	ZOTU_0422	ZOTU_0423
HFFG_45	0	0	0	0	1	0	0	0
HFFG_46	0	0	0	0	0	0	0	0
HFFG_47	0	0	0	0	0	0	1	0
HFFG_61	0	0	0	0	0	0	0	0
HFFG_62	0	0	0	0	0	0	1	0
HFFG_63	0	0	0	0	0	0	0	0
HFFG_64	0	0	0	0	0	0	0	0
HFFG_76	0	0	0	0	0	0	0	0

Kingdom	Bacteria	Bacteria	Bacteria	Bacteria	Bacteria	Bacteria	Bacteria	Bacteria
Phylum	Actinobacteria	Proteobacteria	Firmicutes	Firmicutes	Firmicutes	Firmicutes	Actinobacteria	Firmicutes
Class	Actinobacteria	Gammaproteobacteria	Bacilli	Bacilli	Bacilli	Clostridia	Actinobacteria	Bacilli
Order	Actinomycetales	Pasteurellales	Lactobacillales	Bacillales	Bacillales	Clostridiales	Actinomycetales	Bacillales
Family	Corynebacteriaceae	Pasteurellaceae	Lactobacillaceae	Bacillaceae_	Bacillales_Incertae_Sedis_XI	Clostridiaceae_	Actinomycetaceae	Staphylococcaceae
Genus	Corynebacterium	Actinobacillus	Lactobacillus	Bacillus	Gemella	Clostridium_sensu_stricto	Actinomyces	Staphylococcus
#NAME	ZOTU_0424	ZOTU_0425	ZOTU_0426	ZOTU_0427	ZOTU_0428	ZOTU_0429	ZOTU_0430	ZOTU_0431
CD_49	0	0	0	0	0	0	0	0
CD_50	0	0	0	0	0	0	0	0
CD_51	0	0	0	0	0	0	0	0
CD_52	0	0	0	0	0	0	0	0
CD_65	0	0	0	0	0	0	0	0
CD_66	0	0	0	0	0	2	0	0
CD_67	0	0	0	0	0	0	0	0
CD_68	0	0	0	0	0	0	0	0
CD_78	0	0	0	0	0	0	0	0
CD_79	0	0	0	0	0	0	0	0
CD_80	0	0	0	0	0	0	0	0
CDFG_54	0	0	0	0	0	0	0	0
CDFG_55	0	0	0	0	0	0	0	0
CDFG_56	0	0	0	0	0	0	0	0
CDFG_70	0	0	0	0	0	0	0	0
CDFG_71	0	0	0	0	0	0	0	0
HF_41	0	0	0	0	0	0	0	0
HF_42	0	0	0	0	0	0	0	0
HF_43	0	0	0	0	0	0	0	0
HF_44	0	0	0	0	0	0	0	0
HF_57	0	0	0	0	0	0	0	0
HF_58	0	0	0	0	0	0	0	0
HF_59	0	0	0	0	0	0	0	0
HF_60	0	0	0	0	0	0	0	0
HF_73	0	0	0	0	0	0	0	0
HF_74	0	0	0	0	0	0	0	0
HF_75	0	0	0	0	0	0	0	0

Kingdom	Bacteria	Bacteria	Bacteria	Bacteria	Bacteria	Bacteria	Bacteria	Bacteria
Phylum	Actinobacteria	Proteobacteria	Firmicutes	Firmicutes	Firmicutes	Firmicutes	Actinobacteria	Firmicutes
Class	Actinobacteria	Gammaproteobacteria	Bacilli	Bacilli	Bacilli	Clostridia	Actinobacteria	Bacilli
Order	Actinomycetales	Pasteurellales	Lactobacillales	Bacillales	Bacillales	Clostridiales	Actinomycetales	Bacillales
Family	Corynebacteriaceae	Pasteurellaceae	Lactobacillaceae	Bacillaceae_	Bacillales_Incertae_Sedis_XI	Clostridiaceae_	Actinomycetaceae	Staphylococcaceae
Genus	Corynebacterium	Actinobacillus	Lactobacillus	Bacillus	Gemella	Clostridium_sensu_stricto	Actinomyces	Staphylococcus
#NAME	ZOTU_0424	ZOTU_0425	ZOTU_0426	ZOTU_0427	ZOTU_0428	ZOTU_0429	ZOTU_0430	ZOTU_0431
HFFG_45	0	0	0	0	0	0	0	0
HFFG_46	0	0	0	0	0	0	0	3
HFFG_47	0	0	0	0	0	0	0	0
HFFG_61	0	0	0	0	0	0	0	0
HFFG_62	0	0	0	0	0	0	1	0
HFFG_63	0	0	0	0	0	0	0	0
HFFG_64	0	0	0	0	0	0	1	0
HFFG_76	0	0	0	0	0	0	0	0

Kingdom	Bacteria	Bacteria	Bacteria	Bacteria	Bacteria	Bacteria	Bacteria	Bacteria
Phylum	Proteobacteria	Firmicutes	Firmicutes	Firmicutes	Actinobacteria	Firmicutes	Firmicutes	Bacteroidetes
Class	Alphaproteobacteria	Erysipelotrichia	Clostridia	Clostridia	Actinobacteria	Clostridia	Clostridia	Bacteroidia
Order	Rhizobiales	Erysipelotrichales	Clostridiales	Clostridiales	Coriobacteriales	Clostridiales	Clostridiales	Bacteroidales
Family	Bradyrhizobiaceae	Erysipelotrichaceae	Clostridiaceae_	Lachnospiraceae	Coriobacteriaceae	Lachnospiraceae	Lachnospiraceae	Porphyromonadaceae
Genus	Bradyrhizobium	Erysipelotrichaceae_incertae_sedis	Alkaliphilus	Clostridium_XlVa	Enterorhabdus	Clostridium_XlVa	Clostridium_XlVa	Parabacteroides
#NAME	ZOTU_0432	ZOTU_0433	ZOTU_0434	ZOTU_0435	ZOTU_0436	ZOTU_0437	ZOTU_0438	ZOTU_0439
CD_49	0	0	0	1	0	0	0	0
CD_50	0	0	0	0	1	0	0	0
CD_51	0	0	0	0	0	0	0	0
CD_52	0	0	0	0	1	0	0	0
CD_65	0	0	0	0	0	0	0	0
CD_66	0	0	0	0	0	0	0	0
CD_67	0	0	0	0	0	0	0	0
CD_68	0	0	0	0	0	1	0	0
CD_78	0	0	0	0	0	0	0	0
CD_79	0	0	0	0	0	0	0	0
CD_80	0	0	0	0	0	0	0	0
CDFG_54	0	0	0	0	1	0	0	0
CDFG_55	0	0	0	0	0	0	0	0
CDFG_56	0	0	0	0	0	0	1	0
CDFG_70	0	0	0	0	0	0	0	0
CDFG_71	0	0	0	0	0	0	0	0
HF_41	0	0	0	0	0	0	0	0
HF_42	0	0	1	0	0	0	0	0
HF_43	0	0	0	0	0	0	0	0
HF_44	0	0	0	0	0	0	0	0
HF_57	1	0	0	1	0	0	0	0
HF_58	0	0	0	1	0	0	0	0
HF_59	0	0	0	0	0	0	0	0
HF_60	0	0	0	0	0	0	0	0
HF_73	0	0	0	0	0	0	0	0
HF_74	0	0	0	0	0	0	0	0
HF_75	0	0	0	0	0	0	0	0

Kingdom	Bacteria	Bacteria	Bacteria	Bacteria	Bacteria	Bacteria	Bacteria	Bacteria
Phylum	Proteobacteria	Firmicutes	Firmicutes	Firmicutes	Actinobacteria	Firmicutes	Firmicutes	Bacteroidetes
Class	Alphaproteobacteria	Erysipelotrichia	Clostridia	Clostridia	Actinobacteria	Clostridia	Clostridia	Bacteroidia
Order	Rhizobiales	Erysipelotrichales	Clostridiales	Clostridiales	Coriobacteriales	Clostridiales	Clostridiales	Bacteroidales
Family	Bradyrhizobiaceae	Erysipelotrichaceae	Clostridiaceae_	Lachnospiraceae	Coriobacteriaceae	Lachnospiraceae	Lachnospiraceae	Porphyromonadaceae
Genus	Bradyrhizobium	Erysipelotrichaceae_incertae_sedis	Alkaliphilus	Clostridium_XlVa	Enterorhabdus	Clostridium_XlVa	Clostridium_XlVa	Parabacteroides
#NAME	ZOTU_0432	ZOTU_0433	ZOTU_0434	ZOTU_0435	ZOTU_0436	ZOTU_0437	ZOTU_0438	ZOTU_0439
HFFG_45	0	0	0	0	0	0	1	0
HFFG_46	0	0	0	0	0	0	0	0
HFFG_47	0	0	0	0	0	0	0	0
HFFG_61	0	0	0	0	0	0	0	0
HFFG_62	0	0	0	0	0	0	0	0
HFFG_63	0	0	0	0	0	0	0	0
HFFG_64	0	0	0	0	0	0	0	0
HFFG_76	0	0	0	1	1	2	5	0

Kingdom	Bacteria	Bacteria	Bacteria	Bacteria	Bacteria	Bacteria	Bacteria	Bacteria
Phylum	Firmicutes	Firmicutes	Firmicutes	Firmicutes	Firmicutes	Actinobacteria	Firmicutes	Actinobacteria
Class	Clostridia	Clostridia	Bacilli	Clostridia	Bacilli	Actinobacteria	Clostridia	Actinobacteria
Order	Clostridiales	Clostridiales	Lactobacillales	Clostridiales	Lactobacillales	Coriobacteriales	Clostridiales	Actinomycetales
Family	Lachnospiraceae	Peptostreptococcaceae	Enterococcaceae	Clostridiales_Incertae_Sedis_XII	Aerococcaceae	Coriobacteriaceae	Lachnospiraceae	Micrococcaceae
Genus	Clostridium_XlVa	Romboutsia	Enterococcus	Guggenheimella	Abiotrophia	Senegalimassilia	Blautia	Micrococcus
#NAME	ZOTU_0440	ZOTU_0441	ZOTU_0442	ZOTU_0443	ZOTU_0444	ZOTU_0445	ZOTU_0446	ZOTU_0447
CD_49	0	0	0	0	0	0	0	0
CD_50	0	0	5	0	0	0	0	0
CD_51	0	38	13	0	0	4	0	0
CD_52	0	0	1	0	0	1	0	0
CD_65	0	0	0	0	0	0	0	0
CD_66	0	0	3	0	0	0	0	0
CD_67	0	0	0	0	0	0	0	0
CD_68	0	0	4	0	0	0	0	0
CD_78	0	1	1	0	0	0	0	0
CD_79	0	0	0	0	0	0	0	0
CD_80	0	0	0	0	0	0	0	0
CDFG_54	0	4	1	0	0	0	0	0
CDFG_55	0	9	4	0	0	2	0	0
CDFG_56	0	4	1	0	0	0	0	0
CDFG_70	0	0	26	0	0	1	0	0
CDFG_71	0	0	0	0	0	0	0	0
HF_41	0	0	1	0	0	0	0	0
HF_42	0	0	1	0	0	1	0	0
HF_43	0	0	1	0	0	1	0	0
HF_44	0	1	4	0	0	3	0	0
HF_57	0	0	1	0	0	0	0	0
HF_58	0	0	3	0	0	0	0	0
HF_59	0	0	1	0	0	0	0	0
HF_60	0	0	1	0	0	0	0	0
HF_73	0	0	0	0	0	0	1	0
HF_74	0	0	7	0	0	1	0	0
HF_75	0	1	0	0	0	0	1	0

Kingdom	Bacteria	Bacteria	Bacteria	Bacteria	Bacteria	Bacteria	Bacteria	Bacteria
Phylum	Firmicutes	Firmicutes	Firmicutes	Firmicutes	Firmicutes	Actinobacteria	Firmicutes	Actinobacteria
Class	Clostridia	Clostridia	Bacilli	Clostridia	Bacilli	Actinobacteria	Clostridia	Actinobacteria
Order	Clostridiales	Clostridiales	Lactobacillales	Clostridiales	Lactobacillales	Coriobacteriales	Clostridiales	Actinomycetales
Family	Lachnospiraceae	Peptostreptococcaceae	Enterococcaceae	Clostridiales_Incertae_Sedis_XII	Aerococcaceae	Coriobacteriaceae	Lachnospiraceae	Micrococcaceae
Genus	Clostridium_XlVa	Romboutsia	Enterococcus	Guggenheimella	Abiotrophia	Senegalimassilia	Blautia	Micrococcus
#NAME	ZOTU_0440	ZOTU_0441	ZOTU_0442	ZOTU_0443	ZOTU_0444	ZOTU_0445	ZOTU_0446	ZOTU_0447
HFFG_45	0	0	0	0	0	0	0	0
HFFG_46	0	1	1	0	0	0	0	0
HFFG_47	0	0	0	0	0	0	0	0
HFFG_61	0	0	0	0	0	0	0	0
HFFG_62	0	0	3	0	0	0	0	0
HFFG_63	0	0	2	0	0	0	0	0
HFFG_64	0	0	3	0	0	0	0	0
HFFG_76	0	0	12	0	0	0	2	0

Kingdom	Bacteria	Bacteria	Bacteria	Bacteria	Bacteria	Bacteria	Bacteria	Bacteria
Phylum	Firmicutes	Actinobacteria	Proteobacteria	Firmicutes	Actinobacteria	Actinobacteria	Firmicutes	Firmicutes
Class	Clostridia	Actinobacteria	Gammaproteobacteria	Clostridia	Actinobacteria	Actinobacteria	Clostridia	Bacilli
Order	Clostridiales	Coriobacteriales	Legionellales	Clostridiales	Actinomycetales	Coriobacteriales	Clostridiales	Bacillales
Family	Lachnospiraceae	Coriobacteriaceae	Legionellaceae	Lachnospiraceae	Micrococcaceae	Coriobacteriaceae	Peptostreptococcaceae	Paenibacillaceae_
Genus	Clostridium_XlVa	Enterorhabdus	Legionella	Clostridium_XlVa	Rothia	Enterorhabdus	Clostridium_XI	Paenibacillus
#NAME	ZOTU_0448	ZOTU_0449	ZOTU_0450	ZOTU_0451	ZOTU_0452	ZOTU_0453	ZOTU_0454	ZOTU_0455
CD_49	0	0	0	0	0	3	1	0
CD_50	0	0	0	0	0	1	0	0
CD_51	0	0	0	0	0	1	0	0
CD_52	0	0	0	0	0	1	0	0
CD_65	0	0	0	0	0	0	0	0
CD_66	0	0	0	0	0	0	0	0
CD_67	0	0	0	0	0	0	0	0
CD_68	0	0	0	0	0	2	0	0
CD_78	0	0	0	0	0	0	0	0
CD_79	0	0	0	0	0	0	0	0
CD_80	0	0	0	0	0	0	0	0
CDFG_54	0	0	0	0	0	0	0	0
CDFG_55	0	0	0	0	0	2	0	0
CDFG_56	0	1	0	0	0	0	0	0
CDFG_70	0	0	0	0	0	0	0	0
CDFG_71	0	0	0	0	0	0	0	0
HF_41	0	0	0	0	0	0	0	0
HF_42	0	0	0	0	0	0	0	0
HF_43	0	0	0	0	0	0	0	0
HF_44	0	0	0	0	0	0	0	0
HF_57	0	0	0	0	0	0	0	0
HF_58	0	0	0	0	0	0	0	0
HF_59	0	0	0	0	0	0	0	0
HF_60	0	0	0	0	0	0	0	0
HF_73	0	1	0	0	0	1	0	0
HF_74	0	0	0	0	0	0	0	0
HF_75	0	0	0	0	0	0	0	0

Kingdom	Bacteria	Bacteria	Bacteria	Bacteria	Bacteria	Bacteria	Bacteria	Bacteria
Phylum	Firmicutes	Actinobacteria	Proteobacteria	Firmicutes	Actinobacteria	Actinobacteria	Firmicutes	Firmicutes
Class	Clostridia	Actinobacteria	Gammaproteobacteria	Clostridia	Actinobacteria	Actinobacteria	Clostridia	Bacilli
Order	Clostridiales	Coriobacteriales	Legionellales	Clostridiales	Actinomycetales	Coriobacteriales	Clostridiales	Bacillales
Family	Lachnospiraceae	Coriobacteriaceae	Legionellaceae	Lachnospiraceae	Micrococcaceae	Coriobacteriaceae	Peptostreptococcaceae	Paenibacillaceae_
Genus	Clostridium_XlVa	Enterorhabdus	Legionella	Clostridium_XlVa	Rothia	Enterorhabdus	Clostridium_XI	Paenibacillus
#NAME	ZOTU_0448	ZOTU_0449	ZOTU_0450	ZOTU_0451	ZOTU_0452	ZOTU_0453	ZOTU_0454	ZOTU_0455
HFFG_45	0	0	0	0	0	1	0	0
HFFG_46	0	0	0	0	0	0	0	0
HFFG_47	0	0	0	0	0	0	0	0
HFFG_61	0	0	0	0	0	0	0	0
HFFG_62	0	0	0	0	0	0	0	0
HFFG_63	0	0	0	0	0	0	0	0
HFFG_64	0	0	0	0	0	0	0	0
HFFG_76	0	0	0	0	0	0	0	0

Kingdom	Bacteria	Bacteria	Bacteria	Bacteria	Bacteria
Phylum	Firmicutes	Firmicutes	Firmicutes	Firmicutes	Firmicutes
Class	Bacilli	Bacilli	Clostridia	Bacilli	Erysipelotrichia
Order	Bacillales	Bacillales	Clostridiales	Bacillales	Erysipelotrichales
Family	Paenibacillaceae_	Bacillaceae_	Clostridiaceae_	Paenibacillaceae_	Erysipelotrichaceae
Genus	Paenibacillus	Virgibacillus	Alkaliphilus	Paenibacillus	Kandleria
#NAME	ZOTU_0456	ZOTU_0457	ZOTU_0458	ZOTU_0459	ZOTU_0460
CD_49	0	0	0	0	0
CD_50	0	0	0	0	0
CD_51	0	0	0	0	0
CD_52	0	0	0	0	0
CD_65	0	0	0	0	0
CD_66	0	0	0	0	0
CD_67	0	0	0	0	0
CD_68	0	0	0	0	0
CD_78	0	0	0	0	0
CD_79	0	0	0	0	0
CD_80	0	0	0	0	0
CDFG_54	0	0	0	0	0
CDFG_55	0	0	0	0	0
CDFG_56	0	0	0	0	0
CDFG_70	0	0	0	0	0
CDFG_71	0	0	0	0	0
HF_41	0	0	0	0	0
HF_42	0	0	0	0	0
HF_43	0	0	0	0	0
HF_44	0	0	0	0	0
HF_57	0	0	0	0	0
HF_58	0	0	0	0	0
HF_59	0	0	0	0	0
HF_60	0	0	0	0	0
HF_73	0	0	0	0	0
HF_74	0	0	0	0	0
HF_75	0	0	0	0	0

Kingdom	Bacteria	Bacteria	Bacteria	Bacteria	Bacteria
Phylum	Firmicutes	Firmicutes	Firmicutes	Firmicutes	Firmicutes
Class	Bacilli	Bacilli	Clostridia	Bacilli	Erysipelotrichia
Order	Bacillales	Bacillales	Clostridiales	Bacillales	Erysipelotrichales
Family	Paenibacillaceae_	Bacillaceae_	Clostridiaceae_	Paenibacillaceae_	Erysipelotrichaceae
Genus	Paenibacillus	Virgibacillus	Alkaliphilus	Paenibacillus	Kandleria
#NAME	ZOTU_0456	ZOTU_0457	ZOTU_0458	ZOTU_0459	ZOTU_0460
HFFG_45	0	0	0	0	0
HFFG_46	0	0	0	0	0
HFFG_47	0	0	0	0	0
HFFG_61	13	0	0	0	0
HFFG_62	1	0	0	0	0
HFFG_63	0	0	0	0	0
HFFG_64	0	0	0	0	0
HFFG_76	0	0	0	0	0

Table S14 (pages S-235 to S-344). Raw OTU count data from 16S sequencing of colon contents.

Kingdom	Bacteria	Bacteria	Bacteria	Bacteria	Bacteria	Bacteria	Bacteria	Bacteria	Bacteria
Phylum	Firmicutes	Verrucomicrobia	Firmicutes	Firmicutes	Firmicutes	Firmicutes	Actinobacteria	Firmicutes	Firmicutes
Class	Erysipelotrichia	Verrucomicrobiae	Bacilli	Erysipelotrichia	Bacilli	Bacilli	Actinobacteria	Bacilli	Bacilli
Order	Erysipelotrichales	Verrucomicrobiales	Lactobacillales	Erysipelotrichales	Bacillales	Lactobacillales	Bifidobacteriales	Bacillales	Bacillales
Family	Erysipelotrichaceae	Verrucomicrobiaceae	Enterococcaceae	Erysipelotrichaceae	Staphylococcaceae	Lactobacillaceae	Bifidobacteriaceae	Bacillaceae_	Staphylococcaceae
Genus	Allobaculum	Akkermansia	Enterococcus	Catenisphaera	Staphylococcus	Lactobacillus	Bifidobacterium	Bacillus	Staphylococcus
#NAME	ZOTU_0001	ZOTU_0002	ZOTU_0003	ZOTU_0004	ZOTU_0005	ZOTU_0006	ZOTU_0007	ZOTU_0008	ZOTU_0009
CD_49	18631	11143	4083	14587	363	2314	4105	7	0
CD_50	68308	15540	11234	15416	134	1063	18529	8	0
CD_51	42088	38323	19915	968	977	707	4767	27	0
CD_52	87163	15725	28336	24310	31	2384	13523	24	2
CD_65	21016	11181	4936	11278	20	372	4647	3854	0
CD_66	19750	8274	3124	13102	23	339	5275	16699	0
CD_67	16385	9276	3685	8461	1443	808	7352	9	0
CD_68	22263	5299	9263	16493	62	270	3724	55	0
CD_78	32465	15111	4922	7135	80	2	5544	0	2
CD_79	4603	3678	1487	511	96	11	596	1	1
CD_80	31350	10874	6065	5218	140	102	5750	39	4
CDFG_53	12173	7543	1711	448	781	9	711	4	3
CDFG_54	41860	70222	34558	4034	22	23	1336	1	1
CDFG_55	44960	32737	22202	5434	170	52	5416	167	6
CDFG_56	7275	8027	2540	811	8	95	1064	14568	30
CDFG_69	41095	882	3844	4876	155	31	4693	7	0
CDFG_70	8662	5324	4069	29	32	19	1282	4	0
CDFG_71	31418	2930	8292	4972	242	45	4396	4	2
HF_41	21540	4092	17130	32524	25	1458	526	64	1
HF_42	14228	35428	34128	2745	157	182	1132	94	6
HF_43	50147	42	24965	70611	32	27483	21	10	1
HF_44	37978	331	26933	56950	129	20995	17	23	16
HF_57	7733	2660	17726	2999	196	52	83	65	0
HF_58	9860	19558	23596	12117	287	1677	129	1350	0
HF_59	1612	6431	4120	1039	2	322	10	51647	0
HF_60	4132	12456	16679	1288	0	900	5	326	0
HF_72	15039	267	9086	14591	173	7657	362	58	2

Kingdom	Bacteria	Bacteria	Bacteria	Bacteria	Bacteria	Bacteria	Bacteria	Bacteria	Bacteria
Phylum	Firmicutes	Verrucomicrobia	Firmicutes	Firmicutes	Firmicutes	Firmicutes	Actinobacteria	Firmicutes	Firmicutes
Class	Erysipelotrichia	Verrucomicrobiae	Bacilli	Erysipelotrichia	Bacilli	Bacilli	Actinobacteria	Bacilli	Bacilli
Order	Erysipelotrichales	Verrucomicrobiales	Lactobacillales	Erysipelotrichales	Bacillales	Lactobacillales	Bifidobacteriales	Bacillales	Bacillales
Family	Erysipelotrichaceae	Verrucomicrobiaceae	Enterococcaceae	Erysipelotrichaceae	Staphylococcaceae	Lactobacillaceae	Bifidobacteriaceae	Bacillaceae_	Staphylococcaceae
Genus	Allobaculum	Akkermansia	Enterococcus	Catenisphaera	Staphylococcus	Lactobacillus	Bifidobacterium	Bacillus	Staphylococcus
#NAME	ZOTU_0001	ZOTU_0002	ZOTU_0003	ZOTU_0004	ZOTU_0005	ZOTU_0006	ZOTU_0007	ZOTU_0008	ZOTU_0009
HF_73	20155	152	9099	17197	609	8858	340	51	1
HF_74	17232	76	933	16001	56	11841	189	196	0
HFFG_45	5017	2558	3159	3742	4	1119	58	0	2476
HFFG_46	2238	4655	942	1126	5	133	5	0	13676
HFFG_47	5638	2529	898	5083	1	883	172	16	3694
HFFG_48	6481	3376	328	4221	4	1738	453	5811	406
HFFG_61	10274	759	3482	3538	3	839	61	2	0
HFFG_62	7356	4899	4513	2766	290	1970	124	8	1
HFFG_63	6499	2604	2045	3073	20	2686	64	13	1
HFFG_64	4749	7888	6236	2223	48	1936	58	759	19
HFFG_75	13675	18467	8785	2289	78	11661	14	42326	0
HFFG_76	16113	18566	15301	4239	1166	4029	14	9674	0
HFFG_77	7459	4137	6669	1450	343	1679	12	13	0

Kingdom	Bacteria	Bacteria	Bacteria	Bacteria	Bacteria	Bacteria	Bacteria	Bacteria
Phylum	Firmicutes	Firmicutes	Bacteroidetes	Firmicutes	Firmicutes	Actinobacteria	Firmicutes	Firmicutes
Class	Clostridia	Bacilli	Bacteroidia	Clostridia	Clostridia	Actinobacteria	Bacilli	Clostridia
Order	Clostridiales	Lactobacillales	Bacteroidales	Clostridiales	Clostridiales	Coriobacteriales	Bacillales	Clostridiales
Family	Peptostreptococcaceae	Streptococcaceae	Bacteroidaceae	Lachnospiraceae	Lachnospiraceae	Coriobacteriaceae	Staphylococcaceae	Lachnospiraceae
Genus	Romboutsia	Lactococcus	Bacteroides	Clostridium_XlVa	Acetatifactor	Enterorhabdus	Staphylococcus	Clostridium_XlVa
#NAME	ZOTU_0010	ZOTU_0011	ZOTU_0012	ZOTU_0013	ZOTU_0014	ZOTU_0015	ZOTU_0016	ZOTU_0017
CD_49	381	50	1095	8	112	324	0	20
CD_50	22	238	1339	5	301	1667	0	0
CD_51	1626	87	9891	5	942	2345	0	61
CD_52	26	109	571	2	38	2090	0	24
CD_65	11	31	611	311	74	167	0	0
CD_66	9	61	1347	5	102	238	0	0
CD_67	73	12	2320	1	139	145	0	0
CD_68	24	62	227	0	571	91	0	0
CD_78	120	63	469	0	2	666	0	6
CD_79	69	8	284	2	2	101	0	1
CD_80	247	88	115	1	25	1100	0	6
CDFG_53	178	14	1	0	1	61	0	26
CDFG_54	3664	178	27	143	11	814	1	811
CDFG_55	2062	171	17	0	127	742	0	1124
CDFG_56	272	22	8	1	11	113	0	88
CDFG_69	253	38	7	0	61	47	0	99
CDFG_70	368	53	6	1	26	118	0	316
CDFG_71	105	32	0	3	24	124	0	91
HF_41	1179	115	681	219	29	2642	0	3
HF_42	28	309	58	9101	218	2990	1	106
HF_43	990	223	29	1245	354	3455	0	46
HF_44	694	80	5	971	97	1074	0	21
HF_57	693	107	13	133	630	302	0	37
HF_58	1908	312	81	186	3596	711	0	238
HF_59	438	131	41	56	1716	91	0	67
HF_60	2074	110	3	159	1138	194	0	25
HF_72	431	29	210	212	312	698	2	28

Kingdom	Bacteria	Bacteria	Bacteria	Bacteria	Bacteria	Bacteria	Bacteria	Bacteria
Phylum	Firmicutes	Firmicutes	Bacteroidetes	Firmicutes	Firmicutes	Actinobacteria	Firmicutes	Firmicutes
Class	Clostridia	Bacilli	Bacteroidia	Clostridia	Clostridia	Actinobacteria	Bacilli	Clostridia
Order	Clostridiales	Lactobacillales	Bacteroidales	Clostridiales	Clostridiales	Coriobacteriales	Bacillales	Clostridiales
Family	Peptostreptococcaceae	Streptococcaceae	Bacteroidaceae	Lachnospiraceae	Lachnospiraceae	Coriobacteriaceae	Staphylococcaceae	Lachnospiraceae
Genus	Romboutsia	Lactococcus	Bacteroides	Clostridium_XlVa	Acetatifactor	Enterorhabdus	Staphylococcus	Clostridium_XlVa
#NAME	ZOTU_0010	ZOTU_0011	ZOTU_0012	ZOTU_0013	ZOTU_0014	ZOTU_0015	ZOTU_0016	ZOTU_0017
HF_73	581	30	3	19	99	207	125	0
HF_74	1034	39	15	614	928	190	1	6
HFFG_45	594	71	270	6	98	542	1	76
HFFG_46	352	9	4	2	20	75	0	229
HFFG_47	117	18	3	49	2	220	0	48
HFFG_48	179	8	7	0	32	363	6	64
HFFG_61	137	11	2	67	23	47	0	87
HFFG_62	400	124	10	24	54	210	0	248
HFFG_63	499	24	3	22	20	65	0	143
HFFG_64	1054	31	4	5	1265	83	34	2726
HFFG_75	2306	480	64	254	50	601	0	2110
HFFG_76	1658	82	93	1211	72	953	0	1249
HFFG_77	338	17	2	103	121	481	0	149

Kingdom	Bacteria	Bacteria	Bacteria	Bacteria	Bacteria	Bacteria	Bacteria	Bacteria
Phylum	Firmicutes	Firmicutes	Bacteroidetes	Firmicutes	Firmicutes	Bacteroidetes	Proteobacteria	Firmicutes
Class	Clostridia	Clostridia	Bacteroidia	Erysipelotrichia	Clostridia	Bacteroidia	Betaproteobacteria	Clostridia
Order	Clostridiales	Clostridiales	Bacteroidales	Erysipelotrichales	Clostridiales	Bacteroidales	Burkholderiales	Clostridiales
Family	Clostridiaceae_	Peptostreptococcaceae	Porphyromonadaceae	Erysipelotrichaceae	Ruminococcaceae	Rikenellaceae	Sutterellaceae	Lachnospiraceae
Genus	Clostridium_sensu_stricto	Romboutsia	Barnesiella	Allobaculum	Pseudoflavonifractor	Alistipes	Parasutterella	Clostridium_XlVa
#NAME	ZOTU_0018	ZOTU_0019	ZOTU_0020	ZOTU_0021	ZOTU_0022	ZOTU_0023	ZOTU_0024	ZOTU_0025
CD_49	41	104	1657	0	70	833	313	74
CD_50	16	9	1479	0	579	310	474	36
CD_51	5084	381	3103	0	776	512	232	137
CD_52	3	5	268	0	54	1476	174	75
CD_65	26	1	228	0	192	267	66	16
CD_66	7	1	61	0	42	840	164	37
CD_67	14	37	909	0	37	860	142	0
CD_68	57	10	38	0	101	410	222	0
CD_78	296	25	255	0	14	531	88	0
CD_79	42	18	136	0	7	82	23	3
CD_80	454	69	78	0	40	318	19	2
CDFG_53	373	92	14	0	10	93	3	15
CDFG_54	2280	1709	88	0	1013	567	19	7
CDFG_55	2524	847	54	0	187	740	30	222
CDFG_56	456	119	40	0	25	177	2	18
CDFG_69	13	69	345	0	21	111	34	3
CDFG_70	0	76	176	0	60	234	2	25
CDFG_71	10	40	87	0	27	348	6	9
HF_41	93	474	31	0	71	13	7	13
HF_42	26	6	50	0	129	191	27	240
HF_43	18	380	0	0	96	26	39	128
HF_44	41	303	0	0	55	7	2	134
HF_57	1	301	8	0	158	32	18	195
HF_58	27	892	20	0	852	131	109	660
HF_59	11	205	10	0	190	62	84	163
HF_60	66	919	0	0	397	4	44	413
HF_72	4	199	10	0	239	5	2	83

Kingdom	Bacteria	Bacteria	Bacteria	Bacteria	Bacteria	Bacteria	Bacteria	Bacteria
Phylum	Firmicutes	Firmicutes	Bacteroidetes	Firmicutes	Firmicutes	Bacteroidetes	Proteobacteria	Firmicutes
Class	Clostridia	Clostridia	Bacteroidia	Erysipelotrichia	Clostridia	Bacteroidia	Betaproteobacteria	Clostridia
Order	Clostridiales	Clostridiales	Bacteroidales	Erysipelotrichales	Clostridiales	Bacteroidales	Burkholderiales	Clostridiales
Family	Clostridiaceae_	Peptostreptococcaceae	Porphyromonadaceae	Erysipelotrichaceae	Ruminococcaceae	Rikenellaceae	Sutterellaceae	Lachnospiraceae
Genus	Clostridium_sensu_stricto	Romboutsia	Barnesiella	Allobaculum	Pseudoflavonifractor	Alistipes	Parasutterella	Clostridium_XlVa
#NAME	ZOTU_0018	ZOTU_0019	ZOTU_0020	ZOTU_0021	ZOTU_0022	ZOTU_0023	ZOTU_0024	ZOTU_0025
HF_73	4	240	2	0	13	4	4	85
HF_74	1	454	27	0	63	10	32	324
HFFG_45	283	282	128	0	35	84	2	0
HFFG_46	57	156	72	0	102	18	3	157
HFFG_47	19	33	32	0	6	7	3	11
HFFG_48	34	87	47	0	24	10	1	16
HFFG_61	9	79	7	0	63	10	2	427
HFFG_62	23	171	181	0	63	135	8	172
HFFG_63	9	209	12	0	73	20	3	42
HFFG_64	109	485	62	0	311	6	39	360
HFFG_75	261	1031	800	0	326	404	15	831
HFFG_76	385	699	505	0	379	231	69	495
HFFG_77	91	135	53	0	82	17	3	6

Kingdom	Bacteria	Bacteria	Bacteria	Bacteria	Bacteria	Bacteria	Bacteria	Bacteria
Phylum	Actinobacteria	Firmicutes	Bacteroidetes	Firmicutes	Firmicutes	Firmicutes	Bacteroidetes	Firmicutes
Class	Actinobacteria	Clostridia	Bacteroidia	Clostridia	Bacilli	Erysipelotrichia	Bacteroidia	Clostridia
Order	Coriobacteriales	Clostridiales	Bacteroidales	Clostridiales	Bacillales	Erysipelotrichales	Bacteroidales	Clostridiales
Family	Coriobacteriaceae	Eubacteriaceae	Porphyromonadaceae	Ruminococcaceae	Planococcaceae	Erysipelotrichaceae	Porphyromonadaceae	Lachnospiraceae
Genus	Enterorhabdus	Eubacterium	Parabacteroides	Pseudoflavonifractor	Lysinibacillus	Turicibacter	Barnesiella	Clostridium_XlVa
#NAME	ZOTU_0026	ZOTU_0027	ZOTU_0028	ZOTU_0029	ZOTU_0030	ZOTU_0031	ZOTU_0032	ZOTU_0033
CD_49	393	0	1	1	0	0	7	21
CD_50	72	0	0	12	0	0	3	694
CD_51	83	1	0	48	1	0	5	317
CD_52	213	0	2	0	0	0	2	227
CD_65	42	0	7	9	1	0	1	775
CD_66	35	0	142	3	1	0	1	25
CD_67	152	0	0	0	1	0	2	39
CD_68	59	0	92	1	1	0	0	44
CD_78	15	0	0	2	3	0	0	23
CD_79	2	0	0	1	361	0	0	7
CD_80	33	0	1	7	82	1	2	104
CDFG_53	13	0	0	0	0	0	145	3
CDFG_54	94	134	0	88	0	1	885	53
CDFG_55	176	6	0	22	10	4	374	69
CDFG_56	21	0	2	6	2	2	20	9
CDFG_69	45	0	0	1	0	335	0	7
CDFG_70	20	0	2	8	1	430	0	34
CDFG_71	52	0	0	8	23	56	0	85
HF_41	288	3	2	519	0	2	299	148
HF_42	72	3	0	8438	0	2	1177	1906
HF_43	173	0	0	1327	0	0	323	1263
HF_44	81	0	1	601	0	1	78	363
HF_57	133	0	0	3	0	0	105	11
HF_58	264	0	0	21	0	0	916	13
HF_59	13	0	0	5	0	0	654	8
HF_60	33	0	0	29	0	0	119	4
HF_72	1538	0	1	9	8940	0	346	69

Kingdom	Bacteria	Bacteria	Bacteria	Bacteria	Bacteria	Bacteria	Bacteria	Bacteria
Phylum	Actinobacteria	Firmicutes	Bacteroidetes	Firmicutes	Firmicutes	Firmicutes	Bacteroidetes	Firmicutes
Class	Actinobacteria	Clostridia	Bacteroidia	Clostridia	Bacilli	Erysipelotrichia	Bacteroidia	Clostridia
Order	Coriobacteriales	Clostridiales	Bacteroidales	Clostridiales	Bacillales	Erysipelotrichales	Bacteroidales	Clostridiales
Family	Coriobacteriaceae	Eubacteriaceae	Porphyromonadaceae	Ruminococcaceae	Planococcaceae	Erysipelotrichaceae	Porphyromonadaceae	Lachnospiraceae
Genus	Enterorhabdus	Eubacterium	Parabacteroides	Pseudoflavonifractor	Lysinibacillus	Turicibacter	Barnesiella	Clostridium_XlVa
#NAME	ZOTU_0026	ZOTU_0027	ZOTU_0028	ZOTU_0029	ZOTU_0030	ZOTU_0031	ZOTU_0032	ZOTU_0033
HF_73	242	0	0	3	84	0	49	30
HF_74	184	0	0	7	23	2	491	148
HFFG_45	15	1	0	6	0	7	1	4
HFFG_46	1	73	0	11	0	9	0	10
HFFG_47	6	1	1	0	0	1	0	0
HFFG_48	10	4	1	0	4	1	0	8
HFFG_61	7	0	9	3	1	5	0	29
HFFG_62	57	1	68	5	1	16	0	20
HFFG_63	10	0	13	3	4	11	3	13
HFFG_64	15	5	35	30	85	117	2	15
HFFG_75	25	0	0	18	5547	280	3	6
HFFG_76	67	0	0	36	6	106	0	26
HFFG_77	27	0	0	6	0	37	0	17

Kingdom	Bacteria	Bacteria	Bacteria	Bacteria	Bacteria	Bacteria	Bacteria	Bacteria	Bacteria
Phylum	Firmicutes	Actinobacteria	Bacteroidetes	Firmicutes	Firmicutes	Firmicutes	Actinobacteria	Firmicutes	Firmicutes
Class	Erysipelotrichia	Actinobacteria	Bacteroidia	Clostridia	Clostridia	Clostridia	Actinobacteria	Clostridia	Clostridia
Order	Erysipelotrichales	Coriobacteriales	Bacteroidales	Clostridiales	Clostridiales	Clostridiales	Coriobacteriales	Clostridiales	Clostridiales
Family	Erysipelotrichaceae	Coriobacteriaceae	Porphyromonadaceae	Clostridiaceae_	Lachnospiraceae	Lachnospiraceae	Coriobacteriaceae	Lachnospiraceae	Lachnospiraceae
Genus	Clostridium_XVIII	Parvibacter	Barnesiella	Alkaliphilus	Clostridium_XlVa	Clostridium_XlVa	Senegalimassilia	Clostridium_XlVa	Clostridium_XlVa
#NAME	ZOTU_0034	ZOTU_0035	ZOTU_0036	ZOTU_0037	ZOTU_0038	ZOTU_0039	ZOTU_0040	ZOTU_0041	ZOTU_0042
CD_49	101	24	688	0	5	2	1	27	18
CD_50	46	470	509	0	13	7	25	116	72
CD_51	128	115	994	1	161	1	169	64	167
CD_52	69	140	294	0	10	1	35	30	8
CD_65	138	15	57	0	15	3	9	7	44
CD_66	25	38	128	0	4	4	16	10	14
CD_67	41	9	1041	0	2	2	7	14	35
CD_68	41	30	78	0	9	0	6	32	45
CD_78	476	44	32	0	3	1	20	3	20
CD_79	58	9	11	0	2	1	14	0	1
CD_80	152	47	5	0	9	0	57	7	49
CDFG_53	38	83	1	0	6	0	2	16	4
CDFG_54	245	471	130	0	345	16	81	1076	12
CDFG_55	237	674	23	0	57	22	137	235	4
CDFG_56	22	33	9	0	15	3	14	73	3
CDFG_69	23	131	31	0	7	0	24	18	0
CDFG_70	93	208	19	0	63	0	32	63	1
CDFG_71	22	103	26	0	7	1	85	32	17
HF_41	14	603	4	0	11	0	75	6	0
HF_42	128	143	20	0	532	29	515	250	0
HF_43	47	524	27	0	136	6	207	119	0
HF_44	17	264	3	0	60	2	211	44	0
HF_57	207	43	25	0	105	0	10	109	316
HF_58	349	156	72	0	266	13	43	392	975
HF_59	114	22	98	0	155	3	8	77	162
HF_60	136	77	10	0	160	1	12	85	96
HF_72	31	201	6	0	239	4	166	5	15

Kingdom	Bacteria	Bacteria	Bacteria	Bacteria	Bacteria	Bacteria	Bacteria	Bacteria	Bacteria
Phylum	Firmicutes	Actinobacteria	Bacteroidetes	Firmicutes	Firmicutes	Firmicutes	Actinobacteria	Firmicutes	Firmicutes
Class	Erysipelotrichia	Actinobacteria	Bacteroidia	Clostridia	Clostridia	Clostridia	Actinobacteria	Clostridia	Clostridia
Order	Erysipelotrichales	Coriobacteriales	Bacteroidales	Clostridiales	Clostridiales	Clostridiales	Coriobacteriales	Clostridiales	Clostridiales
Family	Erysipelotrichaceae	Coriobacteriaceae	Porphyromonadaceae	Clostridiaceae_	Lachnospiraceae	Lachnospiraceae	Coriobacteriaceae	Lachnospiraceae	Lachnospiraceae
Genus	Clostridium_XVIII	Parvibacter	Barnesiella	Alkaliphilus	Clostridium_XlVa	Clostridium_XlVa	Senegalimassilia	Clostridium_XlVa	Clostridium_XlVa
#NAME	ZOTU_0034	ZOTU_0035	ZOTU_0036	ZOTU_0037	ZOTU_0038	ZOTU_0039	ZOTU_0040	ZOTU_0041	ZOTU_0042
HF_73	18	42	7	0	42	3	31	35	34
HF_74	43	54	9	0	103	4	37	59	222
HFFG_45	42	35	4	0	2	0	11	2	7
HFFG_46	30	12	32	0	6	0	2	12	62
HFFG_47	31	15	1	0	0	0	3	4	6
HFFG_48	6	20	13	0	4	0	12	35	13
HFFG_61	36	11	13	0	2	0	0	29	30
HFFG_62	105	65	21	0	5	0	2	29	8
HFFG_63	64	8	3	0	2	0	0	55	19
HFFG_64	25	16	115	0	34	4	2	129	37
HFFG_75	719	0	162	0	8	2	62	46	53
HFFG_76	582	0	236	0	35	5	68	129	80
HFFG_77	182	0	13	0	6	1	13	18	40

Kingdom	Bacteria	Bacteria	Bacteria	Bacteria	Bacteria	Bacteria	Bacteria	Bacteria	Bacteria
Phylum	Firmicutes	Firmicutes	Actinobacteria	Firmicutes	Firmicutes	Firmicutes	Firmicutes	Bacteroidetes	Firmicutes
Class	Clostridia	Clostridia	Actinobacteria	Bacilli	Clostridia	Clostridia	Clostridia	Bacteroidia	Clostridia
Order	Clostridiales	Clostridiales	Coriobacteriales	Bacillales	Clostridiales	Clostridiales	Clostridiales	Bacteroidales	Clostridiales
Family	Lachnospiraceae	Lachnospiraceae	Coriobacteriaceae	Staphylococcaceae	Lachnospiraceae	Ruminococcaceae	Lachnospiraceae	Porphyromonadaceae	Clostridiaceae_
Genus	Clostridium_XlVa	Blautia	Asaccharobacter	Staphylococcus	Acetatifactor	Oscillibacter	Clostridium_XlVa	Parabacteroides	Alkaliphilus
#NAME	ZOTU_0043	ZOTU_0044	ZOTU_0045	ZOTU_0046	ZOTU_0047	ZOTU_0048	ZOTU_0049	ZOTU_0050	ZOTU_0051
CD_49	1	38	0	1	75	19	23	0	99
CD_50	4	78	0	10	8	9	30	0	153
CD_51	6	41	0	96	208	166	69	0	32
CD_52	1	126	0	0	19	6	22	0	105
CD_65	8	0	0	0	35	5	38	131	49
CD_66	0	2	0	0	20	5	8	231	11
CD_67	0	6	0	2	80	17	17	729	31
CD_68	3	3	0	0	24	17	19	190	9
CD_78	3	7	0	0	0	1	8	58	67
CD_79	0	0	0	0	1	1	1	48	10
CD_80	1	16	0	0	2	5	8	44	14
CDFG_53	3	176	0	0	3	4	2	0	14
CDFG_54	755	550	0	0	127	95	241	0	165
CDFG_55	283	550	0	0	20	19	14	0	48
CDFG_56	123	39	1	0	29	6	16	0	38
CDFG_69	1	258	0	0	3	6	14	1	11
CDFG_70	790	172	0	0	7	20	88	0	9
CDFG_71	191	112	0	0	2	6	11	0	13
HF_41	1	15	1	0	14	4	8	9	5
HF_42	9	208	2	0	13	331	224	1	387
HF_43	8	70	0	0	108	32	30	0	24
HF_44	2	37	1	0	22	19	14	0	130
HF_57	0	11	0	0	131	7	12	2	61
HF_58	0	100	13	0	286	241	81	4	157
HF_59	0	8	1	0	77	78	16	0	32
HF_60	2	15	0	0	17	118	46	0	31
HF_72	0	42	9	82	56	50	24	1	0

Kingdom	Bacteria	Bacteria	Bacteria	Bacteria	Bacteria	Bacteria	Bacteria	Bacteria	Bacteria
Phylum	Firmicutes	Firmicutes	Actinobacteria	Firmicutes	Firmicutes	Firmicutes	Firmicutes	Bacteroidetes	Firmicutes
Class	Clostridia	Clostridia	Actinobacteria	Bacilli	Clostridia	Clostridia	Clostridia	Bacteroidia	Clostridia
Order	Clostridiales	Clostridiales	Coriobacteriales	Bacillales	Clostridiales	Clostridiales	Clostridiales	Bacteroidales	Clostridiales
Family	Lachnospiraceae	Lachnospiraceae	Coriobacteriaceae	Staphylococcaceae	Lachnospiraceae	Ruminococcaceae	Lachnospiraceae	Porphyromonadaceae	Clostridiaceae_
Genus	Clostridium_XlVa	Blautia	Asaccharobacter	Staphylococcus	Acetatifactor	Oscillibacter	Clostridium_XlVa	Parabacteroides	Alkaliphilus
#NAME	ZOTU_0043	ZOTU_0044	ZOTU_0045	ZOTU_0046	ZOTU_0047	ZOTU_0048	ZOTU_0049	ZOTU_0050	ZOTU_0051
HF_73	0	26	5	11	4	17	8	0	5
HF_74	0	23	5	5	37	78	26	0	16
HFFG_45	24	33	1	0	6	5	5	2	19
HFFG_46	0	8	0	1	15	29	2	0	6
HFFG_47	1	11	0	0	1	3	2	0	6
HFFG_48	0	16	0	5	16	1	10	0	8
HFFG_61	33	3	2	0	15	5	9	0	12
HFFG_62	0	15	3	0	26	27	0	0	11
HFFG_63	0	12	3	0	15	7	3	0	2
HFFG_64	0	13	2	0	211	57	16	1	6
HFFG_75	0	139	176	0	36	86	8	0	113
HFFG_76	0	153	295	64	118	253	15	0	21
HFFG_77	0	23	72	1	2	8	4	0	21

Kingdom	Bacteria	Bacteria	Bacteria	Bacteria	Bacteria	Bacteria	Bacteria	Bacteria	Bacteria
Phylum	Firmicutes	Firmicutes	Firmicutes	Bacteroidetes	Firmicutes	Firmicutes	Firmicutes	Firmicutes	Firmicutes
Class	Clostridia	Clostridia	Clostridia	Bacteroidia	Clostridia	Clostridia	Clostridia	Clostridia	Clostridia
Order	Clostridiales	Clostridiales	Clostridiales	Bacteroidales	Clostridiales	Clostridiales	Clostridiales	Clostridiales	Clostridiales
Family	Clostridiaceae_	Ruminococcaceae	Ruminococcaceae	Porphyromonadaceae	Lachnospiraceae	Lachnospiraceae	Ruminococcaceae	Lachnospiraceae	Clostridiaceae_
Genus	Alkaliphilus	Flavonifractor	Clostridium_IV	Barnesiella	Clostridium_XlVa	Acetatifactor	Oscillibacter	Ruminococcus	Alkaliphilus
#NAME	ZOTU_0052	ZOTU_0053	ZOTU_0054	ZOTU_0055	ZOTU_0056	ZOTU_0057	ZOTU_0058	ZOTU_0059	ZOTU_0060
CD_49	10	9	25	479	0	6	3	10	0
CD_50	0	13	84	386	23	4	5	0	3
CD_51	0	198	195	760	91	34	18	3	0
CD_52	1	14	36	457	0	1	10	12	0
CD_65	0	4	47	73	497	1	1	0	0
CD_66	0	11	30	127	304	1	2	5	1
CD_67	0	0	11	174	1	15	7	11	0
CD_68	0	21	37	92	540	10	5	13	0
CD_78	0	4	25	0	0	0	1	0	0
CD_79	0	1	9	0	0	0	0	1	0
CD_80	1	0	21	1	3	1	5	0	0
CDFG_53	49	3	55	8	1	2	3	0	0
CDFG_54	3	80	515	147	4	46	91	1	0
CDFG_55	427	22	433	81	0	9	28	7	0
CDFG_56	76	15	60	33	2	1	2	1	0
CDFG_69	33	3	33	0	12	0	3	0	0
CDFG_70	84	1	71	1	31	7	46	1	0
CDFG_71	39	5	67	0	0	1	9	3	0
HF_41	4	64	16	41	3	0	3	0	226
HF_42	0	277	177	128	2	44	7	13	3042
HF_43	0	10	33	87	2	45	7	24	2
HF_44	0	0	25	24	2	9	1	9	1
HF_57	0	7	13	19	2	20	29	28	0
HF_58	0	19	51	174	2	35	131	428	0
HF_59	11	14	15	55	1	5	48	87	0
HF_60	2	37	47	23	0	9	41	32	0
HF_72	8	21	2	121	0	5	13	11	0

Kingdom	Bacteria	Bacteria	Bacteria	Bacteria	Bacteria	Bacteria	Bacteria	Bacteria	Bacteria
Phylum	Firmicutes	Firmicutes	Firmicutes	Bacteroidetes	Firmicutes	Firmicutes	Firmicutes	Firmicutes	Firmicutes
Class	Clostridia	Clostridia	Clostridia	Bacteroidia	Clostridia	Clostridia	Clostridia	Clostridia	Clostridia
Order	Clostridiales	Clostridiales	Clostridiales	Bacteroidales	Clostridiales	Clostridiales	Clostridiales	Clostridiales	Clostridiales
Family	Clostridiaceae_	Ruminococcaceae	Ruminococcaceae	Porphyromonadaceae	Lachnospiraceae	Lachnospiraceae	Ruminococcaceae	Lachnospiraceae	Clostridiaceae_
Genus	Alkaliphilus	Flavonifractor	Clostridium_IV	Barnesiella	Clostridium_XlVa	Acetatifactor	Oscillibacter	Ruminococcus	Alkaliphilus
#NAME	ZOTU_0052	ZOTU_0053	ZOTU_0054	ZOTU_0055	ZOTU_0056	ZOTU_0057	ZOTU_0058	ZOTU_0059	ZOTU_0060
HF_73	2	7	1	18	8	5	6	5	0
HF_74	288	30	29	37	0	27	27	37	1
HFFG_45	0	6	49	15	58	0	1	0	0
HFFG_46	84	17	16	32	0	4	16	1	0
HFFG_47	33	0	17	6	3	0	1	1	0
HFFG_48	34	6	6	9	4	0	7	1	0
HFFG_61	19	2	3	0	0	1	8	10	0
HFFG_62	194	0	20	0	0	2	3	2	0
HFFG_63	37	3	10	0	0	6	11	7	0
HFFG_64	216	14	14	2	0	13	55	23	0
HFFG_75	765	26	167	0	3	5	29	6	0
HFFG_76	977	68	146	0	12	32	46	6	0
HFFG_77	205	2	23	0	2	0	9	1	0

Kingdom	Bacteria	Bacteria	Bacteria	Bacteria	Bacteria	Bacteria	Bacteria	Bacteria	Bacteria
Phylum	Firmicutes	Firmicutes	Bacteroidetes	Firmicutes	Firmicutes	Firmicutes	Actinobacteria	Firmicutes	Firmicutes
Class	Clostridia	Clostridia	Bacteroidia	Clostridia	Clostridia	Clostridia	Actinobacteria	Clostridia	Clostridia
Order	Clostridiales	Clostridiales	Bacteroidales	Clostridiales	Clostridiales	Clostridiales	Coriobacteriales	Clostridiales	Clostridiales
Family	Lachnospiraceae	Lachnospiraceae	Porphyromonadaceae	Lachnospiraceae	Ruminococcaceae	Lachnospiraceae	Coriobacteriaceae	Lachnospiraceae	Ruminococcaceae
Genus	Blautia	Clostridium_XlVa	Barnesiella	Acetatifactor	Clostridium_III	Acetatifactor	Paraeggerthella	Clostridium_XlVa	Ruminococcus
#NAME	ZOTU_0061	ZOTU_0062	ZOTU_0063	ZOTU_0064	ZOTU_0065	ZOTU_0066	ZOTU_0067	ZOTU_0068	ZOTU_0069
CD_49	0	0	445	13	36	8	8	8	5
CD_50	1	0	338	0	76	5	40	11	6
CD_51	0	0	663	8	87	19	84	18	16
CD_52	52	38	372	4	36	6	13	14	0
CD_65	0	0	65	0	13	2	9	0	1
CD_66	1	8	135	4	18	0	1	1	9
CD_67	0	2	135	10	6	1	2	1	1
CD_68	0	21	70	0	29	3	2	0	4
CD_78	0	2	0	0	5	1	10	0	1
CD_79	0	0	0	0	2	0	2	0	0
CD_80	0	1	0	0	13	0	30	0	2
CDFG_53	0	1	6	1	29	0	7	1	42
CDFG_54	11	84	125	41	16	3	22	101	154
CDFG_55	22	29	65	6	168	1	29	23	67
CDFG_56	6	19	27	2	47	1	1	4	9
CDFG_69	0	23	0	1	0	0	8	0	40
CDFG_70	12	18	0	9	0	0	10	2	42
CDFG_71	10	38	0	2	0	0	7	8	20
HF_41	1	0	49	3	47	1	22	0	5
HF_42	37	0	96	6	39	5	7	75	43
HF_43	20	1	65	21	1	34	11	3	27
HF_44	9	0	19	5	36	12	12	0	7
HF_57	4	0	16	0	6	21	1	22	2
HF_58	35	0	104	24	49	314	5	103	21
HF_59	10	0	44	3	4	24	2	69	14
HF_60	3	0	6	3	21	11	3	47	12
HF_72	1	31	103	19	20	1	11	0	21

Kingdom	Bacteria	Bacteria	Bacteria	Bacteria	Bacteria	Bacteria	Bacteria	Bacteria	Bacteria
Phylum	Firmicutes	Firmicutes	Bacteroidetes	Firmicutes	Firmicutes	Firmicutes	Actinobacteria	Firmicutes	Firmicutes
Class	Clostridia	Clostridia	Bacteroidia	Clostridia	Clostridia	Clostridia	Actinobacteria	Clostridia	Clostridia
Order	Clostridiales	Clostridiales	Bacteroidales	Clostridiales	Clostridiales	Clostridiales	Coriobacteriales	Clostridiales	Clostridiales
Family	Lachnospiraceae	Lachnospiraceae	Porphyromonadaceae	Lachnospiraceae	Ruminococcaceae	Lachnospiraceae	Coriobacteriaceae	Lachnospiraceae	Ruminococcaceae
Genus	Blautia	Clostridium_XlVa	Barnesiella	Acetatifactor	Clostridium_III	Acetatifactor	Paraeggerthella	Clostridium_XlVa	Ruminococcus
#NAME	ZOTU_0061	ZOTU_0062	ZOTU_0063	ZOTU_0064	ZOTU_0065	ZOTU_0066	ZOTU_0067	ZOTU_0068	ZOTU_0069
HF_73	0	3	12	2	15	3	4	6	3
HF_74	17	58	51	43	39	15	4	18	16
HFFG_45	1	9	23	2	20	2	10	0	10
HFFG_46	0	16	30	18	9	0	3	0	8
HFFG_47	0	7	1	2	4	0	2	0	3
HFFG_48	2	9	4	7	0	0	4	0	1
HFFG_61	0	33	0	14	1	10	0	0	1
HFFG_62	0	27	0	10	14	2	9	0	0
HFFG_63	0	12	0	3	4	1	1	0	1
HFFG_64	1	18	1	58	8	10	3	0	11
HFFG_75	8	62	2	42	32	6	142	17	5
HFFG_76	18	35	0	81	36	12	162	0	5
HFFG_77	6	12	0	0	3	2	40	1	3

Kingdom	Bacteria	Bacteria	Bacteria	Bacteria	Bacteria	Bacteria	Bacteria	Bacteria	Bacteria
Phylum	Firmicutes	Firmicutes	Firmicutes	Firmicutes	Firmicutes	Firmicutes	Firmicutes	Firmicutes	Firmicutes
Class	Clostridia	Clostridia	Clostridia	Clostridia	Clostridia	Clostridia	Clostridia	Clostridia	Clostridia
Order	Clostridiales	Clostridiales	Clostridiales	Clostridiales	Clostridiales	Clostridiales	Clostridiales	Clostridiales	Clostridiales
Family	Ruminococcaceae	Lachnospiraceae	Lachnospiraceae	Lachnospiraceae	Catabacteriaceae	Lachnospiraceae	Ruminococcaceae	Ruminococcaceae	Lachnospiraceae
Genus	Clostridium_IV	Clostridium_XlVa	Clostridium_XlVa	Acetatifactor	Catabacter	Clostridium_XlVa	Clostridium_III	Sporobacter	Acetatifactor
#NAME	ZOTU_0070	ZOTU_0071	ZOTU_0072	ZOTU_0073	ZOTU_0074	ZOTU_0075	ZOTU_0076	ZOTU_0077	ZOTU_0078
CD_49	1	0	17	26	13	0	47	50	0
CD_50	0	0	3	10	64	0	42	21	0
CD_51	3	0	35	90	84	0	93	260	0
CD_52	9	0	10	14	8	0	15	12	0
CD_65	0	0	0	24	20	0	22	3	0
CD_66	0	0	0	7	8	1	22	12	9
CD_67	2	0	0	37	8	0	14	80	2
CD_68	0	0	0	14	20	1	21	17	34
CD_78	0	2	0	3	11	0	20	11	4
CD_79	0	0	1	0	5	0	2	12	0
CD_80	0	0	0	2	5	3	0	15	3
CDFG_53	1	0	0	0	1	0	1	3	0
CDFG_54	127	13	0	53	57	3	31	6	0
CDFG_55	28	3	0	10	27	0	25	3	0
CDFG_56	25	66	1	4	3	0	6	1	0
CDFG_69	0	12	1	0	8	0	5	3	6
CDFG_70	10	575	3	2	8	0	13	0	0
CDFG_71	0	7	2	1	8	0	1	0	2
HF_41	0	0	0	11	6	0	2	13	0
HF_42	2	0	0	6	56	1	62	363	6
HF_43	4	0	0	44	30	0	52	1	2
HF_44	0	1	0	5	8	0	20	0	2
HF_57	4	0	37	55	9	0	13	0	21
HF_58	33	0	125	140	63	0	48	1	195
HF_59	26	1	41	25	17	0	8	0	18
HF_60	8	0	69	11	35	0	31	1	1
HF_72	0	32	0	32	30	0	21	4	3

Kingdom	Bacteria	Bacteria	Bacteria	Bacteria	Bacteria	Bacteria	Bacteria	Bacteria	Bacteria
Phylum	Firmicutes	Firmicutes	Firmicutes	Firmicutes	Firmicutes	Firmicutes	Firmicutes	Firmicutes	Firmicutes
Class	Clostridia	Clostridia	Clostridia	Clostridia	Clostridia	Clostridia	Clostridia	Clostridia	Clostridia
Order	Clostridiales	Clostridiales	Clostridiales	Clostridiales	Clostridiales	Clostridiales	Clostridiales	Clostridiales	Clostridiales
Family	Ruminococcaceae	Lachnospiraceae	Lachnospiraceae	Lachnospiraceae	Catabacteriaceae	Lachnospiraceae	Ruminococcaceae	Ruminococcaceae	Lachnospiraceae
Genus	Clostridium_IV	Clostridium_XlVa	Clostridium_XlVa	Acetatifactor	Catabacter	Clostridium_XlVa	Clostridium_III	Sporobacter	Acetatifactor
#NAME	ZOTU_0070	ZOTU_0071	ZOTU_0072	ZOTU_0073	ZOTU_0074	ZOTU_0075	ZOTU_0076	ZOTU_0077	ZOTU_0078
HF_73	1	0	0	2	2	0	3	0	4
HF_74	4	18	0	17	15	0	12	2	29
HFFG_45	0	54	0	2	3	0	9	27	0
HFFG_46	2	19	0	9	7	0	5	12	0
HFFG_47	0	27	0	0	0	0	0	1	0
HFFG_48	2	99	0	4	2	0	14	6	0
HFFG_61	4	0	0	9	5	0	2	2	0
HFFG_62	2	0	4	10	7	0	7	0	0
HFFG_63	3	0	2	9	2	0	2	0	1
HFFG_64	72	1	0	91	39	1	8	11	6
HFFG_75	5	0	175	8	26	0	51	3	10
HFFG_76	2	0	125	54	34	1	99	19	7
HFFG_77	3	0	2	4	5	1	9	1	1

Kingdom	Bacteria	Bacteria	Bacteria	Bacteria	Bacteria	Bacteria	Bacteria	Bacteria	Bacteria
Phylum	Firmicutes	Bacteroidetes	Firmicutes	Firmicutes	Firmicutes	Firmicutes	Firmicutes	Firmicutes	Firmicutes
Class	Clostridia	Bacteroidia	Clostridia	Clostridia	Clostridia	Clostridia	Clostridia	Clostridia	Clostridia
Order	Clostridiales	Bacteroidales	Clostridiales	Clostridiales	Clostridiales	Clostridiales	Clostridiales	Clostridiales	Clostridiales
Family	Lachnospiraceae	Porphyromonadaceae	Ruminococcaceae	Ruminococcaceae	Ruminococcaceae	Clostridiaceae_	Ruminococcaceae	Lachnospiraceae	Lachnospiraceae
Genus	Murimonas	Coprobacter	Oscillibacter	Clostridium_IV	Pseudoflavonifractor	Alkaliphilus	Clostridium_IV	Acetatifactor	Acetatifactor
#NAME	ZOTU_0079	ZOTU_0080	ZOTU_0081	ZOTU_0082	ZOTU_0083	ZOTU_0084	ZOTU_0085	ZOTU_0086	ZOTU_0087
CD_49	2	1	9	33	1	0	0	9	0
CD_50	7	0	0	32	17	0	16	2	0
CD_51	21	0	87	112	83	3	30	20	0
CD_52	3	0	1	13	3	0	0	2	0
CD_65	0	68	0	7	47	0	3	0	0
CD_66	0	167	2	8	14	0	0	3	0
CD_67	2	298	1	7	12	0	3	0	0
CD_68	2	43	6	11	30	0	4	2	2
CD_78	1	0	1	0	6	0	0	0	0
CD_79	0	0	0	1	0	0	0	0	0
CD_80	2	0	0	1	4	9	10	1	0
CDFG_53	0	0	1	6	0	0	0	0	0
CDFG_54	47	0	29	36	38	0	39	2	7
CDFG_55	21	0	2	4	16	1	4	0	1
CDFG_56	5	1	0	6	2	2	2	1	1
CDFG_69	0	0	2	4	5	0	0	0	1
CDFG_70	16	0	0	1	6	1	2	0	3
CDFG_71	2	0	2	0	0	458	1	0	0
HF_41	1	1	4	71	4	2	2	0	0
HF_42	2	2	52	235	81	30	169	1	35
HF_43	8	0	39	19	12	0	11	3	0
HF_44	0	1	10	12	8	2	9	1	0
HF_57	7	1	0	87	1	0	14	19	0
HF_58	76	2	144	150	37	0	79	202	0
HF_59	26	0	55	62	6	0	27	19	0
HF_60	3	0	59	16	8	0	25	20	0
HF_72	1	25	28	110	133	21	37	1	0

Kingdom	Bacteria	Bacteria	Bacteria	Bacteria	Bacteria	Bacteria	Bacteria	Bacteria	Bacteria
Phylum	Firmicutes	Bacteroidetes	Firmicutes	Firmicutes	Firmicutes	Firmicutes	Firmicutes	Firmicutes	Firmicutes
Class	Clostridia	Bacteroidia	Clostridia	Clostridia	Clostridia	Clostridia	Clostridia	Clostridia	Clostridia
Order	Clostridiales	Bacteroidales	Clostridiales	Clostridiales	Clostridiales	Clostridiales	Clostridiales	Clostridiales	Clostridiales
Family	Lachnospiraceae	Porphyromonadaceae	Ruminococcaceae	Ruminococcaceae	Ruminococcaceae	Clostridiaceae_	Ruminococcaceae	Lachnospiraceae	Lachnospiraceae
Genus	Murimonas	Coprobacter	Oscillibacter	Clostridium_IV	Pseudoflavonifractor	Alkaliphilus	Clostridium_IV	Acetatifactor	Acetatifactor
#NAME	ZOTU_0079	ZOTU_0080	ZOTU_0081	ZOTU_0082	ZOTU_0083	ZOTU_0084	ZOTU_0085	ZOTU_0086	ZOTU_0087
HF_73	1	3	8	18	14	10	6	4	0
HF_74	6	24	8	60	134	57	32	1	0
HFFG_45	5	5	3	1	1	48	1	0	0
HFFG_46	4	31	10	6	3	0	3	0	0
HFFG_47	1	3	0	2	0	29	0	2	0
HFFG_48	1	7	1	3	1	13	8	0	0
HFFG_61	6	3	2	8	22	0	4	9	0
HFFG_62	0	14	0	14	2	1	4	3	0
HFFG_63	6	3	3	9	3	0	0	2	0
HFFG_64	19	24	29	58	4	1	45	14	1
HFFG_75	6	0	46	39	47	481	35	6	0
HFFG_76	48	0	96	17	42	440	71	12	1
HFFG_77	10	0	6	9	5	5	17	0	0

Kingdom	Bacteria	Bacteria	Bacteria	Bacteria	Bacteria	Bacteria	Bacteria	Bacteria	Bacteria
Phylum	Firmicutes	Firmicutes	Firmicutes	Bacteroidetes	Firmicutes	Firmicutes	Firmicutes	Firmicutes	Firmicutes
Class	Clostridia	Clostridia	Clostridia	Bacteroidia	Clostridia	Clostridia	Clostridia	Clostridia	Clostridia
Order	Clostridiales	Clostridiales	Clostridiales	Bacteroidales	Clostridiales	Clostridiales	Clostridiales	Clostridiales	Clostridiales
Family	Lachnospiraceae	Lachnospiraceae	Eubacteriaceae	Porphyromonadaceae	Ruminococcaceae	Lachnospiraceae	Ruminococcaceae	Lachnospiraceae	Lachnospiraceae
Genus	Blautia	Clostridium_XlVa	Eubacterium	Barnesiella	Saccharofermentans	Clostridium_XlVa	Clostridium_IV	Anaerostipes	Clostridium_XlVb
#NAME	ZOTU_0088	ZOTU_0089	ZOTU_0090	ZOTU_0091	ZOTU_0092	ZOTU_0093	ZOTU_0094	ZOTU_0095	ZOTU_0096
CD_49	7	16	0	188	6	0	18	0	8
CD_50	18	44	0	156	17	2	156	0	27
CD_51	22	54	0	198	34	5	2	0	33
CD_52	27	13	0	96	12	2	31	0	6
CD_65	1	35	0	4	7	1	21	0	1
CD_66	0	4	0	39	15	0	1	0	1
CD_67	1	8	0	13	6	0	15	0	0
CD_68	0	11	0	20	14	0	10	0	0
CD_78	3	14	0	4	1	0	24	0	4
CD_79	0	0	0	3	0	0	0	0	5
CD_80	2	12	1	1	5	0	12	0	7
CDFG_53	35	0	0	1	0	0	0	2	0
CDFG_54	120	11	0	17	35	5	51	566	24
CDFG_55	131	4	0	4	23	0	38	54	5
CDFG_56	12	3	0	4	3	0	16	30	0
CDFG_69	72	0	0	1	0	0	8	1	2
CDFG_70	52	4	0	6	4	8	26	4	2
CDFG_71	34	1	0	1	1	1	15	11	0
HF_41	5	1	3	4	3	0	31	0	2
HF_42	51	4	1230	3	345	5	45	0	138
HF_43	28	78	7	10	14	2	84	0	6
HF_44	14	13	102	0	9	2	22	0	6
HF_57	8	25	0	5	0	5	3	0	7
HF_58	18	27	0	30	2	66	1	0	76
HF_59	8	29	0	35	4	19	0	0	30
HF_60	4	17	0	4	31	4	0	0	93
HF_72	10	23	1	2	3	0	48	1	1

Kingdom	Bacteria	Bacteria	Bacteria	Bacteria	Bacteria	Bacteria	Bacteria	Bacteria	Bacteria
Phylum	Firmicutes	Firmicutes	Firmicutes	Bacteroidetes	Firmicutes	Firmicutes	Firmicutes	Firmicutes	Firmicutes
Class	Clostridia	Clostridia	Clostridia	Bacteroidia	Clostridia	Clostridia	Clostridia	Clostridia	Clostridia
Order	Clostridiales	Clostridiales	Clostridiales	Bacteroidales	Clostridiales	Clostridiales	Clostridiales	Clostridiales	Clostridiales
Family	Lachnospiraceae	Lachnospiraceae	Eubacteriaceae	Porphyromonadaceae	Ruminococcaceae	Lachnospiraceae	Ruminococcaceae	Lachnospiraceae	Lachnospiraceae
Genus	Blautia	Clostridium_XlVa	Eubacterium	Barnesiella	Saccharofermentans	Clostridium_XlVa	Clostridium_IV	Anaerostipes	Clostridium_XlVb
#NAME	ZOTU_0088	ZOTU_0089	ZOTU_0090	ZOTU_0091	ZOTU_0092	ZOTU_0093	ZOTU_0094	ZOTU_0095	ZOTU_0096
HF_73	3	8	0	1	0	0	8	0	1
HF_74	8	30	0	5	6	4	22	0	2
HFFG_45	13	4	0	1	11	1	2	2	1
HFFG_46	6	6	0	8	6	0	6	14	0
HFFG_47	2	1	0	0	3	0	4	1	0
HFFG_48	9	2	0	6	3	0	8	6	0
HFFG_61	2	3	0	1	3	0	6	11	1
HFFG_62	4	2	0	0	4	0	1	17	0
HFFG_63	0	2	0	0	4	0	8	20	0
HFFG_64	2	11	0	5	12	0	8	79	9
HFFG_75	31	5	0	57	23	0	21	0	8
HFFG_76	40	9	0	89	30	1	69	0	5
HFFG_77	9	12	0	0	9	1	12	0	1

Kingdom	Bacteria	Bacteria	Bacteria	Bacteria	Bacteria	Bacteria	Bacteria	Bacteria	Bacteria
Phylum	Firmicutes	Firmicutes	Firmicutes	Firmicutes	Firmicutes	Firmicutes	Firmicutes	Firmicutes	Firmicutes
Class	Clostridia	Clostridia	Clostridia	Clostridia	Clostridia	Clostridia	Clostridia	Clostridia	Clostridia
Order	Clostridiales	Clostridiales	Clostridiales	Clostridiales	Clostridiales	Clostridiales	Clostridiales	Clostridiales	Clostridiales
Family	Clostridiaceae_	Ruminococcaceae	Lachnospiraceae	Ruminococcaceae	Lachnospiraceae	Lachnospiraceae	Lachnospiraceae	Lachnospiraceae	Lachnospiraceae
Genus	Alkaliphilus	Clostridium_IV	Clostridium_XlVa	Pseudoflavonifractor	Acetatifactor	Clostridium_XlVa	Roseburia	Marvinbryantia	Clostridium_XlVa
#NAME	ZOTU_0097	ZOTU_0098	ZOTU_0099	ZOTU_0100	ZOTU_0101	ZOTU_0102	ZOTU_0103	ZOTU_0104	ZOTU_0105
CD_49	0	0	1	8	2	4	1	0	0
CD_50	0	0	2	11	0	1	36	7	0
CD_51	0	1	2	63	1	21	17	0	0
CD_52	0	0	0	2	3	6	2	5	0
CD_65	0	0	93	5	0	5	8	0	0
CD_66	0	0	50	4	1	1	0	1	0
CD_67	0	0	1	0	0	2	3	0	0
CD_68	0	0	20	9	0	2	5	0	0
CD_78	0	0	58	0	0	0	2	0	0
CD_79	0	0	1	0	0	0	0	0	0
CD_80	0	2	3	2	0	1	2	0	0
CDFG_53	0	15	3	0	0	0	0	0	0
CDFG_54	0	658	2	50	0	23	8	16	0
CDFG_55	0	62	148	6	0	15	10	8	0
CDFG_56	0	69	23	3	1	4	1	2	0
CDFG_69	0	58	1	2	0	0	0	2	0
CDFG_70	0	0	2	4	0	1	9	0	0
CDFG_71	1	132	0	3	0	1	3	6	0
HF_41	2	0	0	0	2	0	0	2	0
HF_42	1	0	104	13	2	13	28	40	0
HF_43	0	0	3	0	5	6	10	9	0
HF_44	1	0	0	0	2	1	9	24	0
HF_57	0	0	1	1	9	14	11	0	0
HF_58	0	0	3	12	129	72	36	0	0
HF_59	0	0	0	8	15	18	13	0	0
HF_60	0	0	0	3	10	27	2	0	0
HF_72	0	1	1	2	0	3	5	0	0

Kingdom	Bacteria	Bacteria	Bacteria	Bacteria	Bacteria	Bacteria	Bacteria	Bacteria	Bacteria
Phylum	Firmicutes	Firmicutes	Firmicutes	Firmicutes	Firmicutes	Firmicutes	Firmicutes	Firmicutes	Firmicutes
Class	Clostridia	Clostridia	Clostridia	Clostridia	Clostridia	Clostridia	Clostridia	Clostridia	Clostridia
Order	Clostridiales	Clostridiales	Clostridiales	Clostridiales	Clostridiales	Clostridiales	Clostridiales	Clostridiales	Clostridiales
Family	Clostridiaceae_	Ruminococcaceae	Lachnospiraceae	Ruminococcaceae	Lachnospiraceae	Lachnospiraceae	Lachnospiraceae	Lachnospiraceae	Lachnospiraceae
Genus	Alkaliphilus	Clostridium_IV	Clostridium_XlVa	Pseudoflavonifractor	Acetatifactor	Clostridium_XlVa	Roseburia	Marvinbryantia	Clostridium_XlVa
#NAME	ZOTU_0097	ZOTU_0098	ZOTU_0099	ZOTU_0100	ZOTU_0101	ZOTU_0102	ZOTU_0103	ZOTU_0104	ZOTU_0105
HF_73	0	0	1	4	1	1	0	0	0
HF_74	0	0	8	11	0	1	3	0	0
HFFG_45	0	0	0	2	1	2	3	0	0
HFFG_46	0	0	0	4	0	4	2	0	0
HFFG_47	0	0	0	0	1	1	1	0	0
HFFG_48	0	0	0	3	0	2	1	0	0
HFFG_61	0	0	2	3	4	4	6	0	0
HFFG_62	0	0	2	5	0	1	3	0	0
HFFG_63	0	0	1	0	0	3	3	0	0
HFFG_64	1	5	4	20	4	7	5	0	0
HFFG_75	0	0	4	46	1	4	16	1	0
HFFG_76	0	0	6	29	1	19	28	0	0
HFFG_77	0	4	0	2	0	0	23	0	0

Kingdom	Bacteria	Bacteria	Bacteria	Bacteria	Bacteria	Bacteria	Bacteria	Bacteria	Bacteria
Phylum	Actinobacteria	Firmicutes	Firmicutes	Firmicutes	Firmicutes	Firmicutes	Firmicutes	Firmicutes	Firmicutes
Class	Actinobacteria	Clostridia	Clostridia	Clostridia	Clostridia	Clostridia	Clostridia	Clostridia	Clostridia
Order	Actinomycetales	Clostridiales	Clostridiales	Clostridiales	Clostridiales	Clostridiales	Clostridiales	Clostridiales	Clostridiales
Family	Mycobacteriaceae	Ruminococcaceae	Ruminococcaceae	Clostridiaceae_	Lachnospiraceae	Ruminococcaceae	Lachnospiraceae	Lachnospiraceae	Lachnospiraceae
Genus	Mycobacterium	Clostridium_IV	Flavonifractor	Alkaliphilus	Acetatifactor	Oscillibacter	Blautia	Clostridium_XlVa	Clostridium_XlVa
#NAME	ZOTU_0106	ZOTU_0107	ZOTU_0108	ZOTU_0109	ZOTU_0110	ZOTU_0111	ZOTU_0112	ZOTU_0113	ZOTU_0114
CD_49	0	0	0	0	0	2	0	0	6
CD_50	0	1	1	0	0	9	0	6	0
CD_51	0	18	0	0	0	16	0	14	22
CD_52	0	6	12	0	0	5	15	1	5
CD_65	0	2	0	0	0	15	0	3	0
CD_66	0	1	1	0	0	10	0	1	0
CD_67	0	12	1	0	0	0	0	1	17
CD_68	0	23	0	0	0	14	0	0	1
CD_78	0	1	0	0	0	2	0	0	0
CD_79	0	0	0	0	0	1	0	0	0
CD_80	1	0	0	0	0	2	0	0	0
CDFG_53	0	0	1	0	0	0	0	0	2
CDFG_54	0	5	51	0	0	23	4	25	5
CDFG_55	0	4	2	0	0	6	7	39	0
CDFG_56	0	0	2	0	0	0	2	1	1
CDFG_69	0	2	0	0	0	0	1	1	0
CDFG_70	0	1	2	0	1	1	16	17	0
CDFG_71	0	0	2	0	1	1	0	5	0
HF_41	0	1	27	5	0	8	0	0	1
HF_42	1	50	326	1	1	525	7	5	8
HF_43	0	3	0	0	0	57	0	3	3
HF_44	0	4	5	0	1	29	1	0	1
HF_57	0	16	0	3	0	4	0	1	0
HF_58	0	107	0	217	0	8	8	25	0
HF_59	0	39	1	0	0	2	2	8	0
HF_60	0	29	0	0	0	5	0	5	0
HF_72	0	1	0	0	0	9	3	0	1

Kingdom	Bacteria	Bacteria	Bacteria	Bacteria	Bacteria	Bacteria	Bacteria	Bacteria	Bacteria
Phylum	Actinobacteria	Firmicutes	Firmicutes	Firmicutes	Firmicutes	Firmicutes	Firmicutes	Firmicutes	Firmicutes
Class	Actinobacteria	Clostridia	Clostridia	Clostridia	Clostridia	Clostridia	Clostridia	Clostridia	Clostridia
Order	Actinomycetales	Clostridiales	Clostridiales	Clostridiales	Clostridiales	Clostridiales	Clostridiales	Clostridiales	Clostridiales
Family	Mycobacteriaceae	Ruminococcaceae	Ruminococcaceae	Clostridiaceae_	Lachnospiraceae	Ruminococcaceae	Lachnospiraceae	Lachnospiraceae	Lachnospiraceae
Genus	Mycobacterium	Clostridium_IV	Flavonifractor	Alkaliphilus	Acetatifactor	Oscillibacter	Blautia	Clostridium_XlVa	Clostridium_XlVa
#NAME	ZOTU_0106	ZOTU_0107	ZOTU_0108	ZOTU_0109	ZOTU_0110	ZOTU_0111	ZOTU_0112	ZOTU_0113	ZOTU_0114
HF_73	0	3	0	0	0	5	0	1	0
HF_74	0	11	3	0	0	21	2	3	0
HFFG_45	2	6	4	112	0	1	0	1	0
HFFG_46	1	7	2	34	0	0	0	3	2
HFFG_47	0	2	0	91	1	0	0	0	0
HFFG_48	0	4	0	0	0	1	0	2	0
HFFG_61	0	1	4	0	0	3	0	2	3
HFFG_62	0	0	0	0	0	1	0	0	1
HFFG_63	0	3	3	0	1	0	0	2	4
HFFG_64	0	50	4	1	0	1	0	6	48
HFFG_75	0	44	2	224	1	0	0	7	15
HFFG_76	0	52	3	0	1	2	4	16	15
HFFG_77	0	7	0	0	0	2	2	34	3

Kingdom	Bacteria	Bacteria	Bacteria	Bacteria	Bacteria	Bacteria	Bacteria	Bacteria
Phylum	Firmicutes	Firmicutes	Firmicutes	Firmicutes	Firmicutes	Firmicutes	Firmicutes	Firmicutes
Class	Clostridia	Clostridia	Bacilli	Clostridia	Clostridia	Clostridia	Clostridia	Clostridia
Order	Clostridiales	Clostridiales	Bacillales	Clostridiales	Clostridiales	Clostridiales	Clostridiales	Clostridiales
Family	Lachnospiraceae	Lachnospiraceae	Bacillaceae_	Lachnospiraceae	Clostridiales_Incertae_Sedis_XII	Ruminococcaceae	Lachnospiraceae	Lachnospiraceae
Genus	Clostridium_XlVa	Clostridium_XlVa	Bacillus	Clostridium_XlVa	Guggenheimella	Clostridium_IV	Clostridium_XlVa	Lachnospiracea_incertae_sedis
#NAME	ZOTU_0115	ZOTU_0116	ZOTU_0117	ZOTU_0118	ZOTU_0119	ZOTU_0120	ZOTU_0121	ZOTU_0122
CD_49	5	0	2	0	0	0	0	14
CD_50	15	0	23	0	0	1	0	5
CD_51	1	0	0	0	0	0	2	39
CD_52	9	0	1	0	0	0	0	5
CD_65	10	0	0	0	0	0	1	0
CD_66	0	0	0	0	0	0	3	0
CD_67	3	0	1	0	0	0	6	0
CD_68	0	0	0	0	0	0	2	0
CD_78	2	0	0	0	0	0	1	0
CD_79	0	0	0	0	0	0	0	0
CD_80	1	0	0	0	0	0	2	0
CDFG_53	0	0	0	0	0	0	0	0
CDFG_54	5	7	0	0	0	2	2	51
CDFG_55	0	8	0	0	0	0	4	42
CDFG_56	13	26	0	0	0	0	0	11
CDFG_69	0	3	0	0	0	1	1	0
CDFG_70	1	247	0	0	0	0	0	0
CDFG_71	1	0	0	0	0	0	0	0
HF_41	0	0	0	0	1	0	0	0
HF_42	27	1	0	0	59	0	5	0
HF_43	7	0	0	0	0	1	1	68
HF_44	23	0	0	0	0	0	0	1
HF_57	17	0	0	0	0	0	0	8
HF_58	51	0	0	0	0	0	3	115
HF_59	7	0	0	0	0	0	2	57
HF_60	0	0	0	0	0	0	1	5
HF_72	2	16	0	0	0	1	3	3

Kingdom	Bacteria	Bacteria	Bacteria	Bacteria	Bacteria	Bacteria	Bacteria	Bacteria
Phylum	Firmicutes	Firmicutes	Firmicutes	Firmicutes	Firmicutes	Firmicutes	Firmicutes	Firmicutes
Class	Clostridia	Clostridia	Bacilli	Clostridia	Clostridia	Clostridia	Clostridia	Clostridia
Order	Clostridiales	Clostridiales	Bacillales	Clostridiales	Clostridiales	Clostridiales	Clostridiales	Clostridiales
Family	Lachnospiraceae	Lachnospiraceae	Bacillaceae_	Lachnospiraceae	Clostridiales_Incertae_Sedis_XII	Ruminococcaceae	Lachnospiraceae	Lachnospiraceae
Genus	Clostridium_XlVa	Clostridium_XlVa	Bacillus	Clostridium_XlVa	Guggenheimella	Clostridium_IV	Clostridium_XlVa	Lachnospiracea_incertae_sedis
#NAME	ZOTU_0115	ZOTU_0116	ZOTU_0117	ZOTU_0118	ZOTU_0119	ZOTU_0120	ZOTU_0121	ZOTU_0122
HF_73	1	0	0	0	0	0	0	2
HF_74	16	5	0	0	0	1	0	111
HFFG_45	0	26	0	0	0	0	1	0
HFFG_46	2	5	0	0	0	0	0	0
HFFG_47	0	13	0	0	0	0	0	0
HFFG_48	2	35	0	0	0	0	0	0
HFFG_61	3	0	0	0	0	0	1	0
HFFG_62	0	0	0	0	0	0	2	0
HFFG_63	0	0	0	0	0	0	2	0
HFFG_64	5	1	0	0	0	0	21	0
HFFG_75	1	0	0	0	0	0	5	0
HFFG_76	3	0	0	0	0	0	4	8
HFFG_77	3	0	0	0	0	0	0	0

Kingdom	Bacteria	Bacteria	Bacteria	Bacteria	Bacteria	Bacteria	Bacteria	Bacteria	Bacteria
Phylum	Firmicutes	Firmicutes	Firmicutes	Firmicutes	Firmicutes	Firmicutes	Firmicutes	Firmicutes	Firmicutes
Class	Clostridia	Clostridia	Bacilli	Clostridia	Clostridia	Clostridia	Clostridia	Clostridia	Clostridia
Order	Clostridiales	Clostridiales	Bacillales	Clostridiales	Clostridiales	Clostridiales	Clostridiales	Clostridiales	Clostridiales
Family	Lachnospiraceae	Lachnospiraceae	Paenibacillaceae_	Clostridiaceae_	Lachnospiraceae	Lachnospiraceae	Lachnospiraceae	Lachnospiraceae	Eubacteriaceae
Genus	Clostridium_XlVa	Clostridium_XlVa	Brevibacillus	Clostridium_sensu_stricto	Clostridium_XlVa	Clostridium_XlVa	Acetatifactor	Clostridium_XlVa	Eubacterium
#NAME	ZOTU_0123	ZOTU_0124	ZOTU_0125	ZOTU_0126	ZOTU_0127	ZOTU_0128	ZOTU_0129	ZOTU_0130	ZOTU_0131
CD_49	0	4	0	3	1	0	3	0	21
CD_50	3	1	0	54	2	0	0	0	28
CD_51	6	27	0	12	2	0	1	0	8
CD_52	0	2	0	0	1	0	0	3	42
CD_65	2	2	0	38	0	0	0	2	22
CD_66	1	1	0	0	0	0	0	0	7
CD_67	1	0	0	2	3	0	4	1	15
CD_68	4	1	0	0	0	0	1	2	4
CD_78	0	2	0	2	0	0	0	0	7
CD_79	0	0	0	1	0	0	0	0	1
CD_80	4	0	0	1	0	0	0	0	1
CDFG_53	0	0	0	0	0	0	0	1	0
CDFG_54	71	10	0	29	0	0	9	2	11
CDFG_55	186	63	0	10	2	0	0	3	3
CDFG_56	46	4	0	1	0	0	0	3	0
CDFG_69	0	1	0	4	0	0	0	0	0
CDFG_70	19	3	0	0	0	0	0	1	4
CDFG_71	6	2	0	4	0	0	1	2	3
HF_41	0	0	0	1	1	0	0	1	4
HF_42	3	21	0	57	0	0	0	39	40
HF_43	3	1	0	0	0	0	7	11	6
HF_44	1	1	0	10	0	0	1	1	1
HF_57	7	0	0	6	6	0	1	2	6
HF_58	17	9	0	5	24	1	7	4	13
HF_59	30	1	0	7	30	0	1	8	15
HF_60	9	1	0	15	3	0	0	0	16
HF_72	13	0	0	0	0	0	4	2	11

Kingdom	Bacteria	Bacteria	Bacteria	Bacteria	Bacteria	Bacteria	Bacteria	Bacteria	Bacteria
Phylum	Firmicutes	Firmicutes	Firmicutes	Firmicutes	Firmicutes	Firmicutes	Firmicutes	Firmicutes	Firmicutes
Class	Clostridia	Clostridia	Bacilli	Clostridia	Clostridia	Clostridia	Clostridia	Clostridia	Clostridia
Order	Clostridiales	Clostridiales	Bacillales	Clostridiales	Clostridiales	Clostridiales	Clostridiales	Clostridiales	Clostridiales
Family	Lachnospiraceae	Lachnospiraceae	Paenibacillaceae_	Clostridiaceae_	Lachnospiraceae	Lachnospiraceae	Lachnospiraceae	Lachnospiraceae	Eubacteriaceae
Genus	Clostridium_XlVa	Clostridium_XlVa	Brevibacillus	Clostridium_sensu_stricto	Clostridium_XlVa	Clostridium_XlVa	Acetatifactor	Clostridium_XlVa	Eubacterium
#NAME	ZOTU_0123	ZOTU_0124	ZOTU_0125	ZOTU_0126	ZOTU_0127	ZOTU_0128	ZOTU_0129	ZOTU_0130	ZOTU_0131
HF_73	7	0	0	3	1	0	1	4	2
HF_74	3	0	0	0	0	0	4	4	2
HFFG_45	3	0	0	1	3	0	0	0	1
HFFG_46	9	0	0	1	9	0	5	1	4
HFFG_47	4	0	0	1	0	0	0	1	0
HFFG_48	5	0	0	1	2	0	0	0	1
HFFG_61	0	0	0	1	1	0	4	5	2
HFFG_62	0	0	0	0	0	0	4	0	3
HFFG_63	0	0	0	0	0	0	2	0	1
HFFG_64	1	0	0	8	12	0	14	7	0
HFFG_75	2	0	0	7	0	0	8	2	4
HFFG_76	13	0	0	0	0	0	18	18	22
HFFG_77	2	0	0	0	1	0	0	4	8

Kingdom	Bacteria	Bacteria	Bacteria	Bacteria	Bacteria	Bacteria	Bacteria	Bacteria
Phylum	Firmicutes	Firmicutes	Firmicutes	Firmicutes	Firmicutes	Firmicutes	Firmicutes	Firmicutes
Class	Clostridia	Clostridia	Erysipelotrichia	Clostridia	Clostridia	Erysipelotrichia	Clostridia	Clostridia
Order	Clostridiales	Clostridiales	Erysipelotrichales	Clostridiales	Clostridiales	Erysipelotrichales	Clostridiales	Clostridiales
Family	Ruminococcaceae	Lachnospiraceae	Erysipelotrichaceae	Lachnospiraceae	Lachnospiraceae	Erysipelotrichaceae	Lachnospiraceae	Ruminococcaceae
Genus	Clostridium_IV	Acetatifactor	Coprobacillus	Clostridium_XlVa	Acetatifactor	Erysipelotrichaceae_incertae_sedis	Dorea	Clostridium_IV
#NAME	ZOTU_0132	ZOTU_0133	ZOTU_0134	ZOTU_0135	ZOTU_0136	ZOTU_0137	ZOTU_0138	ZOTU_0139
CD_49	8	0	1	3	0	0	0	2
CD_50	2	0	0	0	0	1	0	0
CD_51	23	0	14	6	0	21	0	2
CD_52	6	1	1	0	0	6	0	3
CD_65	4	0	0	1	0	0	0	2
CD_66	7	0	0	0	1	0	0	1
CD_67	10	0	1	1	2	0	0	2
CD_68	7	0	0	1	10	1	0	0
CD_78	13	0	41	1	0	0	0	0
CD_79	8	0	4	0	0	0	0	0
CD_80	0	0	55	2	0	1	0	1
CDFG_53	0	0	0	0	0	2	0	0
CDFG_54	4	2	0	12	0	6	34	54
CDFG_55	2	1	9	5	0	25	6	2
CDFG_56	0	0	0	1	0	7	2	1
CDFG_69	0	0	0	0	1	7	0	0
CDFG_70	0	1	2	1	0	3	1	5
CDFG_71	2	0	0	0	0	3	0	0
HF_41	0	0	0	0	2	12	0	0
HF_42	0	19	1	11	5	226	0	2
HF_43	9	0	0	4	0	4	0	7
HF_44	1	0	0	1	0	10	0	1
HF_57	1	0	1	6	8	2	0	13
HF_58	18	0	5	37	40	5	1	20
HF_59	7	0	8	13	7	2	0	18
HF_60	3	0	9	10	0	5	0	13
HF_72	0	0	2	3	0	16	0	31

Kingdom	Bacteria	Bacteria	Bacteria	Bacteria	Bacteria	Bacteria	Bacteria	Bacteria
Phylum	Firmicutes	Firmicutes	Firmicutes	Firmicutes	Firmicutes	Firmicutes	Firmicutes	Firmicutes
Class	Clostridia	Clostridia	Erysipelotrichia	Clostridia	Clostridia	Erysipelotrichia	Clostridia	Clostridia
Order	Clostridiales	Clostridiales	Erysipelotrichales	Clostridiales	Clostridiales	Erysipelotrichales	Clostridiales	Clostridiales
Family	Ruminococcaceae	Lachnospiraceae	Erysipelotrichaceae	Lachnospiraceae	Lachnospiraceae	Erysipelotrichaceae	Lachnospiraceae	Ruminococcaceae
Genus	Clostridium_IV	Acetatifactor	Coprobacillus	Clostridium_XlVa	Acetatifactor	Erysipelotrichaceae_incertae_sedis	Dorea	Clostridium_IV
#NAME	ZOTU_0132	ZOTU_0133	ZOTU_0134	ZOTU_0135	ZOTU_0136	ZOTU_0137	ZOTU_0138	ZOTU_0139
HF_73	0	0	0	0	1	1	0	0
HF_74	1	1	0	0	5	5	0	5
HFFG_45	0	0	5	1	0	3	0	0
HFFG_46	4	0	13	1	0	3	2	4
HFFG_47	0	0	0	1	0	1	0	0
HFFG_48	1	0	2	1	0	2	1	2
HFFG_61	2	0	0	0	0	0	0	3
HFFG_62	5	0	0	1	0	3	2	1
HFFG_63	3	0	1	3	0	2	4	2
HFFG_64	18	0	2	5	2	1	14	59
HFFG_75	8	0	27	1	3	35	2	9
HFFG_76	33	0	40	12	1	8	15	5
HFFG_77	0	0	0	1	0	7	2	1

Kingdom	Bacteria	Bacteria	Bacteria	Bacteria	Bacteria	Bacteria	Bacteria	Bacteria
Phylum	Firmicutes	Firmicutes	Firmicutes	Firmicutes	Firmicutes	Firmicutes	Firmicutes	Firmicutes
Class	Erysipelotrichia	Clostridia	Clostridia	Clostridia	Clostridia	Clostridia	Clostridia	Clostridia
Order	Erysipelotrichales	Clostridiales	Clostridiales	Clostridiales	Clostridiales	Clostridiales	Clostridiales	Clostridiales
Family	Erysipelotrichaceae	Lachnospiraceae	Clostridiaceae_	Lachnospiraceae	Ruminococcaceae	Clostridiaceae_	Ruminococcaceae	Ruminococcaceae
Genus	Erysipelotrichaceae_incertae_sedis	Eisenbergiella	Alkaliphilus	Clostridium_XlVa	Intestinimonas	Alkaliphilus	Ruminococcus	Clostridium_IV
#NAME	ZOTU_0140	ZOTU_0141	ZOTU_0142	ZOTU_0143	ZOTU_0144	ZOTU_0145	ZOTU_0146	ZOTU_0147
CD_49	1	0	0	2	5	0	0	0
CD_50	0	3	0	1	1	0	0	0
CD_51	0	7	0	32	28	0	1	12
CD_52	20	0	0	4	2	0	0	0
CD_65	0	2	0	6	0	0	1	3
CD_66	0	0	0	0	4	0	0	0
CD_67	2	1	0	3	6	0	0	0
CD_68	0	5	0	2	7	0	0	0
CD_78	0	0	0	0	0	0	0	0
CD_79	0	0	0	0	0	0	0	0
CD_80	3	1	0	0	1	0	0	0
CDFG_53	12	0	0	0	0	4	0	0
CDFG_54	3	0	0	8	5	45	43	21
CDFG_55	1	0	0	18	4	99	14	4
CDFG_56	27	0	0	1	0	11	2	3
CDFG_69	0	0	0	0	0	0	0	1
CDFG_70	5	2	0	1	1	3	4	61
CDFG_71	8	3	0	1	0	15	2	4
HF_41	0	0	0	1	2	0	1	0
HF_42	0	0	0	2	2	0	24	0
HF_43	0	7	0	4	8	0	0	0
HF_44	0	9	0	1	0	0	1	0
HF_57	7	4	0	2	16	0	1	0
HF_58	137	21	0	6	20	0	4	4
HF_59	10	7	0	3	6	0	3	2
HF_60	28	2	0	1	1	0	3	4
HF_72	5	0	0	1	3	1	1	2

Kingdom	Bacteria	Bacteria	Bacteria	Bacteria	Bacteria	Bacteria	Bacteria	Bacteria
Phylum	Firmicutes	Firmicutes	Firmicutes	Firmicutes	Firmicutes	Firmicutes	Firmicutes	Firmicutes
Class	Erysipelotrichia	Clostridia	Clostridia	Clostridia	Clostridia	Clostridia	Clostridia	Clostridia
Order	Erysipelotrichales	Clostridiales	Clostridiales	Clostridiales	Clostridiales	Clostridiales	Clostridiales	Clostridiales
Family	Erysipelotrichaceae	Lachnospiraceae	Clostridiaceae_	Lachnospiraceae	Ruminococcaceae	Clostridiaceae_	Ruminococcaceae	Ruminococcaceae
Genus	Erysipelotrichaceae_incertae_sedis	Eisenbergiella	Alkaliphilus	Clostridium_XlVa	Intestinimonas	Alkaliphilus	Ruminococcus	Clostridium_IV
#NAME	ZOTU_0140	ZOTU_0141	ZOTU_0142	ZOTU_0143	ZOTU_0144	ZOTU_0145	ZOTU_0146	ZOTU_0147
HF_73	21	0	0	0	1	0	2	0
HF_74	0	3	0	1	3	0	1	5
HFFG_45	15	1	0	0	1	0	0	0
HFFG_46	7	1	0	5	2	0	0	3
HFFG_47	7	0	0	0	0	0	0	0
HFFG_48	2	2	0	0	2	0	0	0
HFFG_61	12	1	0	0	1	0	1	0
HFFG_62	19	2	0	1	0	0	0	0
HFFG_63	0	0	0	1	0	0	0	0
HFFG_64	11	4	0	5	3	0	4	1
HFFG_75	0	6	0	3	1	0	3	6
HFFG_76	10	5	0	4	9	0	9	1
HFFG_77	12	0	0	0	0	0	0	0

Kingdom	Bacteria	Bacteria	Bacteria	Bacteria	Bacteria	Bacteria	Bacteria	Bacteria	Bacteria
Phylum	Firmicutes	Firmicutes	Firmicutes	Firmicutes	Firmicutes	Firmicutes	Firmicutes	Actinobacteria	Firmicutes
Class	Clostridia	Clostridia	Clostridia	Clostridia	Clostridia	Clostridia	Clostridia	Actinobacteria	Clostridia
Order	Clostridiales	Clostridiales	Clostridiales	Clostridiales	Clostridiales	Clostridiales	Clostridiales	Coriobacteriales	Clostridiales
Family	Lachnospiraceae	Lachnospiraceae	Clostridiaceae_	Clostridiaceae_	Eubacteriaceae	Lachnospiraceae	Ruminococcaceae	Coriobacteriaceae	Proteinivoraceae
Genus	Anaerosporobacter	Butyrivibrio	Alkaliphilus	Alkaliphilus	Eubacterium	Clostridium_XlVa	Flavonifractor	Adlercreutzia	Proteinivorax
#NAME	ZOTU_0148	ZOTU_0149	ZOTU_0150	ZOTU_0151	ZOTU_0152	ZOTU_0153	ZOTU_0154	ZOTU_0155	ZOTU_0156
CD_49	2	1	0	9	18	3	0	3	0
CD_50	4	0	0	2	12	9	6	11	23
CD_51	3	0	0	0	14	20	0	1	13
CD_52	1	2	0	0	43	6	0	22	2
CD_65	0	0	0	1	4	5	6	2	3
CD_66	1	0	0	7	3	0	2	2	5
CD_67	0	0	0	9	14	8	0	2	3
CD_68	0	0	0	0	0	2	3	0	1
CD_78	0	0	0	1	4	6	1	0	7
CD_79	0	0	0	0	2	0	0	0	1
CD_80	0	0	0	1	0	5	0	0	0
CDFG_53	0	0	0	2	3	0	1	1	0
CDFG_54	16	0	0	1	14	3	3	26	8
CDFG_55	5	1	0	2	12	4	1	12	8
CDFG_56	1	1	0	6	2	0	0	0	3
CDFG_69	0	0	0	7	1	0	0	0	0
CDFG_70	2	0	0	5	9	1	0	0	1
CDFG_71	0	0	0	12	3	0	0	0	4
HF_41	1	0	0	0	1	0	1	0	23
HF_42	8	0	0	0	32	1	0	0	13
HF_43	12	0	0	0	1	5	0	0	10
HF_44	3	0	0	0	1	1	0	1	5
HF_57	0	0	0	0	1	0	0	0	1
HF_58	2	0	0	0	5	10	0	0	10
HF_59	2	0	0	0	9	6	0	0	2
HF_60	1	0	0	0	10	0	0	0	4
HF_72	2	0	0	0	1	1	0	8	10

Kingdom	Bacteria	Bacteria	Bacteria	Bacteria	Bacteria	Bacteria	Bacteria	Bacteria	Bacteria
Phylum	Firmicutes	Firmicutes	Firmicutes	Firmicutes	Firmicutes	Firmicutes	Firmicutes	Actinobacteria	Firmicutes
Class	Clostridia	Clostridia	Clostridia	Clostridia	Clostridia	Clostridia	Clostridia	Actinobacteria	Clostridia
Order	Clostridiales	Clostridiales	Clostridiales	Clostridiales	Clostridiales	Clostridiales	Clostridiales	Coriobacteriales	Clostridiales
Family	Lachnospiraceae	Lachnospiraceae	Clostridiaceae_	Clostridiaceae_	Eubacteriaceae	Lachnospiraceae	Ruminococcaceae	Coriobacteriaceae	Proteinivoraceae
Genus	Anaerosporobacter	Butyrivibrio	Alkaliphilus	Alkaliphilus	Eubacterium	Clostridium_XlVa	Flavonifractor	Adlercreutzia	Proteinivorax
#NAME	ZOTU_0148	ZOTU_0149	ZOTU_0150	ZOTU_0151	ZOTU_0152	ZOTU_0153	ZOTU_0154	ZOTU_0155	ZOTU_0156
HF_73	3	0	0	0	0	0	0	4	3
HF_74	4	0	0	0	0	5	5	0	4
HFFG_45	1	0	0	5	5	0	2	1	2
HFFG_46	0	0	0	0	6	1	0	0	0
HFFG_47	1	0	0	0	3	0	0	0	0
HFFG_48	0	1	0	5	2	0	2	0	0
HFFG_61	0	0	0	1	0	0	0	0	0
HFFG_62	0	0	0	30	1	3	0	6	3
HFFG_63	0	0	0	16	2	2	0	2	3
HFFG_64	1	2	0	1	0	7	0	3	0
HFFG_75	0	1	0	52	16	9	0	1	6
HFFG_76	1	2	0	54	11	4	0	3	7
HFFG_77	0	0	0	1	6	0	0	0	2

Kingdom	Bacteria	Bacteria	Bacteria	Bacteria	Bacteria	Bacteria	Bacteria
Phylum	Firmicutes	Bacteroidetes	Firmicutes	Firmicutes	Firmicutes	Firmicutes	Firmicutes
Class	Clostridia	Bacteroidia	Clostridia	Clostridia	Clostridia	Clostridia	Clostridia
Order	Clostridiales	Bacteroidales	Clostridiales	Clostridiales	Clostridiales	Clostridiales	Clostridiales
Family	Lachnospiraceae	Porphyromonadaceae	Lachnospiraceae	Lachnospiraceae	Lachnospiraceae	Lachnospiraceae	Lachnospiraceae
Genus	Lachnospiracea_incertae_sedis	Barnesiella	Clostridium_XlVa	Clostridium_XlVa	Clostridium_XlVa	Butyrivibrio	Clostridium_XlVa
#NAME	ZOTU_0157	ZOTU_0158	ZOTU_0159	ZOTU_0160	ZOTU_0161	ZOTU_0162	ZOTU_0163
CD_49	5	1	0	0	1	0	1
CD_50	18	0	0	0	0	0	3
CD_51	13	1	0	0	0	0	0
CD_52	6	0	0	0	1	2	0
CD_65	4	0	0	0	1	0	1
CD_66	0	0	0	0	0	0	1
CD_67	0	0	0	0	0	0	0
CD_68	4	0	0	0	1	0	1
CD_78	4	0	0	0	0	0	0
CD_79	1	0	0	0	0	0	0
CD_80	7	0	0	0	0	0	0
CDFG_53	2	0	0	0	0	0	0
CDFG_54	34	0	6	0	2	0	10
CDFG_55	26	0	0	0	0	1	1
CDFG_56	11	0	0	0	0	0	1
CDFG_69	1	0	0	0	0	0	0
CDFG_70	21	0	1	0	0	0	1
CDFG_71	4	0	0	1	0	0	0
HF_41	0	0	0	0	0	0	0
HF_42	7	0	0	0	0	0	4
HF_43	6	0	0	0	0	0	0
HF_44	0	0	1	0	0	0	1
HF_57	0	0	0	0	6	0	0
HF_58	2	0	0	0	30	0	0
HF_59	0	0	1	0	16	0	0
HF_60	0	0	0	0	2	0	0
HF_72	0	0	0	0	2	0	0

Kingdom	Bacteria	Bacteria	Bacteria	Bacteria	Bacteria	Bacteria	Bacteria
Phylum	Firmicutes	Bacteroidetes	Firmicutes	Firmicutes	Firmicutes	Firmicutes	Firmicutes
Class	Clostridia	Bacteroidia	Clostridia	Clostridia	Clostridia	Clostridia	Clostridia
Order	Clostridiales	Bacteroidales	Clostridiales	Clostridiales	Clostridiales	Clostridiales	Clostridiales
Family	Lachnospiraceae	Porphyromonadaceae	Lachnospiraceae	Lachnospiraceae	Lachnospiraceae	Lachnospiraceae	Lachnospiraceae
Genus	Lachnospiracea_incertae_sedis	Barnesiella	Clostridium_XlVa	Clostridium_XlVa	Clostridium_XlVa	Butyrivibrio	Clostridium_XlVa
#NAME	ZOTU_0157	ZOTU_0158	ZOTU_0159	ZOTU_0160	ZOTU_0161	ZOTU_0162	ZOTU_0163
HF_73	0	0	0	0	0	0	0
HF_74	3	0	0	0	3	0	0
HFFG_45	0	0	0	0	0	0	5
HFFG_46	0	0	0	3	0	0	0
HFFG_47	0	0	0	0	0	0	1
HFFG_48	0	0	1	3	0	0	5
HFFG_61	0	0	2	0	0	0	0
HFFG_62	0	0	1	0	0	0	3
HFFG_63	0	0	2	0	0	0	1
HFFG_64	1	0	1	0	0	3	17
HFFG_75	0	0	2	3	0	0	0
HFFG_76	1	0	12	2	0	1	11
HFFG_77	1	0	0	2	0	0	1

Kingdom	Bacteria	Bacteria	Bacteria	Bacteria	Bacteria	Bacteria	Bacteria	Bacteria
Phylum	Firmicutes	Firmicutes	Firmicutes	Firmicutes	Firmicutes	Firmicutes	Firmicutes	Tenericutes
Class	Clostridia	Clostridia	Clostridia	Clostridia	Clostridia	Clostridia	Clostridia	Mollicutes
Order	Clostridiales	Clostridiales	Clostridiales	Clostridiales	Clostridiales	Clostridiales	Clostridiales	Anaeroplasmatales
Family	Lachnospiraceae	Clostridiaceae_	Lachnospiraceae	Lachnospiraceae	Ruminococcaceae	Lachnospiraceae	Lachnospiraceae	Anaeroplasmataceae
Genus	Lachnospiracea_incertae_sedis	Geosporobacter	Clostridium_XlVa	Clostridium_XlVa	Anaerotruncus	Clostridium_XlVa	Clostridium_XlVa	Anaeroplasma
#NAME	ZOTU_0164	ZOTU_0165	ZOTU_0166	ZOTU_0167	ZOTU_0168	ZOTU_0169	ZOTU_0170	ZOTU_0171
CD_49	0	1	0	0	7	0	1	0
CD_50	0	14	1	1	7	4	0	0
CD_51	0	6	7	1	27	1	16	0
CD_52	0	10	2	1	1	0	2	2
CD_65	0	0	2	1	12	3	0	7
CD_66	0	8	0	0	0	1	1	134
CD_67	0	8	1	0	5	1	2	32
CD_68	0	10	2	0	1	1	7	17
CD_78	2	9	1	0	0	1	8	0
CD_79	0	4	0	0	0	0	0	0
CD_80	0	17	0	0	3	2	6	1
CDFG_53	0	1	0	0	0	0	0	0
CDFG_54	0	18	8	27	8	30	2	0
CDFG_55	0	10	1	3	1	34	1	0
CDFG_56	0	3	0	1	0	3	0	0
CDFG_69	0	0	0	0	0	0	0	0
CDFG_70	0	0	3	1	0	16	0	0
CDFG_71	0	0	0	0	0	0	0	0
HF_41	2	7	0	0	3	0	0	10
HF_42	0	0	1	7	12	1	2	0
HF_43	0	0	10	1	4	0	1	0
HF_44	0	1	4	0	0	0	0	0
HF_57	0	0	0	0	1	0	0	0
HF_58	0	2	4	2	15	0	14	4
HF_59	0	5	0	1	5	0	10	1
HF_60	0	7	3	1	8	0	1	0
HF_72	0	1	0	0	10	7	0	0

Kingdom	Bacteria	Bacteria	Bacteria	Bacteria	Bacteria	Bacteria	Bacteria	Bacteria
Phylum	Firmicutes	Firmicutes	Firmicutes	Firmicutes	Firmicutes	Firmicutes	Firmicutes	Tenericutes
Class	Clostridia	Clostridia	Clostridia	Clostridia	Clostridia	Clostridia	Clostridia	Mollicutes
Order	Clostridiales	Clostridiales	Clostridiales	Clostridiales	Clostridiales	Clostridiales	Clostridiales	Anaeroplasmatales
Family	Lachnospiraceae	Clostridiaceae_	Lachnospiraceae	Lachnospiraceae	Ruminococcaceae	Lachnospiraceae	Lachnospiraceae	Anaeroplasmataceae
Genus	Lachnospiracea_incertae_sedis	Geosporobacter	Clostridium_XlVa	Clostridium_XlVa	Anaerotruncus	Clostridium_XlVa	Clostridium_XlVa	Anaeroplasma
#NAME	ZOTU_0164	ZOTU_0165	ZOTU_0166	ZOTU_0167	ZOTU_0168	ZOTU_0169	ZOTU_0170	ZOTU_0171
HF_73	0	0	0	1	1	1	0	2
HF_74	0	1	2	13	8	13	1	2
HFFG_45	0	0	0	1	0	0	1	0
HFFG_46	0	3	0	8	5	0	0	0
HFFG_47	0	0	0	2	0	1	0	0
HFFG_48	0	0	0	0	2	0	1	1
HFFG_61	0	0	0	3	0	1	2	0
HFFG_62	0	4	0	1	0	0	0	0
HFFG_63	0	0	0	4	1	0	0	0
HFFG_64	0	4	0	34	0	2	0	0
HFFG_75	0	4	0	26	6	0	3	0
HFFG_76	0	7	0	30	23	0	0	0
HFFG_77	0	0	0	0	1	0	0	0

Kingdom	Bacteria	Bacteria	Bacteria	Bacteria	Bacteria	Bacteria	Bacteria	Bacteria	Bacteria
Phylum	Firmicutes	Firmicutes	Firmicutes	Firmicutes	Firmicutes	Firmicutes	Firmicutes	Firmicutes	Tenericutes
Class	Clostridia	Clostridia	Clostridia	Clostridia	Clostridia	Clostridia	Clostridia	Clostridia	Mollicutes
Order	Clostridiales	Clostridiales	Clostridiales	Clostridiales	Clostridiales	Clostridiales	Clostridiales	Clostridiales	Entomoplasmatales
Family	Clostridiaceae_	Ruminococcaceae	Lachnospiraceae	Lachnospiraceae	Ruminococcaceae	Lachnospiraceae	Lachnospiraceae	Ruminococcaceae	Spiroplasmataceae
Genus	Alkaliphilus	Oscillibacter	Acetatifactor	Blautia	Clostridium_IV	Dorea	Clostridium_XlVa	Oscillibacter	Spiroplasma
#NAME	ZOTU_0172	ZOTU_0173	ZOTU_0174	ZOTU_0175	ZOTU_0176	ZOTU_0177	ZOTU_0178	ZOTU_0179	ZOTU_0180
CD_49	8	0	0	0	1	1	1	14	0
CD_50	0	0	0	0	0	0	7	1	8
CD_51	0	2	6	0	12	0	7	22	10
CD_52	0	0	2	0	1	0	1	0	15
CD_65	0	0	1	0	1	0	5	1	0
CD_66	0	0	0	0	2	0	0	0	14
CD_67	0	0	0	0	4	0	0	10	1
CD_68	0	0	1	0	1	0	9	3	0
CD_78	0	0	0	0	0	0	0	0	7
CD_79	0	0	0	0	4	0	0	0	0
CD_80	0	2	0	0	0	2	0	0	1
CDFG_53	0	0	0	0	2	0	0	0	0
CDFG_54	0	14	5	0	10	15	21	1	0
CDFG_55	0	3	1	0	4	3	3	1	0
CDFG_56	0	0	0	0	0	0	0	0	0
CDFG_69	0	0	0	0	1	0	0	1	1
CDFG_70	0	0	0	0	2	4	4	0	5
CDFG_71	0	1	0	0	0	0	0	1	12
HF_41	1	1	0	0	0	0	0	0	0
HF_42	36	11	0	0	9	0	0	0	10
HF_43	34	0	2	0	3	0	0	0	17
HF_44	0	2	0	0	0	0	0	0	4
HF_57	0	0	0	0	0	0	0	0	1
HF_58	0	1	1	0	8	1	0	7	18
HF_59	0	0	0	0	0	0	1	3	3
HF_60	0	0	0	0	2	0	2	4	16
HF_72	0	0	1	0	0	0	3	5	2

Kingdom	Bacteria	Bacteria	Bacteria	Bacteria	Bacteria	Bacteria	Bacteria	Bacteria	Bacteria
Phylum	Firmicutes	Firmicutes	Firmicutes	Firmicutes	Firmicutes	Firmicutes	Firmicutes	Firmicutes	Tenericutes
Class	Clostridia	Clostridia	Clostridia	Clostridia	Clostridia	Clostridia	Clostridia	Clostridia	Mollicutes
Order	Clostridiales	Clostridiales	Clostridiales	Clostridiales	Clostridiales	Clostridiales	Clostridiales	Clostridiales	Entomoplasmatales
Family	Clostridiaceae_	Ruminococcaceae	Lachnospiraceae	Lachnospiraceae	Ruminococcaceae	Lachnospiraceae	Lachnospiraceae	Ruminococcaceae	Spiroplasmataceae
Genus	Alkaliphilus	Oscillibacter	Acetatifactor	Blautia	Clostridium_IV	Dorea	Clostridium_XlVa	Oscillibacter	Spiroplasma
#NAME	ZOTU_0172	ZOTU_0173	ZOTU_0174	ZOTU_0175	ZOTU_0176	ZOTU_0177	ZOTU_0178	ZOTU_0179	ZOTU_0180
HF_73	0	1	0	0	0	0	0	1	1
HF_74	0	0	1	0	0	0	2	1	4
HFFG_45	0	0	0	0	0	0	0	1	0
HFFG_46	0	0	0	0	2	0	0	4	1
HFFG_47	0	0	0	0	0	0	0	1	0
HFFG_48	0	0	0	0	0	0	0	0	0
HFFG_61	0	0	0	0	0	0	0	0	2
HFFG_62	0	0	0	0	3	0	0	0	0
HFFG_63	0	0	0	0	0	4	0	2	0
HFFG_64	0	0	0	0	1	7	0	0	2
HFFG_75	0	1	0	0	5	2	0	2	8
HFFG_76	0	3	1	0	11	9	0	5	5
HFFG_77	0	0	0	0	3	2	0	0	1

Kingdom	Bacteria	Bacteria	Bacteria	Bacteria	Bacteria	Bacteria	Bacteria	Bacteria	Bacteria
Phylum	Firmicutes	Firmicutes	Firmicutes	Firmicutes	Firmicutes	Firmicutes	Firmicutes	Firmicutes	Firmicutes
Class	Clostridia	Clostridia	Clostridia	Clostridia	Clostridia	Clostridia	Clostridia	Clostridia	Clostridia
Order	Clostridiales	Clostridiales	Clostridiales	Clostridiales	Clostridiales	Clostridiales	Clostridiales	Halanaerobiales	Clostridiales
Family	Lachnospiraceae	Lachnospiraceae	Ruminococcaceae	Lachnospiraceae	Lachnospiraceae	Lachnospiraceae	Lachnospiraceae	Halanaerobiaceae	Ruminococcaceae
Genus	Acetatifactor	Clostridium_XlVa	Butyricicoccus	Clostridium_XlVa	Clostridium_XlVa	Lachnospira	Clostridium_XlVa	Halanaerobium	Clostridium_IV
#NAME	ZOTU_0181	ZOTU_0182	ZOTU_0183	ZOTU_0184	ZOTU_0185	ZOTU_0186	ZOTU_0187	ZOTU_0188	ZOTU_0189
CD_49	3	1	1	0	0	0	1	0	0
CD_50	0	0	0	0	0	0	8	0	0
CD_51	5	1	6	0	0	0	2	0	0
CD_52	2	0	0	0	1	0	0	0	2
CD_65	2	1	0	1	0	0	2	0	0
CD_66	0	0	0	0	0	0	0	0	0
CD_67	3	14	0	0	0	0	0	0	0
CD_68	3	0	1	0	0	0	0	0	0
CD_78	0	1	0	0	0	0	0	0	0
CD_79	0	0	0	0	0	0	0	0	0
CD_80	0	0	0	0	0	0	0	0	0
CDFG_53	0	0	0	0	0	0	0	0	0
CDFG_54	5	3	0	0	0	13	5	0	1
CDFG_55	0	5	0	0	0	2	1	0	3
CDFG_56	2	0	0	0	0	0	0	0	0
CDFG_69	0	0	1	0	0	0	0	0	2
CDFG_70	0	0	0	0	0	1	0	0	3
CDFG_71	0	1	0	0	0	0	0	0	0
HF_41	0	0	0	0	0	0	0	0	1
HF_42	1	9	5	0	0	0	11	0	4
HF_43	0	8	1	0	0	0	2	0	0
HF_44	0	1	0	0	0	0	0	0	0
HF_57	3	2	0	2	3	0	0	0	0
HF_58	19	9	2	24	22	0	3	0	0
HF_59	6	2	3	12	8	0	0	0	0
HF_60	4	0	3	0	0	0	0	0	0
HF_72	4	0	1	0	2	0	0	0	12

Kingdom	Bacteria	Bacteria	Bacteria	Bacteria	Bacteria	Bacteria	Bacteria	Bacteria	Bacteria
Phylum	Firmicutes	Firmicutes	Firmicutes	Firmicutes	Firmicutes	Firmicutes	Firmicutes	Firmicutes	Firmicutes
Class	Clostridia	Clostridia	Clostridia	Clostridia	Clostridia	Clostridia	Clostridia	Clostridia	Clostridia
Order	Clostridiales	Clostridiales	Clostridiales	Clostridiales	Clostridiales	Clostridiales	Clostridiales	Halanaerobiales	Clostridiales
Family	Lachnospiraceae	Lachnospiraceae	Ruminococcaceae	Lachnospiraceae	Lachnospiraceae	Lachnospiraceae	Lachnospiraceae	Halanaerobiaceae	Ruminococcaceae
Genus	Acetatifactor	Clostridium_XlVa	Butyricicoccus	Clostridium_XlVa	Clostridium_XlVa	Lachnospira	Clostridium_XlVa	Halanaerobium	Clostridium_IV
#NAME	ZOTU_0181	ZOTU_0182	ZOTU_0183	ZOTU_0184	ZOTU_0185	ZOTU_0186	ZOTU_0187	ZOTU_0188	ZOTU_0189
HF_73	0	0	0	0	0	0	0	0	2
HF_74	4	4	0	0	3	0	2	0	2
HFFG_45	0	0	3	0	0	0	0	0	0
HFFG_46	0	0	5	0	0	2	0	0	0
HFFG_47	0	0	0	0	0	0	0	0	0
HFFG_48	0	0	0	0	0	1	0	0	0
HFFG_61	0	0	0	0	0	3	0	0	2
HFFG_62	0	0	2	0	0	1	0	24	1
HFFG_63	1	0	0	0	0	2	0	0	0
HFFG_64	4	0	16	0	0	5	2	0	0
HFFG_75	0	0	7	0	0	5	2	0	0
HFFG_76	2	0	3	0	0	10	1	0	3
HFFG_77	0	1	2	0	0	0	2	0	6

Kingdom	Bacteria	Bacteria	Bacteria	Bacteria	Bacteria	Bacteria	Bacteria	Bacteria	Bacteria
Phylum	Actinobacteria	Firmicutes	Firmicutes	Firmicutes	Firmicutes	Firmicutes	Firmicutes	Firmicutes	Firmicutes
Class	Actinobacteria	Clostridia	Clostridia	Clostridia	Clostridia	Clostridia	Clostridia	Clostridia	Clostridia
Order	Actinomycetales	Clostridiales	Clostridiales	Clostridiales	Clostridiales	Clostridiales	Clostridiales	Clostridiales	Clostridiales
Family	Streptomycetaceae	Lachnospiraceae	Lachnospiraceae	Lachnospiraceae	Ruminococcaceae	Ruminococcaceae	Ruminococcaceae	Lachnospiraceae	Lachnospiraceae
Genus	Streptomyces	Ruminococcus	Roseburia	Clostridium_XlVa	Clostridium_III	Oscillibacter	Anaerotruncus	Clostridium_XlVa	Clostridium_XlVa
#NAME	ZOTU_0190	ZOTU_0191	ZOTU_0192	ZOTU_0193	ZOTU_0194	ZOTU_0195	ZOTU_0196	ZOTU_0197	ZOTU_0198
CD_49	0	0	0	0	0	0	2	0	1
CD_50	0	2	0	1	0	2	9	0	0
CD_51	0	0	1	0	0	2	16	0	5
CD_52	0	2	0	0	0	0	1	0	1
CD_65	0	3	0	0	0	2	6	0	0
CD_66	0	0	0	0	0	0	1	0	1
CD_67	0	0	0	0	0	1	5	0	0
CD_68	0	0	2	0	0	0	4	0	1
CD_78	0	1	0	0	0	0	0	0	0
CD_79	0	0	0	0	0	0	0	0	0
CD_80	0	0	0	0	0	0	0	0	0
CDFG_53	1	0	0	0	0	0	0	0	0
CDFG_54	0	0	2	0	0	6	2	0	0
CDFG_55	0	0	0	0	0	4	0	1	0
CDFG_56	0	0	0	0	2	0	0	1	0
CDFG_69	0	0	0	0	3	0	0	0	0
CDFG_70	0	0	0	0	1	5	0	0	0
CDFG_71	0	0	1	0	3	0	1	0	0
HF_41	0	0	0	0	0	4	2	0	0
HF_42	0	1	0	0	0	11	6	0	14
HF_43	0	0	0	0	7	5	8	0	4
HF_44	0	1	0	0	0	12	4	0	2
HF_57	6	0	1	6	0	0	2	0	0
HF_58	26	12	4	15	0	10	8	0	1
HF_59	1	8	8	7	0	5	6	0	0
HF_60	0	0	0	1	0	2	3	0	0
HF_72	0	1	1	1	0	1	8	0	1

Kingdom	Bacteria	Bacteria	Bacteria	Bacteria	Bacteria	Bacteria	Bacteria	Bacteria	Bacteria
Phylum	Actinobacteria	Firmicutes	Firmicutes	Firmicutes	Firmicutes	Firmicutes	Firmicutes	Firmicutes	Firmicutes
Class	Actinobacteria	Clostridia	Clostridia	Clostridia	Clostridia	Clostridia	Clostridia	Clostridia	Clostridia
Order	Actinomycetales	Clostridiales	Clostridiales	Clostridiales	Clostridiales	Clostridiales	Clostridiales	Clostridiales	Clostridiales
Family	Streptomycetaceae	Lachnospiraceae	Lachnospiraceae	Lachnospiraceae	Ruminococcaceae	Ruminococcaceae	Ruminococcaceae	Lachnospiraceae	Lachnospiraceae
Genus	Streptomyces	Ruminococcus	Roseburia	Clostridium_XlVa	Clostridium_III	Oscillibacter	Anaerotruncus	Clostridium_XlVa	Clostridium_XlVa
#NAME	ZOTU_0190	ZOTU_0191	ZOTU_0192	ZOTU_0193	ZOTU_0194	ZOTU_0195	ZOTU_0196	ZOTU_0197	ZOTU_0198
HF_73	0	3	0	0	0	1	0	0	0
HF_74	1	7	1	2	0	2	0	0	2
HFFG_45	0	0	0	0	0	0	0	1	0
HFFG_46	0	1	0	0	0	2	0	0	1
HFFG_47	0	0	0	0	0	0	0	5	0
HFFG_48	0	0	0	0	3	3	4	21	0
HFFG_61	0	0	1	0	0	1	0	0	0
HFFG_62	0	0	0	0	0	0	1	0	0
HFFG_63	2	0	0	0	0	0	0	0	0
HFFG_64	9	0	1	0	0	1	0	0	0
HFFG_75	1	0	1	0	15	7	11	0	0
HFFG_76	1	0	0	0	7	16	13	0	2
HFFG_77	0	0	0	0	4	2	1	0	0

Kingdom	Bacteria	Bacteria	Bacteria	Bacteria	Bacteria	Bacteria	Bacteria	Bacteria	Bacteria
Phylum	Firmicutes	Firmicutes	Firmicutes	Firmicutes	Firmicutes	Firmicutes	Firmicutes	Firmicutes	Firmicutes
Class	Clostridia	Clostridia	Clostridia	Clostridia	Clostridia	Clostridia	Clostridia	Clostridia	Clostridia
Order	Clostridiales	Clostridiales	Clostridiales	Clostridiales	Clostridiales	Clostridiales	Clostridiales	Clostridiales	Clostridiales
Family	Ruminococcaceae	Lachnospiraceae	Lachnospiraceae	Lachnospiraceae	Lachnospiraceae	Ruminococcaceae	Lachnospiraceae	Lachnospiraceae	Ruminococcaceae
Genus	Intestinimonas	Clostridium_XlVa	Acetatifactor	Acetatifactor	Clostridium_XlVa	Anaerotruncus	Clostridium_XlVa	Clostridium_XlVa	Hydrogenoanaerobacterium
#NAME	ZOTU_0199	ZOTU_0200	ZOTU_0201	ZOTU_0202	ZOTU_0203	ZOTU_0204	ZOTU_0205	ZOTU_0206	ZOTU_0207
CD_49	0	0	0	1	1	6	0	0	0
CD_50	0	2	0	0	0	16	1	1	1
CD_51	18	6	1	1	14	20	0	3	3
CD_52	0	1	0	0	0	5	0	1	1
CD_65	0	0	0	0	0	7	0	0	0
CD_66	0	2	0	0	0	6	0	0	2
CD_67	0	0	0	0	0	3	0	0	9
CD_68	1	0	0	0	0	1	0	1	0
CD_78	0	0	0	0	0	6	0	0	0
CD_79	0	0	0	0	0	2	0	0	0
CD_80	0	0	0	0	0	3	0	2	3
CDFG_53	0	2	0	0	0	0	0	0	0
CDFG_54	9	19	2	4	0	3	0	10	11
CDFG_55	2	9	0	0	0	1	0	0	0
CDFG_56	2	1	0	0	0	0	0	3	0
CDFG_69	1	1	0	0	0	0	0	1	1
CDFG_70	1	11	0	0	0	0	0	1	0
CDFG_71	2	2	0	0	0	0	0	1	0
HF_41	0	0	0	0	25	1	0	0	0
HF_42	1	7	0	0	20	1	0	12	2
HF_43	2	0	1	4	0	3	0	0	1
HF_44	1	0	0	0	3	4	0	0	1
HF_57	0	0	1	2	0	1	0	0	3
HF_58	0	0	0	1	0	4	0	0	10
HF_59	0	0	0	0	0	2	0	0	4
HF_60	3	0	0	0	0	0	0	0	4
HF_72	0	0	1	0	0	1	0	0	3

Kingdom	Bacteria	Bacteria	Bacteria	Bacteria	Bacteria	Bacteria	Bacteria	Bacteria	Bacteria
Phylum	Firmicutes	Firmicutes	Firmicutes	Firmicutes	Firmicutes	Firmicutes	Firmicutes	Firmicutes	Firmicutes
Class	Clostridia	Clostridia	Clostridia	Clostridia	Clostridia	Clostridia	Clostridia	Clostridia	Clostridia
Order	Clostridiales	Clostridiales	Clostridiales	Clostridiales	Clostridiales	Clostridiales	Clostridiales	Clostridiales	Clostridiales
Family	Ruminococcaceae	Lachnospiraceae	Lachnospiraceae	Lachnospiraceae	Lachnospiraceae	Ruminococcaceae	Lachnospiraceae	Lachnospiraceae	Ruminococcaceae
Genus	Intestinimonas	Clostridium_XlVa	Acetatifactor	Acetatifactor	Clostridium_XlVa	Anaerotruncus	Clostridium_XlVa	Clostridium_XlVa	Hydrogenoanaerobacterium
#NAME	ZOTU_0199	ZOTU_0200	ZOTU_0201	ZOTU_0202	ZOTU_0203	ZOTU_0204	ZOTU_0205	ZOTU_0206	ZOTU_0207
HF_73	0	0	0	0	0	0	0	0	0
HF_74	3	0	1	1	0	2	0	1	5
HFFG_45	2	0	0	0	7	1	0	0	0
HFFG_46	0	0	0	0	0	1	0	0	1
HFFG_47	0	0	0	0	5	0	0	0	0
HFFG_48	0	0	0	0	0	2	0	0	0
HFFG_61	0	0	0	0	0	0	0	0	0
HFFG_62	0	0	0	0	0	0	0	0	0
HFFG_63	0	0	0	0	0	0	0	0	1
HFFG_64	2	0	0	0	0	1	0	0	4
HFFG_75	2	0	0	0	44	0	0	0	4
HFFG_76	3	1	0	0	7	0	0	0	10
HFFG_77	0	0	0	0	6	0	0	0	1

Kingdom	Bacteria	Bacteria	Bacteria	Bacteria	Bacteria	Bacteria	Bacteria	Bacteria	Bacteria
Phylum	Firmicutes	Firmicutes	Firmicutes	Firmicutes	Firmicutes	Firmicutes	Firmicutes	Firmicutes	Firmicutes
Class	Clostridia	Clostridia	Clostridia	Clostridia	Clostridia	Clostridia	Clostridia	Clostridia	Clostridia
Order	Clostridiales	Clostridiales	Clostridiales	Clostridiales	Clostridiales	Clostridiales	Clostridiales	Clostridiales	Clostridiales
Family	Lachnospiraceae	Clostridiaceae_	Clostridiaceae_	Lachnospiraceae	Clostridiaceae_	Lachnospiraceae	Peptostreptococcaceae	Ruminococcaceae	Ruminococcaceae
Genus	Clostridium_XlVa	Alkaliphilus	Alkaliphilus	Clostridium_XlVa	Alkaliphilus	Acetatifactor	Filifactor	Intestinimonas	Ruminococcus
#NAME	ZOTU_0208	ZOTU_0209	ZOTU_0210	ZOTU_0211	ZOTU_0212	ZOTU_0213	ZOTU_0214	ZOTU_0215	ZOTU_0216
CD_49	0	10	0	0	0	0	0	2	0
CD_50	3	2	0	2	7	2	0	4	0
CD_51	1	20	0	1	0	0	0	16	7
CD_52	1	3	0	1	56	0	0	0	0
CD_65	2	3	0	0	0	0	0	4	0
CD_66	0	11	0	0	5	0	0	1	0
CD_67	0	12	0	0	21	0	0	0	1
CD_68	4	9	0	0	0	1	0	0	0
CD_78	0	1	0	0	0	0	0	0	0
CD_79	0	2	0	0	0	0	0	0	0
CD_80	0	1	0	0	15	0	0	1	0
CDFG_53	0	0	0	0	3	1	0	0	1
CDFG_54	3	0	0	10	0	4	0	7	17
CDFG_55	4	0	0	4	1	12	0	3	3
CDFG_56	0	0	0	1	2	0	0	2	1
CDFG_69	2	0	8	2	0	0	0	1	0
CDFG_70	0	0	1	2	0	0	0	1	0
CDFG_71	1	0	1	0	0	1	0	2	0
HF_41	0	1	0	0	1	0	0	3	0
HF_42	19	1	0	4	0	1	0	4	0
HF_43	9	0	0	0	0	1	0	2	0
HF_44	0	0	1	0	0	0	0	2	0
HF_57	1	0	0	0	0	0	0	0	0
HF_58	2	0	0	0	0	0	0	1	0
HF_59	0	0	0	0	0	0	0	1	1
HF_60	1	0	0	0	0	1	0	1	0
HF_72	0	0	0	0	0	0	0	4	0

Kingdom	Bacteria	Bacteria	Bacteria	Bacteria	Bacteria	Bacteria	Bacteria	Bacteria	Bacteria
Phylum	Firmicutes	Firmicutes	Firmicutes	Firmicutes	Firmicutes	Firmicutes	Firmicutes	Firmicutes	Firmicutes
Class	Clostridia	Clostridia	Clostridia	Clostridia	Clostridia	Clostridia	Clostridia	Clostridia	Clostridia
Order	Clostridiales	Clostridiales	Clostridiales	Clostridiales	Clostridiales	Clostridiales	Clostridiales	Clostridiales	Clostridiales
Family	Lachnospiraceae	Clostridiaceae_	Clostridiaceae_	Lachnospiraceae	Clostridiaceae_	Lachnospiraceae	Peptostreptococcaceae	Ruminococcaceae	Ruminococcaceae
Genus	Clostridium_XlVa	Alkaliphilus	Alkaliphilus	Clostridium_XlVa	Alkaliphilus	Acetatifactor	Filifactor	Intestinimonas	Ruminococcus
#NAME	ZOTU_0208	ZOTU_0209	ZOTU_0210	ZOTU_0211	ZOTU_0212	ZOTU_0213	ZOTU_0214	ZOTU_0215	ZOTU_0216
HF_73	0	0	0	0	0	0	0	0	0
HF_74	0	1	0	1	0	0	0	2	2
HFFG_45	0	0	0	0	0	0	0	1	1
HFFG_46	0	0	0	1	0	0	0	1	0
HFFG_47	0	0	0	0	0	0	0	0	0
HFFG_48	0	0	0	0	0	0	0	0	0
HFFG_61	0	0	0	0	0	0	0	0	0
HFFG_62	0	0	0	0	0	0	0	1	0
HFFG_63	0	0	0	0	0	0	0	4	0
HFFG_64	1	0	0	5	0	1	0	0	1
HFFG_75	0	0	0	3	0	1	0	3	2
HFFG_76	0	0	0	4	0	2	0	3	20
HFFG_77	0	0	0	1	0	0	0	4	2

Kingdom	Bacteria	Bacteria	Bacteria	Bacteria	Bacteria	Bacteria	Bacteria	Bacteria
Phylum	Firmicutes	Firmicutes	Firmicutes	Bacteroidetes	Firmicutes	Actinobacteria	Firmicutes	Firmicutes
Class	Clostridia	Clostridia	Clostridia	Bacteroidia	Clostridia	Actinobacteria	Clostridia	Clostridia
Order	Clostridiales	Clostridiales	Clostridiales	Bacteroidales	Clostridiales	Coriobacteriales	Clostridiales	Clostridiales
Family	Lachnospiraceae	Ruminococcaceae	Lachnospiraceae	Porphyromonadaceae	Ruminococcaceae	Coriobacteriaceae	Ruminococcaceae	Ruminococcaceae
Genus	Lachnospiracea_incertae_sedis	Sporobacter	Acetatifactor	Barnesiella	Sporobacter	Eggerthella	Anaerotruncus	Clostridium_IV
#NAME	ZOTU_0217	ZOTU_0218	ZOTU_0219	ZOTU_0220	ZOTU_0221	ZOTU_0222	ZOTU_0223	ZOTU_0224
CD_49	0	0	2	0	0	0	0	0
CD_50	0	0	1	0	0	43	2	0
CD_51	0	0	3	0	0	2	0	2
CD_52	0	0	1	0	0	1	0	0
CD_65	2	0	1	0	0	0	2	0
CD_66	1	0	0	0	0	1	0	0
CD_67	2	0	0	0	0	0	1	1
CD_68	11	0	1	0	0	0	0	1
CD_78	0	0	0	0	0	0	0	0
CD_79	0	0	0	0	0	0	0	0
CD_80	0	0	1	0	0	0	0	0
CDFG_53	0	0	0	0	0	0	0	0
CDFG_54	0	0	3	0	0	1	1	2
CDFG_55	0	0	2	0	0	0	7	0
CDFG_56	0	0	0	0	0	0	0	0
CDFG_69	3	0	0	0	0	8	0	0
CDFG_70	1	0	0	0	0	1	0	0
CDFG_71	6	0	0	0	0	6	0	0
HF_41	0	37	0	0	0	0	0	0
HF_42	0	9	5	0	0	0	0	6
HF_43	0	0	6	0	0	0	0	0
HF_44	0	0	0	0	0	0	0	1
HF_57	0	0	0	0	1	0	4	0
HF_58	0	0	0	0	13	0	15	12
HF_59	0	0	0	0	2	0	4	4
HF_60	0	0	0	0	5	1	3	0
HF_72	0	0	2	0	0	0	2	1

Kingdom	Bacteria	Bacteria	Bacteria	Bacteria	Bacteria	Bacteria	Bacteria	Bacteria
Phylum	Firmicutes	Firmicutes	Firmicutes	Bacteroidetes	Firmicutes	Actinobacteria	Firmicutes	Firmicutes
Class	Clostridia	Clostridia	Clostridia	Bacteroidia	Clostridia	Actinobacteria	Clostridia	Clostridia
Order	Clostridiales	Clostridiales	Clostridiales	Bacteroidales	Clostridiales	Coriobacteriales	Clostridiales	Clostridiales
Family	Lachnospiraceae	Ruminococcaceae	Lachnospiraceae	Porphyromonadaceae	Ruminococcaceae	Coriobacteriaceae	Ruminococcaceae	Ruminococcaceae
Genus	Lachnospiracea_incertae_sedis	Sporobacter	Acetatifactor	Barnesiella	Sporobacter	Eggerthella	Anaerotruncus	Clostridium_IV
#NAME	ZOTU_0217	ZOTU_0218	ZOTU_0219	ZOTU_0220	ZOTU_0221	ZOTU_0222	ZOTU_0223	ZOTU_0224
HF_73	0	0	0	0	0	0	1	0
HF_74	0	0	0	0	0	0	1	2
HFFG_45	0	0	0	0	0	0	1	0
HFFG_46	0	0	0	0	1	0	0	0
HFFG_47	0	0	0	0	0	0	0	0
HFFG_48	0	0	0	0	0	0	3	0
HFFG_61	13	0	0	0	1	1	3	1
HFFG_62	1	0	0	0	2	14	0	1
HFFG_63	6	0	1	0	1	2	0	0
HFFG_64	10	0	1	0	7	0	5	1
HFFG_75	0	0	2	0	0	26	0	5
HFFG_76	0	0	1	0	3	1	2	3
HFFG_77	0	0	0	0	0	1	3	0

Kingdom	Bacteria	Bacteria	Bacteria	Bacteria	Bacteria	Bacteria	Bacteria	Bacteria	Bacteria
Phylum	Firmicutes	Firmicutes	Firmicutes	Firmicutes	Firmicutes	Bacteroidetes	Firmicutes	Firmicutes	Firmicutes
Class	Clostridia	Clostridia	Clostridia	Clostridia	Clostridia	Bacteroidia	Clostridia	Clostridia	Clostridia
Order	Clostridiales	Clostridiales	Clostridiales	Clostridiales	Clostridiales	Bacteroidales	Clostridiales	Clostridiales	Clostridiales
Family	Lachnospiraceae	Ruminococcaceae	Lachnospiraceae	Clostridiaceae_	Lachnospiraceae	Rikenellaceae	Ruminococcaceae	Lachnospiraceae	Lachnospiraceae
Genus	Clostridium_XlVa	Hydrogenoanaerobacterium	Acetatifactor	Alkaliphilus	Clostridium_XlVa	Alistipes	Acetanaerobacterium	Clostridium_XlVb	Clostridium_XlVa
#NAME	ZOTU_0225	ZOTU_0226	ZOTU_0227	ZOTU_0228	ZOTU_0229	ZOTU_0230	ZOTU_0231	ZOTU_0232	ZOTU_0233
CD_49	0	4	0	0	0	0	5	0	0
CD_50	0	8	0	0	0	0	1	0	0
CD_51	0	7	6	0	0	0	4	1	0
CD_52	0	2	0	0	0	0	0	0	0
CD_65	0	2	0	0	0	0	2	0	0
CD_66	0	0	1	0	0	0	2	0	0
CD_67	0	3	1	0	0	0	2	0	0
CD_68	0	1	2	0	0	0	1	0	0
CD_78	0	0	0	0	0	0	2	3	0
CD_79	0	0	0	0	0	0	0	0	0
CD_80	0	0	0	0	0	0	5	0	0
CDFG_53	0	0	0	0	0	0	1	0	0
CDFG_54	0	5	1	0	0	0	35	2	0
CDFG_55	0	0	0	0	0	0	8	0	0
CDFG_56	0	1	0	0	0	0	5	0	0
CDFG_69	0	0	2	0	0	0	1	1	0
CDFG_70	0	2	0	0	0	0	4	1	0
CDFG_71	0	0	0	0	0	0	0	0	0
HF_41	0	0	0	0	0	0	3	0	0
HF_42	0	5	0	0	34	25	10	0	0
HF_43	0	1	3	0	1	77	1	0	0
HF_44	0	0	1	0	3	3	0	0	0
HF_57	0	0	2	0	0	0	0	0	0
HF_58	0	2	16	0	0	0	4	2	3
HF_59	0	0	6	0	0	0	0	0	1
HF_60	0	1	6	0	0	0	0	1	0
HF_72	0	1	0	0	0	6	0	0	0

Kingdom	Bacteria	Bacteria	Bacteria	Bacteria	Bacteria	Bacteria	Bacteria	Bacteria	Bacteria
Phylum	Firmicutes	Firmicutes	Firmicutes	Firmicutes	Firmicutes	Bacteroidetes	Firmicutes	Firmicutes	Firmicutes
Class	Clostridia	Clostridia	Clostridia	Clostridia	Clostridia	Bacteroidia	Clostridia	Clostridia	Clostridia
Order	Clostridiales	Clostridiales	Clostridiales	Clostridiales	Clostridiales	Bacteroidales	Clostridiales	Clostridiales	Clostridiales
Family	Lachnospiraceae	Ruminococcaceae	Lachnospiraceae	Clostridiaceae_	Lachnospiraceae	Rikenellaceae	Ruminococcaceae	Lachnospiraceae	Lachnospiraceae
Genus	Clostridium_XlVa	Hydrogenoanaerobacterium	Acetatifactor	Alkaliphilus	Clostridium_XlVa	Alistipes	Acetanaerobacterium	Clostridium_XlVb	Clostridium_XlVa
#NAME	ZOTU_0225	ZOTU_0226	ZOTU_0227	ZOTU_0228	ZOTU_0229	ZOTU_0230	ZOTU_0231	ZOTU_0232	ZOTU_0233
HF_73	0	2	0	0	0	7	0	0	0
HF_74	0	0	6	0	1	20	1	0	0
HFFG_45	0	0	0	0	0	0	0	0	0
HFFG_46	0	2	0	0	0	0	1	0	0
HFFG_47	0	0	0	0	0	0	0	0	0
HFFG_48	0	0	0	0	0	0	1	0	0
HFFG_61	0	0	0	0	0	0	0	0	0
HFFG_62	0	0	0	0	0	0	0	1	0
HFFG_63	0	0	0	0	1	0	0	0	0
HFFG_64	0	2	7	0	0	0	14	0	1
HFFG_75	0	2	0	0	0	0	7	0	0
HFFG_76	0	6	0	0	4	0	5	1	0
HFFG_77	0	0	1	0	0	0	0	0	0

Kingdom	Bacteria	Bacteria	Bacteria	Bacteria	Bacteria	Bacteria	Bacteria	Bacteria	Bacteria
Phylum	Firmicutes	Actinobacteria	Firmicutes	Firmicutes	Firmicutes	Firmicutes	Firmicutes	Firmicutes	Firmicutes
Class	Clostridia	Actinobacteria	Clostridia	Clostridia	Clostridia	Clostridia	Clostridia	Clostridia	Clostridia
Order	Clostridiales	Actinomycetales	Clostridiales	Clostridiales	Clostridiales	Clostridiales	Clostridiales	Clostridiales	Clostridiales
Family	Clostridiaceae_	Streptomycetaceae	Lachnospiraceae	Lachnospiraceae	Lachnospiraceae	Lachnospiraceae	Lachnospiraceae	Clostridiaceae_	Lachnospiraceae
Genus	Alkaliphilus	Streptomyces	Clostridium_XlVa	Lachnospiracea_incertae_sedis	Clostridium_XlVa	Butyrivibrio	Clostridium_XlVa	Alkaliphilus	Clostridium_XlVa
#NAME	ZOTU_0234	ZOTU_0235	ZOTU_0236	ZOTU_0237	ZOTU_0238	ZOTU_0239	ZOTU_0240	ZOTU_0241	ZOTU_0242
CD_49	0	0	0	0	0	1	0	0	0
CD_50	0	0	4	0	0	1	0	0	0
CD_51	0	0	6	0	0	0	0	0	6
CD_52	0	0	0	0	0	2	0	0	0
CD_65	0	0	1	0	0	0	1	0	0
CD_66	0	0	0	0	0	1	0	0	0
CD_67	0	0	1	0	0	0	0	0	0
CD_68	0	0	0	0	0	0	0	0	0
CD_78	0	0	0	0	0	0	0	0	0
CD_79	0	0	0	0	0	0	0	0	0
CD_80	0	0	0	0	0	3	0	0	0
CDFG_53	0	0	0	0	0	0	0	0	0
CDFG_54	0	0	10	5	0	1	0	0	0
CDFG_55	0	0	1	5	0	1	0	0	0
CDFG_56	0	0	0	0	0	1	0	0	0
CDFG_69	0	0	0	0	0	0	0	0	0
CDFG_70	0	0	0	2	0	1	0	0	0
CDFG_71	0	0	0	0	0	0	0	0	0
HF_41	0	0	0	0	0	0	0	4	0
HF_42	0	0	21	0	0	0	0	8	3
HF_43	0	0	2	6	0	0	0	0	5
HF_44	0	1	0	2	0	0	0	0	0
HF_57	0	0	0	0	1	3	0	0	0
HF_58	0	0	0	2	4	15	0	0	0
HF_59	0	0	0	2	0	4	0	0	0
HF_60	0	0	1	0	0	7	0	0	0
HF_72	0	0	0	0	0	0	0	0	0

Kingdom	Bacteria	Bacteria	Bacteria	Bacteria	Bacteria	Bacteria	Bacteria	Bacteria	Bacteria
Phylum	Firmicutes	Actinobacteria	Firmicutes	Firmicutes	Firmicutes	Firmicutes	Firmicutes	Firmicutes	Firmicutes
Class	Clostridia	Actinobacteria	Clostridia	Clostridia	Clostridia	Clostridia	Clostridia	Clostridia	Clostridia
Order	Clostridiales	Actinomycetales	Clostridiales	Clostridiales	Clostridiales	Clostridiales	Clostridiales	Clostridiales	Clostridiales
Family	Clostridiaceae_	Streptomycetaceae	Lachnospiraceae	Lachnospiraceae	Lachnospiraceae	Lachnospiraceae	Lachnospiraceae	Clostridiaceae_	Lachnospiraceae
Genus	Alkaliphilus	Streptomyces	Clostridium_XlVa	Lachnospiracea_incertae_sedis	Clostridium_XlVa	Butyrivibrio	Clostridium_XlVa	Alkaliphilus	Clostridium_XlVa
#NAME	ZOTU_0234	ZOTU_0235	ZOTU_0236	ZOTU_0237	ZOTU_0238	ZOTU_0239	ZOTU_0240	ZOTU_0241	ZOTU_0242
HF_73	0	0	0	1	0	0	0	0	0
HF_74	0	4	0	9	0	3	0	0	2
HFFG_45	0	0	0	0	0	0	0	0	0
HFFG_46	0	0	0	0	0	0	3	0	0
HFFG_47	0	0	0	0	0	0	0	0	0
HFFG_48	0	0	0	2	0	0	0	0	0
HFFG_61	0	0	0	4	2	1	0	0	0
HFFG_62	0	0	0	4	0	0	0	0	0
HFFG_63	0	0	0	1	0	0	0	0	0
HFFG_64	0	0	0	7	6	2	0	0	0
HFFG_75	0	0	2	13	0	0	0	0	1
HFFG_76	0	0	0	9	0	3	0	0	2
HFFG_77	0	0	0	0	0	0	12	0	0

Kingdom	Bacteria	Bacteria	Bacteria	Bacteria	Bacteria	Bacteria	Bacteria
Phylum	Actinobacteria	Firmicutes	Firmicutes	Firmicutes	Firmicutes	Firmicutes	Firmicutes
Class	Actinobacteria	Clostridia	Clostridia	Clostridia	Erysipelotrichia	Clostridia	Clostridia
Order	Coriobacteriales	Clostridiales	Clostridiales	Clostridiales	Erysipelotrichales	Clostridiales	Clostridiales
Family	Coriobacteriaceae	Lachnospiraceae	Lachnospiraceae	Ruminococcaceae	Erysipelotrichaceae	Lachnospiraceae	Lachnospiraceae
Genus	Enterorhabdus	Lachnospiracea_incertae_sedis	Lachnospiracea_incertae_sedis	Clostridium_IV	Catenisphaera	Clostridium_XlVa	Roseburia
#NAME	ZOTU_0243	ZOTU_0244	ZOTU_0245	ZOTU_0246	ZOTU_0247	ZOTU_0248	ZOTU_0249
CD_49	3	0	1	0	0	0	0
CD_50	1	0	0	1	0	0	0
CD_51	1	1	3	12	0	0	0
CD_52	2	0	0	2	1	0	0
CD_65	1	0	0	1	1	0	0
CD_66	1	0	0	2	0	0	0
CD_67	2	0	0	2	0	0	0
CD_68	1	0	0	3	1	0	0
CD_78	0	0	0	0	0	0	0
CD_79	0	0	0	0	0	0	0
CD_80	1	0	0	0	1	0	0
CDFG_53	0	0	0	0	1	0	0
CDFG_54	1	11	0	6	1	12	0
CDFG_55	3	6	4	4	0	1	0
CDFG_56	2	1	1	1	0	0	0
CDFG_69	0	0	0	0	0	1	0
CDFG_70	0	1	0	0	0	0	0
CDFG_71	1	0	0	0	0	2	0
HF_41	8	0	0	3	0	0	1
HF_42	1	1	4	7	0	1	0
HF_43	4	1	0	0	0	0	0
HF_44	0	1	0	2	0	0	0
HF_57	1	0	0	0	0	0	0
HF_58	8	0	2	5	0	0	1
HF_59	0	0	7	0	0	0	0
HF_60	1	0	2	2	0	0	0
HF_72	33	0	0	0	0	0	0

Kingdom	Bacteria	Bacteria	Bacteria	Bacteria	Bacteria	Bacteria	Bacteria
Phylum	Actinobacteria	Firmicutes	Firmicutes	Firmicutes	Firmicutes	Firmicutes	Firmicutes
Class	Actinobacteria	Clostridia	Clostridia	Clostridia	Erysipelotrichia	Clostridia	Clostridia
Order	Coriobacteriales	Clostridiales	Clostridiales	Clostridiales	Erysipelotrichales	Clostridiales	Clostridiales
Family	Coriobacteriaceae	Lachnospiraceae	Lachnospiraceae	Ruminococcaceae	Erysipelotrichaceae	Lachnospiraceae	Lachnospiraceae
Genus	Enterorhabdus	Lachnospiracea_incertae_sedis	Lachnospiracea_incertae_sedis	Clostridium_IV	Catenisphaera	Clostridium_XlVa	Roseburia
#NAME	ZOTU_0243	ZOTU_0244	ZOTU_0245	ZOTU_0246	ZOTU_0247	ZOTU_0248	ZOTU_0249
HF_73	6	0	1	0	0	0	0
HF_74	2	2	0	2	0	0	0
HFFG_45	0	0	1	3	0	0	0
HFFG_46	0	0	0	1	0	0	0
HFFG_47	0	0	0	0	0	0	0
HFFG_48	0	0	1	1	0	0	0
HFFG_61	0	0	0	0	0	0	0
HFFG_62	2	0	0	0	0	0	0
HFFG_63	0	0	0	0	0	0	0
HFFG_64	0	2	0	0	0	0	0
HFFG_75	0	0	1	2	0	0	0
HFFG_76	4	1	3	6	0	0	0
HFFG_77	0	0	0	0	0	0	0

Kingdom	Bacteria	Bacteria	Bacteria	Bacteria	Bacteria	Bacteria	Bacteria	Bacteria	Bacteria
Phylum	Firmicutes	Firmicutes	Firmicutes	Firmicutes	Firmicutes	Firmicutes	Firmicutes	Firmicutes	Firmicutes
Class	Clostridia	Clostridia	Clostridia	Bacilli	Clostridia	Clostridia	Clostridia	Clostridia	Clostridia
Order	Clostridiales	Clostridiales	Clostridiales	Bacillales	Clostridiales	Clostridiales	Clostridiales	Clostridiales	Clostridiales
Family	Lachnospiraceae	Peptostreptococcaceae	Lachnospiraceae	Staphylococcaceae	Lachnospiraceae	Lachnospiraceae	Lachnospiraceae	Lachnospiraceae	Lachnospiraceae
Genus	Marvinbryantia	Filifactor	Acetatifactor	Staphylococcus	Clostridium_XlVa	Clostridium_XlVa	Butyrivibrio	Clostridium_XlVa	Clostridium_XlVb
#NAME	ZOTU_0250	ZOTU_0251	ZOTU_0252	ZOTU_0253	ZOTU_0254	ZOTU_0255	ZOTU_0256	ZOTU_0257	ZOTU_0258
CD_49	0	26	0	25	0	0	0	0	0
CD_50	12	1	2	5	2	0	6	0	0
CD_51	0	14	1	83	1	0	0	0	4
CD_52	0	11	0	2	0	0	0	0	0
CD_65	0	0	0	0	1	1	4	0	0
CD_66	0	0	1	0	0	0	1	0	1
CD_67	0	0	0	104	1	0	0	0	0
CD_68	0	0	1	7	0	0	0	0	1
CD_78	0	0	1	6	0	0	0	0	0
CD_79	0	0	0	2	0	0	0	0	0
CD_80	1	0	2	17	1	0	0	0	0
CDFG_53	0	0	1	24	1	0	0	0	0
CDFG_54	0	0	7	2	34	0	3	0	0
CDFG_55	0	0	6	8	9	0	1	0	0
CDFG_56	0	1	0	0	0	0	0	0	0
CDFG_69	0	0	0	6	1	0	0	0	0
CDFG_70	0	0	2	0	0	0	0	0	0
CDFG_71	0	3	0	7	4	0	0	0	0
HF_41	0	0	0	2	0	0	0	0	0
HF_42	7	1	12	8	0	1	0	0	2
HF_43	0	0	0	0	0	4	7	0	4
HF_44	0	0	1	4	0	0	0	0	0
HF_57	0	0	0	9	0	0	2	0	0
HF_58	0	0	0	12	0	0	18	1	0
HF_59	0	0	0	0	0	1	1	1	0
HF_60	0	0	1	0	0	0	0	0	0
HF_72	0	0	1	2	1	0	0	0	0

Kingdom	Bacteria	Bacteria	Bacteria	Bacteria	Bacteria	Bacteria	Bacteria	Bacteria	Bacteria
Phylum	Firmicutes	Firmicutes	Firmicutes	Firmicutes	Firmicutes	Firmicutes	Firmicutes	Firmicutes	Firmicutes
Class	Clostridia	Clostridia	Clostridia	Bacilli	Clostridia	Clostridia	Clostridia	Clostridia	Clostridia
Order	Clostridiales	Clostridiales	Clostridiales	Bacillales	Clostridiales	Clostridiales	Clostridiales	Clostridiales	Clostridiales
Family	Lachnospiraceae	Peptostreptococcaceae	Lachnospiraceae	Staphylococcaceae	Lachnospiraceae	Lachnospiraceae	Lachnospiraceae	Lachnospiraceae	Lachnospiraceae
Genus	Marvinbryantia	Filifactor	Acetatifactor	Staphylococcus	Clostridium_XlVa	Clostridium_XlVa	Butyrivibrio	Clostridium_XlVa	Clostridium_XlVb
#NAME	ZOTU_0250	ZOTU_0251	ZOTU_0252	ZOTU_0253	ZOTU_0254	ZOTU_0255	ZOTU_0256	ZOTU_0257	ZOTU_0258
HF_73	0	0	0	30	1	0	0	0	0
HF_74	0	0	1	0	2	0	2	0	2
HFFG_45	2	0	0	15	0	0	0	0	2
HFFG_46	0	0	0	17	0	0	0	0	0
HFFG_47	0	0	0	5	0	0	0	0	0
HFFG_48	0	2	0	0	0	0	0	0	1
HFFG_61	0	0	0	0	0	0	0	0	0
HFFG_62	0	0	0	14	0	0	0	0	0
HFFG_63	1	0	0	1	0	0	0	0	0
HFFG_64	2	0	0	6	2	0	0	0	0
HFFG_75	0	0	0	1	1	0	0	0	1
HFFG_76	0	0	1	65	2	1	0	0	3
HFFG_77	0	0	0	18	0	0	0	0	0

Kingdom	Bacteria	Bacteria	Bacteria	Bacteria	Bacteria	Bacteria	Bacteria	Bacteria	Bacteria
Phylum	Firmicutes	Firmicutes	Tenericutes	Firmicutes	Tenericutes	Firmicutes	Firmicutes	Tenericutes	Firmicutes
Class	Clostridia	Clostridia	Mollicutes	Clostridia	Mollicutes	Clostridia	Clostridia	Mollicutes	Clostridia
Order	Clostridiales	Clostridiales	Acholeplasmatales	Clostridiales	Acholeplasmatales	Clostridiales	Clostridiales	Entomoplasmatales	Clostridiales
Family	Lachnospiraceae	Lachnospiraceae	Acholeplasmataceae	Lachnospiraceae	Acholeplasmataceae	Clostridiaceae_	Lachnospiraceae	Spiroplasmataceae	Ruminococcaceae
Genus	Clostridium_XlVa	Clostridium_XlVa	Acholeplasma	Clostridium_XlVa	Acholeplasma	Geosporobacter	Clostridium_XlVa	Spiroplasma	Butyricicoccus
#NAME	ZOTU_0259	ZOTU_0260	ZOTU_0261	ZOTU_0262	ZOTU_0263	ZOTU_0264	ZOTU_0265	ZOTU_0266	ZOTU_0267
CD_49	0	0	0	0	0	0	4	0	0
CD_50	0	0	0	0	0	0	5	0	0
CD_51	1	0	0	0	25	0	4	0	3
CD_52	0	0	0	0	0	0	1	0	0
CD_65	0	0	0	0	0	0	1	0	0
CD_66	1	0	0	0	0	0	0	0	2
CD_67	0	0	0	0	0	0	1	0	0
CD_68	0	0	0	0	0	0	0	0	0
CD_78	0	0	0	0	16	0	0	0	0
CD_79	0	0	1	0	0	0	0	0	0
CD_80	0	0	0	0	3	0	2	0	0
CDFG_53	0	0	0	0	0	0	0	0	0
CDFG_54	0	0	0	0	4	0	0	0	0
CDFG_55	1	0	0	0	0	0	0	0	0
CDFG_56	0	0	0	0	0	0	0	0	0
CDFG_69	0	0	2	0	3	0	0	0	0
CDFG_70	1	0	0	0	3	0	0	0	1
CDFG_71	0	0	5	0	2	0	0	0	0
HF_41	1	0	0	0	0	0	0	4	0
HF_42	76	0	0	0	0	0	1	98	0
HF_43	4	0	0	0	0	0	0	0	0
HF_44	7	0	0	0	0	0	0	1	0
HF_57	0	0	0	0	0	0	0	0	0
HF_58	0	0	0	0	0	0	3	0	1
HF_59	0	0	0	0	0	0	6	0	0
HF_60	0	0	0	0	0	0	1	0	0
HF_72	0	0	0	0	0	0	0	0	0

Kingdom	Bacteria	Bacteria	Bacteria	Bacteria	Bacteria	Bacteria	Bacteria	Bacteria	Bacteria
Phylum	Firmicutes	Firmicutes	Tenericutes	Firmicutes	Tenericutes	Firmicutes	Firmicutes	Tenericutes	Firmicutes
Class	Clostridia	Clostridia	Mollicutes	Clostridia	Mollicutes	Clostridia	Clostridia	Mollicutes	Clostridia
Order	Clostridiales	Clostridiales	Acholeplasmatales	Clostridiales	Acholeplasmatales	Clostridiales	Clostridiales	Entomoplasmatales	Clostridiales
Family	Lachnospiraceae	Lachnospiraceae	Acholeplasmataceae	Lachnospiraceae	Acholeplasmataceae	Clostridiaceae_	Lachnospiraceae	Spiroplasmataceae	Ruminococcaceae
Genus	Clostridium_XlVa	Clostridium_XlVa	Acholeplasma	Clostridium_XlVa	Acholeplasma	Geosporobacter	Clostridium_XlVa	Spiroplasma	Butyricicoccus
#NAME	ZOTU_0259	ZOTU_0260	ZOTU_0261	ZOTU_0262	ZOTU_0263	ZOTU_0264	ZOTU_0265	ZOTU_0266	ZOTU_0267
HF_73	0	0	0	0	0	0	0	0	0
HF_74	0	0	0	17	0	0	1	0	0
HFFG_45	1	0	0	1	0	0	0	0	0
HFFG_46	0	0	0	0	0	0	0	0	3
HFFG_47	0	0	0	0	0	0	0	0	0
HFFG_48	0	0	0	0	0	0	0	0	1
HFFG_61	0	0	0	0	0	0	0	0	0
HFFG_62	1	0	0	0	0	0	0	0	0
HFFG_63	0	0	0	0	0	0	0	0	0
HFFG_64	0	0	0	0	0	0	0	0	7
HFFG_75	0	0	6	4	0	0	0	0	2
HFFG_76	0	0	7	11	0	0	0	0	1
HFFG_77	0	0	0	2	4	0	0	0	0

Kingdom	Bacteria	Bacteria	Bacteria	Bacteria	Bacteria	Bacteria	Bacteria	Bacteria	Bacteria
Phylum	Firmicutes	Firmicutes	Firmicutes	Firmicutes	Firmicutes	Firmicutes	Firmicutes	Firmicutes	Firmicutes
Class	Clostridia	Clostridia	Clostridia	Clostridia	Clostridia	Clostridia	Clostridia	Erysipelotrichia	Clostridia
Order	Clostridiales	Clostridiales	Clostridiales	Clostridiales	Clostridiales	Clostridiales	Clostridiales	Erysipelotrichales	Clostridiales
Family	Lachnospiraceae	Lachnospiraceae	Lachnospiraceae	Lachnospiraceae	Ruminococcaceae	Lachnospiraceae	Lachnospiraceae	Erysipelotrichaceae	Clostridiaceae_
Genus	Roseburia	Blautia	Clostridium_XlVa	Clostridium_XlVa	Clostridium_IV	Clostridium_XlVa	Clostridium_XlVa	Holdemania	Geosporobacter
#NAME	ZOTU_0268	ZOTU_0269	ZOTU_0270	ZOTU_0271	ZOTU_0272	ZOTU_0273	ZOTU_0274	ZOTU_0275	ZOTU_0276
CD_49	0	0	0	0	4	1	0	0	0
CD_50	0	0	1	0	0	0	1	0	0
CD_51	0	0	1	0	6	1	0	0	1
CD_52	0	0	3	0	2	0	2	0	0
CD_65	0	0	2	0	1	0	0	0	0
CD_66	0	0	0	0	0	0	0	0	0
CD_67	0	0	0	0	0	0	0	0	0
CD_68	0	0	0	0	1	1	0	0	0
CD_78	0	0	0	1	0	0	1	0	0
CD_79	0	0	0	0	1	0	0	0	0
CD_80	0	0	0	0	0	0	1	0	0
CDFG_53	0	0	0	0	0	0	0	0	0
CDFG_54	0	0	1	0	3	12	9	29	0
CDFG_55	0	0	0	0	2	4	0	16	0
CDFG_56	0	0	0	0	0	0	1	3	1
CDFG_69	0	0	0	0	1	1	0	0	0
CDFG_70	0	0	1	0	0	5	0	0	0
CDFG_71	0	0	1	0	0	1	0	5	11
HF_41	0	0	0	0	1	0	0	0	0
HF_42	1	0	0	0	6	2	0	0	0
HF_43	0	0	2	0	0	0	0	0	0
HF_44	0	0	0	0	0	0	0	0	0
HF_57	0	0	0	0	0	0	0	0	0
HF_58	0	0	1	1	2	0	0	0	0
HF_59	1	0	0	0	1	0	1	0	0
HF_60	0	0	1	0	1	0	0	1	0
HF_72	0	0	0	0	0	0	0	0	0

Kingdom	Bacteria	Bacteria	Bacteria	Bacteria	Bacteria	Bacteria	Bacteria	Bacteria	Bacteria
Phylum	Firmicutes	Firmicutes	Firmicutes	Firmicutes	Firmicutes	Firmicutes	Firmicutes	Firmicutes	Firmicutes
Class	Clostridia	Clostridia	Clostridia	Clostridia	Clostridia	Clostridia	Clostridia	Erysipelotrichia	Clostridia
Order	Clostridiales	Clostridiales	Clostridiales	Clostridiales	Clostridiales	Clostridiales	Clostridiales	Erysipelotrichales	Clostridiales
Family	Lachnospiraceae	Lachnospiraceae	Lachnospiraceae	Lachnospiraceae	Ruminococcaceae	Lachnospiraceae	Lachnospiraceae	Erysipelotrichaceae	Clostridiaceae_
Genus	Roseburia	Blautia	Clostridium_XlVa	Clostridium_XlVa	Clostridium_IV	Clostridium_XlVa	Clostridium_XlVa	Holdemania	Geosporobacter
#NAME	ZOTU_0268	ZOTU_0269	ZOTU_0270	ZOTU_0271	ZOTU_0272	ZOTU_0273	ZOTU_0274	ZOTU_0275	ZOTU_0276
HF_73	0	0	0	0	0	1	0	0	0
HF_74	0	0	0	0	2	0	1	0	0
HFFG_45	0	0	0	0	1	0	0	0	0
HFFG_46	0	0	1	0	2	0	0	1	0
HFFG_47	0	0	0	0	0	0	0	0	0
HFFG_48	0	0	0	0	0	0	0	0	0
HFFG_61	1	0	0	0	0	0	0	0	3
HFFG_62	0	0	0	0	1	0	0	0	0
HFFG_63	0	0	0	0	1	0	0	0	3
HFFG_64	4	0	0	0	0	0	0	0	1
HFFG_75	1	0	0	0	3	0	0	0	0
HFFG_76	0	0	0	0	0	0	0	0	0
HFFG_77	0	0	0	0	0	0	0	0	0

Kingdom	Bacteria	Bacteria	Bacteria	Bacteria	Bacteria	Bacteria	Bacteria
Phylum	Firmicutes	Cyanobacteria_Chloroplast	Firmicutes	Firmicutes	Firmicutes	Firmicutes	Firmicutes
Class	Clostridia	Chloroplast	Clostridia	Clostridia	Clostridia	Clostridia	Clostridia
Order	Clostridiales	Chloroplast	Clostridiales	Clostridiales	Clostridiales	Clostridiales	Clostridiales
Family	Ruminococcaceae	Streptophyta	Lachnospiraceae	Lachnospiraceae	Clostridiaceae_	Lachnospiraceae	Lachnospiraceae
Genus	Oscillibacter	NA	Clostridium_XlVa	Lachnospiracea_incertae_sedis	Clostridium_sensu_stricto	Clostridium_XlVa	Lachnospiracea_incertae_sedis
#NAME	ZOTU_0277	ZOTU_0278	ZOTU_0279	ZOTU_0280	ZOTU_0281	ZOTU_0282	ZOTU_0283
CD_49	2	0	0	0	0	0	1
CD_50	0	0	0	0	0	0	0
CD_51	15	0	0	0	20	1	5
CD_52	0	0	0	0	2	0	6
CD_65	0	0	0	0	0	0	0
CD_66	0	0	0	0	3	0	2
CD_67	4	0	0	0	9	0	0
CD_68	1	0	0	0	6	0	0
CD_78	0	0	0	0	4	0	0
CD_79	0	0	0	0	0	0	1
CD_80	0	0	0	0	0	1	0
CDFG_53	0	0	0	0	2	0	0
CDFG_54	0	1	0	0	5	1	0
CDFG_55	0	17	0	0	1	0	1
CDFG_56	0	0	0	0	1	0	0
CDFG_69	0	1	0	0	0	0	0
CDFG_70	0	0	0	0	1	0	0
CDFG_71	0	0	0	0	0	0	5
HF_41	0	0	0	0	0	1	1
HF_42	3	0	0	0	0	6	0
HF_43	0	0	0	0	0	0	5
HF_44	0	0	0	0	0	1	0
HF_57	0	0	0	0	0	0	0
HF_58	0	0	0	0	0	2	0
HF_59	0	0	0	0	0	1	0
HF_60	0	0	0	0	0	3	0
HF_72	3	0	0	0	0	2	0

Kingdom	Bacteria	Bacteria	Bacteria	Bacteria	Bacteria	Bacteria	Bacteria
Phylum	Firmicutes	Cyanobacteria_Chloroplast	Firmicutes	Firmicutes	Firmicutes	Firmicutes	Firmicutes
Class	Clostridia	Chloroplast	Clostridia	Clostridia	Clostridia	Clostridia	Clostridia
Order	Clostridiales	Chloroplast	Clostridiales	Clostridiales	Clostridiales	Clostridiales	Clostridiales
Family	Ruminococcaceae	Streptophyta	Lachnospiraceae	Lachnospiraceae	Clostridiaceae_	Lachnospiraceae	Lachnospiraceae
Genus	Oscillibacter	NA	Clostridium_XlVa	Lachnospiracea_incertae_sedis	Clostridium_sensu_stricto	Clostridium_XlVa	Lachnospiracea_incertae_sedis
#NAME	ZOTU_0277	ZOTU_0278	ZOTU_0279	ZOTU_0280	ZOTU_0281	ZOTU_0282	ZOTU_0283
HF_73	0	0	0	0	0	0	0
HF_74	0	0	0	0	0	0	1
HFFG_45	0	0	0	0	4	0	0
HFFG_46	1	0	0	0	0	0	1
HFFG_47	0	0	0	0	0	0	0
HFFG_48	0	0	0	0	0	0	1
HFFG_61	0	0	0	0	4	1	0
HFFG_62	0	2	0	0	3	0	0
HFFG_63	0	2	0	0	1	0	0
HFFG_64	0	0	0	0	1	0	0
HFFG_75	0	2	0	0	16	0	0
HFFG_76	1	1	0	0	8	0	0
HFFG_77	0	0	0	0	0	0	0

Kingdom	Bacteria	Bacteria	Bacteria	Bacteria	Bacteria	Bacteria	Bacteria	Bacteria	Bacteria
Phylum	Tenericutes	Firmicutes	Firmicutes	Firmicutes	Firmicutes	Firmicutes	Firmicutes	Firmicutes	Firmicutes
Class	Mollicutes	Clostridia	Clostridia	Clostridia	Clostridia	Clostridia	Clostridia	Clostridia	Bacilli
Order	Entomoplasmatales	Clostridiales	Clostridiales	Clostridiales	Clostridiales	Clostridiales	Clostridiales	Halanaerobiales	Bacillales
Family	Spiroplasmataceae	Clostridiaceae_	Lachnospiraceae	Lachnospiraceae	Ruminococcaceae	Lachnospiraceae	Lachnospiraceae	Halanaerobiaceae	Bacillaceae_
Genus	Spiroplasma	Alkaliphilus	Clostridium_XlVa	Clostridium_XlVa	Intestinimonas	Clostridium_XlVa	Clostridium_XlVa	Halanaerobium	Bacillus
#NAME	ZOTU_0284	ZOTU_0285	ZOTU_0286	ZOTU_0287	ZOTU_0288	ZOTU_0289	ZOTU_0290	ZOTU_0291	ZOTU_0292
CD_49	0	0	0	0	1	0	0	0	0
CD_50	0	0	1	0	0	0	2	0	0
CD_51	2	0	1	0	7	0	0	0	3
CD_52	0	0	1	0	0	1	0	0	0
CD_65	0	0	1	0	0	0	0	0	0
CD_66	0	0	0	0	0	0	0	0	0
CD_67	0	0	1	0	0	0	0	0	0
CD_68	0	0	1	0	0	0	1	0	0
CD_78	1	0	0	0	0	0	0	0	0
CD_79	0	0	0	0	0	0	0	0	0
CD_80	1	0	0	0	0	0	0	0	0
CDFG_53	0	0	0	0	0	0	0	0	0
CDFG_54	0	0	0	1	4	0	4	0	0
CDFG_55	0	0	0	0	2	0	1	0	0
CDFG_56	0	0	0	1	0	0	0	0	0
CDFG_69	10	0	0	0	0	0	0	0	0
CDFG_70	0	32	1	2	0	0	0	0	0
CDFG_71	6	0	0	0	0	1	0	0	0
HF_41	1	0	0	0	0	1	1	0	0
HF_42	10	0	0	0	0	48	7	0	0
HF_43	2	0	0	3	0	0	0	0	0
HF_44	0	0	0	0	0	0	0	0	0
HF_57	0	0	0	2	0	0	0	0	0
HF_58	0	0	2	0	0	0	0	0	0
HF_59	0	0	1	0	0	0	0	0	0
HF_60	0	0	1	0	0	0	0	0	0
HF_72	0	0	0	0	0	2	0	0	1

Kingdom	Bacteria	Bacteria	Bacteria	Bacteria	Bacteria	Bacteria	Bacteria	Bacteria	Bacteria
Phylum	Tenericutes	Firmicutes	Firmicutes	Firmicutes	Firmicutes	Firmicutes	Firmicutes	Firmicutes	Firmicutes
Class	Mollicutes	Clostridia	Clostridia	Clostridia	Clostridia	Clostridia	Clostridia	Clostridia	Bacilli
Order	Entomoplasmatales	Clostridiales	Clostridiales	Clostridiales	Clostridiales	Clostridiales	Clostridiales	Halanaerobiales	Bacillales
Family	Spiroplasmataceae	Clostridiaceae_	Lachnospiraceae	Lachnospiraceae	Ruminococcaceae	Lachnospiraceae	Lachnospiraceae	Halanaerobiaceae	Bacillaceae_
Genus	Spiroplasma	Alkaliphilus	Clostridium_XlVa	Clostridium_XlVa	Intestinimonas	Clostridium_XlVa	Clostridium_XlVa	Halanaerobium	Bacillus
#NAME	ZOTU_0284	ZOTU_0285	ZOTU_0286	ZOTU_0287	ZOTU_0288	ZOTU_0289	ZOTU_0290	ZOTU_0291	ZOTU_0292
HF_73	0	0	0	0	0	0	0	0	0
HF_74	0	0	0	3	0	0	1	0	0
HFFG_45	0	0	0	1	0	0	0	0	0
HFFG_46	0	0	0	0	1	0	0	0	0
HFFG_47	0	0	0	0	0	0	0	0	0
HFFG_48	0	0	0	1	0	0	0	0	0
HFFG_61	0	0	0	0	1	0	0	0	0
HFFG_62	0	0	0	0	0	0	0	0	0
HFFG_63	0	0	0	0	0	0	0	0	0
HFFG_64	0	0	0	0	0	0	0	0	0
HFFG_75	0	0	0	0	1	0	0	0	0
HFFG_76	3	0	1	0	2	0	2	0	0
HFFG_77	0	0	0	2	0	0	0	0	0

Kingdom	Bacteria	Bacteria	Bacteria	Bacteria	Bacteria	Bacteria	Bacteria	Bacteria	Bacteria
Phylum	Firmicutes	Firmicutes	Proteobacteria	Firmicutes	Firmicutes	Actinobacteria	Firmicutes	Firmicutes	Firmicutes
Class	Bacilli	Clostridia	Gammaproteobacteria	Clostridia	Clostridia	Actinobacteria	Clostridia	Clostridia	Clostridia
Order	Lactobacillales	Clostridiales	Pasteurellales	Clostridiales	Clostridiales	Actinomycetales	Clostridiales	Clostridiales	Clostridiales
Family	Streptococcaceae	Ruminococcaceae	Pasteurellaceae	Ruminococcaceae	Lachnospiraceae	Streptomycetaceae	Lachnospiraceae	Lachnospiraceae	Lachnospiraceae
Genus	Streptococcus	Clostridium_IV	Haemophilus	Sporobacter	Clostridium_XlVa	Streptomyces	Clostridium_XlVa	Blautia	Clostridium_XlVa
#NAME	ZOTU_0293	ZOTU_0294	ZOTU_0295	ZOTU_0296	ZOTU_0297	ZOTU_0298	ZOTU_0299	ZOTU_0300	ZOTU_0301
CD_49	0	0	0	2	0	0	0	0	0
CD_50	0	0	0	0	0	0	0	0	0
CD_51	0	0	0	0	0	0	0	0	0
CD_52	0	0	0	0	0	0	0	0	0
CD_65	0	0	0	0	0	0	0	0	0
CD_66	0	0	1	0	0	0	0	0	0
CD_67	0	0	0	0	0	0	0	0	0
CD_68	0	0	0	0	0	0	0	0	0
CD_78	0	0	0	0	0	0	0	0	0
CD_79	0	0	0	0	0	0	0	0	0
CD_80	0	0	0	0	0	0	0	0	0
CDFG_53	0	0	0	0	0	0	0	0	0
CDFG_54	0	0	0	0	0	0	0	0	0
CDFG_55	0	1	2	0	0	0	0	0	0
CDFG_56	0	0	0	0	0	0	0	0	0
CDFG_69	0	0	0	0	0	0	0	0	0
CDFG_70	0	0	0	0	0	0	0	0	0
CDFG_71	0	0	0	0	0	0	0	0	0
HF_41	0	0	0	0	0	0	0	1	0
HF_42	0	0	0	1	0	0	0	2	0
HF_43	0	0	1	0	0	0	0	0	0
HF_44	0	0	0	0	0	0	0	0	0
HF_57	0	0	0	0	0	2	0	0	0
HF_58	0	0	0	1	5	6	0	0	0
HF_59	0	1	1	1	1	2	0	3	0
HF_60	2	1	0	0	0	0	0	0	0
HF_72	0	0	0	0	0	0	0	0	0

Kingdom	Bacteria	Bacteria	Bacteria	Bacteria	Bacteria	Bacteria	Bacteria	Bacteria	Bacteria
Phylum	Firmicutes	Firmicutes	Proteobacteria	Firmicutes	Firmicutes	Actinobacteria	Firmicutes	Firmicutes	Firmicutes
Class	Bacilli	Clostridia	Gammaproteobacteria	Clostridia	Clostridia	Actinobacteria	Clostridia	Clostridia	Clostridia
Order	Lactobacillales	Clostridiales	Pasteurellales	Clostridiales	Clostridiales	Actinomycetales	Clostridiales	Clostridiales	Clostridiales
Family	Streptococcaceae	Ruminococcaceae	Pasteurellaceae	Ruminococcaceae	Lachnospiraceae	Streptomycetaceae	Lachnospiraceae	Lachnospiraceae	Lachnospiraceae
Genus	Streptococcus	Clostridium_IV	Haemophilus	Sporobacter	Clostridium_XlVa	Streptomyces	Clostridium_XlVa	Blautia	Clostridium_XlVa
#NAME	ZOTU_0293	ZOTU_0294	ZOTU_0295	ZOTU_0296	ZOTU_0297	ZOTU_0298	ZOTU_0299	ZOTU_0300	ZOTU_0301
HF_73	0	0	0	0	0	0	0	0	0
HF_74	0	2	0	0	0	0	0	0	0
HFFG_45	0	0	0	0	0	0	0	0	0
HFFG_46	0	1	0	0	0	0	0	0	0
HFFG_47	0	0	0	0	0	0	0	0	0
HFFG_48	0	0	0	0	0	0	0	0	0
HFFG_61	0	0	0	0	0	0	0	0	0
HFFG_62	0	0	0	0	0	0	0	0	0
HFFG_63	0	0	0	0	0	0	0	0	0
HFFG_64	0	5	0	0	0	1	0	0	0
HFFG_75	0	0	0	0	0	0	0	0	0
HFFG_76	0	0	0	0	0	0	0	0	0
HFFG_77	0	0	0	0	0	0	0	0	0

Kingdom	Bacteria	Bacteria	Bacteria	Bacteria	Bacteria	Bacteria	Bacteria	Bacteria	Bacteria
Phylum	Firmicutes	Firmicutes	Firmicutes	Firmicutes	Tenericutes	Firmicutes	Firmicutes	Firmicutes	Firmicutes
Class	Clostridia	Bacilli	Bacilli	Clostridia	Mollicutes	Clostridia	Clostridia	Clostridia	Clostridia
Order	Clostridiales	Bacillales	Lactobacillales	Clostridiales	Acholeplasmatales	Clostridiales	Clostridiales	Clostridiales	Clostridiales
Family	Lachnospiraceae	Bacillaceae_	Enterococcaceae	Lachnospiraceae	Acholeplasmataceae	Ruminococcaceae	Lachnospiraceae	Ruminococcaceae	Ruminococcaceae
Genus	Acetatifactor	Bacillus	Enterococcus	Clostridium_XlVa	Acholeplasma	Anaerotruncus	Acetatifactor	Flavonifractor	Ethanoligenens
#NAME	ZOTU_0302	ZOTU_0303	ZOTU_0304	ZOTU_0305	ZOTU_0306	ZOTU_0307	ZOTU_0308	ZOTU_0309	ZOTU_0310
CD_49	0	0	20	0	0	0	0	0	0
CD_50	0	0	8	0	0	1	0	2	0
CD_51	0	0	8	1	0	4	0	2	0
CD_52	0	0	46	0	0	0	0	0	1
CD_65	0	0	2	0	0	0	0	3	0
CD_66	0	0	2	0	0	0	0	0	0
CD_67	0	0	6	0	0	0	0	0	0
CD_68	0	0	2	0	0	2	0	1	0
CD_78	0	0	0	0	0	0	0	0	0
CD_79	0	0	0	0	0	0	0	0	1
CD_80	0	0	1	0	0	0	0	0	0
CDFG_53	0	0	0	0	0	0	0	0	0
CDFG_54	0	0	0	1	0	0	1	0	1
CDFG_55	0	0	1	2	0	1	2	1	1
CDFG_56	0	0	0	1	0	0	0	1	1
CDFG_69	0	0	0	0	0	1	0	0	0
CDFG_70	0	0	0	0	0	0	0	0	0
CDFG_71	0	0	3	0	0	1	0	0	0
HF_41	0	0	19	0	0	0	0	1	0
HF_42	0	0	2	0	51	3	0	10	0
HF_43	2	0	298	0	0	0	0	0	2
HF_44	0	0	155	0	0	0	0	0	1
HF_57	0	0	0	0	0	0	0	0	0
HF_58	0	0	15	0	0	2	2	1	0
HF_59	0	0	1	0	0	0	0	0	0
HF_60	0	0	1	0	0	0	0	0	0
HF_72	0	0	50	0	0	0	0	0	0

Kingdom	Bacteria	Bacteria	Bacteria	Bacteria	Bacteria	Bacteria	Bacteria	Bacteria	Bacteria
Phylum	Firmicutes	Firmicutes	Firmicutes	Firmicutes	Tenericutes	Firmicutes	Firmicutes	Firmicutes	Firmicutes
Class	Clostridia	Bacilli	Bacilli	Clostridia	Mollicutes	Clostridia	Clostridia	Clostridia	Clostridia
Order	Clostridiales	Bacillales	Lactobacillales	Clostridiales	Acholeplasmatales	Clostridiales	Clostridiales	Clostridiales	Clostridiales
Family	Lachnospiraceae	Bacillaceae_	Enterococcaceae	Lachnospiraceae	Acholeplasmataceae	Ruminococcaceae	Lachnospiraceae	Ruminococcaceae	Ruminococcaceae
Genus	Acetatifactor	Bacillus	Enterococcus	Clostridium_XlVa	Acholeplasma	Anaerotruncus	Acetatifactor	Flavonifractor	Ethanoligenens
#NAME	ZOTU_0302	ZOTU_0303	ZOTU_0304	ZOTU_0305	ZOTU_0306	ZOTU_0307	ZOTU_0308	ZOTU_0309	ZOTU_0310
HF_73	0	0	93	0	0	0	0	0	0
HF_74	0	0	26	0	0	0	0	0	0
HFFG_45	0	0	8	0	0	1	0	0	0
HFFG_46	0	0	0	0	0	0	0	0	1
HFFG_47	0	0	2	0	0	0	0	0	0
HFFG_48	0	0	1	0	0	0	0	0	0
HFFG_61	0	0	8	0	0	0	1	0	0
HFFG_62	0	0	21	0	0	2	0	0	0
HFFG_63	0	0	15	0	0	0	0	0	0
HFFG_64	0	0	19	0	0	2	0	0	0
HFFG_75	0	0	58	0	0	5	0	0	0
HFFG_76	0	0	37	0	0	1	0	0	0
HFFG_77	0	0	34	0	0	0	0	0	0

Kingdom	Bacteria	Bacteria	Bacteria	Bacteria	Bacteria	Bacteria	Bacteria	Bacteria
Phylum	Firmicutes	Firmicutes	Proteobacteria	Firmicutes	Firmicutes	Firmicutes	Firmicutes	Firmicutes
Class	Clostridia	Erysipelotrichia	Betaproteobacteria	Clostridia	Clostridia	Clostridia	Clostridia	Clostridia
Order	Clostridiales	Erysipelotrichales	Neisseriales	Clostridiales	Clostridiales	Clostridiales	Clostridiales	Clostridiales
Family	Lachnospiraceae	Erysipelotrichaceae	Neisseriaceae	Lachnospiraceae	Lachnospiraceae	Clostridiaceae_	Lachnospiraceae	Lachnospiraceae
Genus	Butyrivibrio	Holdemania	Neisseria	Ruminococcus	Lachnospiracea_incertae_sedis	Alkaliphilus	Clostridium_XlVa	Butyrivibrio
#NAME	ZOTU_0311	ZOTU_0312	ZOTU_0313	ZOTU_0314	ZOTU_0315	ZOTU_0316	ZOTU_0317	ZOTU_0318
CD_49	0	0	0	0	0	0	0	0
CD_50	0	0	0	0	0	0	0	0
CD_51	0	0	0	0	0	0	0	0
CD_52	0	0	1	0	0	0	0	0
CD_65	0	0	0	0	0	0	0	0
CD_66	0	0	0	0	0	0	0	0
CD_67	0	0	0	0	0	0	0	0
CD_68	0	0	0	0	0	0	0	0
CD_78	0	0	0	0	0	0	0	0
CD_79	0	0	0	0	0	0	0	0
CD_80	0	0	0	0	0	0	0	0
CDFG_53	0	0	0	0	0	0	0	0
CDFG_54	0	0	2	2	0	0	1	0
CDFG_55	1	0	0	0	0	0	0	0
CDFG_56	0	0	0	0	0	0	0	0
CDFG_69	0	0	0	0	0	0	0	0
CDFG_70	0	0	0	0	0	0	1	0
CDFG_71	0	0	0	0	0	0	0	0
HF_41	0	0	0	1	0	0	0	0
HF_42	0	0	0	0	1	0	5	0
HF_43	0	2	0	0	0	0	3	0
HF_44	0	0	0	0	0	0	1	1
HF_57	0	0	1	0	0	0	0	0
HF_58	0	0	0	0	0	0	0	0
HF_59	0	4	0	0	0	0	0	0
HF_60	0	8	1	0	0	0	0	0
HF_72	0	1	0	0	0	0	0	0

Kingdom	Bacteria	Bacteria	Bacteria	Bacteria	Bacteria	Bacteria	Bacteria	Bacteria
Phylum	Firmicutes	Firmicutes	Proteobacteria	Firmicutes	Firmicutes	Firmicutes	Firmicutes	Firmicutes
Class	Clostridia	Erysipelotrichia	Betaproteobacteria	Clostridia	Clostridia	Clostridia	Clostridia	Clostridia
Order	Clostridiales	Erysipelotrichales	Neisseriales	Clostridiales	Clostridiales	Clostridiales	Clostridiales	Clostridiales
Family	Lachnospiraceae	Erysipelotrichaceae	Neisseriaceae	Lachnospiraceae	Lachnospiraceae	Clostridiaceae_	Lachnospiraceae	Lachnospiraceae
Genus	Butyrivibrio	Holdemania	Neisseria	Ruminococcus	Lachnospiracea_incertae_sedis	Alkaliphilus	Clostridium_XlVa	Butyrivibrio
#NAME	ZOTU_0311	ZOTU_0312	ZOTU_0313	ZOTU_0314	ZOTU_0315	ZOTU_0316	ZOTU_0317	ZOTU_0318
HF_73	0	0	0	0	0	0	0	0
HF_74	0	2	0	0	0	0	0	0
HFFG_45	0	0	0	0	0	0	0	0
HFFG_46	0	0	0	0	0	0	0	0
HFFG_47	0	0	0	0	0	0	0	0
HFFG_48	0	0	0	0	0	0	0	0
HFFG_61	0	0	0	0	0	0	0	0
HFFG_62	0	0	0	0	0	0	0	0
HFFG_63	0	0	0	0	0	0	0	0
HFFG_64	0	0	0	0	0	0	0	0
HFFG_75	0	0	0	0	0	0	0	0
HFFG_76	0	0	0	0	0	0	0	0
HFFG_77	0	0	0	0	0	0	0	0

Kingdom	Bacteria	Bacteria	Bacteria	Bacteria	Bacteria	Bacteria	Bacteria	Bacteria
Phylum	Firmicutes	Firmicutes	Firmicutes	Firmicutes	Firmicutes	Bacteroidetes	Firmicutes	Firmicutes
Class	Clostridia	Clostridia	Clostridia	Clostridia	Clostridia	Bacteroidia	Clostridia	Clostridia
Order	Clostridiales	Clostridiales	Clostridiales	Clostridiales	Clostridiales	Bacteroidales	Clostridiales	Clostridiales
Family	Clostridiaceae_	Lachnospiraceae	Lachnospiraceae	Clostridiaceae_	Lachnospiraceae	Porphyromonadaceae	Lachnospiraceae	Gracilibacteraceae
Genus	Clostridium_sensu_stricto	Butyrivibrio	Clostridium_XlVa	Clostridium_sensu_stricto	Clostridium_XlVa	Parabacteroides	Clostridium_XlVa	Lutispora
#NAME	ZOTU_0319	ZOTU_0320	ZOTU_0321	ZOTU_0322	ZOTU_0323	ZOTU_0324	ZOTU_0325	ZOTU_0326
CD_49	0	0	0	2	0	0	0	0
CD_50	0	3	0	0	0	0	0	0
CD_51	0	1	0	4	0	0	0	0
CD_52	0	0	0	0	0	0	1	0
CD_65	0	0	0	0	0	0	1	0
CD_66	1	0	0	1	0	0	0	0
CD_67	2	0	0	1	0	0	0	0
CD_68	0	0	0	2	0	0	0	0
CD_78	0	0	0	0	0	0	0	0
CD_79	0	0	0	0	0	0	0	0
CD_80	0	0	0	0	0	0	0	0
CDFG_53	0	0	0	0	0	0	0	0
CDFG_54	4	0	0	0	0	0	0	0
CDFG_55	3	1	0	0	0	0	0	0
CDFG_56	0	0	0	1	0	0	0	1
CDFG_69	0	0	0	0	0	0	0	0
CDFG_70	1	0	0	0	0	0	0	0
CDFG_71	0	0	0	0	0	0	0	0
HF_41	2	0	1	0	0	0	0	0
HF_42	0	0	3	0	0	0	0	0
HF_43	0	1	1	0	0	0	1	0
HF_44	0	0	0	5	0	0	1	0
HF_57	0	1	0	0	0	0	0	0
HF_58	0	4	0	4	0	0	0	0
HF_59	0	1	0	0	0	0	0	0
HF_60	0	1	0	0	0	0	0	0
HF_72	0	0	0	0	0	0	0	0

Kingdom	Bacteria	Bacteria	Bacteria	Bacteria	Bacteria	Bacteria	Bacteria	Bacteria
Phylum	Firmicutes	Firmicutes	Firmicutes	Firmicutes	Firmicutes	Bacteroidetes	Firmicutes	Firmicutes
Class	Clostridia	Clostridia	Clostridia	Clostridia	Clostridia	Bacteroidia	Clostridia	Clostridia
Order	Clostridiales	Clostridiales	Clostridiales	Clostridiales	Clostridiales	Bacteroidales	Clostridiales	Clostridiales
Family	Clostridiaceae_	Lachnospiraceae	Lachnospiraceae	Clostridiaceae_	Lachnospiraceae	Porphyromonadaceae	Lachnospiraceae	Gracilibacteraceae
Genus	Clostridium_sensu_stricto	Butyrivibrio	Clostridium_XlVa	Clostridium_sensu_stricto	Clostridium_XlVa	Parabacteroides	Clostridium_XlVa	Lutispora
#NAME	ZOTU_0319	ZOTU_0320	ZOTU_0321	ZOTU_0322	ZOTU_0323	ZOTU_0324	ZOTU_0325	ZOTU_0326
HF_73	0	0	0	0	0	0	0	0
HF_74	2	1	0	3	0	0	2	0
HFFG_45	1	0	0	0	0	4	0	0
HFFG_46	0	0	0	0	0	2	0	0
HFFG_47	0	0	0	0	0	1	0	0
HFFG_48	0	0	0	1	0	1	0	0
HFFG_61	0	0	0	0	0	0	0	0
HFFG_62	0	0	0	0	0	0	0	0
HFFG_63	0	0	0	0	0	0	0	0
HFFG_64	0	0	0	0	0	0	0	0
HFFG_75	0	0	0	1	0	0	0	0
HFFG_76	0	0	0	8	0	0	0	0
HFFG_77	0	0	0	0	0	0	0	0

Kingdom	Bacteria	Bacteria	Bacteria	Bacteria	Bacteria	Bacteria	Bacteria	Bacteria
Phylum	Firmicutes	Firmicutes	Firmicutes	Firmicutes	Firmicutes	Firmicutes	Firmicutes	Bacteroidetes
Class	Clostridia	Clostridia	Clostridia	Clostridia	Clostridia	Clostridia	Clostridia	Bacteroidia
Order	Clostridiales	Clostridiales	Clostridiales	Clostridiales	Clostridiales	Clostridiales	Clostridiales	Bacteroidales
Family	Lachnospiraceae	Lachnospiraceae	Peptostreptococcaceae	Ruminococcaceae	Clostridiales_Incertae_Sedis_XI	Lachnospiraceae	Ruminococcaceae	Porphyromonadaceae
Genus	Clostridium_XlVa	Ruminococcus	Clostridium_XI	Clostridium_IV	Tissierella	Acetitomaculum	Oscillibacter	Barnesiella
#NAME	ZOTU_0327	ZOTU_0328	ZOTU_0329	ZOTU_0330	ZOTU_0331	ZOTU_0332	ZOTU_0333	ZOTU_0334
CD_49	0	0	0	0	0	0	0	0
CD_50	0	0	0	1	0	0	0	0
CD_51	0	0	0	2	1	0	0	0
CD_52	0	0	0	0	0	0	0	0
CD_65	0	0	0	0	0	0	0	0
CD_66	0	0	0	0	0	0	0	0
CD_67	0	0	0	0	0	0	0	0
CD_68	0	0	0	1	0	0	0	0
CD_78	0	0	0	1	0	0	0	0
CD_79	0	0	0	0	0	0	0	0
CD_80	0	0	0	0	0	0	0	0
CDFG_53	0	0	0	0	0	0	0	0
CDFG_54	0	1	0	3	0	0	0	3
CDFG_55	0	1	0	0	0	0	0	4
CDFG_56	0	0	0	0	0	0	0	0
CDFG_69	0	0	0	0	0	0	0	0
CDFG_70	0	0	0	0	0	0	0	0
CDFG_71	0	0	0	0	0	0	0	0
HF_41	0	0	0	2	0	0	0	0
HF_42	3	0	0	0	0	0	0	8
HF_43	0	0	0	0	1	0	0	7
HF_44	1	0	0	1	0	0	0	0
HF_57	0	0	0	0	0	0	0	0
HF_58	0	0	0	1	0	0	0	0
HF_59	0	0	0	2	0	0	0	0
HF_60	0	0	0	0	0	0	0	0
HF_72	0	0	0	1	0	0	0	0

Kingdom	Bacteria	Bacteria	Bacteria	Bacteria	Bacteria	Bacteria	Bacteria	Bacteria
Phylum	Firmicutes	Firmicutes	Firmicutes	Firmicutes	Firmicutes	Firmicutes	Firmicutes	Bacteroidetes
Class	Clostridia	Clostridia	Clostridia	Clostridia	Clostridia	Clostridia	Clostridia	Bacteroidia
Order	Clostridiales	Clostridiales	Clostridiales	Clostridiales	Clostridiales	Clostridiales	Clostridiales	Bacteroidales
Family	Lachnospiraceae	Lachnospiraceae	Peptostreptococcaceae	Ruminococcaceae	Clostridiales_Incertae_Sedis_XI	Lachnospiraceae	Ruminococcaceae	Porphyromonadaceae
Genus	Clostridium_XlVa	Ruminococcus	Clostridium_XI	Clostridium_IV	Tissierella	Acetitomaculum	Oscillibacter	Barnesiella
#NAME	ZOTU_0327	ZOTU_0328	ZOTU_0329	ZOTU_0330	ZOTU_0331	ZOTU_0332	ZOTU_0333	ZOTU_0334
HF_73	0	0	0	0	0	0	0	0
HF_74	0	0	0	1	0	0	0	0
HFFG_45	0	0	0	0	0	0	0	0
HFFG_46	0	0	0	0	0	0	0	0
HFFG_47	0	0	0	0	0	0	0	0
HFFG_48	0	0	0	0	0	0	0	0
HFFG_61	0	0	0	0	0	0	0	0
HFFG_62	0	0	0	0	0	0	0	0
HFFG_63	0	0	0	0	0	0	0	0
HFFG_64	0	0	0	1	0	0	0	0
HFFG_75	0	0	0	2	0	0	0	0
HFFG_76	0	0	0	2	0	0	0	0
HFFG_77	0	0	0	1	0	0	0	0

Kingdom	Bacteria	Bacteria	Bacteria	Bacteria	Bacteria	Bacteria	Bacteria	Bacteria
Phylum	Firmicutes	Firmicutes	Bacteroidetes	Firmicutes	Firmicutes	Firmicutes	Firmicutes	Firmicutes
Class	Clostridia	Clostridia	Bacteroidia	Clostridia	Clostridia	Clostridia	Clostridia	Clostridia
Order	Clostridiales	Clostridiales	Bacteroidales	Clostridiales	Clostridiales	Clostridiales	Clostridiales	Clostridiales
Family	Ruminococcaceae	Lachnospiraceae	Porphyromonadaceae	Lachnospiraceae	Lachnospiraceae	Lachnospiraceae	Lachnospiraceae	Ruminococcaceae
Genus	Sporobacter	Anaerosporobacter	Parabacteroides	Clostridium_XlVa	Clostridium_XlVa	Lachnospiracea_incertae_sedis	Clostridium_XlVa	Clostridium_IV
#NAME	ZOTU_0335	ZOTU_0336	ZOTU_0337	ZOTU_0338	ZOTU_0339	ZOTU_0340	ZOTU_0341	ZOTU_0342
CD_49	0	0	0	0	0	0	1	0
CD_50	0	0	0	1	0	0	0	0
CD_51	1	2	0	1	1	0	1	0
CD_52	0	0	0	0	0	0	0	0
CD_65	0	0	0	0	0	0	0	0
CD_66	0	0	0	0	0	0	0	0
CD_67	1	0	0	0	0	0	1	0
CD_68	0	0	0	0	0	0	0	0
CD_78	0	0	0	0	0	0	0	0
CD_79	0	0	0	0	0	0	0	0
CD_80	0	0	0	0	0	0	0	0
CDFG_53	0	0	0	0	0	0	0	0
CDFG_54	0	0	0	2	3	0	0	1
CDFG_55	0	0	0	1	1	0	0	0
CDFG_56	0	0	0	0	0	0	0	0
CDFG_69	0	0	0	0	0	0	0	0
CDFG_70	0	0	0	0	0	0	0	0
CDFG_71	0	0	0	0	0	0	0	0
HF_41	0	0	0	0	0	0	0	0
HF_42	1	0	0	0	1	1	0	0
HF_43	0	0	0	0	0	0	1	0
HF_44	0	0	0	0	0	0	0	0
HF_57	0	0	0	0	0	0	0	0
HF_58	0	0	0	0	0	0	0	0
HF_59	0	1	0	0	0	0	0	0
HF_60	0	0	0	0	0	0	0	0
HF_72	0	0	0	0	0	0	0	0

Kingdom	Bacteria	Bacteria	Bacteria	Bacteria	Bacteria	Bacteria	Bacteria	Bacteria
Phylum	Firmicutes	Firmicutes	Bacteroidetes	Firmicutes	Firmicutes	Firmicutes	Firmicutes	Firmicutes
Class	Clostridia	Clostridia	Bacteroidia	Clostridia	Clostridia	Clostridia	Clostridia	Clostridia
Order	Clostridiales	Clostridiales	Bacteroidales	Clostridiales	Clostridiales	Clostridiales	Clostridiales	Clostridiales
Family	Ruminococcaceae	Lachnospiraceae	Porphyromonadaceae	Lachnospiraceae	Lachnospiraceae	Lachnospiraceae	Lachnospiraceae	Ruminococcaceae
Genus	Sporobacter	Anaerosporobacter	Parabacteroides	Clostridium_XlVa	Clostridium_XlVa	Lachnospiracea_incertae_sedis	Clostridium_XlVa	Clostridium_IV
#NAME	ZOTU_0335	ZOTU_0336	ZOTU_0337	ZOTU_0338	ZOTU_0339	ZOTU_0340	ZOTU_0341	ZOTU_0342
HF_73	0	0	0	0	0	0	0	0
HF_74	0	0	0	0	0	0	0	0
HFFG_45	0	0	0	1	0	0	1	0
HFFG_46	0	0	0	0	0	0	0	0
HFFG_47	0	0	0	0	0	0	0	0
HFFG_48	0	0	0	0	0	0	0	0
HFFG_61	0	0	0	0	0	0	0	0
HFFG_62	0	0	0	0	0	0	0	0
HFFG_63	0	0	0	0	0	0	0	0
HFFG_64	0	0	0	0	0	0	0	0
HFFG_75	0	0	0	0	0	0	0	0
HFFG_76	0	0	0	1	0	0	0	0
HFFG_77	0	0	0	0	0	0	0	0

Kingdom	Bacteria	Bacteria	Bacteria	Bacteria	Bacteria	Bacteria	Bacteria	Bacteria	Bacteria
Phylum	Firmicutes	Firmicutes	Firmicutes	Firmicutes	Firmicutes	Firmicutes	Firmicutes	Firmicutes	Firmicutes
Class	Clostridia	Clostridia	Clostridia	Clostridia	Bacilli	Clostridia	Clostridia	Clostridia	Clostridia
Order	Clostridiales	Clostridiales	Clostridiales	Clostridiales	Bacillales	Clostridiales	Clostridiales	Clostridiales	Clostridiales
Family	Clostridiaceae_	Lachnospiraceae	Lachnospiraceae	Lachnospiraceae	Paenibacillaceae_	Lachnospiraceae	Lachnospiraceae	Lachnospiraceae	Lachnospiraceae
Genus	Clostridium_sensu_stricto	Blautia	Acetitomaculum	Lachnobacterium	Paenibacillus	Ruminococcus	Marvinbryantia	Acetatifactor	Blautia
#NAME	ZOTU_0343	ZOTU_0344	ZOTU_0345	ZOTU_0346	ZOTU_0347	ZOTU_0348	ZOTU_0349	ZOTU_0350	ZOTU_0351
CD_49	0	0	0	0	0	0	0	0	0
CD_50	0	0	0	0	0	0	4	0	0
CD_51	1	0	0	0	0	0	0	1	0
CD_52	1	0	0	0	0	0	0	0	0
CD_65	0	0	0	0	0	0	0	0	0
CD_66	1	0	0	0	0	0	0	0	0
CD_67	2	0	0	0	0	0	0	0	0
CD_68	0	0	0	0	0	0	0	0	0
CD_78	1	0	0	0	0	0	0	0	0
CD_79	0	0	0	0	0	0	0	0	0
CD_80	0	0	0	0	0	0	1	0	0
CDFG_53	0	0	0	0	0	0	0	0	0
CDFG_54	0	0	1	1	0	0	0	0	1
CDFG_55	0	0	0	0	0	0	0	0	0
CDFG_56	0	0	0	0	1	0	0	0	0
CDFG_69	0	0	0	0	0	0	0	0	0
CDFG_70	0	0	0	0	0	1	0	0	0
CDFG_71	0	0	0	0	0	0	0	0	0
HF_41	0	0	0	0	0	0	0	0	0
HF_42	0	0	0	0	0	0	0	0	1
HF_43	3	0	0	0	0	0	0	2	0
HF_44	0	0	0	0	0	0	0	0	0
HF_57	1	0	0	0	0	0	0	2	0
HF_58	0	0	0	2	0	0	0	0	1
HF_59	0	0	0	0	0	0	0	0	0
HF_60	0	0	0	0	0	0	0	0	0
HF_72	1	0	0	0	0	0	0	0	0

Kingdom	Bacteria	Bacteria	Bacteria	Bacteria	Bacteria	Bacteria	Bacteria	Bacteria	Bacteria
Phylum	Firmicutes	Firmicutes	Firmicutes	Firmicutes	Firmicutes	Firmicutes	Firmicutes	Firmicutes	Firmicutes
Class	Clostridia	Clostridia	Clostridia	Clostridia	Bacilli	Clostridia	Clostridia	Clostridia	Clostridia
Order	Clostridiales	Clostridiales	Clostridiales	Clostridiales	Bacillales	Clostridiales	Clostridiales	Clostridiales	Clostridiales
Family	Clostridiaceae_	Lachnospiraceae	Lachnospiraceae	Lachnospiraceae	Paenibacillaceae_	Lachnospiraceae	Lachnospiraceae	Lachnospiraceae	Lachnospiraceae
Genus	Clostridium_sensu_stricto	Blautia	Acetitomaculum	Lachnobacterium	Paenibacillus	Ruminococcus	Marvinbryantia	Acetatifactor	Blautia
#NAME	ZOTU_0343	ZOTU_0344	ZOTU_0345	ZOTU_0346	ZOTU_0347	ZOTU_0348	ZOTU_0349	ZOTU_0350	ZOTU_0351
HF_73	1	0	0	0	0	0	1	0	0
HF_74	3	0	0	0	0	0	0	0	1
HFFG_45	0	0	0	0	0	0	0	0	0
HFFG_46	0	0	0	0	0	0	0	0	1
HFFG_47	0	0	0	0	0	0	0	0	0
HFFG_48	0	0	0	0	0	0	0	0	0
HFFG_61	0	0	0	0	0	0	0	0	0
HFFG_62	0	0	0	0	0	0	0	0	0
HFFG_63	0	0	0	0	0	0	0	0	0
HFFG_64	0	0	0	0	0	0	0	0	0
HFFG_75	1	0	0	0	0	0	0	0	0
HFFG_76	4	0	2	0	0	0	0	0	1
HFFG_77	0	0	0	0	0	0	0	0	0

Kingdom	Bacteria	Bacteria	Bacteria	Bacteria	Bacteria	Bacteria	Bacteria	Bacteria	Bacteria
Phylum	Firmicutes	Firmicutes	Firmicutes	Firmicutes	Firmicutes	Firmicutes	Firmicutes	Actinobacteria	Firmicutes
Class	Clostridia	Clostridia	Clostridia	Clostridia	Clostridia	Erysipelotrichia	Bacilli	Actinobacteria	Bacilli
Order	Clostridiales	Clostridiales	Clostridiales	Clostridiales	Clostridiales	Erysipelotrichales	Lactobacillales	Coriobacteriales	Lactobacillales
Family	Ruminococcaceae	Lachnospiraceae	Lachnospiraceae	Lachnospiraceae	Ruminococcaceae	Erysipelotrichaceae	Lactobacillaceae	Coriobacteriaceae	Lactobacillaceae
Genus	Intestinimonas	Acetatifactor	Acetatifactor	Shuttleworthia	Clostridium_IV	Catenisphaera	Lactobacillus	Enterorhabdus	Lactobacillus
#NAME	ZOTU_0352	ZOTU_0353	ZOTU_0354	ZOTU_0355	ZOTU_0356	ZOTU_0357	ZOTU_0358	ZOTU_0359	ZOTU_0360
CD_49	0	0	0	0	0	0	0	15	0
CD_50	0	0	0	0	0	0	0	103	0
CD_51	0	1	0	0	0	0	0	31	0
CD_52	0	0	0	0	0	0	0	95	0
CD_65	0	0	0	0	0	0	0	2	0
CD_66	0	0	0	0	0	0	0	13	0
CD_67	0	0	0	0	0	0	0	17	0
CD_68	0	0	0	0	0	0	0	2	0
CD_78	0	0	0	0	0	0	0	20	0
CD_79	0	0	0	0	0	0	0	1	0
CD_80	0	0	0	0	0	0	0	31	0
CDFG_53	0	0	0	0	1	0	0	1	0
CDFG_54	2	0	0	0	0	0	0	3	0
CDFG_55	0	0	0	0	1	0	2	23	0
CDFG_56	1	0	0	0	1	0	0	1	0
CDFG_69	0	0	0	0	0	0	0	4	0
CDFG_70	0	0	0	0	0	0	0	4	0
CDFG_71	0	0	0	0	2	0	0	5	0
HF_41	0	0	0	0	0	0	0	2	0
HF_42	0	0	0	1	0	0	0	2	0
HF_43	0	1	0	0	0	0	0	0	0
HF_44	0	0	0	0	1	0	0	0	0
HF_57	0	0	0	0	0	0	0	0	0
HF_58	0	0	0	2	0	0	0	0	0
HF_59	0	0	0	0	0	0	0	0	0
HF_60	0	0	0	0	0	0	0	0	0
HF_72	0	0	0	0	0	1	0	0	0

Kingdom	Bacteria	Bacteria	Bacteria	Bacteria	Bacteria	Bacteria	Bacteria	Bacteria	Bacteria
Phylum	Firmicutes	Firmicutes	Firmicutes	Firmicutes	Firmicutes	Firmicutes	Firmicutes	Actinobacteria	Firmicutes
Class	Clostridia	Clostridia	Clostridia	Clostridia	Clostridia	Erysipelotrichia	Bacilli	Actinobacteria	Bacilli
Order	Clostridiales	Clostridiales	Clostridiales	Clostridiales	Clostridiales	Erysipelotrichales	Lactobacillales	Coriobacteriales	Lactobacillales
Family	Ruminococcaceae	Lachnospiraceae	Lachnospiraceae	Lachnospiraceae	Ruminococcaceae	Erysipelotrichaceae	Lactobacillaceae	Coriobacteriaceae	Lactobacillaceae
Genus	Intestinimonas	Acetatifactor	Acetatifactor	Shuttleworthia	Clostridium_IV	Catenisphaera	Lactobacillus	Enterorhabdus	Lactobacillus
#NAME	ZOTU_0352	ZOTU_0353	ZOTU_0354	ZOTU_0355	ZOTU_0356	ZOTU_0357	ZOTU_0358	ZOTU_0359	ZOTU_0360
HF_73	0	0	0	0	1	3	0	0	0
HF_74	0	0	0	0	0	5	0	0	0
HFFG_45	0	0	0	0	0	0	0	0	0
HFFG_46	0	0	0	0	0	0	0	0	0
HFFG_47	0	0	0	0	0	3	0	0	0
HFFG_48	0	0	0	0	1	0	0	0	0
HFFG_61	0	0	0	0	0	0	0	0	0
HFFG_62	0	0	0	0	1	2	0	0	0
HFFG_63	0	0	0	0	0	0	0	0	0
HFFG_64	0	0	0	2	0	5	0	0	0
HFFG_75	0	0	2	1	1	0	0	0	0
HFFG_76	1	0	1	0	2	0	0	0	0
HFFG_77	0	0	0	0	0	0	0	0	0

Kingdom	Bacteria	Bacteria	Bacteria	Bacteria	Bacteria	Bacteria	Bacteria	Bacteria
Phylum	Proteobacteria	Firmicutes	Firmicutes	Firmicutes	Firmicutes	Firmicutes	Firmicutes	Firmicutes
Class	Gammaproteobacteria	Bacilli	Clostridia	Clostridia	Clostridia	Clostridia	Clostridia	Clostridia
Order	Pseudomonadales	Bacillales	Clostridiales	Clostridiales	Clostridiales	Clostridiales	Clostridiales	Clostridiales
Family	Pseudomonadaceae	Staphylococcaceae	Natranaerovirga	Lachnospiraceae	Lachnospiraceae	Lachnospiraceae	Lachnospiraceae	Clostridiaceae_
Genus	Pseudomonas	Staphylococcus	NA	Clostridium_XlVa	Clostridium_XlVa	Clostridium_XlVa	Lachnospiracea_incertae_sedis	Alkaliphilus
#NAME	ZOTU_0361	ZOTU_0362	ZOTU_0363	ZOTU_0364	ZOTU_0365	ZOTU_0366	ZOTU_0367	ZOTU_0368
CD_49	0	4	0	0	0	0	0	0
CD_50	0	1	0	0	0	0	0	0
CD_51	0	7	0	0	1	0	0	0
CD_52	0	1	0	0	0	0	0	0
CD_65	0	2	0	0	0	0	0	0
CD_66	0	3	0	0	0	0	0	0
CD_67	0	20	0	0	0	0	0	0
CD_68	0	0	0	0	0	0	0	0
CD_78	0	7	0	0	0	0	0	0
CD_79	0	0	0	0	0	0	0	0
CD_80	0	1	0	0	0	0	0	0
CDFG_53	0	1	0	0	0	0	0	0
CDFG_54	0	0	0	0	2	0	0	0
CDFG_55	0	1	0	0	2	0	0	0
CDFG_56	0	5	0	0	0	0	0	0
CDFG_69	0	0	0	0	0	0	0	0
CDFG_70	0	0	0	0	0	0	0	0
CDFG_71	0	5	0	0	0	0	0	0
HF_41	0	0	0	0	0	0	0	0
HF_42	0	1	0	0	0	0	0	0
HF_43	0	0	0	0	0	0	0	0
HF_44	0	2	0	0	1	0	0	0
HF_57	0	0	0	0	0	0	0	0
HF_58	0	0	0	0	0	0	0	0
HF_59	0	0	0	0	0	0	0	0
HF_60	0	0	0	0	0	0	0	0
HF_72	0	1	0	0	0	0	0	0

Kingdom	Bacteria	Bacteria	Bacteria	Bacteria	Bacteria	Bacteria	Bacteria	Bacteria
Phylum	Proteobacteria	Firmicutes	Firmicutes	Firmicutes	Firmicutes	Firmicutes	Firmicutes	Firmicutes
Class	Gammaproteobacteria	Bacilli	Clostridia	Clostridia	Clostridia	Clostridia	Clostridia	Clostridia
Order	Pseudomonadales	Bacillales	Clostridiales	Clostridiales	Clostridiales	Clostridiales	Clostridiales	Clostridiales
Family	Pseudomonadaceae	Staphylococcaceae	Natranaerovirga	Lachnospiraceae	Lachnospiraceae	Lachnospiraceae	Lachnospiraceae	Clostridiaceae_
Genus	Pseudomonas	Staphylococcus	NA	Clostridium_XlVa	Clostridium_XlVa	Clostridium_XlVa	Lachnospiracea_incertae_sedis	Alkaliphilus
#NAME	ZOTU_0361	ZOTU_0362	ZOTU_0363	ZOTU_0364	ZOTU_0365	ZOTU_0366	ZOTU_0367	ZOTU_0368
HF_73	0	2	0	0	1	0	0	0
HF_74	0	1	0	0	0	0	0	0
HFFG_45	0	0	0	0	0	0	0	0
HFFG_46	0	0	0	0	0	0	0	0
HFFG_47	0	1	0	0	0	0	0	0
HFFG_48	0	19	0	0	0	0	0	0
HFFG_61	0	0	0	0	0	0	0	0
HFFG_62	0	2	0	0	0	0	0	0
HFFG_63	0	0	0	0	0	0	0	0
HFFG_64	0	1	0	0	0	0	0	0
HFFG_75	0	12	0	0	0	0	0	0
HFFG_76	0	118	0	0	0	0	0	0
HFFG_77	0	3	0	0	0	0	0	0

Kingdom	Bacteria	Bacteria	Bacteria	Bacteria	Bacteria	Bacteria	Bacteria	Bacteria	Bacteria
Phylum	Firmicutes	Firmicutes	Actinobacteria	Firmicutes	Firmicutes	Firmicutes	Firmicutes	Firmicutes	Firmicutes
Class	Clostridia	Clostridia	Actinobacteria	Bacilli	Bacilli	Bacilli	Bacilli	Clostridia	Clostridia
Order	Clostridiales	Clostridiales	Actinomycetales	Lactobacillales	Bacillales	Lactobacillales	Bacillales	Clostridiales	Clostridiales
Family	Clostridiaceae_	Peptostreptococcaceae	Dietziaceae	Enterococcaceae	Planococcaceae	Streptococcaceae	Bacillaceae_	Lachnospiraceae	Ruminococcaceae
Genus	Clostridium_sensu_stricto	Clostridium_XI	Dietzia	Enterococcus	Viridibacillus	Streptococcus	Bacillus	Acetatifactor	Clostridium_IV
#NAME	ZOTU_0369	ZOTU_0370	ZOTU_0371	ZOTU_0372	ZOTU_0373	ZOTU_0374	ZOTU_0375	ZOTU_0376	ZOTU_0377
CD_49	0	0	0	1	6	0	0	0	0
CD_50	1	0	0	0	0	0	0	1	1
CD_51	0	0	0	39	13	0	0	0	10
CD_52	6	0	0	0	3	0	2	1	0
CD_65	0	0	0	1	115	0	29	0	0
CD_66	0	0	0	1	399	0	139	0	0
CD_67	3	0	0	0	14	0	5	0	0
CD_68	0	0	0	0	1	0	0	0	0
CD_78	0	0	0	2	1	0	1	0	0
CD_79	0	0	0	0	1	0	0	0	1
CD_80	1	0	0	6	0	0	0	0	1
CDFG_53	1	0	0	0	10	0	0	0	0
CDFG_54	0	0	0	11	0	0	2	0	0
CDFG_55	0	0	0	17	13	0	1	0	0
CDFG_56	0	0	0	1	270	0	48	0	0
CDFG_69	0	0	0	1	1	0	1	0	0
CDFG_70	0	0	0	0	0	0	0	0	0
CDFG_71	0	0	0	0	2	0	0	0	0
HF_41	0	0	0	0	3	0	3	0	0
HF_42	0	0	0	0	12	0	0	0	0
HF_43	0	0	0	0	0	0	1	1	0
HF_44	0	0	0	1	2	0	2	0	0
HF_57	0	0	0	0	6	0	0	1	0
HF_58	0	0	0	0	59	0	5	6	0
HF_59	0	0	0	0	356	2	15	1	1
HF_60	0	0	0	0	19	0	0	0	0
HF_72	0	0	0	0	5	0	2	0	0

Kingdom	Bacteria	Bacteria	Bacteria	Bacteria	Bacteria	Bacteria	Bacteria	Bacteria	Bacteria
Phylum	Firmicutes	Firmicutes	Actinobacteria	Firmicutes	Firmicutes	Firmicutes	Firmicutes	Firmicutes	Firmicutes
Class	Clostridia	Clostridia	Actinobacteria	Bacilli	Bacilli	Bacilli	Bacilli	Clostridia	Clostridia
Order	Clostridiales	Clostridiales	Actinomycetales	Lactobacillales	Bacillales	Lactobacillales	Bacillales	Clostridiales	Clostridiales
Family	Clostridiaceae_	Peptostreptococcaceae	Dietziaceae	Enterococcaceae	Planococcaceae	Streptococcaceae	Bacillaceae_	Lachnospiraceae	Ruminococcaceae
Genus	Clostridium_sensu_stricto	Clostridium_XI	Dietzia	Enterococcus	Viridibacillus	Streptococcus	Bacillus	Acetatifactor	Clostridium_IV
#NAME	ZOTU_0369	ZOTU_0370	ZOTU_0371	ZOTU_0372	ZOTU_0373	ZOTU_0374	ZOTU_0375	ZOTU_0376	ZOTU_0377
HF_73	0	0	0	0	5	0	2	0	0
HF_74	0	0	0	0	0	1	4	0	0
HFFG_45	0	0	0	2	0	0	0	0	0
HFFG_46	0	0	0	0	1	0	1	0	0
HFFG_47	0	0	0	0	0	0	3	0	0
HFFG_48	0	0	0	0	27	0	22	0	0
HFFG_61	0	0	0	0	0	0	0	0	0
HFFG_62	0	0	0	0	4	0	1	0	0
HFFG_63	0	0	0	0	0	0	0	0	0
HFFG_64	0	0	0	1	47	0	3	0	0
HFFG_75	0	1	0	1	704	0	85	0	0
HFFG_76	0	0	1	7	569	0	36	0	0
HFFG_77	0	0	0	0	4	0	0	0	0

Kingdom	Bacteria	Bacteria	Bacteria	Bacteria	Bacteria	Bacteria	Bacteria	Bacteria
Phylum	Firmicutes	Firmicutes	Firmicutes	Firmicutes	Firmicutes	Firmicutes	Firmicutes	Firmicutes
Class	Clostridia	Clostridia	Clostridia	Clostridia	Bacilli	Clostridia	Clostridia	Clostridia
Order	Clostridiales	Clostridiales	Clostridiales	Clostridiales	Bacillales	Clostridiales	Clostridiales	Clostridiales
Family	Ruminococcaceae	Lachnospiraceae	Lachnospiraceae	Clostridiaceae_	Planococcaceae	Lachnospiraceae	Clostridiaceae_	Lachnospiraceae
Genus	Oscillibacter	Acetatifactor	Clostridium_XlVa	Clostridium_sensu_stricto	Viridibacillus	Clostridium_XlVa	Clostridium_sensu_stricto	Clostridium_XlVa
#NAME	ZOTU_0378	ZOTU_0379	ZOTU_0380	ZOTU_0381	ZOTU_0382	ZOTU_0383	ZOTU_0384	ZOTU_0385
CD_49	0	0	0	0	3	0	0	0
CD_50	0	0	0	0	0	0	0	0
CD_51	1	0	0	0	2	0	0	1
CD_52	0	1	0	0	1	0	0	0
CD_65	0	0	0	0	1	0	0	0
CD_66	0	0	0	0	2	0	0	0
CD_67	0	0	0	0	7	0	0	0
CD_68	0	0	0	0	0	0	0	0
CD_78	0	0	0	0	0	0	0	0
CD_79	0	0	0	0	1	0	0	0
CD_80	0	0	0	0	0	0	0	0
CDFG_53	0	0	0	0	0	0	0	0
CDFG_54	2	0	8	0	0	0	0	0
CDFG_55	0	0	0	0	0	0	0	0
CDFG_56	0	0	0	0	0	0	0	0
CDFG_69	0	0	0	0	0	0	0	0
CDFG_70	1	0	0	0	0	0	0	0
CDFG_71	0	0	0	0	0	0	0	0
HF_41	0	0	3	0	0	0	0	0
HF_42	0	0	0	15	0	3	0	0
HF_43	0	0	0	0	0	0	0	0
HF_44	0	0	0	0	0	0	0	0
HF_57	0	0	0	0	0	0	0	0
HF_58	0	1	0	0	2	0	0	0
HF_59	0	1	0	0	0	0	0	0
HF_60	0	0	0	0	0	0	0	0
HF_72	0	0	0	0	2	0	0	0

Kingdom	Bacteria	Bacteria	Bacteria	Bacteria	Bacteria	Bacteria	Bacteria	Bacteria
Phylum	Firmicutes	Firmicutes	Firmicutes	Firmicutes	Firmicutes	Firmicutes	Firmicutes	Firmicutes
Class	Clostridia	Clostridia	Clostridia	Clostridia	Bacilli	Clostridia	Clostridia	Clostridia
Order	Clostridiales	Clostridiales	Clostridiales	Clostridiales	Bacillales	Clostridiales	Clostridiales	Clostridiales
Family	Ruminococcaceae	Lachnospiraceae	Lachnospiraceae	Clostridiaceae_	Planococcaceae	Lachnospiraceae	Clostridiaceae_	Lachnospiraceae
Genus	Oscillibacter	Acetatifactor	Clostridium_XlVa	Clostridium_sensu_stricto	Viridibacillus	Clostridium_XlVa	Clostridium_sensu_stricto	Clostridium_XlVa
#NAME	ZOTU_0378	ZOTU_0379	ZOTU_0380	ZOTU_0381	ZOTU_0382	ZOTU_0383	ZOTU_0384	ZOTU_0385
HF_73	0	0	0	0	3	0	0	0
HF_74	0	0	0	0	0	0	0	0
HFFG_45	0	0	0	0	0	0	0	0
HFFG_46	0	0	0	0	0	0	0	1
HFFG_47	0	0	0	0	0	0	0	0
HFFG_48	0	0	0	0	0	0	0	0
HFFG_61	0	0	0	0	0	0	0	0
HFFG_62	0	0	0	0	0	0	0	0
HFFG_63	0	0	0	0	0	0	0	0
HFFG_64	0	0	0	0	0	0	0	0
HFFG_75	0	0	0	0	6	0	0	0
HFFG_76	0	0	0	0	38	0	0	0
HFFG_77	0	0	0	0	0	0	0	0

Kingdom	Bacteria	Bacteria	Bacteria	Bacteria	Bacteria	Bacteria	Bacteria	Bacteria
Phylum	Firmicutes	Firmicutes	Firmicutes	Proteobacteria	Cyanobacteria_Chloroplast	Tenericutes	Cyanobacteria_Chloroplast	Firmicutes
Class	Clostridia	Clostridia	Negativicutes	Betaproteobacteria	Chloroplast	Mollicutes	Chloroplast	Clostridia
Order	Clostridiales	Clostridiales	Selenomonadales	Neisseriales	Chloroplast	Acholeplasmatales	Chloroplast	Clostridiales
Family	Ruminococcaceae	Clostridiaceae_	Veillonellaceae	Neisseriaceae	Streptophyta	Acholeplasmataceae	Streptophyta	Lachnospiraceae
Genus	Clostridium_IV	Alkaliphilus	Veillonella	Neisseria	NA	Acholeplasma	NA	Coprococcus
#NAME	ZOTU_0386	ZOTU_0387	ZOTU_0388	ZOTU_0389	ZOTU_0390	ZOTU_0391	ZOTU_0392	ZOTU_0393
CD_49	0	0	0	0	0	0	0	0
CD_50	0	0	0	0	0	0	0	0
CD_51	0	0	0	0	0	0	0	0
CD_52	0	0	0	0	0	0	0	0
CD_65	0	0	0	0	0	0	0	0
CD_66	0	0	0	0	0	0	0	0
CD_67	0	0	0	0	0	0	0	0
CD_68	0	0	0	0	0	0	0	0
CD_78	0	0	0	0	0	0	0	0
CD_79	0	0	0	0	0	0	0	0
CD_80	0	0	0	0	0	0	0	0
CDFG_53	0	0	0	0	0	0	0	0
CDFG_54	0	0	0	0	0	0	0	0
CDFG_55	0	0	0	0	4	0	1	0
CDFG_56	0	0	0	0	0	0	0	0
CDFG_69	0	0	0	0	0	0	0	0
CDFG_70	0	0	0	0	0	0	0	0
CDFG_71	0	0	0	0	0	0	0	0
HF_41	1	0	0	0	0	0	0	0
HF_42	0	0	0	0	0	0	0	0
HF_43	0	0	0	0	0	0	0	0
HF_44	0	0	0	0	0	0	0	0
HF_57	0	0	0	0	0	0	0	0
HF_58	0	0	0	0	0	0	0	1
HF_59	0	0	0	0	0	2	0	0
HF_60	0	0	0	0	0	4	0	0
HF_72	0	0	0	0	0	0	0	0

Kingdom	Bacteria	Bacteria	Bacteria	Bacteria	Bacteria	Bacteria	Bacteria	Bacteria
Phylum	Firmicutes	Firmicutes	Firmicutes	Proteobacteria	Cyanobacteria_Chloroplast	Tenericutes	Cyanobacteria_Chloroplast	Firmicutes
Class	Clostridia	Clostridia	Negativicutes	Betaproteobacteria	Chloroplast	Mollicutes	Chloroplast	Clostridia
Order	Clostridiales	Clostridiales	Selenomonadales	Neisseriales	Chloroplast	Acholeplasmatales	Chloroplast	Clostridiales
Family	Ruminococcaceae	Clostridiaceae_	Veillonellaceae	Neisseriaceae	Streptophyta	Acholeplasmataceae	Streptophyta	Lachnospiraceae
Genus	Clostridium_IV	Alkaliphilus	Veillonella	Neisseria	NA	Acholeplasma	NA	Coprococcus
#NAME	ZOTU_0386	ZOTU_0387	ZOTU_0388	ZOTU_0389	ZOTU_0390	ZOTU_0391	ZOTU_0392	ZOTU_0393
HF_73	0	0	0	0	0	0	0	0
HF_74	0	0	0	0	0	0	0	1
HFFG_45	0	0	0	0	0	0	0	0
HFFG_46	0	0	0	0	0	1	0	0
HFFG_47	0	0	0	0	0	0	0	0
HFFG_48	0	0	0	0	0	0	0	0
HFFG_61	0	0	0	0	0	0	0	0
HFFG_62	0	0	0	0	0	0	0	0
HFFG_63	0	0	0	0	0	0	0	0
HFFG_64	0	0	0	0	0	0	0	0
HFFG_75	0	0	0	0	0	2	1	0
HFFG_76	0	0	0	0	0	1	0	0
HFFG_77	1	0	0	0	0	0	0	0

Kingdom	Bacteria	Bacteria	Bacteria	Bacteria	Bacteria	Bacteria	Bacteria
Phylum	Bacteroidetes	Firmicutes	Bacteroidetes	Proteobacteria	Actinobacteria	Firmicutes	Firmicutes
Class	Bacteroidia	Negativicutes	Bacteroidia	Betaproteobacteria	Actinobacteria	Clostridia	Clostridia
Order	Bacteroidales	Selenomonadales	Bacteroidales	Burkholderiales	Actinomycetales	Clostridiales	Clostridiales
Family	Porphyromonadaceae	Veillonellaceae	Porphyromonadaceae	Burkholderiaceae	Corynebacteriaceae	Clostridiales_Incertae_Sedis_XIII	Lachnospiraceae
Genus	Parabacteroides	Veillonella	Parabacteroides	Burkholderia	Corynebacterium	Anaerovorax	Acetatifactor
#NAME	ZOTU_0394	ZOTU_0395	ZOTU_0396	ZOTU_0397	ZOTU_0398	ZOTU_0399	ZOTU_0400
CD_49	0	0	0	0	0	1	0
CD_50	0	0	0	0	0	3	0
CD_51	0	0	0	0	0	1	0
CD_52	0	0	0	0	0	0	0
CD_65	4	0	0	0	0	0	0
CD_66	9	0	0	0	0	0	0
CD_67	25	0	0	0	0	0	0
CD_68	10	0	0	0	0	0	0
CD_78	3	0	0	0	0	0	0
CD_79	2	0	0	0	0	0	0
CD_80	2	0	0	0	0	0	0
CDFG_53	0	0	0	0	0	0	0
CDFG_54	0	0	0	0	1	0	0
CDFG_55	0	0	0	0	0	0	0
CDFG_56	0	0	0	0	0	0	0
CDFG_69	0	0	0	0	0	0	0
CDFG_70	0	0	0	0	0	0	0
CDFG_71	0	0	0	0	0	0	0
HF_41	1	0	0	0	0	0	0
HF_42	0	0	0	0	0	0	0
HF_43	0	2	0	0	0	0	0
HF_44	0	0	0	0	0	1	0
HF_57	0	0	0	0	0	0	0
HF_58	0	0	0	0	0	0	0
HF_59	0	0	0	0	0	0	0
HF_60	0	1	0	0	0	1	0
HF_72	0	0	0	0	0	0	0

Kingdom	Bacteria	Bacteria	Bacteria	Bacteria	Bacteria	Bacteria	Bacteria
Phylum	Bacteroidetes	Firmicutes	Bacteroidetes	Proteobacteria	Actinobacteria	Firmicutes	Firmicutes
Class	Bacteroidia	Negativicutes	Bacteroidia	Betaproteobacteria	Actinobacteria	Clostridia	Clostridia
Order	Bacteroidales	Selenomonadales	Bacteroidales	Burkholderiales	Actinomycetales	Clostridiales	Clostridiales
Family	Porphyromonadaceae	Veillonellaceae	Porphyromonadaceae	Burkholderiaceae	Corynebacteriaceae	Clostridiales_Incertae_Sedis_XIII	Lachnospiraceae
Genus	Parabacteroides	Veillonella	Parabacteroides	Burkholderia	Corynebacterium	Anaerovorax	Acetatifactor
#NAME	ZOTU_0394	ZOTU_0395	ZOTU_0396	ZOTU_0397	ZOTU_0398	ZOTU_0399	ZOTU_0400
HF_73	0	0	0	0	0	0	0
HF_74	0	0	0	0	0	0	0
HFFG_45	0	0	0	0	0	0	0
HFFG_46	0	0	0	0	0	0	0
HFFG_47	0	0	0	0	0	0	0
HFFG_48	0	0	0	0	0	0	0
HFFG_61	0	0	0	0	0	0	0
HFFG_62	0	0	0	0	0	0	0
HFFG_63	0	0	0	0	0	0	0
HFFG_64	0	0	0	0	0	0	0
HFFG_75	0	0	0	0	0	0	0
HFFG_76	0	0	0	0	0	0	0
HFFG_77	0	0	0	0	0	0	0

Kingdom	Bacteria	Bacteria	Bacteria	Bacteria	Bacteria	Bacteria	Bacteria	Bacteria
Phylum	Firmicutes	Proteobacteria	Firmicutes	Firmicutes	Actinobacteria	Firmicutes	Firmicutes	Firmicutes
Class	Erysipelotrichia	Betaproteobacteria	Negativicutes	Clostridia	Actinobacteria	Clostridia	Clostridia	Clostridia
Order	Erysipelotrichales	Burkholderiales	Selenomonadales	Clostridiales	Actinomycetales	Clostridiales	Clostridiales	Clostridiales
Family	Erysipelotrichaceae	Burkholderiaceae	Acidaminococcaceae	Eubacteriaceae	Corynebacteriaceae	Lachnospiraceae	Ruminococcaceae	Ruminococcaceae
Genus	Clostridium_XVIII	Ralstonia	Phascolarctobacterium	Anaerofustis	Corynebacterium	Acetatifactor	Oscillibacter	Ruminococcus
#NAME	ZOTU_0401	ZOTU_0402	ZOTU_0403	ZOTU_0404	ZOTU_0405	ZOTU_0406	ZOTU_0407	ZOTU_0408
CD_49	4	0	0	0	0	0	0	0
CD_50	4	0	0	0	0	0	0	0
CD_51	4	0	0	0	0	0	0	0
CD_52	6	0	0	0	0	0	0	0
CD_65	9	0	0	0	0	0	0	0
CD_66	4	0	0	0	0	0	0	0
CD_67	2	0	0	0	0	0	1	0
CD_68	1	0	0	0	0	0	0	0
CD_78	24	0	0	0	0	0	0	0
CD_79	1	0	0	0	0	0	0	0
CD_80	8	0	0	0	0	0	0	0
CDFG_53	1	0	0	0	0	0	0	0
CDFG_54	13	0	0	0	0	0	1	0
CDFG_55	12	0	0	0	0	0	0	0
CDFG_56	1	0	0	0	0	0	0	0
CDFG_69	1	0	0	1	0	0	0	0
CDFG_70	2	0	0	0	0	0	0	0
CDFG_71	0	0	0	0	0	0	0	0
HF_41	0	0	0	0	0	0	0	0
HF_42	0	0	0	0	0	0	0	0
HF_43	0	0	0	0	0	0	0	0
HF_44	0	0	0	0	0	0	0	0
HF_57	1	0	0	0	0	0	0	0
HF_58	8	0	0	0	0	0	0	0
HF_59	0	0	0	0	0	0	0	0
HF_60	0	0	0	0	0	0	0	0
HF_72	1	0	0	0	0	0	0	0

Kingdom	Bacteria	Bacteria	Bacteria	Bacteria	Bacteria	Bacteria	Bacteria	Bacteria
Phylum	Firmicutes	Proteobacteria	Firmicutes	Firmicutes	Actinobacteria	Firmicutes	Firmicutes	Firmicutes
Class	Erysipelotrichia	Betaproteobacteria	Negativicutes	Clostridia	Actinobacteria	Clostridia	Clostridia	Clostridia
Order	Erysipelotrichales	Burkholderiales	Selenomonadales	Clostridiales	Actinomycetales	Clostridiales	Clostridiales	Clostridiales
Family	Erysipelotrichaceae	Burkholderiaceae	Acidaminococcaceae	Eubacteriaceae	Corynebacteriaceae	Lachnospiraceae	Ruminococcaceae	Ruminococcaceae
Genus	Clostridium_XVIII	Ralstonia	Phascolarctobacterium	Anaerofustis	Corynebacterium	Acetatifactor	Oscillibacter	Ruminococcus
#NAME	ZOTU_0401	ZOTU_0402	ZOTU_0403	ZOTU_0404	ZOTU_0405	ZOTU_0406	ZOTU_0407	ZOTU_0408
HF_73	0	0	0	0	0	0	0	0
HF_74	1	0	0	0	0	0	0	0
HFFG_45	0	0	0	0	0	0	0	0
HFFG_46	1	0	0	0	0	0	1	0
HFFG_47	0	0	0	0	0	0	0	0
HFFG_48	0	0	0	0	0	0	0	0
HFFG_61	1	0	0	0	0	0	0	0
HFFG_62	2	0	0	0	0	0	0	0
HFFG_63	0	0	0	0	0	0	0	0
HFFG_64	0	0	0	0	0	1	0	0
HFFG_75	10	0	0	0	0	0	1	0
HFFG_76	17	0	0	0	0	1	2	0
HFFG_77	3	1	0	0	0	0	0	0

Kingdom	Bacteria	Bacteria	Bacteria	Bacteria	Bacteria	Bacteria	Bacteria
Phylum	Bacteroidetes	Firmicutes	Actinobacteria	Proteobacteria	Cyanobacteria_Chloroplast	Bacteroidetes	Tenericutes
Class	Bacteroidia	Clostridia	Actinobacteria	Betaproteobacteria	Chloroplast	Bacteroidia	Mollicutes
Order	Bacteroidales	Clostridiales	Coriobacteriales	Neisseriales	Chloroplast	Bacteroidales	Acholeplasmatales
Family	Porphyromonadaceae	Lachnospiraceae	Coriobacteriaceae	Neisseriaceae	Streptophyta	Porphyromonadaceae	Acholeplasmataceae
Genus	Parabacteroides	Clostridium_XlVa	Atopobium	Neisseria	NA	Parabacteroides	Acholeplasma
#NAME	ZOTU_0409	ZOTU_0410	ZOTU_0411	ZOTU_0412	ZOTU_0413	ZOTU_0414	ZOTU_0415
CD_49	0	0	0	0	0	0	0
CD_50	0	0	0	0	0	0	0
CD_51	0	0	0	0	0	0	0
CD_52	0	0	0	0	0	0	0
CD_65	0	0	0	0	0	0	0
CD_66	0	0	0	0	0	3	0
CD_67	0	0	0	0	0	26	0
CD_68	0	0	0	0	0	1	0
CD_78	0	0	0	0	0	1	0
CD_79	0	0	0	0	0	0	0
CD_80	0	0	0	0	0	0	0
CDFG_53	0	0	1	0	0	0	0
CDFG_54	0	0	1	0	0	0	2
CDFG_55	0	0	0	0	1	0	0
CDFG_56	0	0	0	0	0	0	1
CDFG_69	0	0	0	0	0	0	0
CDFG_70	0	0	0	0	0	0	0
CDFG_71	0	0	0	0	0	0	0
HF_41	0	0	0	0	0	0	0
HF_42	0	0	0	0	0	0	3
HF_43	0	0	0	0	0	0	0
HF_44	0	0	0	0	0	0	0
HF_57	0	0	0	0	0	0	0
HF_58	0	0	0	0	0	0	0
HF_59	0	0	0	0	0	0	0
HF_60	0	0	0	0	0	0	0
HF_72	0	0	0	0	0	0	0

Kingdom	Bacteria	Bacteria	Bacteria	Bacteria	Bacteria	Bacteria	Bacteria
Phylum	Bacteroidetes	Firmicutes	Actinobacteria	Proteobacteria	Cyanobacteria_Chloroplast	Bacteroidetes	Tenericutes
Class	Bacteroidia	Clostridia	Actinobacteria	Betaproteobacteria	Chloroplast	Bacteroidia	Mollicutes
Order	Bacteroidales	Clostridiales	Coriobacteriales	Neisseriales	Chloroplast	Bacteroidales	Acholeplasmatales
Family	Porphyromonadaceae	Lachnospiraceae	Coriobacteriaceae	Neisseriaceae	Streptophyta	Porphyromonadaceae	Acholeplasmataceae
Genus	Parabacteroides	Clostridium_XlVa	Atopobium	Neisseria	NA	Parabacteroides	Acholeplasma
#NAME	ZOTU_0409	ZOTU_0410	ZOTU_0411	ZOTU_0412	ZOTU_0413	ZOTU_0414	ZOTU_0415
HF_73	0	0	0	0	0	0	0
HF_74	0	0	0	0	0	0	0
HFFG_45	0	0	0	0	0	0	0
HFFG_46	0	0	0	0	0	0	0
HFFG_47	0	0	0	0	0	0	0
HFFG_48	0	0	0	0	0	0	0
HFFG_61	0	0	0	0	0	0	0
HFFG_62	0	0	0	0	0	0	0
HFFG_63	0	0	0	0	0	0	0
HFFG_64	0	0	0	0	0	0	0
HFFG_75	0	0	0	0	1	0	0
HFFG_76	0	0	0	0	0	0	0
HFFG_77	0	0	0	0	0	0	0

Kingdom	Bacteria	Bacteria	Bacteria	Bacteria	Bacteria	Bacteria	Bacteria	Bacteria
Phylum	Firmicutes	Firmicutes	Firmicutes	Cyanobacteria_Chloroplast	Firmicutes	Actinobacteria	Firmicutes	Firmicutes
Class	Clostridia	Clostridia	Clostridia	Chloroplast	Clostridia	Actinobacteria	Bacilli	Bacilli
Order	Clostridiales	Clostridiales	Clostridiales	Chloroplast	Clostridiales	Coriobacteriales	Bacillales	Bacillales
Family	Clostridiaceae_	Lachnospiraceae	Clostridiaceae_	Streptophyta	Lachnospiraceae	Coriobacteriaceae	Staphylococcaceae	Bacillaceae_
Genus	Clostridium_sensu_stricto	Clostridium_XlVa	Clostridium_sensu_stricto	NA	Butyrivibrio	Enterorhabdus	Staphylococcus	Caldibacillus
#NAME	ZOTU_0416	ZOTU_0417	ZOTU_0418	ZOTU_0419	ZOTU_0420	ZOTU_0421	ZOTU_0422	ZOTU_0423
CD_49	0	0	1	0	0	3	1	0
CD_50	0	0	0	0	0	23	0	0
CD_51	0	0	1	0	0	4	0	0
CD_52	0	0	0	0	0	25	0	0
CD_65	0	0	0	0	0	2	5	0
CD_66	0	0	0	0	0	5	45	0
CD_67	0	0	0	0	0	3	7	0
CD_68	0	0	0	0	0	0	0	0
CD_78	0	0	0	0	0	1	0	0
CD_79	0	0	0	0	0	0	0	0
CD_80	0	0	0	0	0	10	0	0
CDFG_53	0	0	0	0	0	0	1	0
CDFG_54	0	0	0	0	0	2	0	0
CDFG_55	0	0	0	1	0	6	0	0
CDFG_56	0	0	0	0	0	0	4	0
CDFG_69	0	0	0	0	0	1	1	0
CDFG_70	0	0	0	0	0	1	0	0
CDFG_71	0	0	0	0	0	0	0	0
HF_41	0	0	0	0	0	0	0	0
HF_42	0	0	0	0	0	2	0	0
HF_43	0	0	2	0	0	0	0	0
HF_44	0	0	0	0	0	0	0	0
HF_57	0	0	0	0	0	0	0	0
HF_58	0	0	0	0	0	0	1	0
HF_59	0	0	0	0	0	0	4	0
HF_60	0	0	0	0	0	0	0	0
HF_72	0	0	0	0	0	2	21	0

Kingdom	Bacteria	Bacteria	Bacteria	Bacteria	Bacteria	Bacteria	Bacteria	Bacteria
Phylum	Firmicutes	Firmicutes	Firmicutes	Cyanobacteria_Chloroplast	Firmicutes	Actinobacteria	Firmicutes	Firmicutes
Class	Clostridia	Clostridia	Clostridia	Chloroplast	Clostridia	Actinobacteria	Bacilli	Bacilli
Order	Clostridiales	Clostridiales	Clostridiales	Chloroplast	Clostridiales	Coriobacteriales	Bacillales	Bacillales
Family	Clostridiaceae_	Lachnospiraceae	Clostridiaceae_	Streptophyta	Lachnospiraceae	Coriobacteriaceae	Staphylococcaceae	Bacillaceae_
Genus	Clostridium_sensu_stricto	Clostridium_XlVa	Clostridium_sensu_stricto	NA	Butyrivibrio	Enterorhabdus	Staphylococcus	Caldibacillus
#NAME	ZOTU_0416	ZOTU_0417	ZOTU_0418	ZOTU_0419	ZOTU_0420	ZOTU_0421	ZOTU_0422	ZOTU_0423
HF_73	0	0	0	0	0	1	0	0
HF_74	0	0	0	0	0	0	0	0
HFFG_45	0	0	0	0	0	0	2	0
HFFG_46	0	0	0	0	0	0	2	0
HFFG_47	0	0	0	0	0	0	1	0
HFFG_48	0	0	0	0	0	1	8	0
HFFG_61	0	0	0	0	0	0	0	0
HFFG_62	0	0	0	0	0	0	0	1
HFFG_63	0	0	0	1	0	0	1	0
HFFG_64	0	0	0	0	0	0	0	0
HFFG_75	0	0	0	0	0	0	9	0
HFFG_76	0	1	0	0	0	0	6	0
HFFG_77	0	0	0	0	0	0	0	0

Kingdom	Bacteria	Bacteria	Bacteria	Bacteria	Bacteria	Bacteria	Bacteria	Bacteria
Phylum	Actinobacteria	Proteobacteria	Firmicutes	Firmicutes	Firmicutes	Firmicutes	Actinobacteria	Firmicutes
Class	Actinobacteria	Gammaproteobacteria	Bacilli	Bacilli	Bacilli	Clostridia	Actinobacteria	Bacilli
Order	Actinomycetales	Pasteurellales	Lactobacillales	Bacillales	Bacillales	Clostridiales	Actinomycetales	Bacillales
Family	Corynebacteriaceae	Pasteurellaceae	Lactobacillaceae	Bacillaceae_	Bacillales_Incertae_Sedis_XI	Clostridiaceae_	Actinomycetaceae	Staphylococcaceae
Genus	Corynebacterium	Actinobacillus	Lactobacillus	Bacillus	Gemella	Clostridium_sensu_stricto	Actinomyces	Staphylococcus
#NAME	ZOTU_0424	ZOTU_0425	ZOTU_0426	ZOTU_0427	ZOTU_0428	ZOTU_0429	ZOTU_0430	ZOTU_0431
CD_49	0	0	0	0	0	0	0	1
CD_50	0	0	0	0	0	0	0	0
CD_51	0	0	0	0	0	0	0	0
CD_52	0	0	0	0	0	1	0	0
CD_65	0	0	0	0	0	0	0	0
CD_66	0	0	0	0	0	1	0	0
CD_67	0	0	0	0	0	0	0	1
CD_68	0	0	0	0	0	0	0	0
CD_78	0	0	0	0	0	1	0	0
CD_79	0	0	0	0	0	0	0	0
CD_80	0	0	0	0	0	0	0	0
CDFG_53	0	0	0	0	0	0	0	0
CDFG_54	0	0	0	0	0	0	0	0
CDFG_55	0	0	0	0	0	0	0	0
CDFG_56	0	0	0	0	0	0	0	0
CDFG_69	0	0	0	0	0	0	0	0
CDFG_70	0	0	0	0	0	0	0	0
CDFG_71	0	0	0	0	0	0	0	0
HF_41	0	0	0	0	0	0	0	0
HF_42	0	0	0	0	0	0	0	0
HF_43	0	0	0	0	0	0	0	0
HF_44	1	0	0	0	0	0	0	2
HF_57	0	0	0	0	0	0	0	0
HF_58	0	0	0	0	0	0	0	0
HF_59	0	0	0	0	0	0	0	0
HF_60	0	0	0	0	0	0	0	0
HF_72	0	0	0	0	0	0	0	2

Kingdom	Bacteria	Bacteria	Bacteria	Bacteria	Bacteria	Bacteria	Bacteria	Bacteria
Phylum	Actinobacteria	Proteobacteria	Firmicutes	Firmicutes	Firmicutes	Firmicutes	Actinobacteria	Firmicutes
Class	Actinobacteria	Gammaproteobacteria	Bacilli	Bacilli	Bacilli	Clostridia	Actinobacteria	Bacilli
Order	Actinomycetales	Pasteurellales	Lactobacillales	Bacillales	Bacillales	Clostridiales	Actinomycetales	Bacillales
Family	Corynebacteriaceae	Pasteurellaceae	Lactobacillaceae	Bacillaceae_	Bacillales_Incertae_Sedis_XI	Clostridiaceae_	Actinomycetaceae	Staphylococcaceae
Genus	Corynebacterium	Actinobacillus	Lactobacillus	Bacillus	Gemella	Clostridium_sensu_stricto	Actinomyces	Staphylococcus
#NAME	ZOTU_0424	ZOTU_0425	ZOTU_0426	ZOTU_0427	ZOTU_0428	ZOTU_0429	ZOTU_0430	ZOTU_0431
HF_73	1	0	0	0	0	0	0	1
HF_74	0	0	0	0	0	0	0	1
HFFG_45	0	0	0	0	0	0	0	1
HFFG_46	0	0	0	0	0	0	0	1
HFFG_47	0	0	0	0	0	0	0	1
HFFG_48	0	0	0	0	0	0	0	1
HFFG_61	0	0	0	0	0	0	0	0
HFFG_62	0	0	0	0	0	0	0	2
HFFG_63	0	0	0	0	0	0	0	0
HFFG_64	1	0	0	0	0	0	0	0
HFFG_75	0	0	0	0	0	0	0	1
HFFG_76	0	0	0	0	0	0	0	4
HFFG_77	0	0	0	0	0	0	0	1

Kingdom	Bacteria	Bacteria	Bacteria	Bacteria	Bacteria	Bacteria	Bacteria	Bacteria
Phylum	Proteobacteria	Firmicutes	Firmicutes	Firmicutes	Actinobacteria	Firmicutes	Firmicutes	Bacteroidetes
Class	Alphaproteobacteria	Erysipelotrichia	Clostridia	Clostridia	Actinobacteria	Clostridia	Clostridia	Bacteroidia
Order	Rhizobiales	Erysipelotrichales	Clostridiales	Clostridiales	Coriobacteriales	Clostridiales	Clostridiales	Bacteroidales
Family	Bradyrhizobiaceae	Erysipelotrichaceae	Clostridiaceae_	Lachnospiraceae	Coriobacteriaceae	Lachnospiraceae	Lachnospiraceae	Porphyromonadaceae
Genus	Bradyrhizobium	Erysipelotrichaceae_incertae_sedis	Alkaliphilus	Clostridium_XlVa	Enterorhabdus	Clostridium_XlVa	Clostridium_XlVa	Parabacteroides
#NAME	ZOTU_0432	ZOTU_0433	ZOTU_0434	ZOTU_0435	ZOTU_0436	ZOTU_0437	ZOTU_0438	ZOTU_0439
CD_49	0	0	0	0	0	0	0	0
CD_50	0	0	0	0	0	0	0	0
CD_51	0	0	0	0	0	0	0	0
CD_52	0	0	0	0	1	0	0	0
CD_65	0	0	0	0	0	0	0	0
CD_66	0	0	0	0	0	0	0	0
CD_67	0	0	0	0	0	0	0	0
CD_68	0	0	0	0	0	0	0	0
CD_78	1	0	0	0	0	0	0	0
CD_79	0	0	0	0	0	0	0	0
CD_80	0	0	0	0	0	0	0	0
CDFG_53	0	0	0	0	0	0	0	0
CDFG_54	0	0	0	0	1	0	0	0
CDFG_55	0	0	0	0	0	0	0	0
CDFG_56	0	0	0	0	0	0	0	0
CDFG_69	0	0	0	0	0	0	0	0
CDFG_70	0	0	0	0	0	0	0	0
CDFG_71	0	0	0	0	0	0	0	0
HF_41	0	0	0	0	0	0	0	0
HF_42	0	0	15	0	0	0	0	0
HF_43	0	0	0	0	0	0	0	0
HF_44	0	0	0	0	0	0	0	0
HF_57	0	0	0	0	0	0	0	0
HF_58	0	0	0	0	0	0	0	0
HF_59	0	0	0	0	0	0	0	0
HF_60	0	0	0	0	0	0	0	0
HF_72	0	0	0	0	1	0	0	0

Kingdom	Bacteria	Bacteria	Bacteria	Bacteria	Bacteria	Bacteria	Bacteria	Bacteria
Phylum	Proteobacteria	Firmicutes	Firmicutes	Firmicutes	Actinobacteria	Firmicutes	Firmicutes	Bacteroidetes
Class	Alphaproteobacteria	Erysipelotrichia	Clostridia	Clostridia	Actinobacteria	Clostridia	Clostridia	Bacteroidia
Order	Rhizobiales	Erysipelotrichales	Clostridiales	Clostridiales	Coriobacteriales	Clostridiales	Clostridiales	Bacteroidales
Family	Bradyrhizobiaceae	Erysipelotrichaceae	Clostridiaceae_	Lachnospiraceae	Coriobacteriaceae	Lachnospiraceae	Lachnospiraceae	Porphyromonadaceae
Genus	Bradyrhizobium	Erysipelotrichaceae_incertae_sedis	Alkaliphilus	Clostridium_XlVa	Enterorhabdus	Clostridium_XlVa	Clostridium_XlVa	Parabacteroides
#NAME	ZOTU_0432	ZOTU_0433	ZOTU_0434	ZOTU_0435	ZOTU_0436	ZOTU_0437	ZOTU_0438	ZOTU_0439
HF_73	0	0	0	0	0	0	0	0
HF_74	0	0	0	0	0	0	0	0
HFFG_45	0	0	0	0	0	0	0	0
HFFG_46	0	0	0	0	0	0	0	0
HFFG_47	0	0	0	0	0	0	0	0
HFFG_48	0	0	0	0	0	0	0	0
HFFG_61	0	0	0	0	0	0	0	0
HFFG_62	0	0	0	0	0	0	0	0
HFFG_63	0	0	0	0	0	0	0	0
HFFG_64	0	0	0	0	0	0	0	0
HFFG_75	0	0	0	0	5	0	0	0
HFFG_76	0	0	0	0	22	0	1	0
HFFG_77	0	0	0	0	3	0	0	0

Kingdom	Bacteria	Bacteria	Bacteria	Bacteria	Bacteria	Bacteria	Bacteria	Bacteria
Phylum	Firmicutes	Firmicutes	Firmicutes	Firmicutes	Firmicutes	Actinobacteria	Firmicutes	Actinobacteria
Class	Clostridia	Clostridia	Bacilli	Clostridia	Bacilli	Actinobacteria	Clostridia	Actinobacteria
Order	Clostridiales	Clostridiales	Lactobacillales	Clostridiales	Lactobacillales	Coriobacteriales	Clostridiales	Actinomycetales
Family	Lachnospiraceae	Peptostreptococcaceae	Enterococcaceae	Clostridiales_Incertae_Sedis_XII	Aerococcaceae	Coriobacteriaceae	Lachnospiraceae	Micrococcaceae
Genus	Clostridium_XlVa	Romboutsia	Enterococcus	Guggenheimella	Abiotrophia	Senegalimassilia	Blautia	Micrococcus
#NAME	ZOTU_0440	ZOTU_0441	ZOTU_0442	ZOTU_0443	ZOTU_0444	ZOTU_0445	ZOTU_0446	ZOTU_0447
CD_49	0	1	1	0	0	0	0	0
CD_50	0	0	3	0	0	4	0	0
CD_51	0	18	9	0	0	10	0	0
CD_52	0	0	11	0	0	1	0	0
CD_65	0	0	3	0	0	0	0	0
CD_66	0	0	20	0	0	1	0	0
CD_67	0	0	2	0	0	0	0	0
CD_68	0	0	3	0	0	0	0	0
CD_78	0	0	3	0	0	0	0	0
CD_79	0	0	0	0	0	0	0	0
CD_80	0	2	0	0	0	2	0	0
CDFG_53	0	0	0	0	0	0	0	0
CDFG_54	0	13	15	0	0	3	0	0
CDFG_55	0	9	6	0	0	5	0	0
CDFG_56	0	0	9	0	0	0	0	0
CDFG_69	0	0	8	0	0	0	0	0
CDFG_70	0	0	39	0	0	0	0	0
CDFG_71	0	0	6	0	0	0	0	0
HF_41	0	0	6	0	0	2	0	0
HF_42	0	0	50	0	0	12	0	0
HF_43	0	0	9	0	0	6	0	0
HF_44	0	0	8	0	0	3	0	0
HF_57	0	0	2	0	0	0	0	0
HF_58	0	0	3	0	0	0	0	0
HF_59	0	0	0	0	0	0	0	0
HF_60	0	0	5	0	0	0	0	0
HF_72	0	2	1	0	0	7	3	0

Kingdom	Bacteria	Bacteria	Bacteria	Bacteria	Bacteria	Bacteria	Bacteria	Bacteria
Phylum	Firmicutes	Firmicutes	Firmicutes	Firmicutes	Firmicutes	Actinobacteria	Firmicutes	Actinobacteria
Class	Clostridia	Clostridia	Bacilli	Clostridia	Bacilli	Actinobacteria	Clostridia	Actinobacteria
Order	Clostridiales	Clostridiales	Lactobacillales	Clostridiales	Lactobacillales	Coriobacteriales	Clostridiales	Actinomycetales
Family	Lachnospiraceae	Peptostreptococcaceae	Enterococcaceae	Clostridiales_Incertae_Sedis_XII	Aerococcaceae	Coriobacteriaceae	Lachnospiraceae	Micrococcaceae
Genus	Clostridium_XlVa	Romboutsia	Enterococcus	Guggenheimella	Abiotrophia	Senegalimassilia	Blautia	Micrococcus
#NAME	ZOTU_0440	ZOTU_0441	ZOTU_0442	ZOTU_0443	ZOTU_0444	ZOTU_0445	ZOTU_0446	ZOTU_0447
HF_73	0	0	0	0	0	1	0	0
HF_74	0	0	1	0	0	0	2	0
HFFG_45	0	1	1	0	0	0	0	0
HFFG_46	0	0	0	0	0	0	0	0
HFFG_47	0	0	0	0	0	0	0	0
HFFG_48	0	0	2	0	0	0	0	0
HFFG_61	0	0	3	0	0	0	0	0
HFFG_62	0	0	1	0	0	0	0	1
HFFG_63	0	0	0	0	0	0	1	0
HFFG_64	0	2	11	0	0	0	0	0
HFFG_75	0	8	12	0	0	2	0	0
HFFG_76	0	1	24	0	0	1	1	0
HFFG_77	0	0	8	0	0	0	1	0

Kingdom	Bacteria	Bacteria	Bacteria	Bacteria	Bacteria	Bacteria	Bacteria	Bacteria
Phylum	Firmicutes	Actinobacteria	Proteobacteria	Firmicutes	Actinobacteria	Actinobacteria	Firmicutes	Firmicutes
Class	Clostridia	Actinobacteria	Gammaproteobacteria	Clostridia	Actinobacteria	Actinobacteria	Clostridia	Bacilli
Order	Clostridiales	Coriobacteriales	Legionellales	Clostridiales	Actinomycetales	Coriobacteriales	Clostridiales	Bacillales
Family	Lachnospiraceae	Coriobacteriaceae	Legionellaceae	Lachnospiraceae	Micrococcaceae	Coriobacteriaceae	Peptostreptococcaceae	Paenibacillaceae_
Genus	Clostridium_XlVa	Enterorhabdus	Legionella	Clostridium_XlVa	Rothia	Enterorhabdus	Clostridium_XI	Paenibacillus
#NAME	ZOTU_0448	ZOTU_0449	ZOTU_0450	ZOTU_0451	ZOTU_0452	ZOTU_0453	ZOTU_0454	ZOTU_0455
CD_49	0	0	0	0	0	19	0	0
CD_50	0	0	0	0	0	6	0	0
CD_51	0	0	0	0	0	0	1	0
CD_52	0	0	0	0	0	12	0	0
CD_65	0	0	0	0	0	1	0	0
CD_66	0	0	0	0	0	2	0	0
CD_67	0	1	0	0	0	17	0	0
CD_68	0	0	0	0	0	0	1	0
CD_78	0	0	0	0	0	0	0	0
CD_79	0	0	0	0	0	0	0	0
CD_80	0	0	0	0	0	0	0	0
CDFG_53	0	0	1	0	0	0	0	0
CDFG_54	0	0	0	0	0	0	0	0
CDFG_55	0	0	0	0	0	5	0	0
CDFG_56	0	0	1	0	0	0	0	0
CDFG_69	0	0	0	0	0	0	0	0
CDFG_70	0	0	0	0	0	1	0	0
CDFG_71	0	0	0	0	0	1	0	0
HF_41	0	4	0	0	0	0	2	0
HF_42	0	0	0	0	0	0	0	0
HF_43	0	1	0	0	0	0	0	0
HF_44	0	0	0	0	0	0	0	0
HF_57	0	0	0	0	0	0	0	0
HF_58	0	1	0	0	0	0	0	0
HF_59	0	0	0	0	0	0	0	0
HF_60	0	0	0	0	0	0	0	0
HF_72	0	3	0	0	0	6	0	0

Kingdom	Bacteria	Bacteria	Bacteria	Bacteria	Bacteria	Bacteria	Bacteria	Bacteria
Phylum	Firmicutes	Actinobacteria	Proteobacteria	Firmicutes	Actinobacteria	Actinobacteria	Firmicutes	Firmicutes
Class	Clostridia	Actinobacteria	Gammaproteobacteria	Clostridia	Actinobacteria	Actinobacteria	Clostridia	Bacilli
Order	Clostridiales	Coriobacteriales	Legionellales	Clostridiales	Actinomycetales	Coriobacteriales	Clostridiales	Bacillales
Family	Lachnospiraceae	Coriobacteriaceae	Legionellaceae	Lachnospiraceae	Micrococcaceae	Coriobacteriaceae	Peptostreptococcaceae	Paenibacillaceae_
Genus	Clostridium_XlVa	Enterorhabdus	Legionella	Clostridium_XlVa	Rothia	Enterorhabdus	Clostridium_XI	Paenibacillus
#NAME	ZOTU_0448	ZOTU_0449	ZOTU_0450	ZOTU_0451	ZOTU_0452	ZOTU_0453	ZOTU_0454	ZOTU_0455
HF_73	0	0	0	0	0	0	0	0
HF_74	0	1	0	0	0	0	0	0
HFFG_45	0	0	0	0	0	0	0	0
HFFG_46	0	0	0	0	0	0	0	0
HFFG_47	0	0	0	0	0	0	0	0
HFFG_48	0	0	0	0	0	0	0	0
HFFG_61	0	0	0	0	0	0	0	0
HFFG_62	0	0	0	0	0	0	0	0
HFFG_63	0	0	0	0	0	0	0	0
HFFG_64	0	0	0	0	0	0	0	0
HFFG_75	0	0	0	0	0	0	0	0
HFFG_76	0	0	0	0	0	0	0	0
HFFG_77	0	0	0	0	0	0	0	0

Kingdom	Bacteria	Bacteria	Bacteria	Bacteria	Bacteria
Phylum	Firmicutes	Firmicutes	Firmicutes	Firmicutes	Firmicutes
Class	Bacilli	Bacilli	Clostridia	Bacilli	Erysipelotrichia
Order	Bacillales	Bacillales	Clostridiales	Bacillales	Erysipelotrichales
Family	Paenibacillaceae_	Bacillaceae_	Clostridiaceae_	Paenibacillaceae_	Erysipelotrichaceae
Genus	Paenibacillus	Virgibacillus	Alkaliphilus	Paenibacillus	Kandleria
#NAME	ZOTU_0456	ZOTU_0457	ZOTU_0458	ZOTU_0459	ZOTU_0460
CD_49	0	0	0	0	0
CD_50	0	0	0	0	0
CD_51	0	0	0	0	0
CD_52	0	0	0	0	0
CD_65	0	0	0	0	0
CD_66	0	0	0	0	0
CD_67	0	0	0	0	0
CD_68	0	0	0	0	0
CD_78	0	0	0	0	0
CD_79	0	0	0	0	0
CD_80	0	0	0	0	0
CDFG_53	0	0	0	0	0
CDFG_54	0	0	0	0	0
CDFG_55	0	0	0	0	0
CDFG_56	0	0	0	0	0
CDFG_69	0	0	0	0	0
CDFG_70	0	0	0	0	0
CDFG_71	0	0	0	0	0
HF_41	0	0	0	0	0
HF_42	0	0	0	0	0
HF_43	0	0	0	0	0
HF_44	0	0	0	0	0
HF_57	0	0	0	0	0
HF_58	0	0	0	0	0
HF_59	0	0	0	0	0
HF_60	0	0	0	0	0
HF_72	0	0	0	0	0

Kingdom	Bacteria	Bacteria	Bacteria	Bacteria	Bacteria
Phylum	Firmicutes	Firmicutes	Firmicutes	Firmicutes	Firmicutes
Class	Bacilli	Bacilli	Clostridia	Bacilli	Erysipelotrichia
Order	Bacillales	Bacillales	Clostridiales	Bacillales	Erysipelotrichales
Family	Paenibacillaceae_	Bacillaceae_	Clostridiaceae_	Paenibacillaceae_	Erysipelotrichaceae
Genus	Paenibacillus	Virgibacillus	Alkaliphilus	Paenibacillus	Kandleria
#NAME	ZOTU_0456	ZOTU_0457	ZOTU_0458	ZOTU_0459	ZOTU_0460
HF_73	0	0	0	0	0
HF_74	0	0	0	0	0
HFFG_45	0	0	0	0	0
HFFG_46	0	0	0	0	1
HFFG_47	0	0	0	0	1
HFFG_48	0	0	0	0	0
HFFG_61	0	0	0	0	0
HFFG_62	0	0	0	0	0
HFFG_63	0	0	0	0	0
HFFG_64	0	0	0	0	0
HFFG_75	0	0	0	0	0
HFFG_76	0	0	0	0	0
HFFG_77	0	0	0	0	0
